# Reinterpreting precipitation isotope records from Asian monsoon region through the hidden role of atmospheric updraft

**DOI:** 10.1038/s41467-026-77635-5

**Published:** 2026-09-11

**Authors:** Zhuanxia Zhang, Wusheng Yu, L. G. Thompson, Deliang Chen, Hai Cheng, Stephen Lewis, Hongchun Li, Antonios Mamalakis, Supriyo Chakraborty, Kei Yoshimura, Liangcheng Tan, Oliver Kracht, M. Midhun, Neha Trivedi, R. K. Trivedi, Zhaowei Jing, Jingyi Zhang, Rong Guo, Pengjie Ren, Qiaoyi Wang, Ziran Lei

**Affiliations:** 1 State Key Laboratory of Tibetan Plateau Earth System, Environment and Resources, Institute of Tibetan Plateau Research, Chinese Academy of Sciences, Beijing, China; 2 University of Chinese Academy of Sciences, Beijing, China; 3 Byrd Polar and Climate Research Center, The Ohio State University, Columbus, USA; 4 Department of Earth System Science, Tsinghua University, Beijing, China; 5 Institute for Carbon Neutrality, Tsinghua University, Beijing, China; 6 Institute of Global Environmental Change, Xi’an Jiaotong University, Xi’an, China; 7 Yunnan Key Laboratory of Plateau Geographical Processes & Environmental Changes, Faculty of Geography, Yunnan Normal University, Kunming, China; 8 Catchment to Reef Research Group, Centre for Tropical Water and Aquatic Ecosystem Research, James Cook University, Townsville, Australia; 9 Department of Geosciences, National Taiwan University, Taipei, Taiwan, ROC; 10 Department of Environmental Sciences, University of Virginia, Charlottesville, VA USA; 11 School of Data Science, University of Virginia, Charlottesville, VA USA; 12 Climate Variability and Prediction, Indian Institute of Tropical Meteorology, Ministry of Earth Sciences, Pune, India; 13 Institute of Industrial Science, The University of Tokyo, Kashiwa, Japan; 14 State Key Laboratory of Loess Science, Institute of Earth Environment, Chinese Academy of Sciences, Xi’an, Shaanxi China; 15 Isotope Hydrology Section, Division of Physical and Chemical Sciences, Department of Nuclear Sciences and Applications, IAEA, Vienna, Austria; 16 Department of Atmospheric Sciences, Cochin University of Science and Technology, Kochi, India; 17 Department of Applied Geology, Dr. H. S. Gour Vishwavidyalaya, Sagar, India; 18 School of Ecology and Nature Conservation, Beijing Forestry University, Beijing, China; 19 College of Tourism, Henan Normal University, Xinxiang, China

**Keywords:** Climate sciences, Hydrology

## Abstract

Speleothem oxygen isotope records (δ^18^O_s_) from the Asian monsoon region provide valuable insights into past hydroclimate variability, yet their interpretation relies on understanding the controls on modern precipitation isotopes (δ^18^O_p_). Although numerous explanations for the spatiotemporal variability of δ^18^O_p_ across this region have been proposed, the primary controls remain unresolved. Here we propose a hypothesis to explain δ^18^O_p_ variability from δ^18^O_p_ records at 46 sites along a transect spanning the Asian monsoon region. Our results show that seasonally low δ^18^O_p_ values migrate gradually westward along the east–west transect. We further demonstrate that the corresponding westward migration of atmospheric updraft, particularly convection, controls δ^18^O_p_ variations along the transect. In contrast to previous interpretations emphasizing monsoon moisture advection, our findings highlight atmospheric updraft as an important control on δ^18^O_p_ variations across the Asian monsoon region. These findings provide an alternative perspective on interpreting the climate signals preserved in Asian δ^18^O_s_ records.

## Abstract (web summary)

Using precipitation isotope data from 46 sites across the Asian monsoon region, this study reveals atmospheric updraft drives isotope variations. This finding offers an alternative lens for interpreting climate signals in Asian speleothem records.

## Introduction

The Asian monsoon region is home to more than half of the global population^1,2^. Abrupt shifts in Asian monsoon intensity and precipitation patterns have profound implications for livelihoods and socioeconomic development across the monsoon region^3–5^. Hence, understanding climate variability in this region on decadal^6^, centennial^7^, and even orbital timescales^8,9^ is a major focus of scientific research. However, long-term instrumental records remain scarce^1,10^, as extensive instrumental observation networks were not established until the mid-20th century. Fortunately, this region hosts abundant karst landforms rich in speleothems^11^. As a result, speleothem oxygen isotope (δ^18^O_s_) records from the region provide a valuable proxy for reconstructing past climate^12–19^.

Previous studies have widely applied Asian δ^18^O_s_ records as a proxy for monsoon precipitation^20–22^ and/or monsoon intensity^8,9,23–25^. However, the interpretation of Asian δ^18^O_s_ records remains strongly debated, initially motivated by discrepancies between speleothem-based reconstructions^8^ and marine-based monsoon proxies^26–28^. What is particularly striking is the contrast between the pronounced heterogeneity of precipitation across the Asian monsoon region^29–31^ and the remarkable spatial consistency of Asian δ^18^O_s_ records during the Holocene^30,32^ and the Pleistocene^6^. These apparent divergences have prompted researchers to re-examine the climate signals preserved in Asian δ^18^O_s_ records by analyzing modern precipitation δ^18^O (δ^18^O_p_) variability^33–38^.

Recently, several hypotheses have emerged to explain δ^18^O_p_ variability^39–42^. These include the inverse temperature effect, where δ^18^O_p_ and surface temperature show consistent inverse correlations^38^, and the stratiform fraction theory, where δ^18^O_p_ reflects rain type proportions^43^. However, a recent study challenged this theory, demonstrating that δ^18^O_p_ is independent of rain type proportions^44^. Many studies have also proposed the circulation effect, suggesting that δ^18^O_p_ in the Asian monsoon region reflects moisture sources^45–49^, particularly South Asian monsoon moisture^50–53^. Nevertheless, other studies have contended that seasonal shifts in moisture sources alone are insufficient to fully explain the observed δ^18^O_p_ variability^54^. These contradictions lead to a central question: what are the principal drivers controlling δ^18^O_p_ variations across the Asian monsoon region?

**Fig. 1:**
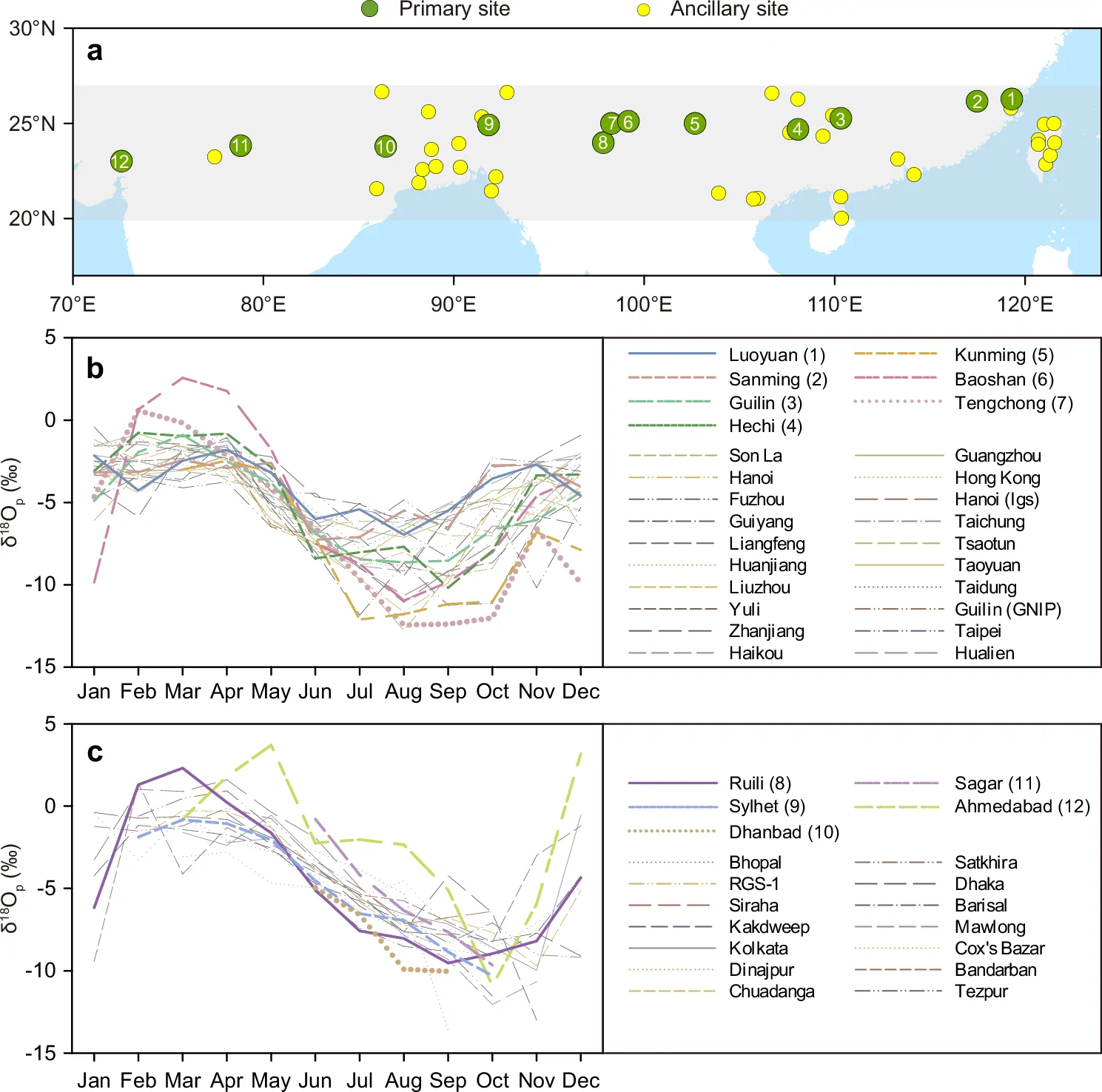
Sampling sites in the study region and seasonal variations of observed multi-year mean δ^18^O_p_ at 46 precipitation sites across the Asian monsoon region. **a** Precipitation sampling sites, green dots indicate the 12 primary sites selected for this study (Supplementary Table 1), while yellow dots represent the 34 ancillary sites (Supplementary Table 2). The gray rectangle denotes the east–west transect across the Asian monsoon region. **b**, **c** Seasonal variations of δ^18^O_p_ at 46 precipitation sites in the East Asian (**b**) and South Asian (**c**) monsoon regions. The 12 primary sites are marked by thick lines and the 34 ancillary sites by thin lines. Numbers in the brackets in (**b**, **c**) correspond to the green dots in (**a**). δ^18^O_p_ values are expressed as per mil (‰) deviations relative to VSMOW or VSMOW2.

To address this question, we compiled δ^18^O_p_ data from Global Network of Isotopes in Precipitation (GNIP) stations and other published and unpublished sources, totaling 8636 samples from 46 precipitation sites including 12 primary sites (Supplementary Table 1) and 34 ancillary sites (Supplementary Table 2) along an east–west transect of the Asian monsoon region (20–27° N, 70–125° E) (Fig. 1a, see Materials and Methods for details on the criteria for site selection). We then characterize the spatiotemporal patterns of δ^18^O_p_ along the transect. Finally, we explore the mechanisms governing δ^18^O_p_ variations across the Asian monsoon region. Our findings link this region’s δ^18^O_p_ variability to vertical atmospheric dynamics rather than to monsoon moisture advection, with important implications for interpreting the climate signals preserved in Asian δ^18^O_s_ records.

## Results and discussion

### Seasonal variations and spatial distributions of δ^18^O_p_

To investigate seasonal δ^18^O_p_ variations between the East Asian and South Asian monsoon regions, we established the east–west transect with ~ 98° E as the dividing line^55,56^. In these two regions, we analyzed seasonal δ^18^O_p_ variations at each site using multi-year mean monthly values from the 12 primary and 34 ancillary sites. We find that, for most sites in the East Asian monsoon region, the duration of lower δ^18^O_p_ values is approximately two months shorter than that for most sites in the South Asian monsoon region (Fig. 1b, c). For most sites in the East Asian monsoon region, δ^18^O_p_ continuously decreases from May to July/August, followed by an increase in August/September (Fig. 1b). In contrast, for most sites in the South Asian monsoon region, δ^18^O_p_ continues to decline from May through October (Fig. 1c). However, monsoon intensity in the South Asian monsoon region typically peaks in July and August^57–59^. Consequently, δ^18^O_p_ in the South Asian monsoon region is seasonally decoupled from monsoon intensity (see Supplementary Text 1 for details).

**Fig. 2:**
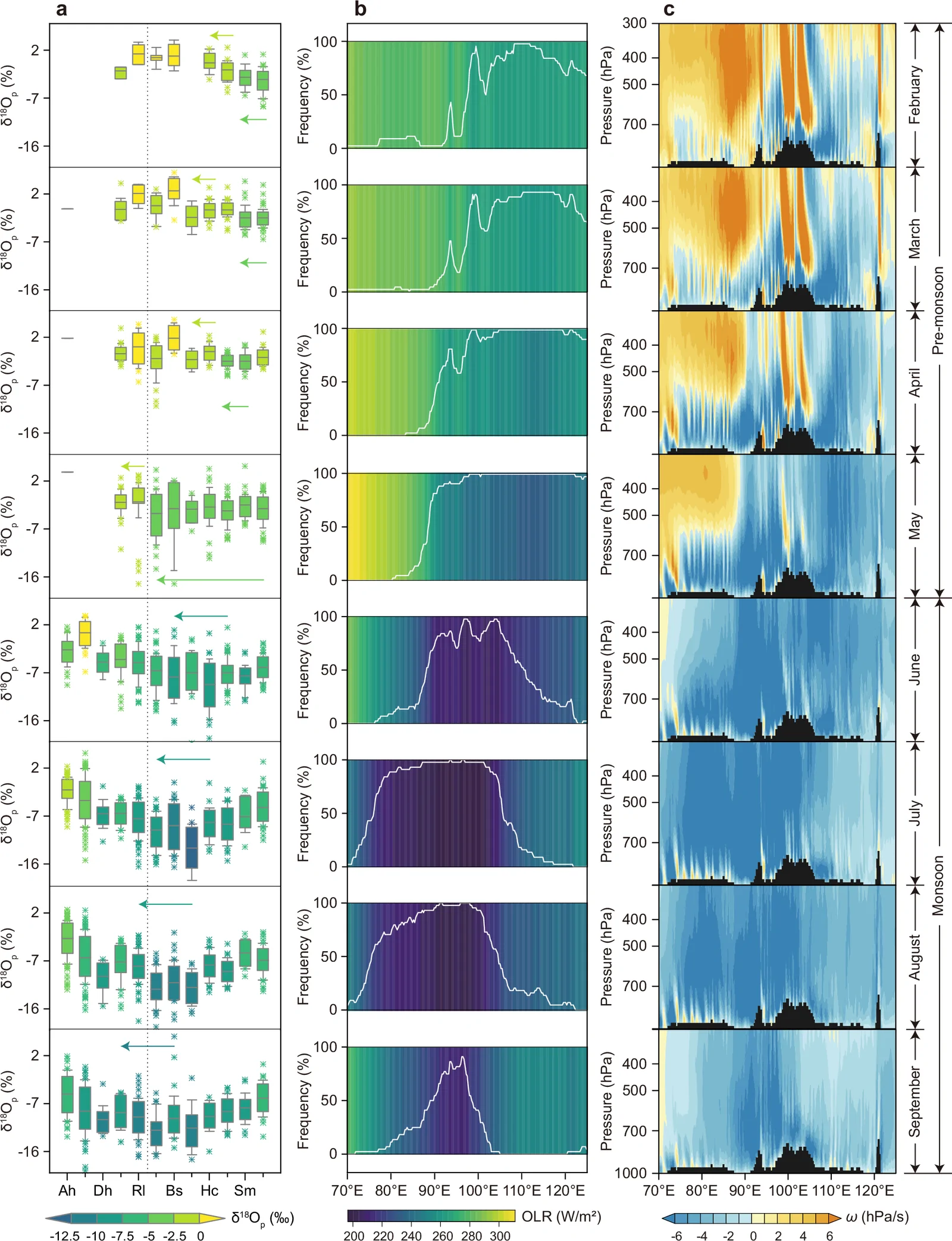
Spatial distribution of δ^18^O_p_ at the 12 primary sites and seasonal changes of atmospheric updraft along the transect during the pre-monsoon and monsoon seasons. **a** δ^18^O_p_ spatial distribution. Abbreviations: Ah (Ahmedabad), Dh (Dhanbad), Rl (Ruili), Bs (Baoshan), Hc (Hechi), and Sm (Sanming). In each box plot, the box represents the 20th–80th percentile range, whiskers extend to the 10th–90th percentiles, horizontal lines inside the boxes indicate the mean, and stars represent outliers. Horizontal arrows highlight the westward extension of the low δ^18^O_p_ zone. Dotted lines mark the dividing line of the East Asian and South Asian monsoon regions. **b** Seasonal variations in outgoing longwave radiation (OLR, shading) and the frequencies of relatively low OLR values (white line) along the transect over 1979–2022. **c** Vertical profiles of meridional mean vertical velocity (*ω*) over 1979–2022, averaged over 20–27° N.

Previous studies have often attributed δ^18^O_p_ variations in the South and East Asian summer monsoon regions to a rainout effect occurring along long-distance monsoon moisture advection from the Indian Ocean^50–53^ (hereafter denoted as the rainout effect_ad_). This mechanism would produce a west-high, east-low δ^18^O_p_ pattern along the transect. To test this hypothesis, we analyzed the spatial distribution of multi-year mean monthly δ^18^O_p_ values along the transect for each month (Fig. 2a and Supplementary Fig. 1). Indeed, during the pre-monsoon season (February–May, see Materials and Methods for seasonal classification, Supplementary Fig. 2), δ^18^O_p_ exhibits higher values in the west of the ~ 98° E dividing line and lower values in the east. However, from February to May, the seasonally lower δ^18^O_p_ values in the East Asian monsoon region expand westward, progressing from the easternmost sites (Luoyuan, Sanming) through Guilin and Hechi to the middle sites (Kunming, Baoshan, Tengchong) (Fig. 2a).

During the monsoon season (June–September), the observed pattern clearly contrasts with the hypothesis. Specifically, the lower δ^18^O_p_ zone, marked by a decrease from pre-monsoon values, migrates westward to Baoshan and Tengchong, ultimately crossing the dividing line to Ruili and Sylhet (Fig. 2a). However, the easternmost and westernmost sites retain relatively high δ^18^O_p_ values. This creates a distinct V-shaped spatial pattern, with higher δ^18^O_p_ values at both ends and lower values in the middle (Fig. 2a). Clearly, δ^18^O_p_ in the East Asian monsoon region is spatially decoupled from the monotonically decreasing pattern (an expected L-shaped or ↘ trend) that would result from the rainout effect_ad_ occurring along South Asian monsoon moisture advection (see Supplementary Text 2 for details). The observed seasonal and spatial decouplings demonstrate that South Asian monsoon moisture advection cannot fully explain δ^18^O_p_ variations.

Since explanation based solely on monsoon moisture advection is insufficient, a shift in research focus is necessary. Specifically, the approach should move from the previous advection perspective to a vertical one that emphasizes atmospheric updraft, including frontal lifting and convection, which has received less attention. In fact, this shift was driven by our findings whereby δ^18^O_p_ exhibits a stronger positive correlation with outgoing longwave radiation (OLR, an indicator of atmospheric updraft intensity: the lower the OLR, the stronger the atmospheric updraft^57,60^, see Supplementary Fig. 3 and Text 3 for details), in sharp contrast to the weaker relationships found with monsoon indices (Supplementary Text 4, Table 3, and Table 4). Notably, our definition of atmospheric updraft extends beyond convection prevalent in the monsoon season; it also includes synoptic-scale processes such as frontal lifting^61,62^ and convective instability^63,64^ in the pre- and post-monsoon seasons, particularly in the East Asian monsoon region.

### δ^18^O_p_ and atmospheric updraft

Understanding how atmospheric updraft influences δ^18^O_p_ at precipitation sites is important. During a precipitation event, as air masses are lifted to higher altitudes with lower condensation temperatures, water isotopes undergo enhanced Rayleigh fractionation, leading to lower moisture δ^18^O and consequently lower δ^18^O_p_ values^38,52^. Importantly, during the formation of the first-time raindrops, the δ^18^O of moisture experiences a substantial rainout effect (occurring within the atmospheric updraft; hereafter denoted as the rainout effect_au_). This effect lowers the δ^18^O values of the residual moisture^65,66^. When atmospheric updraft intensifies, residual moisture rises further and condenses to form second-time raindrops with even lower δ^18^O_p_ values^67^. This process continues, with each successive raindrop having lower δ^18^O_p_ values. Thus, stronger updraft increases uplift altitudes, reduces condensation temperatures, and amplifies the rainout effect_au_, further lowering δ^18^O_p_^65–67^.

To explore the influence of atmospheric updraft on δ^18^O_p_ along the east–west transect, we analyzed the spatial distributions of multi-year mean monthly outgoing longwave radiation (OLR) and vertical velocity (another indicator of atmospheric updraft intensity: negative and positive values indicate upward and downward motions, respectively) for 1979–2022 (Fig. 2b, c and Supplementary Figs. 4, 5). During the pre-monsoon season, OLR is significantly lower in the East Asian monsoon region than in the South Asian monsoon region (Fig. 2b and Supplementary Fig. 4a–d). As a consequence, a higher frequency of relatively low OLR values occurs primarily across the East Asian monsoon region (Fig. 2b). Meanwhile, a similar pattern is observed for vertical velocity, which is considerably lower in the East Asian monsoon region (Fig. 2c). Vertical velocity profiles indicate that large-scale ascent dominates in the East Asian monsoon region, whereas large-scale subsidence occurs in the South Asian monsoon region (Fig. 2c). These results demonstrate that in the pre-monsoon season local atmospheric updraft is relatively stronger in the East Asian monsoon region but weaker in the South Asian monsoon region. This spatial pattern determines δ^18^O_p_ distribution during the pre-monsoon season, with lower δ^18^O_p_ in the East Asian monsoon region compared to values in the west of the dividing line (Fig. 2a). Over time, zones with lower OLR and vertical velocity gradually expand westward, driving a corresponding westward expansion of lower δ^18^O_p_ values (Fig. 2a).

During the monsoon season, OLR and vertical velocity further decrease along the transect relative to the pre-monsoon season (Fig. 2b, c and Supplementary Fig. 4e–h), indicating stronger atmospheric updraft, particularly enhanced convection. As a result, the decrease in δ^18^O_p_ values westward along the east–west transect is more pronounced than during the pre-monsoon season (Fig. 2a). Throughout the monsoon season, lower OLR and vertical velocity values migrate consistently westward to the middle of the transect, producing a V-shaped spatial pattern of OLR and vertical velocity (Fig. 2b, c and Supplementary Fig. 4e–h). Consequently, the enhanced atmospheric updraft migrates to the middle of the transect, causing δ^18^O_p_ to decrease in the middle. In the east, where atmospheric updraft is relatively weaker, δ^18^O_p_ is relatively high, explaining the V-shaped δ^18^O_p_ pattern during the monsoon season rather than a monotonically decreasing L‑shaped one (or ↘ trend) driven by the rainout effect_ad_.

Notably, although low OLR and vertical velocity reach Sagar (located at the west of the middle of the transect) in July–August, the δ^18^O_p_ at this site does not decrease as expected. This discrepancy likely reflects spatial scale differences: δ^18^O_p_ captures site-scale variability (Fig. 2a), whereas OLR and vertical velocity represent large-scale averages (over 20–27° N, Fig. 2b, c). Therefore, comparing δ^18^O_p_ with OLR at the site scale in July–August would likely yield better agreement (Fig. 2a and Supplementary Fig. 4f, g).

By September, atmospheric updraft weakens across the East Asian monsoon region but remains strong in parts of the South Asian monsoon region (Fig. 2b, c and Supplementary Fig. 4h). Indeed, strong updraft persists into October in some parts of the South Asian monsoon regions (Supplementary Figs. 4i, 5a). This explains why δ^18^O_p_ values in the East Asian monsoon region reach a minimum in July–August, while values in the South Asian monsoon region continue decreasing through the monsoon season and into October (Fig. 1b, c).

**Fig. 3:**
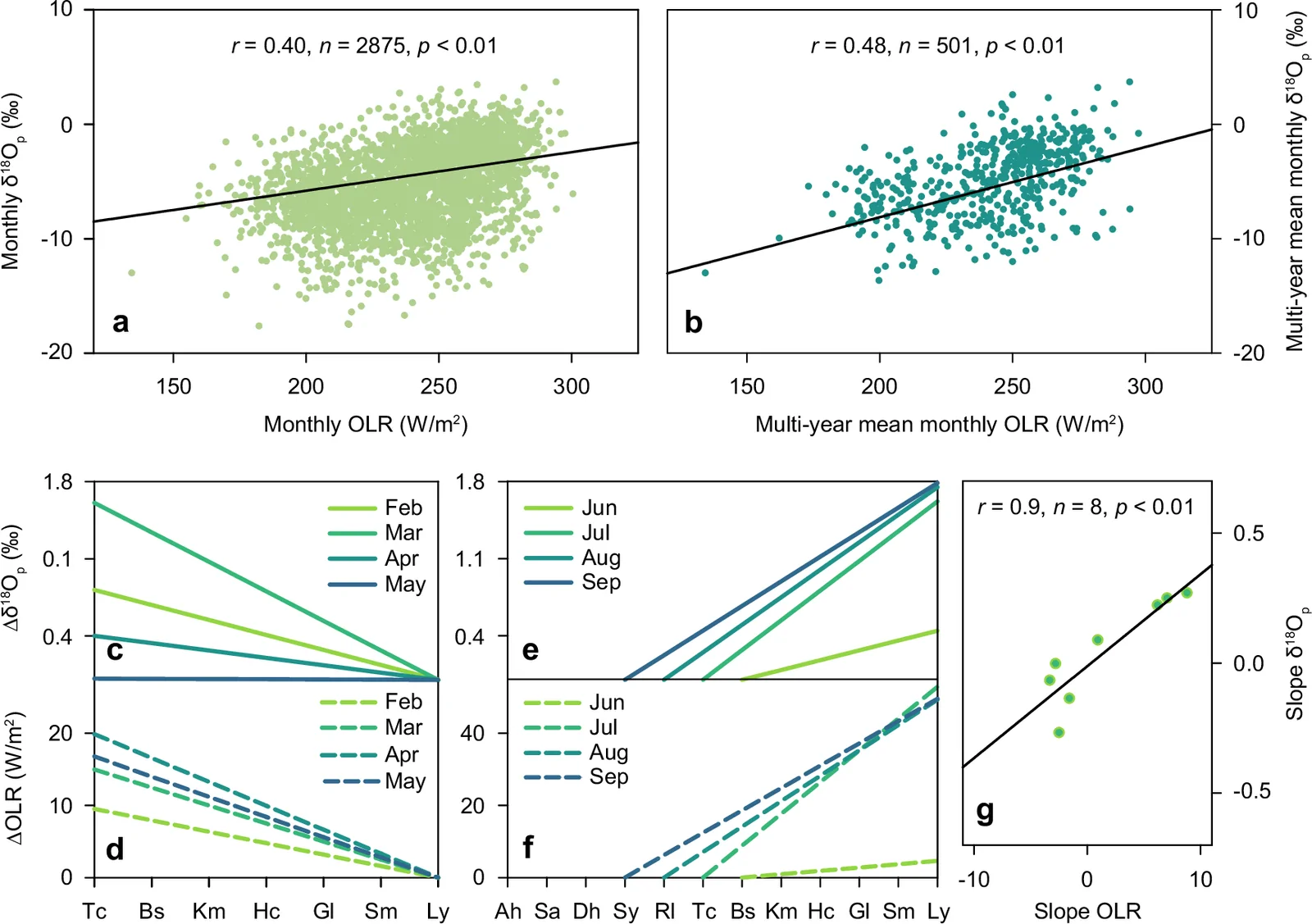
Correlations between δ^18^O_p_ and outgoing longwave radiation (OLR) along the transect. **a**,** b** Linear regressions of δ^18^O_p_ at the 46 sites across the study region against corresponding OLR on different timescales. **c**, **d** Gradients of δ^18^O_p_ and OLR from Luoyuan to Tengchong during the pre-monsoon season. **e**,** f** Gradients of δ^18^O_p_ and OLR from Luoyuan to Baoshan, Tengchong, Ruili, and Sylhet during the monsoon season. Values on the y-axes in (**c**–**f**) were calculated using the base value method (Materials and Methods). **g** Correlation between the gradients of δ^18^O_p_ and OLR during the pre-monsoon and monsoon seasons. δ^18^O_p_ values are reported as per mil (‰) deviations from VSMOW or VSMOW2. Abbreviations: Ah (Ahmedabad), Sa (Sagar), Dh (Dhanbad), Sy (Sylhet), Rl (Ruili), Tc (Tengchong), Bs (Baoshan), Km (Kunming), Hc (Hechi), Gl (Guilin), Sm (Sanming), and Ly (Luoyuan).

We further assessed the influence of atmospheric updraft on δ^18^O_p_. First, we calculated the correlation between δ^18^O_p_ and OLR (Fig. 3a, b). The results show a significant positive correlation between observed δ^18^O_p_ and the corresponding OLR at all 46 sites along the east–west transect (Fig. 3a, b). We then analyzed the gradients of δ^18^O_p_ and OLR along the transect from east to west during the pre-monsoon and monsoon seasons (see Materials and Methods, and Supplementary Text 5 for details). During the pre-monsoon season, δ^18^O_p_ from the easternmost site (Luoyuan) to Tengchong shows a negative gradient for each month (Fig. 3c and Supplementary Table 5), matching the corresponding negative gradient of OLR (Fig. 3d and Supplementary Table 5). During the monsoon season (June–September), however, both δ^18^O_p_ and OLR show positive gradients compared with the pre-monsoon season. Moreover, the coverage of the positive OLR gradient gradually extends westward from the easternmost site to increasingly distant sites over time, thereby driving the corresponding coverage of the positive δ^18^O_p_ gradient westward (Fig. 3e, f and Supplementary Table 5). Notably, although the gradients are not statistically significant for most individual pre-monsoon and monsoon months (Supplementary Table 5), a consistent contrast emerges at the seasonal scale. Specifically, the gradients remain negative throughout the pre-monsoon period, whereas they remain positive throughout the monsoon season. This consistent sign reversal highlights a pronounced shift between the two periods. We further calculated the correlation between the gradients of δ^18^O_p_ and OLR and found that δ^18^O_p_ gradients from February to September correlate strongly with OLR gradients *(r* = 0.9, *p* < 0.01; Fig. 3g). These results demonstrate that atmospheric updraft exerts a notable control over δ^18^O_p_ along the transect.

Our atmospheric updraft hypothesis contrasts with previous views that δ^18^O_p_ is primarily driven by South Asian monsoon moisture advection^50–53^. Instead, we show that atmospheric updraft plays a very important role, as it offers a more robust explanation for the observed spatiotemporal patterns across the Asian monsoon region.

**Fig. 4:**
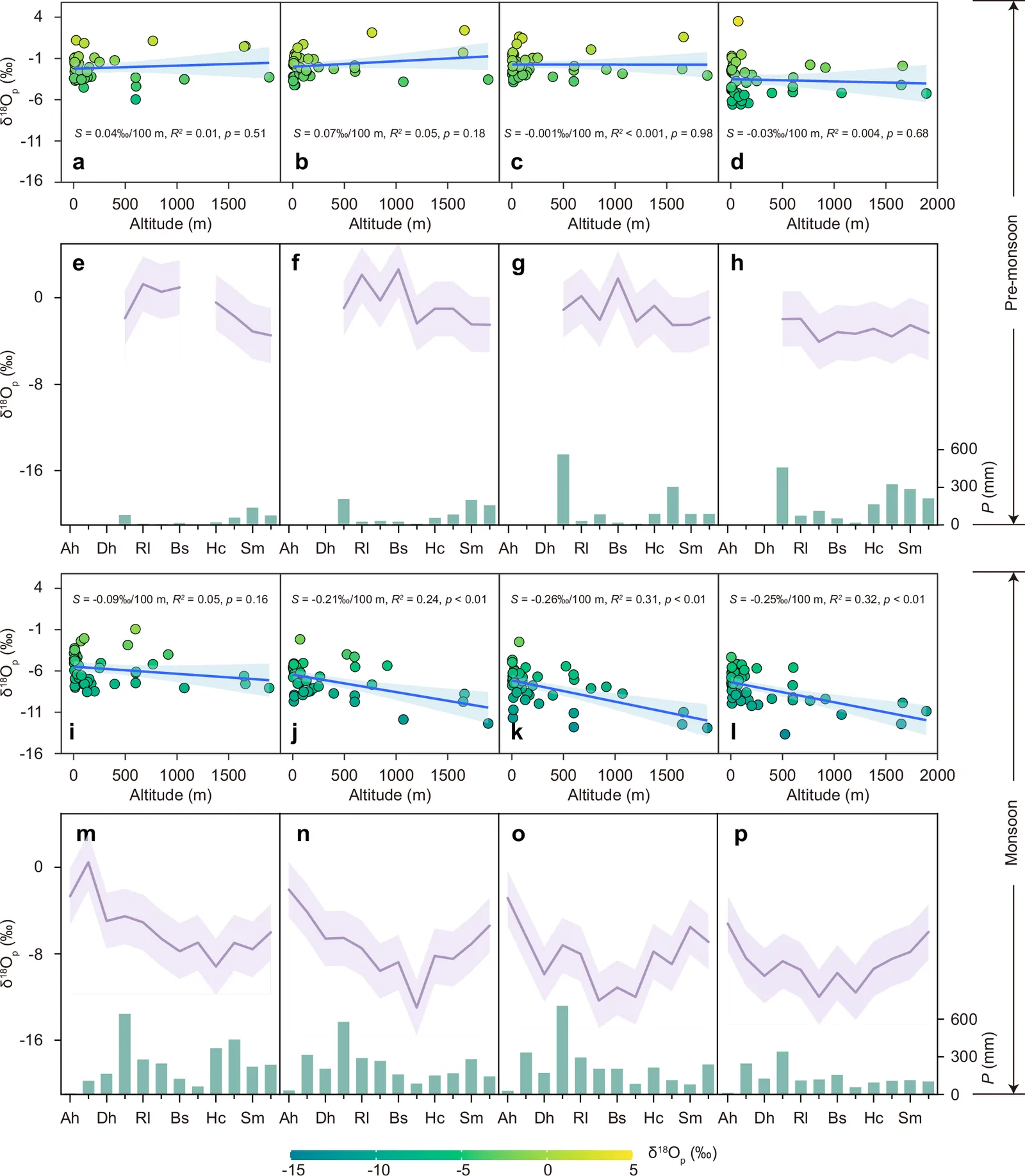
Seasonal variations in δ^18^O_p_–altitude correlations at the 46 sites and spatial changes of δ^18^O_p_ and precipitation amount at the 12 primary sites. **a**–**d**, **i**–**l** Linear regressions of δ^18^O_p_ at the 46 sites across the study region against altitude, fitted for each month: February (**a**), March (**b**), April (**c**), May (**d**), June (**i**), July (**j**), August (**k**), and September (**l**). The blue band around the regression line indicates the 95% confidence interval. *S* denotes the slope of the regression line. **e**–**h**, **m**–**p** Multi-year mean monthly δ^18^O_p_ (line) with ±1σ standard deviation (shading) and precipitation amount distributions during the pre-monsoon and monsoon seasons: February (**e**), March (**f**), April (**g**), May (**h**), June (**m**), July (**n**), August (**o**), and September (**p**). Abbreviations: Ah (Ahmedabad), Dh (Dhanbad), Rl (Ruili), Bs (Baoshan), Hc (Hechi), and Sm (Sanming). δ^18^O_p_ values are expressed as per mil (‰) deviations relative to VSMOW or VSMOW2.

One might argue that terrain could influence δ^18^O_p_, given the abundance of high mountains in the middle of the transect. Theoretically, increasing altitude is expected to result in gradually lower δ^18^O_p_ values, implying a negative correlation between the two. To assess the potential influence of terrain on δ^18^O_p_, we examined whether δ^18^O_p_ values were consistently negatively correlated with altitude on a monthly basis. The results show that correlations is time transgressive, with only significant negative correlations occurring in the monsoon seasons (Fig. 4a–d, i–l), reflecting the westward expansion or migration of low δ^18^O_p_ from the pre-monsoon to monsoon seasons. This temporal variability stands in clear contrast to the inherent stability of the terrain. In addition, classification of sites by Köppen-Geiger climate zone^68^ (Supplementary Fig. 6) reveals inconsistent δ^18^O_p_–altitude correlations, with both positive and negative relationships observed. Classification by UNEP-WCMC altitude ranges^69^ (Supplementary Fig. 7) shows that the negative correlations were not statistically significant (*P* > 0.05). These findings demonstrate that terrain exerts a limited influence on δ^18^O_p_ variations across the Asian monsoon region.

In addition, the amount effect (higher precipitation amount leads to lower δ^18^O_p_) has been proposed as a critical control on δ^18^O_p_ variations in this region. However, the spatial patterns of precipitation amount along the transect (Fig. 4e–h, 4m–p), the correlation analyses between δ^18^O_p_ and precipitation amount (Supplementary Fig. 8a, b), and a comparison of two linear regression models all indicate that precipitation amount plays only a secondary role in modulating δ^18^O_p_ across this region (see Supplementary Text 6 and Supplementary Table S6 for details). Moreover, this secondary influence of the amount effect on δ^18^O_p_ is most likely attributable to the role of atmospheric updraft (Supplementary Fig. 8c, d) (see Supplementary Text 6 for details).

### Mechanisms of atmospheric updraft changes

Since variations in atmospheric updraft determine δ^18^O_p_ patterns along this transect, what dynamics drive the westward migration of updraft? To investigate the underlying mechanism, we turn to the large-scale circulation systems. In the Asian monsoon region, precipitation is influenced not only by monsoonal circulation but also by the combined effects of the Intertropical Convergence Zone (ITCZ)^70,71^, the Indo-Pacific Warm Pool (IPWP)^72,73^, and the western Pacific subtropical high (WPSH)^74–77^. The ITCZ is a deep convective cloud band in the tropics that generates low-pressure convergence and initiates large-scale uplift^70,71^. This uplift is further intensified by the IPWP, the world’s largest warm pool, which can induce strong atmospheric updraft^72,73^. Meanwhile, the WPSH, a large-scale anticyclonic circulation system in the western Pacific^74^, plays a key role in shaping precipitation patterns across the East Asian monsoon region^75–77^. Therefore, variations in atmospheric updraft along this transect may be associated with these climate systems.

**Fig. 5:**
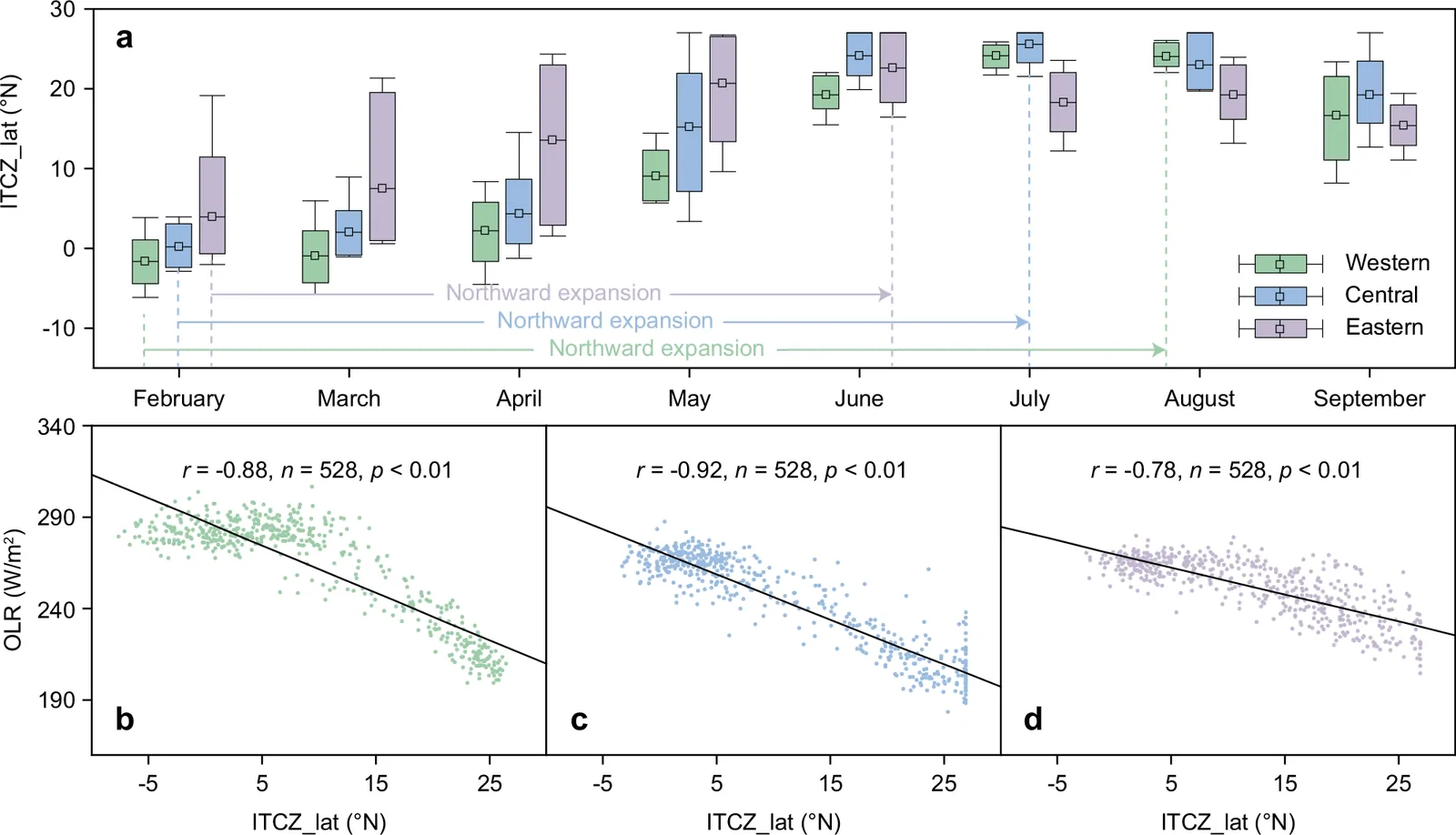
Intertropical Convergence Zone (ITCZ) northern boundary location within the respective longitude ranges of the three subregions, and correlations between outgoing longwave radiation (OLR) in these subregions and the corresponding ITCZ northern boundary location during 1979–2022. **a** ITCZ northern boundary location (ITCZ_lat) during the pre-monsoon and monsoon seasons. Western, Central, and Eastern represent the three subregions: Western (70–95° E), Central (95–105° E), and Eastern (105–125° E). In each box plot, the box represents the 15th–85th percentile range, and whiskers extend to the 5th–95th percentiles. Inside each box, the mean is shown as both a hollow square and a horizontal line. **b**–**d** Correlations between OLR in the three subregions and ITCZ northern boundary location (1979–2022, all months combined): Western (**b**), Central (**c**), and Eastern (**d**).

**Fig. 6:**
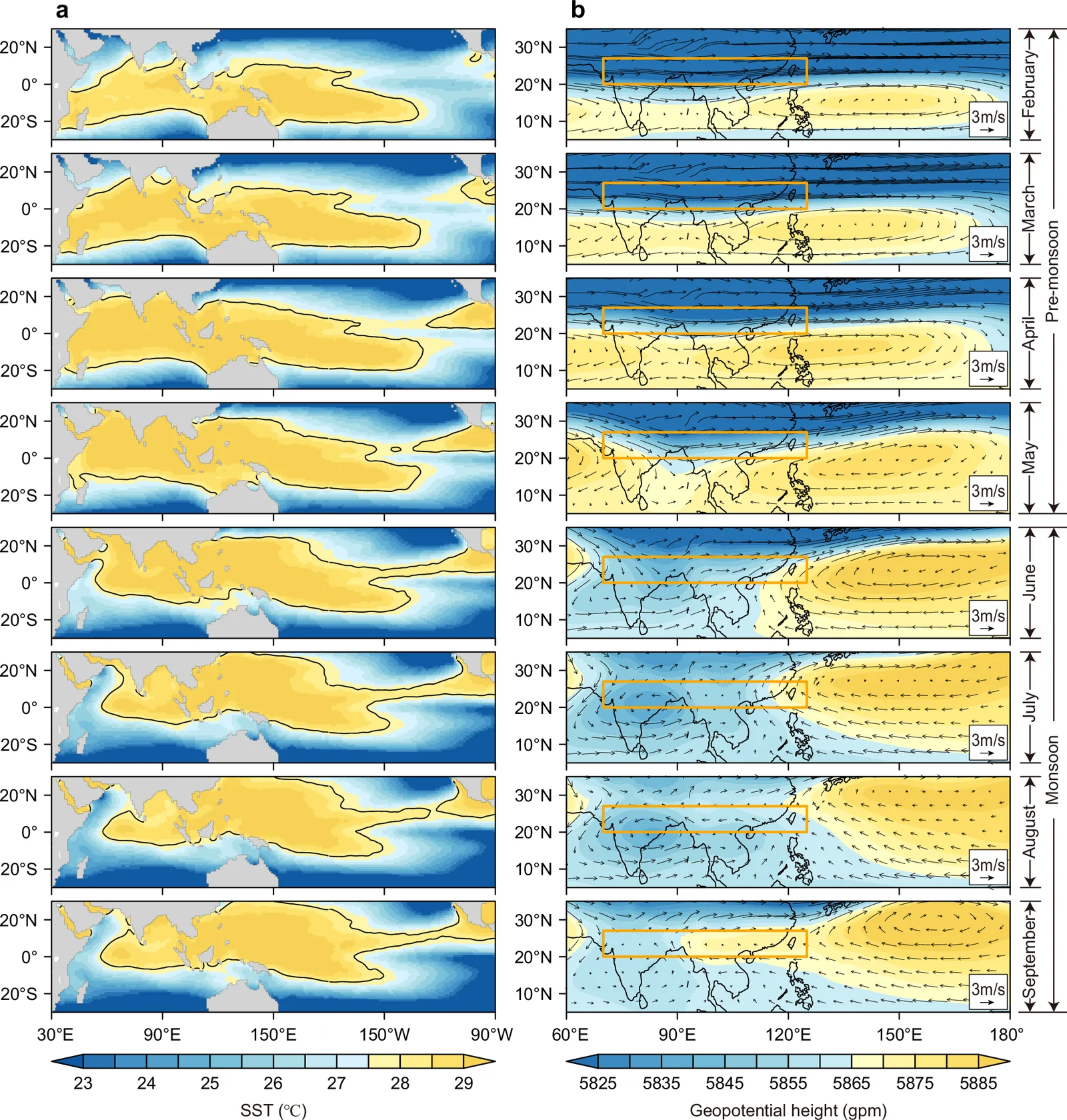
Spatial distributions of monthly sea surface temperatures (SSTs), 500 hPa geopotential height, and wind field over 1979–2022 during the pre‑monsoon and monsoon seasons. **a** Spatial distributions of SSTs. The black line indicates the 28 °C SST, marking the extent of the Indo-Pacific Warm Pool. **b** Spatial distributions of 500 hPa geopotential height and wind field. Shading represents geopotential height, arrows indicate the wind field, and the orange rectangle marks the study region.

**Fig. 7:**
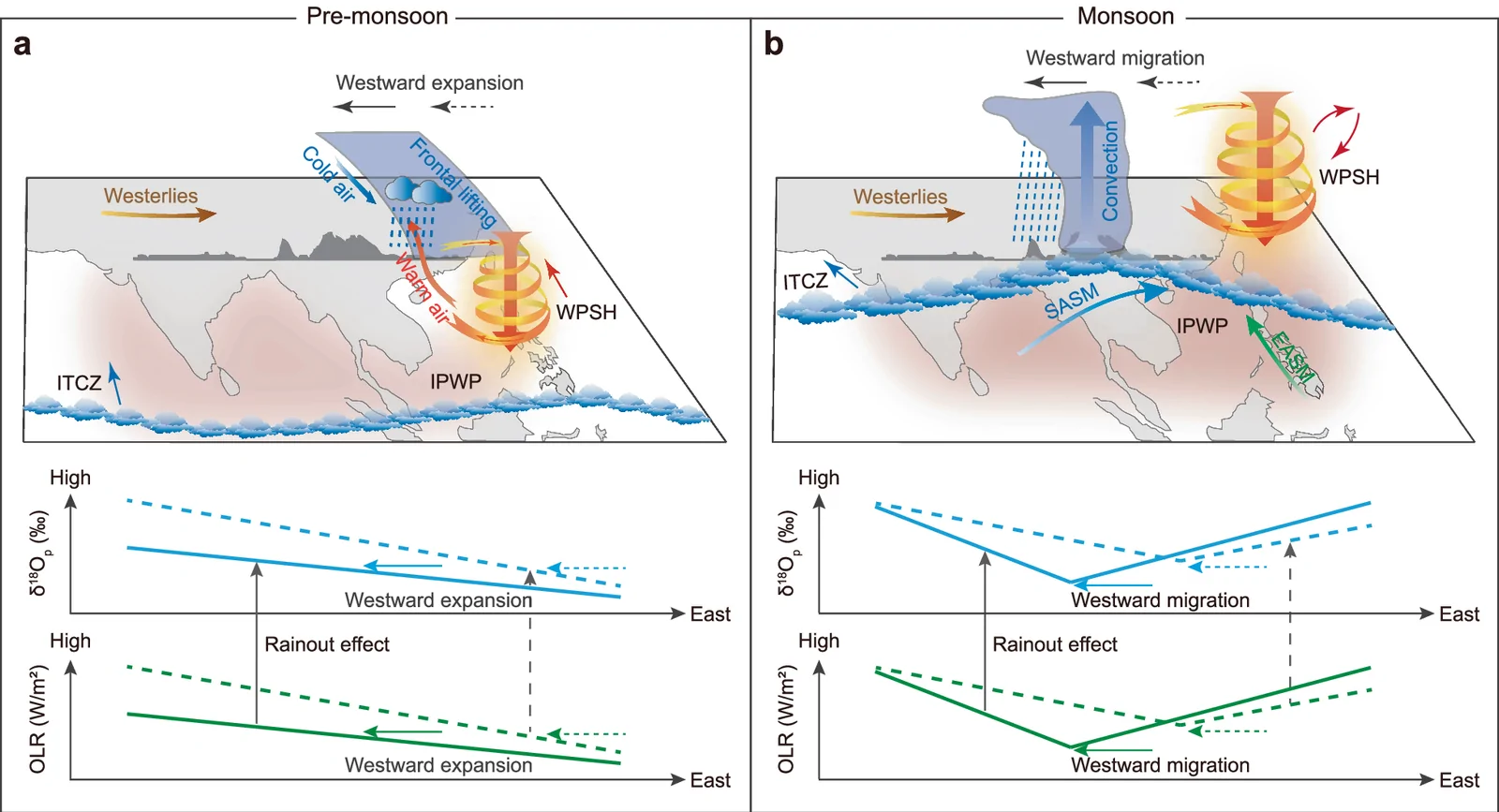
Schematic illustration of mechanisms controlling the spatial distributions of δ^18^O_p_ along the transect during the pre-monsoon and monsoon seasons. **a** Pre-monsoon season. **b** Monsoon season. Horizontal solid arrows indicate the final state (May during the pre-monsoon season or September during the monsoon season) of stronger atmospheric updraft and lower δ^18^O_p_ migrating westward, while horizontal dashed arrows represent the prior state (February during the pre-monsoon season or June during the monsoon season). The deep gray shading in the topographic map shows the terrain profile along the east–west transect. Abbreviations: ITCZ, Intertropical Convergence Zone; IPWP, Indo-Pacific Warm Pool; WPSH, Western Pacific subtropical high; SASM, South Asian summer monsoon; EASM, East Asian summer monsoon; OLR, outgoing longwave radiation. The continuous band of piled‑up blue clouds denotes the ITCZ. The grayish‑red shaded area indicates the WPSH. The orange downward spiral arrow, with a central vertical red downward arrow, denotes the WPSH. The blue and red arrows adjacent to ITCZ and WPSH indicate their movement directions during the pre-monsoon and monsoon seasons. Figure created with Adobe Illustrator 2024.

To explore this link, we analyzed the spatial distributions of the multi-year mean monthly ITCZ (Fig. 5 and Supplementary Figs. 9 and 10), IPWP (Fig. 6a and Supplementary Fig. 11a), and WPSH (Fig. 6b and Supplementary Fig. 11b) over 1979–2022. During the pre-monsoon season (February–May), the ITCZ moves gradually northward (Fig. 5a and Supplementary Fig. 9a–d). As it remains south of the study region from February to April, its influence is very weak (Fig. 5a and Supplementary Fig. 9a–c). In May, however, it reaches the East Asian monsoon region and induces a relatively strong atmospheric updraft in the region (Fig. 5a and Supplementary Fig. 9d). Concurrently, the IPWP strengthens and expands northward from February to May, eventually reaching the southern coast of China. These shifts produce a relatively strong atmospheric updraft in the East Asian monsoon region (Fig. 6a). In parallel, the WPSH shifts northward, with its ridge moving from approximately 15° N in February to around 19° N in May (Fig. 6b). The southerly winds along the western flank of the WPSH transport warm, moist air from the IPWP region northwards into the East Asian monsoon region. When this warm, moist air collides with southward-flowing cold air from the mid-latitudes, an inclined updraft forms along the frontal zone, a process known as frontal lifting^75–77^. This frontal lifting further enhances atmospheric updraft in the region. Hence, the combined influence of the ITCZ, IPWP, and WPSH results in a substantially stronger atmospheric updraft in the East Asian monsoon region than in the South Asian monsoon region during the pre-monsoon season, particularly in May (Fig. 2b, c and Fig. 7a). This enhanced updraft mechanistically explains the lower δ^18^O_p_ values in the East Asian monsoon region compared to the South Asian monsoon region during this season (Fig. 7a). Importantly, from February to May, as the IPWP and WPSH intensify and shift northward, their influence also extends westward (Fig. 6). This westward expansion contributes to the gradual strengthening of atmospheric updraft and its westward expansion along the transect (Fig. 7a), driving the westward expansion of low δ^18^O_p_ values (Fig. 7a).

In comparison, during the monsoon season (June to August), the WPSH ridge moves eastward and shifts northward^77^, from approximately 23° N in June to 30° N in August (Fig. 6b). As a result, its influence on atmospheric updraft along the transect weakens. Meanwhile, both the ITCZ and IPWP continue to migrate northward (Figs. 5a, 6a, and Supplementary Fig. 9–g), leading to a substantial increase in atmospheric convection across the Asian monsoon region (Fig. 2b, c and Supplementary Fig. 4e–g). Importantly, as the ITCZ progresses northward, it also gradually expands westward, reaching the western Bay of Bengal by August (Fig. 5a). Consequently, the ITCZ drives the gradually strengthening convection further westward along the transect. The influence of the ITCZ causes convection to become stronger in the middle of the transect (Fig. 7b), producing the V-shaped pattern of δ^18^O_p_ values along the east–west transect (Fig. 7b).

In September, although the influence of the ITCZ weakens to some extent in the East Asian monsoon region, it remains relatively active in the South Asian monsoon region (Fig. 5a and Supplementary Fig. 9h). Consequently, convection remains relatively strong in the South Asian monsoon region (Fig. 2b, c and Supplementary Fig. 4h). During this period, easterly winds driven by the westward expansion of the WPSH collide with the moisture from the Indian Ocean (Fig. 6b), thereby strengthening atmospheric updraft in the South Asian monsoon region. These processes lead to the lower δ^18^O_p_ values in September than in August across the South Asian monsoon region (Fig. 1c). It should be noted that September δ^18^O_p_ values in the South Asian monsoon region are not lower than those in the East Asian monsoon region. This finding may be attributed to the generally higher background isotopic values in the former compared to the latter^78,79^.

### Implications and outlook

Our study reveals that the westward migration of low δ^18^O_p_ values along the east–west transect tracks the corresponding westward migration of atmospheric updraft, producing δ¹⁸Oₚ patterns that mirror the spatial patterns of updraft intensity. We therefore propose a vertical perspective that identifies atmospheric updraft as an important control on δ^18^O_p_ variations across the Asian monsoon region. Our findings offer insights into the question of which climate signals are preserved in Asian δ^18^O_s_ records, suggesting that these records may capture variations in atmospheric updraft intensity. Furthermore, the δ^18^O_p_–updraft relationship provides an observational constraint for evaluating isotope-enabled General Circulation Models (GCMs), particularly their ability to reproduce the isotopic imprint of atmospheric updraft—a process that warrants greater attention in future model–observation comparisons. More broadly, these findings may also have implications for δ^18^O_p_ variability in other monsoon systems, where similar updraft dynamics may exert comparable controls.

We acknowledge that δ^18^O_p_ variations arise from the interplay of multiple factors, including vertical and horizontal processes, topography, and meteorological conditions (e.g., temperature and precipitation). We thus advocate incorporating these multiple factors to develop a holistic (rather than single-effect) model for understanding δ^18^O_p_ variability not only across the Asian monsoon region but also globally. To better interpret Asian δ^18^O_s_ records, in-cave factors (e.g., dripwater δ^18^O^80–85^, selective recharge^86,87^) should also be considered, as they may attenuate, lag, or even decouple the δ^18^O_p_ signal before being archived. In addition, to further enhance the reliability of interpretation, future work should adopt a link perspective, integrating δ^18^O_p_ with dripwater δ^18^O^11^.

## Methods

### Selection of study region

Previous studies have suggested that δ^18^O in speleothems (δ^18^O_s_) along the South Asian monsoon moisture channel gradually decrease due to the rainout effect_ad_ occurring along the monsoon moisture advection^50,53^. However, significant latitudinal variations in δ^18^O_s_ may also result from pronounced latitude effects (δ^18^O systematically decreases from tropical to polar zones) and/or temperature effects (δ^18^O decreases with lower condensation temperatures)^88^. These confounding factors make the South Asian monsoon moisture channel unsuitable for isolating the primary controls on δ^18^O in precipitation (δ^18^O_p_) across the Asian monsoon region. To minimize these influences, we selected a transect extending from the east coast of China to the west coast of India (70–125° E) within a latitudinal range of 20–27° N (Fig. 1a).

### Selection criteria for observed δ^18^O_p_ data

We retrieved the observed δ^18^O_p_ data from three sources, including data from the GNIP dataset, published studies, and two unpublished sites (Hechi and Baoshan). Details on sample collection, storage, and measurement are available in ref. ^38^. We compiled δ^18^O_p_ observations along the east–west transect using the following selection criteria: (1) the dataset must cover the period 1979–2022; and (2) the sample size must be at least 50 (*n* ≥ 50) for daily sampling sites and at least 30 (*n* ≥ 30) for monthly sampling sites. Notably, although Hong Kong’s observations date back to 1961, we limited the analysis to the period 1979–2022 for consistency with the other sites. A total of 46 sites met these criteria (Supplementary Table 1 and Table 2). Among these 46 sites, seven have both daily and monthly δ^18^O_p_ data. Therefore, when counting the number of sites, these seven sites will not be counted repeatedly.

To further reduce possible latitude effects on δ^18^O_p_, we identified 12 primary sites (Supplementary Table 1) within the 46 sites, based on the following criteria: (1) observation sites located within a narrow latitudinal band of 1° ± 1.5°; (2) availability of high-precision (daily) δ^18^O_p_ observations; and (3) maximum possible observation duration. The remaining 34 sites served as ancillary sites to validate the spatial patterns of the primary sites (Supplementary Table 2).

Given that circulation fields and precipitation amount in the Asian monsoon region exhibit distinct seasonal variations^52^, the spatial patterns of δ^18^O_p_ values at the 12 primary sites were analyzed across different seasons. The seasons are classified based on this criterion: the pre-monsoon (February–May), monsoon (June–September), and post-monsoon (October–January) seasons. The post-monsoon season contributes minimally to annual precipitation (Supplementary Fig. 2) and was therefore not considered further (Supplementary Fig. 1).

### Meteorological Data

Meteorological datasets used in this study include observed precipitation records, reanalysis data, and monsoon indices. Observed precipitation amount data were obtained from the China Meteorological Administration National Meteorological Science Data Center, the National Oceanic and Atmospheric Administration, and the GNIP dataset. These precipitation amount data were used to analyze the proportions of precipitation during the different seasons to the annual precipitation and to analyze the spatial distributions of precipitation amount during the pre-monsoon and monsoon seasons.

The study utilized the ERA5 reanalysis data (0.25°×0.25° resolution) provided by the European Centre for Medium-Range Weather Forecasts (ECMWF). The ERA5 reanalysis data on single levels are available from ref. ^89^. The ERA5 reanalysis data on pressure levels are available from ref. ^90^. Among them, vertical velocity (*ω*) and outgoing longwave radiation (OLR) were used to quantify the intensity of atmospheric updraft. The lower the *ω* and OLR, the stronger the atmospheric updraft. We used specific humidity combined with meridional and zonal winds to calculate the moisture flux. The geopotential height was used to assess the western Pacific subtropical high intensity. Cloud cover and precipitation were used to delineate the Intertropical Convergence Zone (ITCZ). In addition, sea surface temperature (SST) data (1° × 1° resolution) provided by HadISST1^91^ were used to evaluate Indo-Pacific Warm Pool (IPWP) variability.

### Probabilistic tracking of the ITCZ

To track the ITCZ position, we applied a multivariate probabilistic framework developed by refs. ^92,93^. The combination of multiple variables thereby enhances the robustness of the tracking approach. Specifically, we utilized total cloud cover and precipitation amount—proxies for deep convection—to track the multi-year mean monthly ITCZ position during 1979–2022. Following the methodology, we calculated zonal-mean values for both variables within overlapping longitudinal windows of width *w* = 15°. The ITCZ locations were identified as the latitudes corresponding to the upper 15% (probability of non-exceedance *α* = 85%) of the joint distribution of zonal precipitation and zonal total cloud cover. Finally, the empirical probability density function (PDF) of the ITCZ location was estimated based on these identified points for each longitudinal window.

To quantify the location of the ITCZ and its dynamical linkage with atmospheric updraft, we first divided the transect into three subregions: Western (70–95° E), Central (95–105° E), and Eastern (105–125° E). Using the empirical probability density functions (PDFs) derived from the multivariate probabilistic framework described above, we extracted the monthly northern boundary of the ITCZ within the longitudinal range of each subregion over 1979–2022. For a given month (e.g., January 1979), the procedure was as follows: for each longitudinal grid point within a subregion, we scanned southward from 27° N to 27° S and recorded the latitude where the PDF value first exceeded 0 as the ITCZ’s northern boundary for that longitude and month. After extracting the northern boundaries for all longitudes that had a valid boundary in that month, we calculated the arithmetic mean of these latitudes across all longitudes within each subregion. This mean constitutes the ITCZ’s northern boundary index for that subregion for that month. Repeating this procedure for every month from January 1979 to December 2022 yielded a continuous time series of the ITCZ’s northern boundary for each of the three subregions over the entire period. Finally, we calculated the correlation between OLR within each subregion and the corresponding northern boundary of the ITCZ. The significant positive correlation between the ITCZ’s northern boundary and OLR (Fig. 5b–d) supports a robust dynamical linkage between the ITCZ and atmospheric updraft along this transect.

### Calculation of slopes of δ^18^O_p_ and OLR values

To analyze the gradients of δ^18^O_p_ and OLR values along the transect from east to west during the pre-monsoon and monsoon seasons, we standardized the δ^18^O_p_ and OLR values at the 12 primary sites using the base value method (see Supplementary Text 5 for details).

## Supplementary information

Supplementary Information

Transparent Peer Review file

## Source data

Source Data

## Data Availability

All datasets used in this study are publicly available. The δ^18^O_p_ data at the Baoshan and Hechi sites are available in the Figshare repository (https://doi.org/10.6084/m9.figshare.33319041)^94^. The δ^18^O_p_ data at other sites are obtained from the GNIP dataset (https://iaea.org/services/networks/gnip) and the published literature (see Supplementary Table 1 and Table 2). Observed precipitation amount data were obtained from the China Meteorological Administration National Meteorological Science Data Center (https://data.cma.cn/) and the National Oceanic and Atmospheric Administration (https://www.ncei.noaa.gov/maps/daily/), and the GNIP dataset (https://iaea.org/services/networks/gnip). The ERA5 reanalysis data on single levels are available from https://cds.climate.copernicus.eu/cdsapp#!/dataset/reanalysis-era5-single-levels-monthly-means?tab=form. The ERA5 reanalysis data on pressure levels are available from https://cds.climate.copernicus.eu/cdsapp#!/dataset/reanalysis-era5-pressure-levels-monthly-means?tab=form. The sea surface temperature (SST) data are available from https://www.metoffice.gov.uk/hadobs/hadisst. Source data are available from the Supplementary Data files. Source data are provided in this paper.

## Code availability

The computational codes used for data analysis and visualization are freely accessible. Data processing was performed using CDO (Climate Data Operators, Version 1.9.3, https://code.mpimet.mpg.de/projects/cdo) and NCL (NCAR Command Language, Version 6.6.2, https://www.ncl.ucar.edu).
