# Supplementary material for: Reinterpreting precipitation isotope records from Asian monsoon region through the hidden role of atmospheric updraft
Source: Nat Commun. 2026 Sep 11;17:10728. doi: 10.1038/s41467-026-77635-5 (PMC13658188; doi:10.1038/s41467-026-77635-5)
Supplement: Supplementary file 1 [file 41467_2026_77635_MOESM1_ESM.pdf]

Supplementary Information for

Reinterpreting precipitation isotope records from Asian monsoon region through the hidden role of atmospheric updraft

Zhuanxia Zhang, Wusheng Yu*, L.G. Thompson, Deliang Chen, Hai Cheng, Stephen Lewis, Hongchun Li, Antonios Mamalakis, Supriyo Chakraborty, Kei Yoshimura, Liangcheng Tan, Oliver Kracht, M. Midhun, Neha Trivedi, R.K. Trivedi, Zhaowei Jing, Jingyi Zhang, Rong Guo, Pengjie Ren, Qiaoyi Wang & Ziran Lei

Emial: yuws@itpcas.ac.cn (W. Yu)

Included Materials:

Supplementary Texts 1 to 6

Supplementary Figures 1 to 11

Supplementary Tables 1 to 6

Text S1. Seasonal decoupling between $\delta^{18}O_p$ and monsoon intensity

We investigated seasonal $\delta^{18}O_p$ variations across the East Asian and South Asian monsoon regions. We find that in the South Asian monsoon region, there exists a marked seasonal decoupling between $\delta^{18}O_p$ and the intensity of the South Asian monsoon (Here, monsoon intensity refers to the strength of atmospheric circulation (i.e., wind fields), rather than local precipitation amount). Specifically, $\delta^{18}O_p$ in the South Asian monsoon region continuously decreases from May to October (Fig. 1c), while the peak South Asian summer monsoon moisture transport occurs in July–August[1-3]. This disconnect implies that although $\delta^{18}O_p$ tracks South Asian monsoon moisture transport during the early summer, it does not do so directly from late summer to mid-autumn.

Text S2. Spatial decoupling between $\delta^{18}O_p$ and isotopic depletion during advection

Previous studies have identified the Indian Ocean as the dominant moisture source for the South and East Asian summer monsoon rainfall, both in the modern climate[3,4] and during the last deglaciation[5,6], although additional contributions from the Pacific Ocean and the South China Sea influence the East Asian monsoon region[3]. As a consequence, $\delta^{18}O_p$ variations in these regions have often been linked to the rainout effect occurring along long-distance monsoon moisture advection from the Indian Ocean[3-6] (occurring within the advection; hereafter denoted as the rainout effect$_{ad}$). Should this be the case, $\delta^{18}O_p$ is expected to exhibit a high in the west, low in the east

pattern along this east–west transect. This is because the heavy isotopes in moisture from the Indian Ocean are gradually depleted by the rainout $effect_{ad}$ occurring along the monsoon moisture advection. However, in our study $\delta^{18}O_p$ values increase rather than decrease from the middle of the transect to the easternmost sites during each month of the monsoon season (June–September) (Fig. 2a). Our analysis reveals a strong spatial decoupling between $\delta^{18}O_p$ and the gradual isotopic depletion caused by the rainout $effect_{ad}$ occurring along the South Asian monsoon moisture advection.

Text S3. Correlations between OLR and vertical velocity

To test whether OLR is a robust proxy for atmospheric updraft intensity, we examined correlations between OLR and vertical velocity. In this region, OLR exhibits significant positive correlations with vertical velocity at 300, 400, and 500 hPa (Supplementary Fig. 3). Because negative vertical velocity indicates upward motion, these positive correlations indicate that lower OLR corresponds to stronger atmospheric updraft. These results confirm that OLR can be used as an effective indicator of atmospheric updraft intensity in this study region.

Text S4. Correlations between $\delta^{18}O_p$ and monsoon indices

We examined correlations between $\delta^{18}O_p$ at five sites (Hong Kong, Guangzhou, Kunming, Dhaka, and Sylhet) with long-term (>10-year) records and various monsoon intensity indices. We used the South Asian Summer Monsoon Index (SASMI) (http://lijianping.cn/dct/page/65540) and the Indian Monsoon Index (IMI)[7,8], as well as the East Asian Summer Monsoon Index (EASMI)

(http://lijianping.cn/dct/page/65540) and the Northwestern Pacific Monsoon Index (WNPMI)[7,8]. In most cases, correlations between $\delta^{18}O_p$ and monsoon indices on both monthly and annual timescales are either weakly negative or non-significant, and in some instances, positive correlations even appear unexpectedly (Supplementary Table 3 and Table 4). In contrast, $\delta^{18}O_p$ across both timescales consistently shows significant positive correlations with outgoing longwave radiation (OLR), an indicator of atmospheric updraft (convection) intensity (the lower the OLR, the stronger the atmospheric updraft)[9,10]. These results further support that the South Asian monsoon moisture advection (or the East Asian monsoon moisture advection) has a limited influence on $\delta^{18}O_p$.

Text S5. Calculation of slopes of $\delta^{18}O_p$ and OLR values

To analyze the gradients of precipitation isotope ($\delta^{18}O_p$) and outgoing longwave radiation (OLR) values along the transect from east to west during the pre-monsoon and monsoon seasons, we standardized the $\delta^{18}O_p$ and OLR values from 12 primary sites using the base value method. Given the inconsistency in the lengths of observational $\delta^{18}O_p$ records across different sites, and to ensure temporal consistency with the calculation of the OLR gradient over the period 1979–2022, we utilized IsoGSM2-simulated $\delta^{18}O_p$ data[11] spanning the same interval (1979–2022) to compute the $\delta^{18}O_p$ gradient along the transect. The IsoGSM2 simulations in this region have been robustly demonstrated to correlate strongly with in situ measurements, across both the East Asian[12] and South Asian monsoon domains[13]. During the pre-monsoon

season, lower $\delta^{18}O_p$ and OLR expand progressively westward from Luoyuan (the easternmost site) to Kunming–Baoshan–Tengchong (the middle sites) along the east–west transect. From February to May, $\delta^{18}O_p$ and OLR show a spatial pattern of low in the east and high in the west of the ~98° E dividing line, yielding negative slopes from the middle to the easternmost sites. Hence, we used the monthly $\delta^{18}O_p$ and OLR values at Luoyuan as the base values. We then subtracted the corresponding base values from the monthly $\delta^{18}O_p$ and OLR at Luoyuan, Sanming, Guilin, Hechi, Kunming, Baoshan, and Tengchong for each month during the pre-monsoon season. Thus, the $\Delta\delta^{18}O_p$ and ΔOLR at Luoyuan are 0‰ and 0 W/m^2, respectively. Finally, we calculated the slopes of $\Delta\delta^{18}O_p$ and ΔOLR using the values from the seven sites (Tengchong, Baoshan, Kunming, Hechi, Guilin, Sanming, and Luoyuan) for each month, thereby deriving the spatial gradients (Fig. 3c, d).

During the monsoon season, the lower values of $\delta^{18}O_p$ and OLR appear around the middle of the transect, resulting in a V-shaped pattern. Consequently, $\delta^{18}O_p$ and OLR values increase from the middle to the easternmost site (Luoyuan). This implies that the gradients (slopes) from the middle to Luoyuan are positive. If the slopes are negative, this indicates that the lower values do not extend westward to that site. Hence, we define the site immediately east of such a site as the critical site. For standardization, the monthly $\delta^{18}O_p$ and OLR values at the critical site were used as the base values. As the critical site is different from June to September, the base value also differs by month. We then normalized the $\delta^{18}O_p$ and OLR values at the critical site and other eastern sites by subtracting the corresponding base values. Finally, we

calculated the slopes of the $\Delta\delta^{18}O_p$ and ΔOLR from the critical site to Luoyuan for each monsoon month.

Taking June as an example, both $\delta^{18}O_p$ and OLR slopes are positive when calculated from six sites (Baoshan, Kunming, Hechi, Guilin, Sanming, and Luoyuan). Including Tengchong yields negative slopes, indicating that Tengchong is the specific site in June. Therefore, Boshan, the site immediately east of Tengchong, is defined as the critical site. Thus, in June, we used Baoshan as the base site, setting $\Delta\delta^{18}O_p$ and ΔOLR at 0‰ and 0 W/m². We then standardized the values at all sites between Baoshan and Luoyuan by subtracting the base values, and calculated the slopes from these six sites.

In July–September, the specific sites were Ruili (July), Sylhet (August), and Dhanbad (September). Accordingly, their immediately adjacent eastern sites (Tengchong, Ruili, and Sylhet, respectively) served as the critical sites. We then calculated slopes from the seven sites (Tengchong, Baoshan, Kunming, Hechi, Guilin, Sanming, and Luoyuan) in July, eight sites in August (Ruili to Luoyuan), and nine sites in September (Sylhet to Luoyuan) (Fig. 3e, f).

Text S6. Assessing the influence of precipitation amount on $\delta^{18}O_p$

Others might contend that $\delta^{18}O_p$ variations in this region are significantly influenced by the amount effect (i.e., higher precipitation amount leads to lower $\delta^{18}O_p$). To evaluate this contention, we first examined the spatial distribution of precipitation amount at the 12 primary sites along the transect for both the pre-monsoon and

monsoon seasons (Fig. 4e-h, 4m-p), following the same approach used for OLR. If the classical amount effect were the dominated control, we would expect a progressive east-to-west decrease in precipitation amount during the pre-monsoon season, and an inversed V-shaped (Λ-shaped) precipitation amount pattern during the monsoon season, corresponding to the minimum $\delta^{18}O_p$ values in the middle of the transect. However, our observations do not support these expectations. During the pre-monsoon season, only the eastern domain shows an east-to-west decrease in precipitation amount, whereas the western domain exhibits anomalously high precipitation at Sylhet together with relatively high $\delta^{18}O_p$ values. During the monsoon season, precipitation amount follows a V-shaped rather than a Λ-shaped pattern: precipitation amount is relatively low in the middle of the transect, where $\delta^{18}O_p$ reaches its minimum. These spatial patterns are therefore inconsistent with a simple amount effect interpretation. These results indicate that precipitation amount is not the key factor governing the patterns of $\delta^{18}O_p$ along the transect during the pre-monsoon and monsoon seasons.

We then quantified the correlations between $\delta^{18}O_p$ and precipitation amount at all 46 sites. On both monthly and multi-year monthly mean scales, although a negative correlation is observed (Supplementary Fig. 8a, b), the correlations are weaker than those between $\delta^{18}O_p$ and OLR (Fig. 3a, b). More importantly, the scatter points of $\delta^{18}O_p$ versus precipitation amount are not evenly distributed around the regression lines (Supplementary Fig. 8a, b). These results indicate that the influence of precipitation amount on $\delta^{18}O_p$ is weaker than that of atmospheric updraft.

To quantitatively assess the relative importance of atmospheric updraft (as represented by OLR) and precipitation amount (P) in driving variability in $\delta^{18}O_p$ across the region, we further constructed and compared two linear regression models: Model 1 (OLR only) and Model 2 (OLR and P) (Supplementary Table 6). The result showed that Model 2 yielded a lower AIC (12,363.42) than Model 1 (AIC = 12,390.25); however, the corresponding increase in R^2 was only 0.0098. Five-fold cross-validation showed that Model 1 achieved a slightly lower root-mean-square error (RMSE = 3.1227) than Model 2 (RMSE = 3.1310). Given the comparable predictive performance of both models and the principle of parsimony, we selected the simpler model (Model 1) as the more robust representation. Furthermore, analysis of the standardized regression coefficients (Beta) for Model 2 revealed that the absolute coefficient for OLR (0.3514) was approximately three times that of P (−0.1158). Collectively, these results reinforce that precipitation amount plays a secondary role in modulating $\delta^{18}O_p$ in this region, compared with atmospheric updraft (OLR).

We next examined the correlation between precipitation amount and OLR/vertical velocity (Supplementary Fig. 8c, d). Significant correlations were found at both monthly and multi-year monthly mean scales, indicating that precipitation amount is closely linked to atmospheric updraft—a finding consistent with fundamental meteorology, as vertical motion is one of the primary controls on precipitation formation. This close coupling implies that precipitation amount does not act as an independent driver of $\delta^{18}O_p$; rather, the influence of precipitation amount on $\delta^{18}O_p$ is likely mediated by, or co-varies with, the atmospheric updraft that governs

both precipitation amount and $\delta^{18}O_p$. Consequently, the major role of atmospheric updraft (OLR) in explaining $\delta^{18}O_p$ variability (as shown in our models) is physically plausible, whereas the secondary role of precipitation likely reflects its shared dependence on atmospheric updraft strength rather than an independent mechanistic effect.

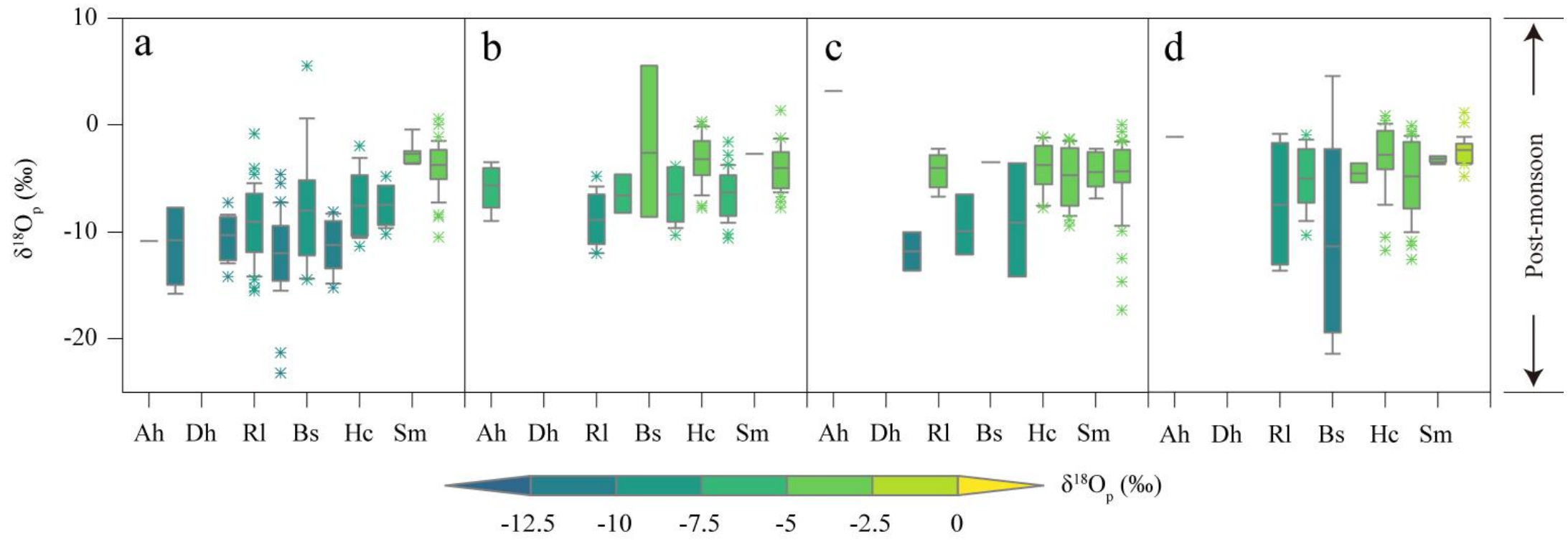

Fig. S1 | Spatial distributions of observed $\delta^{18}O_p$ at the 12 primary sites along the east–west transect during the post-monsoon season. The post-monsoon season includes October (**a**), November (**b**), December (**c**), and January (**d**). Ah, Dh, Rl, Bs, Hc, and Sm correspond to Ahmedabad, Dhanbad, Ruili, Baoshan, Hechi, and Sanming, respectively. In each box plot, the box represents the 20th–80th percentile range, whiskers extend to the 10th–90th percentile, horizontal lines inside the boxes indicate the mean, and dots represent outliers. $\delta^{18}O_p$ values are expressed as per mil (‰) deviations relative to VSMOW or VSMOW2.

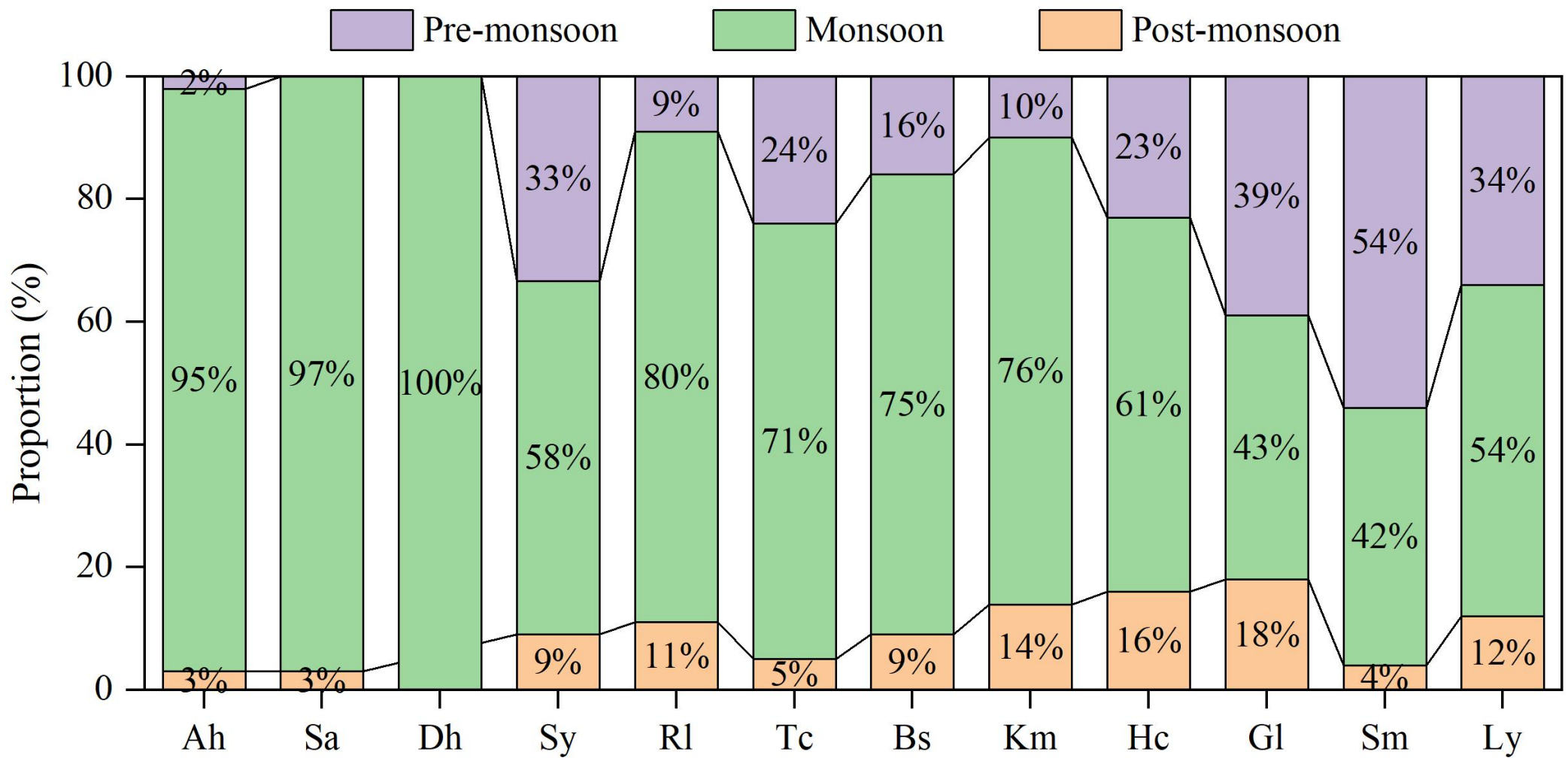

Fig. S2 | Seasonal contributions to annual precipitation at the 12 primary sites along the east–west transect.

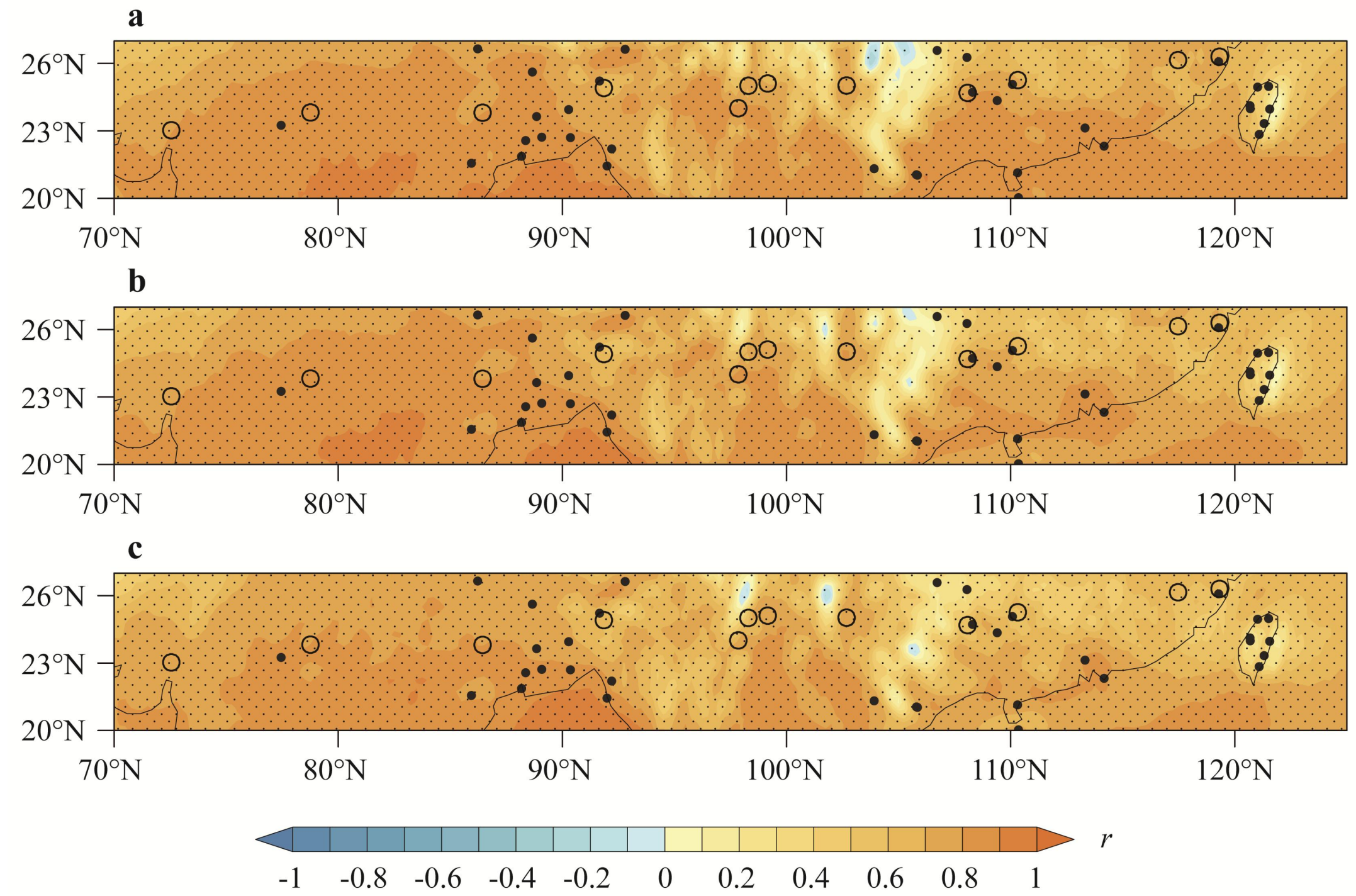

Fig. S3 | Spatial correlations between monthly vertical velocity (*ω*) and the corresponding outgoing longwave radiation (OLR) from 1979 to 2022. a Vertical velocity at 300 hPa. **b** Vertical velocity at 400 hPa. **c** Vertical velocity at 500 hPa. Negative and positive *ω* values indicate upward and downward motions, respectively. Open black circles indicate the 12 primary sites selected for this study (Supplementary Table 1), while black dots represent the 34 ancillary sites (Supplementary Table 2). The black dots denote correlations that are significant at the 95% confidence level.

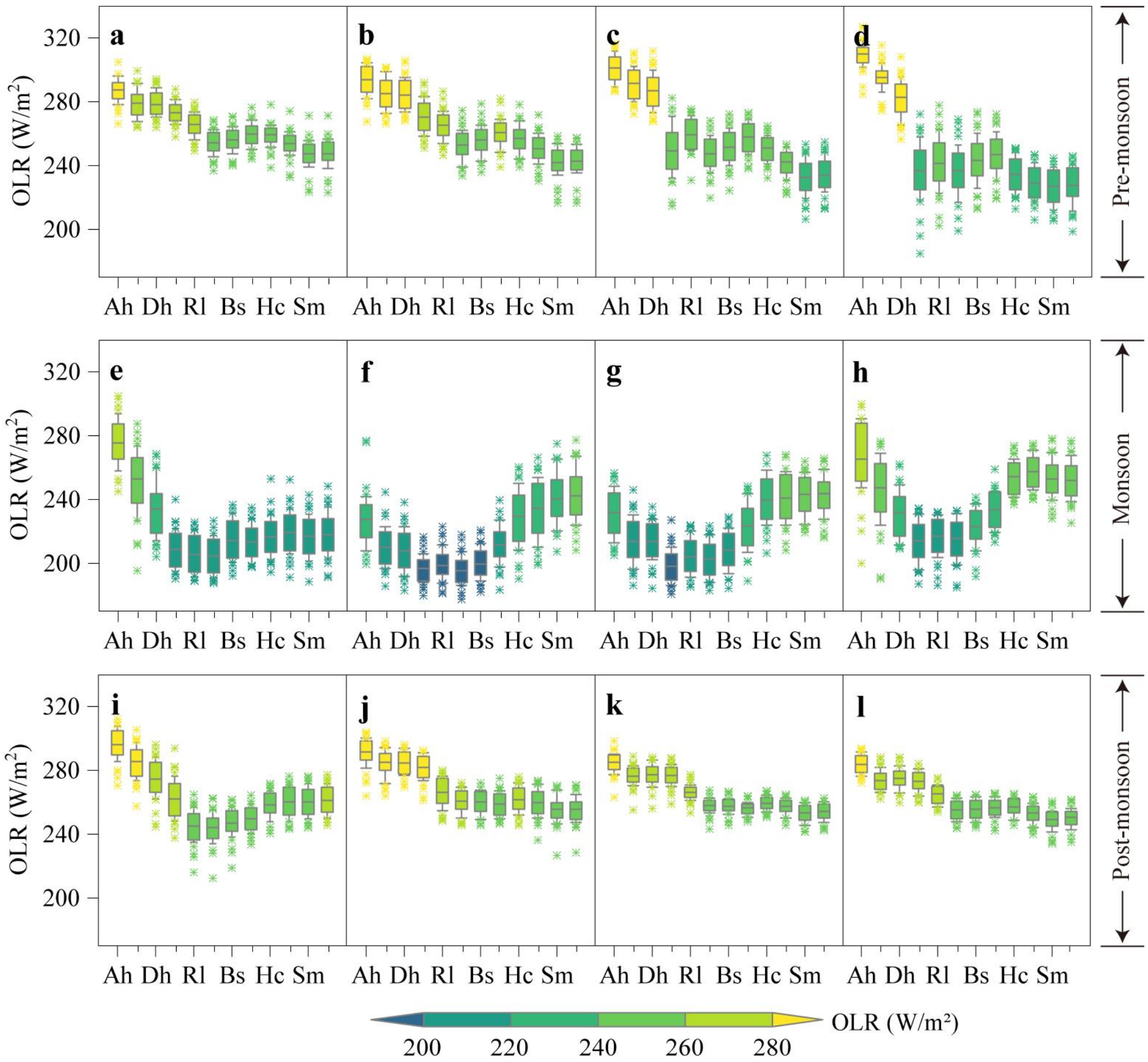

Fig. S4 | Spatial distributions of outgoing longwave radiation (OLR) at the 12 primary sites along the east–west transect from 1979 to 2022. a–d Pre-monsoon season. **e–h** Monsoon season. **i–l** Post-monsoon season. Abbreviations: Ah (Ahmedabad), Dh (Dhanbad), Rl (Ruili), Bs (Baoshan), Hc (Hechi), and Sm (Sanming). In each box plot, the box represents the 20th–80th percentile range, whiskers extend to the 10th–90th percentile, the horizontal lines inside the boxes indicate the mean, and dots represent outliers.

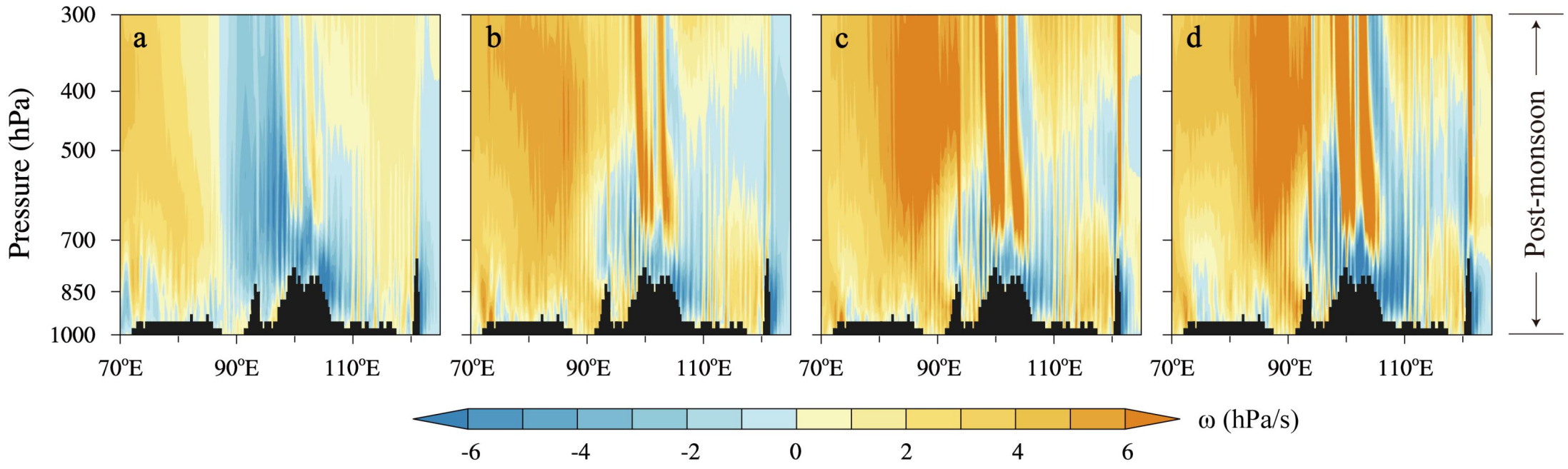

Fig. S5 | Vertical profiles of meridional average vertical velocity (*ω*) from 1979 to 2022 during the post-monsoon season. The meridional average is calculated over 20–27° N. Negative and positive *ω* values indicate upward and downward motions, respectively. The post-monsoon season includes October (**a**), November (**b**), December (**c**), and January (**d**).

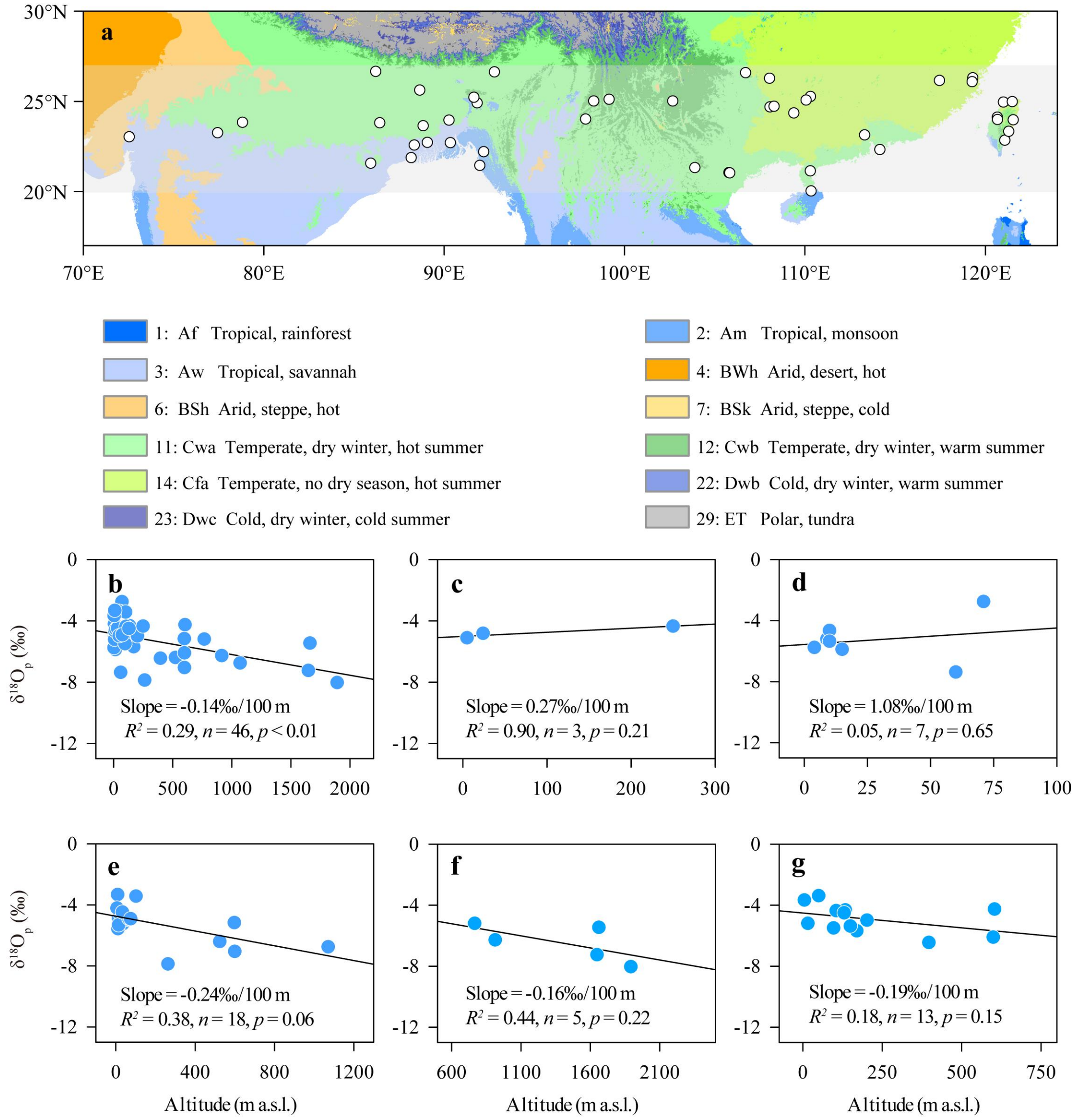

Fig. S6 | Correlations between observed $\delta^{18}O_p$ values and altitudes across five climate zones. **a** Climate zones according to the Köppen-Geiger climate classification[14]. **b** Correlations for the 46 sites across the entire study region. **c–g** Correlations for the climate zones: Tropical, monsoon (Am) (**c**); Tropical, savannah (Aw) (**d**); Temperate, dry winter, hot summer (Cwa) (**e**); Temperate, dry winter, warm summer (Cwb) (**f**); and Temperate, no dry season, hot summer (Cfa) (**g**). $\delta^{18}O_p$ values are expressed as per mil (‰) deviations relative to VSMOW or VSMOW2.

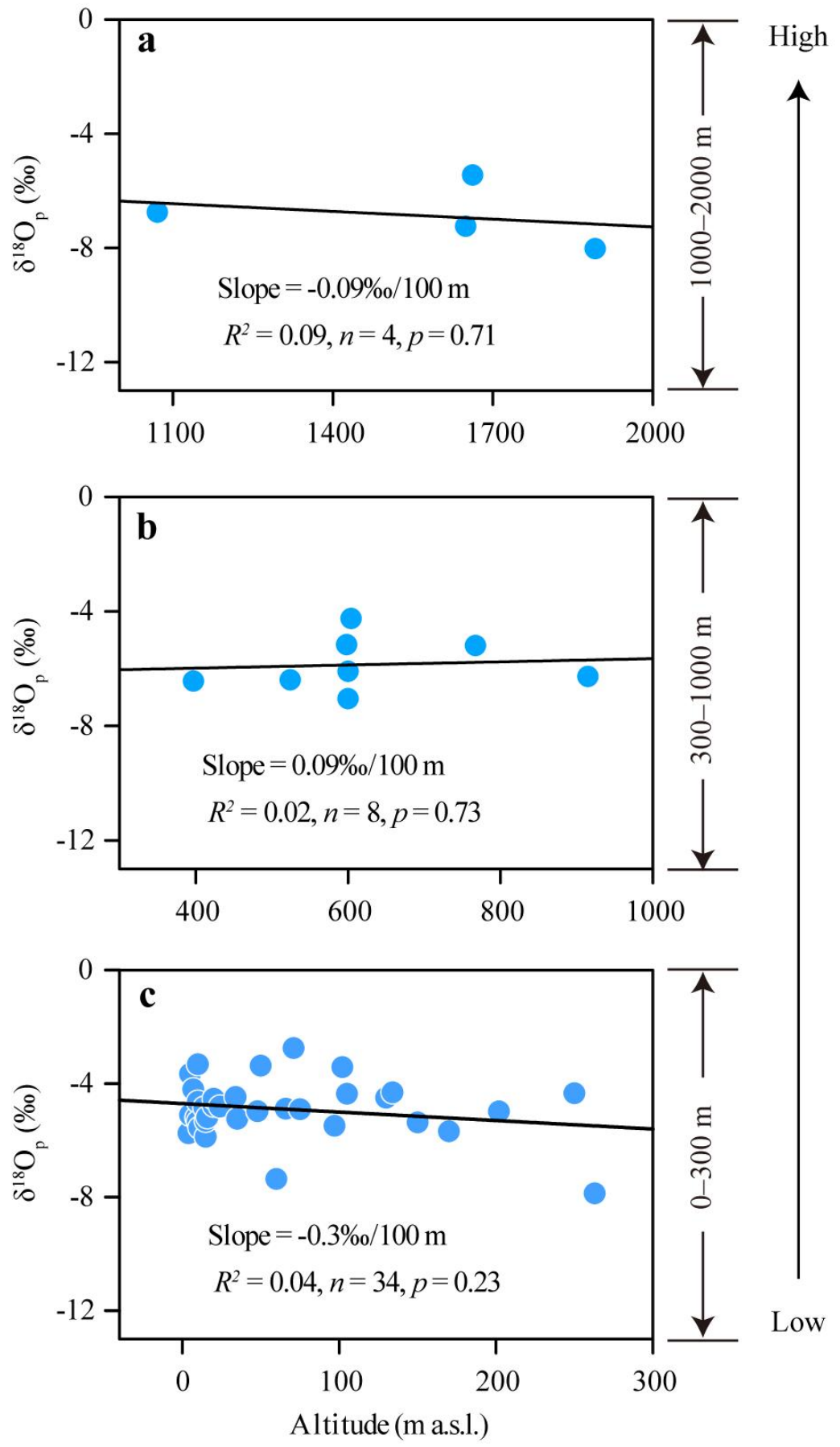

Fig. S7 | Correlations between observed δ^{18}O$_p$ values and altitudes. The 46 precipitation sites were categorized into three altitude ranges based on the UNEP-WCMC mountain classification[15]. **a**–**c** Correlations for the altitude ranges 1000–2000 m (**a**), 300–1000 m (**b**), and 0–300 m (**c**). δ^{18}O$_p$ values are expressed as per mil (‰) deviations relative to VSMOW or VSMOW2.

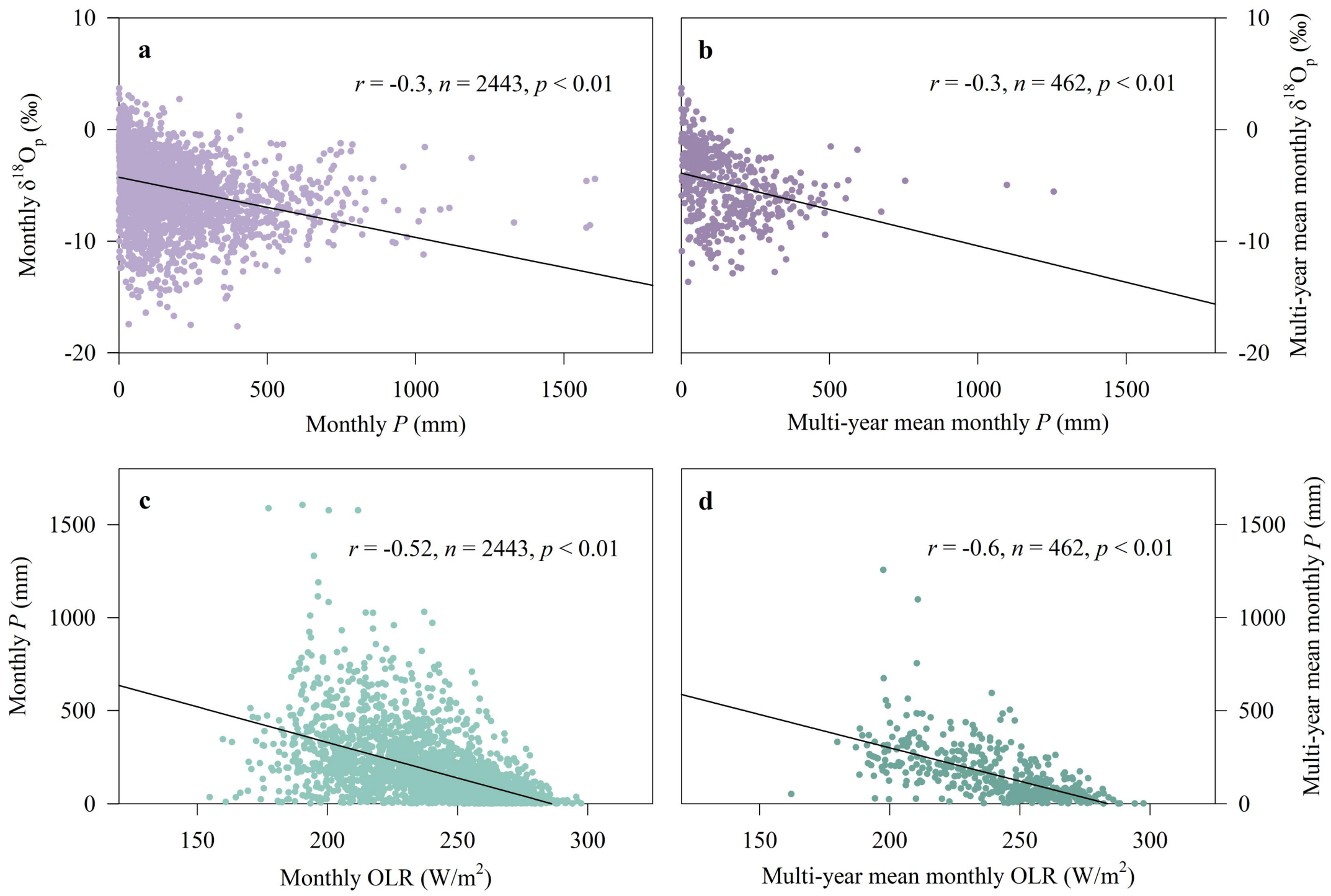

Fig. S8 | Correlations between precipitation amount (P) and both $\delta^{18}O_p$ and outgoing longwave radiation (OLR) along the transect. a–b Linear regressions of P against the corresponding $\delta^{18}O_p$ at the 46 sites across the study region on different timescales. **c–d** Linear regressions of P at the 46 sites across the study region against the corresponding OLR on different timescales.

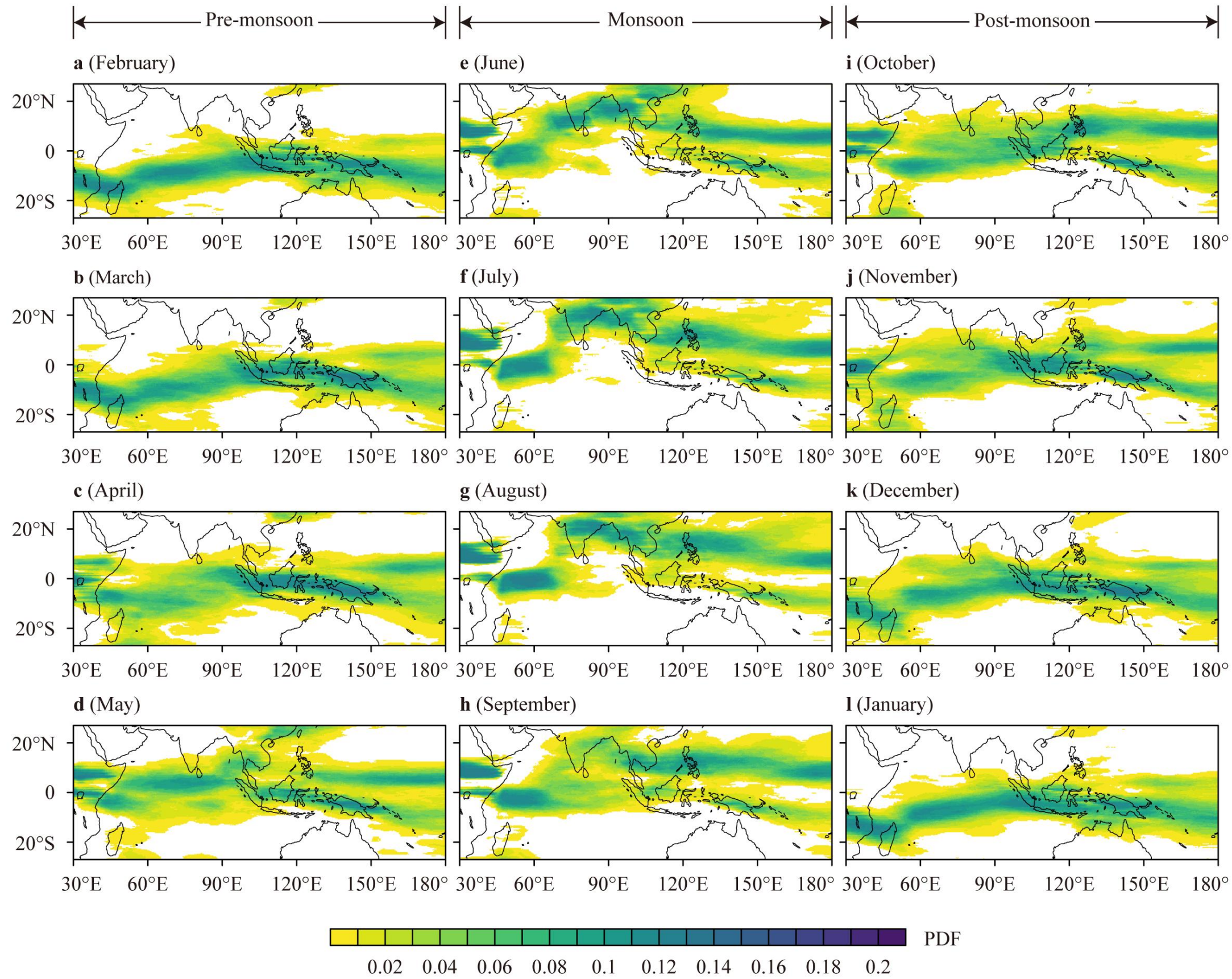

Fig. S9 | Location of the Intertropical Convergence Zone (ITCZ) over 1979–2022. **a–d** Pre-monsoon season. **e–h** Monsoon season. **i–l** Post-monsoon season. Abbreviations: PDF (the empirical probability density function).

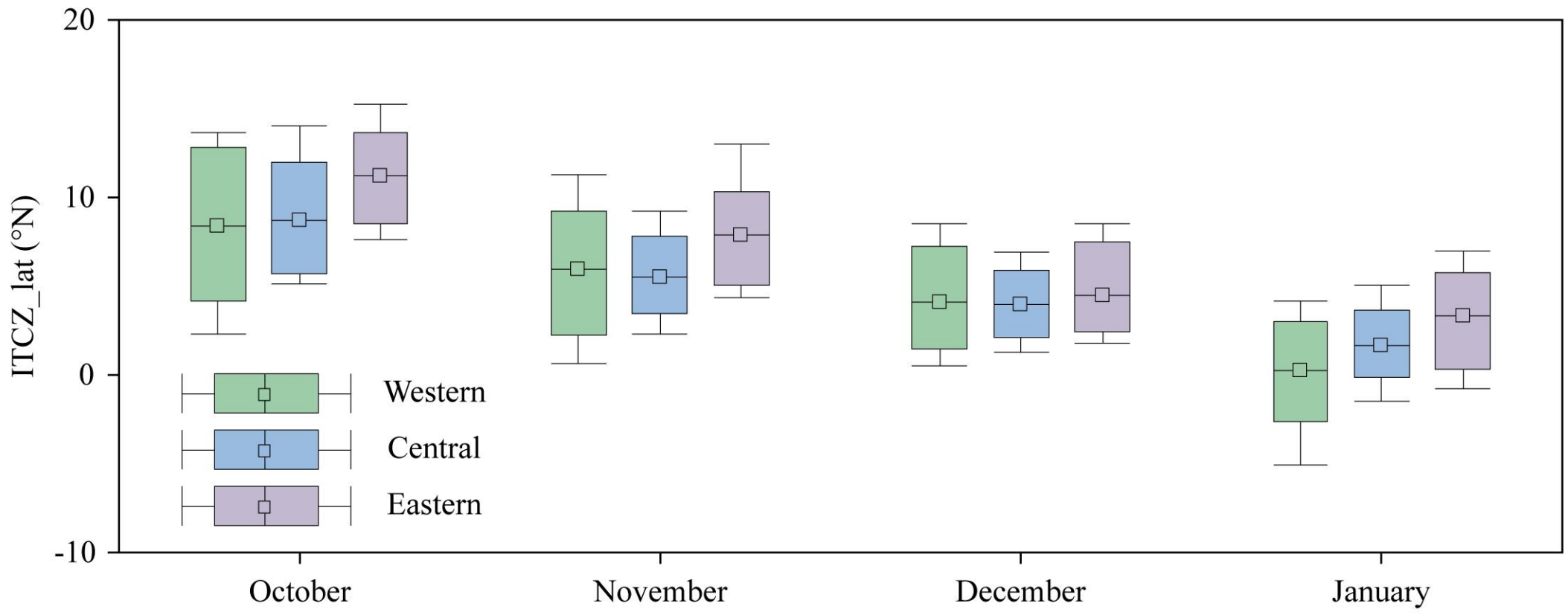

Fig. S10 | Intertropical Convergence Zone (ITCZ) northern boundary location within the respective longitude ranges of three subregions over 1979–2022 during the post-monsoon season. Western, Central, and Eastern represent the three subregions: Western (70–95° E), Central (95–105° E), and Eastern (105–125° E). In each box plot, the box represents the 15th–85th percentile range, and whiskers extend to the 5th–95th percentiles. Inside each box, the mean is shown as both a hollow square and a horizontal line.

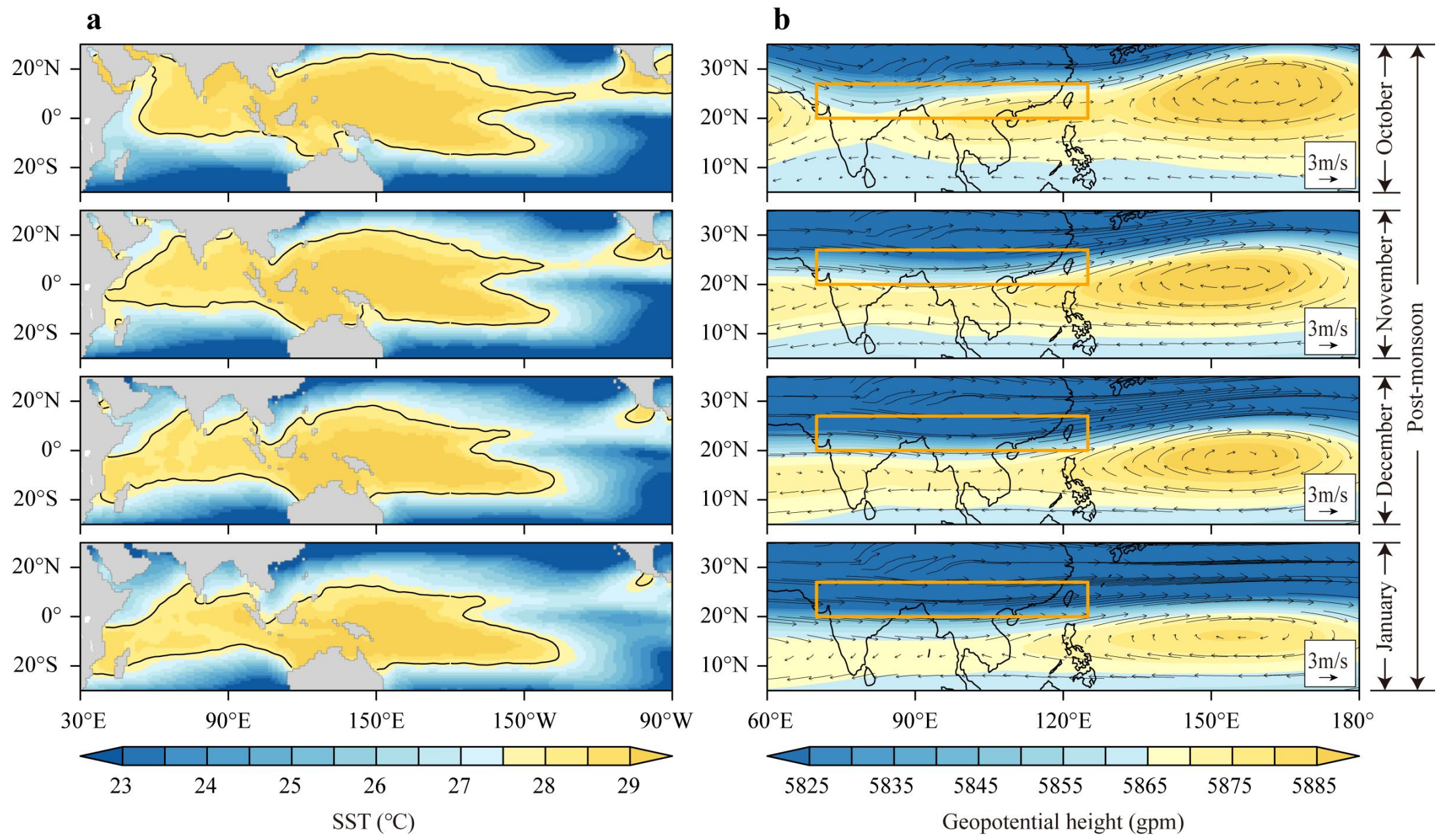

Fig. S11 | Spatial distributions of monthly sea surface temperatures (SSTs), 500 hPa geopotential height, and wind field over 1979–2022 during the post-monsoon season. a Spatial distributions of SSTs. The black line indicates the 28 °C SST, marking the extent of the Indo-Pacific Warm Pool. **b** Spatial distributions of 500 hPa geopotential height and wind field. Shading represents geopotential height, arrows indicate the wind field, and the orange rectangle marks the study region.

Table S1 | Summary data for the 12 primary precipitation sampling sites in the region of 20–27° N, 70–125° E. The sampling sites are listed by longitude from east to west.

No.	Site	Latitude (° N)	Longitude (° E)	Elevation (m a.s.l.)	Period	Number of samples	Time scale	Reference
1	Luoyuan	26.3	119.32	604	2018/1–2021/12	529	Daily	Ref. [16]
2	Sanming	26.15	117.47	134	2019/1–2020/12	209	Daily	Ref. [17]
3	Guilin	25.26	110.32	150	2010/1–2010/12; 2012/1–2012/12; 2015/1–2016/12	450	Daily	Ref. [18-20]
4	Hechi	24.69	108.08	202	2015/1–2017/12	303	Daily	This study
5	Kunming	25.02	102.68	1892	1986/4–2011/12	284	Daily/Monthly	Ref. [21]; GNIP
6	Baoshan	25.11	99.16	1662	2017/8–2019/8	215	Daily	This study
7	Tengchong	25.01	98.3	1649	2009/1–2011/12	343	Daily	Ref. [22]
8	Ruili	24.01	97.85	767	2018/1–2021/12	450	Daily	Ref. [16]
9	Sylhet	24.91	91.85	20	2009/5–2021/9	363	Daily/Monthly	Ref. [23]; GNIP
10	Dhanbad	23.81	86.44	263	2013/6–2013/9	61	Daily	Ref. [24]
11	Sagar	23.82	78.76	598	2016/6–2021/10	291	Daily	Ref. [25]
12	Ahmedabad	23.03	72.55	71	2005/7–2016/9	329	Daily	Ref. [26]

Table S2 | Summary data for the 34 ancillary precipitation sampling sites in the region of 20–27° N, 70–125° E. The sampling sites are listed by longitude from east to west.

No.	Site	Latitude (° N)	Longitude (° E)	Elevation (m a.s.l.)	Period	Number of samples	Time scale	Referenc
1	Hualien	23.97	121.56	50	2003/7–2006/5	35	Monthly	Ref. [27]
2	Taipei	24.98	121.51	5	2004/3–2006/12	34	Monthly	Ref. [27]
3	Yuli	23.33	121.31	130	2005/1–2007/8	31	Monthly	Ref. [27]
4	Taidung	22.84	121.09	250	2004/5–2007/11	38	Monthly	Ref. [27]
5	Taoyuan	24.95	121.02	105	2004/1–2007/4	35	Monthly	Ref. [27]
6	Tsaotun	23.99	120.69	75	2001/7–2006/11	54	Monthly	Ref. [27]
7	Taichung	24.12	120.68	34	2001/6–2007/10	67	Monthly	Ref. [27]
8	Fuzhou	26.08	119.28	16	1985/10–1992/12	71	Monthly	GNIP
9	Hong Kong	22.32	114.17	66	1979/1–2022/12	496	Monthly	GNIP
10	Guangzhou	23.13	113.32	7	1986/4–2014/12	1046	Daily/Monthly	Ref. [28]; GNIP
11	Haikou	20.03	110.35	15	1988/3–2000/7	59	Monthly	GNIP
12	Zhanjiang	21.14	110.31	10	2015/5/7–2020/4	412	Daily	Ref. [29]
13	Guilin (GNIP)	25.07	110.08	170	1983/1–1990/12	92	Monthly	GNIP

14	Liuzhou	24.35	109.4	97	1988/1–1992/8	53	Monthly	GNIP
15	Huanjiang	24.72	108.3	397	2011/1–2020/12	94	Monthly	Ref. [30]
16	Liangfeng	26.27	108.05	600	2011/5–2014/4	36	Monthly	Ref. [31]
17	Guiyang	26.58	106.72	1071	1988/2–1992/12	55	Monthly	GNIP
18	Hanoi (Igs)	21.03	105.84	15	2015/1–2021/11	80	Monthly	GNIP
19	Hanoi	21.05	105.8	11	2004/9–2007/12	38	Monthly	GNIP
20	Son La	21.32	103.91	600	2017/4–2021/11	46	Monthly	GNIP
21	Tezpur	26.63	92.8	48	2015/4–2018/9	235	Daily	Ref. [32]
22	Bandarban	22.2	92.2	24	2015/3–2021/11	48	Monthly	GNIP
23	Cox's Bazar	21.44	91.99	5	2014/5–2021/10	154	Daily/Monthly	Ref. [33]; GNIP
24	Mawlong	25.22	91.66	915	2007/4–2008/10	68	Daily	Ref. [34]; GNIP
25	Barisal	22.7	90.36	10	2013/5–2021/10	436	Daily/Monthly	Ref. [33]; GNIP
26	Dhaka	23.95	90.28	14	2009/3–2021/12	119	Monthly	GNIP
27	Satkhira	22.72	89.08	10	2015/4–2021/9	162	Daily/Monthly	Ref. [24]; GNIP
28	Chuadanga	23.64	88.85	20	2014/5–2021/11	62	Monthly	GNIP
29	Dinajpur	25.62	88.66	35	2014/2–2021/10	62	Monthly	GNIP
30	Kolkata	22.57	88.36	9	2004/6–2018/10	206	Daily/Weekly	Ref. [32,35]

31	Kakdweep	21.87	88.18	4	2015/4–2016/11	53	Daily	Ref. [32]
32	Siraha	26.65	86.22	102	2014/5–2017/10	222	Daily	Ref. [36]
33	RGS-1	21.56	85.94	60	2016/7–2016/9	52	Daily	Ref. [37]
34	Bhopal	23.25	77.45	524	2013/6–2013/9	58	Daily	Ref. [24]

Table S3 | Correlations of observed monthly δ$^{18}O_p$, East Asia Summer Monsoon Index (EASMI), Western North Pacific Monsoon Index (WNPMI), South Asia Summer Monsoon Index (SASMI), Indian Monsoon Index (IMI), and local outgoing longwave radiation (OLR) at five sites of Hong Kong, Guangzhou, Kunming, Sylhet, and Dhaka.

Site	Period	$R_{\delta\text{-EASMI}}$	$R_{\delta\text{-WNPMI}}$	$R_{\delta\text{-SASMI}}$	$R_{\delta\text{-IMI}}$	$R_{\delta\text{-OLR}}$
Hong Kong	1973–2022†	0.04 (*n* = 146, *p* = 0.627)	-0.17* (*n* = 146, *p* = 0.038)	-0.02 (*n* = 196, *p* = 0.800)	-0.04 (*n* = 196, *p* = 0.550)	0.55** (*n* = 552, *p* = 0.000)
Guangzhou	1986–2014	0.36* (*n* = 33, *p* = 0.038)	0.20 (*n* = 33, *p* = 0.264)	-0.23 (*n* = 45, *p* = 0.125)	-0.28 (*n* = 45, *p* = 0.061)	0.50** (*n* = 124, *p* = 0.000)
Kunming	1986–2003	0.17 (*n* = 44, *p* = 0.284)	-0.35* (*n* = 44, *p* = 0.019)	-0.23 (*n* = 59, *p* = 0.087)	-0.33* (*n* = 59, *p* = 0.012)	0.61** (*n* = 152, *p* = 0.000)
Sylhet	2009–2021	0.47** (*n* = 37, *p* = 0.004)	-0.01 (*n* = 37, *p* = 0.957)	-0.17 (*n* = 48, *p* = 0.255)	-0.20 (*n* = 48, *p* = 0.175)	0.38** (*n* = 104, *p* = 0.000)
Dhaka	2009–2021	0.23 (*n* = 39, *p* = 0.161)	-0.42** (*n* = 39, *p* = 0.008)	-0.38** (*n* = 52, *p* = 0.005)	-0.23 (*n* = 52, *p* = 0.095)	0.39** (*n* = 119, *p* = 0.000)

* The *p* values are significant at the 0.05 level.

** The *p* values are significant at the 0.01 level.

† Note that the correlations at Hong Kong were calculated using observed monthly δ$^{18}O_p$ over 1973-2022.

Table S4 | Correlations of observed annual $\delta^{18}O_p$, EASMI, WNPMI, SASMI, IMI, and outgoing longwave radiation (OLR) at five sites of Hong Kong, Guangzhou, Kunming, Sylhet, and Dhaka.

Site	Period	$R_{\delta\text{-EASMI}}$	$R_{\delta\text{-WNPMI}}$	$R_{\delta\text{-SASMI}}$	$R_{\delta\text{-IMI}}$	$R_{\delta\text{-OLR}}$
Hong Kong	1973–2022†	0.07 (n = 50, p = 0.64)	0.12 (n = 50, p = 0.40)	-0.19 (n = 50, p = 0.18)	-0.19 (n = 50, p = 0.19)	0.31* (n = 50, p = 0.03)
Guangzhou	1986–2014	0.49 (n = 12, p = 0.11)	0.57 (n = 12, p = 0.05)	-0.34 (n = 12, p = 0.28)	-0.46 (n = 12, p = 0.13)	0.68* (n = 12, p = 0.02)
Kunming	1986–2003	0.17 (n = 15, p = 0.54)	-0.05 (n = 15, p = 0.87)	-0.08 (n = 15, p = 0.77)	-0.29 (n = 15, p = 0.30)	0.60* (n = 15, p = 0.02)
Sylhet	2009–2021	0.62* (n = 13, p = 0.02)	0.59* (n = 13, p = 0.03)	-0.26 (n = 13, p = 0.38)	-0.52 (n = 13, p = 0.07)	0.15 (n = 13, p = 0.64)
Dhaka	2009–2021	0.57* (n = 13, p = 0.04)	0.38 (n = 13, p = 0.20)	-0.51 (n = 13, p = 0.08)	-0.44 (n = 13, p = 0.13)	0.59* (n = 13, p = 0.03)

* The *p* values are significant at the 0.05 level.

** The *p* values are significant at the 0.01 level.

† Note that the correlations at Hong Kong were calculated using observed monthly $\delta^{18}O_p$ over 1973-2022.

Table S5 | Monthly slopes of $\delta^{18}O_p$ and OLR with associated regression statistics (slope, R^2, RMSE, and p).

Month	$\delta^{18}O_p$_slope	$\delta^{18}O_p$ _R^2	$\delta^{18}O_p$_RMSE	$\delta^{18}O_p$_p	OLR_slope	OLR_R^2	OLR_RMSE	OLR_p
February	-0.1361	0.3105	0.4055	0.194	-1.5857	0.4604	3.4335	0.094
March	-0.2679	0.776	0.2878	0.009	-2.4982	0.5569	4.4567	0.054
April	-0.0668	0.2593	0.2258	0.243	-3.315	0.5826	5.6118	0.046
May	-0.0018	0.00007	0.4380	0.986	-2.7989	0.593	4.6372	0.043
June	0.0886	0.2617	0.2540	0.3	0.9220	0.5573	1.4035	0.088
July	0.2693	0.3039	0.8150	0.2	8.8086	0.9431	4.3262	< 0.001
August	0.2494	0.1731	1.2490	0.305	7.0470	0.8911	5.6448	< 0.001
September	0.2230	0.0908	1.8219	0.431	6.1948	0.8402	6.9752	0.001

Note: R^2, variance explained by the linear trend; RMSE, root-mean-square error; p, significance of the slope.

Table S6 | Summary of model comparison results

Metric	Model 1 (OLR only)	Model 2 (OLR and *P*)
AIC	12390.25	12363.42
R^2	0.1697	0.1795
RMSE (Five-fold)	3.1227	3.1310
Standardized regression coefficient (Beta)	—	Beta_OLR = 0.3514; Beta_*P* = –0.1158

Note: All models are based on the same set of observations (n = 2443).

References

1. Chakraborty, S. et al. Linkage between precipitation isotopes and biosphere-atmosphere interaction observed in northeast India. *npj Clim. Atmos. Sci.* **5**, 10 (2022).
2. Midhun, M. et al. The effect of monsoon circulation on the stable isotopic composition of rainfall. *J. Geophys. Res. Atmos.* **123**, 5205–5221 (2018).
3. Zhang, J. et al. Coupled effects of moisture transport pathway and convection on stable isotopes in precipitation across the East Asian monsoon region: Implications for paleoclimate reconstruction. *J. Climate* **34**, 9811–9822 (2021).
4. Baker, A. J. et al. Seasonality of westerly moisture transport in the East Asian summer monsoon and its implications for interpreting precipitation $\delta^{18}O$. *J. Geophys. Res. Atmos.* **120**, 5850–5862 (2015).
5. Pausata, F. S. R., Battisti, D. S., Nisancioglu, K. H., & Bitz, C. M. Chinese stalagmite $\delta^{18}O$ controlled by changes in the Indian monsoon during a simulated Heinrich event. *Nat. Geosci.* **4**, 474–480 (2011).
6. He, C. et al. Hydroclimate footprint of pan-Asian monsoon water isotope during the last deglaciation. *Sci. Adv.* **7**, eabe2611 (2021).
7. Wang, B. & Fan, Z. Choice of South Asian summer monsoon indices. *Bull. Amer. Meteor. Soc.* **80**, 629–638 (1999).
8. Wang, B., Wu, R. & Lau, W. K. M. Interannual variability of Asian summer monsoon: Contrast between the Indian and western North Pacific-East Asian

monsoons. *J. Climate* **14**, 4073–4090 (2001).

9. Liebmann, B. & Smith, C. A. Description of a complete (interpolated) outgoing longwave radiation dataset. *Bull. Amer. Meteorol. Soc.* **77**, 1275–1277 (1996).

10. Webster, P. J. et al. Monsoons: Processes, predictability, and the prospects for prediction. *J. Geophys. Res.* **103**, 14451–14510 (1998).

11. Yoshimura, K., Kanamitsu, M., Noone, D. & Oki T. Historical isotope simulation using Reanalysis atmospheric data. *J. Geophys. Res. Atmos.* **113**, D19108 (2008).

12. Chiang, J. C. H., Herman, M. J., Yoshimura, K., & Fung, I. Y. Enriched East Asian oxygen isotope of precipitation indicates reduced summer seasonality in regional climate and westerlies. *Proc. Natl. Acad. Sci. U.S.A.* **117**, 14745–1475 (2020).

13. Zhang, J. et al. Controls on stable water isotopes in monsoonal precipitation across the Bay of Bengal: Atmosphere and surface analysis. *Geophys. Res. Lett.* **50**, e2022GL102229 (2023).

14. Beck, H. E. et al. High-resolution (1 km) Köppen-Geiger maps for 1901–2099 based on constrained CMIP6 projections. *Sci. Data* **10**, 724 (2023).

15. UNEP World Conservation Monitoring Centre. *Mountain Watch: Environmental Change and Sustainable Development in Mountains*. Technical Report. Cambridge, UK: UNEP-WCMC (2002).

16. Guo, H. et al. Global abnormal precipitation ^{18}O depletion during late/post monsoon season. *Earth Planet. Sci. Lett.* **641**, 118815 (2024).

17. Cai, J., Yang, Y., Yang, Z., Qiu, W. & Jiang, X. Isotope composition of daily

precipitation from 2019 to 2020 in Sanming, southeastern China. *Front. Environ. Sci*. **10**, 1061882 (2022).

18. Wu, X., Zhu, X., Zhang, M., Bai, X. & Zhang, B. high-resolution stable isotope record of atmospheric precipitation in Guilin. *Resour. Environ. Yangtze Basin.* **22**, 7 (2013).
19. Wu, X., Zhu, X., Pan, M. & Zhang, M. Seasonal variability of oxygen and hydrogen stable isotopes in precipitation and cave drip water at Guilin, southwest China. *Environ. Earth Sci*. **72**, 3183–3191 (2014).
20. Wu, X. et al. Effect of the El Niño–Southern Oscillation on hydrogen and oxygen isotope ratios of precipitation in Guilin, SW China. *Isot. Environ. Healt. S.* **57**, 67–81 (2020).
21. Li, G., Zhang, X., Xu, Y., Wang, Y. & Wu, H. Synoptic time-series surveys of precipitation $\delta^{18}O$ and its relationship with moisture sources in Yunnan, southwest China. *Quatern. Int.* **440**, 40–51 (2017).
22. Wu, H. et al. Temporal variations of stable isotopes in precipitation from Yungui Plateau: Insights from moisture source and rainout effect. *J. Hydrometeorol.* **23**, 39–51 (2022).
23. Islam, M. R. et al. Controls on spatiotemporal variations of stable isotopes in precipitation across Bangladesh. *Atmos. Res.* **247**, 105224 (2021).
24. Midhun, M. et al. The effect of monsoon circulation on the stable isotopic composition of rainfall. *J. Geophys. Res. Atmos*. **123**, 5205–5221 (2018).
25. Chakraborty, S. et al. Precipitation isotopes and monsoon dynamics in the core

monsoon zone of India. *Sci. Rep.* **15**, 6761 (2025).

26. Oza, H. et al. Hydrometeorological processes in semi-arid western India: insights from long term isotope record of daily precipitation. *Clim. Dyn.* **54**, 2745–2757 (2020).
27. Peng, T. et al. Stable isotopic characteristic of Taiwan's precipitation: A case study of western Pacific monsoon region. *Earth Planet. Sci. Lett.* **289**, 357–366 (2010).
28. Yang, X., Davis, M., Acharya, S. & Yao, T. Asian monsoon variations revealed from stable isotopes in precipitation. *Clim. Dyn.* **51**, 2267–2283 (2017).
29. Chen, F. et al. Typhoon control of precipitation dual isotopes in southern China and its palaeoenvironmental implications. *J. Geophys. Res. Atmos.* **126**, e2020JD034336 (2021).
30. Zhang, J. et al. Temporal variations of precipitation driven by local meteorological parameters in southwest China: Insights from 9 years of continuous hydro-meteorological and isotope observations. *J. Hydrol. Reg. Stud.* **46**, 101345 (2023).
31. Duan, W. et al. The transfer of seasonal isotopic variability between precipitation and drip water at eight caves in the monsoon regions of China. *Geochim. Cosmochim. Ac.* **183**, 250–266 (2016).
32. Chakraborty, S. et al. Linkage between precipitation isotopes and biosphere-atmosphere interaction observed in northeast India. *npj Clim. Atmos. Sci.* **5**, 10 (2022).

33. Munksgaard, N. C. et al. Data Descriptor: Daily observations of stable isotope ratios of rainfall in the tropics. *Sci. Rep.* **9**, 14419 (2019).

34. Breitenbach, S. F. M. et al. Strong influence of water vapor source dynamics on stable isotopes in precipitation observed in Southern Meghalaya, NE India. *Earth Planet*. *Sci*. *Lett*. **292**, 212–220 (2010).

35. Sengupta, S. & Sarkar, A. Stable isotope evidence of dual (Arabian Sea and Bay of Bengal) vapour sources in monsoonal precipitation over north India. *Earth Planet*. *Sci*. *Lett*. **250**, 511–521 (2006).

36. Acharya, S. Yang, X., Yao, T. & Shrestha, D. Stable isotopes of precipitation in Nepal Himalaya highlight the topographic influence on moisture transport. *Quatern. Int.* **565**, 22–30 (2020).

37. Ansari, M. A., Noble, J., Deodhar, A., & Kumar, U. S. Atmospheric factors controlling the stable isotopes ($\delta^{18}O$ and $\delta^{2}H$) of the Indian summer monsoon precipitation in a drying region of Eastern India. *J. Hydrol.* **584**, 124636 (2020).
