# Supplementary material for: Reinterpreting precipitation isotope records from Asian monsoon region through the hidden role of atmospheric updraft
Source: Nat Commun. 2026 Sep 11;17:10728. doi: 10.1038/s41467-026-77635-5 (PMC13658188; doi:10.1038/s41467-026-77635-5)
Supplement: Supplementary file 2 [file 41467_2026_77635_MOESM2_ESM.pdf]

nature portfolio

Peer Review File

Reinterpreting precipitation isotope records from Asian monsoon region through the hidden role of atmospheric updraft

Corresponding Author: Professor Wusheng Yu

This file contains all reviewer reports in order by version, followed by all author rebuttals in order by version.

Attachments originally included by the reviewers as part of their assessment can be found at the end of this file.

Version 0:

Reviewer comments:

Reviewer #1

(Remarks to the Author)
In this manuscript, Zhang et al. compile δ18O data from speleothems and rainwater samples across the Asian monsoon domain: to explore the extent to which atmospheric updraft could explain spatially conflicting trends in δ18O signals, relative to established processes including 'rainout' and 'amount' effects. They suggest that their results pertain to a non-trivial influence of updraft as a driver of variations in δ18O on both short (<1-year) and long- (>1000-year) timescales, and that their work underscores an important consideration for interpretation of speleothem δ18O records in the future.
Overall, we believe this study will only be suitable for publication in Nature Communications once the concerns outlined in the attached PDF have been addressed and, hence, we recommend major revisions.

Reviewer #2

(Remarks to the Author)
I co-reviewed this manuscript with one of the reviewers who provided the listed reports. This is part of the Nature Communications initiative to facilitate training in peer review and to provide appropriate recognition for Early Career Researchers who co-review manuscripts.

Reviewer #3

(Remarks to the Author)
Speleothem δ18O records across the Indian and Asian monsoon domain have been frequently used to study past climatic changes. However, numerous interpretations have been put forward to describe their dominant controls, and therefore, the metric of climate change these records actually represent has remained somewhat unclear. Zhang et al. provide a revised interpretation of these based on a zonal analysis of modern rainfall δ18O as well as speleothem records δ18O distributed across the Asian monsoon domain. The authors suggest that updraft in the central portion of their zonal area explains a V-shaped pattern in rainfall δ18O values during the monsoon season. Box plotting speleothem δ18O values distributed on this zonal transect, they observe a similar pattern and suggest that updraft explains the zonal variation of speleothem δ18O values.

I have read the manuscript a few times. This has clearly been an ambitious and large body of work, and I find this research would be of broad interest and provocative. I do have several changes, further considerations and recommendations for additional analysis to be conducted prior to publication. The text may also need to be improved for clarity. However, despite having written a lot, I think many of my concerns are asking for further clarification and quantification to ensure the statistical robustness of the findings, and these changes should be achievable. After this, I believe the work would make an interesting contribution to understanding the variable influences on rainfall (and speleothem) δ18O values.

I have three larger concerns, which are the primary points I would like the authors to address.

Firstly, the authors seem to have used data differently throughout the manuscript. For example, on Fig. 2, we see that not all the sites in the pre-monsoon season are represented with δ18O values. Likewise, the gradients Fig. 4 (c,d,e,f) use different sites to construct each gradient, which means the slope analysis (Fig. 4g) is impacted by these choices, rather than solely the spatial variation across this whole transect. So, perhaps the authors might consider refocusing their analysis on the mid-section to the Eastern section, where they seem to have sufficient data? Instead, they may need to soften their “low in the East, high in the West”, “V-shape” and “westward migrations” claims, or rely more heavily on the modelling data to supplement these findings by bringing them into the main text/figure set up.

Secondly, at current, I think the authors require further work to validate the application to the speleothem record. This is in part because the most convincing match to their precipitation data (Fig. 6b) hasn’t been explained in sufficient detail in the main text or methods to adequately understand how these "multie-year" and relative values were generated. Furthermore, as I’ve stated in my section comments, the proposed trend in Fig. S21 requires additional statistical work to show 1) it is consistent with the regional δ18Op trend, and 2) matches the multi-year mean δ18Os or the regional precipitation data (Fig. 2).

Thirdly, I think the manuscript could be improved by increasing the analysis to more traditional climate variables (monthly precipitation amount and temperature). The authors could consider a more robust multiple variable test through multiple regression analysis, including these and OLR on monthly δ18O. The authors state (in the methods) that they have already collected additional variables, but these don’t seem to have been included beyond this. I think bringing them into the analysis would provide a more-rounded approach to understand the spatial variability of δ18Op in the region. If they do not have any significant impact on δ18Op, then this information could be shown as a supplement.

Comments on sections
Seasonal variations in δ18O
Eastern sites vs. western sites trend
The authors argue that there is a difference in the seasonal δ18O trends between eastern and western regions. Namely, that δ18Op declines from May to July in the east, and declines from May to October in the west. The westernmost sites do not cover the whole year. Is this because it is too dry? Having a faded bar chart of rainfall amount behind each boxplot (Fig. 1) would be helpful to understand the distribution of monthly rainfall at these sites.

Spatial distribution of δ18O
The altitude effect

The authors aim to discount the altitude effect to show that this is not a dominant control on rainfall δ18O. They claim that there is no significant effect of altitude on rainfall δ18O by performing regressions on data binned into 3 climate-based categories. However, the delineations seem to be based on altitude; as such, there’s no reason not to do the regression over the whole dataset (in other words, the regression needs to be conducted without binning the data). If there is still no significant effect, then the authors are correct. If not, then the altitude effect does impact an aspect of the spatial distribution of δ18Op, and this needs to be accounted for by the authors. If this is the case, it is not inherently a problem; the authors have shown that rainfall δ18O varies seasonally across the transect. However, the authors must simply conduct their subsequent analysis, including altitude as a metric (in a multiple regression, for example), or find another way to account for its effect.
If the authors intend to keep climate-based binned data, I suggest they use a system that does not explicitly include altitude to separate the sites (such as the Köppen-Geiger climate classification). However, as I wrote in the last paragraph, I do not think a significant regression with altitude across the whole dataset affects their seasonal component, and therefore, this would be a more preferable approach.

Additionally, from the way this is written (136-144), it is unclear if there is an altitude effect in the region or not. The authors state they find a significant altitude effect in the 250-800m bracket (although the p-value is > 0.05 and therefore not significant), then that there is an “inverse altitude effect” in the region, and then conclude that there is no significant effect on altitude.

Pre-monsoon “low in the East, high in the West”
The authors say they are using 12 sites, but only 9 are included here, with the westernmost seemingly having no data in the pre-monsoon season. Is there simply not enough data from these sites with missing values – are they too dry (this is why I think plotting monthly rainfall would be helpful for the readers)? For the remaining data, the authors may also consider having a quantification statistic for this claim. A Tukey test across all boxes, or a t.test (between sites lumped into East and West) would be helpful. In any case, they may need to provide a more cautious interpretation of the δ18O variation, since there is quite significant overlap between the boxes, which calls into question if the “westward migration” is actually significant.

Monsoon V-shape
I think the authors may need to rule out that the precipitation amount is not controlling δ18O at these sites. Is this V-shape present in the monthly precipitation amount? If these sites simply receive more rainfall, then can rainfall amount explain the speleothem record δ18O? Or if this updraft is actually correlated to rainfall amount (which seems possible), then how do we distinguish their effects? (Perhaps they are distinguishable, and it simply needs to be explained in plainer language).

δ18Op and the advection of monsoon moisture

Are the predictor variables correlated? In cases (Tab. S3) where there are two significant predictor variables, the correlations

are heading in the same direction (Tab S3). So it is likely they are correlated, and distinguishing their effects is more challenging. The authors may benefit from producing a correlation matrix of the predictor variables at each site.

δ18Op and atmospheric updraft

The δ18O and OLR gradient correlation requires further consideration because it includes different datasets at different points, thus, the gradient is influenced by which sites are included, rather than the monthly variations between sites alone. I think the authors need to decide on a set of sites to use in their analysis. If they don't have sufficient data from across all sites, then the data isn't sufficient for their claims.

296-307: is this what the authors are proposing as a conclusion? Or do they have observation/modelling/experimental/literature support? If so, the evidences need to be properly cited, or the authors should make the reader aware that they are proposing something.

Implications for speleothem paleoclimatology

I think the authors may need to conduct a little more work to ensure the statistical robustness of their claim that δ18Op trend from the cave sites matches the δ18Os trend. Can the authors address this by 1) conducting a Tukey test to test for significant differences between the δ18Op means? 2) Provide an additional boxplot of monsoon vs. pre-monsoon season δ18Op at the cave sites? 3) provide a correlation/regression between δ18Os and δ18Op

Methods
The authors state they obtained "vertical velocity (ω), OLR, specific humidity, meridional wind, zonal wind, geopotential height, precipitation amount and cloud cover", but only the OLR seems to be analysed. Have the others been analysed for their significance? If these aren't important, is it necessary to include them in the methods? Based on some of my other comments, though, I would like to see some (at least precipitation amount) considered and the specific climatologies at the cave sites considered.

Calculation of multi-year mean δ18Os values
There are insufficient details here about the relative value method and what the authors did to achieve the results displayed in Fig. 6b. Also, can you please explain how and why these sub-periods were defined?

682-683: Not quite sure what the relevance of including Fig. S23 here is?

Minor line comments
Abstract
57-60: This is confusingly written and ought to be improved for clarity.

Main text

There is some need for clarity on the mentioned effects. "Rain out" seems to have been used twice to mean different things. Do you mean the "Continental effect" (Dangaard, 1964)?

Line 57: "the" Asian monsoon region (definite article)

Line 88: if word count permits, a brief description of the "circulation effect" and "inverse temperature effect" would be useful for the general readership.

Line 93: Evidences -> Evidence (uncountable noun)

Line 125: "the" South Asian monsoon region (definite article)

136: the authors claim that the 250-800 m range shows a significant 'correlation' (this is actually a regression), but looking at the p value, whilst close, it is above 0.05 and therefore does not fulfil the typical criteria for statistical significance.

150: the "12 primary sites" are not all included in the pre-monsoon. The 9 of the sites are.

Line 151: follows -> follow (plural)

Line 379: reflects -> reflect (plural)

Line 687: Note "that" the (subordinating conjunction)

Line 680, 691, 810, 114, 145, 225, 320, 396, 401: multiyear -> multi-year (compound adjective)

Figures

Fig. 2 and Fig. 3. To save on figure caption word count, the authors could label the boxes with their respective months.

Fig. 3: line 771. The authors are missing “February” before the “(a)”?

Fig. 4: correlation coefficients should be reported with an italicised “r” opposed to “R”.
Since all discussion so far has focused on monthly/seasonal timescales, my personal opinion is that the correlation between daily δ18O and OLR (Fig. 4a) isn’t necessary. Can the authors please also run their monthly δ18O and OLR as a regression and plot the R2 value? There is no y-axis on Fig. 4c,d,e,f; where do the gradients come from?

Fig. 6: perhaps 6b would be clearer if the colour the boxes with the corresponding colours of the lines in 6a? Can you also explain in the caption how the delineations in 6a are defined?

Comments on Supplement
Fig. S1: Are these correlations or regressions? If they are correlations, the R should be written as an italicised “r”. It is also necessary to know if this is a spearman’s or pearson’s correlation and if the data fulfils the criteria for normal distribution. If they are regressions, the R value should be written as “R2” and the equation should be given on the chart.

Fig. S1. My concern with either a regression or correlation from this data is that the data does not look particularly linearly distributed. I wonder if the authors can show if the data is normally distributed or not? If not, this will help you to select the correct correlation/regression test for the data (as well as data transformation).

Fig. S4: These are not correlations; they are regressions (since they provide an output equation to predict the data). Why are they separated into 3 boxes, instead of simply regressing altitude and δ18O for all the sites? I think this needs clearer justification. Furthermore, if these are regressions, the R value is likely an R2 rather than a correlation coefficient (r).

Fig. S8, Fig. S9, Fig. S10: The different abscissa scales between the figures create an intensified visual effect, suggesting the spatial difference in $\delta^{18}O$ is much stronger than it is. Moreover, the abrupt change from blue to yellow colour strengthens that effect. I suggest using the same scale for all three figures and adapting the colour scheme to realistically portray the spatial distributions of simulated $\delta^{18}Op$.

Fig. S11. Is there a source for this information?

Fig. S15. The x-axis has been labelled as °N. I think this should be °E?

Fig. S21. Please provide the number of measurements for each box.

Fig. S22. Please can you plot the location of the caves on the map?

Supplementary excel files:
The file is not self-explanatory and would benefit from a small informational text and units in the column headers. The STDEV seems very large for the reported values (i.e., -11.37‰ with a STDEV of 10.00). Only two of the sites have been included (Baoshan and Hechi). The authors must include all the sites analysed. Moreover, they should include the additional metrics they are analysing, and include precipitation amount and temperature at the sites for each of the months, so that other researchers can reproduce their finding

Reviewer #4

(Remarks to the Author)
I co-reviewed this manuscript with one of the reviewers who provided the listed reports. This is part of the Nature Communications initiative to facilitate training in peer review and to provide appropriate recognition for Early Career Researchers who co-review manuscripts.

Version 1:

Reviewer comments:

Reviewer #1

(Remarks to the Author)
In this manuscript, Zhang et al. have made an extensive set of revisions in response to first review. Overall, we can see improvements in the structure and narrative of the manuscript, and the addition/revision of several key figures contributes to a better understanding of the processes and phenomena that the authors intend to resolve. Moreover, we are extremely grateful to the authors for taking so much time to consider, and respond to the first round of comments.

However, we are still struggling with the flow of logic in the manuscript. A common feature of all articles that do well in NCOMMS is that they are clear, and focused in what is used, shown, and described: all in service of addressing a core question. This study is extremely data-rich, to the point where it feels as though the reader is being overloaded with things to think about, and the core question is lost. For example, a lot of new tests and analyses are introduced almost continuously, but in a way that is often non-sensical. We found ourselves asking repeatedly: “what is this adding to the overall story?”, “why is this needed?”, and “what does it tell us?”. Furthermore, the authors provide a huge volume of information in their rebuttal letter, but following several reads, a lot of it seems superfluous to requirement and not directly relevant.

Some other important points:

- We fail to be convinced that the inclusion of paleo-records is valuable, or necessary. It almost felt like these records were included as an afterthought, and would be better presented in a separate paper entirely. Especially given that the authors note that they had merged datasets to create composite records in several cases. This is a procedure that is not simply 'layering' records on top of each other, and so needs far more description than is given in order to be robust, or justified.
- The frequent reference to figures and the large number of supplementary figures continue to impair the readability of the manuscript. We appreciate a large part of this is likely unavoidable given the considerable amount of data and analysis. However, reference to these materials can be streamlined substantially with statements such as: "Details of X and Y are provided in an additional supplementary file".
- The revised manuscript still exhibits some deficiencies in certain sections of the interpretation, and the structure of the arguments – all of which need to be addressed in order to make a convincing case that updraft is a critical mechanism, that could re-shape our understanding of speleothem-derived δ18O records.

Taken together, we believe that this study would be better suited to a longer-format journal, where each analytical component can be carefully laid out without the constraints of a tight word limit. In other words, we do not feel this is a NCOMMS-suitable article – given the volume of data, information, and detail needed in order to present a convincing case.

Reviewer #2

(Remarks to the Author)
I co-reviewed this manuscript with one of the reviewers who provided the listed reports. This is part of the Nature Communications initiative to facilitate training in peer review and to provide appropriate recognition for Early Career Researchers who co-review manuscripts.

Reviewer #3

(Remarks to the Author)
The authors provided comprehensive replies to the comments and have conducted a good amount of extra analysis. The authors have also provided a restructured manuscript and have improved the clarity of the writing and arguments. This revised manuscript provide is a much-refined version and is much easier to read. The additional references and analyses have provided further clarification.

I am pleased the authors have taken time to address the methodology for the relative values of the speleothems. One minor point I could make, is that, at current, the text relies the reader to assume why this method has been chosen. A sentence or two explaining why this method is appropriate, and what it achieves, would help the reader. (One could argue that, assuming no shifts in the moisture source, then, based on the authors' logic, we should always expect every speleothem record to have a predictable longitudinal offset, irrespective of time, and thus we shouldn't need the relative values?).

The additional work on the altitude effect I believe adds robustness to the authors' points. Indeed, the seasonal aspect shows that, whilst there is an altitude effect when one looks at annual precipitation, this is not present seasonally. Thus, showing that another alternative (and seasonally driven) factor is a dominant control on precipitation δ18O, and that correlation with altitude (at an annual scale) may not be causation.

However, I think it is very important that the authors do plot precipitation amount in the main text. I think rainfall data could be added to figure 2. The authors would need a secondary axis (on the right) and simply add the precipitation from fig R22 underneath the δ18Op boxplots. I really think this helps to demonstrate that δ18Op and rainfall amount are disconnected.

Whilst I agree with the authors findings (and that vertical processes have been overlooked), I still suggest that they may wish to future-proof their work with a few words of caution in the text. One of the short-comings of much of the δ18Op work in the Asian monsoon region is that papers frequently advocate for a new understanding of δ18Op and overturning of previous work, only to be superseded a few years later. Likewise, as I alluded to in my previous review, traditional factors (e.g., rainfall amount) as well as others may still have another (perhaps less dominant) effect on the δ18Op. This is why I suggested to conduct a multiple regression with numerous variables along this specific transect (not to try to trip up the authors, but to expand understand the contribution of multiple factors). So, perhaps the authors may wish to apply a little caution and avoid this trap by 1) do not state that there is a "false-altitude effect", but simply explain (as they have) how vertical processes independent of terrain elevation can provide additional information on δ18Op, and 2) add to their conclusion a statement about how future research in the region should develop (integration of vertical processes, horizontal processes, altitude, moisture source, etc) to provide a holistic (rather than single effect) model for understanding δ18Op. I admit, however, that much of this is stylistic and a matter of personal taste.

With exemption of these comments, I think this manuscript does indeed make an interesting contribution to understanding the isotopic composition of rainfall in the South Asian and East Asian monsoon region. The text has been substantially improved and the authors make a convincing case that vertical processes, rather than only horizontal processes, impact δ18Op. I think this would make an interesting published article and I am supportive of their work.

Specific line and figure comments

Line 80: “Error! Reference source not found”
Line 93: “dependent on the ratio of water vapor originating from distant oceans and local ocean”
“Dependent on the ratio of water vapor originating from distant oceans and a local ocean to an observed site”
Line 92-94: Is this not just a reformulation/localisation of the source water effect?
Line 97: “Error! Reference source not found”
Line 180-181: “Error! Reference source not found”
189: What is meant be monsoon intensity? Amount of rainfall? Wind strength?
Line 236: “reduce” reduces
Line 318: “support” supports
Line 442: “https://doi.org/”
Line 471: “ofthe” -> the
Line 569: “49. Baker, A. J. et al. Seasonalityof westerly moisture transport in theEast Asian
summer monsoon and itsimplications for interpreting precipitation δ18O. J. Geophys. Res. Atmos. 120, 5850—5862 (2015).”
“Seasonalityof” -> Seasonality of
“theEast” -> The East
Line 570: “itsimplications” -> its implications
Line 676: : -> , (or delete “including”)
Line 698: - tabstop
Line 727: note that in this study

Fig. S15. Over which time period where these data collected? What is the source of the data?

Reviewer #4

(Remarks to the Author)
I co-reviewed this manuscript with one of the reviewers who provided the listed reports. This is part of the Nature Communications initiative to facilitate training in peer review and to provide appropriate recognition for Early Career Researchers who co-review manuscripts.

Reviewer #5

(Remarks to the Author)
Review report: Zhang et al: Re-interpreting Asian speleothem Isotope records: the hidden role of Atmospheric updraft.

Zhang et al. present an impressive synthesis of published d18O precipitation data across the Asian domain and develop a clear, testable hypothesis: that variability in d18O precip. is sensitive to atmospheric dynamics, particularly processes linked to vertical motion (e.g., updraft strength).

This is a commendable effort and a step in the right direction. However, the manuscript overlooks a fundamental piece of the argument that cannot be ignored. As currently narrated, it implicitly assumes that d18O in precipitation is transferred directly to cave drip waters and then faithfully preserved in speleothem calcite. This assumption is not justified (see for eg Gebregiorgis et al., 2020, Quaternary and references therein for a concise context and starting point on re-interpreting Asian speleothems) and represents a major flaw because it bypasses an extensive body of literature on the complexities of karst hydrology and how these processes shape speleothem d18O proxy formation. For example, see (Chen and Li, 2018, GCA; Zhang and Li, 2019, J. Hydrology) for the implications of in-cave processes for d18O and dD of drip water; (e.g., Duan et al., 2016, GCA) for storage and mixing in the epikarst; residence-time effects (e.g., Qiu et al., 2023, J. Hydrology and references therein); selective recharge/seasonal weighting (e.g., Duan et al., 2016, GCA; Tian et al., 2021, Frontiers in Earth Science); evaporation (e.g., Hendy, 1971, GCA for foundational insight; Markowska et al., 2016, QSR; Deininger et al., 2012, GCA; Duan et al., 2016, GCA); prior calcite precipitation (e.g., Patterson et al., 2024, Nature Communications); and cave ventilation effects (e.g., Lyu et al., 2024, Sci Total Environ), to name a select few, that we know to attenuate, lag, or even decouple the precipitation signal from the speleothem record. Addressing this properly would require an extensive synthesis of cave-drip d18O observations (which are the closest approximation to d18O in speleothem not d18O precip.) and karst hydrologic controls. For this reason, I am unfortunately recommending rejection of the manuscript.

I very much appreciate the effort in addressing the reviewers’ comments and agree that the proposed atmospheric updraft mechanism may plausibly explain variability in d18O precipitation. However, it cannot be extended to d18O in speleothems without a rigorous analysis of cave drip-water d18O data and consideration of karst geology and hydrology. As such, the manuscript makes an unsupported leap from precipitation isotopes to speleothem calcite isotopes, and the evidence presented cannot be used to critically interpret speleothem d18O or warrant publication in Nature Communications.

That said, the d18O precipitation-focused synthesis and atmospheric hypothesis would be a good fit in a more discipline-oriented journals such as GRL, but this would require substantial reframing of the manuscript both for scope and the broader implication of the work re: speleothem d18O proxy formation.

Minor comments (general)

The debate on the interpretation of Asian speleothem d18O records is now decades old and was initially ignited by discrepancies between speleothem-based reconstructions and marine-based proxies of the Asian monsoon (e.g., Clemens et al., 2010, Paleoceanography; Gebregiorgis et al., 2018, Nature Communications; Clemens et al., 2021, Science

Advances), rather than as implied in lines 83–90.

I am not convinced by the statement that the traditional view is that "$\delta^{18}O$ of precipitation is primarily driven by South Asian monsoon moisture advection" (line 296). What is the evidence for this claim?

Re: Mechanisms of atmospheric updraft changes

Line 397–400: Please cite appropriate references here, as there is no conclusive evidence (at least to me) to support the statement that speleothem d18O primarily reflects a climate signal at annual timescales. Are you referring to the temporal resolution of the speleothem records? If so, please state this explicitly. More importantly, why not investigate this claim using available cave drip-water isotope data instead of the 12 precipitation sites selected, which provide the closest modern analogue to d18O recorded in speleothem calcite?

Version 2:

Reviewer comments:

Reviewer #3

(Remarks to the Author)
The authors have made substantial revisions to the text and I appreciate that the authors have provided a section explaining the requirement for further research and provided caveats to their model. As pointed out by another reviewer, the application to the speleothem record will of course require specific in cave monitoring to understand karst processes influencing δ18O variability, and I think removal of the speleothem section does help the flow of the paper. Overall, I find this an interesting paper worth discussion. However:

As I have noted previously, my primary concern with this work is that the authors have not checked for the correlation of precipitation amount with δ18O (as done for OLR/vertical velocity). Neither have they checked the correlation between precipitation amount and OLR/vertical velocity. My suspicion is that OLR/vertical velocity are strongly correlated with rainfall amount (as alluded to in Figure 6 [which is a lovely figure]) since they are meteorologically linked, meaning that the influence of either may not be statistically separated. Checking these is necessary since rainfall amount is a key influence of rainfall δ18O. The authors should also check the AIC scores for models running OLR/vertical velocity against δ18O with and without precipitation amount included as an additional independent (x) variable, which would show whether the model's performance is better or worse with this included.

The authors have previously provided literature-based reasons for not doing this, but given that the authors 1) have the data; 2) there does seem to be some visual relationship between δ18O and precipitation amount in Fig. 2; 3) there is a well-known process-based understanding of precipitation amount effect on δ18O; 4) convection and rainfall are linked, this is necessary to account for the effect of rainfall amount, distinguish effect of OLR/vertical velocity and confirm the authors results.

The authors previously argued that doing so would detract from the novelty of their work. I respectfully disagree. It is a process to ensure the robustness of the interpretation, and could even help their interpretation. Until this is done, whilst finding this work very interesting, I am finding it difficult to determine whether the results represent a significant advancement based on reasoned statistics, or whether this is an incomplete analysis of the system.

Minor comments

Line 247: Why is this unfortunate? I don't think "unfortunately" is in context.
Line 248: "correlations change over time from weak positive, weak negative, and then to strong negative". If the correlations are not significant, they don't need to be described as "weak positive" and "weak negative". There is just no significant correlation. Likewise, there is no need to described significant correlations as "strong negative". They are just significant negative correlations.
Line 253: We have discussed this previously and I think the authors are right that the seasonal variation shows terrain (altitude) isn't a primary driver of the spatial δ18O variability. But, "absent negative δ18OP-altitude correlations" what does this mean? There is either no significant correlation ($P > 0.05$) or a significant correlation ($P < 0.05$).

Line 259-263: "Unlike other monsoon regions (e.g., Africa), precipitation in the Asian monsoon region is influenced not only by monsoonal circulation but also by the combined effects of the Intertropical Convergence Zone (ITCZ)" The African Summer Monsoon (as well as the Indian Summer Monsoon and the South American Monsoon) is indeed strongly influenced by the ITCZ. ITCZ can be removed from the list of influences. I think I know what the authors mean, but it's a bit awkwardly phrased.

Reviewer #5

(Remarks to the Author)
Review report: Zhang et al: Re-interpreting Asian speleothem Isotope records: the hidden role of Atmospheric updraft.

I am reviewing the manuscript by Zhang et al. for the second time. Previously, I recommended rejection of the manuscript

despite the impressive synthesis of published d18O precipitation data across the Asian domain presented in the previous version of the manuscript. I base my recommendation then based on the implicit and incorrect assumption that d18O in precipitation is transferred directly to cave drip waters and then preserved in speleothem calcite. This has now been thoroughly corrected, and I would like to commend the authors for the effort. As the manuscript currently stands, I believe it has the potential to stimulate productive discussion about the mechanistic interpretation of d18O signals faithfully preserved in the Chinese speleothem archives (See Implications and Outlook). I therefore recommend publication of the manuscript pending minor revisions and I would like to commend the authors once again for the constructive engagement.

Minor comments (general)

Line 82-83:
Previous studies have widely applied Asian δ18Os records as a proxy for monsoon precipitation and/or monsoon intensity7,8,20-25.

To improve clarity, I suggest you divide the references: monsoon precipitation (Refs) and monsoon intensity (Refs). Note that monsoon precipitation and monsoon intensity describe related but still different aspects of the monsoon system even if the two terms are used interchangeably in the literature.

Line 83-85: Similarly, I suggest dividing the references according to archive type for clarity and to make it easier to access the cited references, particularly for readers outside the field. Please also consider doing the same for the rest of the text.

Line 168: Rephrase (repetitive):

Considering that atmospheric updraft cannot be ignored
Understanding how atmospheric updraft influences $\delta^{18}O_p$ changes at precipitation sites is important.

Line 247: Rephrase (retain some nuance but still reduce adjectives)

Unfortunately, the results show that correlations change over time from weak positive, weak negative, and then to strong negative (Fig. 3c), reflecting the westward expansion or migration of low δ18Op from the pre-monsoon to monsoon seasons.

The results show that correlations change over time from positive to negative (Fig. 3c), reflecting the westward expansion or migration of low $\delta^{18}O_p$ from the pre-monsoon to monsoon seasons.

Line 259-263: Please add references for each.

Unlike other monsoon regions (e.g., Africa) (e.g., refs), precipitation in the Asian monsoon region is influenced not only by monsoonal circulation but also by the combined effects of the Intertropical Convergence Zone (ITCZ) [ref], the Indo-Pacific Warm Pool (IPWP) (e.g., refs), and the Western Pacific Subtropical High (WPSH) (e.g., refs).

Reviewer #6

(Remarks to the Author)
In this manuscript, Zhang and co-authors provide novel perspectives into the importance of atmospheric updraft in explaining water isotope variability of precipitation (d18O) in the Asian monsoon region. In particular, they utilized d18O rain gauge data (GNIP) along with atmospheric reanalysis/observations to illustrate how the seasonal evolution of d18Op in the Asian monsoon sector can best be explained by the seasonal cycle of updraft circulation. The authors argue that updraft dynamics explains the local minima in d18Op in the central Asian Monsoon sector during the summer monsoon season, which would not be expected from a purely large-scale advection rainout process, which should always show more depleted values downstream in East Asia. Notably, these findings have important implications for our interpretation of d18O of calcite records and our understanding of past hydroclimate variability.
This is my first time reviewing the manuscript although I recognize that the authors have addressed a previous round of comments and made significant revisions in response to them. Overall, I think the manuscript presents important contributions to the field and would be well suited for eventual publication in a high-impact journal such as Nature Communications. I do have several comments (2 major and a few minor) that I believe should be addressed prior to publication that will improve the clarity and logic of the manuscript.
Major comments:
1. Local minima of d18Op: The authors present a detailed discussion of how atmospheric updrafts can lead to local rainout effects that increasingly deplete the oxygen isotopes of the residual moisture, thus leading to the decreasing d18Op in regions of high updrafts. However, I am a little confused as to how this d18Op increases once again further downstream. Take Figure 2g as an example. I understand how there is a decrease in d18Op around Baoshan due to high updrafts at this longitude (Figure 3b, August), but why does the d18Op increase again further east (downstream) if the residual moisture of the air parcel that arrives here has already been depleted from the updrafts? Is because the East Asian d18Op is somewhat Pacific sourced, replenishing its d18Op? To me, if updrafts are the only/primary factor controlling d18Op, I would expect not a “V” shape in d18Op, but an “L” shape where the angle corresponds to where updrafts occur. Perhaps I am missing something?

2. Quantifying updraft contribution to d18Op variability: I am not entirely convinced by the quantification analysis in Figure 4. Figure 4a,b show that a large fraction of d18Op variability cannot be explained by local updrafts (correlation coefficient of only 0.4-0.48), suggesting other factors are important. The authors then compute the slopes of both OLR and d18Op as an east-west transect across the GNIP stations and correlate them to illustrate high correlation (r=0.90) and thus strong influence of updrafts. However, I think that this correlation of monthly slopes does not properly illustrate the noise in their data, as clearly is present from Figure 4a,b. There is no representation of how well each of these monthly slope values explain the data (no RSME of regressions, no significance testing), thus its unclear how well these slopes actually represent the variability. In my mind, the high correlation in Figure 4g demonstrates that qualitatively, the timing of slope changes are largely synchronized between OLR and d18Op, which we can see visually in Figure 2,3. But it does not provide a precise quantification or explained variance of the role of updrafts.
Minor comments:
1. I think Figure 1 would look nicer if panel b and c were vertically stacked, that way the readers could easily see the differences in seasonal timing along the x axis between the east Asian and south Asian sites.
2. I found myself hopping between Figure 2 and Figure 3 to make the connections between d18Op and the atmospheric properties from a seasonal cycle perspective. Is there a way to make these two plots more connected, either by adding the d18Op to Figure 3 or otherwise? Also I found the column c to be a little distracting, given that it is there to primarily make a negative point (perhaps this was in response to another reviewer's comment).
3. I think Figure 5 is not that useful for illustrating the influences of ITCZ, IPWP, and WPSH on updrafts (which I believe is the author's goal). I think something a bit more quantitative such as showing actual updraft velocities in specific regions versus ITCZ location or otherwise would be more helpful to establish the dynamical links.
4. As someone more familiar with tropical climate, I'm used to thinking of OLR as reflective of cloud cover and heavy rainfall, not necessarily updraft strength (although I recognize that the two are related). Could the authors include some more analysis (like correlations) that demonstrate how OLR is a robust proxy for updrafts (vertical velocities)?

Version 3:

Reviewer comments:

Reviewer #3

(Remarks to the Author)
Please see attached document

Reviewer #6

(Remarks to the Author)
I would like to thank the authors for their extensive work in revising the manuscript in consideration of my previous round of comments. The paper has greatly improved in terms of clarity, robustness, and balance of results. I have only two remaining comments on Figure 5 as well as the discussion/outlook before recommending for publication.

Figure 5 analysis:
I appreciate the authors significant efforts in improving the discussion of the various mechanisms that control updrafts in the Asian monsoon region, especially with the new Figure 5 which aims to be more quantitative.
While I believe that Figure 5i,j,k provide good quantitative support that ITCZ latitude shapes OLR (and by association updrafts) variability, I personally don't find Figure 5a-h that useful in this context. These 8 subplots mainly serve to illustrate the seasonal migration of the ITCZ in the three domains but don't provide much else. The text argues that the timing of these ITCZ positions contribute to various processes that increase updrafts (such as frontal lifting) but there is no demonstration of the timing of these circulation changes in the Figure 5 itself. I would recommend perhaps collapsing Figures 5a-h into a single subplot that visualizes the seasonal cycle of ITCZ migration, which should make room for additional analysis. Furthermore, the main text which discusses Figure 5 argues for the influences of the IPWP and WPSH, but much of this analysis is buried in the supplement. I recognize the space constraints of Nature Communications but I think showing some metric of these two features (IPWP and WPSH) and how they shape updrafts in the Asian monsoon in the Main Text would be valuable.

Discussion and outlook:
The authors main point, that atmospheric updrafts play an important role in Asian monsoon d18Op variability, have important implications for a range of climate studies. While the authors acknowledge the implications for interpreting paleoclimate records, I think it is also worth discussing how updraft dynamics may explain biases in simulations of water isotope distributions in climate models. Could biases in model characterizations of convective updrafts explain offsets between isotope enabled models and observations? Furthermore, does this finding of updraft importance perhaps have implications for understanding drivers of other monsoon systems and their d18Op? I would appreciate some discussion of these points.

Version 4:

Reviewer comments:

Reviewer #6

(Remarks to the Author)
I would like to thank the authors for their detailed work in addressing my previous round of comments. I have no more additional comments and now recommend for publication.

Open Access This Peer Review File is licensed under a Creative Commons Attribution 4.0 International License, which permits use, sharing, adaptation, distribution and reproduction in any medium or format, as long as you give appropriate credit to the original author(s) and the source, provide a link to the Creative Commons license, and indicate if changes were made.
In cases where reviewers are anonymous, credit should be given to 'Anonymous Referee' and the source.
The images or other third party material in this Peer Review File are included in the article's Creative Commons license, unless indicated otherwise in a credit line to the material. If material is not included in the article's Creative Commons license and your intended use is not permitted by statutory regulation or exceeds the permitted use, you will need to obtain permission directly from the copyright holder.
To view a copy of this license, visit https://creativecommons.org/licenses/by/4.0/

Response to Reviewers

(Reviewers' statements in black, and our response in blue)

Line163, -the line 163 in the previous manuscript.

L4-L5, -the lines 4-5 in the revised manuscript.

Fig. 1, -the Figure 1 in the revised manuscript.

Figure R1, -the Figure 1 in the response.

REVIEWER COMMENTS

Reviewer #1 (Remarks to the Author):

In this manuscript, Zhang et al. compile $\delta^{18}O$ data from speleothems and rainwater samples across the Asian monsoon domain: to explore the extent to which atmospheric updraft could explain spatially conflicting trends in $\delta^{18}O$ signals, relative to established processes including 'rainout' and 'amount' effects. They suggest that their results pertain to a non-trivial influence of updraft as a driver of variations in $\delta^{18}O$ on both short (<1-year) and long- (>1000-year) timescales, and that their work underscores an important consideration for interpretation of speleothem $\delta^{18}O$ records in the future.

Overall, we believe this study will only be suitable for publication in Nature Communications once the concerns outlined in the attached PDF have been addressed and, hence, we recommend major revisions.

Re: We thank you very much for providing very fair and constructive comments and suggestions on our manuscript. Your comments and suggestions have significantly improved the quality of our manuscript. We have tried to address all the concerns raised by you and have carefully revised the manuscript as follows.

Review of manuscript NCOMMS-25-23387-T:

"Reinterpreting Asian speleothem isotope records: the hidden role of atmospheric updraft"

Note: Alongside the primary reviewer, this manuscript was co-reviewed by an ECR researcher as part of the Nature Communications initiative to facilitate training in peer review and to provide appropriate recognition for Early Career Researchers who co-review manuscripts. Hence, the feedback given in this report reflects the collective

judgement of both reviewers.

GENERAL COMMENTS

In this manuscript, Zhang et al. compile $\delta^{18}O$ data from speleothems and rainwater samples across the Asian monsoon domain: to explore the extent to which atmospheric updraft could explain spatially conflicting trends in $\delta^{18}O$ signals, relative to established processes including 'rainout' and 'amount' effects. They suggest that their results pertain to a non-trivial influence of updraft as a driver of variations in $\delta^{18}O$ on both short (<1-year) and long- (>1000-year) timescales, and that their work underscores an important consideration for interpretation of speleothem $\delta^{18}O$ records in the future.

Overall, we believe this study will only be suitable for publication in Nature Communications once the following concerns have been addressed:

Re: We thank you very much for providing very fair and constructive comments and suggestions on our manuscript. Your comments and suggestions have significantly improved the quality of our manuscript. We have tried to address all the concerns raised by you and have carefully revised the manuscript as follows.

1. The manuscript is written in a way that rests heavily on description of the figures, rather than explanation of how they can be interpreted. The reader is constantly referring to the figures in order to make sense of what the authors are describing in the text, and then these descriptions are often followed by 1-2 bold claims stating what they imply. However, we found none of these statements convincing in the absence of clear, mechanistic explanations for why the patterns were there in the first place. Perhaps the authors assumed that the reader would have a certain degree of prior knowledge in this field, but even if so, the narrative was incredibly hard to follow; to the point where we had to read certain passages several times to make sense of them. Moreover, the authors referred to a new "framework" for interpretation of $\delta^{18}O$ variability in speleothems, but we were hard-pressed to find a framework that could actually be applicable to future studies; beyond simply considering that updraft may be important to consider.

Re: We thank you for pointing these issues out. We have tried to address all the issues as follows:

1) With regard to the writing style, this style might be related to a large body of work involved in our study, because:

a) Spatially, our study is conducted on the transect from the east coast of China to the west coast of India. Firstly, based on previous studies, we divided this transect into the eastern and western domains that correspond to the East Asian and South Asian monsoon regions, and then introduced each site from east to west.

b) Temporally, we divided the seasons into the pre-monsoon (February–May), monsoon (June–September), and post-monsoon seasons (October–January), and then need to analyze each month within each season.

c) In terms of parameters, in addition to $\delta^{18}O_p$ and $\delta^{18}O_s$, they also involve OLR, vertical velocity, moisture flux, ITCZ, IPWP, and WPSH, and the analyses of these parameters are also carried out accordingly for each month.

2) With regard to the absence of a mechanistic explanation for why the patterns were there in the first place and the presumption of a knowledgeable readership, following your comments, we carefully reviewed the entire manuscript and found that some sections do indeed have such issues. For example, in the section "$\delta^{18}O_p$ and atmospheric updraft", we assumed that readers will understand that a stronger atmospheric updraft will result in lower precipitation isotopes. That is, when we discuss how atmospheric updraft affect the variations in $\delta^{18}O_p$ along the transect from the east to the west during the pre-monsoon to monsoon, and to post-monsoon seasons, and even discuss their correlation between $\delta^{18}O_p$ and OLR (atmospheric updraft), we have always assumed that the readers have the prior knowledge. It is not until the end of this section that we use a paragraph to explain how atmospheric updraft affects the variation in $\delta^{18}O_p$. Clearly, we reversed the logical sequence of the text. Following your comment, we moved the description of how atmospheric updraft affects the variations in $\delta^{18}O_p$ in the last paragraph of this section to the beginning. This restructuring provides essential background knowledge at the beginning of this section, thereby readers can better understand our subsequent analyses. Please see L225–L236 in the revised manuscript.

Another example concerns the IsoGSM2 model simulations (lines 104-105 of the previous manuscript). In the previous manuscript, we assumed that readers already

had an understanding of IsoGSM2 model. Following your comments, we added an explanation about the simulation capabilities of IsoGSM2 model in the Introduction section, i.e., "*Additionally, we utilized* $\delta^{18}O_p$ *simulations from the IsoGSM2 model*[54] *to assess our results, because the model can reliably capture the signals of observed* $\delta^{18}O_p$[39]*"* Please see L114–L115 in the revised manuscript. We addressed all similar issues (such as the ~98°E dividing line, OLR as an indicator of atmospheric updraft) one by one. Please see L123–L137 and L210–L211 in the revised manuscript.

Reference:

39. Chiang, J. C. H., Herman, M. J., Yoshimura, K., & Fung, I. Y. Enriched East Asian oxygen isotope of precipitation indicates reduced summer seasonality in regional climate and westerlies. Proc. Natl. Acad. Sci. U.S.A. 117, 14745–1475 (2020).
54. Yoshimura, K., Kanamitsu, M., Noone, D. & Oki T. Historical isotope simulation using Reanalysis atmospheric data. J. Geophys. Res. Atmos. 113, D19108 (2008).

3) With regard to the statement of the new "framework", we agree with you. Following your comments, we changed "new framework" to "*new perspective*." Please see L432 in the revised manuscript. That is, in future studies interpreting $\delta^{18}O_s$ signals, we emphasize that atmospheric updraft ("vertical perspective") plays a important role in controlling Asian $\delta^{18}O_s$ variations than the traditionally emphasized horizontal moisture advection ("horizontal perspective"). To further make clear the "new perspective", we also changed "Our study highlights that atmospheric updraft plays a more important role in controlling Asian $\delta^{18}O_s$ variations than previously recognized. Our hypothesis offers a new perspective on the interpretation of Asian $\delta^{18}O_s$ records." to "*Unlike the conventional view, our study highlights that atmospheric updraft plays an important role in controlling Asian* $\delta^{18}O_s$ *variations. Compared with the traditional interpretation focused on horizontal processes, our hypothesis offers a new perspective emphasizing vertical atmospheric dynamics.*" Please see L64–L67 in the revised manuscript.

We suggest that the authors make major revisions to the structure and narrative of the manuscript, where they…

(a) Walk the reader through each analytical step carefully and thoroughly, and providing sufficient description of the processes, and phenomena, that could be underpinning the emerging patterns. These steps could then be more clearly linked to the research objectives stated at the beginning, and allow the reader to place the findings into a broader context.

(b) Consider all relevant alternative explanations for their observations – to give the reader further confidence that their interpretations are well grounded, and not excessively deterministic.

(c) Clearly link their observations and results to the main objective (and novelty) of the study, which we understood to be: to explore the extent to which atmospheric updraft controls the $\delta^{18}O$ signals encoded in speleothems across the Asian monsoon domain; relative to more established factors (e.g., rainout and amount effects).

Re: We thank you for pointing these issues out. We have tried to address all the issues as follows:

1) With regard to "the structure and narrative of the manuscript", following your suggestion, we made significant modifications to the structure of the manuscript, as follows:

First, we reevaluated the scientific significance of the "Seasonal variations of $\delta^{18}O_p$" section. We found that the purpose of this section is to reveal that there are differences in the seasonal variations of $\delta^{18}O_p$ at each site in the eastern and western domains along the transect, with a dividing line around ~98°E: $\delta^{18}O_p$ values in the eastern domain exhibit continuous decreases from May to July/August, followed by an increase in August/September, whereas in the western domain $\delta^{18}O_p$ values continue to decline from May through October. Strictly speaking, this section still belongs to the differences in spatial patterns - the overall differences between the eastern and western domains; moreover, this section contributes relatively little to the entire manuscript. Hence, we merged the "Seasonal variations of $\delta^{18}O_p$" section into the "Seasonal variations and spatial distributions of $\delta^{18}O_p$" section following your suggestion. Please see L122–L137 in the revised manuscript.

Second, as you mentioned, the finding regarding the dividing line around ~98°E does not break new ground compared to previous studies. We carefully checked the published work and found that some scientists including Jin & Chen (1982) and Wang & LinHo (2002) had already demonstrated that ~98°E is the dividing line between the South Asian monsoon and the East Asian monsoon from the perspective of atmospheric dynamics. Thus, the dividing line revealed by the $\delta^{18}O_p$ data in our study essentially serve as another line of evidence for the conclusion. Following your suggestion, we rewrote this section. That is, we first cited the previous study regarding ~98°E as the dividing line of the Asian monsoon region, and then analyzed the seasonal variation of $\delta^{18}O_p$ in the eastern and western domains, and ultimately confirmed the significant differences in seasonal variation of $\delta^{18}O_p$ between the eastern and western domains. Please see L123–L137 in the revised manuscript.

Third, since the "Seasonal variations and spatial distributions of $\delta^{18}O_p$" section belongs within the Results section, it is unsuitable to include any discussion in this section. Hence, the discussion about whether $\delta^{18}O_p$ are affected by altitude effect (Lines 130-144 in the previous manuscript) should not appear in this section. To address this issue, we moved the previously discussion on the altitude effect in the "Seasonal variations and spatial distributions of $\delta^{18}O_p$" section to the "$\delta^{18}O_p$ and atmospheric updraft" section. Please see L302–L319 in the revised manuscript.

Finally, in the section "$\delta^{18}O_p$ and atmospheric updraft", we assumed that readers will understand that a stronger atmospheric updraft will result in lower precipitation isotopes. That is, when we discuss how atmospheric updraft affect the variations in $\delta^{18}O_p$ along the transect from the east to the west during the pre-monsoon to monsoon, and to post-monsoon seasons, and even discuss their correlation between $\delta^{18}O_p$ and OLR (atmospheric updraft), we have always assumed that the readers have the prior knowledge. It is not until the end of this section that we use a paragraph to explain how atmospheric updraft affects the variation in $\delta^{18}O_p$. Clearly, we reversed the logical sequence of the text. Following your comment, we moved the description of how atmospheric updraft affects the changes in $\delta^{18}O_p$ in the last paragraph of this section to the beginning. This restructuring provides essential background knowledge at the beginning of this section, thereby readers can better understand our subsequent analyses. Please see L225–L236 in the revised manuscript.

Reference:

55. Jin, Z. & Chen, L. On the medium-range oscillation of the East Asia monsoon circulation system and its relation with the Indian monsoon system. *In The National Symposium Collections on the Tropical Summer Monsoon* 204-215 (People's Press Yunnan Province, 1982).

56. Wang, B., & LinHo, Rainy Season of the Asian–Pacific Summer Monsoon. *J. Climate* **15**, 386–398 (2002).

2) With regard to provide detailed descriptions of each analytical step, following your suggestion, we provided detailed and comprehensive descriptions of each analytical step, and also elaborated on the processes and phenomena that support the emerging patterns. We found that such improvements do indeed enable each analytical step to be more clearly linked to the research objectives stated at the beginning, and allow the reader to place the findings into a broader context. For example:

a) With regard to the ~98°E dividing line, as mentioned above, we rewrote this section. That is, we first cited the previous study regarding ~98°E as the dividing line of the Asian monsoon region, and then analyzed the seasonal variation of $\delta^{18}O_p$ in the eastern and western domains and ultimately confirmed the significant differences in seasonal variation of $\delta^{18}O_p$ between the eastern and western domains. Please see L123–L137 in the revised manuscript.

b) With regard to the mechanism that the influence of atmospheric updrafts on $\delta^{18}O_p$, in the previous manuscript, we assumed that readers will understand that a stronger atmospheric updraft will result in lower precipitation isotopes. That is, when we discuss how atmospheric updraft affect the variations in $\delta^{18}O_p$ along the transect from the east to the west during the pre-monsoon to monsoon, and to post-monsoon seasons, and even discuss their correlation between $\delta^{18}O_p$ and OLR (atmospheric updraft), we have always assumed that the readers have the prior knowledge. It is not until the end of this section that we use a paragraph to explain how atmospheric updraft affects the variation in $\delta^{18}O_p$. Clearly, we reversed the logical sequence of the text. Following your comment, we moved the description of how atmospheric updraft affects the changes in $\delta^{18}O_p$ in the last paragraph of this section to the beginning. This

restructuring provides essential background knowledge at the beginning of this section, thereby readers can better understand our subsequent analyses. Please see L225–L236 in the revised manuscript.

c) With regard to the auxiliary evidence from the IsoGSM2 model simulations, in the previous manuscript, we assumed that readers already had an understanding of IsoGSM2 model. Following your comments, we added an explanation about the simulation capabilities of IsoGSM2 model in the Introduction section, i.e., "*Additionally, we utilized $\delta^{18}O_p$ simulations from the IsoGSM2 model*[54] *to assess our results, because the model can reliably capture the signals of observed $\delta^{18}O_p$*[39]*"* Please see L114–L115 in the revised manuscript.

d) With regard to the difference between primary sites and ancillary sites, we added one paragraph to make them clear, i.e., "*To further reduce possible latitude effects on $\delta^{18}O_p$, we identified 12 primary sites (Supplementary Table 1) within the 46 sites, based on the following criteria: (1) observation sites located within a narrow latitudinal band of 1°±1.5°; (2) availability of high-precision (daily) $\delta^{18}O_p$ observations; and (3) maximum possible observation duration. The remaining 34 sites served as ancillary sites to validate the spatial patterns of the primary sites (Supplementary Table 2).*" Please see L687–L692 in the revised manuscript.

e) With regard to the division of the pre-monsoon, monsoon and post-monsoon seasons, following you suggestion, we added one sentence to explain why we used the definition of the pre-monsoon, monsoon, post-monsoon seasons, i.e., "*Given that circulation fields and precipitation amount in the Asian monsoon region exhibit distinct seasonal variations*[50]*, we analyzed the spatial distribution of $\delta^{18}O_p$ values at the 12 primary sites during the pre-monsoon (February–May) (Fig. 2a-d), monsoon (June–September) (Fig. 2e-h), and post-monsoon (October–January) seasons (Supplementary Fig. 4).*" Please see L139–L144 in the revised manuscript.

Reference:

39. Chiang, J. C. H., Herman, M. J., Yoshimura, K., & Fung, I. Y. Enriched East Asian oxygen isotope of precipitation indicates reduced summer seasonality in regional climate and westerlies. Proc. Natl. Acad. Sci. U.S.A. **117**, 14745–1475

(2020).

54. Yoshimura, K., Kanamitsu, M., Noone, D. & Oki T. Historical isotope simulation using Reanalysis atmospheric data. J. Geophys. Res. Atmos. **113**, D19108 (2008).

50. Zhang, J. et al. Coupled effects of moisture transport pathway and convection on stable isotopes in precipitation across the East Asian monsoon region: Implications for paleoclimate reconstruction. J. Climate **34**, 9811–9822 (2021).

3) With regard to all relevant alternative explanations for the observations, in this study, we highlighted that the $\delta^{18}O_p$ along the east–west transect of the Asian monsoon region shows a "low in the east, high in the west of the dividing line (~98° E)" pattern during the pre-monsoon season, and a V-shaped pattern during the monsoon season. There might be three alternative explanations that explain these patterns, namely precipitation amount, advection of monsoon moisture, and altitude. We eliminated all these alternative explanations one by one in the manuscript, as follows:

a) With regard to the potential impact of precipitation amount, following the comments provided by Reviewers #3 and #4, we added a figure to show the spatial pattern of precipitation amount along the east–west transect (Please see Figure R1 or Fig. S15 in the revised Supplementary Information). The result showed that:

i) If precipitation amount affects the V-shaped pattern of $\delta^{18}O_p$ along the east–west transect, precipitation amount along the transect should appear as an "inverted V-shaped" pattern during the monsoon season. However, our precipitation amount data do not show an "inverted V-shaped" pattern, which can not explain the V-shaped pattern of $\delta^{18}O_p$.

ii) More important, a notable discrepancy was observed at the Sylhet site, where monthly precipitation amount is exceptionally high, yet the $\delta^{18}O_p$ at this site did not exhibit the correspondingly expected low values. Instead, the $\delta^{18}O_p$ at this site remained relatively high. This result conflicted with the theory of precipitation "amount effect." Therefore, it can be seen that the spatial pattern of precipitation amount is inconsistent with that of the $\delta^{18}O_p$ along the transect.

Thus, we can conclude that the $\delta^{18}O_p$ along the transect shows little correlation with the precipitation amount. We added a figure to show the spatial pattern of precipitation amount along the east–west transect (Please see Figure R1 or Fig. S15 in the revised Supplementary Information), and we added a paragraph in the manuscript to eliminate the influence of precipitation amount on $\delta^{18}O_p$ along the transect. i.e., "*It could also be proposed that terrain-enhanced updrafts might increase precipitation, linking* $\delta^{18}O_p$ *to precipitation amount. If true, monsoon-season precipitation should show an inverted V-shaped pattern along the transect. However, our results do not support this hypothesis as precipitation amount in the middle is relatively low while it is considerably higher at Sylhet in the western domain (Supplementary Fig. 15).*" Please see L320–L325 in the revised manuscript. Please see Fig. S15 in the revised Supplementary Information.

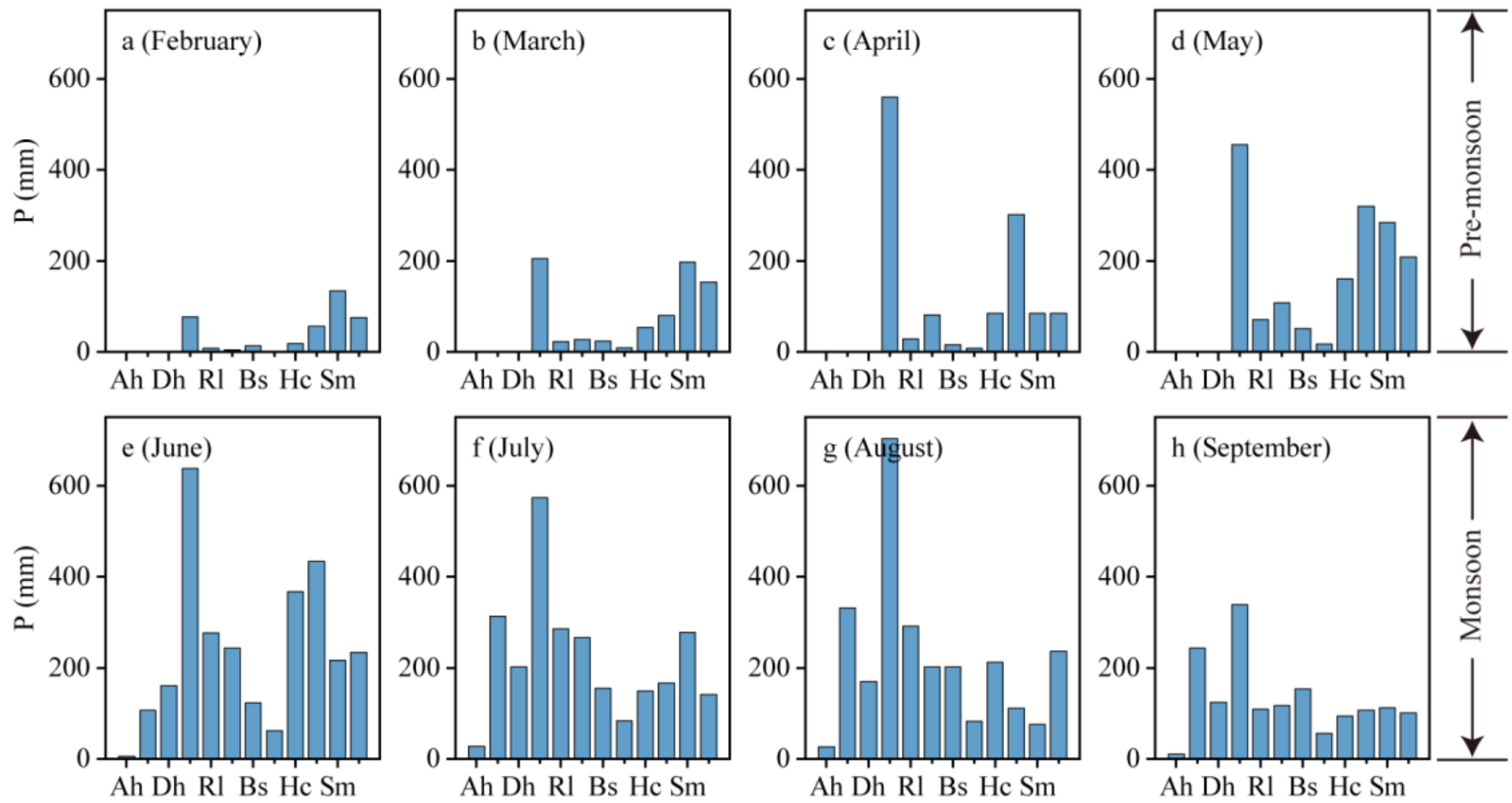

Figure R1. Spatial distributions of precipitation amount at the 12 primary sites along the transect during the pre-monsoon and monsoon seasons. a–d, Pre-monsoon season. e–h, Monsoon season. Abbreviations: Ah (Ahmedabad), Dh (Dhanbad), Rl (Ruili), Bs (Baoshan), Hc (Hechi), and Sm (Sanming).

b) With regard to the potential impact of the monsoon, we eliminated the influence of the monsoon in two sections of the revised manuscript:

i) In the Introduction section, we raised doubts about the influence of monsoon

moisture on $\delta^{18}O_p$ with a paragraph, based on the evidence from measured data. i.e., "Many studies have also argued that $\delta^{18}O_p$ in the Asian monsoon region reflects moisture sources[44-47], particularly South Asian monsoon-related moisture transport[48-50]. If this were correct, $\delta^{18}O_s$ records from upstream regions along the South Asian monsoon moisture pathway should show higher $\delta^{18}O$ values than those from downstream locations. However, available evidence conflicts with this assumption. For example, the $\delta^{18}O_s$ record from Tham Doun Mai cave (Luang Prabang Province, Northern Laos) has a mean value of -9.84‰[51], while Dongge cave (Guizhou Province, southwestern China), located more than 4° to the north, has a higher mean value of -8.92‰[52] over the period 8874–7581 yr BP. Similarly, annual $\delta^{18}O_p$ values from Son La site near Tham Doun Mai cave (2017–2020) show a mean of -7.15‰ (Global Network of Isotopes in Precipitation, GNIP), which is lower than Huanjiang (-6.58‰)[53] near Dongge cave. These contradictions highlight ongoing uncertainties in identifying the key controls on $\delta^{18}O_p$ variations across the Asian monsoon region." Please see L95–L108 in the revised manuscript.

ii) In the Discussion section, we have a complete section entitled "$\delta^{18}O_p$ and the advection of monsoon moisture" to eliminate the influence of monsoon. Please see L164–L223 in the revised manuscript.

c) With regard to the potential impact of altitude, we added two paragraphs to eliminate altitude as a key influence on $\delta^{18}O_p$, i.e., "*One might argue that terrain could influence $\delta^{18}O_p$, given numerous high mountains in the middle of the transect. To test for an "altitude effect" where $\delta^{18}O_p$ decrease with increasing altitude due to lower condensation temperature[69], we analyzed $\delta^{18}O_p$ versus altitude monthly (Fig. 3c). Correlations change over time from weak positive, weak negative, and then to strong negative (Fig. 3c), reflecting the westward migration of low $\delta^{18}O_p$ from pre-monsoon to monsoon, aligning with high-elevation areas and producing a "false" altitude effect (Supplementary Fig. 12). Classification of sites by Köppen-Geiger climate zone[70] (Supplementary Fig. 13) or UNEP-WCMC altitude ranges[71] (Supplementary Fig. 14) shows inconsistent or absent negative $\delta^{18}O_p$–altitude correlations. Previous studies also reported "inverse altitude effects" in this region[72-74]. For example, $\delta^{18}O_p$ on the Ainao Mountains (23.22° N, 102.84° E) increases with altitude[72,73], due to complex moisture sources, and $\delta^{18}O_p$ on the*

leeward side of the Meili Snow Mountains (28.46° N, 98.77° E) shows similar pattern[74]*, due to below-cloud evaporation and recycled moisture mixing. These results indicate altitude does not significantly control* $\delta^{18}O_p$ *distribution. More important, the gradually changing correlation between* $\delta^{18}O_p$ *and altitude further support our conclusion that the low* $\delta^{18}O_p$ *values gradually migrate westward along the transect over time.*" Please see L302–L319 in the revised manuscript.

Correspondingly, we added one figure in the revised manuscript (Fig. 3c), modified one figure in the revised Supplementary Information (Fig. S12), added two figures in the revised Supplementary Information (Figs. S13 and S14) to discuss the relationship between $\delta^{18}O_p$ and altitude. Fig. 3c, Fig. S12, and Figs. S13 and S14 can be referred to Figures R2, R3, R4, and R5 as follows.

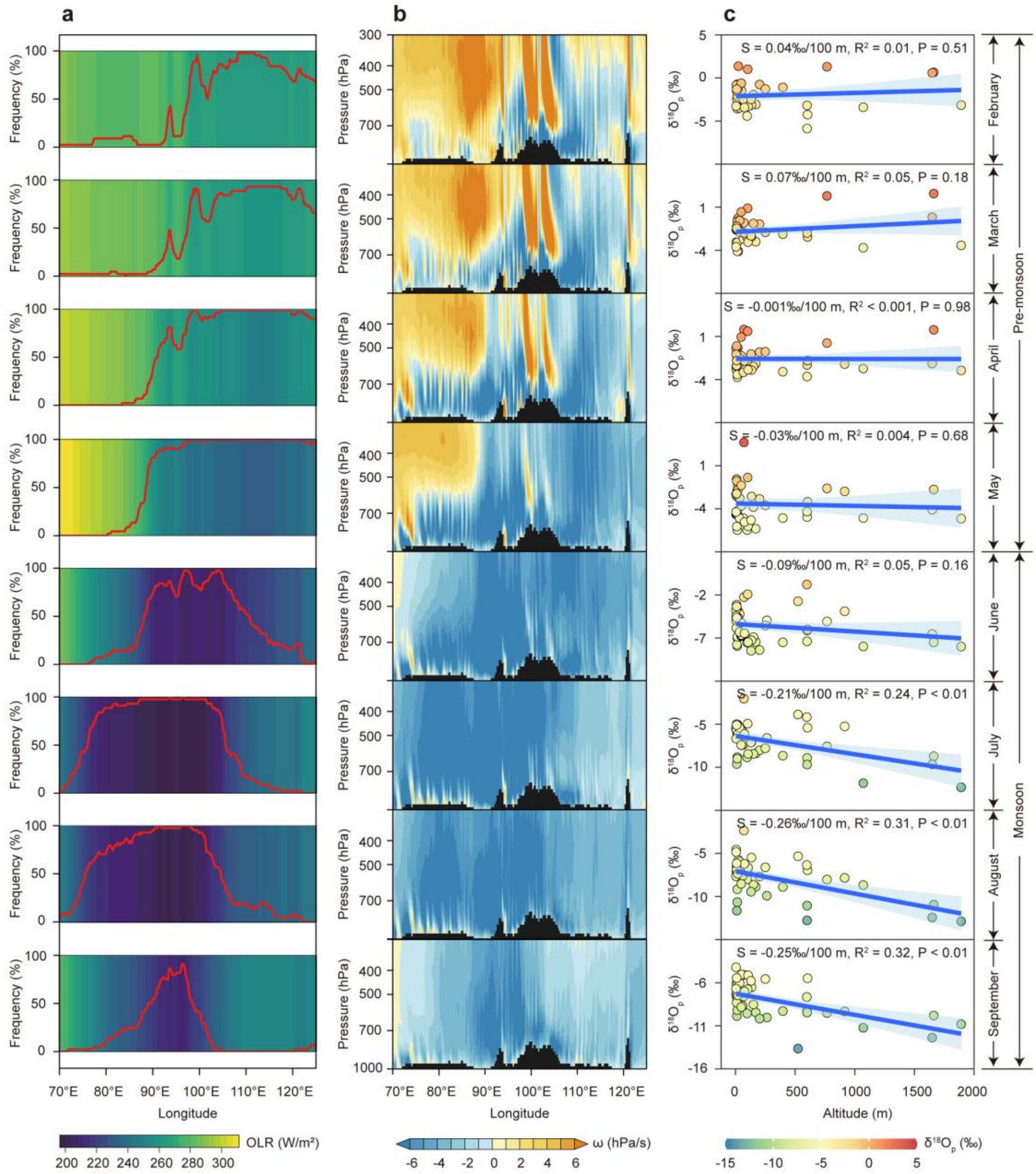

Figure R2. Seasonal changes of atmospheric updraft and altitude effect on $\delta^{18}O_p$ during the pre-monsoon and monsoon seasons over 1979–2022. a Seasonal variations in outgoing longwave radiation (OLR, shading) and the frequencies of relatively low OLR values (red line) along the transect. Lower OLR values indicate stronger convection. b Vertical profiles of meridional mean vertical velocity (ω), averaged over 20–27° N. Negative ω values indicate upward motion (convection), while positive values indicate downward motion (subsidence). c Linear regressions of $\delta^{18}O_p$ at the 46 sites across the study region against altitude, fitted for each month. $\delta^{18}O_p$ values are expressed as per mil (‰) deviations relative to VSMOW or VSMOW2.

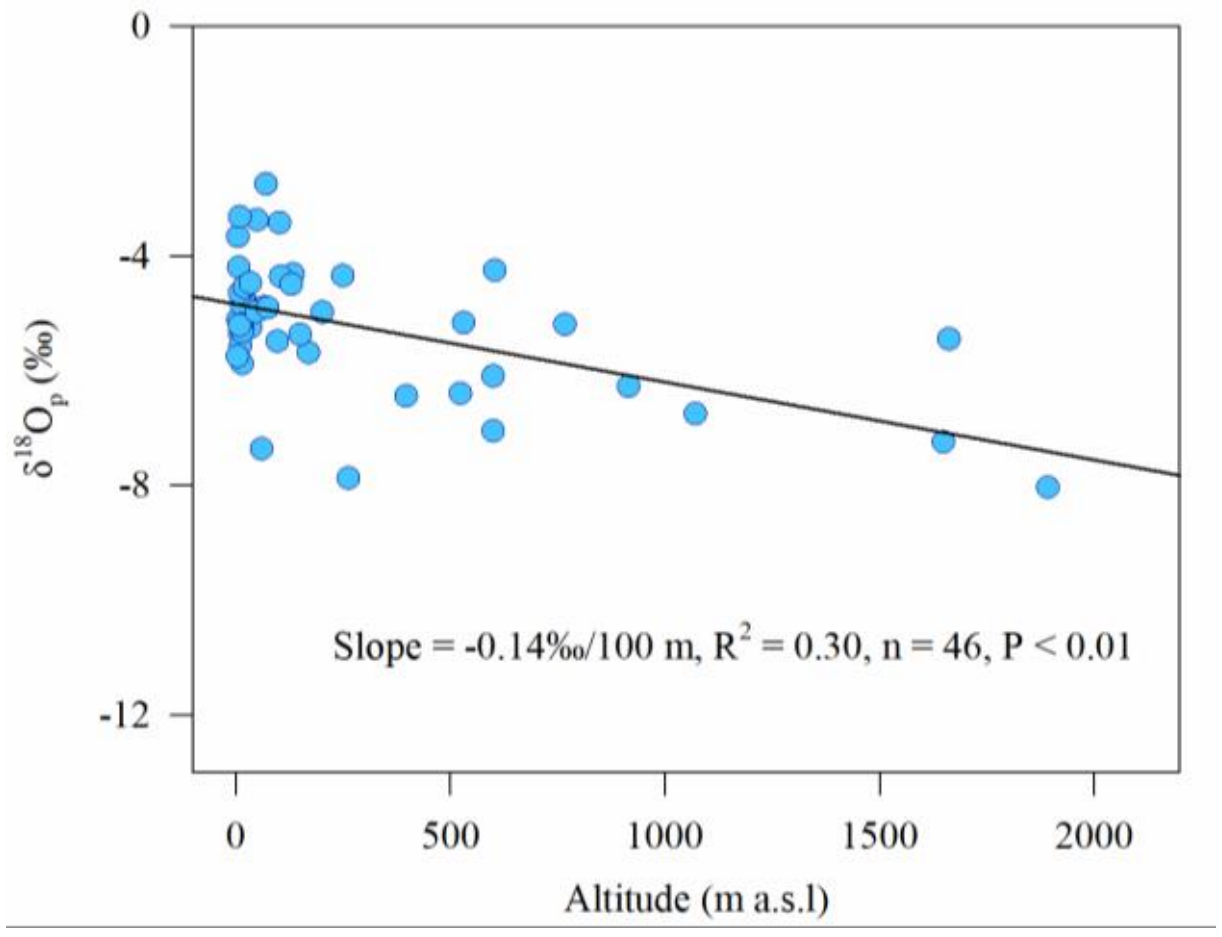

Figure R3. Influence of altitude on observed $\delta^{18}O_P$ at the 46 sites across the study region from 1979 to 2022. $\delta^{18}O_P$ values are expressed as per mil (‰) deviations relative to VSMOW or VSMOW2.

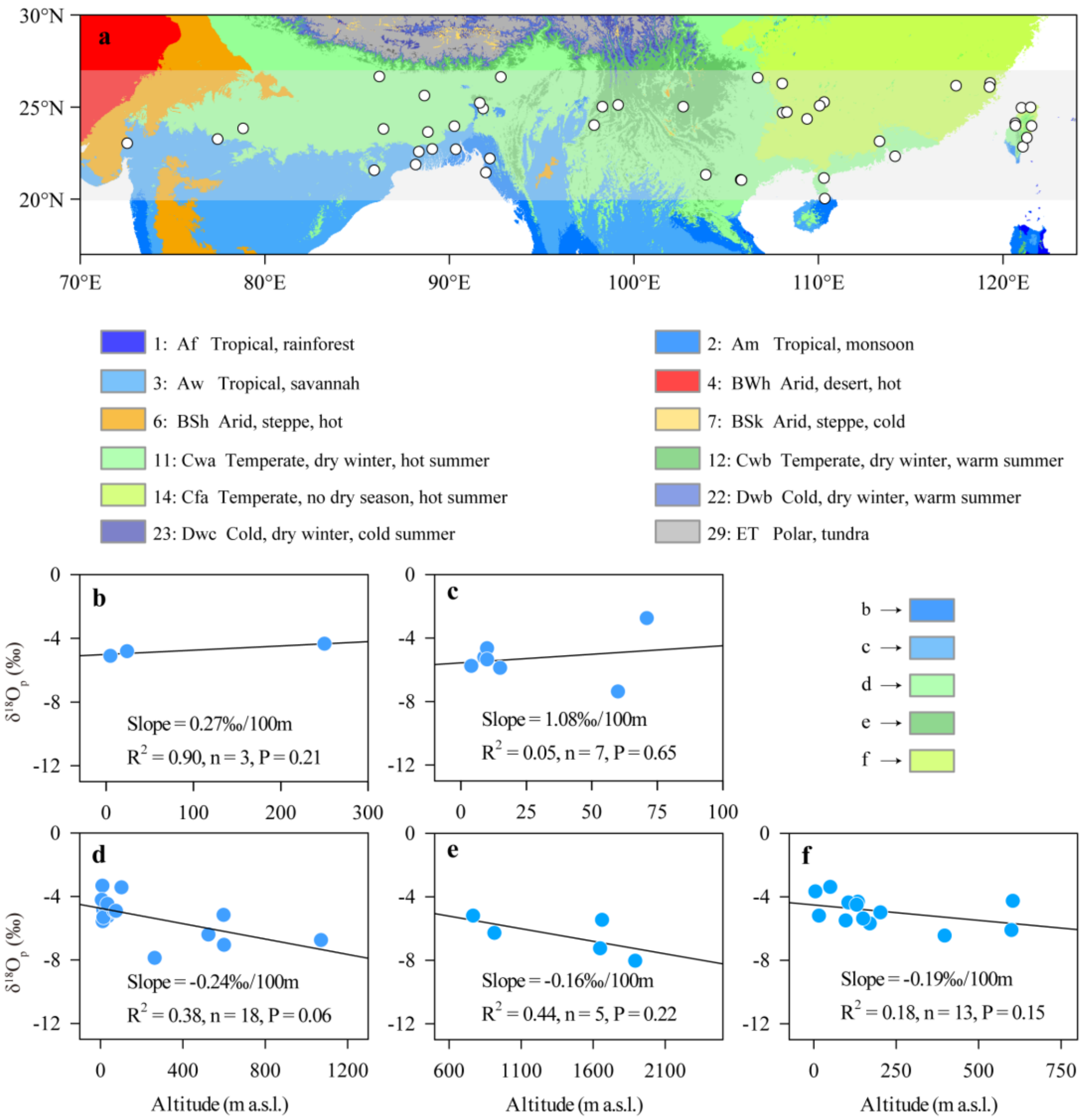

Figure R4. Correlations between observed $\delta^{18}O_p$ values and altitudes across five climate zones. a, Climate zones according to the Köppen-Geiger climate classification[1]. Correlations for the climate zones: Tropical, monsoon (Am) (b); Tropical, savannah (Aw) (c); Temperate, dry winter, hot summer (Cwa) (d); Temperate, dry winter, warm summer (Cwb) (e); and Temperate, no dry season, hot summer (Cfa) (f). $\delta^{18}O_p$ values are expressed as per mil (‰) deviations relative to VSMOW or VSMOW2.

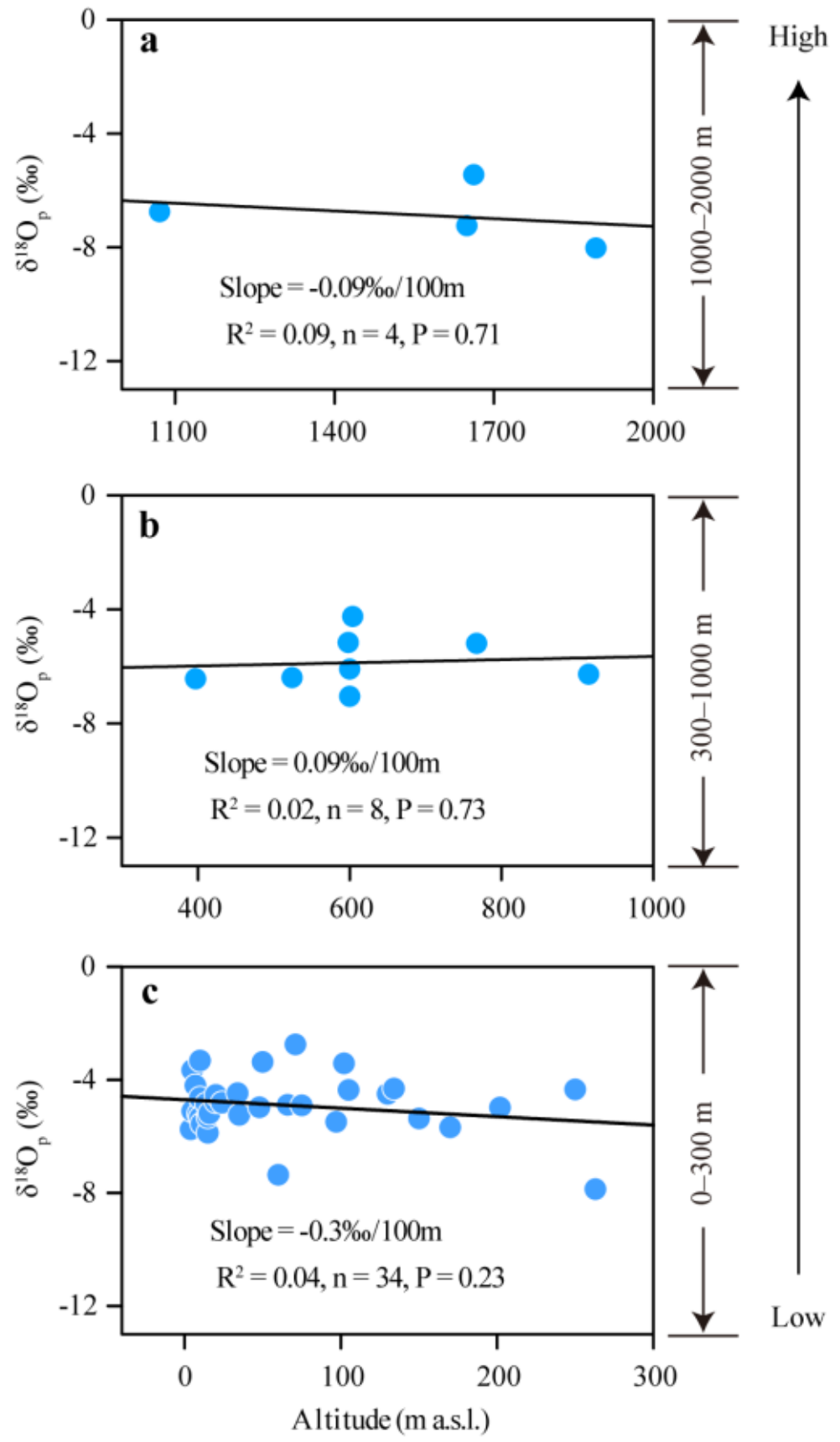

Figure R5. Correlations between observed $\delta^{18}O_p$ values and altitudes. The 46 precipitation sites were categorized into three altitude ranges based on the UNEP-WCMC mountain classification[2]. a–c, Correlations for the altitude ranges 1000–2000 m (a), 300–1000 m (b), and 0–300 m (c). $\delta^{18}O_p$ values are expressed as per mil (‰) deviations relative to VSMOW or VSMOW2.

References:

70. Beck, H. E. et al. High-resolution (1 km) Köppen-Geiger maps for 1901–2099 based on constrained CMIP6 projections. *Sci. Data* **10**, 724 (2023).

71. UNEP World Conservation Monitoring Centre. *Mountain Watch: Environmental Change and Sustainable Development in Mountains.* Technical Report. Cambridge, UK: UNEP-WCMC, 2002.

4) With regard to clearly linking observations and results to the study's main objective and novelty—to assess how atmospheric updraft influences speleothem $\delta^{18}O$ signals across the Asian monsoon region, relative to established factors like rainout and amount effects, indeed, our main objective is to investigate the influence of atmospheric updrafts on $\delta^{18}O_s$ signals in the Asian monsoon region. As we know, $\delta^{18}O_s$ records reflect long-term climate changes. To clarify the climate signals preserved in Asian $\delta^{18}O_s$ records, we analyzed modern $\delta^{18}O_p$ variability. This analysis is feasible because $\delta^{18}O_s$ records can reconstruct past climate conditions under the assumption that $\delta^{18}O_s$ inherits the $\delta^{18}O_p$ signals from outside the cave (Yu et al., 2021). Therefore, by examining how atmospheric updraft affects $\delta^{18}O_p$ in the Asian monsoon region, we can determine the impact of atmospheric updraft on $\delta^{18}O_s$ records.

As mentioned above, based on $\delta^{18}O_p$ analysis, we systematically evaluated alternative explanations, including precipitation amount, rainout effect (occurring during the advection of monsoon moisture), and altitude, and eliminated their influences. That is, we compared these with established factors (rainout effect occurring during the advection of monsoon moisture and precipitation "amount effect") and found that these established factors cannot explain the spatial pattern of $\delta^{18}O_p$ along the transect, nor can they explain the migration of the low $\delta^{18}O_p$ values from east to west. Instead, atmospheric updraft can well explain the spatial pattern of $\delta^{18}O_p$ along the transect and also the migration of the low $\delta^{18}O_p$ values from east to west. Moreover, regardless of the monthly scale or the annual scale, the correlation between $\delta^{18}O_p$ and the indicators of atmospheric updraft (OLR) is more significant. Therefore, compared with other established factors, the role of atmospheric updraft is more crucial.

With regard to the precipitation "amount effect", following the comments provided by Reviewers #3 and #4, we added a figure to show the spatial pattern of precipitation amount along the east–west transect (Please see Figure R1 or Fig. S15 in the revised Supplementary Information). In addition, we added a paragraph in the manuscript to eliminate the influence of precipitation amount on $\delta^{18}O_p$ along the transect. Please see

L320–L325 in the revised manuscript. Please see Fig. S15 in the revised Supplementary Information.

With regard to rainout effect occurring during the advection of monsoon moisture, as mentioned above, we eliminated the influence of monsoon in two sections of the revised manuscript. Please see L95–L108 and L164–L223 in the revised manuscript.

Through our revisions, the manuscript's structure and content have been substantially improved. We hope these improvements will be to your satisfaction.

Reference:

Yu, W. et al. Temperature signals of ice core and speleothem isotopic records from Asian monsoon region as indicated by precipitation $\delta^{18}O$. *Earth Planet. Sci. Lett.* **554**, 116665 (2021).

2. The lack of transparency regarding how datasets were compiled for use in this study. I found it surprising that, for a piece of research that rests so heavily on analysis of pre-existing datasets, very little information was given on the manner in which these records were identified, screened, and evaluated. The study makes no secret of the fact that there is huge diversity in the expressions of speleothem $\delta^{18}O$ signals across this domain, and so there is even greater need for a clear process of determining whether (or not) records were suitable for this work. Hence, we would like to see evidence for a clear, strategic procedure for data selection, and a means of quality-checking the datasets utilized in this study, which could be done by means of intra-study comparisons, data triangulation (e.g., cross-checking isotope measurements across different speleothems within the same system), and/or comparison of interpretations made from the same system.

Re: We thank you for pointing these issues out. We admit the lack of transparency in our dataset compilation method. Following your comments and suggestions, we provided additional explanations in the following aspects to enhance the transparency of the compiled dataset.

1) With regard to the transparency of the $\delta^{18}O_p$ data, we systematically collected all available $\delta^{18}O_p$ data within the study region (70–125° E, 20–27° N). The observed

$\delta^{18}O_p$ data from three sources: including data from the GNIP dataset (https://iaea.org/services/networks/gnip), published studies, and two unpublished sites (Hechi and Baoshan). We then compiled $\delta^{18}O_p$ observations along the east–west transect using the following selection criteria:

a) Data period selection (1979–2022):

This study focused on the 1979–2022 to ensure temporal consistency between observational and model datasets. The decision was made based on two considerations: i) pre-1979 observed data in the study area were sparse, comprising just four observation sites (Karachi, Mumbai, Shillong, and Luang Prabang; see Table R1); moreover, these four sites have no data after 1979; and ii) the IsoGSM2 simulated $\delta^{18}O_p$ data begin in 1979. To ensure temporal consistency between observation and model datasets, we only included sites with data spanning 1979-2022. Consequently, the aforementioned four sites were eliminated from our analysis. Notably, although Hong Kong's observations date back to 1961, we limited the analysis to the period 1979–2022 for consistency with other sites.

Table R1. Summary data of the 4 precipitation sampling sites eliminated due to lack of post-1979 data.

No.	Site	Latitude (°N)	Longitude (°E)	Altitude (m a.s.l.)	Period	Number of samples	Time scale	Reference
1	Karachi	24.9	67.13	23	1961/7-1975/8	45	Monthly	GNIP
2	Mumbai	18.9	72.82	10	1961/6-1977/9	50	Monthly	GNIP
3	Shillong	25.57	91.88	1598	1969/1-1978/10	31	Monthly	GNIP
4	Luang-Prabang	19.88	102.13	305	1961/3-1964/11	26	Monthly	GNIP

b) Sample size criteria for $\delta^{18}O_p$ data:

To ensure statistical significance, we compiled observed $\delta^{18}O_p$ data along the east–west transect using the following selection criteria: the sample size must be at least 50 ($n \geq 50$) for daily sampling sites and at least 30 ($n \geq 30$) for monthly sampling sites. Based on these criteria, we further eliminated seven sites that did not meet the criteria (Alishan, Keelung, Puli, Chiayi, Baojinggong, Wuling, Bilu; Table R2). In this case, a total of 46 sites met these criteria (Figure R3). Among these 46 sites, seven of them have both daily and monthly $\delta^{18}O_p$ data (Figure R4). Therefore, when counting the number of sites, these seven sites will not be counted repeatedly.

Table R2. Summary data of the 7 precipitation sampling sites eliminated based on sample size criteria.

No.	Site	Latitude (° N)	Longitude (° E)	Altitude (m a.s.l.)	Period	Number of samples	Time scale	Reference
1	Baojinggong	24.11	113.35	610	2011/6-2014/1	26	Monthly	Duan et al., 2016
2	Chiayi	23.48	120.46	27	2002/5-2005/9	25	Monthly	Peng et al., 2010
3	Alishan	23.51	120.81	2413	2003/5-2006/11	24	Monthly	Peng et al., 2010
4	Puli	23.97	120.98	732	1999/4-2002/1	24	Monthly	Peng et al., 2010
5	Wuling	24.35	121.31	1800	2005/12-2008/1	26	Monthly	Peng et al., 2010
6	Bilu	24.23	121.32	2350	2005/6-2008/1	28	Monthly	Peng et al., 2010
7	Keelung	25.13	121.71	27	2004/6-2006/5	24	Monthly	Peng et al., 2010

Table R3. Summary data for the 46 precipitation sampling sites in the region of 20-27°N, 70-125°E. The sampling sites are listed by longitude from east to west.

No.	Site	Latitude (°N)	Longitude (°E)	Elevation (m a.s.l.)	Period	Number of samples	Time scale	Reference
1	Ahmedabad	23.03	72.55	71	2005/7–2016/9	329	Daily	Oza et al., 2020
2	Bhopal	23.25	77.45	524	2013/6–2013/9	58	Daily	Midhun et al., 2018
3	Sagar	23.83	78.83	531	2016/6–2021/10	291	Daily	Chakraborty et al., 2025
4	RGS-1	21.56	85.94	60	2016/7–2016/9	52	Daily	Ansari et al., 2020
5	Siraha	26.65	86.22	102	2014/5–2017/10	222	Daily	Acharya et al., 2020
6	Dhanbad	23.81	86.44	263	2013/6–2013/9	61	Daily	Midhun et al., 2018
7	Kakdweep	21.87	88.18	4	2015/4–2016/11	53	Daily	Chakraborty et al., 2022
8	Kolkata	22.57	88.36	9	2004/6–2018/10	206	Daily/Weekly	Chakraborty et al., 2022; Sengupta et al., 2006
9	Dinajpur	25.62	88.66	35	2014/2–2021/10	62	Monthly	GNIP
10	Chuadanga	23.64	88.85	20	2014/5–2021/11	62	Monthly	GNIP
11	Satkhira	22.72	89.08	10	2015/4–2021/9	162	Daily/Monthly	Islam et al., 2021; GNIP
12	Dhaka	23.95	90.28	14	2009/3–2021/12	119	Monthly	GNIP
13	Barisal	22.7	90.36	10	2013/5–2021/10	436	Daily/Monthly	Munksgaard et al., 2019; GNIP
14	Mawlong	25.22	91.66	915	2007/4–2008/10	68	Daily	Breitenbach et al., 2010; GNIP
15	Sylhet	24.91	91.85	20	2009/5–2021/9	363	Daily/Monthly	Islam et al., 2021; GNIP
16	Cox's Bazar	21.44	91.99	5	2014/5–2021/10	154	Daily/Monthly	Munksgaard et al., 2019; GNIP
17	Bandarban	22.2	92.2	24	2015/3–2021/11	48	Monthly	GNIP
18	Tezpur	26.63	92.8	48	2015/4–2018/9	235	Daily	Chakraborty et al., 2022
19	Ruili	24.01	97.85	767	2018/1–2021/12	450	Daily	Guo et al., 2024
20	Tengchong	25.01	98.3	1649	2009/1–2011/12	343	Daily	Wu et al., 2022
21	Baoshan	25.11	99.16	1662	2017/8–2019/8	215	Daily	This study
22	Kunming	25.02	102.68	1892	1986/4–2011/12	284	Daily/Monthly	Li et al., 2017; GNIP
23	Son La	21.32	103.91	600	2017/4–2021/11	46	Monthly	GNIP
24	Hanoi	21.05	105.8	11	2004/9–2007/12	38	Monthly	GNIP
25	Hanoi (Igs)	21.03	105.84	15	2015/1–2021/11	80	Monthly	GNIP
26	Guiyang	26.58	106.72	1071	1988/2–1992/12	55	Monthly	GNIP
27	Liangfeng	26.27	108.05	600	2011/5–2014/4	36	Monthly	Duan et al., 2016
28	Hechi	24.69	108.08	202	2015/1–2017/12	303	Daily	This study
29	Huanjiang	24.72	108.3	397	2011/1–2020/12	94	Monthly	Zhang et al., 2023
30	Liuzhou	24.35	109.4	97	1988/1–1992/8	53	Monthly	GNIP
31	Guilin	25.07	110.08	170	1983/1–1990/12	92	Monthly	GNIP
32	Zhanjiang	21.14	110.31	10	2015/5–2020/4	412	Daily	Chen et al., 2021
33	Institute of Karst Geology	25.26	110.32	150	2010/1–2010/12;<br>2012/1–2012/12;<br>2015/1–2016/12	450	Daily	Wu et al., 2013;<br>Wu et al., 2014;<br>Wu et al., 2020
34	Haikou	20.03	110.35	15	1988/3–2000/7	59	Monthly	GNIP
35	Guangzhou	23.13	113.32	7	1986/4–2014/12	1046	Daily/Monthly	Yang et al., 2017; GNIP
36	Hong Kong	22.32	114.17	66	1979/1–2022/12	496	Monthly	GNIP
37	Sanming	26.15	117.47	134	2019/1–2020/12	209	Daily	Cai et al., 2022
38	Fuzhou	26.08	119.28	16	1985/10–1992/12	71	Monthly	GNIP
39	Luoyuan	26.3	119.32	604	2018/1–2021/12	529	Daily	Guo et al., 2024
40	Taichung	24.12	120.68	34	2001/6–2007/10	67	Monthly	Peng et al., 2010
41	Tsaotun	23.99	120.69	75	2001/7–2006/11	54	Monthly	Peng et al., 2010
42	Taoyuan	24.95	121.02	105	2004/1–2007/4	35	Monthly	Peng et al., 2010
43	Taidung	22.84	121.09	250	2004/5–2007/11	38	Monthly	Peng et al., 2010
44	Yuli	23.33	121.31	130	2005/1–2007/8	31	Monthly	Peng et al., 2010
45	Taipei	24.98	121.51	5	2004/3–2006/12	34	Monthly	Peng et al., 2010
46	Hualien	23.97	121.56	50	2003/7–2006/5	35	Monthly	Peng et al., 2010

Table R4. Summary data of the 7 precipitation sampling sites with both daily and monthly data.

No.	Site	Latitude (°N)	Longitude (°E)	Altitude (m a.s.l.)	Period	Number of samples	Time scale	Reference
1	Kolkata	22.57	88.36	9.14	2015/4-2018/10	170	Daily	Chakraborty et al., 2022
2	Kolkata	22.75	88.5	9.14	2004/6-2005/10	36	half Monthly	Sengupta and Sarkar, 2006
3	Satkhira	22.74	89.07	10	2017/3-2018/7	114	Daily	Islam et al., 2021
4	Satkhira	22.72	89.08	10	2015/4-2021/9	48	Monthly	GNIP
5	Barisal	22.72	90.35	7	2013/5-2018/7	364	Daily	Munksgaard et al., 2019
6	Barisal	22.7	90.36	10	2013/5-2021/10	72	Monthly	GNIP
7	Sylhet	25	91.76	21	2017/2-2018/9	259	Daily	Islam et al., 2021
8	Sylhet	24.91	91.85	20	2009/5-2021/9	104	Monthly	GNIP
9	Cox's Bazar	21.44	91.97	8	2015/3-2015/12	104	Daily	Munksgaard et al., 2019
10	Cox's Bazar	21.44	91.99	5	2014/5-2021/10	50	Monthly	GNIP
11	Kunming	25.01	102.4	1892	2009/1-2011/12	132	Daily	Li et al., 2017
12	Kunming	25.02	102.68	1892	1986/4-2003/12	152	Monthly	GNIP
13	Guangzhou	23.13	113.32	7	1986/4-1989/9	30	Monthly	GNIP
14	Guangzhou	23.13	113.32	7	2007/1-2014/12	1016	Daily	Yang et al., 2017

c) Classification criteria for primary and ancillary sites:

To further reduce possible latitude effects on $\delta^{18}O_p$, we identified 12 primary sites (Table R5) within the 46 site, based on the following criteria:

i) observation sites located within a narrow latitudinal band of 1°±1.5°;

ii) availability of high-precision (daily) $\delta^{18}O_p$ observations;

iii) maximum possible observation duration.

The remaining 34 sites served as ancillary sites to validate the spatial patterns of the primary sites (Table R6). Please see L687–L692 in the revised manuscript.

Table R5. Summary data for the 12 primary precipitation sampling sites in the region of 20–27° N, 70–125° E. The sampling sites are listed by longitude from west to east.

No.	Site	Latitude (° N)	Longitude (° E)	Elevation (m a.s.l.)	Period	Number of samples	Time scale	Reference
1	Luoyuan	26.3	119.32	604	2018/1–2021/12	529	Daily	Guo et al., 2024
2	Sanming	26.15	117.47	134	2019/1–2020/12	209	Daily	Cai et al., 2022
3	Institute of Karst Geology	25.26	110.32	150	2010/1–2010/12; 2012/1–2012/12; 2015/1–2016/12	450	Daily	Wu et al., 2013; Wu et al., 2014; Wu et al., 2020
4	Hechi	24.69	108.08	202	2015/1–2017/12	303	Daily	This study
5	Kunming	25.02	102.68	1892	1986/4–2011/12	284	Daily/Monthly	Li et al., 2017; GNIP
6	Baoshan	25.11	99.16	1662	2017/8–2019/8	215	Daily	This study
7	Tengchong	25.01	98.3	1649	2009/1–2011/12	343	Daily	Wu et al., 2022
8	Ruili	24.01	97.85	767	2018/1–2021/12	450	Daily	Guo et al., 2024
9	Sylhet	24.91	91.85	20	2009/5–2021/9	363	Daily/Monthly	Islam et al., 2021; GNIP
10	Dhanbad	23.81	86.44	263	2013/6–2013/9	61	Daily	Midhun et al., 2018
11	Sagar	23.82	78.76	598	2016/6–2021/10	291	Daily	Chakraborty et al., 2025
12	Ahmedabad	23.03	72.55	71	2005/7–2016/9	329	Daily	Oza et al., 2020

Table R6. Summary data for the 34 ancillary precipitation sampling sites in the region of 20–27° N, 70–125° E. The sampling sites are listed by longitude from west to east.

No.	Site	Latitude (°N)	Longitude (°E)	Elevation (m a.s.l.)	Period	Number of samples	Time scale	Reference
1	Bhopal	23.25	77.45	524	2013/6–2013/9	58	Daily	Midhun et al., 2018
2	RGS-1	21.56	85.94	60	2016/7–2016/9	52	Daily	Ansari et al., 2020
3	Siraha	26.65	86.22	102	2014/5–2017/10	222	Daily	Acharya et al., 2020
4	Kakdweep	21.87	88.18	4	2015/4–2016/11	53	Daily	Chakraborty et al., 2022
5	Kolkata	22.57	88.36	9	2004/6–2018/10	206	Daily/Weekly	Chakraborty et al., 2022; Sengupta et al., 2006
6	Dinajpur	25.62	88.66	35	2014/2–2021/10	62	Monthly	GNIP
7	Chuadanga	23.64	88.85	20	2014/5–2021/11	62	Monthly	GNIP
8	Satkhira	22.72	89.08	10	2015/4–2021/9	162	Daily/Monthly	Islam et al., 2021; GNIP
9	Dhaka	23.95	90.28	14	2009/3–2021/12	119	Monthly	GNIP
10	Barisal	22.7	90.36	10	2013/5–2021/10	436	Daily/Monthly	Munksgaard et al., 2019; GNIP
11	Mawlong	25.22	91.66	915	2007/4–2008/10	68	Daily	Breitenbach et al., 2010; GNIP
12	Cox's Bazar	21.44	91.99	5	2014/5–2021/10	154	Daily/Monthly	Munksgaard et al., 2019; GNIP
13	Bandarban	22.2	92.2	24	2015/3–2021/11	48	Monthly	GNIP
14	Tezpur	26.63	92.8	48	2015/4–2018/9	235	Daily	Chakraborty et al., 2022
15	Son La	21.32	103.91	600	2017/4–2021/11	46	Monthly	GNIP
16	Hanoi	21.05	105.8	11	2004/9–2007/12	38	Monthly	GNIP
17	Hanoi (Igs)	21.03	105.84	15	2015/1–2021/11	80	Monthly	GNIP
18	Guiyang	26.58	106.72	1071	1988/2–1992/12	55	Monthly	GNIP
19	Liangfeng	26.27	108.05	600	2011/5–2014/4	36	Monthly	Duan et al., 2016
20	Huanjiang	24.72	108.3	397	2011/1–2020/12	94	Monthly	Zhang et al., 2023
21	Liuzhou	24.35	109.4	97	1988/1–1992/8	53	Monthly	GNIP
22	Guilin	25.07	110.08	170	1983/1–1990/12	92	Monthly	GNIP
23	Zhanjiang	21.14	110.31	10	2015/5–2020/4	412	Daily	Chen et al., 2021
24	Haikou	20.03	110.35	15	1988/3–2000/7	59	Monthly	GNIP
25	Guangzhou	23.13	113.32	7	1986/4–2014/12	1046	Daily/Monthly	Yang et al., 2017; GNIP
26	Hong Kong	22.32	114.17	66	1979/1–2022/12	496	Monthly	GNIP
27	Fuzhou	26.08	119.28	16	1985/10–1992/12	71	Monthly	GNIP
28	Taichung	24.12	120.68	34	2001/6–2007/10	67	Monthly	Peng et al., 2010
29	Tsaotun	23.99	120.69	75	2001/7–2006/11	54	Monthly	Peng et al., 2010
30	Taoyuan	24.95	121.02	105	2004/1–2007/4	35	Monthly	Peng et al., 2010
31	Taidung	22.84	121.09	250	2004/5–2007/11	38	Monthly	Peng et al., 2010
32	Yuli	23.33	121.31	130	2005/1–2007/8	31	Monthly	Peng et al., 2010
33	Taipei	24.98	121.51	5	2004/3–2006/12	34	Monthly	Peng et al., 2010
34	Hualien	23.97	121.56	50	2003/7–2006/5	35	Monthly	Peng et al., 2010

Following your comments and suggestions, we changed "We compiled $\delta^{18}O_p$ observations along the east–west transect using the following selection criteria: the dataset must cover the period 1979–2022, with a sample size of at least 50 ($n \geq 50$) for daily sampling sites and at least 30 ($n \geq 30$) for monthly sampling sites. A total of 46 sites met these criteria, including data from the GNIP dataset (https://iaea.org/services/networks/gnip), published studies, and two unpublished sites (Hechi and Baoshan) (Supplementary Table 1). Details on sample collection, storage, and measurement are available in ref. 42. We also compiled $\delta^{18}O_s$ data from 13 caves based on published studies (Supplementary Table 2)." to

"***Selection criteria for observed $\delta^{18}O_p$ data***

We retrieved the observed $\delta^{18}O_p$ data from three sources: including data from the GNIP dataset (https://iaea.org/services/networks/gnip), published studies, and two unpublished sites (Hechi and Baoshan). Details on sample collection, storage, and measurement are available in ref. 38. We compiled $\delta^{18}O_p$ observations along the east–west transect using the following selection criteria: (1) the dataset must cover the period 1979–2022; and (2) the sample size must be at least 50 ($n \geq 50$) for daily sampling sites and at least 30 ($n \geq 30$) for monthly sampling sites. Notably, although

Hong Kong's observations date back to 1961, we limited the analysis to the period 1979–2022 for consistency with other sites. A total of 46 sites met these criteria (Supplementary Tables 1 and 2). Among these 46 sites, seven have both daily and monthly $\delta^{18}O_p$ data. Therefore, when counting the number of sites, these seven sites will not be counted repeatedly.

To further reduce possible latitude effects on $\delta^{18}O_p$, we identified 12 primary sites (Supplementary Table 1) within the 46 sites, based on the following criteria: (1) observation sites located within a narrow latitudinal band of 1°±1.5°; (2) availability of high-precision (daily) $\delta^{18}O_p$ observations; and (3) maximum possible observation duration. The remaining 34 sites served as ancillary sites to validate the spatial patterns of the primary sites (Supplementary Table 2). " Please see L675–L692 in the revised manuscript.

References

Acharya, S. Yang, X., Yao, T. & Shrestha, D. Stable isotopes of precipitation in Nepal Himalaya highlight the topographic influence on moisture transport. *Quatern. Int.* **565**, 22–30 (2020).

Ansari, M. A., Noble, J., Deodhar, A., & Kumar, U. S. Atmospheric factors controlling the stable isotopes ($\delta^{18}O$ and $\delta^{2}H$) of the Indian summer monsoon precipitation in a drying region of Eastern India. *J. Hydrol.* **584**, 124636 (2020).

Breitenbach, S. F. M. et al. Strong influence of water vapor source dynamics on stable isotopes in precipitation observed in Southern Meghalaya, NE India. *Earth Planet. Sci. Lett.* **292**, 212–220 (2010).

Cai, J., Yang, Y., Yang, Z., Qiu, W. & Jiang, X. Isotope composition of daily precipitation from 2019 to 2020 in Sanming, southeastern China. *Front. Environ. Sci.* **10**, 1061882 (2022).

Chakraborty, S. et al. Linkage between precipitation isotopes and biosphere-atmosphere interaction observed in northeast India. *npj Clim. Atmos. Sci.* **5**, 10 (2022).

Chakraborty, S. et al. Precipitation isotopes and monsoon dynamics in the core monsoon zone of India. *Sci. Rep.* **15**, 6761 (2025).

Chen, F. et al. Typhoon control of precipitation dual isotopes in southern China and its palaeoenvironmental implications. *J. Geophys. Res. Atmos.* **126**, e2020JD034336

(2021).
Duan, W. et al. The transfer of seasonal isotopic variability between precipitation and drip water at eight caves in the monsoon regions of China. *Geochim. Cosmochim. Ac.* **183**, 250–266 (2016).
Guo, H. et al. Global abnormal precipitation ^{18}O depletion during late/post monsoon season. *Earth Planet. Sci. Lett.* **641**, 118815 (2024).
Islam, M. R. et al. Controls on spatiotemporal variations of stable isotopes in precipitation across Bangladesh. *Atmos. Res.* **247**, 105224 (2021).
Li, G., Zhang, X., Xu, Y., Wang, Y. & Wu, H. Synoptic time-series surveys of precipitation $\delta^{18}O$ and its relationship with moisture sources in Yunnan, southwest China. *Quatern. Int.* **440**, 40–51 (2017).
Midhun, M. et al. The effect of monsoon circulation on the stable isotopic composition of rainfall. *J. Geophys. Res. Atmos.* **123**, 5205–5221 (2018).
Munksgaard, N. C. et al. Data Descriptor: Daily observations of stable isotope ratios of rainfall in the tropics. *Sci. Rep.* **9**, 14419 (2019).
Oza, H. et al. Hydrometeorological processes in semi-arid western India: insights from long term isotope record of daily precipitation. *Clim. Dyn.* **54**, 2745–2757 (2020).
Peng, T. et al. Stable isotopic characteristic of Taiwan's precipitation: A case study of western Pacific monsoon region. *Earth Planet. Sci. Lett.* **289**, 357–366 (2010).
Sengupta, S. & Sarkar, A. Stable isotope evidence of dual (Arabian Sea and Bay of Bengal) vapour sources in monsoonal precipitation over north India. *Earth Planet. Sci. Lett.* **250**, 511–521 (2006).
Wu, H. et al. Temporal variations of stable isotopes in precipitation from Yungui Plateau: Insights from moisture source and rainout effect. *J. Hydrometeorol.* **23**, 39–51 (2022).
Wu, X. et al. Effect of the El Niño–Southern Oscillation on hydrogen and oxygen isotope ratios of precipitation in Guilin, SW China. *Isot. Environ. Healt. S.* **57**, 67–81 (2020).
Wu, X., Zhu, X., Pan, M. & Zhang, M. Seasonal variability of oxygen and hydrogen stable isotopes in precipitation and cave drip water at Guilin, southwest China. *Environ. Earth Sci.* **72**, 3183–3191 (2014).
Wu, X., Zhu, X., Zhang, M., Bai, X. & Zhang, B. high-resolution stable isotope

record of atmospheric precipitation in Guilin. *Resour. Environ. in Yangtze Basin.* **22**, 7 (2013).

Yang, X., Davis, M., Acharya, S. & Yao, T. Asian monsoon variations revealed from stable isotopes in precipitation. *Clim. Dyn.* **51**, 2267–2283 (2017).

Zhang, J. et al. Temporal variations of precipitation driven by local meteorological parameters in southwest China: Insights from 9 years of continuous hydro-meteorological and isotope observations. *J. Hydrol. Reg. Stud.* **46**, 101345 (2023).

2) With regard to the transparency of the $\delta^{18}O_s$ records, we systematically collected all available $\delta^{18}O_s$ records within the study region (70–125° E, 20–27° N). All available $\delta^{18}O_s$ records from three sources: (1) the SISALv3 database (Kaushal et al., 2024; https://ora.ox.ac.uk/objects/uuid:1e91e2ac-ca9f-46e5-85f3-8d82d4d3cfd4); (2) speleothem datasets archived in the World Data Service for Paleoclimatology (NOAA/NCEI; https://www.ncdc.noaa.gov/data-access/paleoclimatology-data/datasets/speleothem); and (3) published studies. To ensure the quality of $\delta^{18}O_s$ records, we conducted data screening based on the following criteria and implemented a strict quality control process:

a) Data period selection (all $\delta^{18}O_s$ records must be dated back to -50,000 AD):

We constrained the data selection period to begin at -50,000 AD, primarily based on the temporal consistency of $\delta^{18}O_s$ records. We found that in this study region, some caves contain only a single $\delta^{18}O_s$ record, while others yield multiple $\delta^{18}O_s$ records within the same cave. It should be noted that some $\delta^{18}O_s$ records only cover the period before -50,000 AD, and this situation involves four caves including Mawmluh, Xiaobailong, Jiangjun, and Dongge. That is, these $\delta^{18}O_s$ records including MAW-3 (Mawmluh), XBL-26, XBL-27, XBL-65, XBL-48, XBL-4, XBL-7 (Xiaobailong), SJJ7, SJJ-300 (Jiangjun), and D8, D3, D4_2005_Kelly, D3_2005 (Dongge) have no data after -50,000 AD (Table R7). Hence, we eliminated these $\delta^{18}O_s$ records.

In addition, several $\delta^{18}O_s$ records contain data before and after -50,000 AD. For these, we only retained the data after -50,000 AD (Table R8).

Note that Shijiangjun cave has two speleothems including SJJ7 (26.20°N, 105.50°E) (Chen et al., 2021) and SJJ-300 (26.28°N, 106.07°E) (Li et al., 2021), and there is a 0.57° longitude difference (approximately 56 kilometers) between them (Table R9). Moreover, the $\delta^{18}O_s$ values of SJJ7 and SJJ-300 during the same period showed a significant deviation (Chen et al., 2021; Li et al., 2021). Therefore, we eliminated the $\delta^{18}O_s$ records from Shijiangjun cave. In total, 48 $\delta^{18}O_s$ records (48 speleothems) from 13 caves met these criteria (Table R10).

Table R7. Summary data for $\delta^{18}O_s$ records from the four caves eliminated due to lack of data after -50,000 AD.

No.	Cave	Speleothem name	Latitude (°N)	Longitude (°E)	Elevation (m a.s.l.)	First year (AD)	Last year (AD)	Source
1	Mawmluh	MAW-3	25.26	91.88	1160	-133539	-97281	SISAL/NOAA/Magiera et al., 2019
2	Xiaobailong	XBL-26	24.2	103.36	1500	-250010	-71110	SISAL/Cai et al., 2015
3	Xiaobailong	XBL-27	24.2	103.36	1500	-187510	-170390	SISAL/Cai et al., 2015
4	Xiaobailong	XBL-65	24.2	103.36	1500	-168780	-165300	SISAL/Cai et al., 2015
5	Xiaobailong	XBL-48	24.2	103.36	1500	-104590	-74660	SISAL/Cai et al., 2015
6	Xiaobailong	XBL-4	24.2	103.36	1500	-80001	-55468	SISAL/Cai et al., 2015
7	Xiaobailong	XBL-7	24.2	103.36	1500	-69050	-57720	SISAL/Cai et al., 2015
8	Jiangjun	JJ0403	22.95	104.82	200	-145156	-131843	SISAL/NOAA/Wassenburg et al., 2021
9	Jiangjun	JJ0406	22.95	104.82	200	-135327	-122389	SISAL/NOAA/Wassenburg et al., 2021
10	Dongge cave	D8	25.28	108.08	680	-223307	-215251	SISAL/NOAA/Cheng et al., 2016
11	Dongge	D3	25.28	108.08	680	-161550	-89250	SISAL/NOAA/Yuan et al., 2004
12	Dongge	D4_2005_Kelly	25.28	108.08	680	-144310	-118410	SISAL/NOAA/Kelly et al., 2006
13	Dongge	D3_2005	25.28	108.08	680	-123360	-97500	SISAL/NOAA/Kelly et al., 2006

Table R8. Summary data for $\delta^{18}O_s$ records contain data before and after -50,000 AD.

No.	Cave	Speleothem name	Latitude (°N)	Longitude (°E)	Elevation (m a.s.l.)	First year (AD)	Last year (AD)	Source
1	Xiaobailong	XBL-1	24.2	103.36	1500	-50644	-34129	NOAA/Cai et al., 2006
2	Southeast Yunnan	SL08	23.5	104	2000	-63272	-10573	Liu et al., 2020
3	Southeast Yunnan	A15B	23.5	104	2000	-50641	-10	Liu et al., 2020
4	Dongge	D4_2004	25.28	108.08	680	-146450	1834	SISAL/NOAA/Yuan et al., 2004
5	Fengyu	F1	24.42	110.28	150	-62053	-2636	Li et al., 2017

Table R9. Summary data for $\delta^{18}O_s$ records from the Shijiangjun Cave.

No.	Cave	Speleothem name	Latitude (°N)	Longitude (°E)	Elevation (m a.s.l.)	First year (AD)	Last year (AD)	Source
1	Shijiangjun	SJJ7	26.2	105.5	1300	-1159	1256	SISAL/NOAA/Chen et al., 2021
2	Shijiangjun	SJJ-300	26.28	106.07	1300	1200	1786	Li et al., 2021

Table R10. Summary data for 48 $\delta^{18}O_s$ records from 13 caves that meet the data period selection criteria, with their corresponding data selection status.

No.	Cave	Speleothem name	Latitude (° N)	Longitude (° E)	Elevation (m a.s.l.)	First year (AD)	Last year (AD)	Source	Data selection status
1	Wah Shikar	WS-B	25.25	91.87	1290	1399	2006	SISAL/NOAA/Sinha et al., 2011	Select all records
2	Mawmluh	MWS-2	25.26	91.82	1290	-40703	-33652	NOAA/Cheng et al., 2021	Select all records
3	Mawmluh	MWS-1	25.26	91.88	1160	-31838	-3583	SISAL/NOAA/Dutt et al., 2015	Select all records
4	Mawmluh	MWS-1	25.25	91.9	1290	-22887	-20557	NOAA/Dong et al., 2022	Exclude all records
5	Mawmluh	KM-1	25.26	91.88	1160	-20767	-4655	SISAL/NOAA/Huguet et al., 2018	Exclude all records
6	Mawmluh	MAW-6	25.26	91.88	1160	-13957	-4561	SISAL/NOAA/Lechleitner et al., 2017	Exclude all records
7	Mawmluh	KM-A	25.26	91.88	1160	-10445	-1703	SISAL/NOAA/Berkelhammer et al., 2012	Retain data spanning from 3581 BC to 1703 BC
8	Mawmluh	ML.2	25.26	91.88	1160	-2584	-1416	SISAL/NOAA/Kathayat et al., 2018	Retain data spanning from 1700 BC to 1416 BC
9	Mawmluh	ML.1	25.26	91.88	1160	-2490	-1828	SISAL/NOAA/Kathayat et al., 2018	Exclude all records
10	Mawmluh	ML-5	25.26	91.71	1290	1081	1855	NOAA/Kathayat et al., 2022	Select all records
11	Mawmluh	ML-8	25.26	91.71	1290	1855	1906	NOAA/Kathayat et al., 2022	Retain data spanning from 1854.80 AD to 1906 AD
12	Mawmluh	MAW-0201	25.26	91.88	1160	1964	2012	SISAL/NOAA/ Myers et al., 2015	Select all records
13	Cherrapunji	Cherrapunji-2	25.2	92.45	1100	-24860	-19932	NOAA/Dong et al., 2022	Select all records
14	Lin Noe Twin	LNT02	21.24	96.44	1300	-35275	-28192	Liu et al., 2020	Retain data spanning from 35275 BC to 28675 BC
15	Lin Noe Twin	LNT01	21.24	96.44	1300	-29367	-27971	Liu et al., 2020	Exclude all records
16	Lin Noe Twin	LNT05	21.24	96.44	1300	-28464	-24153	Liu et al., 2020	Select all records
17	Lin Noe Twin	LNT09B	21.24	96.44	1300	-19724	-18360	Liu et al., 2020	Select all records
18	Lin Noe Twin	LNT04	21.24	96.44	1300	-10728	-10328	Liu et al., 2020	Select all records
19	Lin Noe Twin	LNT09U	21.24	96.44	1300	-162	1990	Liu et al., 2020	Select all records
20	Tham Doun Mai	TM4	20.75	102.65	352	-7359	1170	SISAL/NOAA/Griffiths et al., 2020	Exclude all records
21	Tham Doun Mai	TM5	20.75	102.65	352	-7286	1267	SISAL/NOAA/Griffiths et al., 2020	Select all records
22	Tham Doun Mai	TM-17	20.75	102.65	352	-6961	-5625	NOAA/Wood et al., 2023	Exclude all records
23	Tham Doun Mai	TM11	20.75	102.65	352	-4589	-2937	SISAL/NOAA/Griffiths et al., 2020	Exclude all records
24	Tham Doun Mai	TM-17	20.75	102.65	352	-51	1879	SISAL/NOAA/Wang et al., 2019	Retain data spanning from 1267.4 AD to 1879.5 AD
25	Xiaobailong	XBL-1	24.2	103.36	1500	-50644	-34129	NOAA/Cai et al., 2006	Retain data spanning from 49997 BC to 41707 BC
26	Xiaobailong	XBL-29	24.2	103.36	1500	-41680	-3390	SISAL/Cai et al., 2015	Select all records
27	Xiaobailong	XBL-3	24.2	103.36	1500	-39470	-28190	SISAL/Cai et al., 2015	Exclude all records
28	Xiaobailong	XBL41	24.2	103.36	1500	1760	1981	NOAA/Tan et al., 2017	Select all records
29	Southeast Yunnan	SL08	23.5	104	2000	-63272	-10573	Liu et al., 2020	Exclude all records
30	Southeast Yunnan	A15B	23.5	104	2000	-50641	-10	Liu et al., 2020	Retain data spanning from 49966.6 BC to 20334.9 BC
31	Southeast Yunnan	SY10	23.5	104	2000	-25211	-6058	Liu et al., 2020	Retain data spanning from 20329.5 BC to 7653.53 BC
32	Southeast Yunnan	SL10	23.5	104	2000	-7755	-4791	Liu et al., 2020	Exclude all records
33	Southeast Yunnan	A15U	23.5	104	2000	-7507	1718	Liu et al., 2020	Select all records
34	Southeast Yunnan	A14	23.5	104	2000	-272	340	Liu et al., 2020	Exclude all records
35	Wulu	Wu88	26.05	105.08	1440	-48818	-43693	SISAL/NOAA/Liu et al., 2023	Select all records
36	Wulu	Wulu-30	26.05	105.08	1440	-39531	-34709	SISAL/NOAA/Cheng et al., 2021	Select all records
37	Wulu	Wu37	26.05	105.08	1440	-1357	1915	SISAL/Zhao et al., 2020	Select all records
38	Yamen	Y1	25.48	107.9	570	-14369	-5372	SISAL/NOAA/Yang et al., 2010	Select all records
39	Dongge	D4_2004	25.28	108.08	680	-146450	1834	SISAL/NOAA/Yuan et al., 2004	Exclude all records
40	Dongge	D4_2005	25.28	108.08	680	-13860	1966	SISAL/NOAA/Dykoski et al., 2005	Select all records
41	Dongge	DA_2005	25.28	108.08	680	-6930	2000	SISAL/NOAA/Wang et al., 2005	Exclude all records
42	Dongge	DA_2009	25.28	108.08	680	-6394	-6040	SISAL/NOAA/Cheng et al., 2009	Exclude all records
43	Fengyu	F1	24.42	110.28	150	-62053	-2636	Li et al., 2017	Select all records
44	Xiangshui	X3	25.25	110.92	380	-48011	-17720	SISAL/NOAA/Cosford et al., 2008	Select all records
45	Xianyun	XY4-12	25.56	117	970	-47600	-43380	Zhang et al., 2024	Select all records
46	Xianyun	XY1	25.56	117	970	-39560	-35100	Zhang et al., 2021	Select all records
47	Xianyun	XY3-28	25.56	117	970	-22838	-21580	Qiu et al., 2024	Select all records
48	Xianyun	XY2-27	25.56	117	970	-18348	-13233	Qiu et al., 2022	Select all records

b) The selection criteria for the different $\delta^{18}O_s$ records from the same cave:

When multiple $\delta^{18}O_s$ records from the same cave did not overlap temporally, we combined them to produce a composite record. If a longer $\delta^{18}O_s$ record covered all other records from the same cave, we only selected this longest record. When $\delta^{18}O_s$ records from the same cave partially overlapped temporally, we kept the longest record and merged it with others after removing overlapping segments from the shorter records. The final dataset included 13 $\delta^{18}O_s$ records that met these criteria: 5 individual records (5 speleothems from 5 caves) and 8 composite records (28 speleothems from 8 caves) (Table R11).

Table R11. Summary data for the 13 caves in the region of 20–27° N, 70–125° E. The caves are listed by longitude from west to east.

No.	Site_name	Speleothem name	Latitude (°N)	Longitude (°E)	Elevation (m a.s.l.)	Reference
1	Wah Shikar	WS-B	25.25	91.87	1290	Sinha et al., 2011
2	Mawmluh	MWS-2	25.26	91.82	1290	Cheng et al., 2021
		MWS-1	25.26	91.88	1160	Dutt et al., 2015
		KM-A	25.26	91.88	1160	Berkelhammer et al., 2012
		ML.2	25.26	91.88	1160	Kathayat et al., 2018
		ML-5	25.26	91.71	1290	Kathayat et al., 2022
		ML-8	25.26	91.71	1290	
		MAW-0201	25.26	91.88	1160	Myers et al., 2015
3	Cherrapunji	Cherrapunji-2	25.2	92.45	1100	Dong et al., 2022
4	Lin Noe Twin	LNT02	21.24	96.44	1300	Liu et al., 2020
		LNT05	21.24	96.44	1300	
		LNT09B	21.24	96.44	1300	
		LNT04	21.24	96.44	1300	
		LNT09U	21.24	96.44	1300	
5	Tham Doun Mai	TM5	20.75	102.65	352	Griffiths et al., 2020
		TM-17	20.75	102.65	352	Wang et al., 2019
6	Xiaobailong	XBL-1	24.2	103.36	1500	Cai et al., 2006
		XBL-29	24.2	103.36	1500	Cai et al., 2015
		XBL41	24.2	103.36	1500	Tan et al., 2017
7	Southeast Yunnan	A15B	23.5	104	2000	Liu et al., 2020
		SY10	23.5	104	2000	
		A15U	23.5	104	2000	
8	Wulu	Wu88	26.05	105.08	1440	Liu et al., 2023
		Wulu-30	26.05	105.08	1440	Cheng et al., 2021
		Wu37	26.05	105.08	1440	Zhao et al., 2020
9	Yamen	Y1	25.48	107.9	570	Yang et al., 2010
10	Dongge	D4_2005	25.28	108.08	680	Dykoski et al., 2005
11	Fengyu	F1	24.42	110.28	150	Li et al., 2017
12	Xiangshui	X3	25.25	110.92	380	Cosford et al., 2008
13	Xianyun	XY4-12	25.56	117	970	Zhang et al., 2024
		XY1	25.56	117	970	Zhang et al., 2021
		XY3-28	25.56	117	970	Qiu et al., 2024
		XY2-27	25.56	117	970	Qiu et al., 2022

c) Quality evaluation of $\delta^{18}O_s$ records

To evaluate the quality of our final dataset, we compared the $\delta^{18}O_s$ records from the different caves. The results showed that these records exhibited broadly similar fluctuations over the past 50,000 years (Figure. R6a). In particular, $\delta^{18}O_s$ values increased considerably from -19,931 to -14,370 AD and decreased markedly from -14,369 to -6,961 AD (Figure. R6a). These consistent patterns support the reliability of the dataset.

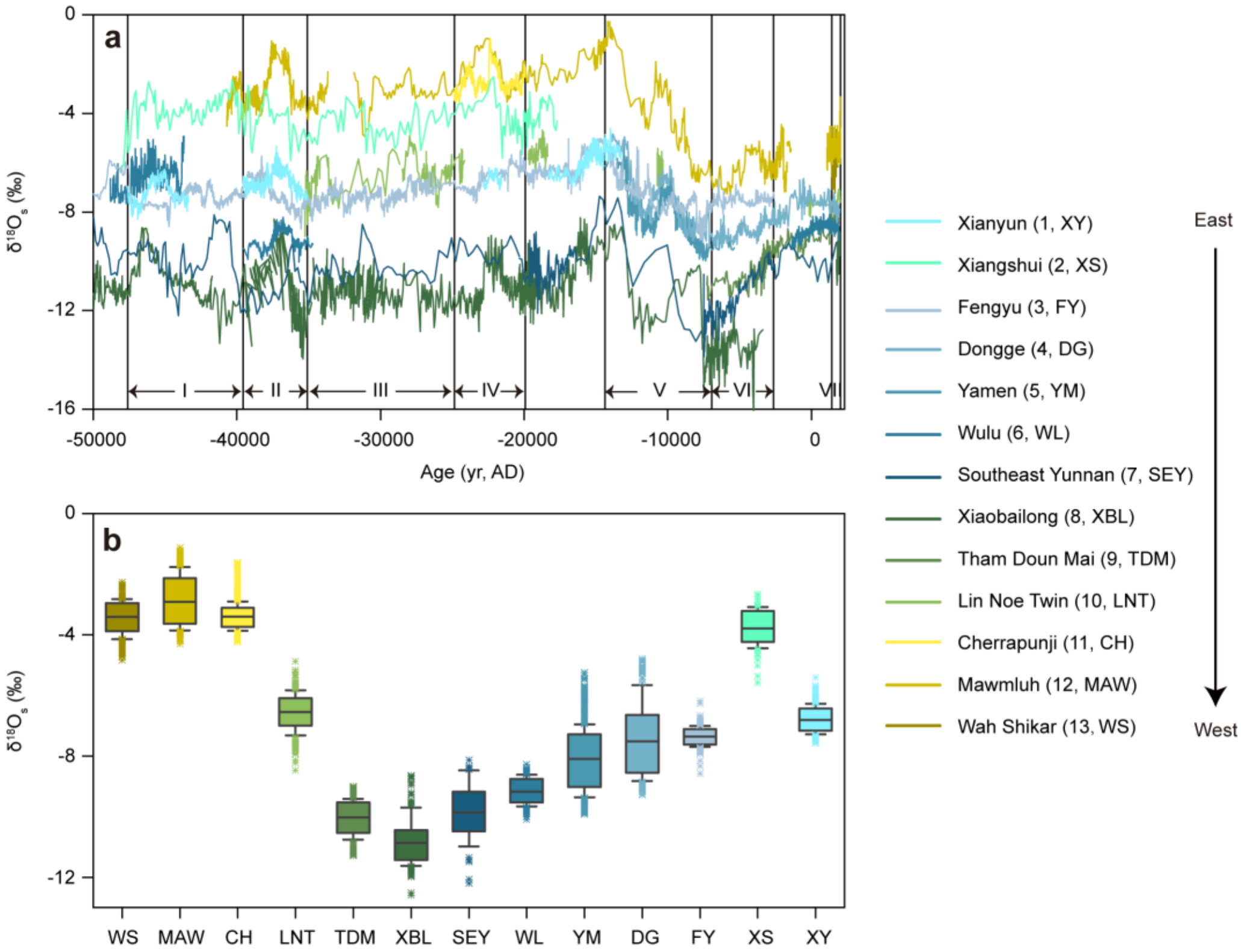

Figure R6. Comparisons of the $\delta^{18}O_s$ records from 13 caves along the transect during the last 50,000 years. a $\delta^{18}O_s$ records from the 13 caves along the transect. Vertical lines indicate the dividing line of each sub-period. b Spatial distribution of multi-year mean $\delta^{18}O_s$ values from the 13 caves, calculated using a “relative value” method (see Materials and Methods). In each box plot, the box represents the 20th–80th percentile range, whiskers extend to the 10th–90th percentiles, horizontal lines inside the boxes indicate the mean, and stars represent outliers. Numbers in brackets correspond to the open circles in Fig. 1a. $\delta^{18}O_s$ values are reported as per mil (‰) deviations from Vienna Peedee belemnite (VPDB).

3) The updated $\delta^{18}O_p$ and $\delta^{18}O_s$ data:

Following your comments, we re-examined the database based on the selection criteria for $\delta^{18}O_p$ and $\delta^{18}O_s$ data as mentioned above. The results showed that, for $\delta^{18}O_p$ data, the number of sites and sample size remained unchanged. For $\delta^{18}O_s$ record, we found a new SISALv3 database compared with the SISALv2 version. Note that this new version was published in 2024 (i.e., after the work of our previous manuscript). Compared to the SISALv2 version that we cited, the SISALv3 database includes additional $\delta^{18}O_s$ records. Hence, we supplemented and adjusted the $\delta^{18}O_s$

records based on the SISALv3 database as follows:

a) For the Mawmluh cave: we added two $\delta^{18}O_s$ records including MWS-1 (Dutt et al., 2015) and ML.2 (Kathayat et al., 2018).

b) For the Tham Doun Mai cave: in the previous manuscript, we only used TM-17 (Wang et al., 2019) and TM-17 (Wood et al., 2023) $\delta^{18}O_s$ records. The former covers the period from -51 to 1879 yr, AD, and the latter covers the period from -6960.9 to -5624.6 yr, AD. In the SISALv3 database, we found that the TM5 $\delta^{18}O_s$ record from this cave covers the period from -7286 to 1267 yr, AD. Clearly, the duration of the TM-17 (Wood et al., 2023) $\delta^{18}O_s$ records was included in that of the TM5 $\delta^{18}O_s$ record. According to the selection criterion of "If a longer $\delta^{18}O_s$ record completely covers all other records in the same cave, we will select only this longest record", we used the TM5 record from this cave, and eliminated the TM-17 (Wood et al., 2023) $\delta^{18}O_s$ records.

c) For the Xiaobailong cave: we added the XBL-29 $\delta^{18}O_s$ record from this cave (Cai et al., 2015).

d) For the Wulu cave: we added two $\delta^{18}O_s$ records from this cave including Wu88 (Liu et al., 2023) and Wu37 (Zhao et al., 2020).

e) For the Dongge cave: in the previous manuscript, we used the D4 (Dykoski et al., 2005) and DA (Cheng et al., 2009) $\delta^{18}O_s$ records. The former covers the period from -13860 to 1966 yr, AD, and the latter covers the period from -6394 to -6040 yr, AD. Clearly, the period of the former included that of the latter. Hence, we retained the former and eliminated the latter.

It should be noted that the core objective of this study is to reveal the spatial patterns of $\delta^{18}O_s$ values along the transect rather than the temporal changes of $\delta^{18}O_s$ records. Hence, our study is different from traditional paleoclimate reconstruction studies. Therefore, we compiled all publicly available $\delta^{18}O_s$ data within the study region that met the selection criteria. Moreover, we found that the updated dataset does not affect our conclusions.

Following your comments and suggestions, we added three paragraphs to improve the transparency of the compiled dataset., i.e., " ***Selection criteria for $\delta^{18}O_s$ data***

We collected all available $\delta^{18}O_s$ records from three sources: (1) the SISALv3 database[0]; https://ora.ox.ac.uk/objects/uuid:1e91e2ac-ca9f-46e5-85f3-8d82d4d3cfd4); (2) speleothem datasets archived in the World Data Service for Paleoclimatology (NOAA/NCEI; https://www.ncdc.noaa.gov/data-access/paleoclimatology-data/datasets/speleothem); and (3) published studies. We compiled $\delta^{18}O_s$ records using the following selection criteria: (1) all $\delta^{18}O_s$ records must be within the study region (20–27°N, 70–125°E); and (2) all records must be dated back to -50,000 AD. We note that Shijiangjun cave has two speleothems including SJJ7 (26.20°N, 105.50°E)[Error! Reference source not found.] and SJJ-300 (26.28°N, 106.07°E)[Error! Reference source not found.], and there is a 0.57° longitude difference (approximately 56 kilometers) between them. Moreover, the $\delta^{18}O_s$ values of SJJ7 and SJJ-300 during the same period had significant deviations[Error! Reference source not found.,Error! Reference source not found.]. Therefore, we eliminated the $\delta^{18}O_s$ records from Shijiangjun cave. In total, 48 $\delta^{18}O_s$ records (48 speleothems) from 13 caves met these criteria.

When multiple $\delta^{18}O_s$ records from the same cave did not overlap temporally, we combined them to produce a composite record. If a longer $\delta^{18}O_s$ record covered all other records from the same cave, we only selected this longest record. When $\delta^{18}O_s$ records from the same cave partially overlapped temporally, we kept the longest record and merged it with others after removing overlapping segments from the shorter records. The final dataset included 13 $\delta^{18}O_s$ records that met these criteria: 5 individual records (5 speleothems from 5 caves) and 8 composite records (28 speleothems from 8 caves) (Supplementary Table S3).

To evaluate the quality of our final dataset, we compared the $\delta^{18}O_s$ records from the different caves. The results showed that these records exhibited broadly similar fluctuations over the past 50,000 years (Fig. 7a). In particular, $\delta^{18}O_s$ values increased considerably from -19,931 to -14,370 AD and decreased markedly from -14,369 to -6,961 AD (Fig. 7a). These consistent patterns support the reliability of the dataset.”
Please see L693–L719 in the revised manuscript.

References:

Berkelhammer, M. et al. An abrupt shift in the Indian monsoon 4000 years ago. in *Climates, Landscapes, and Civilizations* (eds. Liviu Giosan, Dorian Q. Fuller,

Kathleen Nicoll, Rowan K. Flad, & Peter D. Clift) 75–88 (American Geophysical Union, 2012).

Cai, Y. et al. High-resolution absolute-dated Indian Monsoon record between 53 and 36 ka from Xiaobailong Cave, southwestern China. *Geology*, **34**, 621–624 (2006).

Cai, Y. et al. Variability of stalagmite-inferred Indian monsoon precipitation over the past 252,000 y. *Proc. Natl. Acad. Sci. U.S.A.* **112**, 2954-2959 (2015).

Chen, C. J. et al. Karst hydrological changes during the Late-Holocene in Southwestern China. *Quaternary Sci. Rev.* **258**, 106865 (2021).

Cheng, H. et al. Onset and termination of Heinrich Stadial 4 and the underlying climate dynamics. *Commun. Earth Environ.* **2**, 230 (2021).

Cheng, H. et al. The Asian monsoon over the past 640,000 years and ice age terminations. *Nature* **534**, 640–646 (2016).

Cheng, H. et al. Timing and structure of the 8.2 kyr B.P. event inferred from $\delta^{18}O$ records of stalagmites from China, Oman, and Brazil. *Geology* **37**, 1007-1010 (2009).

Cosford, J. et al. Millennial-scale variability in the Asian monsoon: Evidence from oxygen isotope records from stalagmites in southeastern China. *Palaeogeogr. Palaeocl.* **266**, 3–12 (2008).

Dong, X. et al. Coupled atmosphere-ice-ocean dynamics during Heinrich Stadial 2. *Nat. Commun.* **13**, 5867 (2022).

Dutt, S. et al. Abrupt changes in Indian summer monsoon strength during 33,800 to 5500 years B.P. *Geophys. Res. Lett.* **42**, 5526–5532 (2015).

Dykoski, C. A. et al. A high-resolution, absolute-dated Holocene and deglacial Asian monsoon record from Dongge Cave, China. *Earth Planet. Sci. Lett.* **233**, 71–86 (2005).

Griffiths, M. L. et al. End of Green Sahara amplified mid- to late Holocene megadroughts in mainland Southeast Asia. *Nat. Commun.* **11**, 4204 (2020).

Huguet, C. et al. Temperature and Monsoon Tango in a Tropical Stalagmite: Last Glacial-Interglacial Climate Dynamics. *Sci. Rep.* **8**, 5386 (2018).

Kathayat, G. et al. Protracted Indian monsoon droughts of the past millennium and their societal impacts. *Proc. Natl. Acad. Sci. U.S.A.* **119**, e2207487119 (2022).

Kathayat, G. et al., Evaluating the timing and structure of the 4.2 ka event in the

Indian summer monsoon domain from an annually resolved speleothem record from Northeast India. *Clim. Past.* **14**, 1869-1879 (2018).

Kaushal, N. et al. SISALv3: Speleothem Isotopes Synthesis and AnaLysis database version 3.0. University of Oxford (2024).

Kelly, M. J. et al. High-resolution characterization of the Asian Monsoon between 146,000 and 99,000 years B.P. from Dongge Cave, China and global correlation of events surrounding Termination II. *Palaeogeogr. Palaeoclimatol. Palaeoecol.* **236**, 1-38 (2006).

Lechleitner, F. A. et al. Climatic and in-cave influences on $\delta^{18}O$ and $\delta^{13}C$ in a stalagmite from northeastern India through the last deglaciation. *Quat. Res.* **88**, 458-471 (2017).

Li, H. et al. High-resolution $\delta^{18}O$ and $\delta^{13}C$ records during the past 65 ka from Fengyu Cave in Guilin: Variation of monsoonal climates in south China. *Quatern. Int.* **441**, 117–128 (2017).

Li, T. Y. et al. Little Ice Age climate changes in Southwest China from a stalagmite $\delta^{18}O$ record. *Palaeogeogr. Palaeoclimatol. Palaeoecol.* **562**, 110167 (2021).

Liu, D., Mi, X., Liu, S. & Wang, Y. Multi-phased Asian hydroclimate variability during Heinrich Stadial 5. *Clim. Dyn.* **60**, 4003–4016 (2023).

Liu, G. et al. On the glacial-interglacial variability of the Asian monsoon in speleothem $\delta^{18}O$ records. *Sci. Adv.* **6**, eaay8189 (2020).

Magiera, M. et al. Local and regional Indian summer monsoon precipitation dynamics during Termination II and the last interglacial. *Geophys. Res. Lett.* **46**, 12454–12463 (2019).

Myers, C. G. et al. Northeast Indian stalagmite records Pacific decadal climate change: Implications for moisture transport and drought in India. *Geophys. Res. Lett.* **42**, 4124–4132 (2015).

Qiu, W. et al. Double-plunge structure of the East Asian summer monsoon during Heinrich stadial 1 recorded in Xianyun Cave, southeastern China. *Quaternary Sci. Rev.* **282**, 107442 (2022).

Qiu, W. et al. Synchronous anti-phase variations between the East Asian summer monsoon and South America Summer Monsoon during Heinrich stadial 2. *Quaternary Sci. Rev.* **323**, 108434 (2024).

Sinha, A. et al. The leading mode of Indian Summer Monsoon precipitation variability

during the last millennium. *Geophys. Res. Lett.* **38**, L15703 (2011).

Tan, L. et al. Decreasing monsoon precipitation in southwest China during the last 240 years associated with the warming of tropical ocean. *Clim. Dyn.* **48**, 1769–1778 (2017).

Wang, J. et al. Hydroclimatic variability in Southeast Asia over the past two millennia. *Earth Planet. Sci. Lett.* **525**, 115737 (2019).

Wang, Y. et al. The Holocene Asian Monsoon: links to solar changes and North Atlantic climate. *Science* **308**, 854-857 (2005).

Wassenburg, J. A. et al. Penultimate deglaciation Asian monsoon response to North Atlantic circulation collapse. *Nat. Geosci.* **14**, 937–941 (2021).

Wood, C. T. et al. High-resolution, multiproxy speleothem record of the 8.2 ka event from Mainland Southeast Asia. *Paleoceanogr. Paleoclimatol.* **38**, e2023PA004675 (2023).

Yang, Y. et al. Precise dating of abrupt shifts in the Asian Monsoon during the last deglaciation based on stalagmite data from Yamen Cave, Guizhou Province, China. *Sci. China Earth Sci.* **53**, 633–641 (2010).

Yuan, D. et al. Timing, duration, and transitions of the Last Interglacial Asian monsoon. *Science* **304**, 575-578 (2004).

Zhang, X. et al. East Asian summer monsoon evolution during Heinrich Stadial 5. *Palaeogeogr. Palaeocl.* **654**, 112467 (2024).

Zhang, X. et al. Three-phase structure of the East Asia summer monsoon during Heinrich Stadial 4 recorded in Xianyun Cave, southeastern China. *Quaternary Sci. Rev.* **274**, 107267 (2021).

Zhao, K. et al. Late Holocene monsoon precipitation changes in southern China and their linkage to Northern Hemisphere temperature. *Quaternary Sci. Rev.* **232**, 106191 (2020).

3. Frequent use of arbitrary boundaries and definitions. At several points in the manuscript, authors define spatial and temporal domains without clear justification, or rationale, for why these definitions were selected. This lack of justification for certain choices left me questioning several aspects of the study, and especially the interpretations made from the data. We suggest that where these boundaries are included, they are accompanied by 1-2 sentences justifying why they were chosen,

and/or alongside the literature upon which these decisions were made.

Re: We thank you for pointing these issues out. We have tried to address all the issues as follows.

1) With regard to the definition of the dividing line, in the previous manuscript, we intended to use the seasonal variations of $\delta^{18}O_p$ (observed data) along the transect as the criterion for regional division, because that we observed a significant shift in the seasonal variations of $\delta^{18}O_p$ around ~98°E: West of ~98°E, the multi-year monthly mean $\delta^{18}O_p$ values exhibited a continuous decrease from May to September/October; East of ~98°E, the multi-year monthly mean $\delta^{18}O_p$ values showed a continuous decrease from May to July/August, followed by an increase.

Following your comments, we carefully checked the published work, and found that some scientists including Jin & Chen (1982) and Wang & LinHo (2002) had already demonstrated that ~98°E is the dividing line between the South Asian monsoon and the East Asian monsoon from the perspective of atmospheric dynamics. Thus, the dividing line revealed by the $\delta^{18}O_p$ data in our study essentially serve as another evidence for the conclusion. As mentioned above, we rewrote this section. That is, we first cited the previous study regarding ~98°E as the dividing line of the Asian monsoon region, and then analyzed the seasonal variation of $\delta^{18}O_p$ in the eastern and western domains and ultimately confirmed the significant differences in seasonal variation of $\delta^{18}O_p$ between the eastern and western domains. Please see L123–L137 in the revised manuscript.

2) With regard to the division of the pre-monsoon, monsoon and post-monsoon seasons, following you suggestion, we added one sentence to explain why we used the definition of the pre-monsoon, monsoon, post-monsoon seasons, i.e., "*Given that circulation fields and precipitation amount in the Asian monsoon region exhibit distinct seasonal variations*[50]*, we analyzed the spatial distribution of* $\delta^{18}O_p$ *values at the 12 primary sites during the pre-monsoon (February–May) (Fig. 2a-d), monsoon (June–September) (Fig. 2e-h), and post-monsoon (October–January) seasons (Supplementary Fig. 4). The post-monsoon season contributes minimally to annual precipitation (Supplementary Figs. 3 and 5) and is therefore not considered further (Supplementary Fig. 4).*" Please see L139–L146 in the revised manuscript.

Reference:

50. Zhang, J. et al. Coupled effects of moisture transport pathway and convection on stable isotopes in precipitation across the East Asian monsoon region: Implications for paleoclimate reconstruction. *J. Climate* **34**, 9811–9822 (2021).

55. Jin, Z. & Chen, L. On the medium-range oscillation of the East Asia monsoon circulation system and its relation with the Indian monsoon system. *In The National Symposium Collections on the Tropical Summer Monsoon* 204-215 (People's Press Yunnan Province, 1982).

56. Wang, B., & LinHo, Rainy Season of the Asian–Pacific Summer Monsoon. *J. Climate* **15**, 386–398 (2002).

4. Presentation and aesthetic of figures. While we agree that personal taste plays a large role in figure creation, we can't help but feel as though these figures were assembled in a great hurry/as an afterthought, and some even seem unfinished. For example, there are multiple whisker plot figures, colour-coding is largely inconsistent across the figures, and many seem as though they have been merely copied and pasted from the software used to create them; again assuming that the reader already knows how to read them. For some journals this would suffice, however, papers in Nature Communications typically contain figures that not only look aesthetically pleasing, but are also accessible to both specialist and non-specialist readers. With this in mind, we suggest the authors consider (a) how their figures could be combined so not to repeat styles several times, and (b) inclusion of a conceptual, cartoon-style figure to help visualize their proposed mechanisms. Together, both would greatly improve the accessibility of the article to a broad audience.

Re: We thank you for pointing these issues out. Indeed, our previous figures were not visually appealing due to largely inconsistent color-coding and overly similar styles across them. Following your comments and suggestions, we have improved the figures in two key ways:

1) Consolidation and Style Refinement: We combined certain figures to avoid repetitive styles. For example, in the original Fig. 1, we used 12 colors to represent 12 months. However, this approach lacked practical significance. Moreover, this approach closely resembled the style of Fig. 2, and risked confusing readers. As you

suggested, we revised it into a clearer format (Figure R7, as follows). The updated Fig. 1 can be found in the revised manuscript.

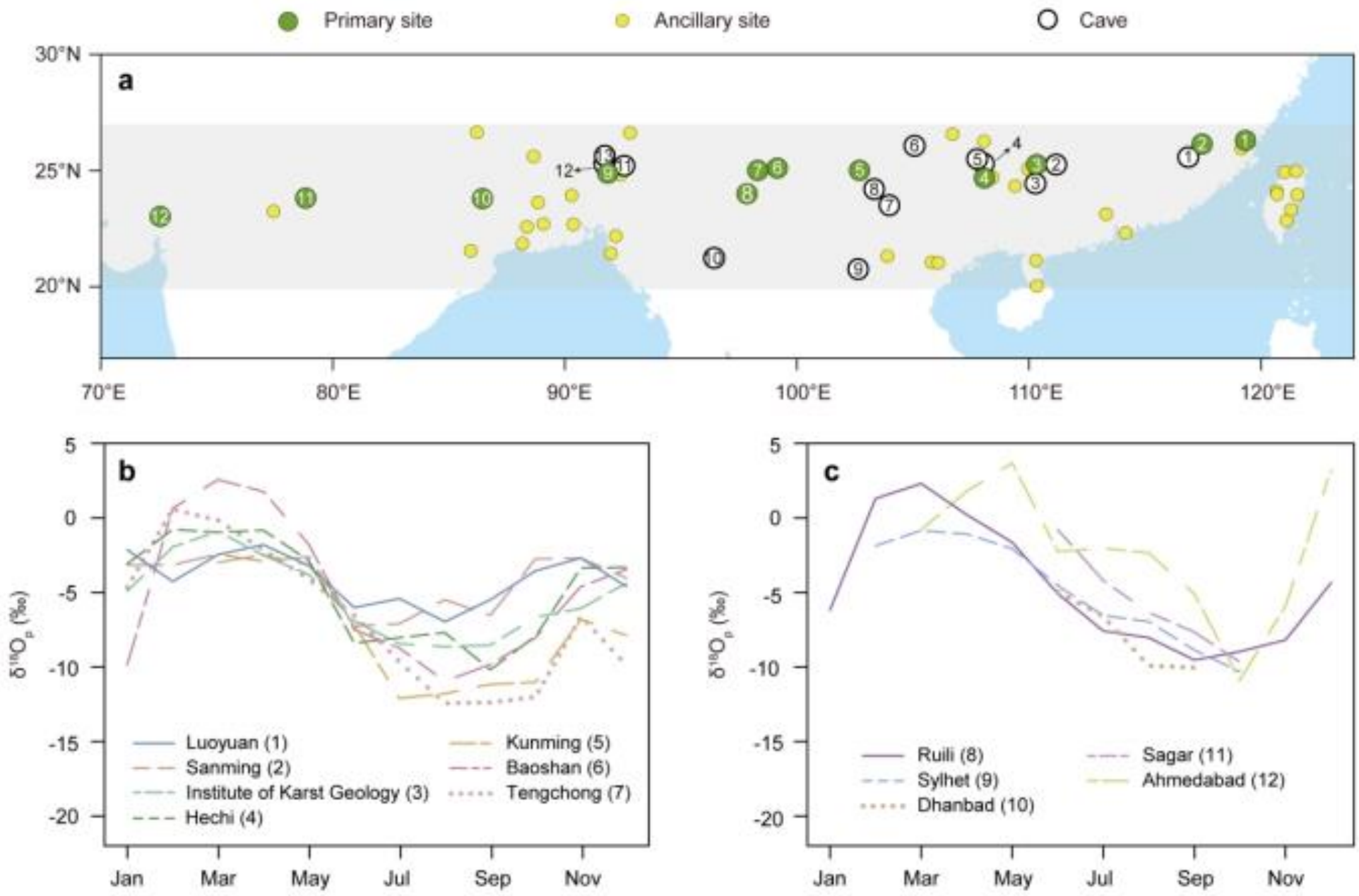

Figure R7. Sampling sites in the study region and seasonal variations of $\delta^{18}O_p$ at the 12 primary sites. a Precipitation sampling sites and locations of caves. Green dots indicate the 12 primary precipitation sites selected for this study (Table S1), while yellow dots represent the 34 ancillary precipitation sites (Table S2). Open circles mark the locations of the 13 caves analyzed within the study region (Table S3). b, c Seasonal variations of $\delta^{18}O_p$ at the 12 primary sites in the eastern (b) and western (c) domains. Numbers in the brackets in b and c correspond to the green dots in a. $\delta^{18}O_p$ values are expressed as per mil (‰) deviations relative to VSMOW or VSMOW2.

For Figure 3, we merged some sub-figures into a vertically arranged composite figure (to avoid repetitive styling) and then integrated it with two other related diagrams (Figure R8, as follows). The revised version is now presented as the new Fig. 3 in the revised manuscript.

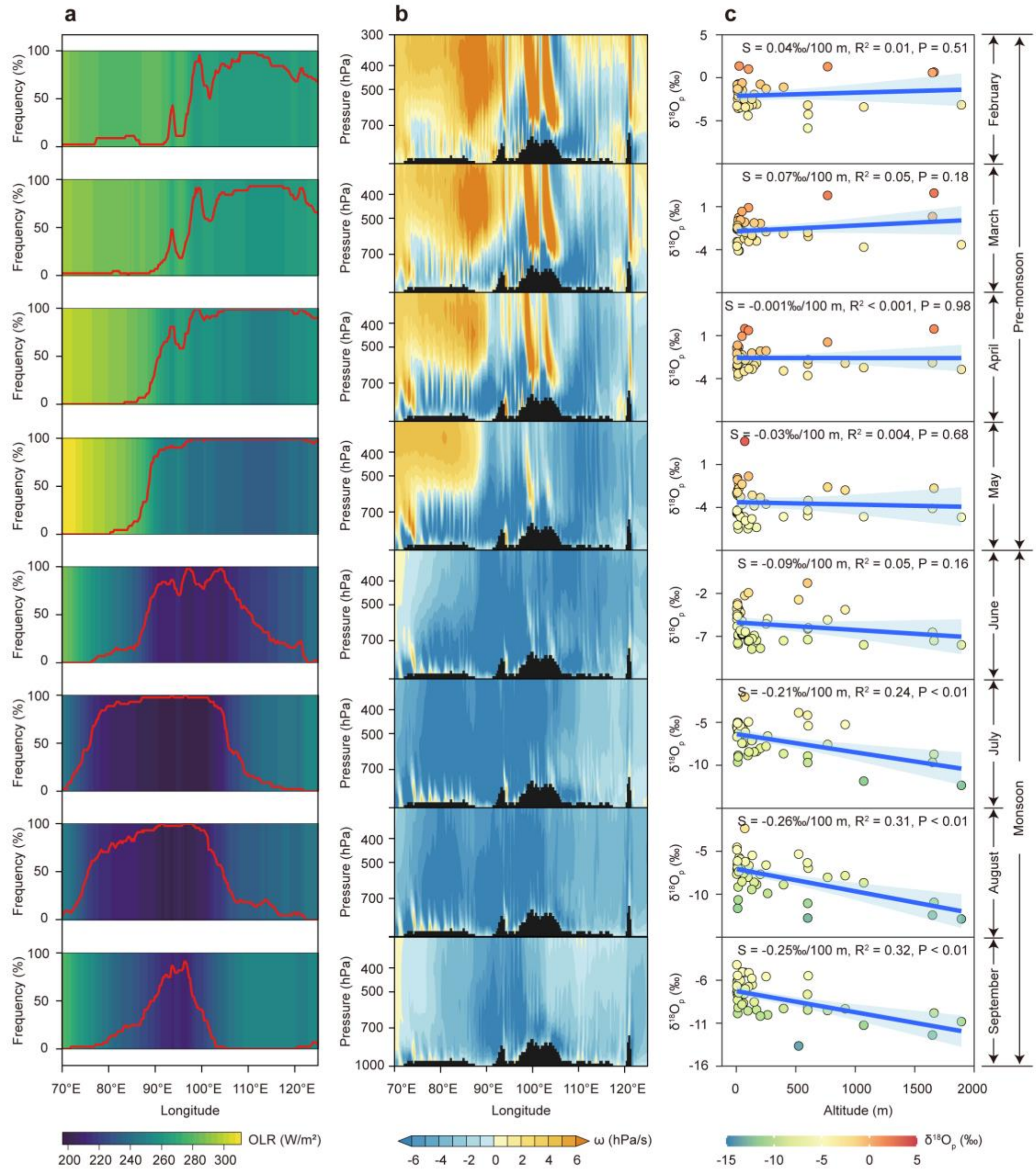

Figure R8. Seasonal changes of atmospheric updraft and altitude effect on $\delta^{18}O_p$ during the pre-monsoon and monsoon seasons over 1979–2022. a Seasonal variations in outgoing longwave radiation (OLR, shading) and the frequencies of relatively low OLR values (red line) along the transect. Lower OLR values indicate stronger convection. b Vertical profiles of meridional mean vertical velocity (ω), averaged over 20–27° N. Negative ω values indicate upward motion (convection), while positive values indicate downward motion (subsidence). c Linear regressions of $\delta^{18}O_p$ at the 46 sites across the study region against altitude, fitted for each month. $\delta^{18}O_p$ values are expressed as per mil (‰) deviations relative to VSMOW or VSMOW2.

2) Enhanced Visualization: We added conceptual, cartoon-style symbols to better

illustrate the proposed mechanisms. For Figure 2, to help readers more intuitively identify the westward expansion of low $\delta^{18}O_P$ values, we added conceptual symbols (horizontal arrows) to visually illustrate the proposed mechanism (Figure R9). The updated Fig. 2 is now included in the revised manuscript.

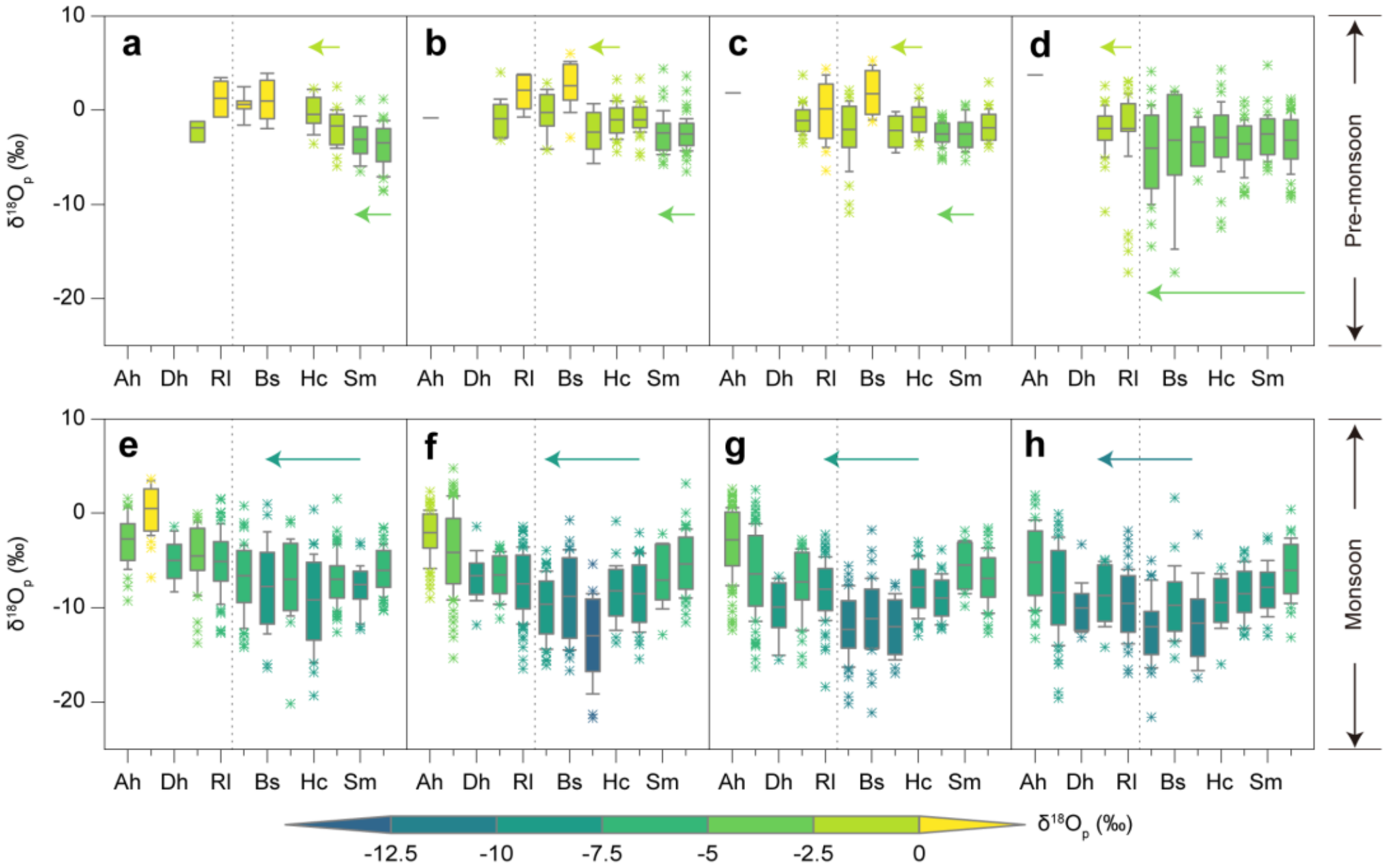

Figure R9. Spatial distributions of $\delta^{18}O_P$ at the 12 primary sites along the transect during the pre-monsoon and monsoon seasons. a–d $\delta^{18}O_P$ distributions during the pre-monsoon season: February (a), March (b), April (c), and May (d). e–h $\delta^{18}O_P$ distributions during the monsoon season: June (e), July (f), August (g), and September (h). Abbreviations: Ah (Ahmedabad), Dh (Dhanbad), Rl (Ruili), Bs (Baoshan), Hc (Hechi), and Sm (Sanming). In each box plot, the box represents the 20th–80th percentile range, whiskers extend to the 10th–90th percentiles, horizontal lines inside the boxes indicate the mean, and stars represent outliers. Horizontal arrows highlight the westward extension of the low $\delta^{18}O_P$ zone. Dotted lines mark the dividing line of the eastern and western domains. $\delta^{18}O_P$ values are expressed as per mil (‰) deviations relative to VSMOW or VSMOW2.

Additionally, we added a new schematic diagram figure to illustrate the mechanisms that spatial distributions of $\delta^{18}O_P$ along the transect during the pre-monsoon and monsoon seasons (Figure R10). Please see the new Fig. 6 in the revised manuscript.

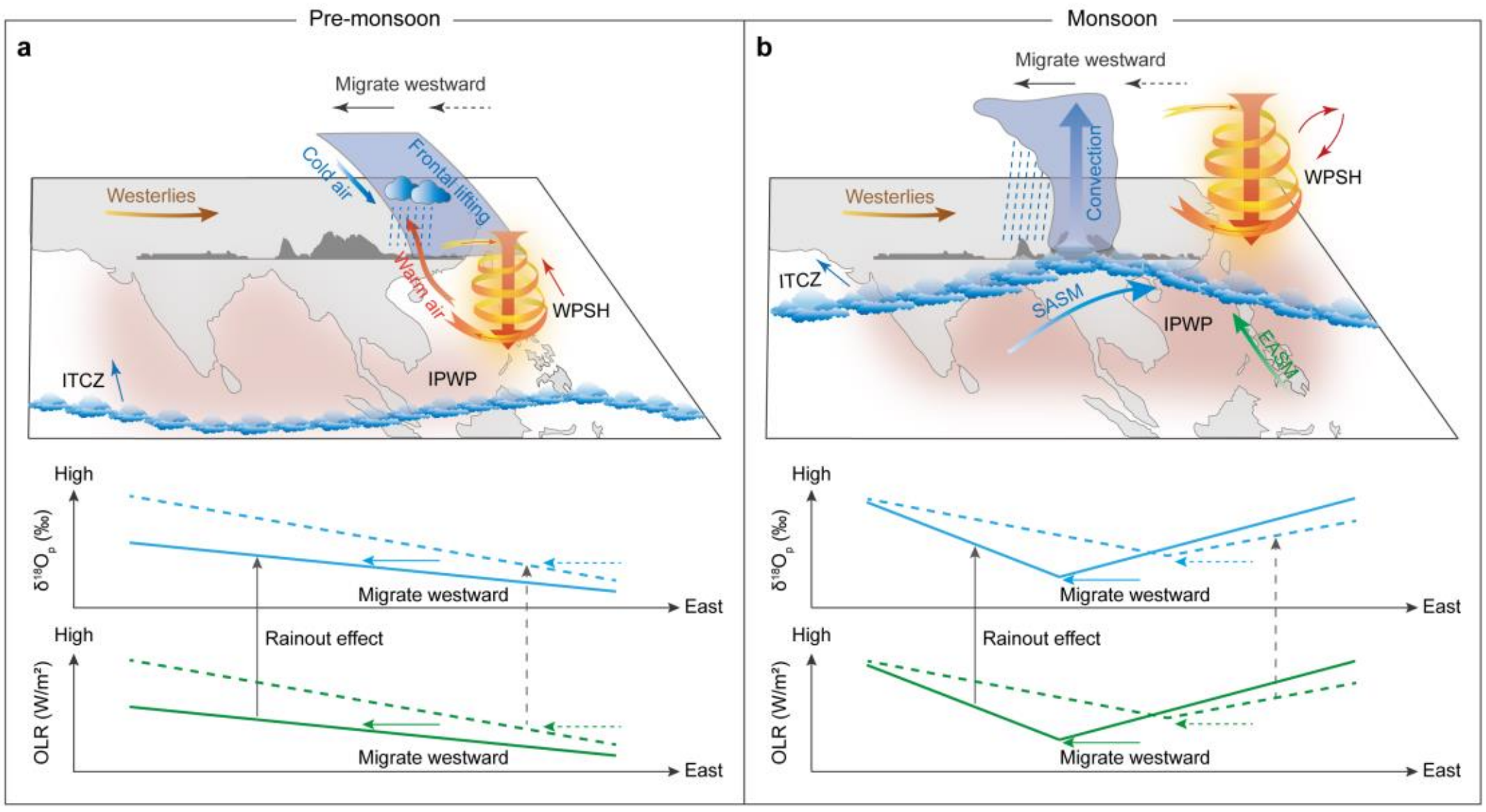

Figure R10. Schematic illustration of mechanisms controlling the spatial distributions of $\delta^{18}O_p$ along the transect during the pre-monsoon and monsoon seasons. a Pre-monsoon season. b Monsoon season. Horizontal solid arrows indicate the final state (May during the pre-monsoon season or September during the monsoon season) of stronger atmospheric updraft and lower $\delta^{18}O_p$ migrating westward, while horizontal dashed arrows represent the prior state (February during the pre-monsoon season or June during the monsoon season). The deep gray shading in the topographic map shows the terrain profile along the east–west transect. Abbreviations: ITCZ, Intertropical Convergence Zone; IPWP, Indo-Pacific Warm Pool; WPSH, Western Pacific subtropical high; SASM, South Asian summer monsoon; EASM, East Asian summer monsoon; OLR, outgoing longwave radiation. The blue and red arrows adjacent to ITCZ and WPSH indicate their movement directions during the pre-monsoon and monsoon seasons. Figure created with Adobe Illustrator 2024.

5. The extremely large number of supplementary figures. Having such a high number meant we were constantly switching back-and-forth between the manuscript and supplementary file, which made reading a lot more difficult. Moreover, we would argue that many of these figures did not add anything to the story and, in certain cases, obscured the key message of the paper.

Re: We thank you for pointing these issues out. We agree with you about many of these figures obscured the key message of the paper. Following your comments and suggestions, we carefully evaluated each figure in Supplementary Information and

found that many of them indeed did not add anything to the story. For example, regarding spatial distributions of simulated $\delta^{18}O_p$ across the study region over 1979–2022 during the pre-monsoon and monsoon seasons (Figs. S8–S10), they are of little significance because we have already confirmed that there is a good correlation between the simulated $\delta^{18}O_p$ data and the measured $\delta^{18}O_p$ data. The result indicates that simulated $\delta^{18}O_p$ data can capture the measured $\delta^{18}O_p$ data very well. Moreover, we have already demonstrated the westward expansion of the low-value zone of $\delta^{18}O_p$ along the transect by using the simulated $\delta^{18}O_p$ data at the 12 primary stations. It is obviously of little significance to further demonstrate the spatial changes with the simulated $\delta^{18}O_p$ data. Therefore, we removed Figs. S8–S10 from Supplementary Information. Moreover, due to that other figures are also redundant, we removed Fig. S20 and Fig. S23 from Supplementary Information. In addition, we moved Fig. S14 from the Supplementary Information to the main text, and changed the style of the figure and merge it into Fig. 3. In this case, we decreased the number of original figures in the Supplementary Information.

DETAILED COMMENTS

Line 51: It would be good to have an indication of the timescales being considered in this study here; given that different timescales will require consideration of different mechanisms, signals, and processes.

Re: We thank you for pointing this issue out. In fact, speleothem oxygen isotope records ($\delta^{18}O_s$) have been widely used to reconstruct paleoclimate (especially monsoon) on different timescales, including the centennial (Tan et al., 2015; Tan et al., 2017), millennial (Zhang et al., 2008; Tan et al., 2018), and orbital scales (Cai et al., 2015; Cheng et al., 2016; Liu et al., 2020; Wang et al., 2008). Following your comment, we changed "speleothem oxygen isotope records ($\delta^{18}O_s$) from the Asian monsoon region are crucial for reconstructing past climate changes" to "*Speleothem oxygen isotope records ($\delta^{18}O_s$) from the Asian monsoon region are crucial for reconstructing past climate variability across timescales.*" Please see L50–L51 in the revised manuscript.

References:

Cai, Y. et al. Variability of stalagmite-inferred Indian monsoon precipitation over the

past 252,000 y. *Proc. Natl. Acad. Sci. U.S.A.* **112**, 2954-2959 (2015).

Cheng, H. et al. The Asian monsoon over the past 640,000 years and ice age terminations. *Nature* **534**, 640–646 (2016).

Tan, L. et al. Centennial- to decadal-scale monsoon precipitation variations in the upper Hanjiang River region, China over the past 6650 years. *Earth Planet. Sci. Lett.* **482**, 58–66 (2018).

Tan, L. et al. Climate significance of speleothem $\delta^{18}O$ from central China on decadal timescale. *J. Asian Earth Sci.* **106**, 150–155 (2015).

Tan, L. et al. Decreasing monsoon precipitation in southwest China during the last 240 years associated with the warming of tropical ocean. *Clim. Dyn.* **48**, 1769–1778 (2017).

Wang, Y. et al. Millennial- and orbital-scale changes in the East Asian monsoon over the past 224,000 years. *Nature* **451**, 1090–1093 (2008).

Zhang, P. et al. A test of climate, sun, and culture relationships from an 1810-year Chinese cave record. *Science* **322**, 940–942 (2008).

Line 53: A new hypothesis for what? This sentence doesn't quite make sense in isolation, as it's not clear exactly what the "new" refers to. Is it (a) a new hypothesis for interpretation of speleothem $\delta^{18}O$ records, or (b) a new hypothesis for diverse expressions of monsoon intensity in speleothems? This should be made more clear.

Re: We thank you for pointing this issue out. It is a new hypothesis for interpretation of speleothem $\delta^{18}O$ records. Following your comment, we changed “we propose a new hypothesis based on…” to “*we propose a new hypothesis to explain Asian $\delta^{18}O_s$ records, based on….*” Please see L53 in the revised manuscript.

Line 54: Are 'sites' and 'caves' not the same thing?

Re: They are not the same thing. The “46 sites” refer to 46 precipitation sampling sites; however, the “13 caves” refer to the locations of the speleothem $\delta^{18}O$ records. In modern precipitation stable isotope studies, “site” or “station” is often used to indicate the location where precipitation samples were collected (outside the caves). However, in speleothem paleoclimatology studies, “cave” is often used to indicate the location where speleothem stable isotope records were obtained (inside the caves). The use of “site” and “cave” enables readers to easily understand what types of data they are

respectively.

Line 57: "isotope variations across the Asian monsoon region."

Re: Following your suggestion, we changed "isotope variations across Asian monsoon region" to "*isotope variations across the Asian monsoon region*." Please see L57–L58 in the revised manuscript.

Line 58: Authors say 'prolonged' - how long are we talking? Hours? Days? Weeks?

Re: We thank you for pointing this issue out. Here, "prolonged precipitation event" refers to the duration of a continuous precipitation event, which is generally several hours or even several days. Following your suggestion, we changed "prolonged" to "*continuous*." Please see L61 in the revised manuscript.

Line 59: If $\delta^{18}O$ signals are going to be differentiated using subscripts, there needs to be explicit definition of what these different subscripts refer to. Even in the abstract, and especially for a journal like NatComms where the readership is likely to be more broad, and not limited to subject specialists who would be familiar with this nomenclature.

Re: We thank you for pointing this issue out. We agree with you. Here, the $\delta^{18}O_p$ indicates precipitation $\delta^{18}O$. When the $\delta^{18}O_p$ first appeared in line 53, we provided its full name. The $\delta^{18}O_s$ indicates Speleothem $\delta^{18}O$, when the $\delta^{18}O_s$ first appeared in line 50, we provided its full name. We provided the full names when they appeared for the first time in Main Text, Methods, and Supplementary Information, respectively. Please see L50, L54, L663 and L670 in the revised manuscript. Please see L24 and L338 in the revised Supplementary Information.

Lines 65-66: This feels like an unsatisfactory end to the abstract, and excessively vague. As a reader, we want a clearer statement of what this 'new perspective' entails, how it is different to other perspectives that are currently available/proposed, and the implications of this revised appraisal of speleothem $\delta^{18}O$ records.

Re: We thank you for pointing this issue out. Our work challenges traditional views, and highlights that the signal preserved in Asian $\delta^{18}O_s$ records is atmospheric updraft ("vertical direction") rather than the monsoon ("horizontal direction"). Following

your comment, we changed "our hypothesis offers a new perspective on the interpretation of Asian $\delta^{18}O_s$ records" to "*Compared with the traditional interpretation focused on horizontal processes, our hypothesis offers a new perspective emphasizing vertical atmospheric dynamics*." Please see L65–L67 in the revised manuscript.

Line 73: 'Climate change' is a broad statement – more specificity is needed on which changes are of particular concern to this region. This will help to clearly set up why the work is novel, and valuable.

Re: We thank you for pointing this issue out. In this study, the "Climate change" includes precipitation and monsoon intensity. Following your comment, we changed "Climate change in this region has profoundly affected human livelihoods and development" to "*Climate change, especially precipitation and monsoon intensity has profound impacts on livelihoods and development*[3-5]." Please see L73–L74 in the revised manuscript.

Line 74: On what timescales?

Re: We thank you for pointing this issue out. It should be "on different timescales, including the decadal, centennial, millennial, and orbital scales." Given that the statement of "on different timescales, including the decadal, centennial, millennial, and orbital scales" is too verbose, we only added "*across timescales*" in the sentence. Please see L75 in the revised manuscript.

Lines 75-76: "However, long-term instrumental records … scarce." Please provide a reference.

Re: Following your suggestion, we provided two references including Cook et al. (2010) and Kathayat et al. (2017). Please see L76 in the revised manuscript.

References:

1. Cook, E. R. et al. Asian monsoon failure and megadrought during the last millennium. *Science* **328**, 486–489 (2010).

10. Kathayat, G. et al. The Indian monsoon variability and civilization changes in the Indian subcontinent. *Sci. adv.* **3**, e1701296 (2017).

Line 77: If $\delta^{18}O$ is going to be singled out, it needs to be made more clear on why this proxy is preferential over proxies such as trace elements, $\delta^{13}C$ etc... which can also provide valuable climatological information.

Re: We thank you for pointing this issue out. Following your suggestion, we added some lines to explain it. That is, we changed "Fortunately, numerous paleoclimate archives[10-13], particularly speleothem oxygen isotope ($\delta^{18}O_s$) records[14-20], serve as valuable indicators for reconstructing past climate conditions in the region" to "*Fortunately, numerous paleoclimate archives provide alternative means to reconstruct past climate*[11-14]*, particularly speleothems*[15-20]*. Among various proxies (trace elements, $\delta^{13}C$, etc.), speleothem oxygen isotope ($\delta^{18}O_s$) records are more valuable for two key reasons: (1) they offer high temporal resolution suitable for paleoclimate studies*[15-20]*; and (2) most importantly, their $\delta^{18}O_s$ records enable direct comparison with well-established ice core $\delta^{18}O$ records from the Antarctic or Greenland*[8,15,20]*.*" Please see L76–L82 in the revised manuscript.

Reference:

11. Gupta, P., Gupta, A. K., Clemens, S. C., Cheng, H. & Majhi, B. Coupled Ocean-atmospheric forcing on Indian Summer Monsoon variability during the middle Holocene: Insights from the Core Monsoon Zone speleothem record. *Palaeogeogr. Palaeocl.* **647**, 112273 (2024).
12. Thompson, L. G. et al. A high-resolution millennial record of the South Asian Monsoon from Himalayan ice cores. *Science* **289**, 1916–1920 (2000).
13. Zhang, H. et al. Collapse ofthe Liangzhu and other Neolithic cultures in the lower Yangtze region in response to climate change. *Sci. Adv.* **7**, eabi9275 (2021).
14. Xu, C. et al. Tree-ring oxygen isotope across monsoon Asia: common signal and local influence. *Quaternary Sci. Rev.* **269**, 107156 (2021).
15. Wang, Y. et al. A High-Resolution Absolute-Dated Late Pleistocene Monsoon Record from Hulu Cave, China. *Science* **294**, 2345 (2001).
16. Tan, L., Cai, Y., Cheng, H., An, Z. & Edwards, R. L. Summer monsoon precipitation variations in central China over the past 750 years derived from a high-resolution absolute-dated stalagmite. *Palaeogeogr Palaeocl.* **280**, 432–439 (2009).

17. Berkelhammer, M. et al. Persistent multidecadal power of the Indian Summer Monsoon. *Earth Planet. Sci. Lett.* **290**, 166–172 (2010).
18. Li, H. et al. High-resolution $\delta^{18}O$ and $\delta^{13}C$ records during the past 65 ka from Fengyu Cave in Guilin: variation of monsoonal climates in south China. *Quatern. Int.* **441**, 117–128 (2017).
19. Sinha, A. et al. Trends and oscillations in the Indian summer monsoon rainfall over the last two millennia. *Nat. Commun.* **6**, 6309 (2015).
20. Liu, G. et al. On the glacial-interglacial variability of the Asian monsoon in speleothem $\delta^{18}O$ records. *Sci. Adv.* **6**, eaay8189 (2020).

Lines 93-98: This paragraph is difficult to follow without a map or a more specific description of the locations within the Asian monsoon region. In addition, the authors consider a rather small area compared to the entire Asian monsoon region to argue for the uncertainties in the key controls on $\delta^{18}O_p$.

Re: We thank you for pointing this issue out. Following your suggestion, we added a more specific description of the locations within the Asian monsoon region. That is, we changed "the $\delta^{18}O_s$ record from Tham Doun Mai cave has a mean value of -9.84‰[47], while Dongge cave, located more than 4° to the north, has a higher mean value of -8.92‰[48] over the period 8874–7581 yr BP." to "*the $\delta^{18}O_s$ record from Tham Doun Mai cave (Luang Prabang Province, Northern Laos) has a mean value of -9.84‰[51], while Dongge cave (Guizhou Province, southwestern China), located more than 4° to the north, has a higher mean value of -8.92‰[52] over the period 8874–7581 yr BP.*" Please see L100–L104 in the revised manuscript.

We agree with you that "the authors consider a rather small area compared to the entire Asian monsoon region to argue for the uncertainties in the key controls on $\delta^{18}O_p$." However, here we merely take it as a contradiction to bring out the existing problems. In the main text we will further explore the factors influencing $\delta^{18}O_p$ on a larger spatial scale.

Line 98: Is Huanjiang a GNIP station as well?

Re: Huangjiang is not a GNIP station. So we cited a reference here (Zhang et al., 2023). Please see L106 in the revised manuscript.

Reference:

53. Zhang, J. et al. Temporal variations of precipitation driven by local meteorological parameters in southwest China: Insights from 9 years of continuous hydro-meteorological and isotope observations. *J. Hydrol. Reg. Stud.* **46**, 101345 (2023).

Lines 99-100: Could it be possible for controls to be different under changing background conditions, or one considers decadal versus millennial timescales? Is there strong evidence for a single control in isolation?

Re: We thank you for pointing these issues out. Under different background conditions, it is possible that the control factors are different. In the speleothem paleoclimate studies, some scientists indeed considered decadal versus millennial timescales, such as the studies of Liu et al. (2015) and Chen et al. (2016). It should be pointed out that speleothem $\delta^{18}O$ records last for thousands or even hundreds of thousands of years, and their climate signals have remained unclear because humans have been unable to go back to the past thousand or even hundreds of thousands of years to conduct observations. Therefore, the climate signals preserved in speleothem $\delta^{18}O$ records have remained a great controversy. Many scientists can only reveal the signals from the perspective of modern precipitation isotopes. During the past few decades, many scientists have been trying to find the dominant factor (or a single control in isolation) from the perspective of modern precipitation stable isotopes. This is precisely the motivation of our study.

References:

Chen, J. et al. On the timing of the East Asian summer monsoon maximum during the Holocene—Does the speleothem oxygen isotope record reflect monsoon rainfall variability? *Sci. China Earth Sci.* **59**, 2328–2338 (2016).

Liu, J. et al. Holocene East Asian summer monsoon records in northern China and their inconsistency with Chinese stalagmite $\delta^{18}O$ records. *Earth Sci. Rev.* **148**, 194–208 (2015).

Line 101: Spell out GNIP in full before abbreviating.

Re: Here, GNIP means "Global Network of Isotopes in Precipitation", and we have already provided the full name when it first appeared on Lines 97-98 in the previous manuscript. Please see L105 in the revised manuscript.

Line 102: Which "published sources" are the authors referring to? Please, provide the reference(s) for the published sources.

Re: We thank you for pointing this issue out. There are too many references for "published sources" to list them all here. For a clearer explanation, we split Table S1 into two tables (Table S1 and S2), listing the 12 primary sites and the 34 auxiliary sites respectively. We provided the references for the "published sources" in the two tables. Hence, we changed "we compiled $\delta^{18}O_p$ observations from GNIP and other published and unpublished sources, totaling 8,636 samples from 46 precipitation sites, as well as $\delta^{18}O_s$ data from 13 caves along an east–west transect of the Asian monsoon region (Fig. 1, Supplementary Tables 1 and 2, Materials and Methods)" to "*Here, we compiled $\delta^{18}O_p$ observations from GNIP and other published and unpublished sources, totaling 8,636 samples from 46 precipitation sites including 12 primary sites (Supplementary Table 1) and 34 ancillary sites (Supplementary Table 2), as well as $\delta^{18}O_s$ data from 13 caves (Supplementary Table 3) along an east–west transect of the Asian monsoon region (Fig. 1, see Materials and Methods for details).*" Please see L109–L113 in the revised manuscript.

Lines 103-104: We would like to see a more detailed description of the workflow used to compile the datasets used in this study. For example:

- How was data sourced?
- How did the authors ensure that suitable records were not missed/overlooked?
- Were any datasets omitted, and if so, why?

We have little doubt that the authors conducted a thorough review of the literature in order to compile their records, but we think being transparent on the process used is important so it is clear the data used hasn't been 'cherry-picked'

Re: We thank you for pointing these issues out. We have tried to address all the issues as follows.

1) With regard to "data source", we will add a paragraph to provide a detailed

explanation of our data source. i.e., "*We collected all available $\delta^{18}O_s$ records from three sources: (1) the SISALv3 database*[0]*; https://ora.ox.ac.uk/objects/uuid:1e91e2ac-ca9f-46e5-85f3-8d82d4d3cfd4); (2) speleothem datasets archived in the World Data Service for Paleoclimatology (NOAA/NCEI; https://www.ncdc.noaa.gov/data-access/paleoclimatology-data/datasets/speleothem); and (3) published studies.*" Please see L694–L698 in the revised manuscript.

2) With regard to whether the $\delta^{18}O_s$ records are missing, following your comments, we re-examined the database based on the selection criteria for $\delta^{18}O_p$ and $\delta^{18}O_s$ data as mentioned above. The results showed that, for $\delta^{18}O_p$ data, the number of sites and sample size remained unchanged. For $\delta^{18}O_s$ record, we found a new SISALv3 database compared with the SISALv2 version. Note that this new version was published in 2024 (i.e., after the work of our previous manuscript). Compared to the SISALv2 version that we cited, the SISALv3 database includes additional $\delta^{18}O_s$ records. Hence, we supplemented and adjusted the $\delta^{18}O_s$ records based on the SISALv3 database as follows:

a) For the Mawmluh cave: we added two $\delta^{18}O_s$ records including MWS-1 (Dutt et al., 2015) and ML.2 (Kathayat et al., 2018).

b) For the Tham Doun Mai cave: in the previous manuscript, we only used TM-17 (Wang et al., 2019) and TM-17 (Wood et al., 2023) $\delta^{18}O_s$ records. The former covers the period from -51 to 1879 yr, AD, and the latter covers the period from -6960.9 to -5624.6 yr, AD. In the SISALv3 database, we found that the TM5 $\delta^{18}O_s$ record from this cave covers the period from -7286 to 1267 yr, AD. Clearly, the duration of the TM-17 (Wood et al., 2023) $\delta^{18}O_s$ records was included in that of the TM5 $\delta^{18}O_s$ record. According to the selection criterion of "If a longer $\delta^{18}O_s$ record completely covers all other records in the same cave, we will select only this longest record", we used the TM5 record from this cave, and eliminated the TM-17 (Wood et al., 2023) $\delta^{18}O_s$ records.

c) For the Xiaobailong cave: we added the XBL-29 $\delta^{18}O_s$ record from this cave (Cai et al., 2015).

d) For the Wulu cave: we added two $\delta^{18}O_s$ records from this cave including Wu88

(Liu et al., 2023) and Wu37 (Zhao et al., 2020).

e) For the Dongge cave: in the previous manuscript, we used the D4 (Dykoski et al., 2005) and DA (Cheng et al., 2009) $\delta^{18}O_s$ records. The former covers the period from -13860 to 1966 yr, AD, and the latter covers the period from -6394 to -6040 yr, AD. Clearly, the period of the former included that of the latter. Hence, we retained the former and eliminated the latter.

It should be noted that the core objective of this study is to reveal the spatial patterns of $\delta^{18}O_s$ values along the transect rather than the temporal changes of $\delta^{18}O_s$ records. Hence, our study is different from traditional paleoclimate reconstruction studies. Therefore, we compiled all publicly available $\delta^{18}O_s$ data within the study region that met the selection criteria. Moreover, we found that the updated dataset does not affect our conclusions.

3) With regard to whether the data were "cherry-picked", we did not cherry-pick the data. However, following your comments, we rechecked the $\delta^{18}O_s$ database, and found that a new SISALv3 database was released in 2024. Hence, we updated the $\delta^{18}O_s$ dataset based on the SISALv3 database. The results remained consistent with our previous findings.

4) To help readers better understand the $\delta^{18}O_s$ records, including their identification, screening, and evaluation process. We added three paragraphs in the revised manuscript. Please see L693–L719 in the revised manuscript.

References:

84. Kaushal, N. et al. SISALv3: Speleothem Isotopes Synthesis and AnaLysis database version 3.0. University of Oxford (2024).

Cai, Y. et al. Variability of stalagmite-inferred Indian monsoon precipitation over the past 252,000 y. *Proc. Natl. Acad. Sci. U.S.A.* **112**, 2954-2959 (2015).

Cheng, H. et al. Timing and structure of the 8.2 kyr B.P. event inferred from $\delta^{18}O$ records of stalagmites from China, Oman, and Brazil. *Geology* **37**, 1007-1010 (2009).

Dutt, S. et al. Abrupt changes in Indian summer monsoon strength during 33,800 to 5500 years B.P. *Geophys. Res. Lett.* **42**, 5526–5532 (2015).

Griffiths, M. L. et al. End of Green Sahara amplified mid- to late Holocene megadroughts in mainland Southeast Asia. *Nat. Commun.* **11**, 4204 (2020).

Kathayat, G. et al., Evaluating the timing and structure of the 4.2 ka event in the Indian summer monsoon domain from an annually resolved speleothem record from Northeast India. *Clim. Past.* **14**, 1869-1879 (2018).

Liu, D., Mi, X., Liu, S. & Wang, Y. Multi-phased Asian hydroclimate variability during Heinrich Stadial 5. *Clim. Dyn.* **60**, 4003–4016 (2023).

Wang, J. et al. Hydroclimatic variability in Southeast Asia over the past two millennia. *Earth Planet. Sci. Lett.* **525**, 115737 (2019).

Wood, C. T. et al. High-resolution, multiproxy speleothem record of the 8.2 ka event from Mainland Southeast Asia. *Paleoceanogr. Paleoclimatol.* **38**, e2023PA004675 (2023).

Zhao, K. et al. Late Holocene monsoon precipitation changes in southern China and their linkage to Northern Hemisphere temperature. *Quaternary Sci. Rev.* **232**, 106191 (2020).

Lines 104-105: This statement assumes prior knowledge of this specific model, which we are not sure all readers will have. Useful here would be a sentence or two explaining why this model was chosen, and what it offers. Also, the authors state that they used the IsoGSM2 model. Which model was used for the study, IsoGSM or IsoGSM2? Please add the provider of the model or a reference.

Re: We thank you for pointing these issues out. In this study, we used the IsoGSM2 model, so we changed "IsoGSM" to "*IsoGSM2*" throughout the manuscript and Supplementary Information. Following your suggestion, when we first mentioned this model, we added one sentence to explain why this model was chosen, and what it offers, i.e., "*Additionally, we utilized $\delta^{18}O_p$ simulations from the IsoGSM2 model*[54] *to assess our results, because the model can reliably capture the signals of observed $\delta^{18}O_p$*[39] *(Supplementary Fig. 1).*" Additionally, we provided a reference. Please see L114–L116 in the revised manuscript.

References:

39. Chiang, J. C. H., Herman, M. J., Yoshimura, K., & Fung, I. Y. Enriched East Asian oxygen isotope of precipitation indicates reduced summer seasonality in regional

climate and westerlies. *Proc. Natl. Acad. Sci. U.S.A.* **117**, 14745–1475 (2020).
54. Yoshimura, K., Kanamitsu, M., Noone, D. & Oki T. Historical isotope simulation using Reanalysis atmospheric data. *J. Geophys. Res. Atmos.* **113**, D19108 (2008).

Lines 106-109: We like how the authors clearly lay out the discrete components of their study, although we think this could be written in a more sensical way. For example: "Our study had four key objectives: (1) to identify....,(2) to evaluate..." - and so on. This gives better opportunity for the reader to understand the 'why' behind this study. We could even go so far as to say that there is no need for more than two objectives….

Re: We thank you for pointing this issue out. We agree with you. In fact, we have only one key objective, that is, to determine the dominant factor governing $\delta^{18}O_p$ variations across the Asian monsoon region. To achieve this objective, we conduct the study in four steps. Following your suggestion, we changed "Our study follows four key steps: (1)… (2)… (3)… and (4)…" to "*We determine the dominant factor governing $\delta^{18}O_p$ variations across the Asian monsoon region through four key steps: (1)… (2)… (3)… and (4)….*" Please see L116–L121 in the revised manuscript.

Lines 112-113: What renders a site "primary" versus "ancillary"? How would the two be differentiated? The caption to Fig. 1 also refers to 34 ancillary precipitation sites. The abstract and the introduction refer to a total of 46 precipitation sites (12 primary + 34 ancillary). Are we right in assuming that 34 is the correct number?

Re: We thank you for pointing these issues out. We have tried to address all the issues as follows.

1) With regard to the number of ancillary sites, we are very sorry for the error in the text. The number of the auxiliary sites should be 34. Thank you very much for pointing out this mistake. We corrected it to 34. Please see L127 in the revised manuscript.

2）With regard to the classification criteria for primary site and auxiliary site, we identified 12 primary sites (Please see Table S1 in the Supplementary Information) and 34 auxiliary sites from the original 46 sites. Primary sites were selected based on the following criteria:

i) observation sites located within a narrow latitudinal band of 1°±1.5°;

ii) availability of high-precision (daily) $\delta^{18}O_p$ observations;

iii) maximum possible observation duration.

The remaining 34 sites served as ancillary sites to validate the spatial patterns of the primary sites (Table S2 in the Supplementary Information).

Following your suggestion, we added several sentences to provide more detailed explanations. i.e., "*To further reduce possible latitude effects on $\delta^{18}O_p$, we identified 12 primary sites (Supplementary Table 1) within the 46 sites, based on the following criteria: (1) observation sites located within a narrow latitudinal band of 1°±1.5°; (2) availability of high-precision (daily) $\delta^{18}O_p$ observations; and (3) maximum possible observation duration. The remaining 34 sites served as ancillary sites to validate the spatial patterns of the primary sites (Supplementary Table 2).*" Please see L687–L692 in the revised manuscript.

Line 114: What time period do all locations cover? We suggest including this information in the text or at least making a reference to Table S1.

Re: We thank you for pointing this issue out. The overall time period of the $\delta^{18}O_p$ data covers 1979–2022, but the time period of $\delta^{18}O_p$ data at each site is different. The shortest time period is only 4 months, such as Bhopal. The longest time period is 1979–2022, such as the Hong Kong site. All such information has been provided in Supplementary Tables 1 and 2. Following your suggestion, in the text we made a reference to Supplementary Tables 1 and 2. Please see L127–L128 in the revised manuscript.

Lines 116-117: What is this distinction based on? An existing study? Instrumental data?

Re: We thank you for pointing this issue out. With regard to the definition of the dividing line, in the previous manuscript, we intended to use the seasonal variations of $\delta^{18}O_p$ (observed data) along the transect as the criterion for regional division, because that we observed a significant shift in the seasonal variations of $\delta^{18}O_p$ around ~98°E: West of ~98°E, the multi-year monthly mean $\delta^{18}O_p$ values exhibited a continuous decrease from May to September/October; East of ~98°E, the multi-year monthly

mean $\delta^{18}O_p$ values showed a continuous decrease from May to July/August, followed by an increase.

Following your comments, we carefully checked the published work, and found that some scientists including Jin & Chen (1982) and Wang & LinHo (2002) had already demonstrated that ~98°E is the dividing line between the South Asian monsoon and the East Asian monsoon from the perspective of atmospheric dynamics. Thus, the dividing line revealed by the $\delta^{18}O_p$ data in our study essentially serve as another evidence for the conclusion. As mentioned above, we rewrote this section. i.e., "*Previous studies have shown that the eastern and western domains of the transect correspond to the East Asian monsoon region and South Asian monsoon region, respectively, with ~98° E serving as the dividing line*[55,56]*. In the eastern and western domains, we examined seasonal $\delta^{18}O_p$ variations at each site using multi-year mean monthly values from 12 primary sites (Supplementary Table 1) and 34 ancillary sites (Supplementary Table 2) along the east–west transect of the Asian monsoon region (20–27° N, 70–125° E). The results show the duration of lower $\delta^{18}O_p$ values is about two months shorter in the east than in the west (Fig. 1b, c and Supplementary Fig. 2). For the vast majority of sites in the eastern domain, the $\delta^{18}O_p$ continuously decreases from May to July/August, followed by a subsequent increase in August/September (Fig. 1b and Supplementary Fig. 2a). In contrast, in the western domain, $\delta^{18}O_p$ continues to decline from May through October (Fig. 1c and Supplementary Fig. 2b). The IsoGSM2–simulated $\delta^{18}O_p$ data from the 12 primary sites (1979–2022) further confirm these regional differences (Supplementary Fig. 3), reinforcing the distinction between the East and South Asian monsoon regimes.*" Please see L123–L137 in the revised manuscript. In this case, we first cited the previous study regarding ~98°E as the dividing line of the Asian monsoon region, and then analyzed the seasonal variation of $\delta^{18}O_p$ in the eastern and western domains and ultimately confirmed the significant differences in seasonal variation of $\delta^{18}O_p$ between the eastern and western domains.

Note that the dividing line was set at 100°E in our previous manuscript. However, in the revised manuscript we found that the precise location of the dividing line should be the region between Ruili (24.01°N, 97.85°E) and Tengchong (25.01°N, 98.3°E), based on the seasonal variation of $\delta^{18}O_p$ at the observed sites along the transect.

Hence, we updated the dividing line (around 98°E). Moreover, this updated dividing line aligns with that of Jin and Chen (1982) and Wang & LinHo (2002).

Reference:

55. Jin, Z. & Chen, L. On the medium-range oscillation of the East Asia monsoon circulation system and its relation with the Indian monsoon system. *In The National Symposium Collections on the Tropical Summer Monsoon* 204-215 (People's Press Yunnan Province, 1982).

56. Wang, B., & LinHo, Rainy Season of the Asian–Pacific Summer Monsoon. *J. Climate* **15**, 386–398 (2002).

Line 118: Or "eastern domain"?

Re: Thank you very much for your suggestion. We changed "eastern part" to "*eastern domain*." Correspondingly, we changed "western part" to "*western domain*." Please see L131 and L133 in the revised manuscript.

Line 120: Which $\delta^{18}O_p$ values are shown in Supplementary Fig. 2 (Mean, Median)? In addition, Supplementary Fig. 2 refers to East Asian monsoon and South Asian monsoon instead of "eastern part" (L. 118) and "western part"(L. 120). Please be more consistent in using technical terms.

Re: We thank you for pointing these issues out. $\delta^{18}O_p$ values are shown in Supplementary Fig. 2 refer to the multi-year mean $\delta^{18}O_p$. Following your comments, we added "multi-year mean" to the caption of Supplementary Fig. 2. i.e., we changed "Seasonal variations of observed $\delta^{18}O_p$ at the 46 precipitation sites across the Asian monsoon region" to "*Seasonal variations in observed multi-year mean $\delta^{18}O_p$ at 46 precipitation sites across the Asian monsoon region*." Please see L85–L86 in the revised Supplementary Information.

Following your suggestions, in Supplementary Fig. 2, we changed "East Asian monsoon region" and "South Asian monsoon region" to "*eastern domain*" and "*western domain*", respectively. Please see L86–L87 in the revised Supplementary Information.

Lines 124-126: This is a circular point: if the line between east and west regions has already been defined as 100°east, then the results don't "indicate" the difference, they simply support a pre-defined idea that the east and west are seasonally different. Thus, the source of this hypothesis should be clearly specified. Also, what would happen if you shifted this band? Would the same clear difference still exist? Also, we note that in line 116 the authors are referring to eastern and western parts of the region, when talking about the 100°E dividing line. Yet here (124), the authors are referring to the East Asian monsoon and South Asian monsoon region. Please be more consistent.

Re: We thank you for pointing these issues out. We have tried to address all the issues as follows.

1) Note that the dividing line was set at 100°E in our previous manuscript. However, in the revised manuscript we found that the precise location of the dividing line should be the region between Ruili (24.01°N, 97.85°E) and Tengchong (25.01°N, 98.3°E), based on the seasonal variation of $\delta^{18}O_p$ at the observed sites along the transect. Hence, we updated the dividing line (around 98°E). Moreover, this updated dividing line aligns with that of Jin and Chen (1982) and Wang & LinHo (2002).

2) We agree with you about the dividing line between eastern and western domains. In the previous manuscript, we intended to use the seasonal variations of $\delta^{18}O_p$ (observed data) along the transect as the criterion for regional division, because that we observed a significant shift in the seasonal variations of $\delta^{18}O_p$ around ~98°E: West of ~98°E, the multi-year monthly mean $\delta^{18}O_p$ values exhibited a continuous decrease from May to September/October; East of ~98°E, the multi-year monthly mean $\delta^{18}O_p$ values showed a continuous decrease from May to July/August, followed by an increase.

Following your comments, we carefully checked the published work, and found that some scientists including Jin & Chen (1982) and Wang & LinHo (2002) had already demonstrated that ~98°E is the dividing line between the South Asian monsoon and the East Asian monsoon from the perspective of atmospheric dynamics. Thus, the dividing line revealed by the $\delta^{18}O_p$ data in our study essentially serve as another evidence for the conclusion. As mentioned above, we rewrote this section. That is, we first cited the previous study regarding ~98°E as the dividing line of the Asian monsoon region, and then analyzed the seasonal variation of $\delta^{18}O_p$ in the eastern and western domains and ultimately confirmed the significant differences in seasonal

variation of $\delta^{18}O_p$ between the eastern and western domains. Please see L123–L137 in the revised manuscript.

3) With regard to what would occur if this dividing line were shifted, following your comment, we have a try to shift this dividing line, and found that the clear spatial patterns would be disrupted, and that the dividing line would not appear.

4) Following your suggestion, we changed "East Asian monsoon region" and "South Asian monsoon region" to "*eastern domain*" and "*western domain*", respectively. Please see L131 and L133 in the revised manuscript.

Line 128: Again, it needs to be made much clearer how well defined this distinction is already, and thus how much new knowledge this study is contributiing to this idea. If this phenomenon is already well established/characterized, what is the purpose of re-examining it here? We don't doubt it was done for a reason, but this reason is not clear from the text as-written.

Re: We thank you for pointing this issue out. As mentioned above, in the previous manuscript, we intended to use the seasonal variations of $\delta^{18}O_p$ (observed data) along the transect as the criterion for regional division, because that we observed a significant shift in the seasonal variations of $\delta^{18}O_p$ around ~98°E: West of ~98°E, the multi-year monthly mean $\delta^{18}O_p$ values exhibited a continuous decrease from May to September/October; East of ~98°E, the multi-year monthly mean $\delta^{18}O_p$ values showed a continuous decrease from May to July/August, followed by an increase.

Following your comments, we carefully checked the published work, and found that some scientists including Jin & Chen (1982) and Wang & LinHo (2002) had already demonstrated that ~98°E is the dividing line between the South Asian monsoon and the East Asian monsoon from the perspective of atmospheric dynamics. Thus, the dividing line revealed by the $\delta^{18}O_p$ data in our study essentially serve as another evidence for the conclusion. As mentioned above, we rewrote this section. That is, we first cited the previous study regarding ~98°E as the dividing line of the Asian monsoon region, and then analyzed the seasonal variation of $\delta^{18}O_p$ in the eastern and western domains and ultimately confirmed the significant differences in seasonal variation of $\delta^{18}O_p$ between the eastern and western domains. Please see L123–L137 in the revised manuscript.

Lines 130-131: Hasn't this already been done in the previous section? Perhaps these two sections could be merged, to improve the narrative flow.

Re: We thank you for pointing this issue out. Following your suggestion, we combined these two sections into the section "Seasonal variations and spatial distributions of $\delta^{18}O_p$." Please see L122–L163 in the revised manuscript.

Lines 134-135: Are these definitions based on established literature?

Re: We thank you for pointing this issue out. These definitions are not based on established literature. We roughly classified the samplings sites based on altitude. Following your comment, we reclassified the samplings sites based on the definitions of UNEP-WCMC criteria.

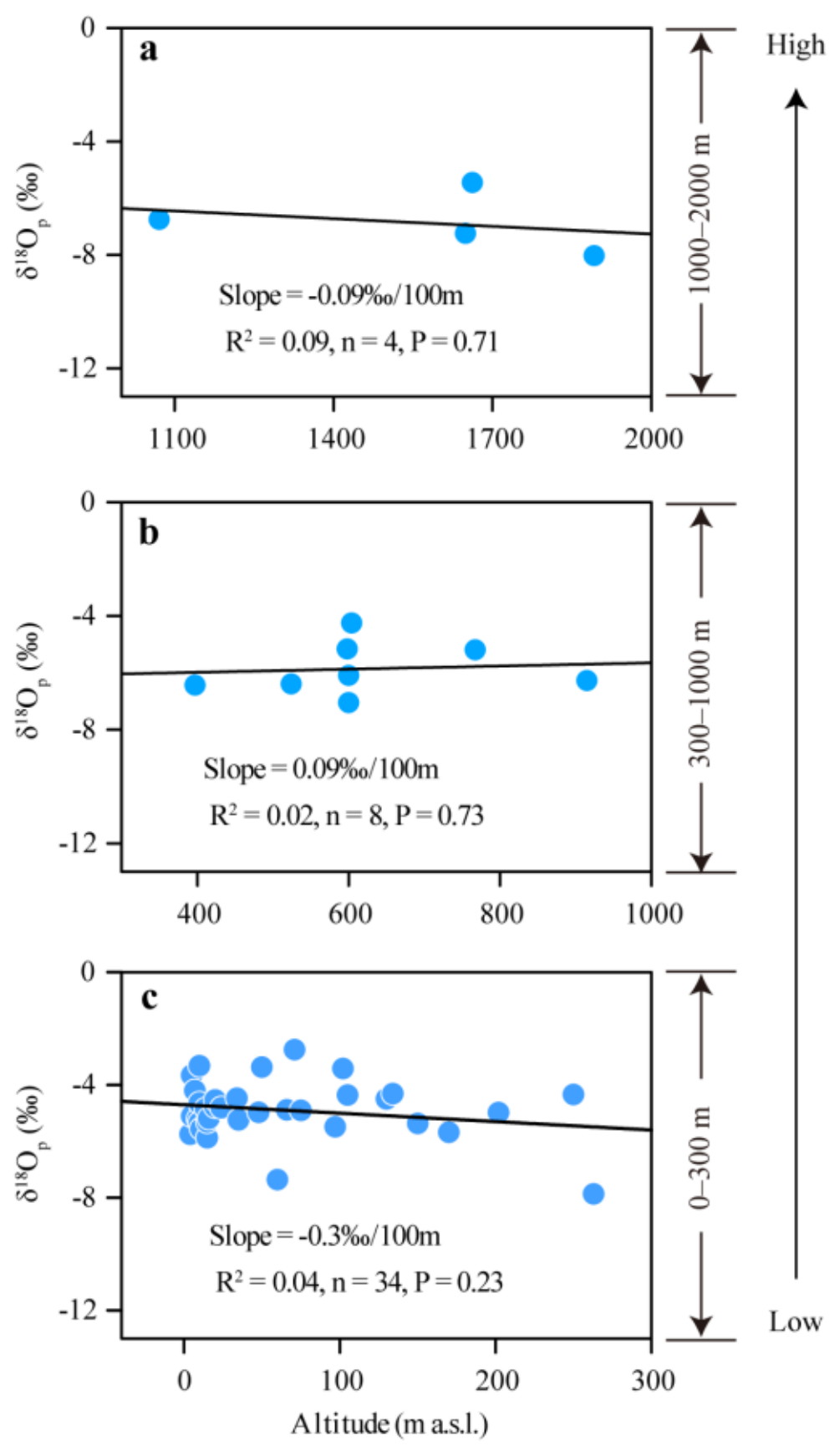

Figure R11. Correlations between observed $\delta^{18}O_p$ values and altitudes. The 46 precipitation sites were categorized into three altitude ranges based on the UNEP-WCMC mountain classification[2]. a–c, Correlations for the altitude ranges 1000–2000 m (a), 300–1000 m (b), and 0–300 m (c). $\delta^{18}O_p$ values are expressed as per mil (‰) deviations relative to VSMOW or VSMOW2.

Please see L309–L311 in the revised manuscript and the updated Fig. S14 in the revised Supplementary Information.

Reference:

UNEP World Conservation Monitoring Centre. *Mountain Watch: Environmental Change and Sustainable Development in Mountains*. Technical Report. Cambridge, UK: UNEP-WCMC, 2002.

Line 136: What correlation test is this referring to? (e.g., Pearson's, Spearman's...) Also, please give the statistical value.

Re: We thank you for pointing this issue out. In our study, all the correlation tests are Pearson's. Following your comment, we added one sentence to make clear it in the Materials and Methods section, i.e., "*Note in this study, all the correlation tests are Pearson's*." Please see L726 in the revised manuscript.

It should be noted that in the revised manuscript, we added a paragraph to specifically discuss the impact of altitude effects on $\delta^{18}O_p$ over time. To highlight key findings, we reduced the discussion of altitude effects based on altitude ranges and combined it with the content discussing the influence of altitude effects based on climate zones. That is, we rewrote and shortened this paragraph. For better narrative flow, we only present the statistical values in Supplementary Fig. S13 and Fig. S14. Please see Fig. S13 and Fig. S14 in the revised Supplementary Information.

Line 138: "Additionally, previous studies…" Which studies? Please add reference.

Re: Following your suggestion, we added some references including Jiao et al. (2019), Jiao et al. (2020) and Kong et al. (2023). Please see L312 in the revised manuscript.

References

72. Jiao, Y. et al. Impacts of moisture sources on the isotopic inverse altitude effect and amount of precipitation in the Hani Rice Terraces region of the Ailao Mountains. *Sci. Total Environ.* **687**, 470–478 (2019).

73. Jiao, Y., Liu, C., Liu, Z., Ding, Y. & Xu, Q. Impacts of moisture sources on the temporal and spatial heterogeneity of monsoon precipitation isotopic altitude

effects. *J. Hydrol.* **583**, 124576 (2020).

74. Kong, Y. et al. Ambiguity in the altitude effect of precipitation isotopes for estimating groundwater recharge elevation and paleoelevation reconstruction in the leeward side of a mountain. *Hydrogeol. J.* **31**, 1259–1270 (2023).

Lines 143-144: Not sure this point has been sufficiently "fleshed out". At the very least, we would like to see a couple of sentences where the authors consider why some records show a negative relationship, and some show a positive. Even if there is no conclusive answer, this should still be considered.

Re: We thank you for pointing this issue out. The definition of the altitude effect is that during the process of water vapor climbing up mountains and forming precipitation, it follows the Rayleigh fractionation. Heavy isotopes preferentially drop, resulting in the depletion of heavy isotopes in the residual water vapor. The residual water vapor continues to climb, and as the temperature decreases, the heavy isotopes in the subsequent precipitation become lower. Therefore, the $\delta^{18}O_p$ gradually decreases with increasing altitude. That is to say, there is a negative correlation between $\delta^{18}O_p$ and altitude. In other words, in our study, if there is a significant negative correlation between $\delta^{18}O_p$ and altitude, it indicates that the $\delta^{18}O_p$ values are affected by the altitude effect. It should be noted that we need to eliminate the influence of the altitude effect, which means we need to ensure that there is no significant negative correlation or even an abnormal positive correlation between $\delta^{18}O_p$ and altitude. This is what is called the "inverse altitude effect." Therefore, we added a sentence in the manuscript to explain why some records show a negative relationship, i.e., "*To test for an "altitude effect" where $\delta^{18}O_p$ decrease with increasing altitude due to lower condensation temperature*[69]." Please see L303–L304 in the revised manuscript.

As for why there might be an abnormal positive correlation between $\delta^{18}O_p$ and altitude, this might be related to the fact that the $\delta^{18}O_p$ are affected by other abnormal factors. For example, $\delta^{18}O_p$ on the Ainao Mountains (23.22° N, 102.84° E) increases with altitude[72,73], due to complex moisture sources, and $\delta^{18}O_p$ on the leeward side of the Meili Snow Mountains (28.46° N, 98.77° E) shows similar pattern[74], due to below-cloud evaporation and recycled moisture mixing. These results indicate altitude

does not significantly control $\delta^{18}O_p$ distribution. It can be found that the deviation from the classical altitude effect is likely attributed to some abnormal factors including complex moisture sources (mixing of multiple moisture sources or dominance of locally recycled moisture, Jiao et al., 2019, 2020), below-cloud evaporation, and mixing of recycled moisture (Kong et al., 2023).

It should be noted that, a detailed discussion on the specific reasons for the positive correlation between $\delta^{18}O_p$ and altitude would go beyond the scope of this study, and it would significantly increase the length of the paper (we would require a separate paper to discuss the mechanism in-depth). Hence, in this study we only prove that the $\delta^{18}O_p$ along this transect are not affected by the altitude effect. Certainly, we cited some references (e.g., Jiao et al., 2019; Jiao et al., 2020; Kong et al., 2023) about the positive correlation between $\delta^{18}O_p$ and altitude, and added some sentences simply explained the reasons ("due to complex moisture sources,…" and "because of below-cloud evaporation and mixing of recycled moisture"). Please see L312–L315 in the revised manuscript.

References:

69. Dansgaard, W. Stable isotopes in precipitation. *Tellus* **16**, 436–468 (1964).

72. Jiao, Y. et al. Impacts of moisture sources on the isotopic inverse altitude effect and amount of precipitation in the Hani Rice Terraces region of the Ailao Mountains. *Sci. Total Environ.* **687**, 470–478 (2019).

73. Jiao, Y., Liu, C., Liu, Z., Ding, Y. & Xu, Q. Impacts of moisture sources on the temporal and spatial heterogeneity of monsoon precipitation isotopic altitude effects. *J. Hydrol.* **583**, 124576 (2020).

74. Kong, Y. et al. Ambiguity in the altitude effect of precipitation isotopes for estimating groundwater recharge elevation and paleoelevation reconstruction in the leeward side of a mountain. *Hydrogeol. J.* **31**, 1259–1270 (2023).

Lines 145-147: Why? Useful here would be a clear link back to the research objectives set out at the beginning, and description of the key characteristics of the pre- and intra-monsoon climate system over East Asia.

Re: We thank you for pointing this issue out. Following your comments, we added a

description of the key characteristics of the climate system in the Asian monsoon region during the pre-monsoon, monsoon, and post-monsoon seasons, i.e., we changed "We now focus on the spatial distribution of multiyear mean monthly $\delta^{18}O_p$ values at the 12 primary sites during the pre-monsoon (Fig.2a-d) and monsoon (Fig.2e-h) seasons. The post-monsoon (October-January) contributes minimally to annual precipitation (Supplementary Fig.5) and is therefore not considered further (Supplementary Fig.6)" to "*We now focus on the spatial distribution of multi-year mean monthly $\delta^{18}O_p$ values from the eastern to western domains for each month (Fig. 2). Given that circulation fields and precipitation amount in the Asian monsoon region exhibit distinct seasonal variations*[50]*, we analyzed the spatial distribution of $\delta^{18}O_p$ values at the 12 primary sites during the pre-monsoon (February–May) (Fig. 2a-d), monsoon (June–September) (Fig. 2e-h), and post-monsoon (October–January) seasons (Supplementary Fig. 4). The post-monsoon season contributes minimally to annual precipitation (Supplementary Figs. 3 and 5) and is therefore not considered further (Supplementary Fig. 4).*" Please see L138–L146 in the revised manuscript.

Reference:
50. Zhang, J. et al. Coupled effects of moisture transport pathway and convection on stable isotopes in precipitation across the East Asian monsoon region: Implications for paleoclimate reconstruction. *J. Climate* **34**, 9811–9822 (2021).

Line 154: Is Guilin one of the 12 primary precipitation sites. There is no reference in the previous text or Figure 1. Where is Guilin located in the map (Fig. 1). Why is Tengchong in brackets? According to Figure 1, Tengchong and Kunming are two different sites about 430km apart.

Re: We are very sorry for making a mistake here. The "Guilin" should be "Institute of Karst Geology." The Institute of Karst Geology is one of the 12 primary precipitation sites. Thank you very much for pointing out this mistake. Following your comment, we changed "Guilin" to "*Institute of Karst Geology.*" Please see L151 in the revised manuscript.

We want to propose that in the East Asian monsoon region, the seasonal low of $\delta^{18}O_p$ values migrates westward from February to May. Initially appearing at Luoyuan and

Sanming (two easternmost sites) in February, it gradually migrates westward to Institute of Karst Geology, Hechi, and finally Kunming-Baoshan-Tengchong by May.

Given that our previous writing (including Tengchong in brackets) was misleading to the readers, we changed "Kunming (Tengchong)" to "*Kunming-Baoshan-Tengchong*." Please see L152 in the revised manuscript.

Line 155: We suggest improving Figure 2 by making it clearer for which precipitation sites the authors are referring to the eastern or western region.

Re: We thank you for pointing this issue out. Following your suggestions, we modified Figure 2 by adding some dotted lines between the sites of Ruili and Tenchong to help readers better understand whether the sampling sites we are referring to the eastern or western domains. In addition, we also added some horizontal arrows in the figure to enable readers to have a clearer understanding of the low $\delta^{18}O_p$ zone extending westward (as follows). The updated figure (Fig. 2) can be seen in the revised manuscript.

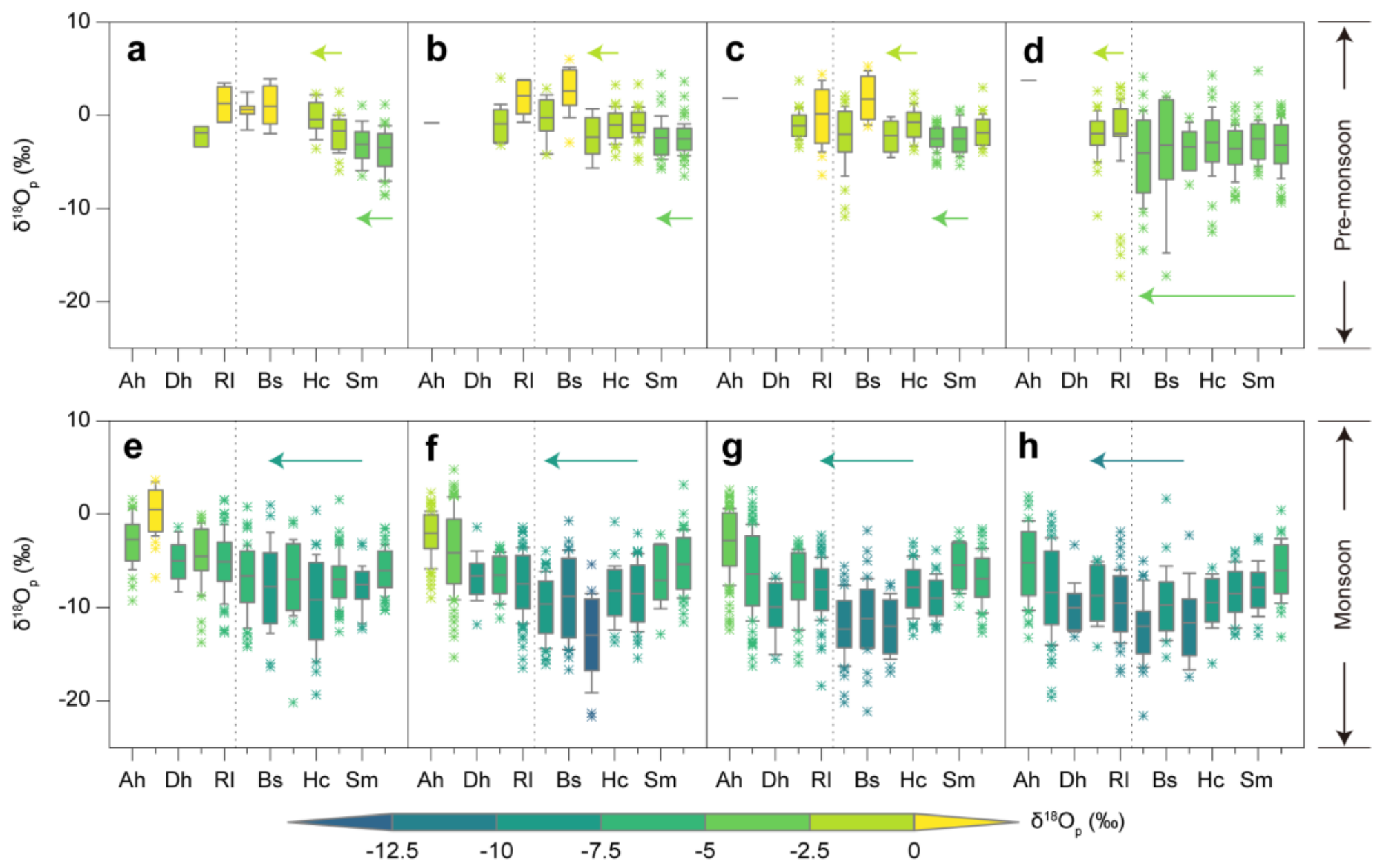

Figure R12. Spatial distributions of $\delta^{18}O_p$ at the 12 primary sites along the transect during the pre-monsoon and monsoon seasons. a–d $\delta^{18}O_p$ distributions during the pre-monsoon season: February (a), March (b), April (c), and May (d). e–h $\delta^{18}O_p$ distributions during the monsoon season: June (e), July (f), August (g), and September (h). Abbreviations: Ah (Ahmedabad), Dh (Dhanbad), Rl (Ruili), Bs (Baoshan), Hc (Hechi), and Sm (Sanming). In each box plot, the box represents the 20th–80th

percentile range, whiskers extend to the 10th–90th percentiles, horizontal lines inside the boxes indicate the mean, and stars represent outliers. Horizontal arrows highlight the westward extension of the low $\delta^{18}O_p$ zone. Dotted lines mark the dividing line of the eastern and western domains. $\delta^{18}O_p$ values are expressed as per mil (‰) deviations relative to VSMOW or VSMOW2.

Line 157: “middle”; Please be more specific.

Re: We thank you for pointing this issue out. Here, “middle” refers to several sites (Baoshan, Tengchong, Ruili, and Sylhet) around the dividing line of the South Asian monsoon region and the East Asian monsoon region. Following your suggestion, we changed “with the low $\delta^{18}O_p$ zone extending further westward to the middle of the transect from June to September (Fig. 2e-h). While $\delta^{18}O_p$ values in the east gradually increase over time, those in the west remain relatively high” to “*with the lower $\delta^{18}O_p$ zone extending westward to Baoshan and Tengchong, and even to the west of the dividing line (Ruili and Sylhet) from June to September (Fig. 2e-h). However, $\delta^{18}O_p$ values in the westernmost sites and the easternmost sites remain relatively high compared to those in the middle of the transect (around the dividing line).*” Please see L154–L158 in the revised manuscript.

Lines 159-161: We cannot fully agree with the observation made by the authors here. Unfortunately, the data for Figure 2 are not included in the source file for a more detailed examination of the spatial evolution of $\delta^{18}O_p$. However, just by assessing and comparing Figure 2e-h, it appears that $\delta^{18}O_p$ values in both the east and west remain similar over time. The strongest change in $\delta^{18}O_p$ values during the monsoon period occurs in the middle of the transect around the dividing line of ~100°E ± 5°rather than at the margins. We cannot see a gradual increase in $\delta^{18}O_p$ values in the eastern part of the transect, the oscillation of the values does not allow a clear statement about the trend over time. The mean of $\delta^{18}O_p$ values for the eastern sites are almost identical in July and September. The IsoGSM-simulated $\delta^{18}O_p$ data (Supplementary Fig. 7) also shows the strongest change in $\delta^{18}O_p$ values in the middle of the transect, while the western and eastern regions stay fairly stable over time.

Re: We thank you for pointing these issues out. Following your comments, we addressed these issues as follows:

1) With regard to source data of the figures, following your comments and editor's requirements, we provided source data for the all figures in our study, so that readers can conduct more detailed examination of the spatial pattern of $\delta^{18}O_p$.

2) With regard to the statement of "a gradual increase in $\delta^{18}O_p$ values in the eastern part of the transect", we agree with you. We carefully re-examined the data and found that the relevant expressions here are incorrect. Indeed, most eastern sites show no significant increasing trend in $\delta^{18}O_p$ values from June to September. Following your suggestion, we removed the statement "While $\delta^{18}O_p$ values in the east gradually increase over time, those in the west remain relatively high." Please see L153–L160 in the revised manuscript.

3) With regard to the comment of "the strongest change in $\delta^{18}O_p$ values during the monsoon period occurs in the middle of the transect around the dividing line of ~100°E ± 5° rather than at the margins", we agree with you, as lower $\delta^{18}O_p$ zone extends westward to the west of the dividing line (Ruili and Sylhet) rather than the westernmost sites (Ahmedabad and Sagar). Following your comments, we changed "During the monsoon season, $\delta^{18}O_p$ values are lower than during the pre-monsoon season, with the low $\delta^{18}O_p$ zone extending further westward to the middle of the transect from June to September (Fig. 2e-h). While $\delta^{18}O_p$ values in the east gradually increase over time, those in the west remain relatively high" to "*During the monsoon season, $\delta^{18}O_p$ values are lower than during the pre-monsoon season, with the lower $\delta^{18}O_p$ zone extending westward to Baoshan and Tengchong, and even to the west of the dividing line (Ruili and Sylhet) from June to September (Fig. 2e-h). However, $\delta^{18}O_p$ values in the westernmost sites and the easternmost sites remain relatively high compared to those in the middle of the transect (around the dividing line).*" Please see L153–L158 in the revised manuscript.

Lines 162-167: We are not entirely clear still how clearly this passage relates links back to the primary focus of this study, and so sentence or two summarising what these findings mean, and why they are important, would be really helpful.

Re: We thank you for pointing this issue out. We used IsoGSM2 model simulations to replicate the observational results from site data. That is, the model simulation data further support our findings. Following your suggestion, we added the term "*further*"

to emphasize it. Please see L161 in the revised manuscript.

Furthermore, following your comments, we added an explanation for using IsoGSM2 model data when first introducing this model in the Introduction section, i.e., "*Additionally, we utilized $\delta^{18}O_p$ simulations from the IsoGSM2 model*[54] *to assess our results, because the model can reliably capture the signals of observed $\delta^{18}O_p$*[39] *(Supplementary Fig. 1).*" Please see L114–L116 in the revised manuscript.

References:

39. Chiang, J. C. H., Herman, M. J., Yoshimura, K., & Fung, I. Y. Enriched East Asian oxygen isotope of precipitation indicates reduced summer seasonality in regional climate and westerlies. Proc. Natl. Acad. Sci. U.S.A. 117, 14745–1475 (2020).
54. Yoshimura, K., Kanamitsu, M., Noone, D. & Oki T. Historical isotope simulation using Reanalysis atmospheric data. J. Geophys. Res. Atmos. 113, D19108 (2008).

Line 168: This section seems to lack a coherent narrative flow, and as a result is hard to follow.

Re: We thank you for pointing this issue out. We agree with you that this section lacked a coherent narrative flow. Following your suggestions, we improved this section via a two-step process. These steps are (1) we merged the original first and second paragraphs as the new first paragraph, and (2) we restructured this section to enhance its logical coherence.

In this case, this section would have a coherent narrative flow as follows:

First paragraph: Fundamental understanding

Through analyzing the moisture field (Supplementary Fig. 7) and published work (Baker et al., 2015; Zhang et al., 2021; He et al., 2021), we established the fundamental understanding that the water vapor in the Asian monsoon region (including both the South Asian monsoon region and East Asian monsoon region) mainly originates from the Indian Ocean. These results explain why previous studies have linked the $\delta^{18}O_p$ variations in the South Asian and East Asian monsoon regions

to the monsoon water vapor transport from the Indian Ocean (Pausata et al., 2011; Baker et al., 2015; Zhang et al., 2021). However, we argued that this traditional understanding was subsequently challenged by the "decoupling" phenomenon observed in the temporal and spatial dimensions.

Second paragraph: Decoupling in the temporal dimension

It was found that there is a temporal difference between the continuous decline in $\delta^{18}O_p$ (May–October) in the South Asian monsoon region and the peak monsoon moisture transport (July–August) (Fig. 1c and Supplementary Figs. 2b and 7f, g). This suggests that the intensity of the South Asian summer monsoon alone cannot fully explain the seasonal variation of $\delta^{18}O_p$.

Third paragraph: Decoupling in the spatial dimension

During the monsoon period, if $\delta^{18}O_p$ in the East Asian monsoon region was primarily controlled by South Asian monsoon moisture advection, we would expect $\delta^{18}O_p$ values to decrease along the South Asian monsoon moisture channel due to a progressive "rainout effect."[48-50,57] This would result in a spatial trend where $\delta^{18}O_p$ decreases from Ruili-Tengchong (the middle of the transect) to Luoyuan (easternmost site), similar to the pre-monsoon seasonal pattern (Fig. 2a-d and Supplementary Fig. 6a-d). However, observations contradict this expectation: $\delta^{18}O_p$ values increase rather than decrease from the middle of the transect to the easternmost sites during each monsoon season month (June–September) (Fig. 2e-h and Supplementary Fig. 6e-h). Hence, the advection of South Asian monsoon moisture alone is not sufficient to explain the $\delta^{18}O_p$ spatial patterns across the East Asian monsoon region.

Fourth paragraph: Further verification using long-term observed data

The analysis of the correlation between five sites in the Asian monsoon region (Hong Kong, Guangzhou, Kunming, Dhaka, and Sylhet) and the monsoon intensity index and OLR further support that the "rainout effect" along monsoon moisture advection has a limited influence on $\delta^{18}O_p$.

Fifth paragraph: Explanation of the reasons

Finally, it was pointed out that the previous studies (Baker et al., 2015; Zhang et al., 2021) were limited by ignoring the "rainout effect" along atmospheric updraft occurring directly at precipitation sites, especially during a continuous precipitation

event.

Please see L164–L223 in the revised manuscript.

References:

Pausata, F. S. R., Battisti, D. S., Nisancioglu, K. H., & Bitz, C. M. Chinese stalagmite $\delta^{18}O$ controlled by changes in the Indian monsoon during a simulated Heinrich event. *Nat. Geosci.* **4**, 474–480 (2011).

Baker, A. J. et al. Seasonalityof westerly moisture transport in theEast Asian summer monsoon and itsimplications for interpreting precipitation $\delta^{18}O$. *J. Geophys. Res. Atmos.* **120**, 5850–5862 (2015).

Zhang, J. et al. Coupled effects of moisture transport pathway and convection on stable isotopes in precipitation across the East Asian monsoon region: Implications for paleoclimate reconstruction. *J. Climate* **34**, 9811–9822 (2021).

He, C. et al. Hydroclimate footprint of pan-Asian monsoon water isotope during the last deglaciation. *Sci. Adv.* **7**, eabe2611 (2021).

Lines 170-177: Have the authors familiarized themselves with the deuterium excess in their precipitation data? To what extent does the deuterium excess in the precipitation data vary spatially and temporally? Is there a trend between the eastern and western parts of the Asian Monsoon region similar to the $\delta^{18}O_p$ data? Also, what are "moisture flux fields"?

Re: We thank you for pointing these issues out. Following your comments, we addressed these issues as follows:

1) Indeed, we familiarize with the d-excess within our precipitation data. Generally, d-excess can serve as an indicator of moisture sources—lower d-excess values typically reflect higher humidity in the source region, whereas higher d-excess values suggest drier source conditions. However, the factors influencing d-excess are highly complex. For instance, abnormal below-cloud evaporation can lead to abnormally low d-excess values. To avoid potential biases introduced by such anomalies, we do not use d-excess in our analysis.

2) We sincerely apologize for the terminology error. Following your comment, we

have read a large number of articles and found that "moisture flux" is the correct term, instead of "moisture flux field" (Zhao & Zhou, 2021; Dorff et al., 2024). The "moisture flux" is calculated from zonal wind, meridional wind, and specific humidity data, which reveals moisture source regions and transport pathways. Following your comment, we added explaination in the "Materials and Methods" section, i.e., "*We used specific humidity combined with meridional and zonal winds to calculate the moisture flux*." Please see L749–L750 in the revised manuscript.

References:

Zhao, Y., & Zhou, T. Interannual variability of precipitation recycle ratio over the Tibetan Plateau. *J. Geophys. Res. Atmos.* **126**, e2020JD033733 (2021).

Dorff, H., Konow, H., Schemann, V., & Ament, F. Observability of moisture transport divergence in Arctic atmospheric rivers by dropsondes, *Atmos. Chem. Phys.* **24**, 8771–8795 (2024).

Line 171: The term "moisture channel" should be described for non-specialist readers.

Re: We thank you for pointing this issue out. Following your suggestion, we added a definition of "moisture channel", i.e., "*a long and narrow area with a large moisture flux*." Please see L168–L169 in the revised manuscript.

Line 173: Seems strange to be opening this (important) section with reference to a supplementary figure...

Re: We thank you for pointing this issue out. We opened this section with reference to a supplementary figure (Supplementary Fig. 7) for two main reasons:

1) Topic relevance: In this section, we will discuss the influence of the advection of monsoon moisture on $\delta^{18}O_p$. In this case, we need to calculate the moisture flux to show the moisture source and moisture transport pathway (Supplementary Fig. 7). Hence, we opened this section with reference to a supplementary figure (Supplementary Fig. 7).

2) Limitation of the number of figures: Given that the main text already contains seven figures, we had to show this figure in the Supplementary Information (Supplementary Fig. 7).

Lines 178-187: This paragraph repeats the key points made in the paragraph above, and so I suggest merging the two for brevity.

Re: We thank you for pointing these issues out. We agree with you and merged these two paragraphs. Please see L165–L180 in the revised manuscript.

Lines 188-191: This does not make sense, and is hard to follow.

Re: We thank you for pointing this issue out. It should be noted that numerous previous studies have demonstrated that $\delta^{18}O_p$ in the Asian monsoon region primarily reflects variations in the advection of South Asian monsoon moisture (i.e., stronger advection of South Asian monsoon moisture corresponds to lower $\delta^{18}O_p$ values). As we know, the South Asian summer monsoon becomes stronger gradually from late May to July (or August) and weakens from September. If the advection of monsoon moisture affects the variations in $\delta^{18}O_p$ in the South Asian monsoon region, $\delta^{18}O_p$ will decrease gradually from late May to July (or August) and increase from September. However, our study identified a crucial temporal decoupling phenomenon: a temporal difference between the continuous decline in $\delta^{18}O_p$ (May–October) in the South Asian monsoon region and the peak monsoon moisture transport (July–August) (Fig. 1c and Supplementary Figs. 2b and 7f, g). This suggests that the intensity of the South Asian summer monsoon alone cannot fully explain the seasonal variation of $\delta^{18}O_p$.

To address this apparent contradiction, in the "$\delta^{18}O_p$ and the advection of monsoon moisture" section we examined the temporal decoupling relationship between advection of South Asian monsoon moisture and $\delta^{18}O_p$ variations, thereby confirming that south Asian summer monsoon alone cannot fully explain seasonal $\delta^{18}O_p$ variations. Following your suggestion, we changed "discrepancy" to "*a marked seasonal decoupling between $\delta^{18}O_p$ and the advection of South Asian monsoon moisture*", which provided a clearer explanation of what this "discrepancy" actually is. Please see L181–L182 in the revised manuscript.

Line 194: What aspects of the SASM? Moisture sources? Strength? Something else...?

Re: It is the monsoon intensity. To avoid causing any confusion for the readers, we changed "South Asian summer monsoon" to "*South Asian summer monsoon*

intensity." Please see L188 in the revised manuscript.

Lines 196-197: Citation needed.

Re: Following your suggestion, we added the following references to the articles, i.e., Pausata et al., 2011; Baker et al., 2015; Zhang et al., 2021; He et al., 2021. Please see L194 in the revised manuscript.

References:

48. Pausata, F. S. R., Battisti, D. S., Nisancioglu, K. H., & Bitz, C. M. Chinese stalagmite $\delta^{18}O$ controlled by changes in the Indian monsoon during a simulated Heinrich event. *Nat. Geosci.* **4**, 474–480 (2011).
49. Baker, A. J. et al. Seasonalityof westerly moisture transport in theEast Asian summer monsoon and itsimplications for interpreting precipitation $\delta^{18}O$. *J. Geophys. Res. Atmos.* **120**, 5850–5862 (2015).
50. Zhang, J. et al. Coupled effects of moisture transport pathway and convection on stable isotopes in precipitation across the East Asian monsoon region: Implications for paleoclimate reconstruction. *J. Climate* **34**, 9811–9822 (2021).
57. He, C. et al. Hydroclimate footprint of pan-Asian monsoon water isotope during the last deglaciation. *Sci. Adv.* **7**, eabe2611 (2021).

Lines 203-205: Repeating "pattern" three times in this sentence.

Re: We thank you for pointing this issue out. Following your comment, we changed "As for the seasonal pattern, this spatial pattern also suggests that moisture advection from the South Asian monsoon alone cannot explain the $\delta^{18}O_p$ spatial patterns in the East Asian monsoon region" to "*Hence, the advection of South Asian monsoon moisture alone is not sufficient to explain the $\delta^{18}O_p$ spatial patterns across the East Asian monsoon region.*" Please see L200–L202 in the revised manuscript.

Line 206: Further evidence for what? Sufficient detail should be given in the opening line of any paragraph.

Re: We thank you for pointing this issue out. It is not suitable to start this paragraph with "further evidence come from." Following your suggestion, we changed "Further evidence comes from correlations between $\delta^{18}O_p$ at five sites (Hong Kong,

Guangzhou, Kunming, Dhaka, and Sylhet) with long-term (>10-year) records and various monsoon intensity indices" to "*We further examined correlations between* $\delta^{18}O_p$ *at five sites (Hong Kong, Guangzhou, Kunming, Dhaka, and Sylhet) with long-term (>10-year) records and various monsoon intensity indices.*" Please see L203–L205 in the revised manuscript.

Line 207: Is decadal scale really long-term? And how long-term are we talking about here? Centuries? We also suggest providing an additional map to show the location of the five sites. This will provide information how these sites are located in relation to the different monsoon regions and the precipitation sites.

Re: We thank you for pointing these issues out. In precipitation stable isotope studies, high-resolution short-term data typically refer to hourly or event-based (precipitation time) and daily-scale measurements. Consequently, for precipitation stable isotope studies, decade-long datasets are generally considered long-term records.

Considering that there are too many figures in the Supplementary Information (based on the comments provided by you and both Reviewers #3 and #4, we added a large number of additional figures), we do not plan to provide an additional map to show the location of the five sites. Nevertheless, comprehensive latitude and longitude information for five sites is provided in Supplementary Tables 1 and 2. Please see Supplementary Tables 1 and 2 in the revised Supplementary Information.

Lines 211-213: Please provide a reference.

Re: We thank you for pointing this issue out. Following your suggestion, we provided the references, i.e., Liebmann et al., 1996; Webster et al., 1998. Please see L211 in the revised manuscript.

References

58. Liebmann, B. & Smith, C. A. Description of a complete (interpolated) outgoing longwave radiation dataset. *Bull. Amer. Meteorol. Soc.* **77**, 1275–1277 (1996).

59. Webster, P. J. et al. Monsoons: Processes, predictability, and the prospects for prediction. *J. Geophys. Res.* **103**, 14451–14510 (1998).

Line 212: Where has this proxy been established as a convection indicator? Citation needed.

Re: We thank you for pointing this issue out. Following your suggestion, we added references. Please see L211 in the revised manuscript.

References

58. Liebmann, B. & Smith, C. A. Description of a complete (interpolated) outgoing longwave radiation dataset. *Bull. Amer. Meteorol. Soc.* **77**, 1275–1277 (1996).
59. Webster, P. J. et al. Monsoons: Processes, predictability, and the prospects for prediction. *J. Geophys. Res.* **103**, 14451–14510 (1998).

Line 218: Or “alongside”? Also, please provide a reference.

Re: We thank you for pointing this issue out. In this section, the design intention of these two sentences is to form a clear contrast. Previous studies mainly focused on the horizontal “rainout effect” occurring along moisture transport pathways (from source regions to precipitation sites), while neglecting the vertical “rainout effect” that directly takes place in the precipitation site and is related to the atmospheric updraft. It seems that the term “alongside” is hard to adequately capture this critical distinction. We therefore maintain the use of “However” to highlight the fundamental differences between these distinct mechanisms.

Following your suggestion, we added two references, i.e., Baker et al., 2015; Zhang et al., 2021. Please see L218 in the revised manuscript.

References:

49. Baker, A. J. et al. Seasonalityof westerly moisture transport in theEast Asian summer monsoon and itsimplications for interpreting precipitation $\delta^{18}O$. *J. Geophys. Res. Atmos.* **120**, 5850–5862 (2015).
50. Zhang, J. et al. Coupled effects of moisture transport pathway and convection on stable isotopes in precipitation across the East Asian monsoon region: Implications for paleoclimate reconstruction. *J. Climate* **34**, 9811–9822 (2021).

Lines 219-220: What constitutes a "continuous" event? Careful with this definition, as

'continuous' can be overly broad and obscure.

Re: We thank you for pointing this issue out. Following your suggestion, we added an explanation for "a continuous precipitation event", i.e., "*the duration of a continuous precipitation event is generally several hours or even several days*." Please see L218–L220 in the revised manuscript.

Line 221: Even if these processes are described on, the reader should be given an impression of what kinds of processes are being referring to.

Re: We thank you for pointing this issue out. Following your suggestion, we provided an impression of what kinds of processes are being referring to, i.e., "*frontal lifting and convective instability*." Please see L222 in the revised manuscript.

Lines 232-233: Helpful would be to delineate between 'upward motion' and 'updraft' - a non-specialist reader may assume they are the same thing.

Re: We thank you for pointing this issue out. Following your comment, we changed "upward motion" to "*large-scale ascent*." In this case, we highlight the direction of the air flow on large spatial scale. To delineate between "large-scale ascent" and "atmospheric updraft", we added "*local*" before "atmospheric updraft." Please see L243–L244 and L247 in the revised manuscript.

Line 248: "…migrating westward." This cannot be clearly identified by the Figures. In Figure 3h vertical velocities and OLR tend to be higher in the western region. I suggest calling it a westward expansion of low vertical velocities and OLR rather than a migration. The spatial area of low vertical velocities and OLR is increasing westward over time.

Re: We thank you for pointing this issue out. Following your comments, we carefully examined the data month by month, and found that lower OLR and vertical velocity values continue migrating westward to the middle of the transect from February to July. Indeed, our analysis confirms that this westward migration ceased during August and September. As shown in Figure 3h, vertical velocities tend to be higher in the western region. These results indicate that the low vertical velocity and OLR values do not continuously migrate westward, but rather stabilize after migrating to the middle of the transect. Hence, the lower vertical velocities and OLR values did not

expand westward to the westernmost sites. Following your comment, we changed "Throughout the monsoon season, low vertical velocity and OLR values continue migrating westward, with the lowest values appearing in the middle of the transect" to "*Throughout the monsoon season, low OLR and vertical velocity values continue migrating westward to the middle of the transect.*" Please see L259–L260 in the revised manuscript.

Lines 249 -250: This observation does not match the changes in vertical velocities and OLR from July to August, showing a decrease in these values.

Re: We thank you for pointing this issue out. Following your comment, we carefully examined this sentence and found that there is indeed a problem with this statement. Therefore, we deleted the sentence "Meanwhile, vertical velocity and OLR values in the East Asian monsoon region gradually increase." Please see L255–L269 in the revised manuscript.

Lines 250-255: A V-shape spatial pattern of vertical velocity and ORL requires increasing velocities in the western transect as well. However, this contradicts the statement made in line 247-249, indicating low vertical velocities and OLR values in the western transect during the monsoon period. A clear V-shape spatial pattern of vertical velocity and ORL as it is described here (high values at the margins, low values in the middle of the transect) can only be observed during September. The authors clarify that the V-shaped pattern indicates a stronger updraft in the center and weaker updraft on the eastern and western margins (line 251-252). However, the months with the strongest V-pattern in the atmospheric updraft (September and to a certain degree June; Fig. 3e & h) are the months with the weakest V-pattern in the $\delta^{18}O_p$ values (Fig. 2e & h). By contrast, the V-shape of $\delta^{18}O_p$ values is strongest pronounced in July and August (Fig. 2f & g) associated with more consistent atmospheric updraft intensity (Fig. 3f & g) from the middle to the margins of the transect. This calls into question the subsequent statement that atmospheric updraft is driving the V-shaped spatial pattern in $\delta^{18}O_p$ (l. 253f). Furthermore, there is no concrete statement on how atmospheric updraft affects $\delta^{18}O_p$. Does $\delta^{18}O_p$ decrease with increasing atmospheric updraft or vice versa? This requires a more detailed discussion.

Re: We thank you for pointing these issues out. Following your comments, we addressed these issues as follows:

1) With regard to this (line 250-255) contradicts the statement made in line 247-249, we checked the line 247-249 "Throughout the monsoon season, low vertical velocity and OLR values continue migrating westward, with the lowest values appearing in the middle of the transect", and found the statement is ambiguous. We changed this sentence to "*Throughout the monsoon season, lower OLR and vertical velocity values continue migrating westward to the middle of the transect*." Please see L259–L260 in the revised manuscript.

2) With regard to the less distinct V-shaped pattern in OLR and vertical velocity during July and August, which contrasts with the pronounced V-shape seen in $\delta^{18}O_p$, we want to emphasize the main features and the overall pattern (which can generally match the $\delta^{18}O_p$ pattern from February to September). We admit that there are indeed differences in the details, that is, the match in July and August is not very good. This inconsistency may arise from different spatial scales: the $\delta^{18}O_p$ pattern reflects local site-scale variability (Fig. 2), whereas Fig. 3 shows the OLR and vertical velocity patterns representing large-scale averages (over 20–27° N). Therefore, when comparing at different spatial scales (Figures 2 and 3), it is understandable that there are subtle differences. However, if comparisons are made at the site scale, the result would become better (Supplementary Fig. S8f, g). Following your comments, we added an explanation for this inconsistency, i.e., "*Notably, although low OLR and vertical velocity reach Sagar in July–August, $\delta^{18}O_p$ at Sagar does not decrease as expected. This discrepancy likely reflects spatial scale differences: $\delta^{18}O_p$ captures site-scale variability (Fig. 2), whereas OLR and vertical velocity are large-scale averages (over 20–27° N, Fig. 3). Therefore, comparing $\delta^{18}O_p$ with OLR at the site scale would likely yield better agreement (Fig. 2f, g and Supplementary Fig. 8f, g).*" Please see L263–L269 in the revised manuscript.

3) With regard to the comments of "there is no concrete statement on how atmospheric updraft affects $\delta^{18}O_p$", in the last paragraph of the "$\delta^{18}O_p$ and atmospheric updraft" section in the previous manuscript, we provide a detailed explanation of the mechanism whereby atmospheric updraft affect $\delta^{18}O_p$. Following your suggestion, we moved this paragraph to the beginning of this section. Please see

L225–L236 in the revised manuscript.

Line 267: Isn't this just another version of the statement at the beginning of the section?

Re: We thank you for pointing this issue out. The beginning of this section, we provided a qualitative analysis. However, here we aim to perform a quantitative analysis, including correlation and gradients. Following your comments, we changed "To quantify the impact of atmospheric updraft on $\delta^{18}O_p$, we first calculated the correlation between $\delta^{18}O_p$ and OLR, as well as with vertical velocity" to "*We further quantified the influence of atmospheric updraft on $\delta^{18}O_p$. First, we calculated the correlation between $\delta^{18}O_p$ and OLR, as well as with vertical velocity.*" Please see L279–L280 in the revised manuscript.

Line 268: Which correlation test was used, and why?

Re: We thank you for pointing this issue out. We used the Pearson's correlation test. Indeed, there are many methods to test whether the monthly OLR and observed $\delta^{18}O_p$ data follow a linear relationship, including the Pearson's correlation test. Since our data satisfy a normal distribution (Figure R13), we used the Pearson's correlation test. Following your comment, we added one sentence in the "Materials and Methods" to make it clear. Please see L726 in the revised manuscript.

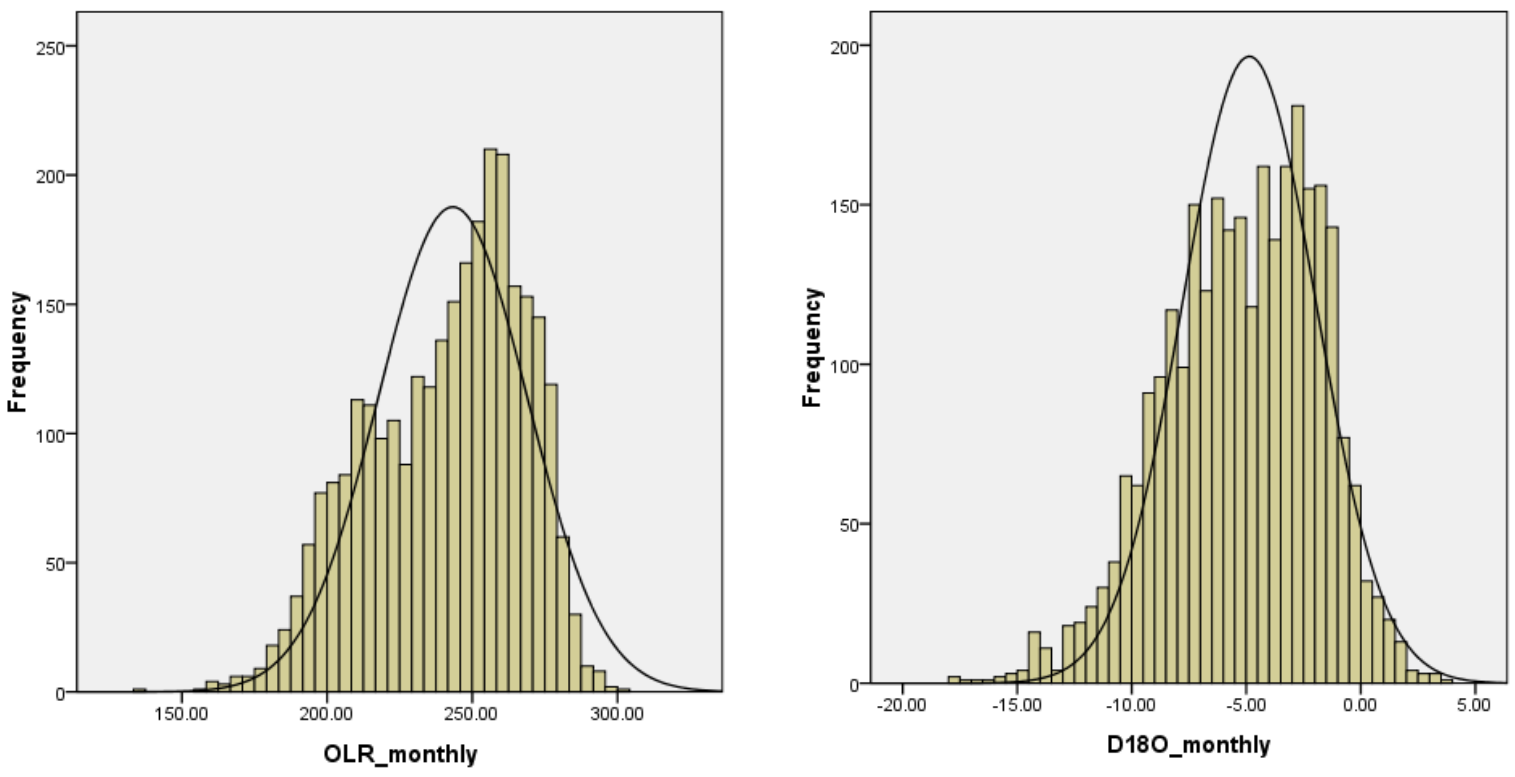

Figure R13. The histogram illustrates the distributions of the datasets including monthly OLR and observed $\delta^{18}O_p$ data.

Lines 284-285: We are not entirely convinced based on the presentation of results here. The authors describe the figure, but don't offer any mechanistic or conceptual

explanations for the observed trends. Please be more specific, how exactly atmospheric updraft affects $\delta^{18}O_p$ values. Stronger atmospheric updraft cause more negative $\delta^{18}O_p$ values or the other way around?

Re: We thank you for pointing this issue out. As mentioned above, following your comments, we carefully examined the "$\delta^{18}O_p$ and atmospheric updraft" section and found that this issue does indeed exist. In this section, we assumed that readers will understand that a stronger atmospheric updraft will result in lower precipitation isotopes. That is, when we discuss how atmospheric updraft affect the variations in $\delta^{18}O_p$ along the transect from the east to the west during the pre-monsoon to monsoon, and to post-monsoon seasons, and even discuss their correlation between $\delta^{18}O_p$ and OLR (atmospheric updraft), we have always assumed that the readers have the prior knowledge. It is not until the end of this section that we use a paragraph to explain how atmospheric updraft affects the variation in $\delta^{18}O_p$. Clearly, we reversed the logical sequence of the text. following your comment, we moved the description of how atmospheric updraft affects the changes in $\delta^{18}O_p$ in the last paragraph of this section to the beginning. This restructuring provides essential background knowledge at the beginning of this section, thereby readers can better understand our subsequent analyses. Please see L225–L236 in the revised manuscript.

With regard to how atmospheric updraft influences $\delta^{18}O_p$ values, we found that stronger atmospheric updraft causes lower $\delta^{18}O_p$ values, and vice versa.

Lines 292-293: In what sense? Further clarification is needed here.

Re: We thank you for pointing this issue out. We checked the sentences "This explains why the correlations between observed $\delta^{18}O_p$ and monsoon indices are much weaker than those between $\delta^{18}O_p$ and OLR on both monthly (Supplementary Table 3) and annual (Supplementary Table 4) timescales. In contrast, atmospheric updraft provides a better explanation for the spatiotemporal patterns of $\delta^{18}O_p$ along the transect of the Asian monsoon region, indicating that $\delta^{18}O_p$ changes are significantly driven by atmospheric updraft", and found that there is a jump from OLR to atmospheric updraft, and the term "In contrast" is unsuitable. To address this issue, we added an explanation for the relationship between OLR and atmospheric updraft, i.e., "*The lower the OLR, the stronger the atmospheric updraft.*" Please see L210–L211 in

the revised manuscript. In addition, we changed "In contrast, atmospheric updraft provides a better explanation for the spatiotemporal patterns of $\delta^{18}O_p$ across the transect of the Asian monsoon region" to "*Atmospheric updraft thus provides a better explanation for the spatiotemporal patterns.*" Please see L300–L301 in the revised manuscript.

Lines 297-307: There are a series of statements made throughout this entire paragraph without any reference being made. Since these are general observations and relationships, we suggest providing appropriate references.

Re: We thank you for pointing this issue out. Following your suggestion, we added relevant references (Yu et al., 2021; Zhang et al., 2021; Cole et al., 1999; Vuille et al., 2003; Kurita, 2013). Please see L225–L236 in the revised manuscript.

References:

Cole, J. E., Rind, D., Webb, R. S., Jouzel, J. & Healy, R. Climatic controls on interannual variability of precipitation $\delta^{18}O$: Simulated influence of temperature, precipitation amount, and vapor source region. *J. Geophys. Res.* **104**, 14223–14235 (1999).

Kurita, N. Water isotopic variability in response to mesoscale convective system over the tropical ocean. *J. Geophys. Res. Atmos.* **118**, 10376–10390 (2013).

Vuille, M., Bradley, R. S., Werner, M., Healy, R. & Keimig, F. Modeling $\delta^{18}O$ in precipitation over the tropical Americas: 1. Interannual variability and climatic controls. *J. Geophys. Res.* **108**, 4174 (2003).

Yu, W. et al. Temperature signals of ice core and speleothem isotopic records from Asian monsoon region as indicated by precipitation $\delta^{18}O$. *Earth Planet. Sci. Lett.* **554**, 116665 (2021).

Zhang, J. et al. Coupled ebelgamanffects of moisture transport pathway and convection on stable isotopes in precipitation across the East Asian monsoon region: Implications for paleoclimate reconstruction. *J. Climate* **34**, 9811–9822 (2021).

Lines 315-316: Why does this play a key role?

Re: We thank you for pointing this issue out. The western Pacific subtropical high

(WPSH) play a key role, because that it persistently dominates the western Pacific, serving as a vast warm anticyclonic circulation system that transports moisture to East Asia (Lau and Li 1984). During the pre-monsoon season (February–May), the southerly winds along the western flank of the WPSH transport warm, moist air northward into the East Asian monsoon region. When this warm, moist air collides with the southward-flowing cold air from the mid-latitudes, an inclined updraft forms along the frontal zone (Chung et al., 2011; Huang et al., 2018; Li et al., 2024). This frontal lifting further enhances atmospheric updraft in the region.

Furthermore, the WPSH exhibits seasonal meridional migration that directly influences the location of East Asian rainbelt. As the WPSH shifts northward, the East Asian precipitation belt follows accordingly. Therefore, this system plays a pivotal role in regional hydrometeorology.

Due to limitation of the length of the main text, we only referenced key studies (including Lau and Li 1984; Chung et al., 2011; Huang et al., 2018; Li et al., 2024) rather than elaborating in detail within this paragraph. However, we provided comprehensive analysis of WPSH's role in influencing $\delta^{18}O_p$ in the subsequent paragraph. Please see L348–L354 in the revised manuscript.

References:

Chung, P. H., Sui, C. H., & Li T. Interannual relationships between the tropical sea surface temperature and summertime subtropical anticyclone over the western North Pacific. *J. Geophys. Res. Atmos.* **116**, D13111 (2011).

Huang, Y., Wang, B., Li, X. & Wang, H. Changes in the influence of the western Pacific subtropical high on Asian summer monsoon rainfall in the late 1990s. *Clim. Dyn.* **51**, 443–455 (2018).

Lau, W. K. M. & Li, M. T. The monsoon over East Asia and its global association-a survey. *Bull. Am. Meteor. Soc*. **65**, 116–125 (1984).

Li, S. et al. The interdecadal changes of the relationship between May-June and July-August NWPSH and their physical mechanisms. *Geophys. Res. Lett.* **51**, e2024GL111289 (2024).

Line 323: Why not "pre-monsoon period", like in the previous sections to keep

consistent pronunciation and improve readability.

Re: We thank you for pointing this issue out. Following your suggestion, we changed "During February–May" to "*During the pre-monsoon period (February–May).*" Please see L341 in the revised manuscript.

Line 330: Are there no references to published literature that would be relevant here? Also, is this not an excessive number of supplementary figures to be referring to?

Re: We thank you for pointing these issues out. Following your comment, we conducted a comprehensive literature review and found there is no relevant reference. Our findings are primarily supported by the IPWP and ITCZ characteristics.

Indeed, this is an excessive number of supplementary figures to be referring to. Following your suggestion, we removed the references of Supplementary Figs. 14 and 20. Please see L348 in the revised manuscript.

Line 349: Please provide a reference to the state of monsoon intensity. June to August = monsoon period

Re: Following your comment, we added a reference (Li et al., 2024) to explain that WPSH will move northeastward during the monsoon period (June to August). Additionally, June to August refers to the monsoon period. Following your comment, we changed "In comparison, from June to August, the WPSH ridge moves eastward and shifts northward" to "*In comparison, during the monsoon season (from June to August), the WPSH ridge moves eastward and shifts northward*[***Error! Reference source not found.***]" Please see L364–L365 in the revised manuscript.

References:

82. Li, S. et al. The interdecadal changes of the relationship between May-June and July-August NWPSH and their physical mechanisms. *Geophys. Res. Lett.* **51**, e2024GL111289 (2024).

Lines 358-362: This paragraph requires further discussion. The extent to which the IPWP affects convection across the transect during the monsoon period is not sufficiently discussed. What "combined influence" are the authors referring to? How

can a gradually westward strengthening convection result in weaker convection at the western margins?

Re: We thank you for pointing these issues out. Following your comments, we addressed these issues as follows:

1) With regard to how can a gradually westward strengthening convection result in weaker convection at the western margins, we carefully examined the expression here and found that this sentence was incorrect. Moreover, we found that this sentence conflicts with the previous expression: "From June to August, both the ITCZ and IPWP continue to migrate northward (Fig. 5e-g and Supplementary Fig. 18e-g), leading to a substantial increase in atmospheric convection across the Asian monsoon region (Fig. 3e-g and Supplementary Figs. 12e-g, 14, and 20e-g)." Please see lines 352-355 in the previous manuscript. We are very sorry for this mistake. Through thorough reanalysis, we found that atmospheric updraft (convection) at both the eastern and western margins do not weaken during the monsoon season (from June to August). Instead, enhanced atmospheric updraft (convection) in the middle of the transect generates the V-shaped pattern. Hence, we removed "weaker in the eastern and western margins." Please see L373–L375 in the revised manuscript.

2) With regard to "combined influence", our data indicate that the ITCZ not only shifts northward but also exhibits a pronounced westward expansion. In contrast, the IPWP only move northward, with no clear evidence of westward extension. In other words, compared to the ITCZ, the influence of the IPWP is relatively limited. Therefore, we do not systematically discuss the role of the IPWP in driving the westward migration of stronger atmospheric updraft (convective) along the transect during monsoon periods. Consequently, the use of the term "combined influence" is problematic here. Following your comment, we removed "combined influence" and now emphasize the dominant role of the ITCZ. That is, we changed "Consequently, the ITCZ and IPWP collectively drive the gradually strengthening convection to move westward along the transect. The combined influence of the ITCZ and IPWP cause the convection to become stronger in the middle (Fig. 3 and Supplementary Figs. 12, 14 and 20), which leads to the V-shaped pattern of $\delta^{18}O_p$ values along the east-west transect" to "*Consequently, the ITCZ drives the gradually strengthening convection to move further westward along the transect. The influence of the ITCZ causes the*

convection to become stronger in the middle (Fig. 6b), which leads to the V-shaped pattern of $\delta^{18}O_p$ values along the east-west transect (Fig. 6b)." Please see L372–L375 in the revised manuscript.

Lines 360-361: Rather than a ton of supplementary screen-grabs from the analysis, we think the manuscript would benefit hugely from inclusion of a conceptual cartoon/diagram that clearly presents these mechanisms at work. This would help the reader to visualise what is going on, and how seasonal changes in updraft dynamics could explain the observed seasonal trends in precipitation $\delta^{18}O$.

Re: We thank you for pointing this issue out. Following your comments, we added a new schematic diagram figure to help readers visualize the the mechanisms that spatial distributions of $\delta^{18}O_p$ along the transect during the pre-monsoon and monsoon seasons (Figure R14). Please see the new Fig. 6 in the revised manuscript.

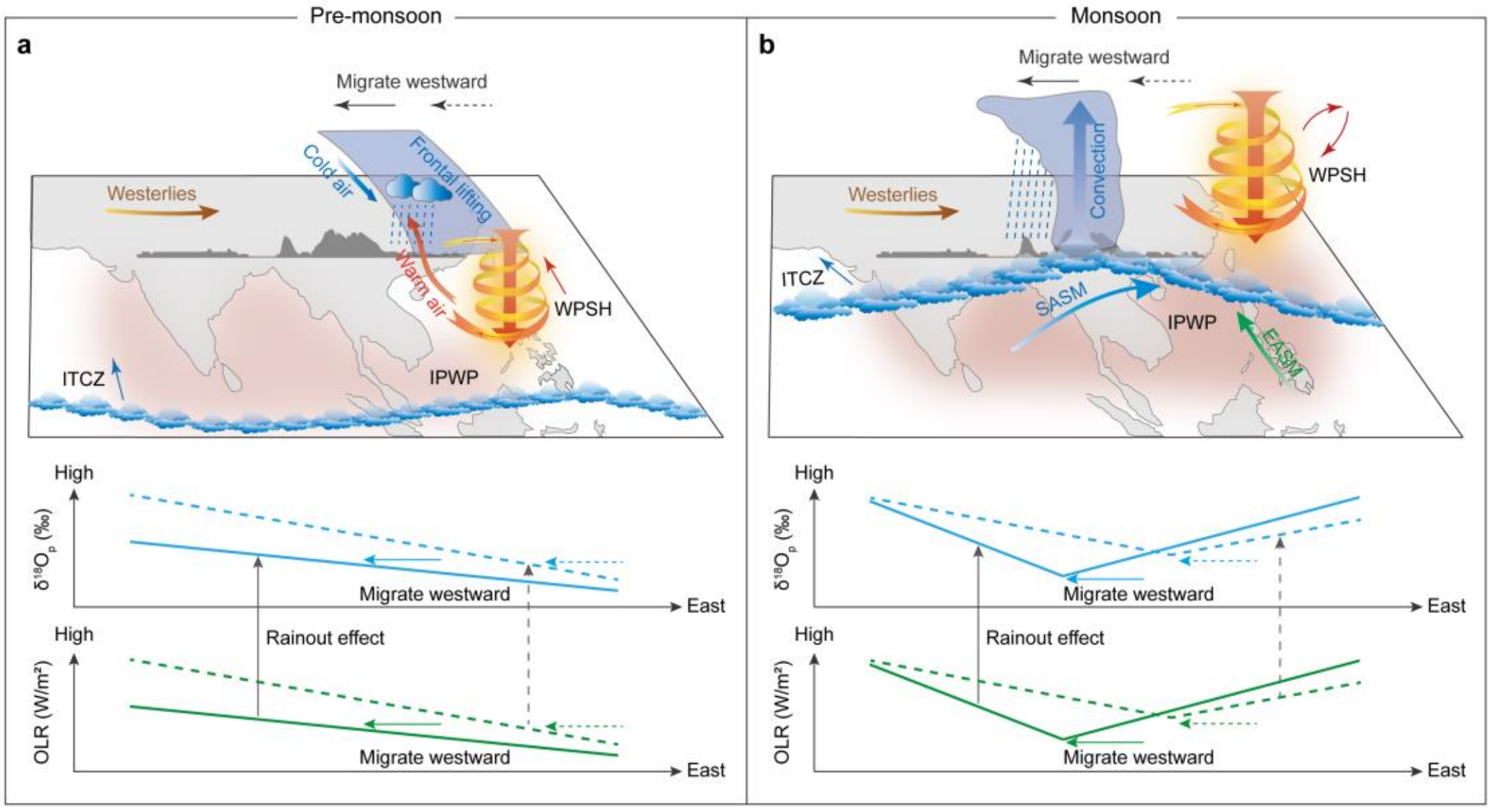

Figure R14. Schematic illustration of mechanisms controlling the spatial distributions of $\delta^{18}O_p$ along the transect during the pre-monsoon and monsoon seasons. a Pre-monsoon season. b Monsoon season. Horizontal solid arrows indicate the final state (May during the pre-monsoon season or September during the monsoon season) of stronger atmospheric updraft and lower $\delta^{18}O_p$ migrating westward, while horizontal dashed arrows represent the prior state (February during the pre-monsoon season or June during the monsoon season). The deep gray shading in the topographic map shows the terrain profile along the east–west transect. Abbreviations: ITCZ,

Intertropical Convergence Zone; IPWP, Indo-Pacific Warm Pool; WPSH, Western Pacific subtropical high; SASM, South Asian summer monsoon; EASM, East Asian summer monsoon; OLR, outgoing longwave radiation. The blue and red arrows adjacent to ITCZ and WPSH indicate their movement directions during the pre-monsoon and monsoon seasons. Figure created with Adobe Illustrator 2024.

Line 378: We think this is overly broad, and the question would be better cast as: “What aspects of monsoonal variability do Asian $\delta^{18}O$ signals capture, and how could these signals be affected by geography?"

Re: We thank you for pointing this issue out. It should be noted that while some studies used Asian speleothem $\delta^{18}O$ records to capture monsoon precipitation signals, others employed them to reconstruct South Asian monsoon intensity. However, our study challenges these interpretations of these previous studies. Therefore, we pose the following question: “However, a fundamental question remains: what do the $\delta^{18}O$ signals preserved in the Asian speleothem archives truly represent?”

In order to better describe the statements of the previous research, we changed “Some studies even used these $\delta^{18}O_s$ records from the East Asian monsoon region to infer changes in the South Asian monsoon” to “*Some studies have also used the $\delta^{18}O_s$ records from the East Asian monsoon region to infer past variability in South Asian monsoon intensity*[48,57].” Please see L392–L394 in the revised manuscript.

Lines 382-383: In the instrumental data? Or the proxy data?

Re: We checked the sentence “The results show that annual $\delta^{18}O_p$ values also follow a distinct V-shaped pattern (Supplementary Fig. 21), consistent with $\delta^{18}O_p$ patterns observed during the monsoon season (Fig. 2e-h)” and found that these are all the instrumental data of the 12 primary sites. That is, the $\delta^{18}O_p$ data from these 12 primary sites represent instrumental data only, while all proxy data ($\delta^{18}O_s$ records) in this study were derived from 13 caves.

Line 392-394: Misleading argument. According to Fig. 1, there is no cave located at the western margin. The most western cave is located at ~93°E, which corresponds to the middle of the transect rather than the margins.

Re: We thank you for pointing this issue out. Indeed, there is no cave located at the western margin. Following your comment, we changed "A comparison of 13 $\delta^{18}O_s$ records spanning the last 50,000 years revealed that values tend to be higher (> –8‰) at the eastern and western margins and lower (< –8‰) in the middle of the transect" to "*A comparison of the $\delta^{18}O_s$ records from 13 caves spanning the last 50,000 years revealed that values tend to be higher (> –8‰) at the east and west and lower (< –8‰) in the middle of the transect (Fig. 7a).*" Please see L409–L411 in the revised manuscript.

Lines 403-404: We think this is a bold statement that is not entirely backed up by the data. From what we understand, temperature differences between the surface and higher altitudes (e.g., lapse rate) can have an effect on updrafts such that greater differences lead to stronger updrafts - due to increased buoyancy. Also, what is to say that orographic lifting couldn't also affect precipitation? We think because the topography of Asia is so diverse, altitudinal and topographic effects need to be explored in much closer detail before all processes aside from updraft can be ruled out.

Re: We thank you for pointing this issue out. Following your suggestion, we have tried to address all the issues as follows:

1) With regard to the comment of "temperature differences between the surface and higher altitudes (e.g., lapse rate) can have an effect on updrafts such that greater differences lead to stronger updrafts - due to increased buoyancy", this means that altitude will affect atmospheric updraft. As we know, topography remains constant. If we assume a relationship exists between atmospheric updraft and altitude, it would imply that the spatial pattern of atmospheric updraft along the transect should match the topographic distribution during both the pre-monsoon and monsoon seasons. That is, given the topography of higher altitude in the middle and lower altitude at the eastern and western margins along this transect, atmospheric updraft should theoretically be strongest in the middle and weaker at the eastern and western margins along this transect during both the pre-monsoon and monsoon seasons. However, this contradicts our findings: our results show that local atmospheric updraft is weaker in the South Asian monsoon region but relatively stronger in the East Asian monsoon

region during the pre-monsoon season, although local atmospheric updraft is indeed stronger in the middle. Therefore, we conclude that in our study, the spatial pattern of atmospheric updraft intensity is not primarily controlled by topography, but rather results from the combined influences of three major atmospheric systems: the ITCZ, IPWP, and WPSH.

2) With regard to whether orographic lifting can also affect precipitation, we analyzed the spatial pattern of precipitation amount along the transect following your comments (Figure R15). The results clearly demonstrate that the precipitation amount pattern does not correspond with the topographic profile during either the pre-monsoon or monsoon seasons. We therefore conclude that precipitation amount in our study region shows little relationship with orographic lifting.

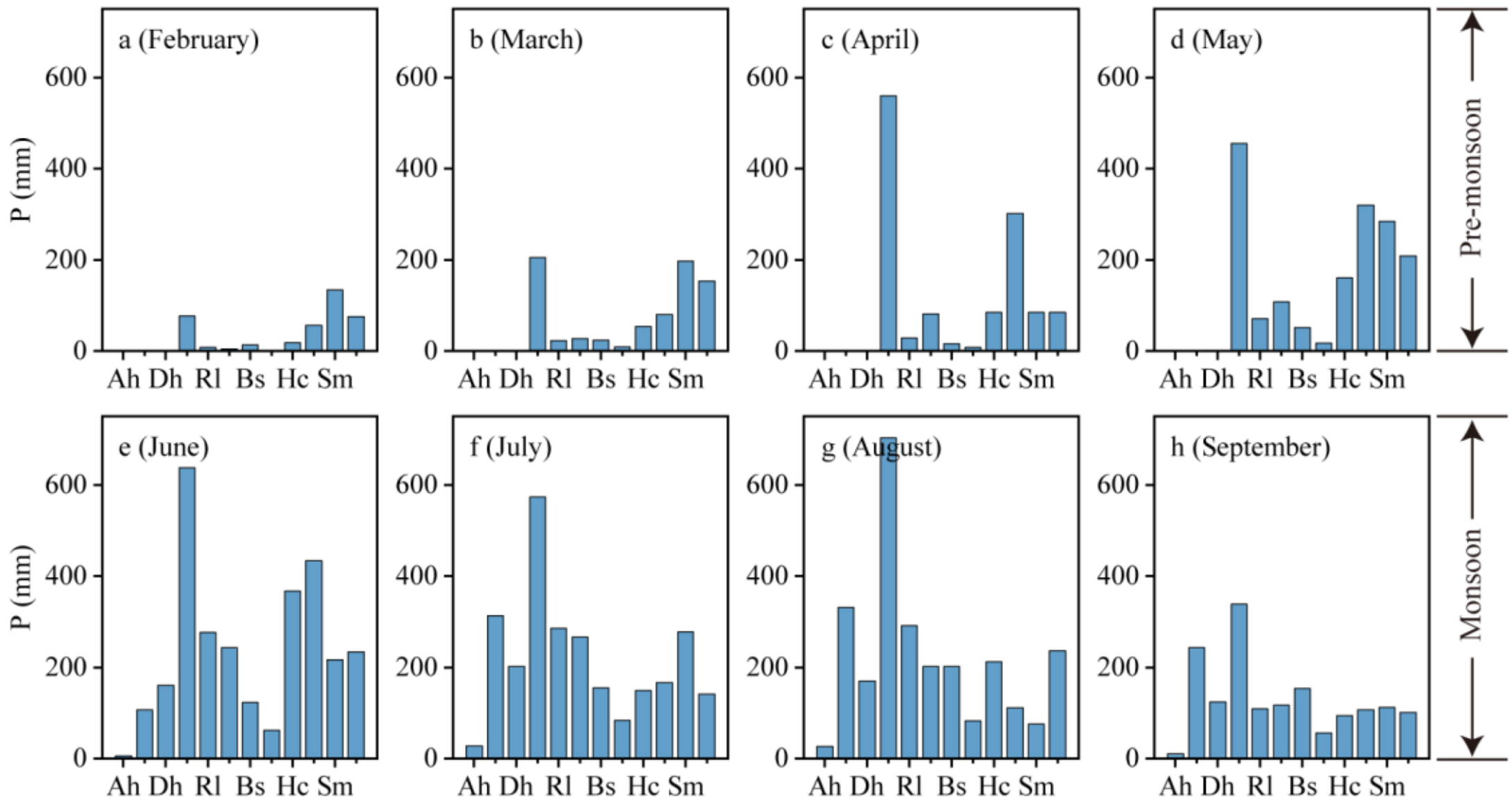

Figure R15. Spatial distributions of precipitation amount at the 12 primary sites along the transect during the pre-monsoon and monsoon seasons. a–d, Pre-monsoon season. e–h, Monsoon season. Abbreviations: Ah (Ahmedabad), Dh (Dhanbad), Rl (Ruili), Bs (Baoshan), Hc (Hechi), and Sm (Sanming).

3) With regard to the comment on the need for more detailed investigation of altitudinal and topographic effects before excluding non-updraft processes, we added several paragraphs in the “$\delta^{18}O_P$ and atmospheric updraft” section to discuss the relationship between altitude (precipitation amount) and $\delta^{18}O_P$, and then to eliminate all processes other than atmospheric updraft, including both “altitude effect” and

precipitation "amount effect." Please see L302–L325 in the revised manuscript.

Notably, our additional analysis reveals that the relationship between $\delta^{18}O_p$ and altitude undergoes gradual changes from February to September (as mentioned above). This finding not only demonstrates the lack of correlation between $\delta^{18}O_p$ and altitude, but also confirms the low $\delta^{18}O_p$ values gradually migrate toward the middle of the transect from the pre-monsoon to the monsoon season (resulting from the westward migration of enhanced atmospheric updrafts). Since $\delta^{18}O_s$ records inherit the signals of $\delta^{18}O_p$, the influence of altitude on $\delta^{18}O_s$ would consequently be very limited. In contrast, atmospheric updraft plays a key role. Clearly, it is unnecessary to re-examine the relationship between $\delta^{18}O_s$ and altitude. Thus, we removed these sentences from the manuscript.

Line 407: Spatial pattern of what?

Re: It is "spatial $\delta^{18}O_s$ patterns." Following your comment, we changed "its impact on the spatial pattern may be secondary to that of atmospheric updraft." to "*spatial $\delta^{18}O_s$ patterns cannot be attributed solely to South Asian monsoon moisture transport*" Please see L428–L429 in the revised manuscript.

Line 409: We are not entirely sure what this "framework" is...By definition, a framework is a conceptual structure upon which ideas, hypotheses, and methods can be built. However, no such framework is presented in this study, and as a researcher, we would be unclear on how to use the results of this work to inform our interpretation of a speleothem record; beyond simply considering that updraft may be important. So, either edit the wording of this statement, or include a more systematic, step-wise description of how this study can be build into those in future.

Re: We thank you for pointing this issue out. We agree with your comments on the new "framework." Following your comments, we changed "new framework" to "new perspective." Please see L432 in the revised manuscript. That is, in future studies interpreting $\delta^{18}O_s$ signals, we emphasize that atmospheric updraft ("vertical perspective") plays a important role in controlling Asian $\delta^{18}O_s$ variations than the traditionally emphasized horizontal moisture advection ("horizontal perspective"). To further make clear the "new perspective", we also changed "Our study highlights that

atmospheric updraft plays a more important role in controlling Asian $\delta^{18}O_s$ variations than previously recognized. Our hypothesis offers a new perspective on the interpretation of Asian $\delta^{18}O_s$ records." to "*Unlike the conventional view, our study highlights that atmospheric updraft plays an important role in controlling Asian $\delta^{18}O_s$ variations. Compared with the traditional interpretation focused on horizontal processes, our hypothesis offers a new perspective emphasizing vertical atmospheric dynamics*." Please see L64–L67 in the revised manuscript.

Lines 410-411: But only on timescales relevant to the Late Pleistocene/Holocene right?

Re: Yes, our study is relevant to the last 50,000 years. Following your suggestion, we added the temporal constraint "*over the last 50,000 years*." Please see L434 in the revised manuscript.

Line 628: Describe what these latitude and temperature effects are.

Re: We thank you for pointing these issues out. Following your comment, we described these terms, i.e., "*However, significant latitudinal variations in $\delta^{18}O_s$ may also result from pronounced "latitude effects" ($\delta^{18}O$ systematically decreases from tropical to polar zones)*[***Error! Reference source not found.***] *and/or "temperature effects" ($\delta^{18}O$ decreases with lower condensation temperatures)*[***Error! Reference source not found.***]" in the revised manuscript. Please see L665–L668 in the revised manuscript.

References:

69. Dansgaard, W. Stable isotopes in precipitation. *Tellus* **16**, 436–468 (1964).

83. Rozanski, K., Araguas-Araguas, L. & Gonfiantini, R. Isotopic patterns in modern global precipitation. In Continental Isotope Indicators of Climate (eds Swart, P. K., Lohmann, K. C., McKenzie, J. & Savin, S.) *Geophys. Monogr. Ser.* **78**, 1–36 (American Geophysical Union, 1993).

Line 744: There are 14 caves marked on the map, whereas the main text and caption refer to 13 caves. Please add numbers and names of the caves.

Re: We thank you for pointing these issues out. We rechecked the ArcGIS mapping data file and found that there is an error. As stated in the previous manuscript (Line54,

Line103, Line392, Line744, and Lines 808-810), and Supplementary Table 3, there should be 13 caves. We are very sorry for this error. We corrected the cave locations and the number of caves in Figure 1, and added numerical labels to all caves in both Fig. 1 and Table S3. Please see the new Fig. 1 in the revised manuscript and Table S3 in the revised Supplementary Information.

Line 809: Figure 6 feels to have been assembled in a great hurry, and as a result appears quite messy. Why not colour-code the curves to match the whisker plots below? Also, we think U-Th ages for the featured speleothems should be included if one seeks to compared time-resolved changes in different datasets.

Re: We thank you for pointing this issue out. Following your suggestion, we have tried to address all the issues as follows:

With regards to the color inconsistency in Figure 6 (new Fig. 7), we systematically recolored all $\delta^{18}O_s$ records using a unified color scheme to ensure perfect matching between each cave's line plot and corresponding whisker plot (Figure R16). Please see the new Fig. 7 in the revised manuscript.

With regard to "U-Th ages", following your comment, we systematically analyzed the U-Th age data of all $\delta^{18}O_s$ records. The results demonstrate that the dating error for the majority of these $\delta^{18}O_s$ records was relatively small (Figure R17), indicating their suitability for this study. Although the X3 $\delta^{18}O_s$ records from Xiangshui cave showed a relatively large dating error (up to 5,994 years). However, as shown in Figure R16a, its $\delta^{18}O_s$ values were generally located between the MAW/CH and LNT/FY/XY curves. That is, regardless of whether the X3 $\delta^{18}O_s$ curve of the Xiangshui Cave is shifted left or right along the X-axis, its $\delta^{18}O_s$ values always remained stable between the MAW/CH and LNT/FY/XY $\delta^{18}O_s$ records. Therefore, using the "relative value" method, the multi-year mean $\delta^{18}O_s$ value of the X3 $\delta^{18}O_s$ from Xiangshui cave should be lower than that of the MAW/CH $\delta^{18}O_s$ record and higher than that of the LNT/FY/XY $\delta^{18}O_s$ record. The results in Figure 16b confirmed our above speculation. This fully proves that the dating error of the X3 $\delta^{18}O_s$ record from Xiangshui cave will not affect our conclusions. More important, our study focuses on the spatial pattern of the multi-year mean values of $\delta^{18}O_s$ record rather than the reconstruction of paleoclimate. Therefore, the impact of dating accuracy on our study conclusions is

very limited. Moreover, although the X3 $\delta^{18}O_s$ record has a high dating error, its trend in period IV (Figure 16a) is highly consistent with the MAW, CH, and SEY $\delta^{18}O_s$ records, further confirming the comparability of the $\delta^{18}O_s$ records in our study.

It should be noted that, given that the $\delta^{18}O_s$ record used in our study were all derived from published studies and our study objective was not to reconstruct paleoclimate, we decided not to include the detailed dating analysis results in the manuscript or Supplementary Information.

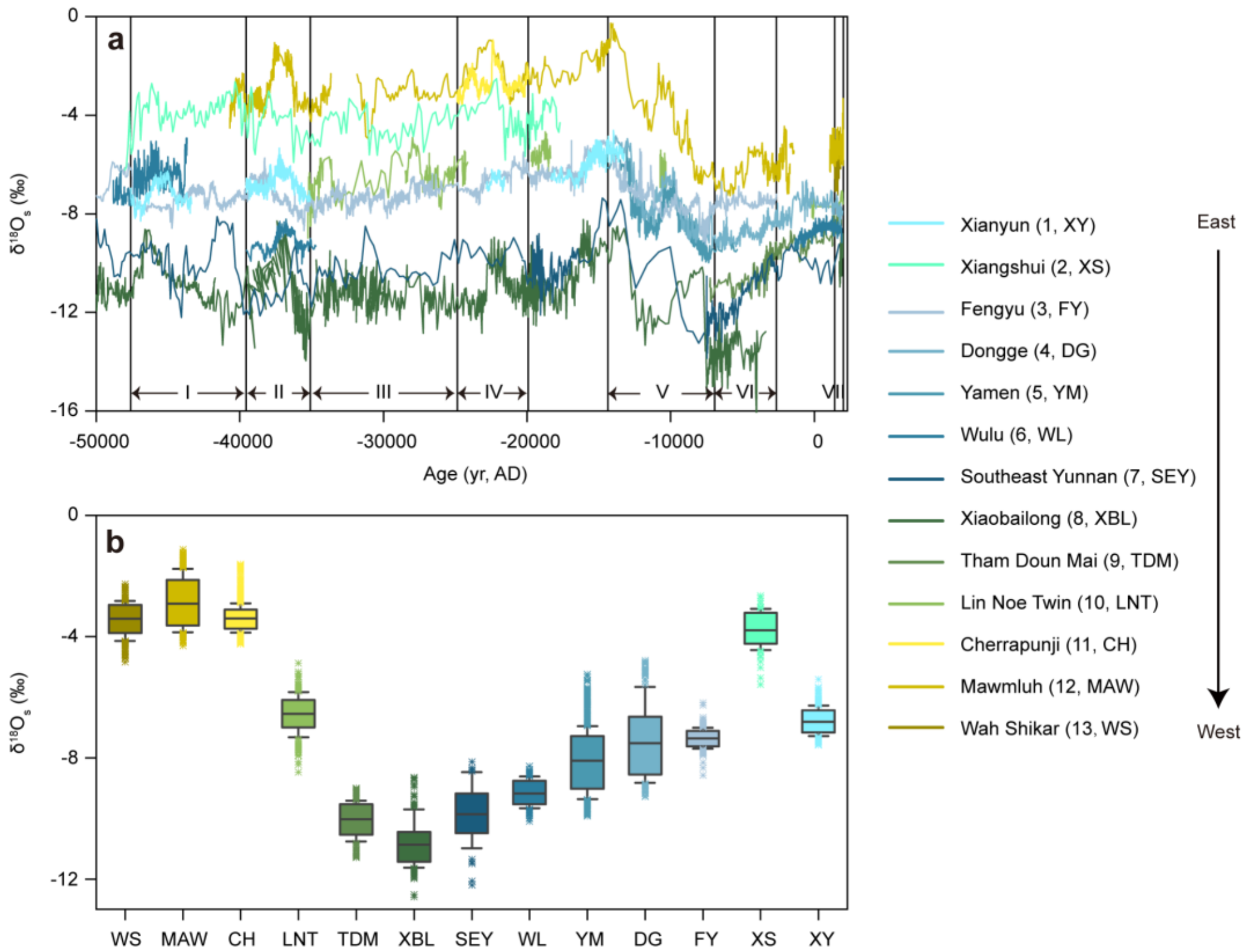

Figure R16. Comparisons of the $\delta^{18}O_s$ records from 13 caves along the transect during the last 50,000 years. a $\delta^{18}O_s$ records from the 13 caves along the transect. Vertical lines indicate the dividing line of each sub-period. b Spatial distribution of multi-year mean $\delta^{18}O_s$ values from the 13 caves, calculated using a “relative value” method (see Materials and Methods). In each box plot, the box represents the 20th–80th percentile range, whiskers extend to the 10th–90th percentiles, horizontal lines inside the boxes indicate the mean, and stars represent outliers. Numbers in brackets correspond to the open circles in Fig. 1a. $\delta^{18}O_s$ values are reported as per mil (‰) deviations from Vienna Peedee belemnite (VPDB).

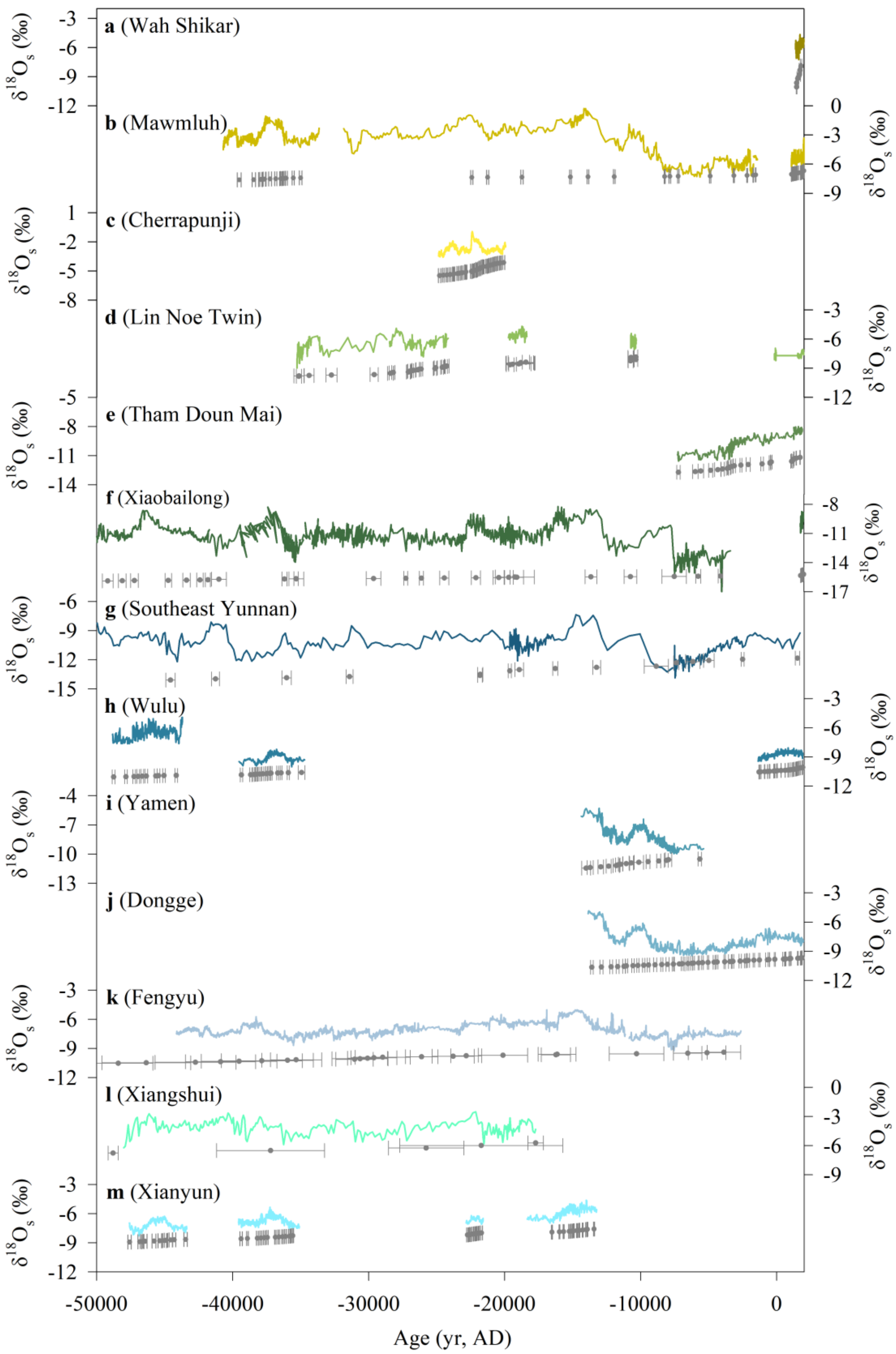

Figure R17. U-Th ages for the $\delta^{18}O_s$ records from 13 caves along the transect during the last 50,000 years. $\delta^{18}O_s$ values are reported as per mil (‰) deviations from Vienna Peedee belemnite (VPDB).

Reviewer #2 (Remarks to the Author):

I co-reviewed this manuscript with one of the reviewers who provided the listed reports. This is part of the Nature Communications initiative to facilitate training in peer review and to provide appropriate recognition for Early Career Researchers who co-review manuscripts.

Re: We thank you very much for providing very fair and constructive comments and suggestions on our manuscript. Your comments and suggestions have significantly improved the quality of our manuscript. We have tried to address all the concerns raised by you and have carefully revised the manuscript. Please see our response as mentioned above.

Reviewer #3 (Remarks to the Author):

Speleothem $\delta^{18}O$ records across the Indian and Asian monsoon domain have been frequently used to study past climatic changes. However, numerous interpretations have been put forward to describe their dominant controls, and therefore, the metric of climate change these records actually represent has remained somewhat unclear. Zhang et al. provide a revised interpretation of these based on a zonal analysis of modern rainfall $\delta^{18}O$ as well as speleothem records $\delta^{18}O$ distributed across the Asian monsoon domain. The authors suggest that updraft in the central portion of their zonal area explains a V-shaped pattern in rainfall $\delta^{18}O$ values during the monsoon season. Box plotting speleothem $\delta^{18}O$ values distributed on this zonal transect, they observe a similar pattern and suggest that updraft explains the zonal variation of speleothem $\delta^{18}O$ values.

I have read the manuscript a few times. This has clearly been an ambitious and large body of work, and I find this research would be of broad interest and provocative. I do have several changes, further considerations and recommendations for additional analysis to be conducted prior to publication. The text may also need to be improved for clarity. However, despite having written a lot, I think many of my concerns are asking for further clarification and quantification to ensure the statistical robustness of the findings, and these changes should be achievable. After this, I believe the work would make an interesting contribution to understanding the variable influences on rainfall (and speleothem) $\delta^{18}O$ values.

Re: Thank you very much for providing very fair, positive, and encouraging comments. Your comments and suggestions greatly improve the quality of our manuscript. We have tried to address all the concerns raised by you and have carefully revised the manuscript as follows.

I have three larger concerns, which are the primary points I would like the authors to address.

Firstly, the authors seem to have used data differently throughout the manuscript. For example, on Fig. 2, we see that not all the sites in the pre-monsoon season are represented with $\delta^{18}O$ values. Likewise, the gradients Fig. 4 (c,d,e,f) use different sites to construct each gradient, which means the slope analysis (Fig. 4g) is impacted

by these choices, rather than solely the spatial variation across this whole transect. So, perhaps the authors might consider refocusing their analysis on the mid-section to the Eastern section, where they seem to have sufficient data? Instead, they may need to soften their “low in the East, high in the West”, “V-shape” and “westward migrations” claims, or rely more heavily on the modelling data to supplement these findings by bringing them into the main text/figure set up.

Re: Thank you very much for pointing these issues out. We have tried to address all the issues as follows:

1) Firstly, we admit that the observational $\delta^{18}O_p$ data are insufficient in the westernmost sites, especially during the pre-monsoon season. Of course, when we use IsoGSM2 model $\delta^{18}O_p$ data with a longer time scale (1979-2022), the results show that the $\delta^{18}O_p$ values in the western domain of the South Asian monsoon region are relatively high (Supplementary Fig. 6), which is consistent with our finding of lower $\delta^{18}O_p$ values in the eastern domain during the pre-monsoon season. However, we do not intend to rely more heavily on the modelling data to supplement these findings by bringing them into the main text/figure set up. It is because that we want readers to be able to see that we still mainly rely on the observed data.

It should be noted that in Fig. 2a-d of our manuscript, we focused on the pre-monsoon season. During this season, we observed a westward migration of low $\delta^{18}O_p$ values from the easternmost site of the East Asian monsoon region toward the dividing line (~98° E) (i.e., the middle of the transect). Based on subsequent analyses, we find that the combined influence of the ITCZ, IPWP, and WPSH drives this gradual westward migrate of low $\delta^{18}O_p$ values from February to May. However, this influence does not extend to the westernmost site of the South Asian monsoon region (i.e., the westernmost site of the transect). Therefore, the absence of $\delta^{18}O_p$ data from sites in the western domain of the South Asian monsoon region during February–May does not affect our conclusions.

Figures 4c and 4d were designed to further validate the gradually westward migration of low $\delta^{18}O_p$ values from the easternmost site of the East Asian monsoon region toward the dividing line (~98°E; i.e., the middle of the transect) during the pre-monsoon season. Therefore, we conducted a gradient analysis from the easternmost site towards the dividing line (~98° E) (that is, from Luoyuan to

Kunming transect) from February to May.

In contrast, Figures 4e and 4f were designed to further validate that the pre-monsoon season pattern shifted to the V-shaped pattern during the monsoon season, which requires focusing on the monsoon season. Our study found that the lower values of $\delta^{18}O_p$ and OLR are appeared around the middle of the transect, resulting in a V-shaped pattern along the transect during the monsoon season. Consequently, $\delta^{18}O_p$ and OLR values increase from the middle to the easternmost site (Luoyuan). This implies that the gradients (slopes) of $\delta^{18}O_p$ and OLR from the middle to Luoyuan should be positive values. That is, if the slopes of $\delta^{18}O_p$ and OLR from a specific site in the middle to Luoyuan are negative values, it suggests that the lower $\delta^{18}O_p$ and OLR values do not extend westward to the specific site. In this case, we should focus on the "critical site" which is most adjacent to the east of the specific site. Clearly, the "critical site" was characterized by lower $\delta^{18}O_p$ and OLR values. Our analysis found that in June the lower $\delta^{18}O_p$ and OLR values appear around Baoshan ("critical site") in the middle of the transect. Then the "critical site" subsequently migrates westward to Tengchong, Ruili, and Sylhet from July to September, respectively. Therefore, gradient analysis can only be conducted from Baoshan in June, Tengchong in July, Ruili in August, and Sylhet in September to the easternmost site Luoyuan, respectively.

In summary, the difference in data used across different sections is because that the study purposes are different.

2) Following the comments provided by you and both reviewers #1 and #2, for the pre-monsoon season, we changed the statement "low in the east, high in the west" to "*$\delta^{18}O_p$ values follow a pattern of lower values in the east and higher values in the west of the ~98° E dividing line*." Please see L147–L148 in the revised manuscript. In addition, we changed the statement "migrates westward" to *"migrate toward the dividing line*." Please see L149 in the revised manuscript.

For the monsoon season, following the comments provided by you and both reviewers #1 and #2, we further clearly describe our findings. i.e., we changd "During the monsoon season, $\delta^{18}O_p$ values are lower than during the pre-monsoon season, with the low $\delta^{18}O_p$ zone extending further westward to the middle of the transect from June to

September” to “*During the monsoon season, $\delta^{18}O_p$ values are lower than during the pre-monsoon season, with the lower $\delta^{18}O_p$ zone extending westward to Baoshan and Tengchong, and even to the west of the dividing line (Ruili and Sylhet) from June to September.*” Please see L153–L155 in the revised manuscript. We also marked some horizontal arrows on Figure 2 to indicate that the low $\delta^{18}O_p$ zone migrates westward. At the same time, we used different colors for the arrows to represent the different low $\delta^{18}O_p$ values during the pre-monsoon and monsoon seasons. Please see Fig. 2 in the revised manuscript.

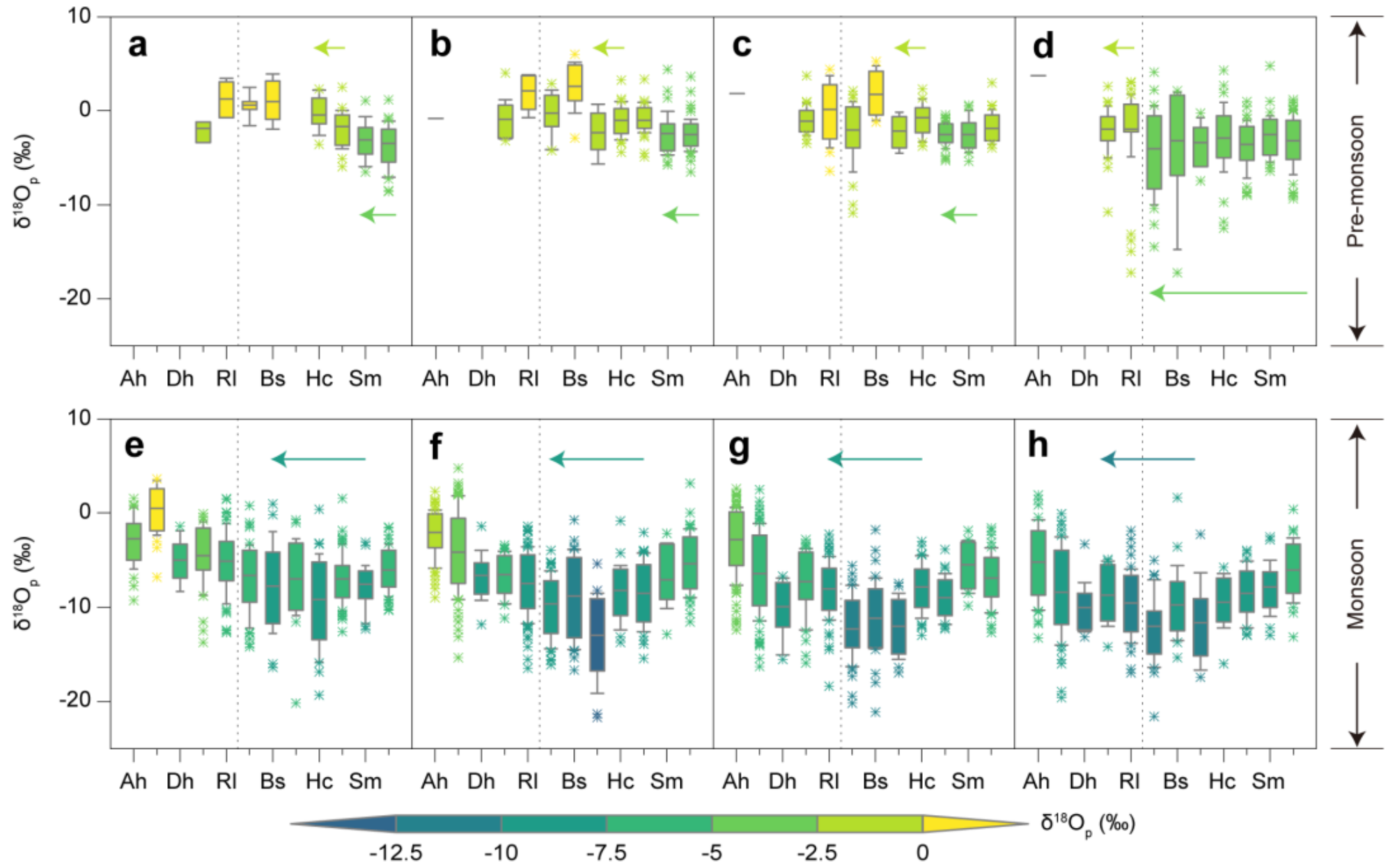

Figure R18. Spatial distributions of $\delta^{18}O_p$ at the 12 primary sites along the transect during the pre-monsoon and monsoon seasons. a–d $\delta^{18}O_p$ distributions during the pre-monsoon season: February (a), March (b), April (c), and May (d). e–h $\delta^{18}O_p$ distributions during the monsoon season: June (e), July (f), August (g), and September (h). Abbreviations: Ah (Ahmedabad), Dh (Dhanbad), Rl (Ruili), Bs (Baoshan), Hc (Hechi), and Sm (Sanming). In each box plot, the box represents the 20th–80th percentile range, whiskers extend to the 10th–90th percentiles, horizontal lines inside the boxes indicate the mean, and stars represent outliers. Horizontal arrows highlight the westward extension of the low $\delta^{18}O_p$ zone. Dotted lines mark the dividing line of the eastern and western domains. $\delta^{18}O_p$ values are expressed as per mil (‰) deviations relative to VSMOW or VSMOW2.

Secondly, at current, I think the authors require further work to validate the application to the speleothem record. This is in part because the most convincing match to their precipitation data (Fig. 6b) hasn't been explained in sufficient detail in the main text or methods to adequately understand how these "multie-year" and relative values were generated. Furthermore, as I've stated in my section comments, the proposed trend in Fig. S21 requires additional statistical work to show 1) it is consistent with the regional $\delta^{18}O_p$ trend, and 2) matches the multi-year mean $\delta^{18}O_s$ or the regional precipitation data (Fig. 2).

Re: We thank you for pointing these issues out. We have tried to address all the issues as follows:

1) Following your suggestions, we added four paragraphs in the "Materials and Methods" section to provide detailed explanations on how these "multi-year mean $\delta^{18}O_s$ values" are calculated. i.e.,

"***Calculation of multi-year mean $\delta^{18}O_s$ values***

The $\delta^{18}O_s$ records from the 13 caves analyzed in this study span different time periods over the last 50,000 years (Fig. 7a). To more clearly compare $\delta^{18}O_s$ records for assessing spatial patterns, we calculated the multi-year mean $\delta^{18}O_s$ value for each cave using a "relative value" method. To calculate the "relative" $\delta^{18}O_s$ values as accurately as possible, we divided the $\delta^{18}O_s$ records into seven sub-periods (Periods I –VII) (Fig. 7a) following two criteria: (1) we ensured that as many $\delta^{18}O_s$ records as possible were available for each sub-period; and (2) all our grouped sub-periods were designed to cover the entire period (the past 50,000 years) as comprehensively as possible.

Based on the distribution characteristics of $\delta^{18}O_s$ records over the past 50,000 years, we calculated the multi-year mean $\delta^{18}O_s$ values for each record within their respective sub-periods: Period I (Fengyu, Xiangshui, Xiaobailong, and Southeast Yunnan), Period II (Mawmluh, Wulu, and Xianyun), Period III (Lin Noe Twin), Period IV (Cherrapunji), Period V (Yamen and Dongge), Period VI (Tham Doun Mai), and Period VII (Wah Shikar). We used the Fengyu $\delta^{18}O_s$ record as a reference, as it provided the longest continuous series (Fig. 7a). For Period I, we calculated the multi-year mean $\delta^{18}O_s$ values directly by using the $\delta^{18}O_s$ records from Fengyu, Xiangshui, Xiaobailong, and Southeast Yunnan caves. For Periods II –VI, we used the

multi-year mean $\delta^{18}O_s$ value from Fengyu Cave in Period I as the base value. The respective $\Delta\delta^{18}O_s$ values for Fengyu cave in Periods II –VI were then calculated by subtracting the base value from the respective Fengyu $\delta^{18}O_s$ records during Periods II-VI, respectively. Obviously, the $\Delta\delta^{18}O_s$ values for Fengyu cave are different in Periods II-VI. Finally, we obtained the multi-year mean $\delta^{18}O_s$ values during Periods II-VI by subtracting the corresponding $\Delta\delta^{18}O_s$ value in each period from the corresponding $\delta^{18}O_s$ records in each period, respectively.

For example, to calculate the multi-year mean $\delta^{18}O_s$ values for the three specific $\delta^{18}O_s$ records in Period II (Mawmluh, Wulu, and Xianyun), we first determined the Fengyu $\Delta\delta^{18}O_s$ value (0.07‰) in Period II by subtracting the Fengyu multi-year mean $\delta^{18}O_s$ value (-7.36‰) in Period I from the Fengyu multi-year mean $\delta^{18}O_s$ value (-7.29‰) in Period II. Finally, we obtained the multi-year mean $\delta^{18}O_s$ values for the Mawmluh, Wulu, and Xianyun caves by subtracting the $\Delta\delta^{18}O_s$ values (0.07‰) from the Mawmluh, Wulu, and Xianyun $\delta^{18}O_s$ records in Period II, respectively.

Notably, the Fengyu $\delta^{18}O_s$ record does not extend into Period VII, where the Wah Shikar $\delta^{18}O_s$ record is the only available dataset (Fig. 7a). For this sub-period, we calculated the multi-year mean $\delta^{18}O_s$ value for Wah Shikar cave using the Mawmluh $\delta^{18}O_s$ record as a reference, given its closer geographic proximity. This approach allowed us to derive relative $\delta^{18}O_s$ values and compute multi-year mean $\delta^{18}O_s$ values for each cave." Please see L774–L811 in the revised manuscript.

2) It is very difficult to establish a correlation between the V-shaped patterns of $\delta^{18}O_p$ and that of $\delta^{18}O_s$, because that the geographical locations of the $\delta^{18}O_p$ sites and $\delta^{18}O_s$ -recording caves do not correspond one-to-one. To verify the application of our findings to speleothem records, we followed your suggestion and added a figure to show the trends of the multi-year mean $\delta^{18}O_p$ and $\delta^{18}O_s$ values from the west to the middle and from the middle to the east of the transect (Figure R19). From this figure, we can compare the spatial distribution of observed $\delta^{18}O_p$ data from 12 primary sites with the spatial distribution of observed $\delta^{18}O_p$ data from all 46 sites along the east-west transect, and with the spatial distribution of multi-year mean $\delta^{18}O_s$ values. We found that: a) The locations of the lowest values of the three V-shaped patterns appear at the similar longitude (~103°E). b) West of 103°E, both $\delta^{18}O_p$ and $\delta^{18}O_s$ show a significant negative correlation with longitude; east of 103°E, both $\delta^{18}O_p$ and

$\delta^{18}O_s$ show a significant positive correlation with longitude. These results indicate that the V-shaped pattern of $\delta^{18}O_s$ matches that of $\delta^{18}O_p$. To address this issue, we add a paragraph, i.e., "*To further verify the implications for interpreting Asian $\delta^{18}O_s$ records, we analyzed the spatial trends of the multi-year mean $\delta^{18}O_s$ values and the annual $\delta^{18}O_p$ observation data from 12 primary sites and all 46 sites along the transect. Our data indicate that the lowest values of all three V-shaped patterns appear at a similar longitude (~103°E; Supplementary Fig. 20). Moreover, west (east) of 103°E, the decreases (increases) in the $\delta^{18}O_s$ and $\delta^{18}O_p$ values with increasing longitude are significant (Supplementary Fig. 20).*" Please see L416–L422 in the revised manuscript. Please see the Fig. S20 in the revised Supplementary Information.

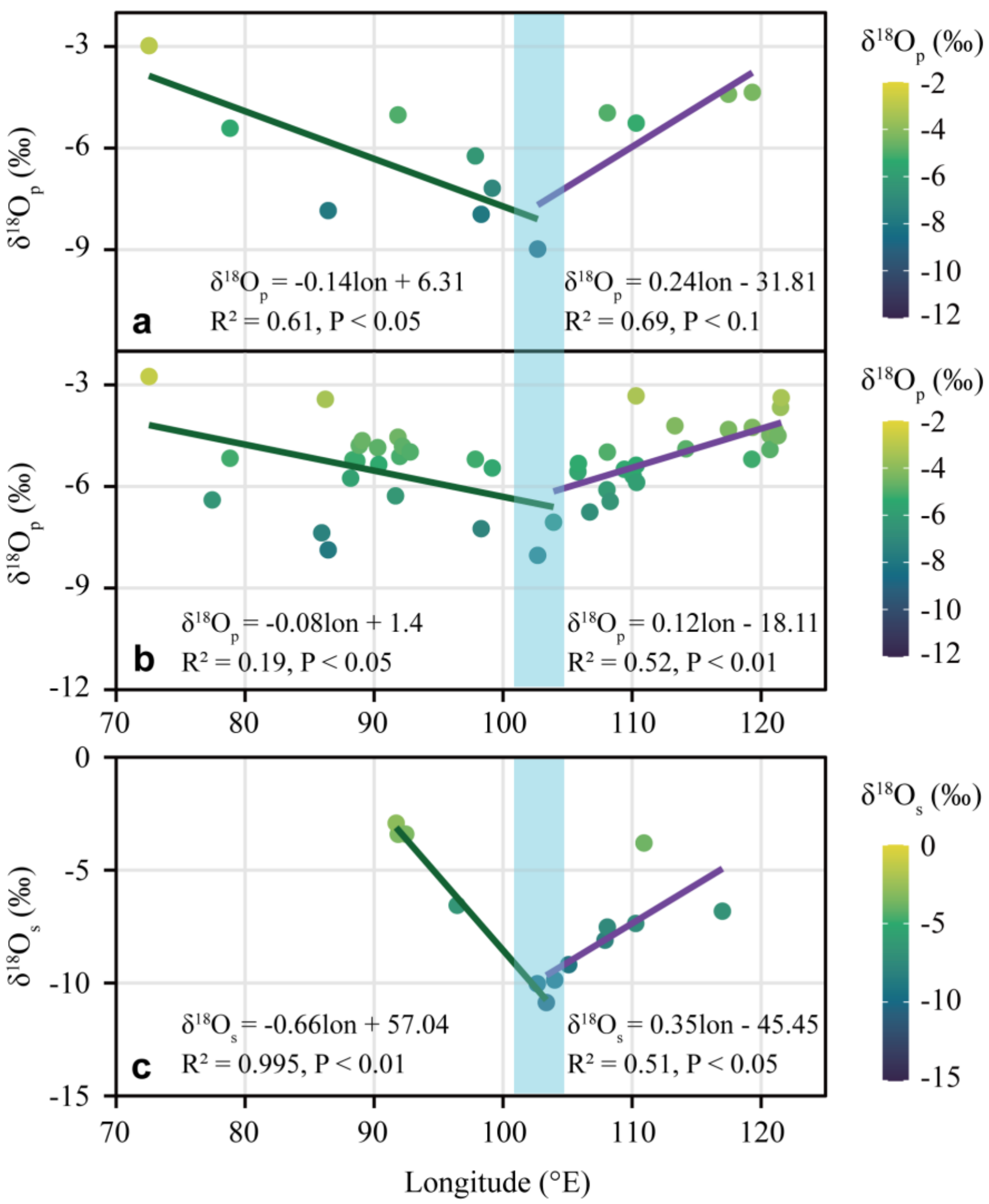

Figure R19. Trends of the multi-year mean $\delta^{18}O_p$ and $\delta^{18}O_s$ values from the west to the middle and from the middle to the east of the transect. A, The 12 primary precipitation sampling sites. b, The 46 precipitation sampling sites across the study region. c, The 13 caves along the transect. Blue-green shading indicates the location

of the lowest $\delta^{18}O$ value. $\delta^{18}O_p$ values are expressed as per mil (‰) deviations relative to VSMOW or VSMOW2. $\delta^{18}O_s$ values are reported as per mil (‰) deviations from Vienna Peedee belemnite (VPDB).

3) Additionally, following your suggestion, we marked the locations of all 13 caves on the original Fig. S22 and labeled them with their corresponding multi-year mean $\delta^{18}O_s$ values (Figure R20). The results demonstrate that the spatial pattern of multi-year mean $\delta^{18}O_s$ values matches well with the that of the IsoGSM2 simulated $\delta^{18}O_p$ along this transect. To address this issue, we added corresponding explanations, i.e., "*Additionally, the spatial distribution of multi-year mean $\delta^{18}O_s$ values closely resembles the annual IsoGSM2–simulated $\delta^{18}O_p$ values (1979–2022), both exhibiting a consistent V-shaped pattern (Supplementary Fig. 21).*" Please see L422–L425 in the revised manuscript. Please see the new Fig. S21 in the revised Supplementary Information.

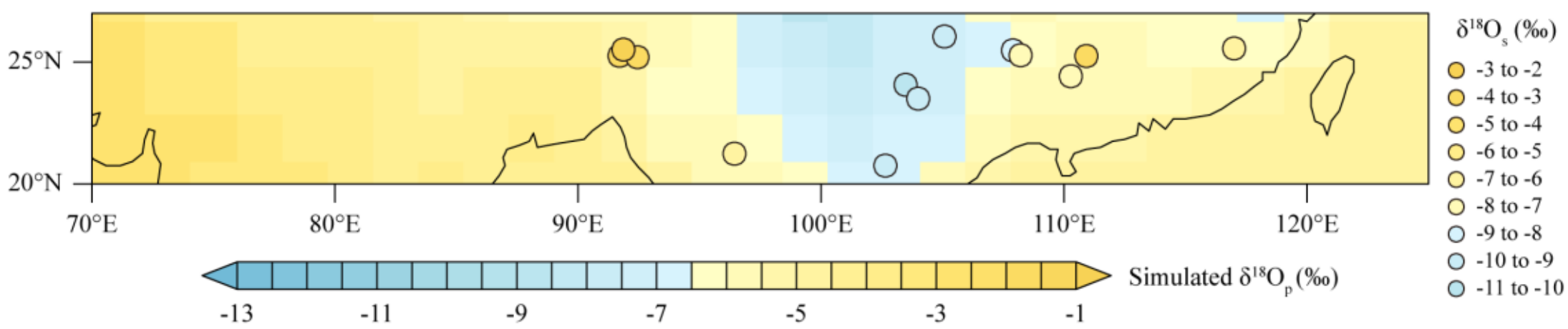

Figure R20. Spatial distribution of IsoGSM2-simulated annual $\delta^{18}O_p$ across the study region over 1979–2022. Multi-year mean $\delta^{18}O_s$ values from 13 caves (dot) across the study region are also shown. $\delta^{18}O_p$ values are expressed as per mil (‰) deviations relative to VSMOW/VSMOW2. $\delta^{18}O_s$ values are reported as per mil (‰) deviations from Vienna Peedee belemnite (VPDB).

Thirdly, I think the manuscript could be improved by increasing the analysis to more traditional climate variables (monthly precipitation amount and temperature). The authors could consider a more robust multiple variable test through multiple regression analysis, including these and OLR on monthly $\delta^{18}O$. The authors state (in the methods) that they have already collected additional variables, but these don't seem to have been included beyond this. I think bringing them into the analysis would provide a more-rounded approach to understand the spatial variability of $\delta^{18}O_p$ in the region. If they do not have any significant impact on $\delta^{18}O_p$, then this information

could be shown as a supplement.

Re: We thank you for pointing these issues out. We addressed these issues as follows:

1) With regard to the additional analysis of more traditional climate variables (temperature and precipitation amount):

a) With regard to "temperature", according to the study of Dansgaard (1964), we know that the temperature effect (characterized by a positive correlation between $\delta^{18}O_p$ and temperature) mainly appears in mid and high latitudes. However, when discuss the variations in $\delta^{18}O_p$ across the Asian monsoon region (particularly in our study region), few studies discussed the relationship between $\delta^{18}O_p$ and temperature, because the $\delta^{18}O_p$ at most sites have an abnormal negative correlation with temperature, which conflicts with the "temperature effect." Clearly, this is another topic (story). Indeed, as early as 2021, we specifically conducted study on this abnormal negative correlation in the Asian monsoon region and defined it as the "inverse temperature effect" (Yu et al., 2021). Given the above reasons, this study does not intend to repeat the discussion on the impact of temperature on the $\delta^{18}O_p$.

b) With regard to "precipitation amount", according to the study of Dansgaard (1964), the precipitation "amount effect" (characterized by a negative correlation between $\delta^{18}O_p$ and precipitation amount) mainly occurs in low latitudes, especially in tropical regions. Since the following reasons, in this study we eliminated the influence of precipitation amount on $\delta^{18}O_p$.

i) Numerous previous studies have already discussed the relationship between $\delta^{18}O_p$ and precipitation amount. If we were to further analyze the relationship between $\delta^{18}O_p$ and precipitation amount in this study, it would seem somewhat repetitive and would seriously undermine the novelty of this study.

ii) Previous paleoclimate studies have shown that the Asian $\delta^{18}O_s$ records exhibit a remarkable spatial consistency across different timescales during the Holocene (Chen et al., 2016; Liu et al., 2015). However, there are significant spatial differences in precipitation amount at different time scales in East Asia, and there is a clear conflict between the $\delta^{18}O_s$ and precipitation amount. Consequently, further discussion of precipitation as a climatic variable for interpreting $\delta^{18}O_s$ record would be unhelpful. This is why our study early eliminated precipitation amount as a potential factor in the

Introduction section.

iii) Aggarwal et al. (2016) demonstrated that the precipitation "amount effect" is extremely weak at multi-year monthly average scale in low-latitude regions (the negative correlation is very weak, when the precipitation ⩾ 300 mm, there is an anomalous positive correlation) (Figure. R21). This implies that further discussion of precipitation amount effect would be of little significance.

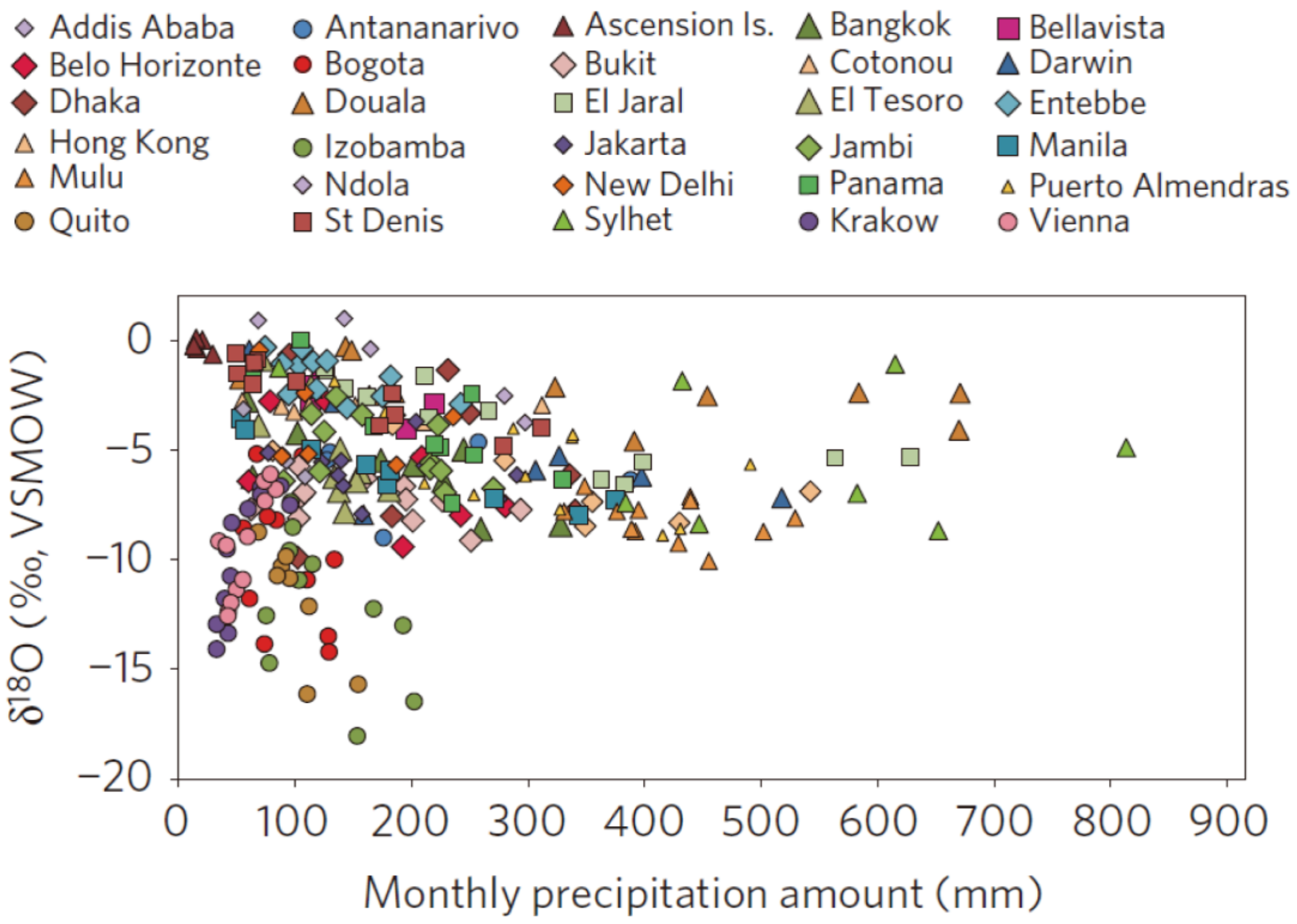

Figure R21. Correlation of mean monthly $\delta^{18}O$ and precipitation amount in tropical and midlatitude precipitation. Note the limited change in $\delta^{18}O$ with the highest precipitation amounts.

iv) More importantly, we used a unique perspective provided by you to eliminate the influence of precipitation amount. Specifically, following your suggestions, we analyzed the spatial pattern of precipitation amount along the transect during the monsoon season. As we propose that $\delta^{18}O_p$ values along the transect show a "V-shaped" pattern, that is, higher $\delta^{18}O_p$ values at the eastern and western margins and lower values in the middle. If precipitation amount affects the V-shaped pattern of $\delta^{18}O_p$ along the east–west transect, precipitation amount along the transect should appear as an "inverted V-shaped" pattern during the monsoon season. However, our

precipitation amount data do not show an "inverted V-shaped" pattern (Figure R22), which can not explain the V-shaped pattern of $\delta^{18}O_p$. More important, a notable discrepancy was observed at the Sylhet site, where monthly precipitation amount is exceptionally high, yet the $\delta^{18}O_p$ at this site did not exhibit the correspondingly expected low values (Figure R22). Instead, the $\delta^{18}O_p$ at this site remained relatively high. This result conflicted with the theory of precipitation "amount effect." Therefore, it can be seen that the spatial pattern of precipitation amount is inconsistent with that of the $\delta^{18}O_p$ along the transect. Thus, we can conclude that the $\delta^{18}O_p$ along the transect shows little correlation with the precipitation amount. We added a figure to show the spatial pattern of precipitation amount along the east–west transect (Please see Figure R22 or Fig. S15 in the revised Supplementary Information), and we added a paragraph in the manuscript to eliminate the influence of precipitation amount on $\delta^{18}O_p$ along the transect. i.e., "*It could also be proposed that terrain-enhanced updrafts might increase precipitation, linking $\delta^{18}O_p$ to precipitation amount. If true, monsoon-season precipitation should show an inverted V-shaped pattern along the transect. However, our results do not support this hypothesis as precipitation amount in the middle is relatively low while it is considerably higher at Sylhet in the western domain (Supplementary Fig. 15).*" Please see Fig. S15 in the revised Supplementary Information and L320–L325 in the revised manuscript.

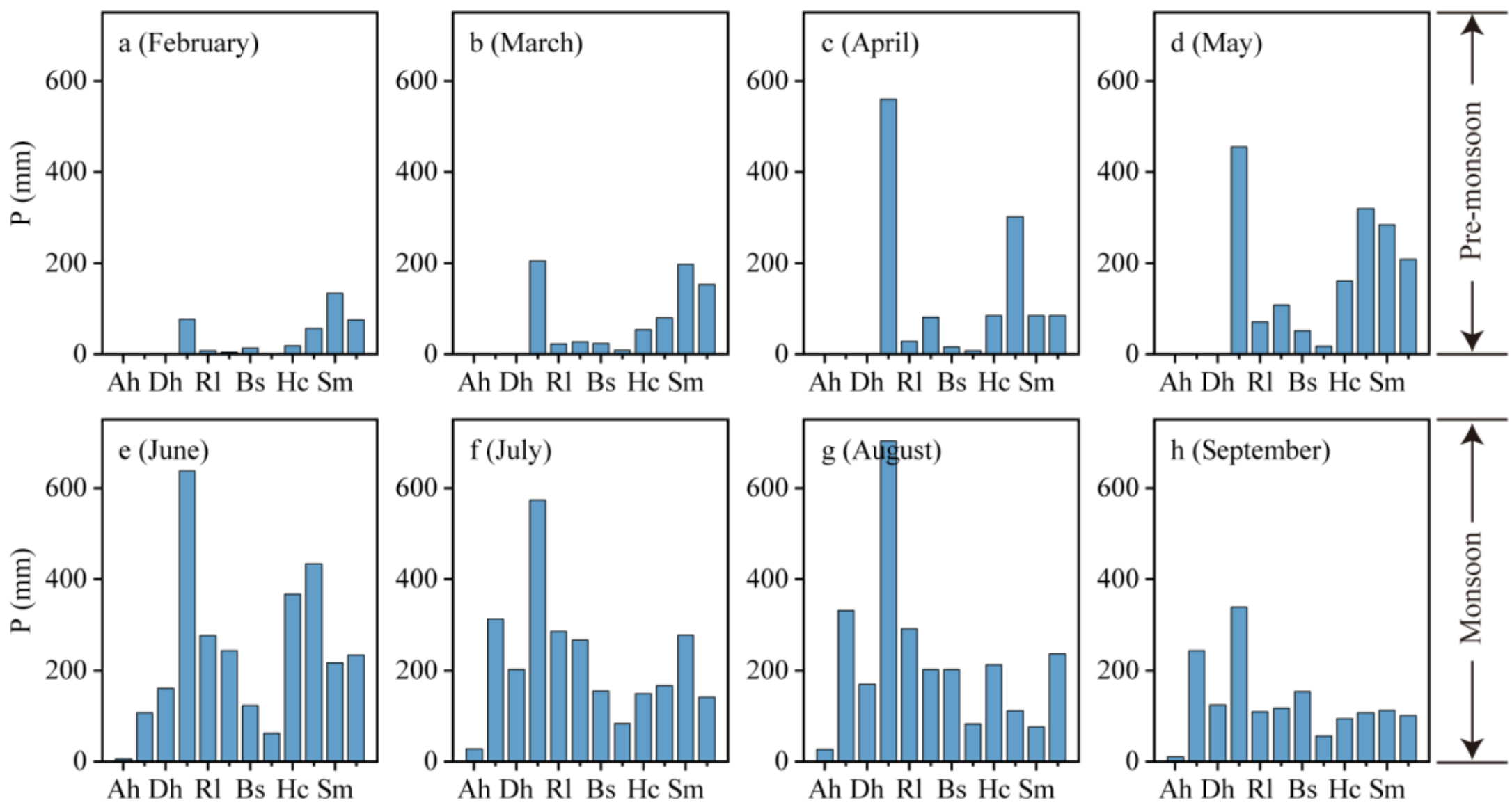

Figure R22. Spatial distributions of precipitation amount at the 12 primary sites along the transect during the pre-monsoon and monsoon seasons. a–d, Pre-monsoon season. e–h, Monsoon season. Abbreviations: Ah (Ahmedabad), Dh (Dhanbad), Rl (Ruili), Bs

(Baoshan), Hc (Hechi), and Sm (Sanming).

c) With regard to the comment of "a more robust multiple variable test through multiple regression analysis, including these and OLR on monthly $\delta^{18}O$", if we use the multiple regression method (including variables such as OLR, precipitation amount, and temperature), this would weaken our primary focus. The main purpose of this study is to highlight the role of atmospheric updrafts in the vertical direction. Moreover, based on the above analysis, we have already eliminated temperature and precipitation amount as a key factor. So we do not intend to include them in the multiple regression method.

2) With regard to not included other variables, it should be noted that the additional variables collected in our study are not used to discuss their relationships with $\delta^{18}O_p$, but used to delineate the Intertropical Convergence Zone (ITCZ) and other things. Following your comment, we added two paragraphs in the "Materials and Methods" section to explain the utility of other variables in our study: "*These precipitation amount data were used to analyze the proportions of precipitation during the different seasons to the annual precipitation and to analyze the spatial distributions of precipitation amount during the pre-monsoon and monsoon seasons.*

The study utilized the ERA5 reanalysis data (0.25°×0.25° resolution) provided by the European Centre for Medium-Range Weather Forecasts (ECMWF). The ERA5 reanalysis data on single levels are available from https://cds.climate.copernicus.eu/cdsapp#!/dataset/reanalysis-era5-single-levels-monthly-means?tab=form, following ref. ***Error! Reference source not found.****. The ERA5 reanalysis data on pressure levels are available from https://cds.climate.copernicus.eu/cdsapp#!/dataset/reanalysis-era5-pressure-levels-monthly-means?tab=form, following ref.* ***Error! Reference source not found.****. Among them, vertical velocity (ω) and outgoing longwave radiation (OLR) were used to quantify the intensity of atmospheric updraft (negative and positive ω values indicate upward and downward motions, respectively). The lower the ω and OLR, the stronger the atmospheric updraft. We used specific humidity combined with meridional and zonal winds to calculate the moisture flux. The geopotential height was used to assess the western Pacific subtropical high intensity. Precipitation and cloud cover were*

*used to delineate the Intertropical Convergence Zone (ITCZ). In addition, sea surface temperature (SST) data (1°×1° resolution) provided by HadISST1 (https://www.metoffice.gov.uk/hadobs/hadisst/)****Error! Reference source not found.*** *were used to evaluate Indo-Pacific Warm Pool (IPWP) variability.*" Please see L734–L755 in the revised manuscript.

References:

Aggarwal, P. K. et al. Proportions of convective and stratiform precipitation revealed in water isotope ratios. *Nat. Geosci.* **9**, 624–629 (2016).

Chen, J. et al. On the timing of the East Asian summer monsoon maximum during the Holocene—Does the speleothem oxygen isotope record reflect monsoon rainfall variability? *Sci. China Earth Sci.* **59**, 2328–2338 (2016).

Dansgaard, W. Stable isotopes in precipitation. *Tellus* **16**, 436–468 (1964).

Liu, J. et al. Holocene East Asian summer monsoon records in northern China and their inconsistency with Chinese stalagmite δ18O records. *Earth Sci. Rev.* **148**, 194–208 (2015).

Yu, W. et al. Temperature signals of ice core and speleothem isotopic records from Asian monsoon region as indicated by precipitation δ^{18}O. *Earth Planet. Sci. Lett.* **554**, 116665 (2021).

Comments on sections

Seasonal variations in δ18O

Eastern sites vs. western sites trend

The authors argue that there is a difference in the seasonal δ^{18}O trends between eastern and western regions. Namely, that $\delta^{18}O_p$ declines from May to July in the east, and declines from May to October in the west. The westernmost sites do not cover the whole year. Is this because it is too dry? Having a faded bar chart of rainfall amount behind each boxplot (Fig. 1) would be helpful to understand the distribution of monthly rainfall at these sites.

Re: We thank you for pointing this issue out. Indeed, the westernmost sites do not cover the whole year. Yes, this is because it is too dry. Following the comments provided by Reviewers #1 and #2, we changed Figure 1 to a new type figure. In the updated Figure 1, we cannot add monthly precipitation amount. So we added monthly

precipitation amount in Figure S3 (seasonal variations of IsoGSM2 simulated $\delta^{18}O_p$ at the 12 primary sites) (Figure. R23). Please see Fig. S4 in the revised Supplementary Information.

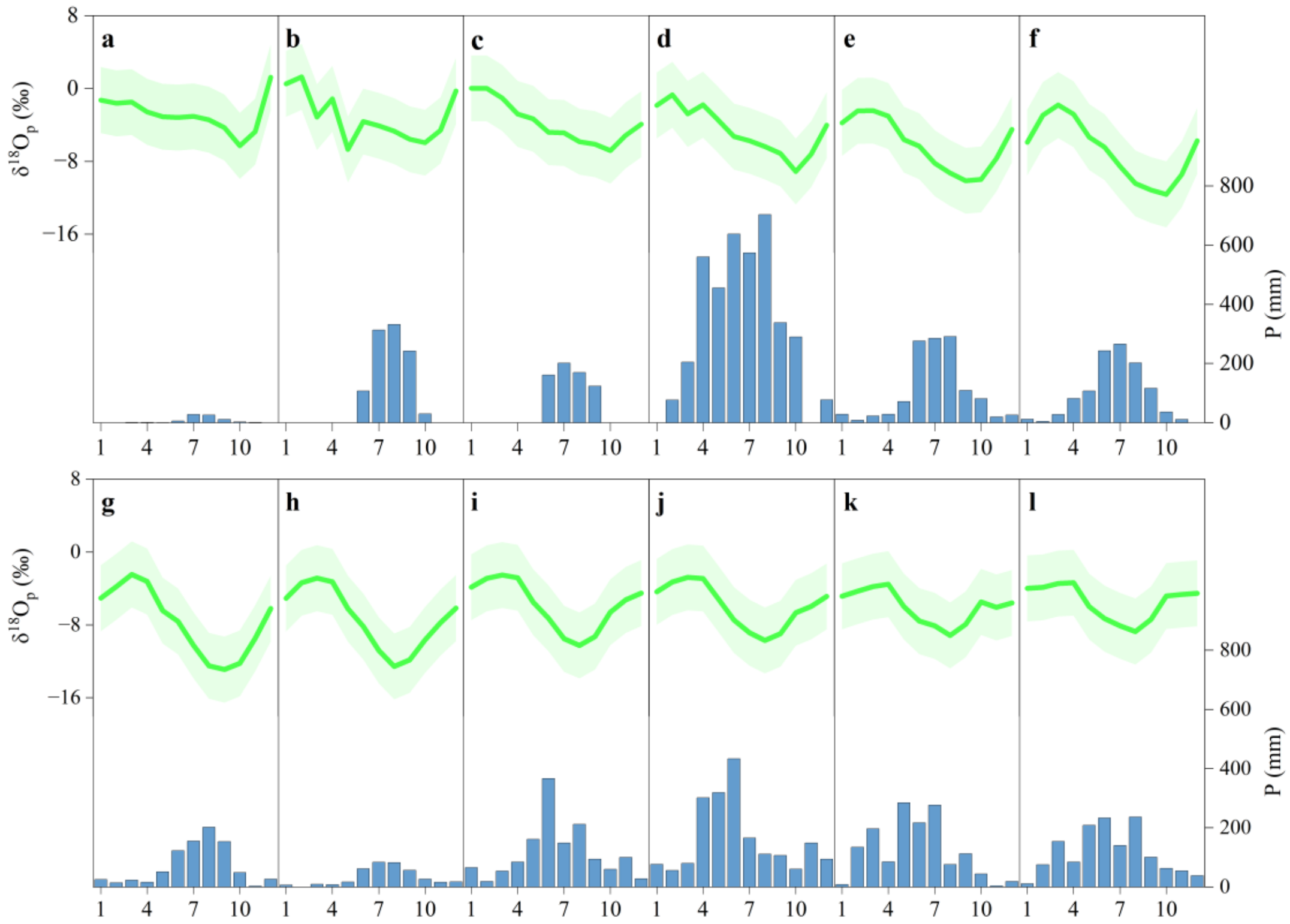

Figure R23. Seasonal variations of IsoGSM2-simulated $\delta^{18}O_p$ and observed precipitation amount at the 12 primary sites along the east–west transect over 1979–2022. The 12 primary sites, arranged from west to east, include Ahmedabad (a), Sagar (b), Dhanbad (c), Sylhet (d), Ruili (e), Tengchong (f), Baoshan (g), Kunming (h), Hechi (i), Institute of Karst Geology (j), Sanming (k), and Luoyuan (l). $\delta^{18}O_p$ values are expressed as per mil (‰) deviations relative to VSMOW or VSMOW2.

Spatial distribution of $\delta^{18}O$

The altitude effect

The authors aim to discount the altitude effect to show that this is not a dominant control on rainfall $\delta^{18}O$. They claim that there is no significant effect of altitude on rainfall $\delta^{18}O$ by performing regressions on data binned into 3 climate-based categories. However, the delineations seem to be based on altitude; as such, there's no reason not to do the regression over the whole dataset (in other words, the regression needs to be conducted without binning the data). If there is still no significant effect, then the

authors are correct. If not, then the altitude effect does impact an aspect of the spatial distribution of $\delta^{18}O_p$, and this needs to be accounted for by the authors. If this is the case, it is not inherently a problem; the authors have shown that rainfall $\delta^{18}O$ varies seasonally across the transect. However, the authors must simply conduct their subsequent analysis, including altitude as a metric (in a multiple regression, for example), or find another way to account for its effect.

If the authors intend to keep climate-based binned data, I suggest they use a system that does not explicitly include altitude to separate the sites (such as the Köppen-Geiger climate classification). However, as I wrote in the last paragraph, I do not think a significant regression with altitude across the whole dataset affects their seasonal component, and therefore, this would be a more preferable approach.

Re: We thank you for pointing these issues out. We addressed these issues as follows:

1) First, following your suggestion, we conducted a regression analysis on the entire dataset (in other words, the regression was conducted without binning the data). The result showed that there is indeed a significant negative correlation between the $\delta^{18}O_p$ and altitude. However, we have the ability to prove that the significant negative correlation mentioned above is a "false" altitude effect.

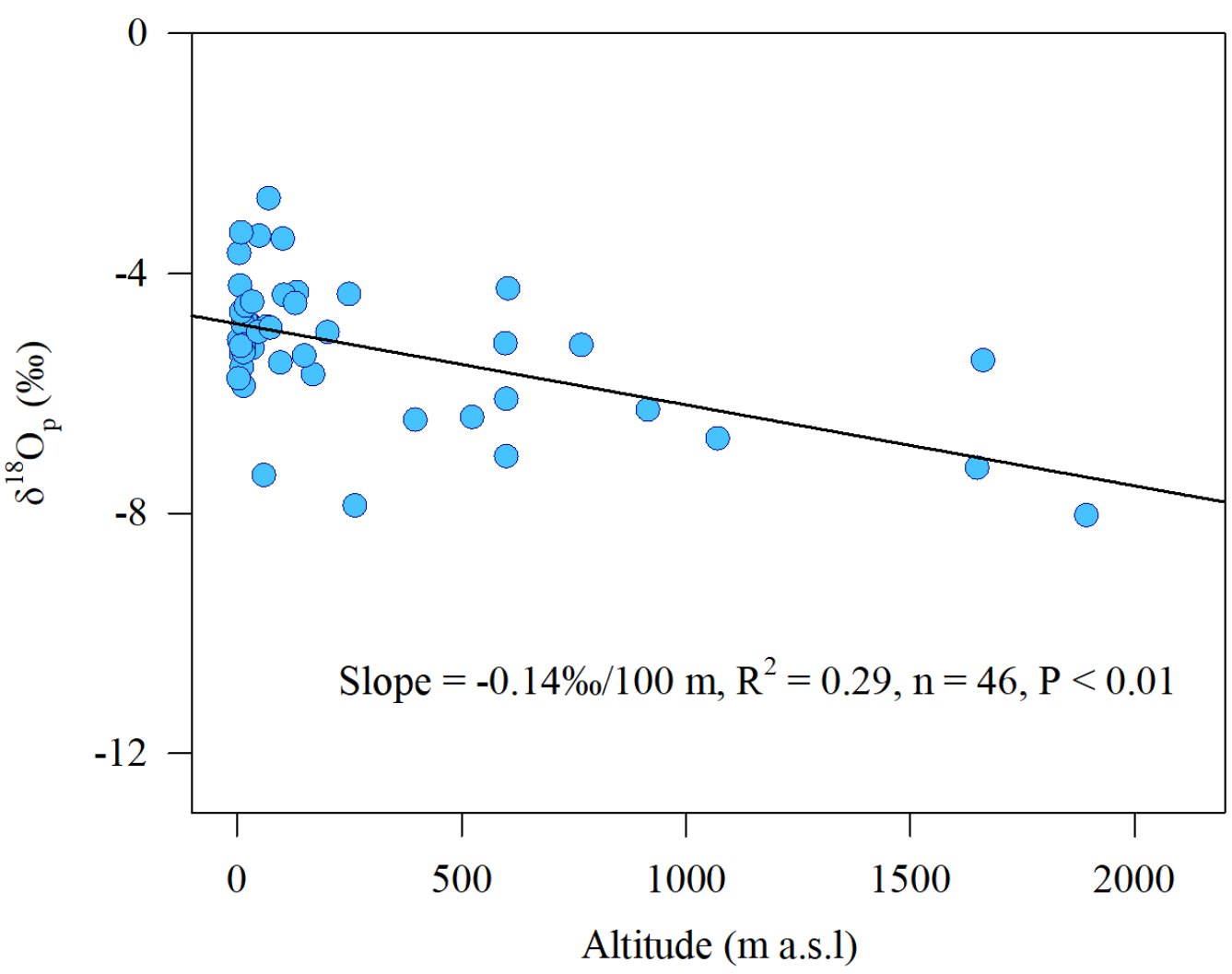

Figure R24. Influence of altitude on observed $\delta^{18}O_p$ at the 46 sites across the study region from 1979 to 2022. $\delta^{18}O_p$ values are expressed as per mil (‰) deviations relative to VSMOW or VSMOW2.

2) Next, we will examine the influence of altitude on $\delta^{18}O_p$ from two major perspectives (spatial perspective and temporal perspective), in order to eliminate

altitude:

a) Spatial perspective - horizontal direction (climate zone):

According to the Köppen-Geiger climate classification (Beck et al., 2023), our study region was divided into five climate zones: Tropical monsoon (Am), Tropical savannah (Aw), Temperate with dry winter and hot summer (Cwa), Temperate with dry winter and warm summer (Cwb), and Temperate with no dry season and hot summer (Cfa). We calculated correlations between $\delta^{18}O_p$ and altitude within each climate zone (Figure R25). The results showed that only one climate zone had a significant negative correlation between $\delta^{18}O_p$ and altitude (Figure R25d), indicating that altitude has limited influence on $\delta^{18}O_p$ variations along this transect.

Conversely, if we use these results to prove the altitude effect ($\delta^{18}O_p$ values show a negative correlation with altitude). Then, readers will doubt our conclusion, because two climate zones show positive correlations between $\delta^{18}O_p$ and altitude, rather than negative correlations, directly conflicting with the "altitude effect" (Figures R25b, c). Furthermore, while Figures R25e and R25f exhibit weak negative correlations, it has not passed the significance test and is far from the ideal "altitude effect" (Figures R25e, f). Therefore, this result can indicate that the influence of altitude on $\delta^{18}O_p$ values along this transect is very limited. Following your suggestion, we added a discussion on the relationship between $\delta^{18}O_p$ and altitude based on the Köppen-Geiger climate classification in the revised manuscript, and added a figure (Figure R25) in the revised Supplementary Information to discuss the relationship between observed $\delta^{18}O_p$ values in five climate zones and the corresponding altitudes. Please see L309–L311 in the revised manuscript and Fig. S13 in the revised Supplementary Information.

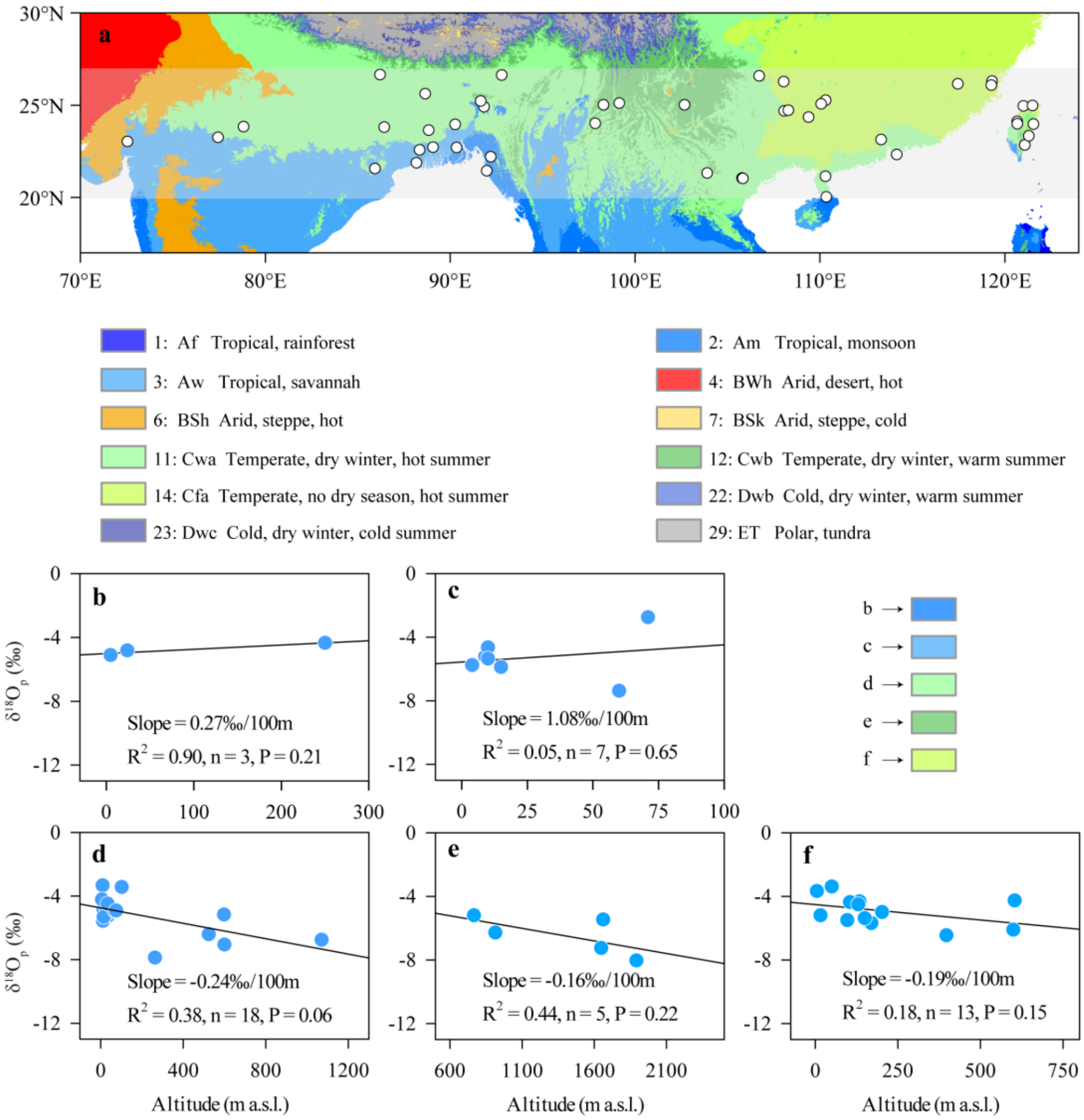

Figure R25. Correlations between observed $\delta^{18}O_p$ values and altitudes across five climate zones. a, Climate zones according to the Köppen-Geiger climate classification[1]. Correlations for the climate zones: Tropical, monsoon (Am) (b); Tropical, savannah (Aw) (c); Temperate, dry winter, hot summer (Cwa) (d); Temperate, dry winter, warm summer (Cwb) (e); and Temperate, no dry season, hot summer (Cfa) (f). $\delta^{18}O_p$ values are expressed as per mil (‰) deviations relative to VSMOW or VSMOW2.

b) Spatial perspective - vertical direction (altitude range):

It should be noted that more previous studies analyzed the relationship between $\delta^{18}O_p$ and altitude based on altitude classification. This is because by calculating the correlation between $\delta^{18}O_p$ and altitude in different altitude ranges, it is possible to reveal the different responses of $\delta^{18}O_p$ to changes in altitude, identifying which

altitude ranges exhibit negative correlations and where anomalous positive correlations occur. For example, Poage and Chamberlain (2001) compiled water isotopes data from 68 studies across global mountain regions. They concluded that the water isotopes decrease linearly with increasing altitude in most regions of the world except in the Himalayas and at altitudes >5000 m (Poage and Chamberlain, 2001). This finding indicates that research on water isotopes and altitude relationships (particularly regarding "inverse altitude effects") primarily focuses on how these relationships vary across specific altitude ranges, especially where abnormally positive correlations appear.

We also provided additional examples, such as: Case 1 (Botsyun & Ehlers, 2021):

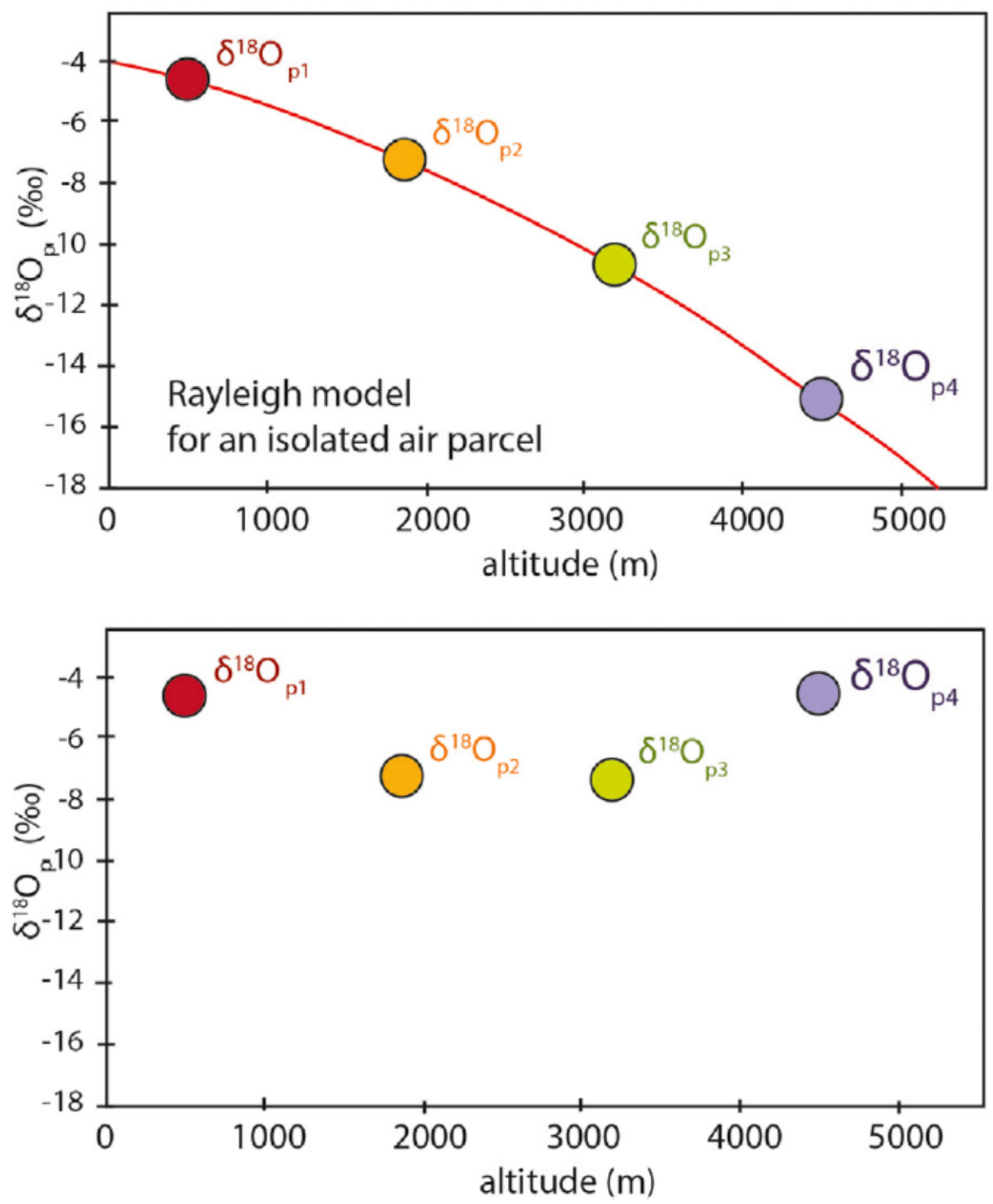

Figure R26. (A) an isotopic lapse rate according to a Rayleigh distillation model for an isolated air parcel; and (B) flattened or inversed isotopic lapse rate. Examples of proxy data interpretations based on iGCM results.

Case 2 (Pant et al., 2021):

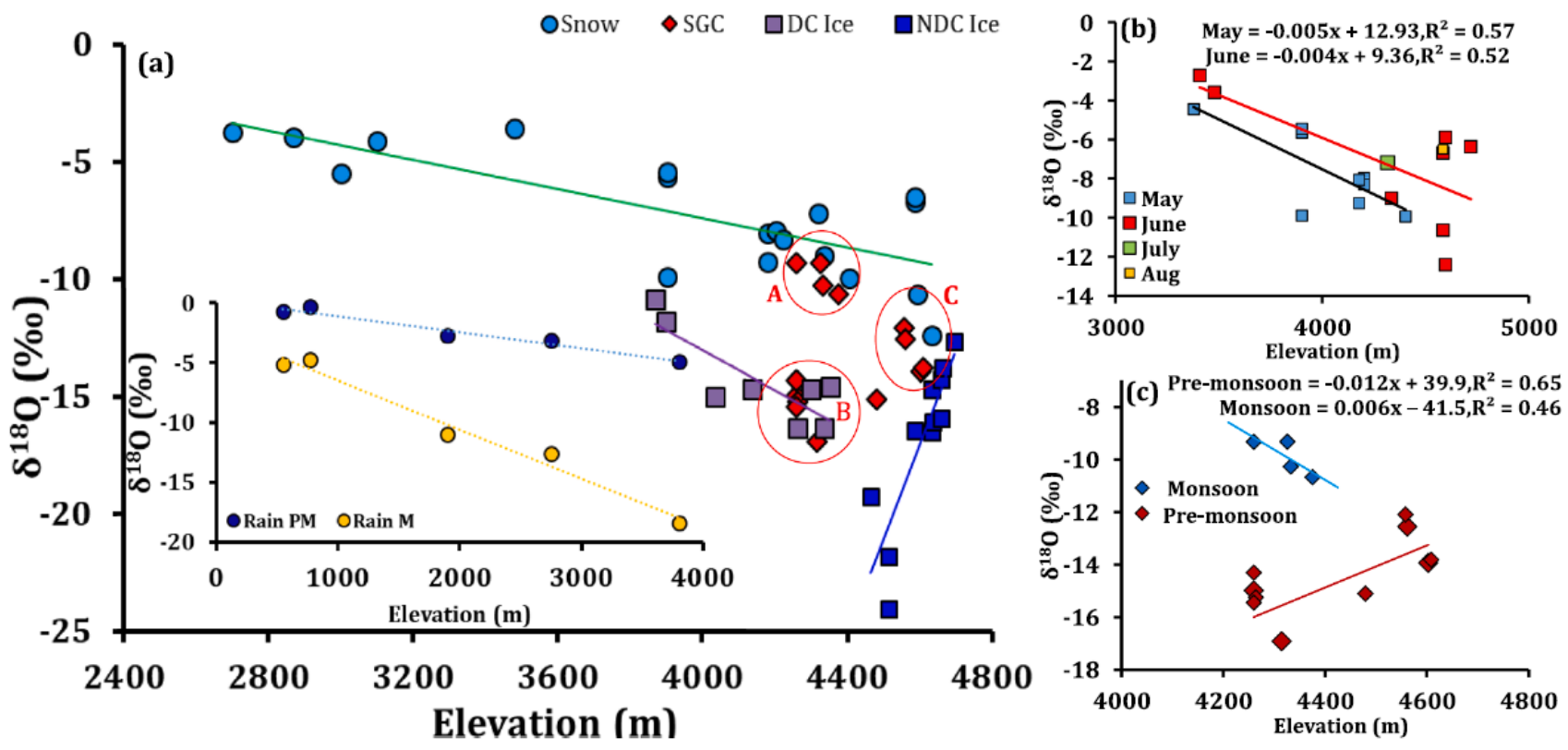

Fig. 8. (a) Variation of $\delta^{18}O$ with altitude in SGC, snow and glacial ice (debris covered and non-debris covered). Inset showing altitude effect in rainfall during pre-monsoon (PM) and monsoon (M) for the Upper Alaknanda River basin. Variation of $\delta^{18}O$ with the altitude during different ablation periods in (b) snow and (c) SGC water.

Figure R27. (a) Variations of $\delta^{18}O$ in water samples from supraglacial channels (SGC), snow and glacial ice (debris covered and non-debris covered) with altitude. Inset showing altitude effect in rainfall during pre-monsoon (PM) and monsoon (M)for the Upper Alaknanda River basin. Variation of $\delta^{18}O$ with the altitude during different ablation periods in (b) snow and (c) SGC water.

Case 3（Bai et al., 2014）：

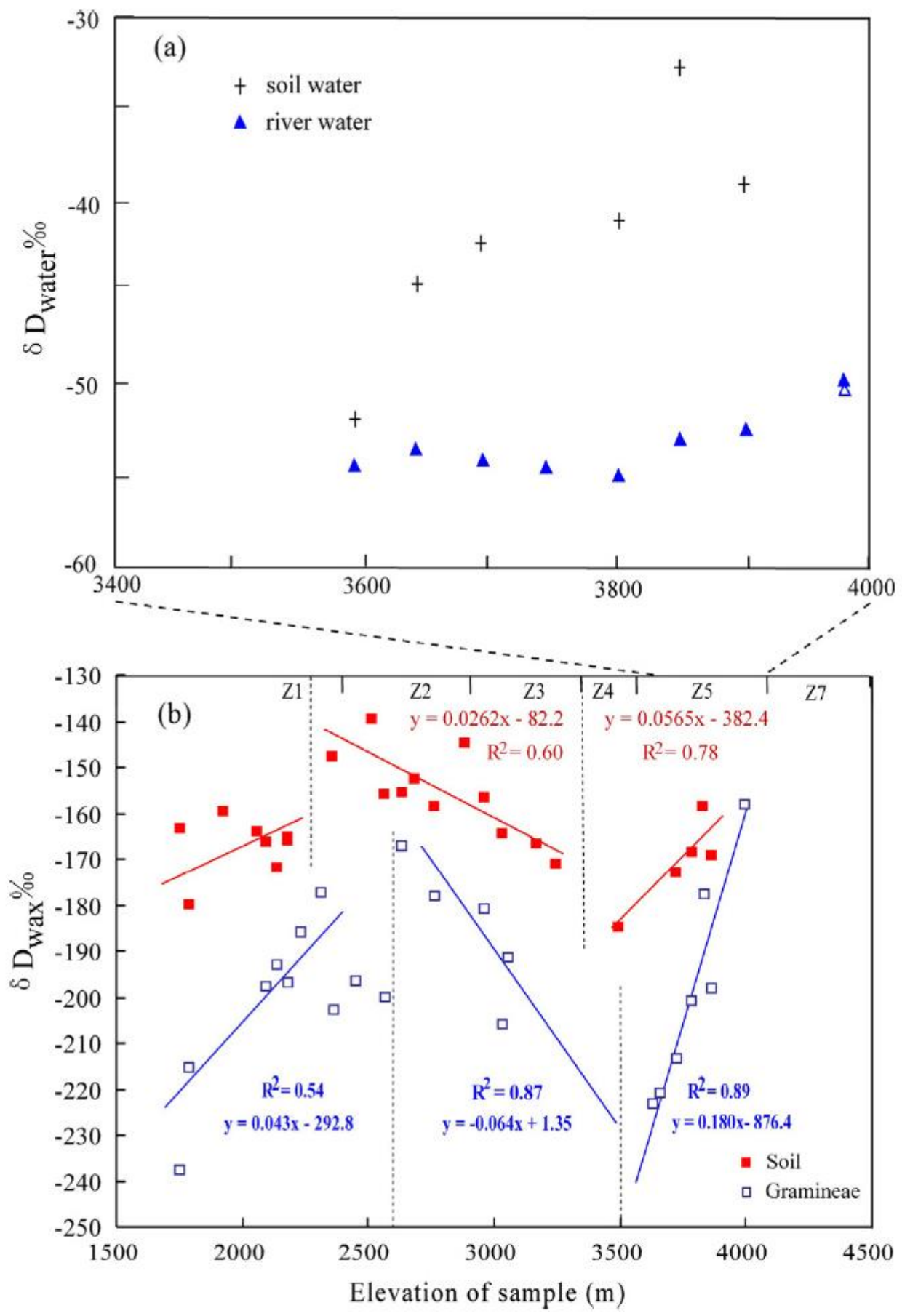

Figure R28. Comparison of Gramineae and soil δD_{wax} values along the Xiying River

watershed with elevation variation. Soil and river water isotope data (δD_{sw} and δD_{rw}) above 3500 mm from the Lenglong Range are also showed on the plot, to analyze the main control factors of the "inverse altitude effect" in the high mountain periglacial regions.

Therefore, in this study, in addition to adding the discussion of the relationship between observed $\delta^{18}O_p$ values and the corresponding altitudes based on the Köppen-Geiger climate classification, we also retained the discussion of the relationship between observed $\delta^{18}O_p$ values and the corresponding altitudes based on the UNEP-WCMC mountain classification. The result show that across these three altitude ranges, no significant negative correlation between $\delta^{18}O_p$ and altitude, indicating that the $\delta^{18}O_p$ are not influenced by the "altitude effect." We added relevant discussion and updated the corresponding figure (Figure R29). Please see L309–L311 in the revised manuscript and the updated Fig. S14 in the revised Supplementary Information.

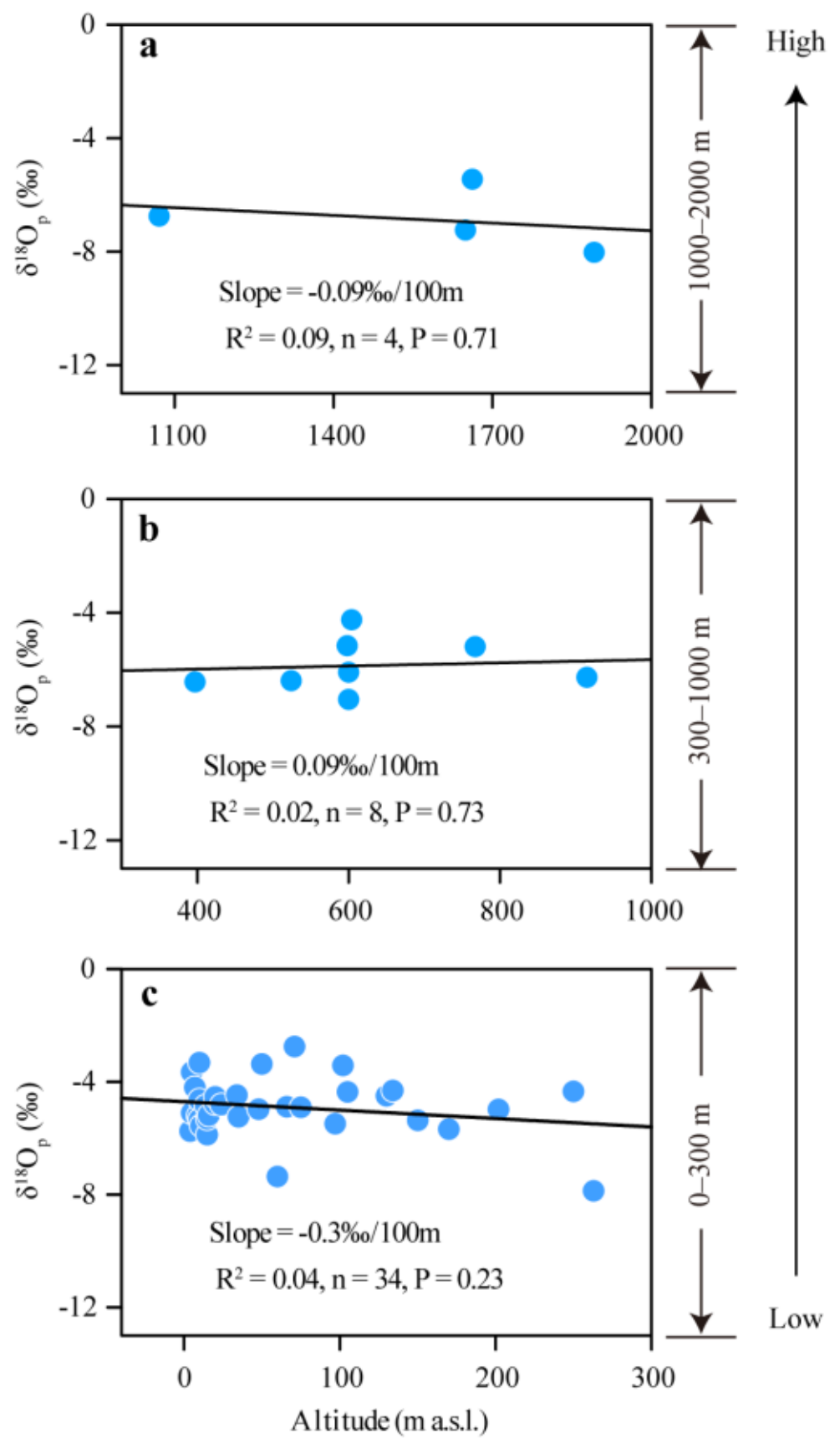

Figure R29. Correlations between observed $\delta^{18}O_p$ values and altitudes. The 46 precipitation sites were categorized into three altitude ranges based on the

UNEP-WCMC mountain classification[2]. a–c, Correlations for the altitude ranges 1000–2000 m (a), 300–1000 m (b), and 0–300 m (c). $\delta^{18}O_p$ values are expressed as per mil (‰) deviations relative to VSMOW or VSMOW2.

2) Temporal perspective

To test for an "altitude effect" where $\delta^{18}O_p$ decrease with increasing altitude due to lower condensation temperature[69], we analyzed $\delta^{18}O_p$ versus altitude monthly (Fig. 3c). Correlations change over time from weak positive, weak negative, and then to strong negative (Fig. 3c), reflecting the westward migration of low $\delta^{18}O_p$ from pre-monsoon to monsoon, aligning with high-elevation areas and producing a "false" altitude effect (Supplementary Fig. 12). In conclusion, the altitude has little influence on the spatial pattern of $\delta^{18}O_p$ along this transect.

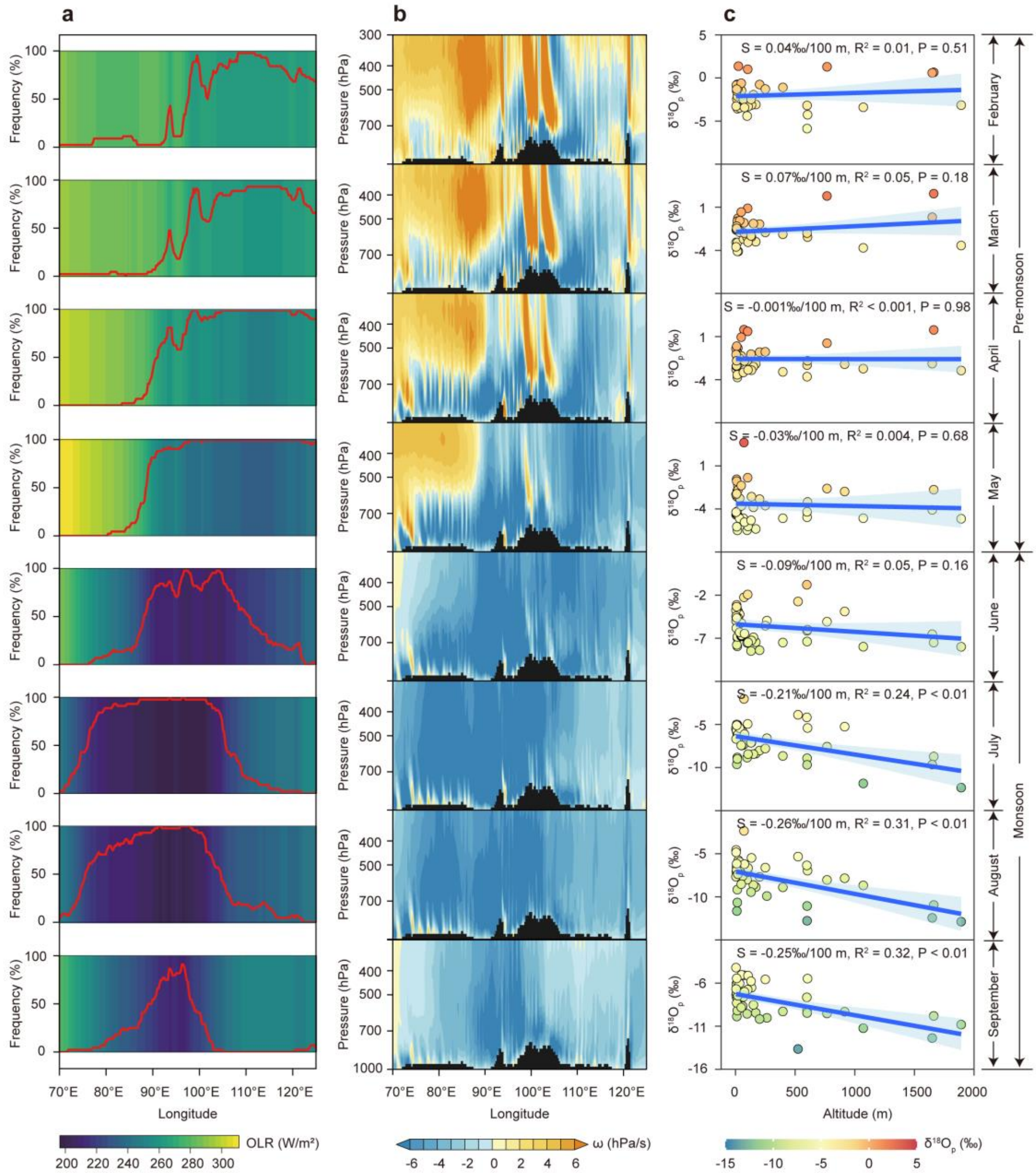

Figure R30. Seasonal changes of atmospheric updraft and altitude effect on $\delta^{18}O_p$ during the pre-monsoon and monsoon seasons over 1979–2022. a Seasonal variations in outgoing longwave radiation (OLR, shading) and the frequencies of relatively low OLR values (red line) along the transect. Lower OLR values indicate stronger convection. b Vertical profiles of meridional mean vertical velocity (ω), averaged over 20–27° N. Negative ω values indicate upward motion (convection), while positive values indicate downward motion (subsidence). c Linear regressions of $\delta^{18}O_p$ at the 46 sites across the study region against altitude, fitted for each month. $\delta^{18}O_p$ values are expressed as per mil (‰) deviations relative to VSMOW or VSMOW2.

Therefore, we added two paragraphs to demonstrate that the altitude does not

significantly control $\delta^{18}O_p$ distribution in the study region. i.e., *"One might argue that terrain could influence $\delta^{18}O_p$, given numerous high mountains in the middle of the transect. To test for an "altitude effect" where $\delta^{18}O_p$ decrease with increasing altitude due to lower condensation temperature[69], we analyzed $\delta^{18}O_p$ versus altitude monthly (Fig. 3c). Correlations change over time from weak positive, weak negative, and then to strong negative (Fig. 3c), reflecting the westward migration of low $\delta^{18}O_p$ from pre-monsoon to monsoon, aligning with high-elevation areas and producing a "false" altitude effect (Supplementary Fig. 12). Classification of sites by Köppen-Geiger climate zone[70] (Supplementary Fig. 13) or UNEP-WCMC altitude ranges[71] (Supplementary Fig. 14) shows inconsistent or absent negative $\delta^{18}O_p$–altitude correlations. Previous studies also reported "inverse altitude effects" in this region[72-74]. For example, $\delta^{18}O_p$ on the Ainao Mountains (23.22° N, 102.84° E) increases with altitude[72,73], due to complex moisture sources, and $\delta^{18}O_p$ on the leeward side of the Meili Snow Mountains (28.46° N, 98.77° E) shows similar pattern[74], due to below-cloud evaporation and recycled moisture mixing. These results indicate altitude does not significantly control $\delta^{18}O_p$ distribution. More important, the gradually changing correlation between $\delta^{18}O_p$ and altitude further support our conclusion that the low $\delta^{18}O_p$ values gradually migrate westward along the transect over time."* Please see L302–L319 and Fig. 3c in the revised manuscript.

References:

69. Dansgaard, W. Stable isotopes in precipitation. *Tellus* **16**, 436–468 (1964).

70. Beck, H. E. et al. High-resolution (1 km) Köppen-Geiger maps for 1901–2099 based on constrained CMIP6 projections. *Sci. Data* **10**, 724 (2023).

71. UNEP World Conservation Monitoring Centre. *Mountain Watch: Environmental Change and Sustainable Development in Mountains*. Technical Report. Cambridge, UK: UNEP-WCMC, 2002.

72. Jiao, Y. et al. Impacts of moisture sources on the isotopic inverse altitude effect and amount of precipitation in the Hani Rice Terraces region of the Ailao Mountains. *Sci. Total Environ.* **687**, 470–478 (2019).

73. Jiao, Y., Liu, C., Liu, Z., Ding, Y. & Xu, Q. Impacts of moisture sources on the temporal and spatial heterogeneity of monsoon precipitation isotopic altitude effects. *J. Hydrol.* **583**, 124576 (2020).

74. Kong, Y. et al. Ambiguity in the altitude effect of precipitation isotopes for estimating groundwater recharge elevation and paleoelevation reconstruction in the leeward side of a mountain. *Hydrogeol. J.* **31**, 1259–1270 (2023).

Bai, Y., Tian, Q., Fang, X. & Wu, F. The "inverse altitude effect" of leaf wax-derived n-alkane δD. *Org. Geochem.* **73**, 90–100 (2014).

Botsyun, S. & Ehlers, T. A. How Can Climate Models Be Used in Paleoelevation Reconstructions? *Front. Earth Sci.* **9**, 624542 (2021).

Pant, N. et al. Tracing the isotopic signatures of cryospheric water and establishing the altitude effect in Central Himalayas: A tool for cryospheric water partitioning. *J. Hydrol.* **595**, 125983 (2021).

Poage, M. A. & Chamberlain, C. P. Empirical relationships between elevation and the stable isotope composition of precipitation and surface waters: considerations for studies of paleoelevation change. *Am. J. Sci.* **301**, 1-15 (2001).

Additionally, from the way this is written (136-144), it is unclear if there is an altitude effect in the region or not. The authors state they find a significant altitude effect in the 250-800m bracket (although the p-value is > 0.05 and therefore not significant), then that there is an "inverse altitude effect" in the region, and then conclude that there is no significant effect on altitude.

Re: We thank you for pointing this issue out. The "altitude effect" requires water isotope values should be negatively correlated with altitude. If the correlation between water isotope values and altitude is abnormally positive, then the result will be conflicted with the "altitude effect." That is to say, the abnormally positive correlation (or no significant correlation) can demonstrate that altitude has no effect on the water isotope values. Note that some studies defined the abnormally positive correlation as "inverse altitude effect." Hence, once $\delta^{18}O_p$ values exhibit an "inverse altitude effect", we can say that $\delta^{18}O_p$ values did not affected by "altitude effect."

We checked the statement in Line 136-138 ("The data indicate an abnormally positive correlation between $\delta^{18}O_p$ values and altitude within the 250–800 m range, whereas no significant correlation exists for sites at 0–250 m or 800–2000 m"). This means that within the 250–800 m range, there is an "inverse altitude effect", while no significant correlation is observed at 0–250 m or 800–2000 m. Therefore, based on

this statement, we can drive the conclusion that “there is no significant effect on altitude,” based on the statement as mentioned above.

Following your suggestion, we rewrote the section on the relationship between altitude and $\delta^{18}O_p$ from a spatial perspective (horizontal and vertical directions) and a temporal perspective to eliminate the influence of altitude on $\delta^{18}O_p$. Please see L302–L319 and Fig. 3c in the revised manuscript.

Pre-monsoon “low in the East, high in the West”

The authors say they are using 12 sites, but only 9 are included here, with the westernmost seemingly having no data in the pre-monsoon season. Is there simply not enough data from these sites with missing values – are they too dry (this is why I think plotting monthly rainfall would be helpful for the readers)? For the remaining data, the authors may also consider having a quantification statistic for this claim. A Tukey test across all boxes, or a t.test (between sites lumped into East and West) would be helpful. In any case, they may need to provide a more cautious interpretation of the $\delta^{18}O$ variation, since there is quite significant overlap between the boxes, which calls into question if the “westward migration” is actually significant.

Re: We thank you for pointing these issues out. Following your comments, we addressed these issues as follows:

1) Indeed, the westernmost sites do not cover the whole year. Yes, this is because it is too dry. It should be noted that in Fig. 2a-d of our manuscript, we focused on the pre-monsoon season. During this period, we observed a westward migration of low $\delta^{18}O_p$ values from the easternmost site of the East Asian monsoon region toward the dividing line (~98° E) (i.e., the middle of the transect). Based on subsequent analyses, we find that the combined influence of the ITCZ, IPWP, and WPSH drives this gradual westward migrate of low $\delta^{18}O_p$ values from February to May. However, this influence does not extend to the westernmost site of the South Asian monsoon region (i.e., the westernmost site of the transect). Therefore, the absence of $\delta^{18}O_p$ data from sites in the western domain of the South Asian monsoon region during February–May does not affect our conclusions. Following your suggestion, we added monthly precipitation amount in Supplementary Fig. S4. Please see the updated Fig. S4 in the revised Supplementary Information.

Following the comments provided by you and both reviewers #1 and #2, for the pre-monsoon season, we changed the statement "low in the east, high in the west" to "*$\delta^{18}O_p$ values follow a pattern of lower values in the east and higher values in the west of the ~98° E dividing line*." Please see L147–L148 in the revised manuscript. In addition, we changed the statement "migrates westward" to "migrates toward the dividing line." Please see L149 in the revised manuscript.

2) We believe that only when the $\delta^{18}O_p$ values undergo a sudden and drastic change can they be detected as having a significant difference by the Tukey test. However, our study found that low $\delta^{18}O_p$ values gradually migrated westward, making it difficult to observe a significant difference. This is because the ITCZ, IPWP, and WPSH all change gradually over time, rather than abruptly jumping. Fortunately, we can use another perspective to confirm that the low $\delta^{18}O_p$ values do indeed migrate westward. That is, since terrain is fixed, the most effective way to determine whether $\delta^{18}O_p$ is influenced by topography is to analyze its relationship with altitude on a monthly basis (Figure R31c). We observed a gradual shift in these correlations over time: they transitioned from a weak positive correlation to a weak negative correlation and eventually to a remarkable negative correlation (Figure R31c). This shift arises because low $\delta^{18}O_p$ values gradually migrate toward the middle of the transect from the pre-monsoon to the monsoon season. As a result, the low $\delta^{18}O_p$ values gradually align with the high-elevation areas in the middle of the transect, creating a "false" altitude effect (Supplementary Fig. 12). In conclusion, the altitude has little influence on the spatial pattern of $\delta^{18}O_p$ along this transect. In summary, the observed temporal shift in the correlation between $\delta^{18}O_p$ and altitude results from the gradual westward migration of stronger atmospheric updrafts, which in turn causes the westward movement of low $\delta^{18}O_p$ values. This demonstrates that altitude has a negligible influence on the spatial distribution of $\delta^{18}O_p$ along this transect. Therefore, we added a paragraph highlighting how the $\delta^{18}O_p$-altitude relationship evolves over time, and this is used to confirm that the low $\delta^{18}O_p$ values do indeed migrate westward. i.e., "*To test for an "altitude effect" where $\delta^{18}O_p$ decrease with increasing altitude due to lower condensation temperature*[69]*, we analyzed $\delta^{18}O_p$ versus altitude monthly (Fig. 3c). Correlations change over time from weak positive, weak negative, and then to strong negative (Fig. 3c), reflecting the westward migration of low $\delta^{18}O_p$ from*

pre-monsoon to monsoon, aligning with high-elevation areas and producing a "false" altitude effect (Supplementary Fig. 12)." Please see L303–L308 in the revised manuscript.

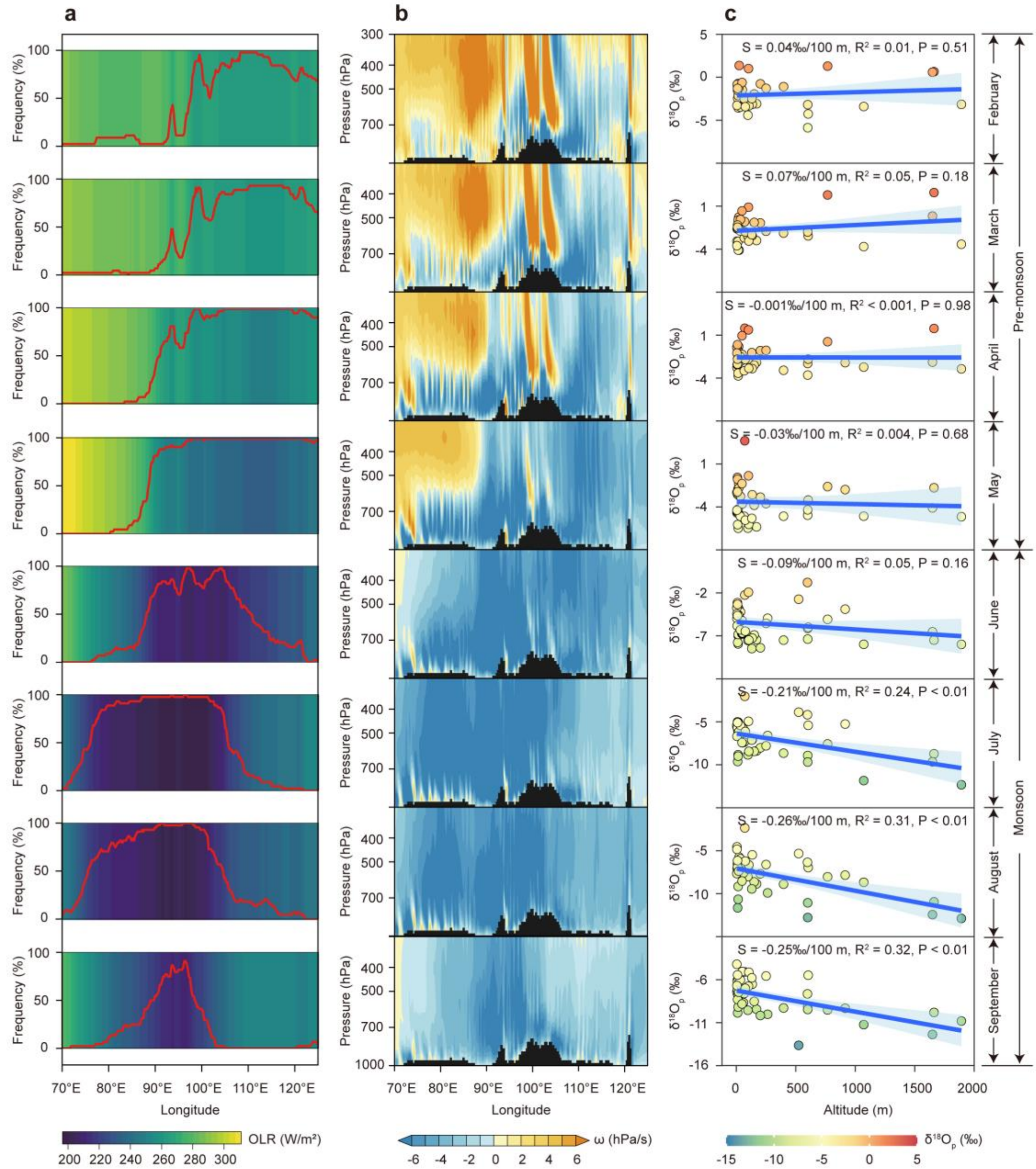

Figure R31. Seasonal changes of atmospheric updraft and altitude effect on $\delta^{18}O_p$ during the pre-monsoon and monsoon seasons over 1979–2022. a Seasonal variations in outgoing longwave radiation (OLR, shading) and the frequencies of relatively low OLR values (red line) along the transect. Lower OLR values indicate stronger convection. b Vertical profiles of meridional mean vertical velocity (ω), averaged over 20–27° N. Negative ω values indicate upward motion (convection), while positive values indicate downward motion (subsidence). c Linear regressions of $\delta^{18}O_p$ at the 46 sites across the study region against altitude, fitted for each month. $\delta^{18}O_p$

values are expressed as per mil (‰) deviations relative to VSMOW or VSMOW2.

Monsoon V-shape

I think the authors may need to rule out that the precipitation amount is not controlling $\delta^{18}O$ at these sites. Is this V-shape present in the monthly precipitation amount? If these sites simply receive more rainfall, then can rainfall amount explain the speleothem record $\delta^{18}O$? Or if this updraft is actually correlated to rainfall amount (which seems possible), then how do we distinguish their effects? (Perhaps they are distinguishable, and it simply needs to be explained in plainer language).

Re: We thank you for pointing this issue out. We addressed these issues as follows:

1) With regard to the relationship between $\delta^{18}O_p$ and precipitation amount, we analyzed the spatial pattern of precipitation amount along the transect during the monsoon season. As we propose that $\delta^{18}O_p$ values along the transect show a "V-shaped" pattern, that is, higher $\delta^{18}O_p$ values at the eastern and western margins and lower values in the middle. If precipitation amount affects the V-shaped pattern of $\delta^{18}O_p$ along the east–west transect, precipitation amount along the transect should appear as an "inverted V-shaped" pattern during the monsoon season. However, our precipitation amount data do not show an "inverted V-shaped" pattern (Figure R32), which can not explain the V-shaped pattern of $\delta^{18}O_p$. More important, a notable discrepancy was observed at the Sylhet site, where monthly precipitation amount is exceptionally high, yet the $\delta^{18}O_p$ at this site did not exhibit the correspondingly expected low values (Figure R32). Instead, the $\delta^{18}O_p$ at this site remained relatively high. This result conflicted with the theory of precipitation "amount effect." Therefore, it can be seen that the spatial pattern of precipitation amount is inconsistent with that of the $\delta^{18}O_p$ along the transect. Thus, we can conclude that the $\delta^{18}O_p$ along the transect shows little correlation with the precipitation amount. We added a figure to show the spatial pattern of precipitation amount along the east–west transect (Please see Figure R32 or Fig. S15 in the revised Supplementary Information), and we added a paragraph in the manuscript to eliminate the influence of precipitation amount on $\delta^{18}O_p$ along the transect.

2) With regard to the relationship between atmospheric updraft and precipitation amount, following your suggestion, we analyzed spatial distribution of OLR and

precipitation amount. The result showed that the patterns of the OLR and precipitation amount were inconsistent (Figure R33). This indicates that atmospheric updraft actually has little contribution to precipitation amount. We added a paragraph to explain that the atmospheric updraft has little contribution to precipitation amount, i.e., "*It could also be proposed that terrain-enhanced updrafts might increase precipitation, linking* $\delta^{18}O_p$ *to precipitation amount. If true, monsoon-season precipitation should show an inverted V-shaped pattern along the transect. However, our results do not support this hypothesis as precipitation amount in the middle is relatively low while it is considerably higher at Sylhet in the western domain (Supplementary Fig. 15).*" Please see L320–L325 in the revised manuscript.

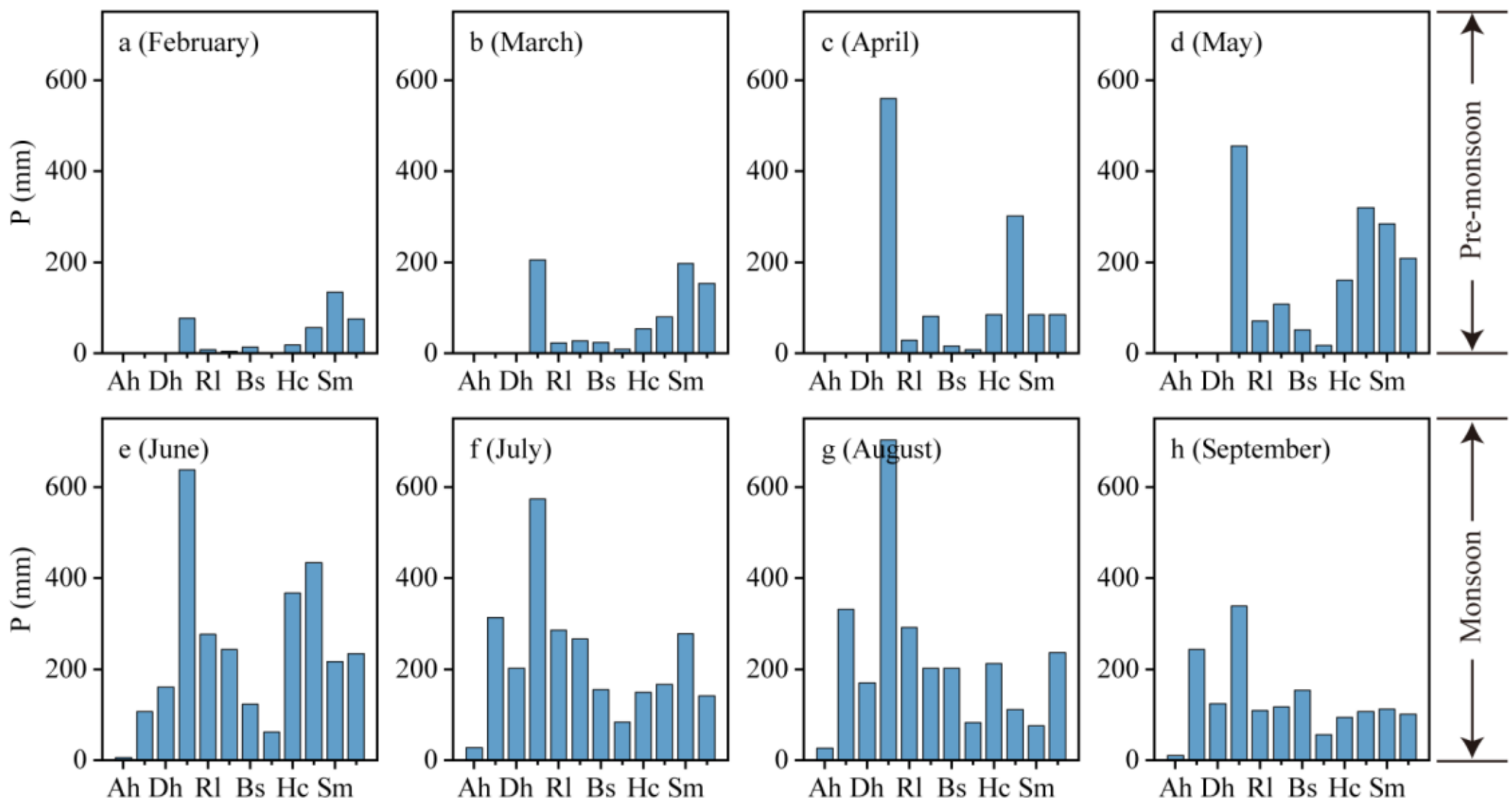

Figure R32. Spatial distributions of precipitation amount at the 12 primary sites along the transect during the pre-monsoon and monsoon seasons. a–d, Pre-monsoon season. e–h, Monsoon season. Abbreviations: Ah (Ahmedabad), Dh (Dhanbad), Rl (Ruili), Bs (Baoshan), Hc (Hechi), and Sm (Sanming).

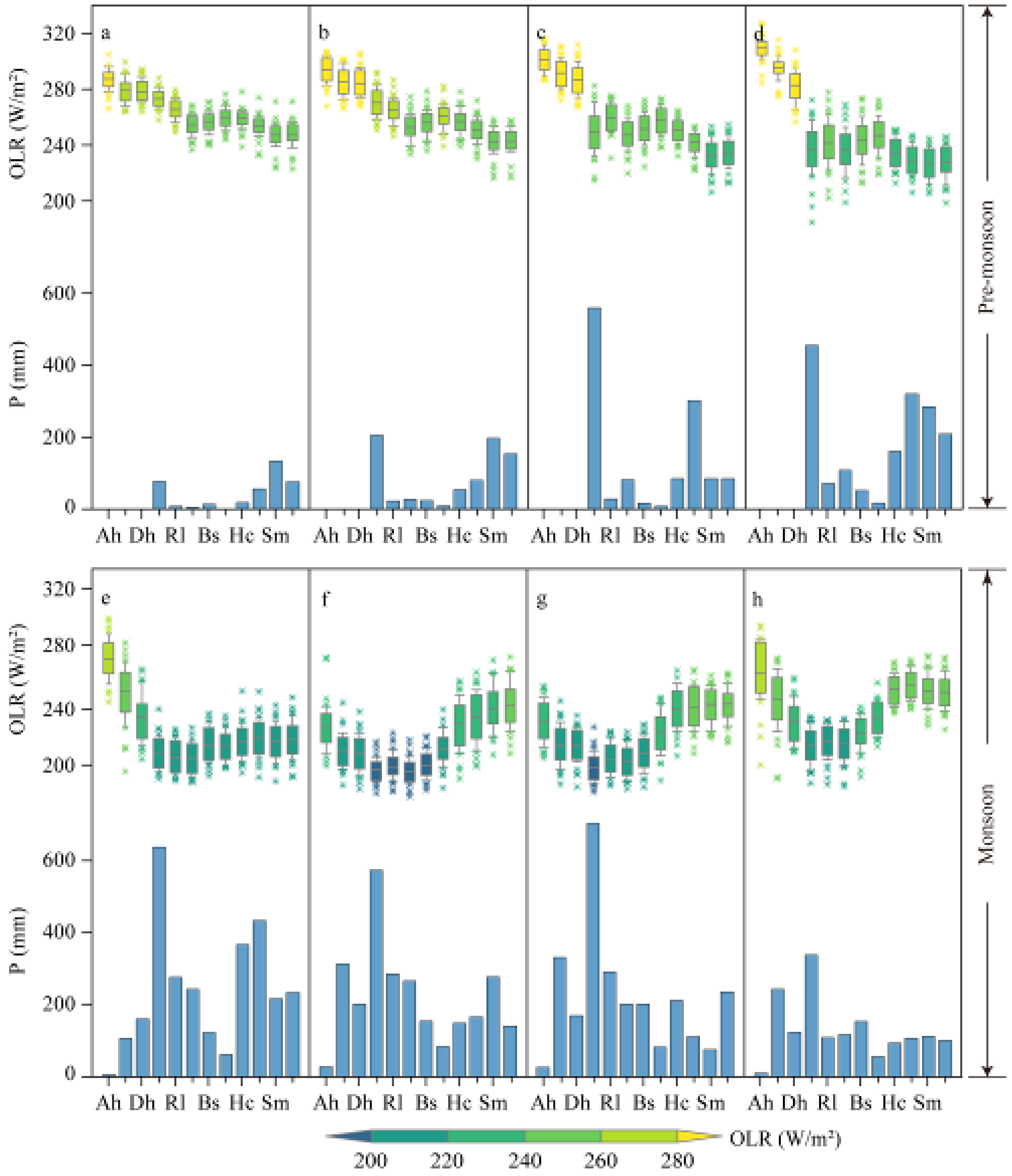

Figure R33. Spatial distributions of outgoing longwave radiation (OLR) and the precipitation amount at the 12 primary sites along the east–west transect over 1979–2022. (a–d) The pre-monsoon season. (e–h) The monsoon season. i–l The post-monsoon season. In each box plot, the box represents the 20th–80th percentile range, whiskers extend to the 10th–90th percentile, horizontal lines inside the boxes indicate the mean, and dots represent outliers.

$\delta^{18}O_P$ and the advection of monsoon moisture

Are the predictor variables correlated? In cases (Tab. S3) where there are two significant predictor variables, the correlations are heading in the same direction (Tab S3). So it is likely they are correlated, and distinguishing their effects is more

challenging. The authors may benefit from producing a correlation matrix of the predictor variables at each site.

Re: We thank you for pointing these issues out. We addressed these issues as follows: Following your comments, we carefully rechecked the original Table S3 (now Tables S4 and S5 in the revised Supplementary Information) and found that there are indeed two significant predictor variables, the correlations are heading in the same direction. However, it should be noted that both SASMI (South Asia Summer Monsoon Index) and IMI (Indian Monsoon Index) represent the intensity of the South Asian monsoon, although these two indices were obtained from different studies with slightly different definition. Therefore, it is reasonable that the correlation direction of $\delta^{18}O_p$ with SASMI and IMI is consistent.

For the two indices of EASMI (East Asia Summer Monsoon Index) and WNPMI (Western North Pacific Monsoon Index), although there are clear distinctions in the characteristics of rainy season and low-level wind variation among east Asia, and the Western North Pacific, the two components of East Asia Summer Monsoon and Western North Pacific Monsoon are no doubt interlinked (Wang and Ho, 2002). This perspective agrees with Tao and Chen (1987), who viewed the East Asian and Western North Pacific monsoons as constituting an integrated system. Thus, it is reasonable that the consistent correlation direction of $\delta^{18}O_p$ with EASMI and WNPMI may also exist.

It should also be noted that if the changes in $\delta^{18}O_p$ are influenced by these monsoon systems, then it is required that the $\delta^{18}O_p$ have a significant negative correlation with these monsoon indices. In this study, we found that at the monthly and annual scales, although the $\delta^{18}O_p$ showed a negative correlation with SASMI and IMI, their correlation was far less significant than that between $\delta^{18}O_p$ and OLR. Especially at the annual scale, the negative correlations between $\delta^{18}O_p$ and SASMI and IMI were not significant. This fully demonstrates that $\delta^{18}O_p$ are limitedly influenced by the South Asian monsoon intensity, but mainly affected by atmospheric updraft. This is why the advection of monsoon moisture in South Asia cannot explain the V-shaped spatial pattern of $\delta^{18}O_p$ along the transect.

For the two indices of EASMI and WNPMI, on a monthly scale, we can find that although there is a negative correlation between $\delta^{18}O_p$ and WNPMI at some sites,

these negative correlations are not as significant as the correlation between $\delta^{18}O_p$ and OLR. In particular, there is an abnormal positive correlation between $\delta^{18}O_p$ and EASMI, indicating that the East Asian monsoon intensity has little contribution to $\delta^{18}O_p$. On an annual scale, we can find that the $\delta^{18}O_p$ at almost all sites show a significant positive correlation with EASMI and WNPMI. This further demonstrates that the changes in $\delta^{18}O_p$ are independent of the influence of the East Asian Monsoon and the Western North Pacific Monsoon.

References:

Wang, B., and Ho, L. Rainy season of the Asian–Pacific summer monsoon. *J. Climate*, **15**, 386–398 (2002).

Tao, S. & Chen, L. A review of recent research on the East Asian summer monsoon in China. Monsoon Meteorology (eds. Chang, C. P. & Krishnamurti, T. N.) 60–92 (Oxford University Press, 1987).

$\delta^{18}O_p$ and atmospheric updraft

The $\delta^{18}O$ and OLR gradient correlation requires further consideration because it includes different datasets at different points, thus, the gradient is influenced by which sites are included, rather than the monthly variations between sites alone. I think the authors need to decide on a set of sites to use in their analysis. If they don't have sufficient data from across all sites, then the data isn't sufficient for their claims.

Re: We thank you for pointing this issue out. We admit that our analysis of the $\delta^{18}O$ and OLR gradient correlation includes different datasets from different sites, but we justify this approach for the following reasons:

Figures 4c and 4d were designed to further validate the gradually westward migration of low $\delta^{18}O_p$ values from the easternmost site of the East Asian monsoon region toward the dividing line (~98°E; i.e., the middle of the transect) during the pre-monsoon season. Therefore, we conducted a gradient analysis from the easternmost site towards the dividing line (~98° E) (that is, from Luoyuan to Kunming transect) from February to May.

In contrast, Figures 4e and 4f were designed to further validate that the pre-monsoon season pattern shifted to the V-shaped pattern during the monsoon season, which

requires focusing on the monsoon season. Our study found that the lower values of $\delta^{18}O_p$ and OLR are appeared around the middle of the transect, resulting in a V-shaped pattern along the transect during the monsoon season. Consequently, $\delta^{18}O_p$ and OLR values increase from the middle to the easternmost site (Luoyuan). This implies that the gradients (slopes) of $\delta^{18}O_p$ and OLR from the middle to Luoyuan should be positive values. That is, if the slopes of $\delta^{18}O_p$ and OLR from a specific site in the middle to Luoyuan are negative values, it suggests that the lower $\delta^{18}O_p$ and OLR values do not extend westward to the specific site. In this case, we should focus on the "critical site" which is most adjacent to the east of the specific site. Clearly, the "critical site" was characterized by lower $\delta^{18}O_p$ and OLR values. Our analysis found that in June the lower $\delta^{18}O_p$ and OLR values appear around Baoshan ("critical site") in the middle of the transect. Then the "critical site" subsequently migrates westward to Tengchong, Ruili, and Sylhet from July to September, respectively. Therefore, gradient analysis can only be conducted from Baoshan in June, Tengchong in July, Ruili in August, and Sylhet in September to the easternmost site Luoyuan, respectively.

In summary, the difference in data used across different sections is because that the study purposes are different.

296-307: is this what the authors are proposing as a conclusion? Or do they have observation/modelling/experimental/literature support? If so, the evidences need to be properly cited, or the authors should make the reader aware that they are proposing something.

Re: We thank you for pointing this issue out. This statement is mainly supported by the literature (Cole et al., 1999; Vuille et al., 2003; Kurita, 2013; Yu et al., 2021; Zhang et al., 2021). Following the comments provided by reviewers #1 and #2, we moved this paragraph to the beginning of the "$\delta^{18}O_p$ and atmospheric updraft" section. Following your comment, we also added these references in the revised manuscript. Please see L225–L236 in the revised manuscript.

References:

Cole, J. E., Rind, D., Webb, R. S., Jouzel, J. & Healy, R. Climatic controls on

interannual variability of precipitation $\delta^{18}O$: Simulated influence of temperature, precipitation amount, and vapor source region. *J. Geophys. Res.* **104**, 14223–14235 (1999).

Kurita, N. Water isotopic variability in response to mesoscale convective system over the tropical ocean. *J. Geophys. Res. Atmos.* **118**, 10376–10390 (2013).

Vuille, M., Bradley, R. S., Werner, M., Healy, R. & Keimig, F. Modeling $\delta^{18}O$ in precipitation over the tropical Americas: 1. Interannual variability and climatic controls. *J. Geophys. Res.* **108**, 4174 (2003).

Yu, W. et al. Temperature signals of ice core and speleothem isotopic records from Asian monsoon region as indicated by precipitation $\delta^{18}O$. *Earth Planet. Sci. Lett.* **554**, 116665 (2021).

Zhang, J. et al. Coupled effects of moisture transport pathway and convection on stable isotopes in precipitation across the East Asian monsoon region: Implications for paleoclimate reconstruction. *J. Climate* **34**, 9811–9822 (2021).

Implications for speleothem paleoclimatology

I think the authors may need to conduct a little more work to ensure the statistical robustness of their claim that $\delta^{18}O_p$ trend from the cave sites matches the $\delta^{18}O_s$ trend. Can the authors address this by 1) conducting a Tukey test to test for significant differences between the $\delta^{18}O_p$ means? 2) Provide an additional boxplot of monsoon vs. pre-monsoon season $\delta^{18}O_p$ at the cave sites? 3) provide a correlation/regression between $\delta^{18}O_s$ and $\delta^{18}O_p$

Re: We thank you for pointing this issue out. We addressed these issues as follows:

1) With regard to the significant differences in the multi-year mean $\delta^{18}O_p$ values shown in the original Figure S21, following your suggestion, we conducted a Tukey test on the data from the original Figure S21. The results showed that although there were significant differences in the multi-year mean $\delta^{18}O_p$ values at most sites, there were no significant differences in the multi-year mean $\delta^{18}O_p$ values at some sites (Table R12).

Table R12. Tukey test of multi-year mean $\delta^{18}O_p$ values across the 12 primary sites along the transect.

Group 1	Group 2	Mean Diff	p-value	Significant
Ah	Bs	-4.2108	0	Yes
Ah	Dh	-4.8704	0	Yes
Ah	Hc	-1.985	0	Yes
Ah	Ikg	-2.2877	0	Yes
Ah	Km	-6.006	0	Yes
Ah	Ly	-1.3776	0.0002	Yes
Ah	Rl	-3.2582	0	Yes
Ah	Sa	-2.4381	0	Yes
Ah	Sm	-1.4344	0.0059	Yes
Ah	Sy	-2.0453	0	Yes
Ah	Tc	-4.9818	0	Yes
Bs	Dh	-0.6596	0.9952	No
Bs	Hc	2.2259	0	Yes
Bs	Ikg	1.9232	0	Yes
Bs	Km	-1.7952	0.0058	Yes
Bs	Ly	2.8332	0	Yes
Bs	Rl	0.9526	0.2018	No
Bs	Sa	1.7727	0.0002	Yes
Bs	Sm	2.7764	0	Yes
Bs	Sy	2.1655	0	Yes
Bs	Tc	-0.771	0.6067	No
Dh	Hc	2.8854	0.0001	Yes
Dh	Ikg	2.5828	0.0004	Yes
Dh	Km	-1.1356	0.8414	No
Dh	Ly	3.4928	0	Yes
Dh	Rl	1.6122	0.168	No
Dh	Sa	2.4323	0.0021	Yes
Dh	Sm	3.436	0	Yes
Dh	Sy	2.8251	0.0001	Yes
Dh	Tc	-0.1114	1	No
Hc	Ikg	-0.3027	0.9982	No

Hc	Km	-4.021	0	Yes
Hc	Ly	0.6074	0.6808	No
Hc	Rl	-1.2732	0.0025	Yes
Hc	Sa	-0.4531	0.9764	No
Hc	Sm	0.5506	0.9496	No
Hc	Sy	-0.0603	1	No
Hc	Tc	-2.9968	0	Yes
Ikg	Km	-3.7183	0	Yes
Ikg	Ly	0.91	0.0334	Yes
Ikg	Rl	-0.9706	0.0249	Yes
Ikg	Sa	-0.1504	1	No
Ikg	Sm	0.8532	0.3786	No
Ikg	Sy	0.2424	0.9999	No
Ikg	Tc	-2.6942	0	Yes
Km	Ly	4.6284	0	Yes
Km	Rl	2.7478	0	Yes
Km	Sa	3.5679	0	Yes
Km	Sm	4.5716	0	Yes
Km	Sy	3.9607	0	Yes
Km	Tc	1.0242	0.4101	No
Ly	Rl	-1.8806	0	Yes
Ly	Sa	-1.0605	0.0255	Yes
Ly	Sm	-0.0568	1	No
Ly	Sy	-0.6677	0.617	No
Ly	Tc	-3.6042	0	Yes
Rl	Sa	0.8201	0.2739	No
Rl	Sm	1.8238	0	Yes
Rl	Sy	1.2129	0.0108	Yes
Rl	Tc	-1.7236	0	Yes
Sa	Sm	1.0037	0.252	No
Sa	Sy	0.3928	0.9947	No
Sa	Tc	-2.5437	0	Yes
Sm	Sy	-0.6109	0.9188	No

Sm	Tc	-3.5474	0	Yes
Sy	Tc	-2.9365	0	Yes

Moreover, the above Tukey test results also cause us confusion. That is, as can be seen from Figure R34, the multi-year mean $\delta^{18}O_p$ values of Sm (-4.41‰) and Ly (-4.35‰) are similar. However, the above Tukey test results show that there is no significant difference between the multi-year mean $\delta^{18}O_p$ values of Sa and Sm (green highlighting), but there is a significant difference between Sa and Ly (green highlighting).

We believe that when the geographical distances between two sites are very large, the multi-year mean $\delta^{18}O_p$ values more easily undergo sudden jumps, compared with the shorter distance between two sites. In such cases, the multi-year mean $\delta^{18}O_p$ values of these two sites can easily be detected by the Tukey test as showing significant differences. However, in our study, the low $\delta^{18}O_p$ values gradual westward migration, especially when the two sites were very close to each other, the changes in the $\delta^{18}O_p$ values of these two sites were small, and it was difficult to observe significant differences. Therefore, the $\delta^{18}O_p$ values of these two sites were not easily detected by the Tukey test. This is because the ITCZ, IPWP, and WPSH, exhibit gradual westward migration over time rather than experiencing sudden jumps. For instance, Ly and Sm are very close, and the difference in multi-year mean $\delta^{18}O_p$ values is very small (Figure R34). Therefore, the Tukey test showed that there was no “significant” difference in multi-year mean $\delta^{18}O_p$ values between Ly and Sm (orange highlighting). The similar result can also be found at the two sites of Hc and the Ikg (orange highlighting) (Table R12). To avoid confusing the readers as well, our study does not intend to use the Tukey test for analysis.

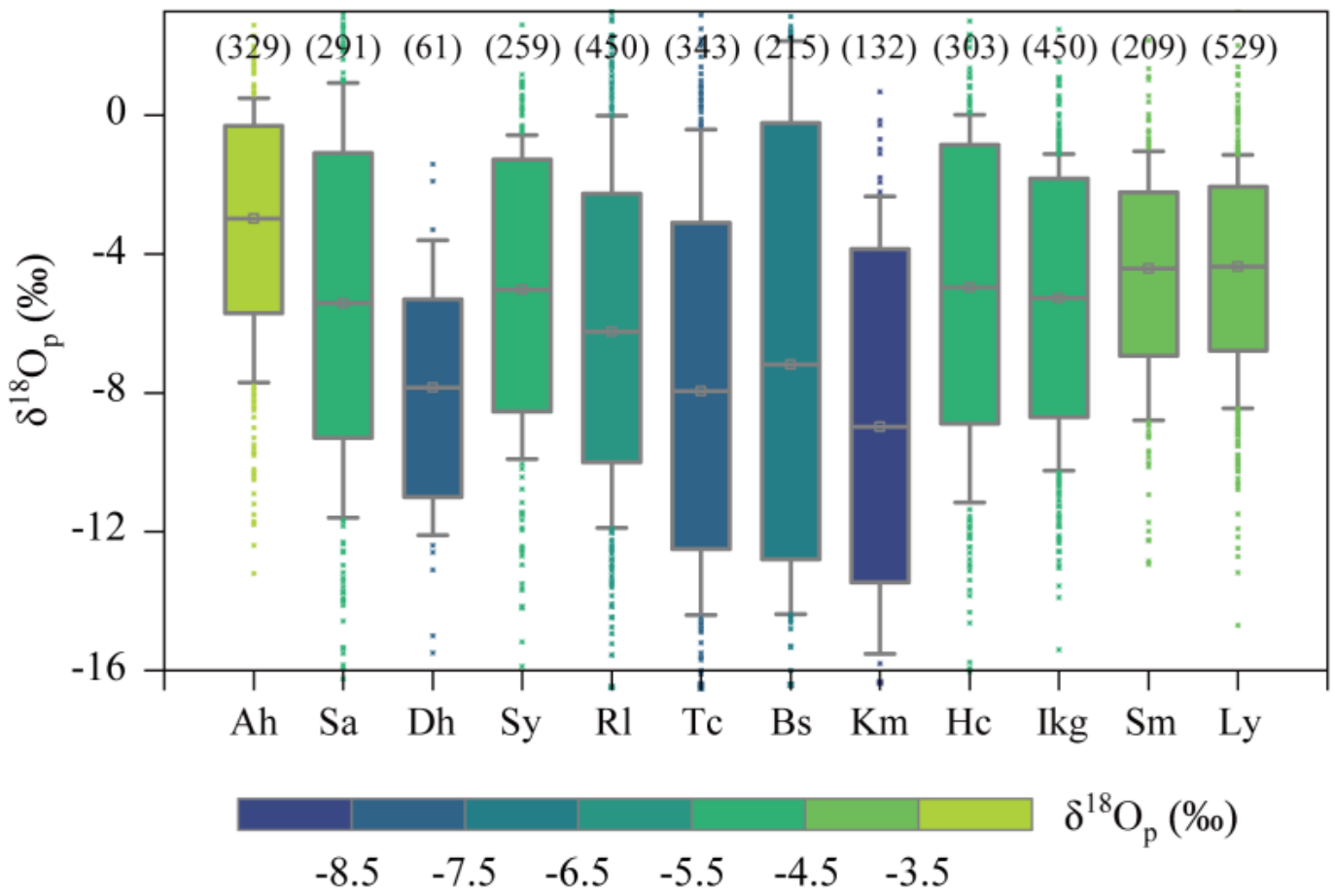

Figure R34. Spatial distributions of observed annual $\delta^{18}O_p$ at the 12 primary sites along the east–west transect. In each box plot, the box represents the 20th–80th percentile range, whiskers extend to the 10th–90th percentile, horizontal lines inside the boxes indicate the mean, and dots represent outliers. The number of measurements for each box was provided in the brackets. $\delta^{18}O_p$ values are expressed as per mil (‰) deviations relative to VSMOW or VSMOW2.

We admit that the V-shaped spatial pattern of the multi-year mean $\delta^{18}O_p$ values along the transect is not very obvious in Figure R34 (Figure S21 in the original Supplementary Information), which might be due to the fact that the X-axis is not longitude but the site number, giving rise to an illusion. When we set the X-axis as longitude, it can be seen that a clear V-shaped pattern (as shown in Figure R35) does exist.

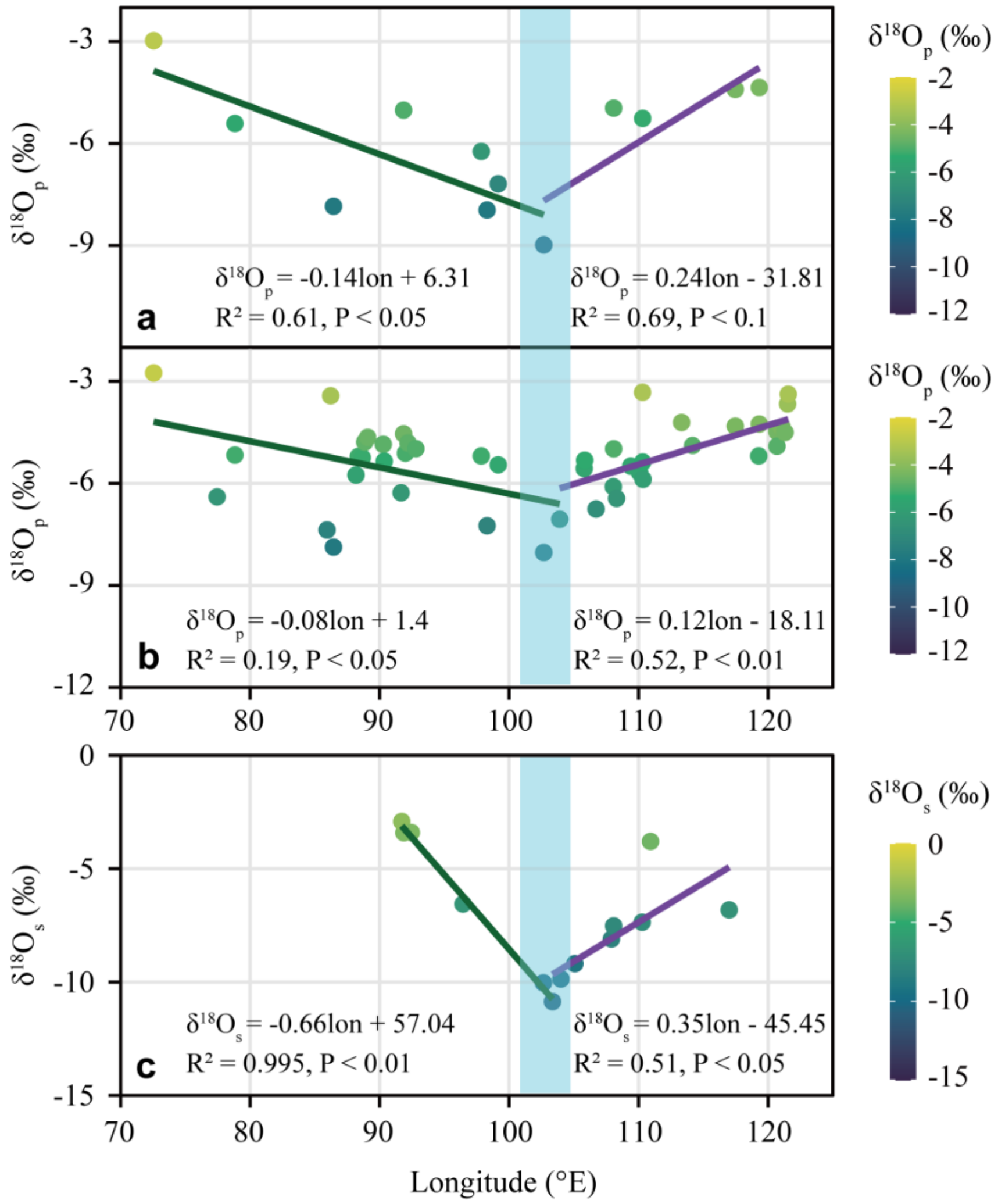

Figure R35. Trends of the multi-year mean $\delta^{18}O_p$ and $\delta^{18}O_s$ values from the west to the middle and from the middle to the east of the transect. A, The 12 primary precipitation sampling sites. b, The 46 precipitation sampling sites across the study region. c, The 13 caves along the transect. Blue-green shading indicates the location of the lowest $\delta^{18}O$ value. $\delta^{18}O_p$ values are expressed as per mil (‰) deviations relative to VSMOW or VSMOW2. $\delta^{18}O_s$ values are reported as per mil (‰) deviations from Vienna Peedee belemnite (VPDB).

2) With regard to an additional boxplot of monsoon vs. pre-monsoon season $\delta^{18}O_p$ at the 12 primary sites, following your comments, we analyzed the spatial distribution of $\delta^{18}O_p$ values at the 12 primary sites during pre-monsoon and monsoon seasons (Figure R36). We found that a distinct V-shaped spatial pattern in $\delta^{18}O_p$ values along the transect during the monsoon season (Figure R36b), this V-shaped pattern was even

more pronounced than the multi-year mean $\delta^{18}O_p$ value (Figure R34), and it matched the $\delta^{18}O_s$ pattern. Conversely, the pre-monsoon season exhibits a clear "low in the east, high in the west" spatial pattern (Figure R36a). Considering that there are already many figures in the Supplementary Information and this result is repetitive with the results for each month, we do not plan to include this figure in the Supplementary Information.

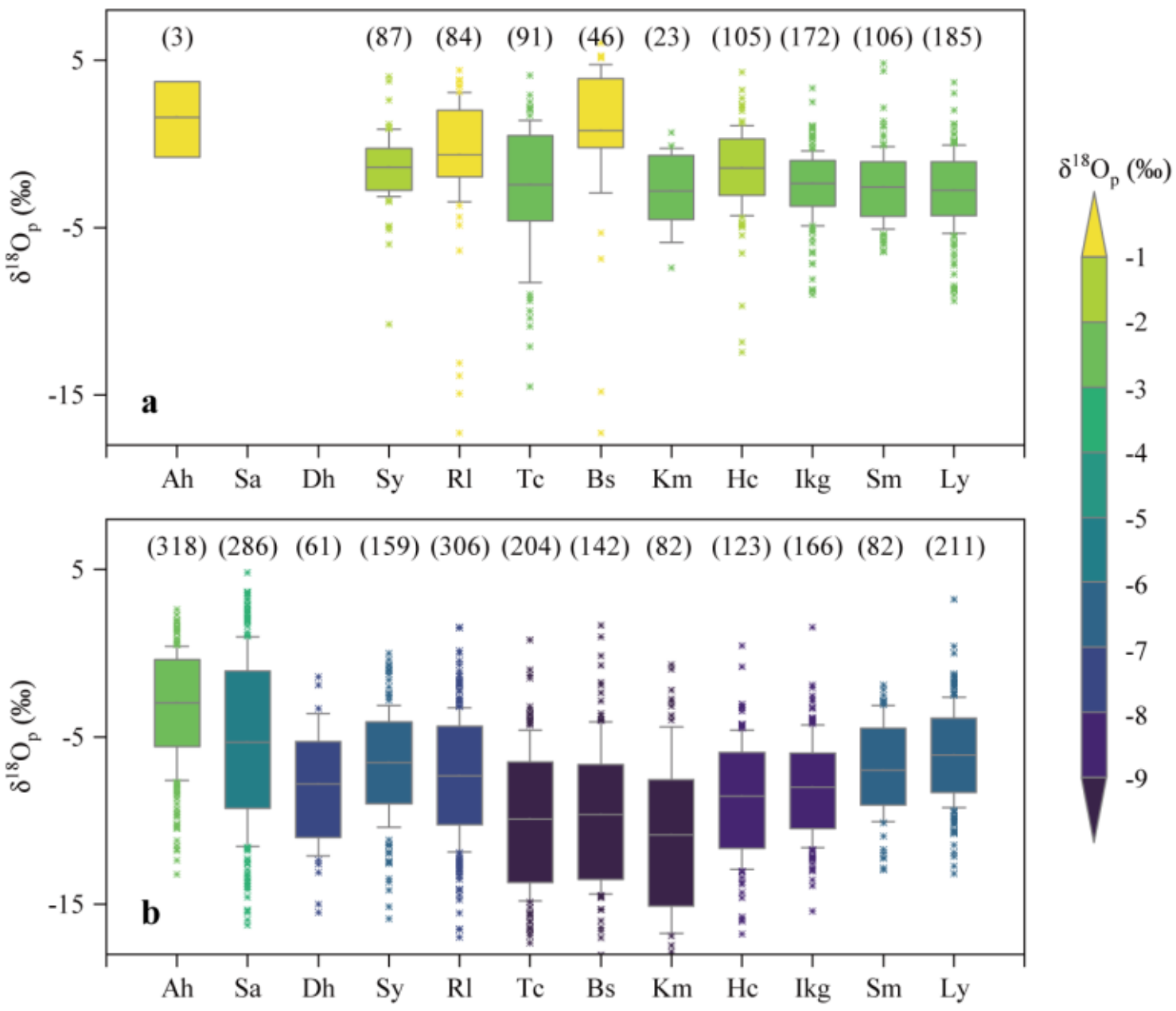

Figure R36. Spatial distributions of $\delta^{18}O_p$ at the 12 primary sites along the east–west transect during the pre-monsoon and monsoon seasons. (a) $\delta^{18}O_p$ distributions during the pre-monsoon season, (b) $\delta^{18}O_p$ distributions during the monsoon season. In each box plot, the box represents the 20th–80th percentile range, whiskers extend to the 10th–90th percentile, horizontal lines inside the boxes indicate the mean, and dots represent outliers. The number of measurements for each box was provided in the brackets.

3) With regard to the correlation/regression between $\delta^{18}O_s$ and $\delta^{18}O_p$, it is very difficult to establish a correlation between the V-shaped patterns of $\delta^{18}O_p$ and that of $\delta^{18}O_s$, because that the geographical locations of the $\delta^{18}O_p$ sites and $\delta^{18}O_s$ -recording caves do not correspond one-to-one. To verify the application of our findings to speleothem records, we followed your suggestion and added a figure to show the trends of the multi-year mean $\delta^{18}O_p$ and $\delta^{18}O_s$ values from the west to the middle and from the middle to the east of the transect (Figure R19). From this figure, we can

compare the spatial distribution of observed $\delta^{18}O_p$ data from 12 primary sites with the spatial distribution of observed $\delta^{18}O_p$ data from all 46 sites along the east-west transect, and with the spatial distribution of multi-year mean $\delta^{18}O_s$ values. We found that: a) The locations of the lowest values of the three V-shaped patterns appear at the similar longitude (~103°E). b) West of 103°E, both $\delta^{18}O_p$ and $\delta^{18}O_s$ show a significant negative correlation with longitude; east of 103°E, both $\delta^{18}O_p$ and $\delta^{18}O_s$ show a significant positive correlation with longitude. These results indicate that the V-shaped pattern of $\delta^{18}O_s$ matches that of $\delta^{18}O_p$. To address this issue, we add a paragraph, i.e., "*To further verify the implications for interpreting Asian $\delta^{18}O_s$ records, we analyzed the spatial trends of the multi-year mean $\delta^{18}O_s$ values and the annual $\delta^{18}O_p$ observation data from 12 primary sites and all 46 sites along the transect. Our data indicate that the lowest values of all three V-shaped patterns appear at a similar longitude (~103°E; Supplementary Fig. 20). Moreover, west (east) of 103°E, the decreases (increases) in the $\delta^{18}O_s$ and $\delta^{18}O_p$ values with increasing longitude are significant (Supplementary Fig. 20).*" Please see L416–L422 in the revised manuscript.

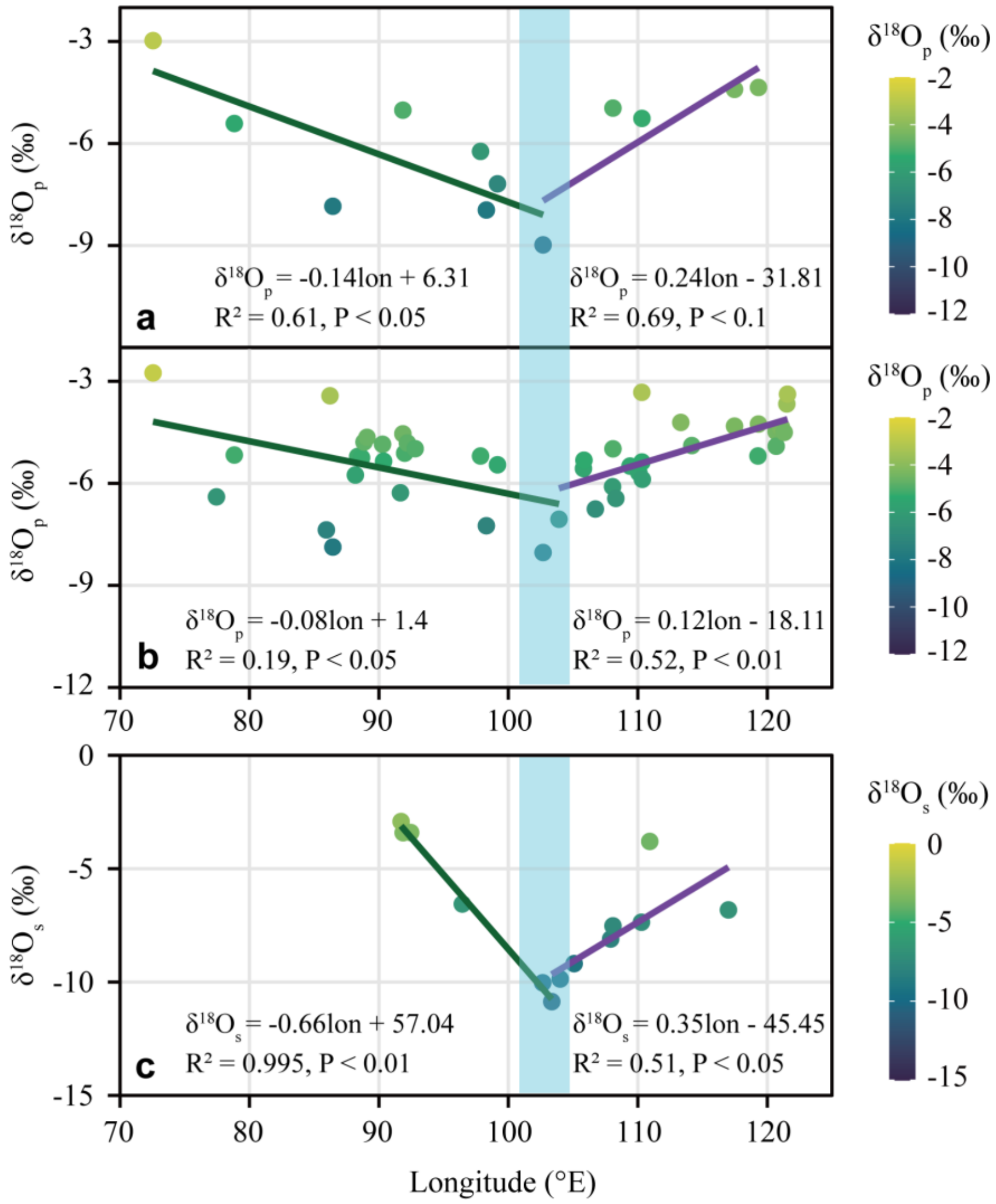

Figure R37. Trends of the multi-year mean $\delta^{18}O_p$ and $\delta^{18}O_s$ values from the west to the middle and from the middle to the east of the transect. A, The 12 primary precipitation sampling sites. b, The 46 precipitation sampling sites across the study region. c, The 13 caves along the transect. Blue-green shading indicates the location of the lowest $\delta^{18}O$ value. $\delta^{18}O_p$ values are expressed as per mil (‰) deviations relative to VSMOW or VSMOW2. $\delta^{18}O_s$ values are reported as per mil (‰) deviations from Vienna Peedee belemnite (VPDB).

Additionally, following your suggestion, we marked the locations of all 13 caves on the original Fig. S22 and labeled them with their corresponding multi-year mean $\delta^{18}O_s$ values (Figure R20). The results demonstrate that the spatial pattern of multi-year mean $\delta^{18}O_s$ values matches well with the that of the IsoGSM2 simulated $\delta^{18}O_p$ along this transect. To address this issue, we added corresponding explanations, i.e., "*Additionally, the spatial distribution of multi-year mean $\delta^{18}O_s$ values closely*

resembles the annual IsoGSM2–simulated $\delta^{18}O_p$ values (1979–2022), both exhibiting a consistent V-shaped pattern (Supplementary Fig. 21)." Please see L422–L425 in the revised manuscript.

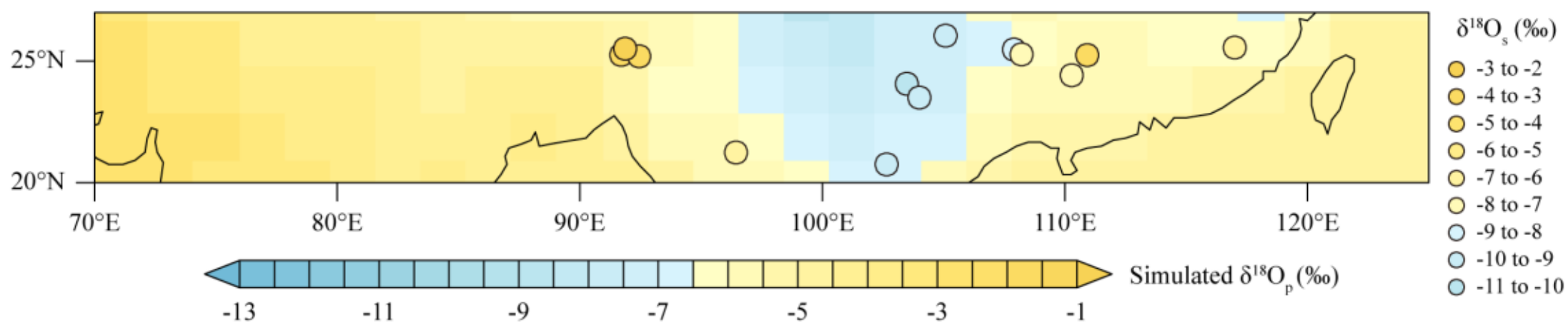

Figure R38. Spatial distribution of IsoGSM2-simulated annual $\delta^{18}O_p$ across the study region over 1979–2022. Multi-year mean $\delta^{18}O_s$ values from 13 caves (dot) across the study region are also shown. $\delta^{18}O_p$ values are expressed as per mil (‰) deviations relative to VSMOW/VSMOW2. $\delta^{18}O_s$ values are reported as per mil (‰) deviations from Vienna Peedee belemnite (VPDB).

Methods

The authors state they obtained "vertical velocity (ω), OLR, specific humidity, meridional wind, zonal wind, geopotential height, precipitation amount and cloud cover", but only the OLR seems to be analysed. Have the others been analysed for their significance? If these aren't important, is it necessary to include them in the methods? Based on some of my other comments, though, I would like to see some (at least precipitation amount) considered and the specific climatologies at the cave sites considered.

Re: We thank you for pointing these issues out. We addressed these issues as follows:

1) With regard to not included other variables, it should be noted that the additional variables collected in our study are not used to discuss their relationships with $\delta^{18}O_p$, but used to delineate the Intertropical Convergence Zone (ITCZ) and other things. Following your comment, we added two paragraphs in the "Materials and Methods" section to explain the utility of other variables in our study:

"*These precipitation amount data were used to analyze the proportions of precipitation during the different seasons to the annual precipitation and to analyze the spatial distributions of precipitation amount during the pre-monsoon and monsoon seasons.*

The study utilized the ERA5 reanalysis data (0.25°×0.25° resolution) provided by the European Centre for Medium-Range Weather Forecasts (ECMWF). The ERA5 reanalysis data on single levels are available from https://cds.climate.copernicus.eu/cdsapp#!/dataset/reanalysis-era5-single-levels-monthly-means?tab=form, following ref. ***Error! Reference source not found.****. The ERA5 reanalysis data on pressure levels are available from https://cds.climate.copernicus.eu/cdsapp#!/dataset/reanalysis-era5-pressure-levels-monthly-means?tab=form, following ref.* ***Error! Reference source not found.****. Among them, vertical velocity (ω) and outgoing longwave radiation (OLR) were used to quantify the intensity of atmospheric updraft (negative and positive ω values indicate upward and downward motions, respectively). The lower the ω and OLR, the stronger the atmospheric updraft. We used specific humidity combined with meridional and zonal winds to calculate the moisture flux. The geopotential height was used to assess the western Pacific subtropical high intensity. Precipitation and cloud cover were used to delineate the Intertropical Convergence Zone (ITCZ). In addition, sea surface temperature (SST) data (1°×1° resolution) provided by HadISST1 (https://www.metoffice.gov.uk/hadobs/hadisst/)*[Error! Reference source not found.] *were used to evaluate Indo-Pacific Warm Pool (IPWP) variability.*" Please see L734–L755 in the revised manuscript.

2) With regard to "precipitation amount", according to the study of Dansgaard (1964), the precipitation "amount effect" (characterized by a negative correlation between $\delta^{18}O_p$ and precipitation amount) mainly occurs in low latitudes, especially in tropical regions. Since the following reasons, in this study we eliminated the influence of precipitation amount on $\delta^{18}O_p$.

i) Numerous previous studies have already discussed the relationship between $\delta^{18}O_p$ and precipitation amount. If we were to further analyze the relationship between $\delta^{18}O_p$ and precipitation amount in this study, it would seem somewhat repetitive and would seriously undermine the novelty of this study.

ii) Previous paleoclimate studies shown that the Asian $\delta^{18}O_s$ records exhibit a remarkable spatial consistency across different timescales during the Holocene (Chen et al., 2016; Liu et al., 2015). However, there are significant spatial differences in precipitation amount at different time scales in East Asia, and there is a clear conflict

between the $\delta^{18}O_s$ and precipitation amount. Consequently, further discussion of precipitation as a climatic variable for interpreting $\delta^{18}O_s$ record would be unhelpful. This is why our study early eliminated precipitation amount as a potential factor in the Introduction section.

iii) Aggarwal et al. (2016) demonstrated that the precipitation “amount effect” is extremely weak at multi-year monthly average scale in low-latitude regions (the negative correlation is very weak, when the precipitation ⩾ 300 mm, there is an anomalous positive correlation) (Figure. R39). This implies that further discussion of precipitation amount effect would be of little significance.

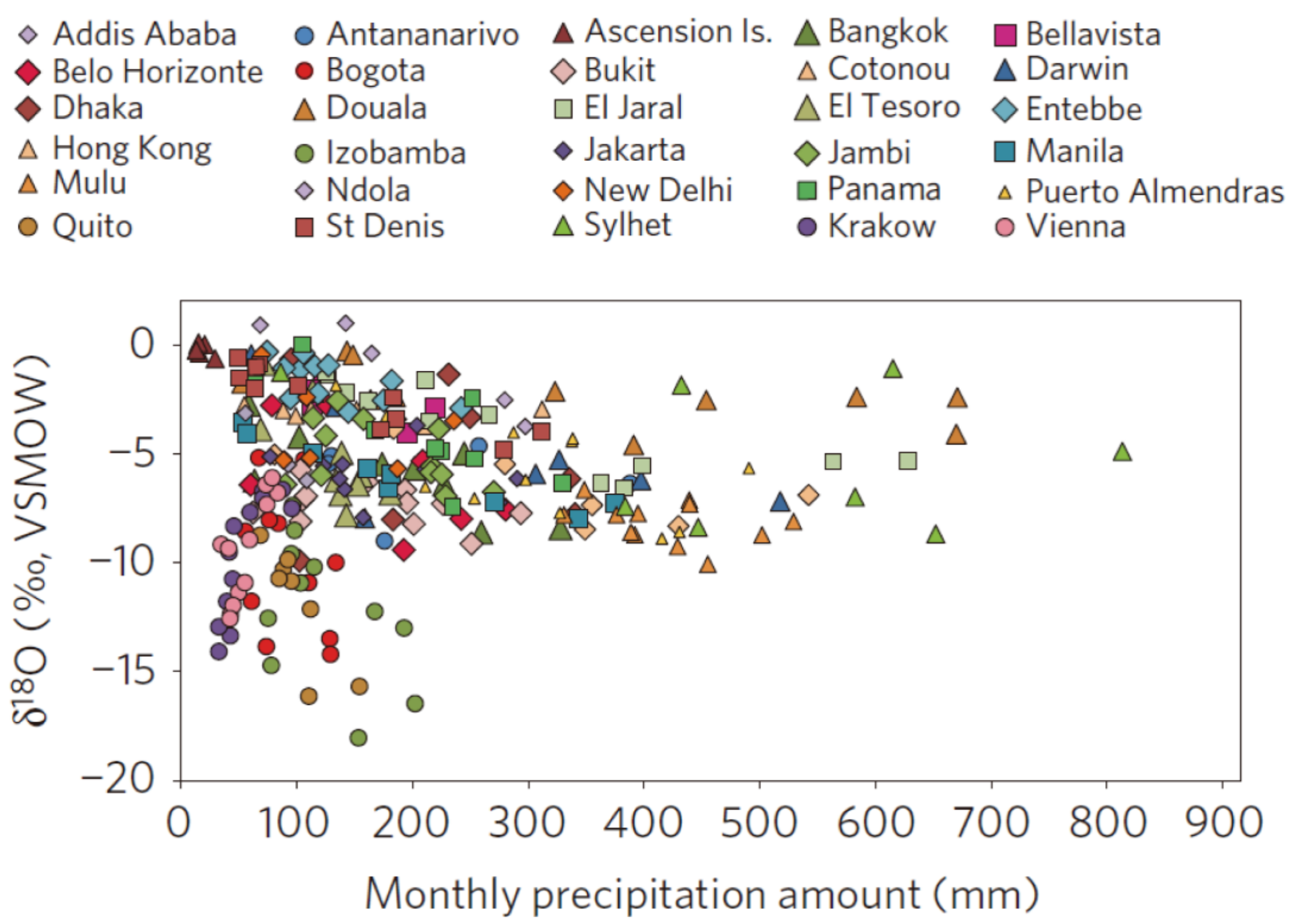

Figure R39. Correlation of mean monthly $\delta^{18}O$ and precipitation amount in tropical and midlatitude precipitation. Note the limited change in $\delta^{18}O$ with the higher precipitation amount.

iv) More importantly, we used a unique perspective provided by you to eliminate the influence of precipitation amount. Specifically, following your suggestions, we analyzed the spatial pattern of precipitation amount along the transect during the monsoon season. As we propose that $\delta^{18}O_P$ values along the transect show a

"V-shaped" pattern, that is, higher $\delta^{18}O_p$ values at the eastern and western margins and lower values in the middle. If precipitation amount affects the V-shaped pattern of $\delta^{18}O_p$ along the east–west transect, precipitation amount along the transect should appear as an "inverted V-shaped" pattern during the monsoon season. However, our precipitation amount data do not show an "inverted V-shaped" pattern (Figure R40), which can not explain the V-shaped pattern of $\delta^{18}O_p$. More important, a notable discrepancy was observed at the Sylhet site, where monthly precipitation amount is exceptionally high, yet the $\delta^{18}O_p$ at this site did not exhibit the correspondingly expected low values (Figure R40). Instead, the $\delta^{18}O_p$ at this site remained relatively high. This result conflicted with the theory of precipitation "amount effect." Therefore, it can be seen that the spatial pattern of precipitation amount is inconsistent with that of the $\delta^{18}O_p$ along the transect. Thus, we can conclude that the $\delta^{18}O_p$ along the transect shows little correlation with the precipitation amount. We added a figure to show the spatial pattern of precipitation amount along the east–west transect (Please see Figure R40 or Fig. S15 in the revised Supplementary Information), and we added a paragraph in the manuscript to eliminate the influence of precipitation amount on $\delta^{18}O_p$ along the transect. i.e., "*It could also be proposed that terrain-enhanced updrafts might increase precipitation, linking $\delta^{18}O_p$ to precipitation amount. If true, monsoon-season precipitation should show an inverted V-shaped pattern along the transect. However, our results do not support this hypothesis as precipitation amount in the middle is relatively low while it is considerably higher at Sylhet in the western domain (Supplementary Fig. 15).*" Please see Fig. S15 in the revised Supplementary Information and L320–L325 in the revised manuscript.

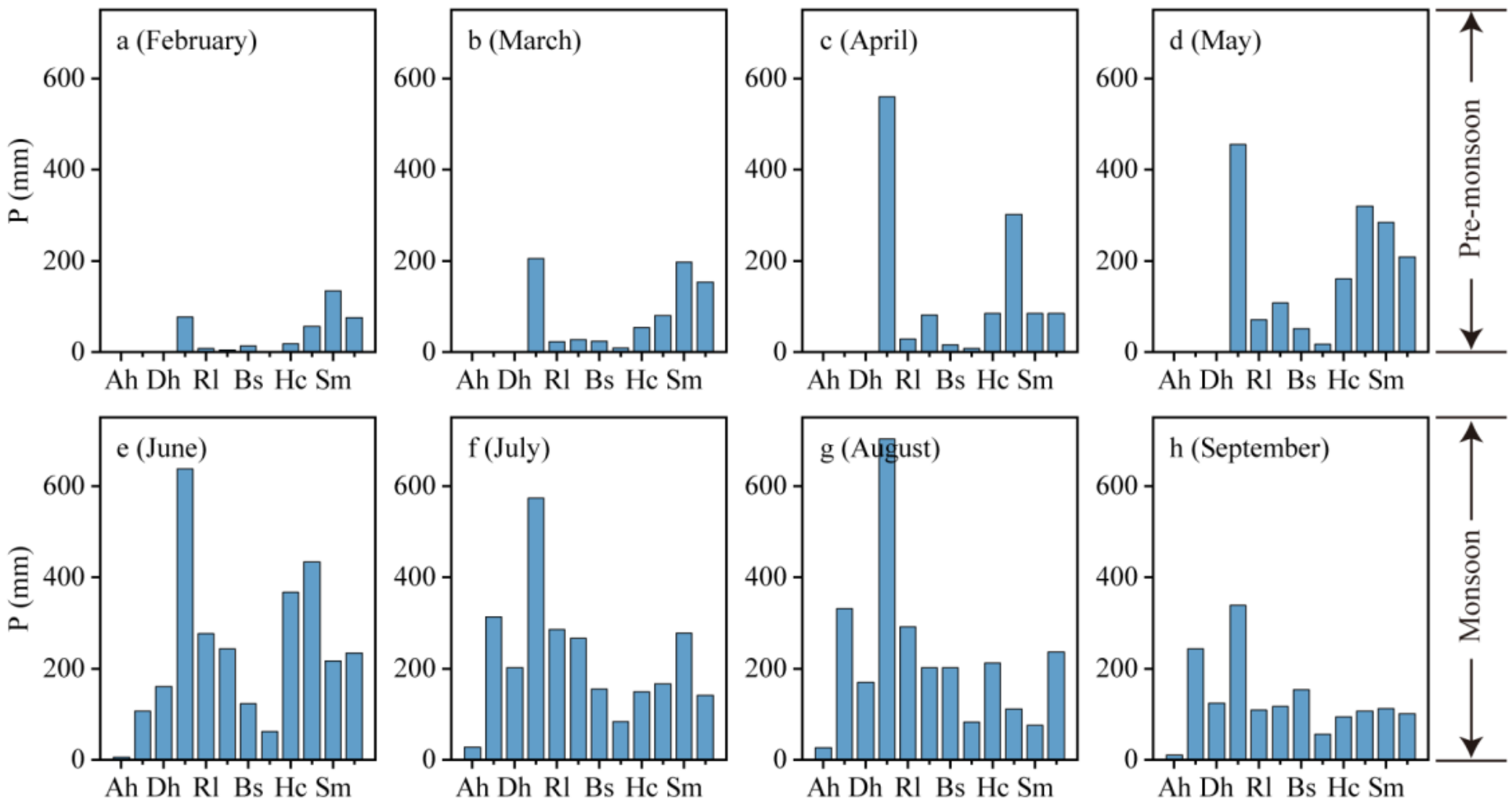

Figure R40. Spatial distributions of precipitation amount at the 12 primary sites along the transect during the pre-monsoon and monsoon seasons. a–d, Pre-monsoon season. e–h, Monsoon season. Abbreviations: Ah (Ahmedabad), Dh (Dhanbad), Rl (Ruili), Bs (Baoshan), Hc (Hechi), and Sm (Sanming).

References:

Aggarwal, P. K. et al. Proportions of convective and stratiform precipitation revealed in water isotope ratios. *Nat. Geosci.* **9**, 624–629 (2016).

Chen, J. et al. On the timing of the East Asian summer monsoon maximum during the Holocene—Does the speleothem oxygen isotope record reflect monsoon rainfall variability? *Sci. China Earth Sci.* **59**, 2328–2338 (2016).

Dansgaard, W. Stable isotopes in precipitation. *Tellus* **16**, 436–468 (1964).

Liu, J. et al. Holocene East Asian summer monsoon records in northern China and their inconsistency with Chinese stalagmite δ18O records. *Earth Sci. Rev.* **148**, 194–208 (2015).

Yu, W. et al. Temperature signals of ice core and speleothem isotopic records from Asian monsoon region as indicated by precipitation $\delta^{18}O$. *Earth Planet. Sci. Lett.* **554**, 116665 (2021).

Calculation of multi-year mean $\delta^{18}O_s$ values

There are insufficient details here about the relative value method and what the authors did to achieve the results displayed in Fig. 6b. Also, can you please explain how and why these sub-periods were defined?

Re: We thank you for pointing these issues out. Following your suggestions, we added four paragraphs in the Methods section to provide detailed explanations of how these "relative values" were calculated and how the sub-periods were defined. i.e., "*The $\delta^{18}O_s$ records from the 13 caves analyzed in this study span different time periods over the last 50,000 years (Fig. 7a). To more clearly compare $\delta^{18}O_s$ records for assessing spatial patterns, we calculated the multi-year mean $\delta^{18}O_s$ value for each cave using a "relative value" method. To calculate the "relative" $\delta^{18}O_s$ values as accurately as possible, we divided the $\delta^{18}O_s$ records into seven sub-periods (Periods I –VII) (Fig. 7a) following two criteria: (1) we ensured that as many $\delta^{18}O_s$ records as possible were available for each sub-period; and (2) all our grouped sub-periods were designed to cover the entire period (the past 50,000 years) as comprehensively as possible.*

Based on the distribution characteristics of $\delta^{18}O_s$ records over the past 50,000 years, we calculated the multi-year mean $\delta^{18}O_s$ values for each record within their respective sub-periods: Period I (Fengyu, Xiangshui, Xiaobailong, and Southeast Yunnan), Period II (Mawmluh, Wulu, and Xianyun), Period III (Lin Noe Twin), Period IV (Cherrapunji), Period V (Yamen and Dongge), Period VI (Tham Doun Mai), and Period VII (Wah Shikar). We used the Fengyu $\delta^{18}O_s$ record as a reference, as it provided the longest continuous series (Fig. 7a). For Period I, we calculated the multi-year mean $\delta^{18}O_s$ values directly by using the $\delta^{18}O_s$ records from Fengyu, Xiangshui, Xiaobailong, and Southeast Yunnan caves. For Periods II –VI, we used the multi-year mean $\delta^{18}O_s$ value from Fengyu Cave in Period I as the base value. The respective $\Delta\delta^{18}O_s$ values for Fengyu cave in Periods II –VI were then calculated by subtracting the base value from the respective Fengyu $\delta^{18}O_s$ records during Periods II-VI, respectively. Obviously, the $\Delta\delta^{18}O_s$ values for Fengyu cave are different in Periods II-VI. Finally, we obtained the multi-year mean $\delta^{18}O_s$ values during Periods II-VI by subtracting the corresponding $\Delta\delta^{18}O_s$ value in each period from the corresponding $\delta^{18}O_s$ records in each period, respectively.

For example, to calculate the multi-year mean $\delta^{18}O_s$ values for the three specific $\delta^{18}O_s$ records in Period II (Mawmluh, Wulu, and Xianyun), we first determined the Fengyu $\Delta\delta^{18}O_s$ value (0.07‰) in Period II by subtracting the Fengyu multi-year mean $\delta^{18}O_s$ value (-7.36‰) in Period I from the Fengyu multi-year mean $\delta^{18}O_s$ value (-7.29‰) in Period II. Finally, we obtained the multi-year mean $\delta^{18}O_s$ values for the

Mawmluh, Wulu, and Xianyun caves by subtracting the Δ$\delta^{18}O_s$ values (0.07‰) from the Mawmluh, Wulu, and Xianyun $\delta^{18}O_s$ records in Period II, respectively.

Notably, the Fengyu $\delta^{18}O_s$ record does not extend into Period VII, where the Wah Shikar $\delta^{18}O_s$ record is the only available dataset (Fig. 7a). For this sub-period, we calculated the multi-year mean $\delta^{18}O_s$ value for Wah Shikar cave using the Mawmluh $\delta^{18}O_s$ record as a reference, given its closer geographic proximity. This approach allowed us to derive relative $\delta^{18}O_s$ values and compute multi-year mean $\delta^{18}O_s$ values for each cave." Please see L775–L811 in the revised manuscript.

682-683: Not quite sure what the relevance of including Fig. S23 here is?

Re: We thank you for pointing this issue out. Following your comment, we check lines 682-683, and found that it is not needed to cite Fig, S23. So, we deleted the reference of the figure. Please see L775–L778 in the revised manuscript.

Minor line comments

Abstract

57-60: This is confusingly written and ought to be improved for clarity.

Re: We thank you for pointing this issue out. Following your comments, we changed "Stronger atmospheric updraft leads to lower condensation temperatures and enhanced "rainout effect" during a prolonged precipitation event, resulting in lower $\delta^{18}O_p$ values" to "*Stronger atmospheric updraft leads to lower $\delta^{18}O_p$ values through two mechanisms: (1) reduced condensation temperatures and (2) enhanced "rainout effect" on local scales (from the surface to higher altitudes) during a continuous precipitation event.*" Please see L58–L61 in the revised manuscript.

There is some need for clarity on the mentioned effects. "Rain out" seems to have been used twice to mean different things. Do you mean the "Continental effect" (Dangaard, 1964)?

Re: We thank you for pointing this issue out. In general, the "rainout effect" refers to the process in which, during the moisture transport from source regions to precipitation sites, the gradual rainout and increasingly depleted composition of water vapor along its trajectory. The farther from its evaporative source that water vapor contributes to local precipitation, the more negative is therefore the correlation with

the local $\delta^{18}O$ signal (Vuille et al., 2003). Hence, the "rainout effect" is not exactly the same as the "Continental effect." Certainly, when moisture is transported from the coast to the land, the isotopes in subsequent precipitation also become more depleted ("continental effect") (Dansgaard, 1964). In this case, the influence of "continental effect" on $\delta^{18}O_p$ is similar to that of the "rainout effect" on $\delta^{18}O_p$.

In the main text, we introduced the "rainout effect" in two sections: (1) in the "$\delta^{18}O_p$ and the advection of monsoon moisture" section, i.e., "*Previous studies have focused mainly on the "rainout effect" along the advection pathway as moisture travels from its source to a precipitation site*[49,50]*. However, they have overlooked the "rainout effect" driven by atmospheric updraft that occurs directly at precipitation sites*[49,50]*, especially during a continuous precipitation event (i.e., the duration of a continuous precipitation event is generally several hours or even several days).*" Please see L215–L220 in the revised manuscript. and (2) in the "$\delta^{18}O_p$ and atmospheric updraft" section, i.e., "*Since atmospheric updraft cannot be ignored, it is important to understand how it affects $\delta^{18}O_p$ changes at precipitation sites. During a precipitation event, as air masses are lifted to higher altitudes with lower condensation temperatures, water isotopes progressively undergo enhanced Rayleigh fractionation, eventually leading to lower moisture $\delta^{18}O$ and consequently lower $\delta^{18}O_p$ values*[38,50]*. Importantly, during the formation of the "first-time" raindrops, moisture isotopes experience a substantial "rainout effect", lowering the $\delta^{18}O$ values of the residual moisture*[66,67]*. When atmospheric updraft intensifies, residual moisture rises further and condenses to form "second-time" raindrops with even lower $\delta^{18}O_p$ values*[68]*. This process continues, with each successive raindrop having lower $\delta^{18}O_p$ values. Thus, stronger updraft increases uplift altitudes, reduce condensation temperatures, and amplifies the "rainout effect", further lowering $\delta^{18}O_p$*[66-68]." Please see L225–L236 in the revised manuscript. Due to the word limit in the abstract, detailed explanations are not provided here.

The above analysis shows that while the term "rainout effect" is used twice to describe the same physical process, the two "rainout effects" operate in different directions. To help readers distinguish these differences, we changed "Stronger atmospheric updraft leads to lower condensation temperatures and enhanced "rainout effect" during a prolonged precipitation event, resulting in lower $\delta^{18}O_p$ values. This

process masks the influence of the "rainout effect" on $\delta^{18}O_p$ along monsoon moisture advection." to "*Stronger atmospheric updraft leads to lower* $\delta^{18}O_p$ *values through two mechanisms: (1) reduced condensation temperatures and (2) enhanced "rainout effect" on local scales (from the surface to higher altitudes) during a continuous precipitation event*." Please see L58–L61 in the revised manuscript.

Reference:

Vuille, M., Bradley, R. S., Werner, M., Healy, R. & Keimig, F. Modeling $\delta^{18}O$ in precipitation over the tropical Americas: 1. Interannual variability and climatic controls. *J. Geophys. Res.* **108**, 4174 (2003).

Line 57: "the" Asian monsoon region (definite article)

Re: Following your suggestion, we added "the" before "Asian monsoon region." Please see L58 in the revised manuscript.

Main text

Line 88: if word count permits, a brief description of the "circulation effect" and "inverse temperature effect" would be useful for the general readership.

Re: We thank you for pointing these issues out. Following your comments, we added a brief description of the "circulation effect" and "inverse temperature effect", i.e., "*Recently, several hypotheses have emerged to explain* $\delta^{18}O_p$ *variability across the Asian monsoon region*[39-42]*, including the "circulation effect" where* $\delta^{18}O_p$ *is dependent on the ratio of water vapor originating from distant oceans and local ocean to an observed site*[42] *and the "inverse temperature effect" where* $\delta^{18}O_p$ *and surface temperature show consistent inverse correlations*[38]." Please see L91–L95 in the revised manuscript.

Line 93: Evidences -> Evidence (uncountable noun)

Re: Following your suggestion, we changed "Evidences" to "Evidence." Please see L100 in the revised manuscript.

Line 125: "the" South Asian monsoon region (definite article)

Re: Following your suggestion, we added "the" before "South Asian monsoon

region." Please see L124 in the revised manuscript.

136: the authors claim that the 250-800 m range shows a significant 'correlation' (this is actually a regression), but looking at the p value, whilst close, it is above 0.05 and therefore does not fulfil the typical criteria for statistical significance.

Re: We thank you for pointing this issue out. In the previous manuscript we intended to indicate significance at the 90% confidence level (P<0.1). Now, following the comments provided by Reviewers #1 and #2, we classified the samplings sites into three altitude ranges, based on the definitions of UNEP-WCMC criteria. The results show that no significant negative correlations between $\delta^{18}O_P$ and altitude across these three altitude ranges, indicating the absence of an "altitude effect" on $\delta^{18}O_P$. We added relevant descriptions and updated the figures accordingly. Please see L309–L311 in the revised manuscript and the updated Fig. S14 in the revised Supplementary Information.

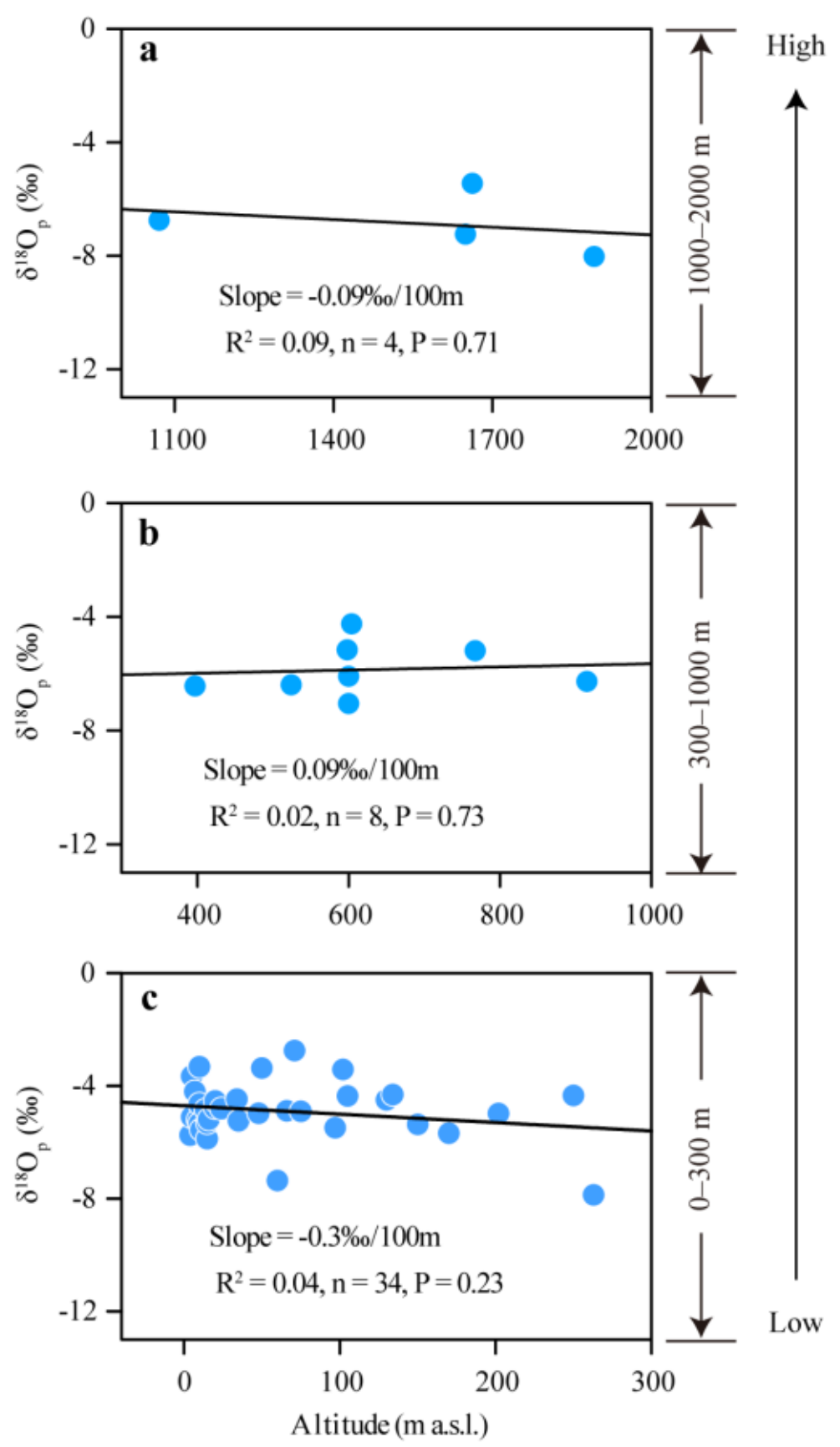

Figure R41. Correlations between observed $\delta^{18}O_P$ values and altitudes. The 46 precipitation sites were categorized into three altitude ranges based on the UNEP-WCMC mountain classification[2]. a–c, Correlations for the altitude ranges

1000–2000 m (a), 300–1000 m (b), and 0–300 m (c). $\delta^{18}O_p$ values are expressed as per mil (‰) deviations relative to VSMOW or VSMOW2.

150: the "12 primary sites" are not all included in the pre-monsoon. The 9 of the sites are.

Re: We thank you for pointing this issue out. Following your comment, we removed the "12" from the sentence. Please see L147 in the revised manuscript.

Line 151: follows -> follow (plural)

Re: Following your suggestion, we changed "follows" to "follow." Please see L147 in the revised manuscript.

Line 379: reflects -> reflect (plural)

Re: Following your suggestion, we changed "reflects" to "reflect." Please see L397 in the revised manuscript.

Line 687: Note "that" the (subordinating conjunction)

Re: We thank you for pointing this issue out. Following your previous comments, we rewrote this part, the revised manuscript has removed this sentence.

Line 680, 691, 810, 114, 145, 225, 320, 396, 401: multiyear -> multi-year (compound adjective)

Re: Following your suggestion, we changed "multiyear" to "multi-year." Please see L126, L138, L338, L413, L417, L774, L777, L784, L791, L796, L799, L801, L802, L803, L808, and L810 in the revised manuscript.

Figures

Fig. 2 and Fig. 3. To save on figure caption word count, the authors could label the boxes with their respective months.

Re: We thank you for pointing this issue out. We found that labeling the months in Figure 2 will make the figure unattractive. Following your suggestion, we instead marked the months in Figure 3. Please see the new Fig. 3 in the revised manuscript.

Fig. 3: line 771. The authors are missing “February” before the “(a)”?

Re: We are very sorry for our negligence. “February” should be added before the “(a).” In this figure we added correlations of $\delta^{18}O_p$ the corresponding altitudes at the 46 sites across the study region and seasonal changes of outgoing longwave radiation (OLR) and frequencies of relatively strong convection events occurred along the transect over 1979–2022 during the pre-monsoon and monsoon seasons. Following editorial requests, we only marked each panel, not marked each sub-figure. Hence, we deleted the sentence. Please see L901–L910 in the revised manuscript.

Fig. 4: correlation coefficients should be reported with an italicised “r” opposed to “R”. Since all discussion so far has focused on monthly/seasonal timescales, my personal opinion is that the correlation between daily $\delta^{18}O$ and OLR (Fig. 4a) isn’t necessary. Can the authors please also run their monthly $\delta^{18}O$ and OLR as a regression and plot the R^2 value? There is no y-axis on Fig. 4c,d,e,f; where do the gradients come from?

Re: We thank you for pointing these issues out. We addressed these issues as follows:

1) This study primarily focuses on examining whether a significant positive correlation exists between $\delta^{18}O$ and OLR. Therefore, we present only the correlation coefficient (r) rather than R^2 values. Moreover, considering that the width of Fig. 4g is too small to include an equation of the regression, we plotted the correlation coefficient (r) in Fig. 4g (Figure R42 as follows). To maintain consistent formatting throughout Fig. 4, we only display the correlation coefficient (r) in both Figs. 4a and 4b. The updated figure (Fig. 4) can be found in the revised manuscript.

2) We agree with you that the correlation between the daily $\delta^{18}O$ and OLR isn’t necessary. Following your suggestion, we deleted the correlation between daily $\delta^{18}O$ and OLR, kept the correlation between the monthly $\delta^{18}O$ and the corresponding OLR, and added the correlation between the multi-year mean monthly $\delta^{18}O$ and the corresponding OLR (Figure R42b).

3) With regard to the comment of “there is no y-axis on Fig. 4c,d,e,f”, following your comment, we added the y-axis labels on Fig. 4c,d,e,f (see revised Figure R42 panels c, d, e, and f below).

4) With regard to the gradients, we used a “based value” method. Due to word limit constraints in the main text, we added only a concise description in the Methods section, i.e., “***Calculation of slopes of $\delta^{18}O_p$ and OLR values***

To analyze the gradients of $\delta^{18}O_p$ and OLR values from east to west during the pre-monsoon and monsoon seasons, we standardized the $\delta^{18}O_p$ and OLR values at the 12 primary sites using the “base value” method (see Supplementary Information for details).” Please see L769–L773 in the revised manuscript. The detailed calculation procedures were provided in the revised Supplementary Information. i.e., “***Calculation of slopes of $\delta^{18}O_p$ and OLR values***

To analyze the gradients of precipitation isotope ($\delta^{18}O_p$) and outgoing longwave radiation (OLR) values from east to west during the pre-monsoon and monsoon seasons, we standardized the $\delta^{18}O_p$ and OLR values from 12 primary sites using the “base value” method. During the pre-monsoon season, lower $\delta^{18}O_p$ and OLR migrate progressively westward from Luoyuan (the easternmost site) to Kunming-Baoshan-Tengchong (the middle sites) along the east–west transect. From February to May, $\delta^{18}O_p$ and OLR show a spatial pattern of “low in the east and high in the west of the ~98° E dividing line”, yielding negative slopes from the middle to the easternmost sites. Hence, we used the monthly $\delta^{18}O_p$ and OLR values at Luoyuan as the base values. We then subtracted the corresponding base values from the monthly $\delta^{18}O_p$ and OLR at Luoyuan, Sanming, Institute of Karst Geology, Hechi, and Kunming for each month during the pre-monsoon season. Thus, the $\Delta\delta^{18}O_p$ and ΔOLR at Luoyuan are 0‰ and 0 W/m^2, respectively. Finally, we calculated the slopes of $\Delta\delta^{18}O_p$ and ΔOLR using the values from the five sites (Kunming, Hechi, Institute of Karst Geology, Sanming, and Luoyuan) for month, thereby deriving the spatial gradients (Fig. 4c, d).

During the monsoon season, the lower values of $\delta^{18}O_p$ and OLR appear around the middle of the transect, resulting in a V-shaped pattern. Consequently, $\delta^{18}O_p$ and OLR values increase from the middle to the easternmost site (Luoyuan). This implies that the gradients (slopes) from the middle to Luoyuan are positive. If the slopes are negative, this indicates that the lower values do not extend westward to that site. Hence, we define the site immediately east of such a site as the “critical site.” For standardization, the monthly $\delta^{18}O_p$ and OLR values at the critical site were used as

the base values. As the critical site is different from June to September, the base value also differ by month. We then normalized the $\delta^{18}O_p$ and OLR values at the critical site and other eastern sites by subtracting the corresponding base values. Finally, we calculated the slopes of the $\Delta\delta^{18}O_p$ and ΔOLR from the critical site to Luoyuan for each monsoon month.

Taking June as an example, both $\delta^{18}O_p$ and OLR slopes are positive when calculated from six sites (Baoshan, Kunming, Hechi, Institute of Karst Geology, Sanming, and Luoyuan). Including Tengchong yields negative slopes, indicating that Tengchong is the specific site in June. Therefore, Boshan, the site immediately east of Tengchong, is defined as the critical site. Thus, in June, we used Baoshan as the base site, setting $\Delta\delta^{18}O_p$ and ΔOLR at 0‰ and 0 W/m². We then standardized the values at all sites between Baoshan and Luoyuan by subtracting the base values, and calculated the slopes from these six sites.

In July-September, the specific sites were Ruili (July), Sylhet (August), and Dhanbad (September). Accordingly, their immediately adjacent eastern sites (Tengchong, Ruili, and Sylhet) served as the critical sites. We then calculated slopes from the seven sites (Tengchong, Baoshan, Kunming, Hechi, Institute of Karst Geology, Sanming, and Luoyuan) in July, eight sites in August (Ruili to Luoyuan), and nine sites in September (Sylhet to Luoyuan) (Fig. 4e, f)." Please see "Calculation of slopes of $\delta^{18}O_p$ and OLR values" section (L23–L64) in the revised Supplementary information.

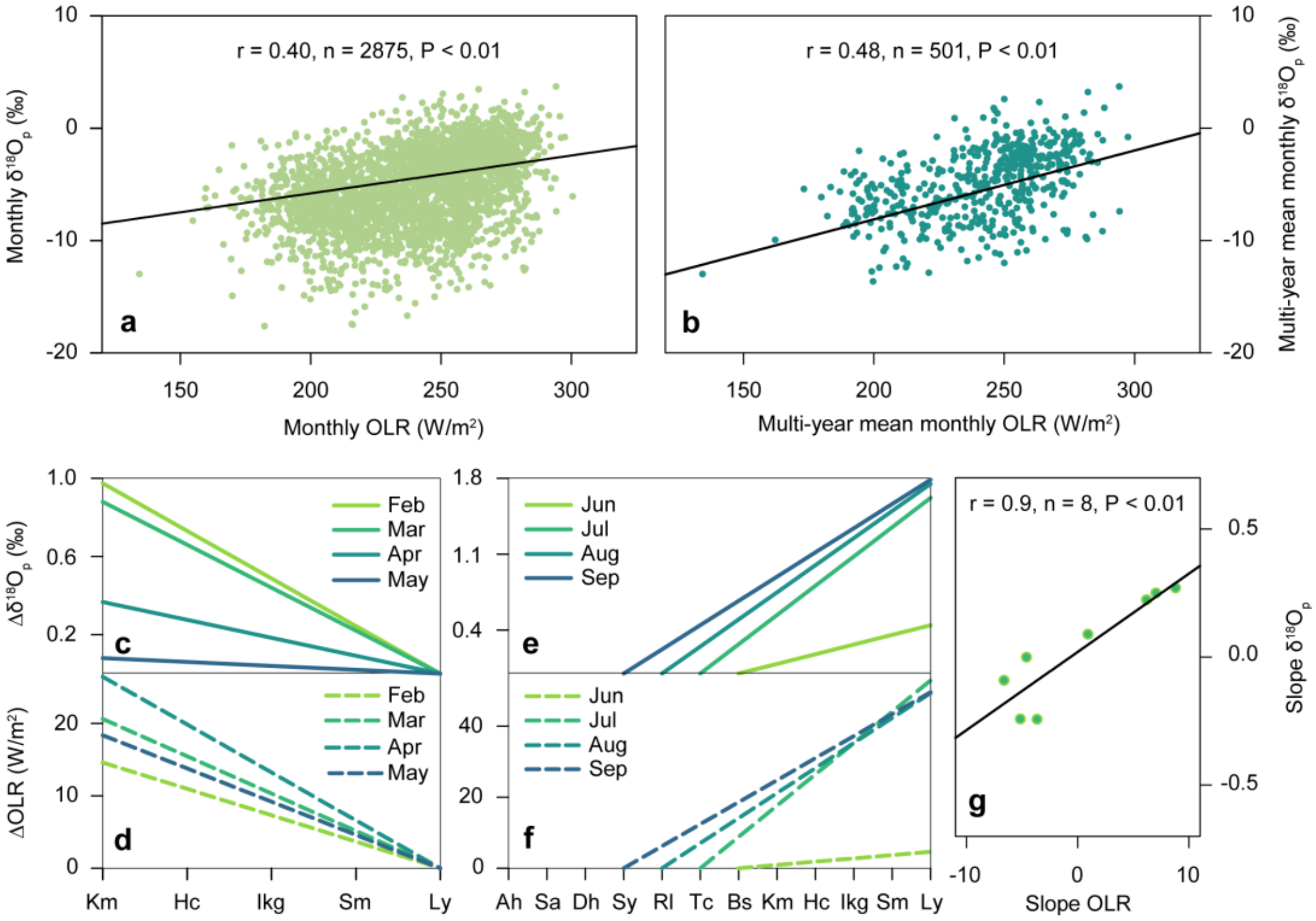

Figure R42. Correlations between $\delta^{18}O_p$ and outgoing longwave radiation (OLR) along the transect. a–b Linear regressions of $\delta^{18}O_p$ at the 46 sites across the study region against corresponding OLR on different timescales. c–d Gradients of $\delta^{18}O_p$ and OLR from Luoyuan to Kunming during the pre-monsoon season. e–f Gradients of $\delta^{18}O_p$ and OLR from Luoyuan to Baoshan, Tengchong, Ruili, and Sylhet during the monsoon season. Values on the y-axes in c–f were calculated using the "base value" method (Materials and Methods). g Correlation between the gradients of $\delta^{18}O_p$ and OLR during the pre-monsoon and monsoon seasons. $\delta^{18}O_p$ values are reported as per mil (‰) deviations from VSMOW or VSMOW2. Abbreviations: Ah (Ahmedabad), Sa (Sagar), Dh (Dhanbad), Sy (Sylhet), Rl (Ruili), Tc (Tengchong), Bs (Baoshan), Km (Kunming), Hc (Hechi), Ikg (Institute of Karst Geology), Sm (Sanming), and Ly (Luoyuan).

Fig. 6: perhaps 6b would be clearer if the colour the boxes with the corresponding colours of the lines in 6a? Can you also explain in the caption how the delineations in 6a are defined?

Re: We thank you for pointing these issues out. Following your suggestion, we changed the colours of the lines in Fig. 6a to the corresponding colours in Fig. 6b (Figure R43 as follows). The updated figure (Fig. 7) can be found in the revised

manuscript.

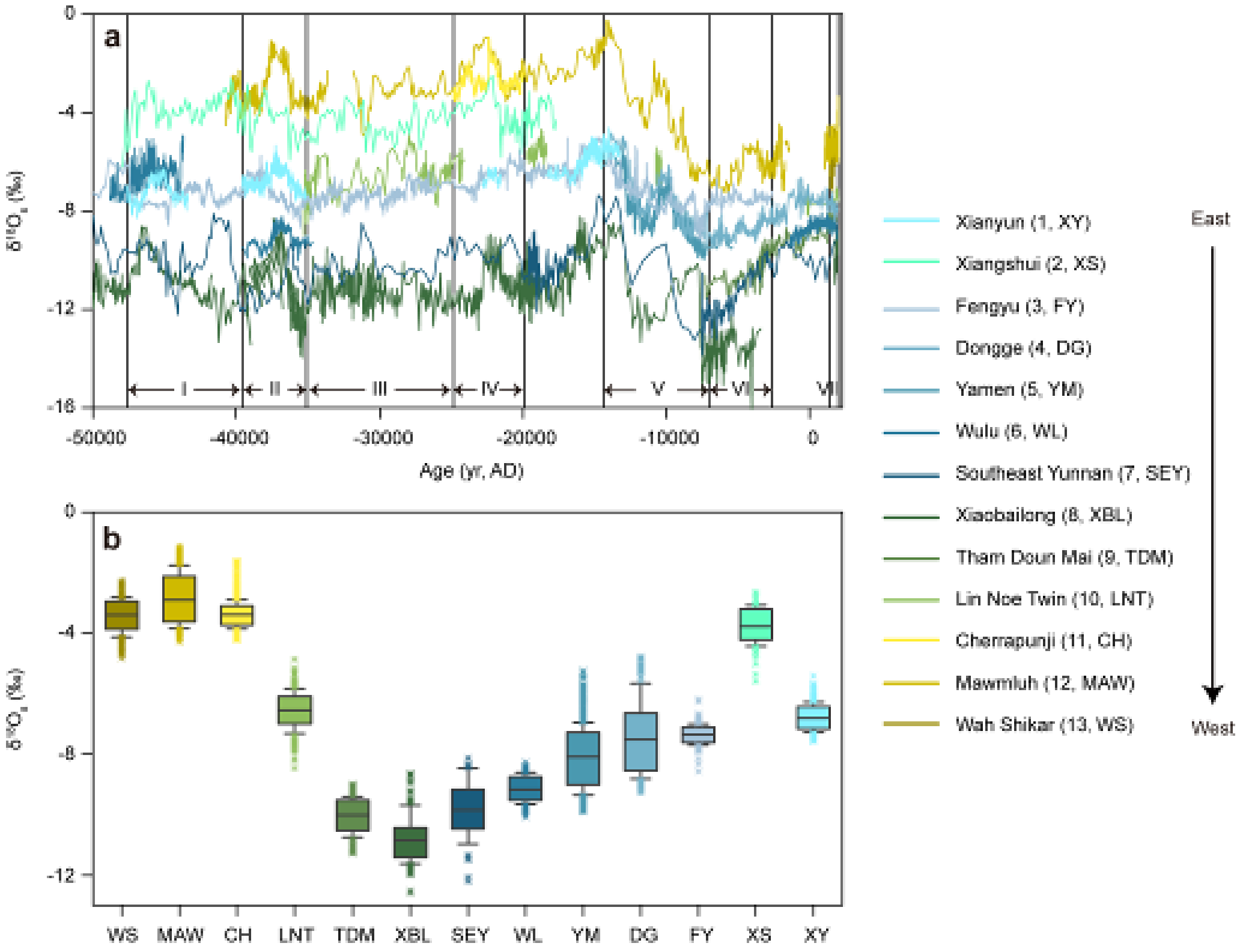

Figure R43. Comparisons of the $\delta^{18}O_s$ records from 13 caves along the transect during the last 50,000 years. a $\delta^{18}O_s$ records from the 13 caves along the transect. Vertical lines indicate the dividing line of each sub-period. b Spatial distribution of multi-year mean $\delta^{18}O_s$ values from the 13 caves, calculated using a "relative value" method (see Materials and Methods). In each box plot, the box represents the 20th–80th percentile range, whiskers extend to the 10th–90th percentiles, horizontal lines inside the boxes indicate the mean, and stars represent outliers. Numbers in brackets correspond to the open circles in Fig. 1a. $\delta^{18}O_s$ values are reported as per mil (‰) deviations from Vienna Peedee belemnite (VPDB).

With regard to the delineations in Fig. 6a (new Fig. 7a), following your suggestion, we added explanations in the Materials and Methods how the delineations in Fig. 6a (new Fig. 7a) are defined. i.e., "*To calculate the "relative" $\delta^{18}O_s$ values as accurately as possible, we divided the $\delta^{18}O_s$ records into seven sub-periods (Periods I –VII) (Fig. 7a) following two criteria: (1) we ensured that as many $\delta^{18}O_s$ records as possible were available for each sub-period; and (2) all our grouped sub-periods were designed to cover the entire period (the past 50,000 years) as comprehensively as possible.*"

Please see L778–L782 in the revised manuscript.

Comments on Supplement

Fig. S1: Are these correlations or regressions? If they are correlations, the R should be written as an italicised "r". It is also necessary to know if this is a spearman's or pearson's correlation and if the data fulfils the criteria for normal distribution. If they are regressions, the R value should be written as "R2" and the equation should be given on the chart.

Re: We thank you for pointing these issues out. These are correlations. Following your suggestions, we changed "R" to "r", and explained that this is a pearson's correlation (as follows). The updated Fig. S1 can be seen in the revised Supplementary Information.

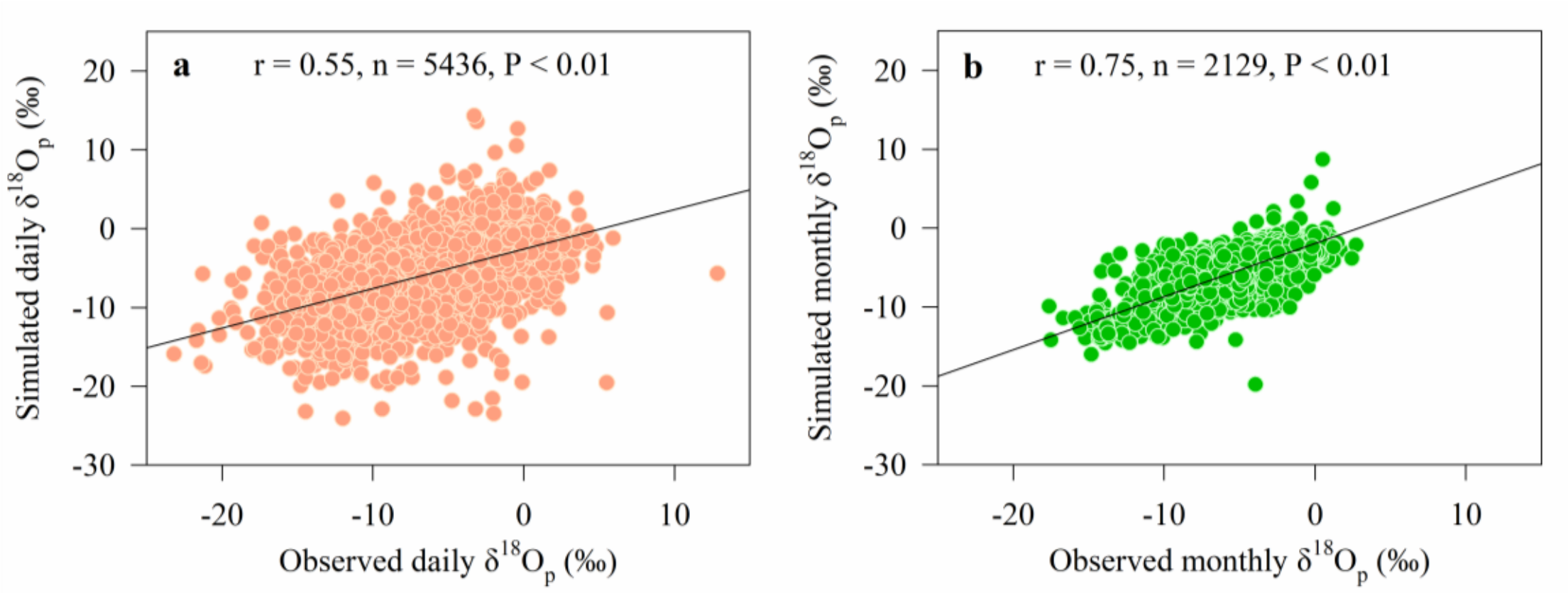

Figure R44. Correlations between observed $\delta^{18}O_p$ and IsoGSM2 simulated $\delta^{18}O_p$ at the 46 precipitation sites across the Asian monsoon region. a Daily sampling sites. b Monthly sampling sites. For both sampling intervals, the Pearson correlation (r) is significant at the 0.01 level (2-tailed). $\delta^{18}O$ in precipitation ($\delta^{18}O_p$) values are expressed as per mil (‰) deviations relative to VSMOW or VSMOW2.

In addition, we used SPSS 14.0 to analyze the data, and found that both the observed $\delta^{18}O_p$ and simulated $\delta^{18}O_p$ data follow a normal distribution (as follows).

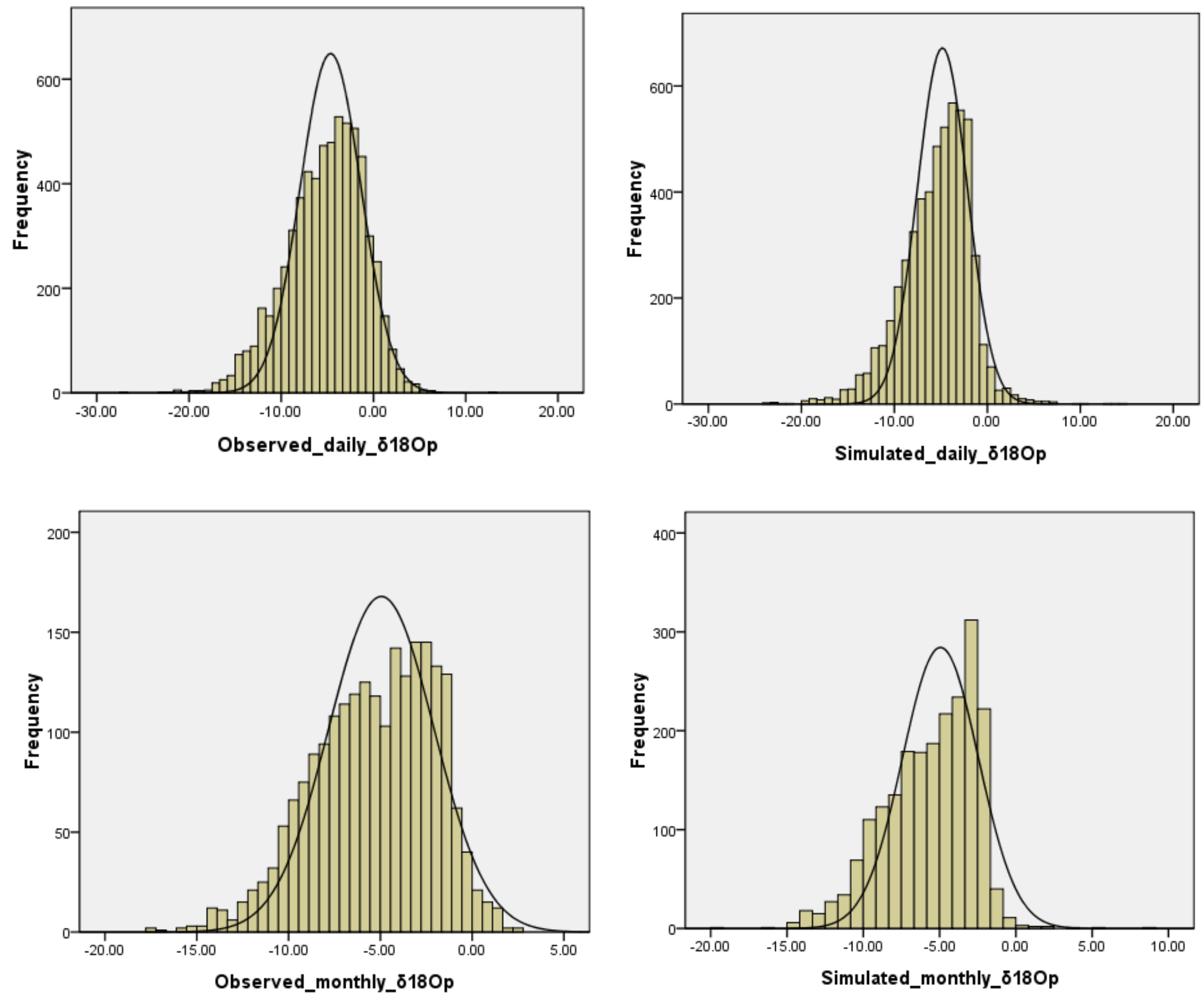

Figure R45. The histogram illustrates the distributions of the datasets including observed $\delta^{18}O_p$ and simulated $\delta^{18}O_p$ data.

Fig. S1. My concern with either a regression or correlation from this data is that the data does not look particularly linearly distributed. I wonder if the authors can show if the data is normally distributed or not? If not, this will help you to select the correct correlation/regression test for the data (as well as data transformation).

Re: We thank you for pointing this issue out. Following your comment, we used SPSS 14.0 to analyze the data, and found that both the observed $\delta^{18}O_p$ and simulated $\delta^{18}O_p$ data are normally distributed (as follows).

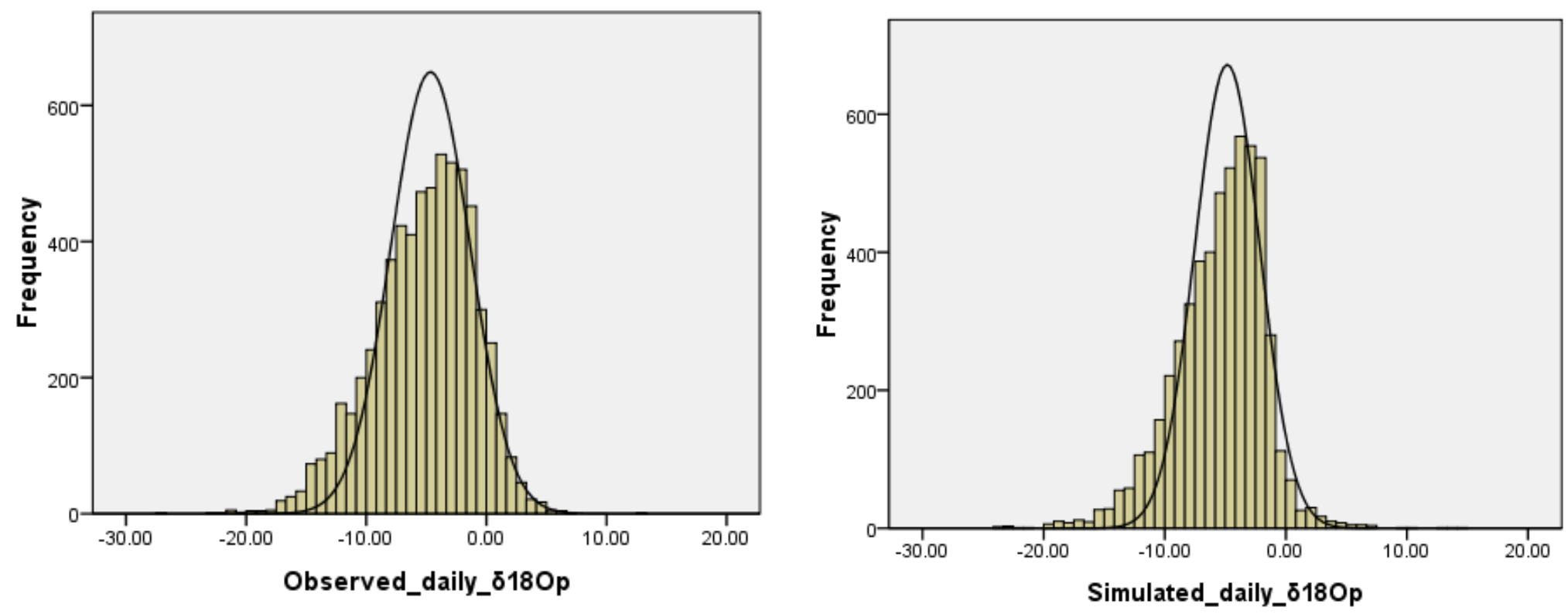

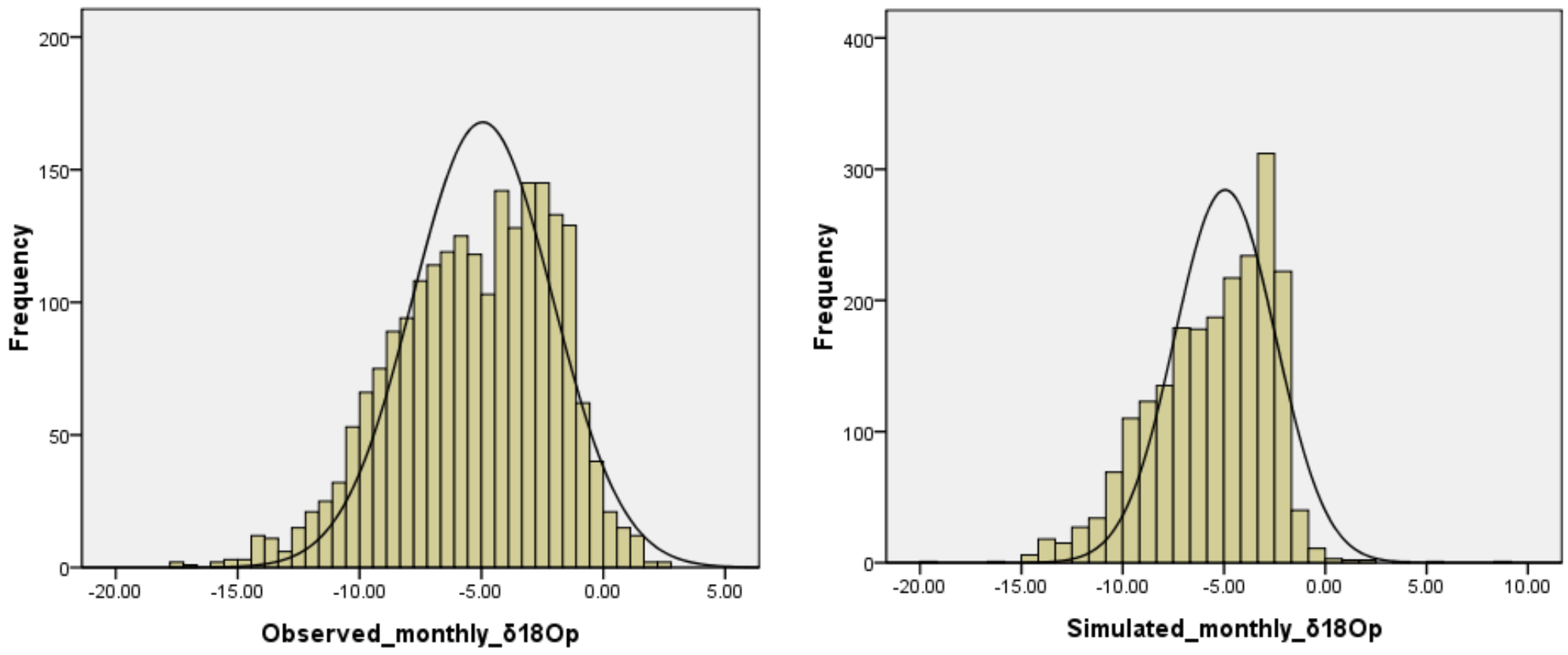

Figure R46. The histogram illustrates the distributions of the datasets including observed $\delta^{18}O_p$ and simulated $\delta^{18}O_p$ data.

Fig. S4: These are not correlations; they are regressions (since they provide an output equation to predict the data). Why are they separated into 3 boxes, instead of simply regressing altitude and $\delta^{18}O$ for all the sites? I think this needs clearer justification. Furthermore, if these are regressions, the R value is likely an R^2 rather than a correlation coefficient (r).

Re: We thank you for pointing this issue out. We agree with you that these are not correlations, but they are regressions. Following your comment, we added the slopes of the output equations, and changed correlation coefficient (r) to R^2. Following your suggestion, we regressed altitude and $\delta^{18}O$ for all the sites over the entire year (as follows). The updated Fig. S12 can be seen in the revised Supplementary Information.

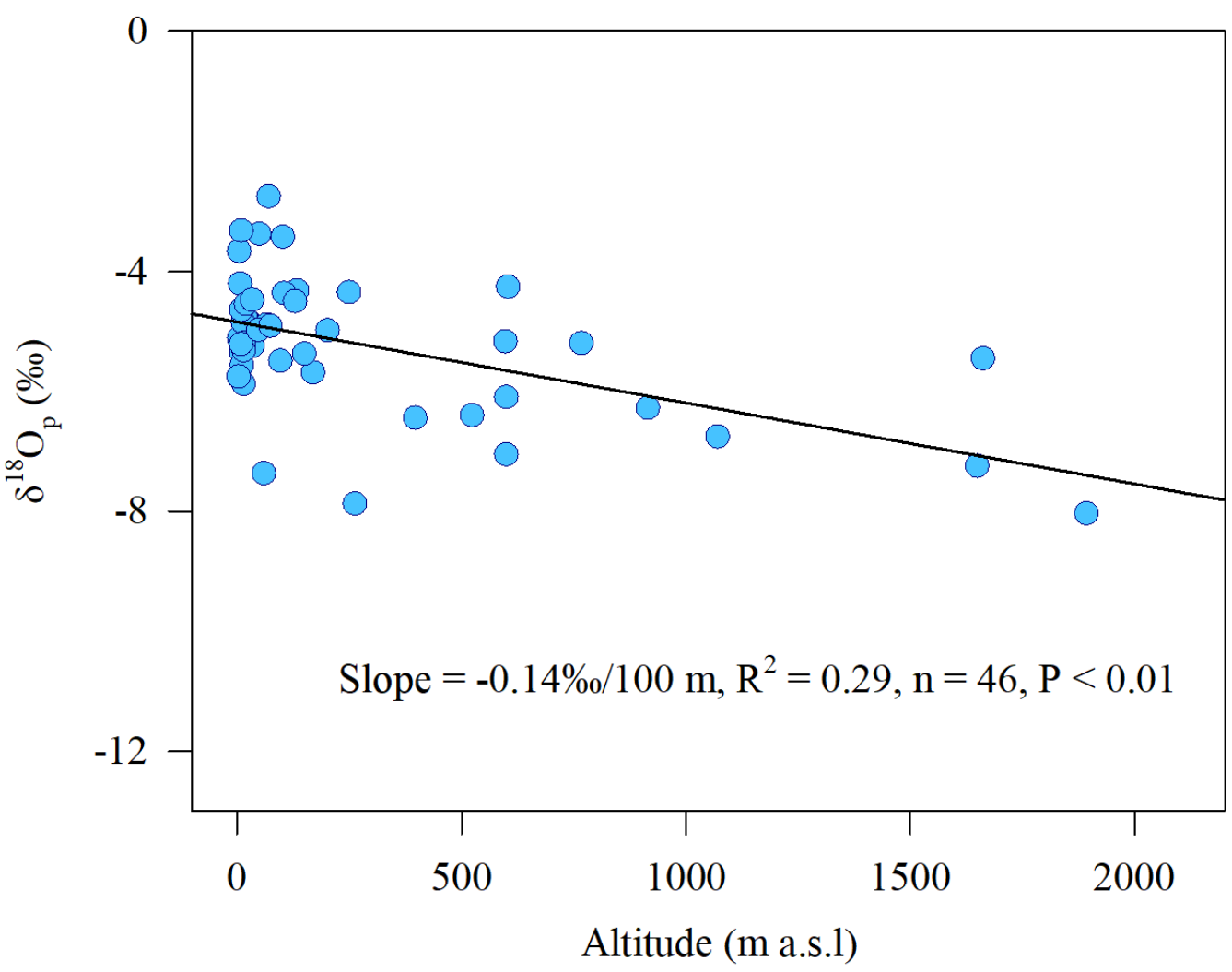

Figure R47. Influence of altitude on observed $\delta^{18}O_p$ at the 46 sites across the study region from 1979 to 2022. $\delta^{18}O_p$ values are expressed as per mil (‰) deviations

relative to VSMOW or VSMOW2.

Moreover, in this study, in addition to adding the discussion of the relationship between observed $\delta^{18}O_P$ values and the corresponding altitudes based on the Köppen-Geiger climate classification, we also retained the discussion of the relationship between observed $\delta^{18}O_P$ values and the corresponding altitudes based on the UNEP-WCMC mountain classification (as follows). Please the updated Fig. S14 in the revised Supplementary Information.

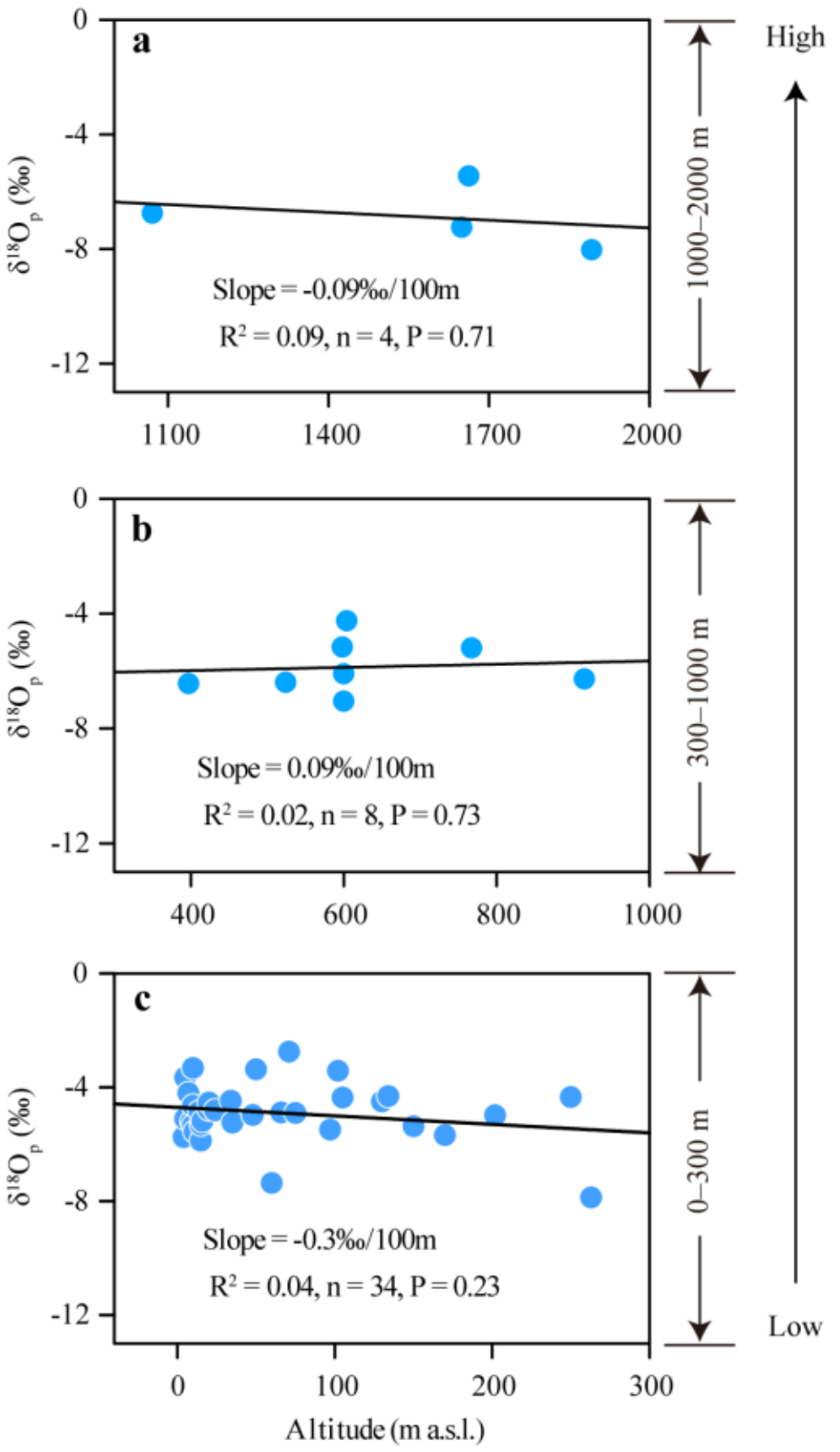

Figure R48. Correlations between observed $\delta^{18}O_P$ values and altitudes. The 46 precipitation sites were categorized into three altitude ranges based on the UNEP-WCMC mountain classification[2]. a–c, Correlations for the altitude ranges 1000–2000 m (a), 300–1000 m (b), and 0–300 m (c). $\delta^{18}O_P$ values are expressed as per mil (‰) deviations relative to VSMOW or VSMOW2.

Fig. S8, Fig. S9, Fig. S10: The different abscissa scales between the figures create an intensified visual effect, suggesting the spatial difference in $\delta^{18}O$ is much stronger than it is. Moreover, the abrupt change from blue to yellow colour strengthens that

effect. I suggest using the same scale for all three figures and adapting the colour scheme to realistically portray the spatial distributions of simulated $\delta^{18}O_p$.

Re: We thank you for pointing this issue out. The Reviewers #1 and #2 commented that the Supplementary Information included an extremely large number of figures and that many of these figures did not add anything to the story. Following their comments, we carefully evaluated each figure in the Supplementary Information and found that many of them indeed did not add anything to the story. For example, regarding spatial distributions of simulated $\delta^{18}O_p$ across the study region over 1979–2022 during the pre-monsoon and monsoon seasons (Figs. S8–S10), they are of little significance because we have already confirmed that there is a good correlation between the simulated $\delta^{18}O_p$ data and the measured $\delta^{18}O_p$ data. That is, the simulated $\delta^{18}O_p$ data can capture the measured $\delta^{18}O_p$ data very well. Moreover, we have already demonstrated the westward expansion of the low-value zone of precipitation stable isotopes along the transect by using the simulated $\delta^{18}O_p$ data at the 12 primary stations. It is obviously of little significance to further demonstrate the spatial changes with the simulated $\delta^{18}O_p$ data. Therefore, we removed Figs. S8–S10 from the Supplementary Information.

Fig. S11. Is there a source for this information?

Re: This figure is plotted by the ERA5 reanalysis data including pecific humidity, meridional wind, and zonal wind. Following your comment, we added some sentences to make clear the source, i.e., "*The study utilized the ERA5 reanalysis data (0.25°×0.25° resolution) provided by the European Centre for Medium-Range Weather Forecasts (ECMWF). The ERA5 reanalysis data on single levels are available from https://cds.climate.copernicus.eu/cdsapp#!/dataset/reanalysis-era5-single-levels-monthly-means?tab=form, following ref.* ***Error! Reference source not found.****. The ERA5 reanalysis data on pressure levels are available from https://cds.climate.copernicus.eu/cdsapp#!/dataset/reanalysis-era5-pressure-levels-monthly-means?tab=form, following ref.* ***Error! Reference source not found.****. Among them, ….. We used specific humidity combined with meridional and zonal winds to calculate the moisture flux.*" Please see L738–L750 in the revised manuscript.

Fig. S15. The x-axis has been labelled as °N. I think this should be °E?

Re: Thank you very much for pointing this issue out. We are very sorry for the mistake caused by our negligence. In addition, we found that there is a similar mistake for Fig. S16. We revised the figures as follows. The updated figures (Fig. S10 and Fig. S11) can be seen in the revised Supplementary Information.

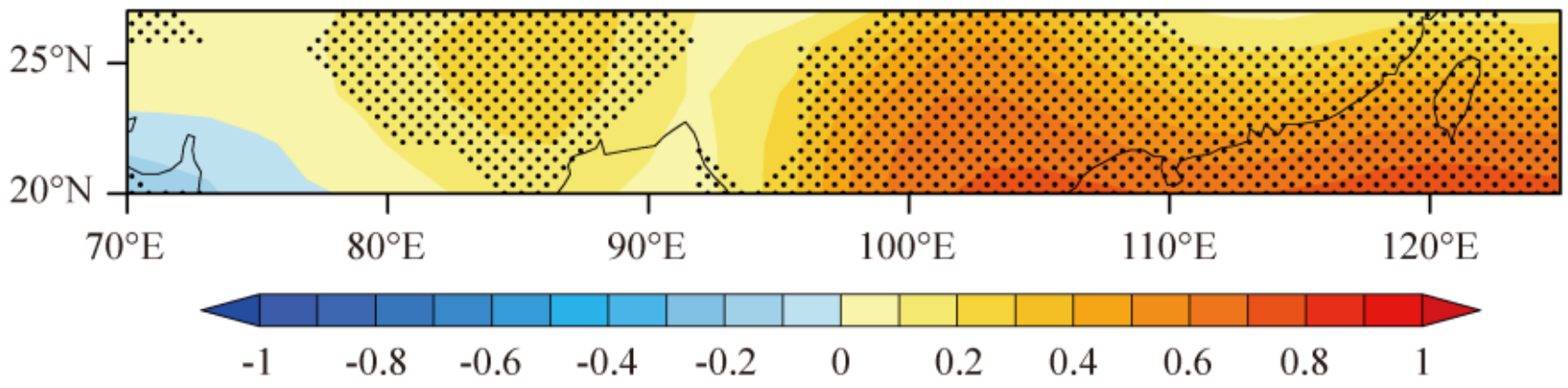

Figure R49. Spatial correlations between monthly IsoGSM2-simulated $\delta^{18}O_p$ and the corresponding outgoing longwave radiation (OLR) from 1979 to 2022.

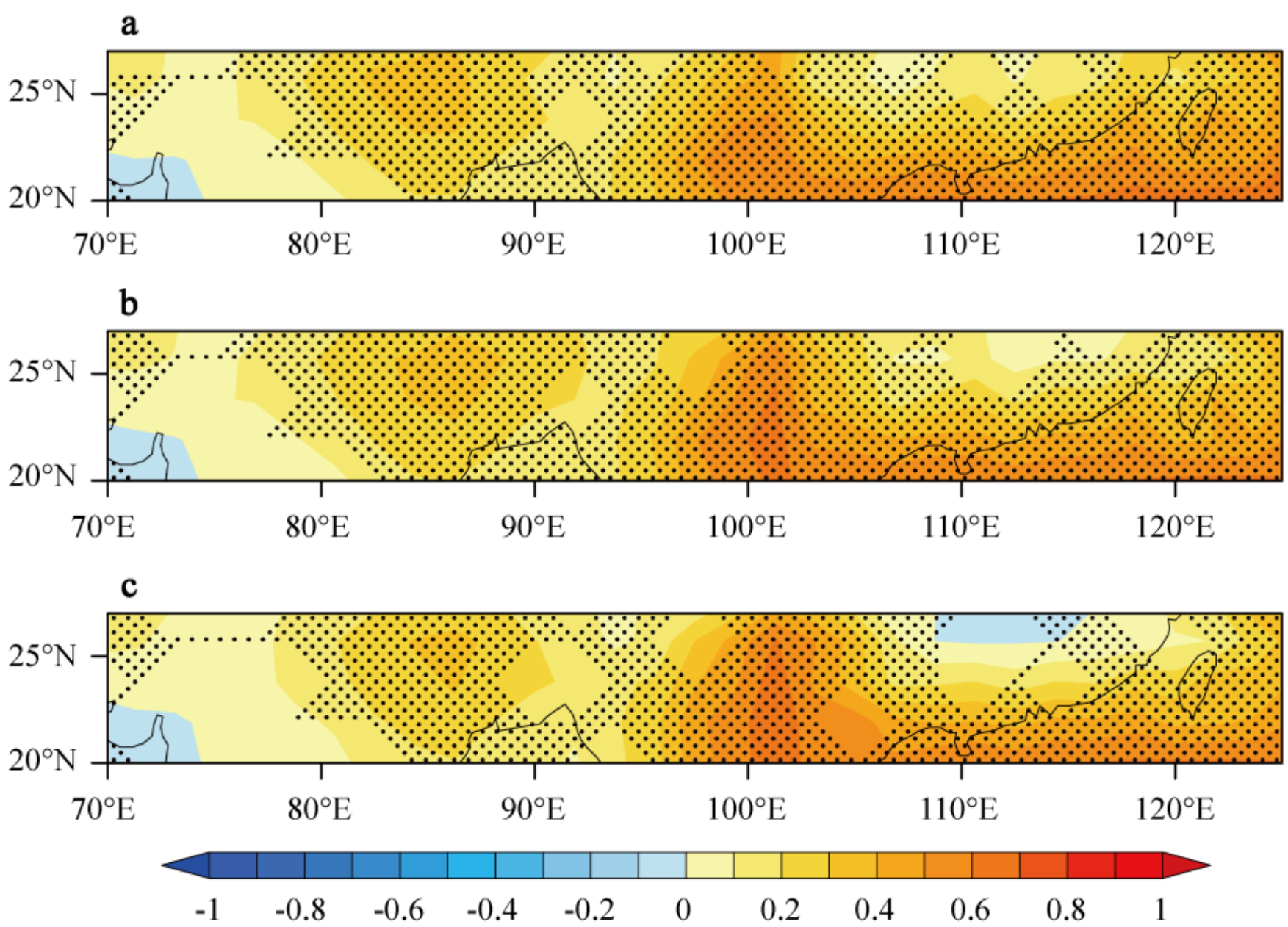

Figure R50. Spatial correlations between monthly IsoGSM2-simulated $\delta^{18}O_p$ and the corresponding vertical velocity from 1979 to 2022. a–c, Vertical velocity at 300 hPa, 400 hPa, and 500 hPa, respectively.

Fig. S21. Please provide the number of measurements for each box.

Re: Following your suggestion, we provided the number of measurements for each box in the bracket (as follows). The updated Fig. S19 can be seen in the revised Supplementary Information.

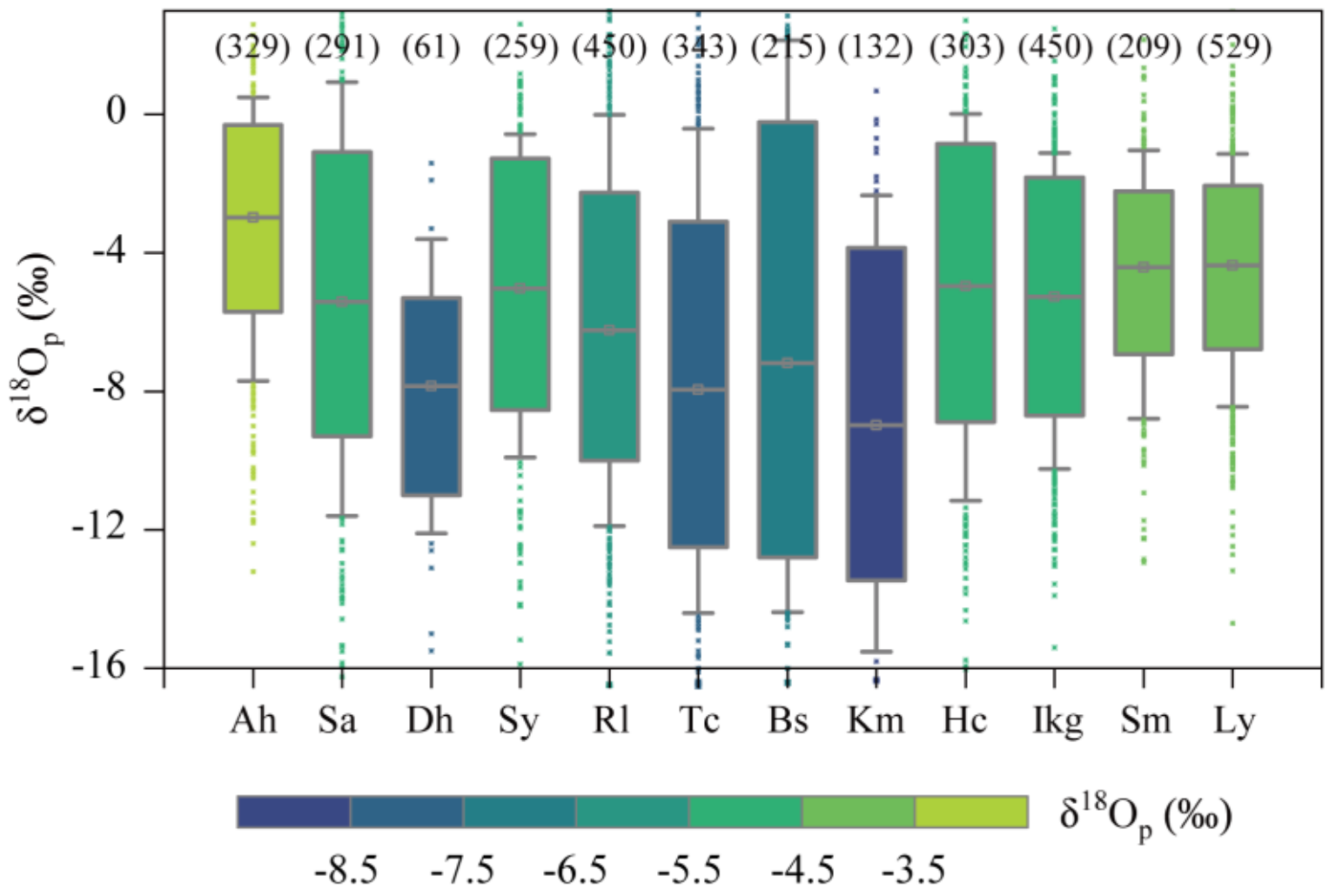

Figure R51. Spatial distributions of observed annual $\delta^{18}O_p$ at the 12 primary sites along the east–west transect. In each box plot, the box represents the 20th–80th percentile range, whiskers extend to the 10th–90th percentile, horizontal lines inside the boxes indicate the mean, and dots represent outliers. The number of measurements for each box was provided in the brackets. $\delta^{18}O_p$ values are expressed as per mil (‰) deviations relative to VSMOW or VSMOW2.

Fig. S22. Please can you plot the location of the caves on the map?

Re: Following your suggestion, we plotted the locations of the caves on the map (as follows). The updated Fig. S21 can be seen in the revised Supplementary Information.

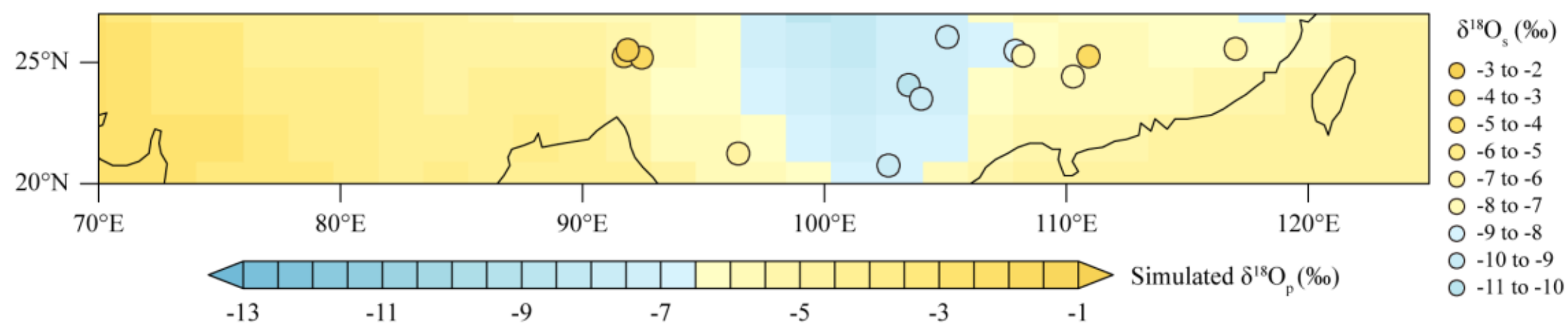

Figure R52. Spatial distribution of IsoGSM2-simulated annual $\delta^{18}O_p$ across the study region over 1979–2022. Multi-year mean $\delta^{18}O_s$ values from 13 caves (dot) across the study region are also shown. $\delta^{18}O_p$ values are expressed as per mil (‰) deviations relative to VSMOW/VSMOW2. $\delta^{18}O_s$ values are reported as per mil (‰) deviations from Vienna Peedee belemnite (VPDB).

Supplementary excel files:

The file is not self-explanatory and would benefit from a small informational text and

units in the column headers. The STDEV seems very large for the reported values (i.e., -11.37‰ with a STDEV of 10.00). Only two of the sites have been included (Baoshan and Hechi). The authors must include all the sites analysed. Moreover, they should include the additional metrics they are analysing, and include precipitation amount and temperature at the sites for each of the months, so that other researchers can reproduce their finding

Re: We thank you for pointing these issues out. Following your suggestions, we added a small informational text and units in the column headers for each sheet. Following your suggestions and the editorial requests, we provided the data for each figure/table in a separate sheet (including all the figures and tables contained in the manuscript and the Supplementary Information), so that other researchers can reproduce our finding. Please see Source Data.zip file.

With regard to the comment of "The STDEV seems very large for the reported values", we carefully examined the $\delta^{18}O_p$ data from the Baoshan site, and found an exceptionally low $\delta^{18}O_p$ value of -21.39‰ appeared on January 3, 2018, which may be associated with an extreme weather event. This result led to the STDEV seems very large for the reported values. Furthermore, we noted that such low $\delta^{18}O_p$ value primarily occurred during the post-monsoon season. However, we did not discuss the results during the post-monsoon season in detail in the paper (the paper mainly focused on the pre-monsoon and monsoon seasons, as the post-monsoon season contributes minimally to annual precipitation and is therefore not considered further). Hence, the "large" STDEV value does not affected our conclusion.

With regard to the comment of "include precipitation amount and temperature at the sites for each of the months", we provided both the multi-year mean monthly precipitation data and monthly precipitation data for all 12 primary sites in the Source Data Figure S3.xls file. It should be noted that these sites are located in the Asian monsoon region where $\delta^{18}O_p$ studies primarily focus on precipitation effects. Consequently, temperature data were not systematically collected for most sites. Furthermore, as mentioned above, this study intentionally does not examine the relationship between $\delta^{18}O_p$ and temperature. Therefore, we not included temperature data for these 12 sites.

Reviewer #4 (Remarks to the Author):

I co-reviewed this manuscript with one of the reviewers who provided the listed reports. This is part of the Nature Communications initiative to facilitate training in peer review and to provide appropriate recognition for Early Career Researchers who co-review manuscripts.

Re: Thank you very much for providing very fair, positive, and encouraging comments. Your comments and suggestions greatly improve the quality of our manuscript. We have tried to address all the concerns raised by you and have carefully revised the manuscript. Please see our response as mentioned above.

Response to Reviewers

(Reviewers' statements in black, and our response in blue)

Line163, -the line 163 in the previous manuscript.

L4-L5, -the lines 4-5 in the revised manuscript.

Fig. 1, -the Figure 1 in the revised manuscript.

Figure R1, -the Figure 1 in the response.

REVIEWER COMMENTS

Reviewer #1 (Remarks to the Author):

In this manuscript, Zhang et al. have made an extensive set of revisions in response to first review. Overall, we can see improvements in the structure and narrative of the manuscript, and the addition/revision of several key figures contributes to a better understanding of the processes and phenomena that the authors intend to resolve. Moreover, we are extremely grateful to the authors for taking so much time to consider, and respond to the first round of comments.

Re: We sincerely thank you for your positive feedback regarding our efforts in the first revisions. We are also very grateful for your constructive comments and suggestions, which have significantly improved the quality of our manuscript. We have tried to address all the concerns raised by you and have carefully revised the manuscript as follows.

However, we are still struggling with the flow of logic in the manuscript. A common feature of all articles that do well in NCOMMS is that they are clear, and focused in what is used, shown, and described: all in service of addressing a core question. This study is extremely data-rich, to the point where it feels as though the reader is being overloaded with things to think about, and the core question is lost. For example, a lot of new tests and analyses are introduced almost continuously, but in a way that is

often non-sensical. We found ourselves asking repeatedly: "what is this adding to the overall story?", "why is this needed?", and "what does it tell us?". Furthermore, the authors provide a huge volume of information in their rebuttal letter, but following several reads, a lot of it seems superfluous to requirement and not directly relevant.

Re: We sincerely thank you for your constructive comments regarding the logical coherence and information overload in the manuscript. Following your comments, we revisited the manuscript and conducted a careful analysis of the logical connections among its sections and paragraphs, confirming that there are indeed shortcomings in its logical coherence. We fully agree that for a high-impact journal such as "*Nature Communications*", a clear and focused narrative is crucial. Consequently, we extensively revised the manuscript as follows:

1. We made significant adjustments to the manuscript's structure, reorganizing certain sections and removing redundant or repetitive content to highlight the research focus, namely "to explore the mechanisms governing $\delta^{18}O_p$ variations across the Asian monsoon region." All these efforts are aimed at ensuring the content is concise, logically clear, and that all data, analyses, and figures serve to advance this central theme. The specific details are as follows:

1) Regarding the discussion on moisture sources influencing $\delta^{18}O_p$ in the Introduction section: In the previous revision, we included an analysis in the Introduction section on whether $\delta^{18}O_p$ in the Asian monsoon region reflects moisture sources, i.e., "If this were correct, $\delta^{18}O_s$ records from upstream regions along the South Asian monsoon moisture pathway should show higher $\delta^{18}O$ values than those from downstream locations. However, available evidence conflicts with this assumption. For example, the $\delta^{18}O_s$ record from Tham Doun Mai cave (Luang Prabang Province, Northern Laos) has a mean value of -9.84‰[51], while Dongge cave (Guizhou Province, southwestern China), located more than 4° to the north, has a higher mean value of -8.92‰[52] over the period 8874–7581 yr BP. Similarly, annual $\delta^{18}O_p$ values from Son La site near Tham Doun Mai cave (2017–2020) show a mean of -7.15‰ (Global Network of Isotopes in Precipitation, GNIP), which is lower than Huanjiang (-6.58‰)[53] near

Dongge cave.”

Given that its overly detailed content risks diluting the main theme, placing such analysis in the Introduction section is indeed inappropriate. Moreover, this could easily lead readers to get lost in specific examples (such as cave names and numerical comparisons), thereby overlooking the overarching scientific question that the article aims to explore. Therefore, we removed this paragraph. Please see L100 in the revised manuscript.

2) Regarding the IsoGSM2 model simulation: In the previous revision, we found that frequently interspersing IsoGSM2 model data to validate the measured results severely disrupted the narrative coherence. Therefore, we removed all content related to the IsoGSM2 model simulation. Please see L107, L125, L149, L277, and L616 in the revised manuscript.

3) Regarding the “$\delta^{18}O_p$ and the advection of monsoon moisture” section: During the revision process, we realized that inserting a lengthy section titled “$\delta^{18}O_p$ and the advection of monsoon moisture” before the core section “$\delta^{18}O_p$ and atmospheric updraft” disrupted the flow of the article and diluted its focus. This prevented the central theme (“the impact of atmospheric updraft on $\delta^{18}O_p$ across the Asian monsoon region”) from being highlighted promptly. As a result, we removed this section. The content of this section has been addressed as follows:

First, we directly removed some of the content.

Second, we moved part of the content to the section “Seasonal variations and spatial distributions of $\delta^{18}O_p$” and reorganized the section accordingly. Please see L125–L128, L129–L133, L149–L151, and L158–L166 in the revised manuscript.

Finally, we moved some of the content to the Supplementary Information, where it has been divided into three sections, including Text S1. Seasonal decoupling between $\delta^{18}O_p$ and monsoon intensity; Text S2. Spatial decoupling between $\delta^{18}O_p$ and isotopic depletion during the advection; and Text S3. Correlations between $\delta^{18}O_p$ and monsoon

indices. Please see L23–L64 in the revised Supplementary Information.

4) Regarding the paleoclimate records ($\delta^{18}O_s$ records): As you and Reviewer #5 pointed out, incorporating this part into the present study does create a logical leap and would be more appropriately addressed in a separate paper. Following your suggestions, we removed all content related to $\delta^{18}O_s$ records (including related analyses, figures, data sources, and methods) to ensure our manuscript remains focused on the study of stable isotopes in modern precipitation. Please see L322 in the revised manuscript.

Accordingly, we changed the manuscript title from "Reinterpreting Asian speleothem isotope records: the hidden role of atmospheric updraft" to "*Reinterpreting precipitation isotope records from the Asian monsoon region: the hidden role of atmospheric updraft*". Please see L1–L2 in the revised manuscript.

5) Regarding the discussion of topographic effects on precipitation: In the previous revision, when discussing the impact of topography on precipitation isotopes, we abruptly inserted a discussion on topographic effects on precipitation amount (Lines 321-326), which deviated significantly from the main theme of our study. Therefore, we removed the discussion on topographic influences on precipitation (please see the detailed explanation below). Please see L255 in the revised manuscript.

2. We changed the writing style of the manuscript to ensure that each discussion point directly serves the core narrative. Instead of continuously introducing new tests and analyses, we now use clear "signposts" to guide readers through the flow of the discussion. Specifically, we added clear transitional sentences at the beginning and end of each paragraph and section. These transitions explicitly state how the findings from the previous discussion lead into the next part of the narrative and explain what the new results mean for the core research question. The specific revisions are as follows:

1) In the Introduction section, after reviewing previous research and existing controversies, we introduced the key scientific question with a query at the end of the third paragraph: "*These contradictions lead to a central question: what are the principal drivers controlling* $\delta^{18}O_p$ *variations across the Asian monsoon region?*" The fourth paragraph then begins with "*To address this question,*" directly echoing the previous context and ensuring narrative coherence. Please see L100–L103 in the revised manuscript.

2) In the "Seasonal variations and spatial distributions of $\delta^{18}O_p$" section, we added a guiding sentence before analyzing the seasonal variations of $\delta^{18}O_p$, i.e., "*To investigate seasonal* $\delta^{18}O_p$ *variations between the East Asian and South Asian monsoon regions, we established an east–west transect (20–27° N, 70–125° E) with ~98° E as the dividing line*[55,56]". Please see L115–L116 in the revised manuscript.

3) In the "Seasonal variations and spatial distributions of $\delta^{18}O_p$" section, we added several guiding sentences before analyzing the spatial variations of $\delta^{18}O_p$, i.e., "*Previous studies have often attributed* $\delta^{18}O_p$ *variations in the South and East Asian summer monsoon regions to a "rainout effect" occurring along long-distance monsoon moisture advection from the Indian Ocean*[50-53] *(occurring within the advection; hereafter denoted as the "rainout effect*$_{ad}$*"). This mechanism predicts a "west-high, east-low"* $\delta^{18}O_p$ *pattern along the transect. To test this expectation, we analyzed the spatial distribution of multi-year mean monthly* $\delta^{18}O_p$ *values along the transect from east to west for each month (Fig. 2 and Supplementary Fig. 2)……*" Please see L129–L135 in the revised manuscript.

4) After analyzing the spatiotemporal decoupling between $\delta^{18}O_p$ and monsoon moisture advection in the Asian monsoon region, we added transitional sentences to introduce the shift in research perspective: moving from a horizontal to a vertical perspective, i.e., "*Since explanation relying solely on the monsoon moisture advection has proven insufficient, a shift in research focus is necessary. Specifically, the*

approach should move from the previous advection perspective to a vertical one that emphasizes atmospheric updraft (including frontal lifting and convection), which have previously been underappreciated." Please see L154–L158 in the revised manuscript.

5) At the beginning of the "Mechanisms of atmospheric updraft changes" section, we added transitional sentences to introduce the relevant discussion, i.e., "*Since variations in atmospheric updraft determine $\delta^{18}O_p$ patterns along this transect, what dynamics drive the westward migration of updraft? To investigate the underlying mechanism, we turn to the large-scale circulation systems*……" Please see L257–L259 in the revised manuscript.

3. Due to structural adjustments, we consequently removed multiple figures and tables. For example, following the removal of the "$\delta^{18}O_p$ and the advection of monsoon moisture" section, Supplementary Fig. S7 has been deleted. After removing the "Speleothem $\delta^{18}O$ records" section, Fig. 7 in the main text, along with Supplementary Figs. S19, S20, and S21, were removed. Additionally, with the removal of content related to the IsoGSM2 model, Supplementary Figs. S1, S3, S6, S10, and S11 were deleted. Furthermore, we merged some figures: Supplementary Fig. S15 has been combined with Fig. 2 in the main text and Supplementary Figs. S12 and S13 were merged.

As a result of these revisions, the number of figures in the main text has been reduced from 7 to 6, while the number of figures in the Supplementary Information has decreased from 21 to 10, and the number of tables in the Supplementary Information has been reduced from 5 to 4. These streamlining modifications have significantly reduced the total number of figures and tables and have helped avoid the negative impact of frequent cross-references on the manuscript's readability.

4. We removed some unnecessary and abrupt terminological concepts, such as the "altitude effect," "inverse altitude effect," and "false altitude effect." Clearly, these

terms or concepts are not the focus of this study.

In short, throughout this round of revisions and responses, we focused on conciseness and adherence to the main theme. We believe that with your detailed and professional comments and our substantive modifications, the manuscript's logic has become significantly clearer, and its key points have been effectively highlighted. Your valuable feedback has played a crucial role in enhancing our manuscript, and we are deeply grateful for it. We hope you will recognize the improved logical flow in the current version.

Some other important points:

- We fail to be convinced that the inclusion of paleo-records is valuable, or necessary. It almost felt like these records were included as an afterthought, and would be better presented in a separate paper entirely. Especially given that the authors note that they had merged datasets to create composite records in several cases. This is a procedure that is not simply 'layering' records on top of each other, and so needs far more description than is given in order to be robust, or justified.

Re: Thank you for your thoughtful review and for raising an important point about the inclusion of paleoclimate records in our manuscript. We have carefully reconsidered this issue and agree completely with your assessment. As you rightly pointed out, the section on paleoclimate records is indeed more suitable to be presented separately in another paper, and we also recognize that the methodology for creating composite records was insufficiently described to meet rigorous scientific standards. We appreciate your guidance in identifying these weaknesses. Accordingly, we have removed the entire section on speleothem $\delta^{18}O$ records from the manuscript, including all related analyses, figures, and discussions. The manuscript has been carefully revised to ensure logical flow and coherence are maintained without this material. We believe these changes have strengthened the paper's focus and clarity, and we thank you again for your constructive input. Please see L50, L53, L54, L61, L98, L106, L322, L616, L660, and L810 in the revised manuscript. Please see L243 and L260 in

the revised Supplementary Information.

- The frequent reference to figures and the large number of supplementary figures continue to impair the readability of the manuscript. We appreciate a large part of this is likely unavoidable given the considerable amount of data and analysis. However, reference to these materials can be streamlined substantially with statements such as: "Details of X and Y are provided in an additional supplementary file".

Re: Thank you for this helpful suggestion regarding figure references and manuscript readability. We fully agree that streamlining the citation of supplementary materials would improve the flow of the paper, and we have made substantial revisions accordingly.

We have significantly reduced the number of figures and tables in both the main text and the Supplementary Information to include only the most essential materials. More importantly, we have adopted the concise referencing style you suggested throughout the manuscript, using summary statements (such as "see Supplementary Text 1 for details", "see Supplementary Text 2 for details") to guide readers to extended information without disrupting the narrative (Please see L128 and L151–L152 in the revised manuscript).

We believe these changes have noticeably enhanced the readability of the manuscript, and we thank you again for your constructive guidance.

- The revised manuscript still exhibits some deficiencies in certain sections of the interpretation, and the structure of the arguments – all of which need to be addressed in order to make a convincing case that updraft is a critical mechanism, that could re-shape our understanding of speleothem-derived $\delta^{18}O$ records.

Re: Thank you for your constructive feedback. We fully agree that making a convincing case for atmospheric updraft as a critical mechanism for reinterpreting speleothem $\delta^{18}O$ records requires clarity and rigor throughout the manuscript.

In this revision, we have carefully reexamined each section where interpretations were previously lacking or where the argument structure weakened the core thesis. Specifically, we have strengthened the mechanistic link between updraft dynamics and $\delta^{18}O$ variability in the discussion section, clarified the underlying assumptions in our interpretive framework, and ensured that each paragraph builds logically toward the central argument. We have also streamlined peripheral content that may have distracted from the main narrative.

We are grateful for your guidance in helping us sharpen the argument and would welcome any further suggestions you may have.

Taken together, we believe that this study would be better suited to a longer-format journal, where each analytical component can be carefully laid out without the constraints of a tight word limit. In other words, we do not feel this is a NCOMMS-suitable article – given the volume of data, information, and detail needed in order to present a convincing case.

Re: Thank you for this important feedback regarding the manuscript's fit for NCOMMS. We fully appreciate your concern that the volume of data and analysis might exceed the typical scope of a NCOMMS article.

In response, we have taken a fundamentally different approach to this revision. Rather than attempting to condense all analyses into a compressed format, we have restructured the manuscript around a single, coherent narrative that foregrounds the conceptual advance (i.e., the atmospheric updraft hypothesis) while strategically moving extensive methodological details and supporting analyses to the Supplementary Information. The main text now focuses on presenting the core argument with the most critical evidence, with clear signposting to supplementary materials for readers seeking deeper technical insight. We believe this structure aligns well with NCOMMS's emphasis on concise, high-impact findings that are supported by rigorous but unobtrusive documentation.

We respectfully submit that this reframed version addresses the essence of your concern: it presents a convincing case for the conceptual breakthrough without requiring readers to navigate excessive detail in the main text. We would be grateful for your reassessment and remain open to further guidance on how the presentation might be refined.

DETAILED COMMENTS

Line 51: "across timescales" as an expression feels incomplete without some definition of what timescales are being referred to. We would suggest either deleting this phrase, or being more specific.

Re: Following your suggestion, we deleted "across timescales." Please see L51 in the revised manuscript.

Lines 52-53: State why the view is debated.

Re: Thank you for this helpful suggestion. We agree that signaling the nature of the debate adds important context for readers. To address your point while keeping the Abstract concise, we changed "Speleothem oxygen isotope records ($\delta^{18}O_s$) from the Asian monsoon region are crucial for reconstructing past climate variability across timescales. Traditionally, these records have been interpreted as indicators of monsoon intensity, but this view remains debated" to "*Speleothem oxygen isotope records ($\delta^{18}O_s$) from the Asian monsoon region provide valuable insights into past hydroclimate variability, yet their interpretation relies fundamentally on understanding the controls of modern precipitation isotopes ($\delta^{18}O_p$). Although numerous explanations for the spatiotemporal variability of $\delta^{18}O_p$ have been proposed, the primary factors across this region remain unresolved*." This phrasing implicitly acknowledges the existence of a debate without enumerating specific arguments, which are now discussed in detail in the Introduction (see L50–L54). We believe this approach strikes an appropriate balance between accessibility and precision.

Lines 53-55: Some rewording we think is needed here to more clearly state the purpose of the study. For example: "Drawing upon modern precipitation isotope records (46 sites), and speleothem data (13 caves) along a transect spanning the Asian monsoon region (eastern China to western India), here we present a novel way to interpret speleothem-derived $\delta^{18}O$ records." ...or words to that effect.

Re: Following your suggestion, we changed the sentence to "*Drawing upon $\delta^{18}O_p$ records from 46 sites along a transect spanning the Asian monsoon region (eastern China to western India), we propose a new hypothesis to explain $\delta^{18}O_p$ records from this region.*" Please see L54–L56 in the revised manuscript.

Lines 64-67: The last two sentences provide basically the same information. We encourage the authors to combine the two sentences into one and conclude the abstract with a clearer/stronger statement.

Re: Following your suggestion, we reshaped the last two sentences to "*Unlike previous views that emphasize the importance of monsoon moisture advection, we highlight that atmospheric updraft exerts the key control on $\delta^{18}O_p$ variations across the Asian monsoon region. Our findings provide a fresh perspective for the interpretation of climate signals preserved in Asian speleothem $\delta^{18}O$ records.*" Please see L63–L67 in the revised manuscript.

Line 66: Zonal? Not sure horizontal is the right word.

Re: We agree that "horizontal processes" was too vague. Following your suggestion, we changed "horizontal processes" to "*monsoon moisture advection*". Please see L61 in the revised manuscript.

Lines 73-74: Suggest re-wording, as this doesn't make full sense. For example: "Abrupt shifts in Asian monsoon intensity can have profound environmental, societal, and political impacts."

Re: Following your suggestion, we rewrote the sentence to "*Abrupt shifts in Asian monsoon intensity and precipitation patterns have profound implications for*

livelihoods and socioeconomic development across the monsoon region[3-5].” Please see L73–L75 in the revised manuscript.

Line 75: What length? Daily? Annual? Decadal? Or greater?

Re: It should include the decadal, centennial, and even orbital timescales. Following your suggestion, we changed the sentence to “*Hence, understanding climate variability in this region on decadal, centennial, and even orbital timescales is a major focus of scientific research*[6-9].” Please see L75–L76 in the revised manuscript.

Lines 75-76: More needed here. Why are they scarce?

Re: Thank you for this question. We agree that clarifying the reason adds valuable context, and we have now incorporated an explanation in the revised manuscript: “*as extensive instrumental observation networks were not established until the mid-20th century.*” Please see L77–L78 in the revised manuscript.

Lines 78-82: We have found that both arguments do not clearly explain why $\delta^{18}O$ has advantages over other proxies in this environmental setting in terms of elucidating the driving forces. High temporal resolution is also provided by the other proxies (trace elements, $\delta^{13}C$, etc.) and is therefore not a unique selling point or a comprehensible “key reason” (line 79) for the use of $\delta^{18}O$. This sentence is also misleading, as it sounds as though you are suggesting that $\delta^{18}O$ in ice cores and speleothems record the same thing (which we know they don't). Make it clear that you are referring to comparison of common patterns/features in these records - that are indicative of large-scale climate events. Also, what added value does the comparison of $\delta^{18}O$ records from monsoon regions with ice core $\delta^{18}O$ records from Antarctica or Greenland provide for elucidating the mechanisms of the Asian monsoon system?

Re: Thank you for your insightful comments. We agree that other indicators (such as trace elements, $\delta^{13}C$, etc.) can also provide high-resolution data. Additionally, we acknowledge that the comparison with ice core records is misleading. Following your suggestions, we have substantially revised this section. The claims regarding

high-resolution advantages and the comparison with ice core records have been removed entirely. Instead, we now introduce the rationale for using speleothem $\delta^{18}O$ by simply noting the region's abundant karst landforms and the resulting availability of speleothem records, which provide a valuable proxy for past climate reconstruction. That is, we rewrote these sentences to "*Fortunately, this region hosts abundant karst landforms rich in speleothems*[11]*. Thanks to this abundance, speleothem oxygen isotope ($\delta^{18}O_s$) records from the region provide a valuable proxy for reconstructing past climate*[12-19]." Please see L78–L81 in the revised manuscript.

Lines 83-85: "Monsoon" is used 4 times in this sentence...

Re: Following your suggestion, we changed the sentence to "*Previous studies have widely applied Asian $\delta^{18}O_s$ records as a proxy for monsoon precipitation and/or monsoon intensity*[7,8,20-25]." Please see L82–L83 in the revised manuscript.

Line 88: What about the Pleistocene? The speleothem data shown in this manuscript cover the last 50,000 years. Also is it a "contradiction", or an "observation"?

Re: The statement on the spatial consistency of speleothem $\delta^{18}O$ is also valid for the Pleistocene. Yes, it is not a "contradiction." Following your comments, we added a reference (Liu et al., 2014) and revised these sentences to "*What is particularly striking is the contrast between the pronounced heterogeneity of precipitation across the Asian monsoon region*[29-31] *and the remarkable spatial consistency of Asian $\delta^{18}O_s$ records from the Holocene*[30,32] *and into the Pleistocene*[9]*. These apparent divergences have prompted researchers to re-examine the climate signals preserved in Asian $\delta^{18}O_s$ records by analyzing modern precipitation $\delta^{18}O$ ($\delta^{18}O_p$) variability*[33-38]." Please see L86–L91 in the revised manuscript.

Reference:

9. Liu, Z. et al. Chinese cave records and the East Asia summer monsoon. *Quaternary Sci. Rev.* **83**, 115–128 (2014).

Lines 91-95: Long sentence – split

Re: Following your suggestion, we split and rewrote this sentence. That is, we changed this long sentence to "*Recently, several hypotheses have emerged to explain $\delta^{18}O_p$ variability*[39-42]*. These include the "inverse temperature effect", where $\delta^{18}O_p$ and surface temperature show consistent inverse correlations*[38]*, and the "stratiform fraction" theory, where $\delta^{18}O_p$ reflects rain type proportions*[43]." Please see L92–L95 in the revised manuscript.

Line 93: local oceans (plural)

Re: Thank you for pointing this out. We have revised the text to address your comment. The two sentences discussing the "circulation effect" and moisture sources have been merged into a single, more concise statement to eliminate redundancy and improve clarity. The revised sentence now reads: "*Many studies also proposed the "circulation effect," suggesting that $\delta^{18}O_p$ in the Asian monsoon region reflects moisture sources*[45-49]*, particularly South Asian monsoon-related moisture*[50-53]." This revision also removes the need for the plural "local oceans," as the term no longer appears in the text. Please see L96–L98 in the revised manuscript.

Lines 97-100: Explain why

Re: Thank you for this question. The explanation for the isotopic pattern is as follows: During moisture transport by the South Asian monsoon, as an air mass moves downstream (e.g., sequentially passing through Station A and then Station B), the heavier water isotopes (e.g., ^{18}O, ^{2}H) condense more readily at the onset of precipitation. When the moisture first reaches an upstream station like A, this isotopic fractionation results in precipitation that is relatively enriched in the heavier isotopes. Consequently, the residual moisture becomes progressively depleted in these heavier isotopes. As this depleted moisture continues toward a downstream station like B, precipitation (and the speleothem $\delta^{18}O$ recording it) in these downstream regions typically exhibits lower $\delta^{18}O$ values than at Station A.

We originally included a more detailed version of this explanation in the Introduction. However, given that its overly detailed content risks diluting the main theme, placing such analysis in the Introduction section is indeed inappropriate. Moreover, this could easily lead readers to get lost in specific examples (such as cave names and numerical comparisons), thereby overlooking the overarching scientific question that the article aims to explore. Therefore, following your previous comments about maintaining focus, we removed this paragraph. Please see L100 in the revised manuscript.

Line 104: It is either "…from the Son La site…" or "…from Son La near…". Check also line 106 "…lower than Huanjiang () near…".

Re: As stated above, we rewrote this paragraph, and removed these statements. Please see L100 in the revised manuscript.

Line 109: A good addition to this paragraph would be an opening 1-2 sentences stating what this study sought to do; directly linking to the knowledge gap described above. Then, go into details on the compilation. Also, change to "… from a select number of GNIP stations and …", as observations of $\delta^{18}O_p$ are provided from several GNIP stations, not the entire network.

Re: Following your suggestion, we introduced the key scientific question with a query at the end of the third paragraph: "*These contradictions lead to a central question: what are the principal drivers controlling $\delta^{18}O_p$ variations across the Asian monsoon region?*" The fourth paragraph then begins with "*To address this question,*" directly echoing the previous context and ensuring narrative coherence. Please see L100–L103 in the revised manuscript.

Meanwhile, we changed "we compiled $\delta^{18}O_p$ observations from GNIP and other published and unpublished sources" to "*we compiled $\delta^{18}O_p$ data from a select number of GNIP stations and other published/unpublished sources.*" Please see L103–L104 in the revised manuscript.

Line 115: The reference cited (Chiang et al., 2020) to justify the use of the IsoGSM2 model across the entire east-west transect lacks some significance. The study by Chiang et al., 2020 only considers a section of the East Asian monsoon region and provides no evidence of model performance and modeled $\delta^{18}O_p$ results for the South Asian monsoon region. How reliable is the IsoGSM2 model in capturing $\delta^{18}O$ signals across the entire spectrum of the east-west transect considered here? Also, what is the purpose of re-modelling data that has been measured directly?

Re: Thank you for raising this important point about the justification and reliability of the IsoGSM2 model data. We agree with your assessment, and following our earlier decision to streamline the manuscript, we have now removed all IsoGSM2 model data and related analyses from the paper. Consequently, the referenced sentence (Chiang et al., 2020) and the associated discussion have been deleted to ensure consistency. Please see L107 in the revised manuscript.

Line 119: This is introduced completely out of the blue, without justification or reasoning. The updraft hypothesis (and theory underpinning it) should be introduced earlier.

Re: We agree that the introduction of "atmospheric updraft" was indeed abrupt and lacked any explanation. Following your suggestion, we rewrote these sentences. That is, we modified these sentences to "*We then identify the spatiotemporal patterns of $\delta^{18}O_p$ along the transect. Finally, we explore the mechanisms governing $\delta^{18}O_p$ variations across the Asian monsoon region. Our findings propose a paradigm shift from the monsoon moisture advection to vertical atmospheric dynamics for understanding this region's $\delta^{18}O_p$ variations, which provides significant implications for the climate signals preserved in Asian $\delta^{18}O_s$ records.*" Please see L107–L113 in the revised manuscript.

Lines 123-124: Strange statement - isn't this obvious if you're already stated that your transect spans the AM domain?

Re: We agree that the original phrasing was indeed somewhat redundant. Following

your suggestion, we rewrote the sentence to "*To investigate seasonal $\delta^{18}O_p$ variations between the East Asian and South Asian monsoon regions, we established an east–west transect (20–27° N, 70–125° E) with ~98° E as the dividing line*[55,56]." Please see L115–L117 in the revised manuscript.

Line 128: Still excessive reference to supplementary information. We appreciate there is lots to consider and not all will fit into the main text, but we think there is a much neater way to refer to these extra materials. For example: "Details for each of the sites considered in this study are included in a supplementary file."

Re: We found that in the final paragraph of the Introduction section, we had already mentioned Supplementary Table 1 and Supplementary Table 2 (Please see L105–L106 in the revised manuscript), providing specific information about the 12 primary and 34 ancillary sites. Therefore, it was unnecessary to cite them again here. Accordingly, we removed these citations. Please see L118–L119 in the revised manuscript.

Line 136: The dividing line at 98°E is not clearly visible in Figure S3, which is why it is not meaningful to rely on this figure to support the distinction between the East Asian and South Asian monsoon regimes.

Re: We agree with your observation that the dividing line at 98°E is not clearly visible in Figure S3. This is primarily due to the coarse spatial resolution of the IsoGSM2 model (2.5°×2.5°, approximately 250 kilometers). For instance, sites such as Ruili located 15 kilometers west of the dividing line, and Tengchong located 30 kilometers east of the dividing line, are actually much closer to the dividing line than the scale of a single grid. Consequently, the model cannot capture the seasonal patterns of observed $\delta^{18}O_p$ between the two sites.

As stated above, we removed the IsoGSM2 model data. Accordingly, we removed this sentence as well. Please see L128 in the revised manuscript.

Line 137: regimes or regions (see lines 124, 140 etc.)

Re: As stated above, we removed the IsoGSM2 model data. Accordingly, we removed this sentence as well. Please see L128 in the revised manuscript.

Lines 138-139: Why? What is being assessed/tested?

Re: We realized that the original sentence failed to clearly articulate the scientific motivation for this analysis. Following your suggestion, we added a few introductory sentences before discussing the spatial characteristics of $\delta^{18}O_p$ to provide better contextual information. That is, before "To test this expectation, we analyzed the spatial distribution of multi-year mean monthly $\delta^{18}O_p$ values along the transect from east to west for each month," we added some sentences ("*Previous studies have often attributed $\delta^{18}O_p$ variations in the South and East Asian summer monsoon regions to a "rainout effect" occurring along long-distance monsoon moisture advection from the Indian Ocean[50-53] (occurring within the advection; hereafter denoted as the "rainout effect$_{ad}$"). This mechanism predicts a "west-high, east-low" $\delta^{18}O_p$ pattern along the transect.")*. Please see L129–L133 in the revised manuscript.

Line 151: Not sure this institution detail is needed - city name is sufficient

Re: Following your suggestion, we changed "Institute of Karst Geology" to "*Guilin.*" Please see L141 and L789 in the revised manuscript.

Line 162: Okay so this clarifies why the model is needed. But this clarification must be made when the model is first introduced, otherwise it's purpose appears redundant. It would also be helpful to incorporate the observed $\delta^{18}O_p$ into Figure S6 to support the argument of "strong consistency" between modeled and observed $\delta^{18}O_p$ data.

Re: As stated above, we removed the IsoGSM2 model data. Accordingly, we removed this sentence as well. Please see L149 in the revised manuscript.

Lines 165-166: Again, state the purpose of doing this as we are now starting a new section. Why is knowing this influence important?

Re: As stated above, we removed the "$\delta^{18}O_p$ and the advection of monsoon moisture"

Section. Accordingly, we removed this sentence as well. Please see L166 in the revised manuscript.

Line 172-174: Please provide a reference for the additional moisture source contributions.

Re: As stated above, we removed the "$\delta^{18}O_p$ and the advection of monsoon moisture" Section. Hence, we moved this sentence into the Supplementary Information. Following your suggestion, we provided a reference (i.e., Zhang et al., 2021). Please see L39 in the revised Supplementary Information.

Reference:

Zhang, J. et al. Coupled effects of moisture transport pathway and convection on stable isotopes in precipitation across the East Asian monsoon region: Implications for paleoclimate reconstruction. *J. Climate* **34**, 9811–9822 (2021).

Line 168-190: The authors observe a continuous decrease from May to October (Line 184). So, what about the coupling between $\delta^{18}O_p$ and moisture transport during late spring to early summer (e.g. May, June, July).

Re: From late spring to early summer (e.g., May, June, July), as the moisture transport from the Indian Ocean intensifies, the $\delta^{18}O_p$ in the South Asian monsoon region correspondingly decreases. This relationship indicates that the two are coupled during this period from late spring to early summer (e.g., May, June, July). Following your comments, we changed this sentence to "*This disconnect implies that although $\delta^{18}O_p$ tracks South Asian monsoon moisture transport during the early summer, it does not do so directly from late summer to mid-autumn.*" Please see L31–L33 in the revised Supplementary Information.

Lines 188-190: This point is already made in the introduction, so here is it not more appropriate to say "confirms that..."?

Re: As stated above, we removed the "$\delta^{18}O_p$ and the advection of monsoon moisture"

Section. Hence, we removed this sentence. Please see L166 in the revised manuscript.

Lines 191-195: Very long sentence – split.

Re: As stated above, we moved this paragraph to Text S2 in the Supplementary Information. Moreover, we split and rewrote this long sentence to "*In this case, along this east–west transect, $\delta^{18}O_p$ is expected to exhibit a "high in the west, low in the east" pattern. This is because the heavy isotopes in moisture from the Indian Ocean are gradually depleted by the "rainout effect$_{ad}$" occurring along the monsoon moisture advection. However, in our study $\delta^{18}O_p$ values increase rather than decrease from the middle of the transect to the easternmost sites during each monsoon season month (June–September) (Fig. 2e-h).*" Please see L43–L48 in the revised Supplementary Information.

Lines 216-221: Repeats a lot of the points made in the introduction, so we are not sure this is needed here. We also think you need to more clearly differentiate between the classic rainout effect (traditional view), and the rainout effect you propose to be influenced by updraft (new view). This could be use of different acronyms with subscripts, or a different name for the mechanism you are proposing, but having them so similar runs a high risk of confusing the reader.

Re: As stated above, the "$\delta^{18}O_p$ and the advection of monsoon moisture" section has been removed. Correspondingly, we removed these sentences. Please see L166 in the revised manuscript.

Following your suggestion, we used distinct acronyms with subscripts to more clearly differentiate between the two interpretations: the classic rainout effect (traditional view), occurring during advection (hereafter "rainout effect$_{ad}$"), and the new rainout effect we propose, influenced by updraft (hereafter "rainout effect$_{au}$"). Please see L131–132 and L174–175 in the revised manuscript.

Line 231: Moisture isotopes? You mean oxygen isotopes?

Re: Yes, they are oxygen isotopes. Following your suggestion, we changed “Moisture isotopes” to “*the $\delta^{18}O$ of moisture*”. Please see L173 in the revised manuscript.

Lines 235-237: See point above - the two "rainout effects" need more clearly separating. Also correct to “reduces”.

Re: As stated above, we used distinct acronyms with subscripts to more clearly differentiate between the two interpretations. Please see L131–132 and L174–175 in the revised manuscript.

Following your comment, we changed “reduce” to “*reduces*.” Please see L179 in the revised manuscript.

Line 252-255: In our opinion, we do not see clear evidence of westward migration of low OLR and vertical velocity during the pre-monsoon season. In contrast, a spatial movement of low OLR and vertical velocities from east to west during the monsoon season is more clearly discernible. During the pre-monsoon season, it appears that a change in OLR and vertical velocity has taken place over the entire transect over time, rather than a continuous spatial change across the transect.

Re: We agree with your opinion. During the pre-monsoon season, the zones of low OLR and vertical velocity expanded westward, rather than merely migrate. Following your comments， we changed “migrate” to “expand.” Please see L198 in the revised manuscript.

Line 265: At this point, it is difficult for the reader to understand where Sagar is located within the transect.

Re: Following your suggestion, we defined the location of this point in the manuscript. That is, we added “*located at the west of the middle of the transect*” in the sentence. Please see L211 in the revised manuscript.

Line 280-281: Which figure shows the correlation between observed $\delta^{18}O_p$ and

vertical velocity. Figure 4 shows only the correlation between $\delta^{18}O_p$ and OLR.

Re: In the previous revision, Figure S11 of the Supplementary Information illustrated the relationship between IsoGSM2 simulated $\delta^{18}O_p$ and vertical velocity. However, as stated above, we removed the IsoGSM2 data. Consequently, we also removed this sentence and the figure. Please see L225 and L227 in the revised manuscript.

Lines 303-306: This is the level of clarity that is needed in the earlier sections.

Re: Thank you for your recognition of our efforts.

Line 314: What does "complex moisture sources" mean?

Re: The phrase "complex moisture sources" mentioned in the previous revision originated from Jiao et al. (2019; 2020), who found that the simpler the moisture source, the more pronounced the altitude effect tends to be. Conversely, the more complex the moisture sources, the less evident the altitude effect becomes, and it may even lead to an inverse altitude effect.

However, as stated above, we deleted the discussion regarding the "inverse altitude effect," and therefore this sentence has been removed. Please see L254 in the revised manuscript.

References:

Jiao, Y. et al. Impacts of moisture sources on the isotopic inverse altitude effect and amount of precipitation in the Hani Rice Terraces region of the Ailao Mountains. *Sci. Total Environ.* **687**, 470–478 (2019).

Jiao, Y., Liu, C., Liu, Z., Ding, Y. & Xu, Q. Impacts of moisture sources on the temporal and spatial heterogeneity of monsoon precipitation isotopic altitude effects. *J. Hydrol.* **583**, 124576 (2020).

Line 319: Or low $\delta^{18}O$ moisture?

Re: It denotes not "low $\delta^{18}O$ moisture" but rather "low $\delta^{18}O$ values in precipitation."

However, as stated above, to ensure the article remains concise and logically coherent, we removed this sentence. Please see L254 in the revised manuscript.

Lines 322-323: Would this also not be affected by the direction of air mass travel toward the Asian landmass? While there may be more mountains in certain regions, the amount of overland travel that the air does prior to reaching the mountains will also differ. We think this point needs more exploration, as orographic precipitation and updraft is a big consideration here given the unique geography of east and south Asia. It's not enough to simply rule it out based on a single graph.

Re: We agree with your view that the mechanisms through which topography influences precipitation are highly complex. It depends not only on static elevation but also on factors such as the direction of air mass travel toward the Asian continent and the amount of overland travel before the air masses reach the mountain ranges, both of which significantly affect the spatial distribution of orographic precipitation. Therefore, it would indeed be insufficiently rigorous to dismiss the influence of topography based solely on a single figure.

However, upon reviewing the context, we realized that exploring deeply into the interactions among these factors would constitute a substantial undertaking. Doing so would not only significantly expand the length of the manuscript but, more importantly, deviate sharply from our core focus. Moreover, our Figure 2 in the main text (Figure R1) has already ruled out the "amount effect" (i.e., the influence of precipitation). Consequently, we removed the discussion regarding the impact of topography on precipitation. Please see L254 in the revised manuscript.

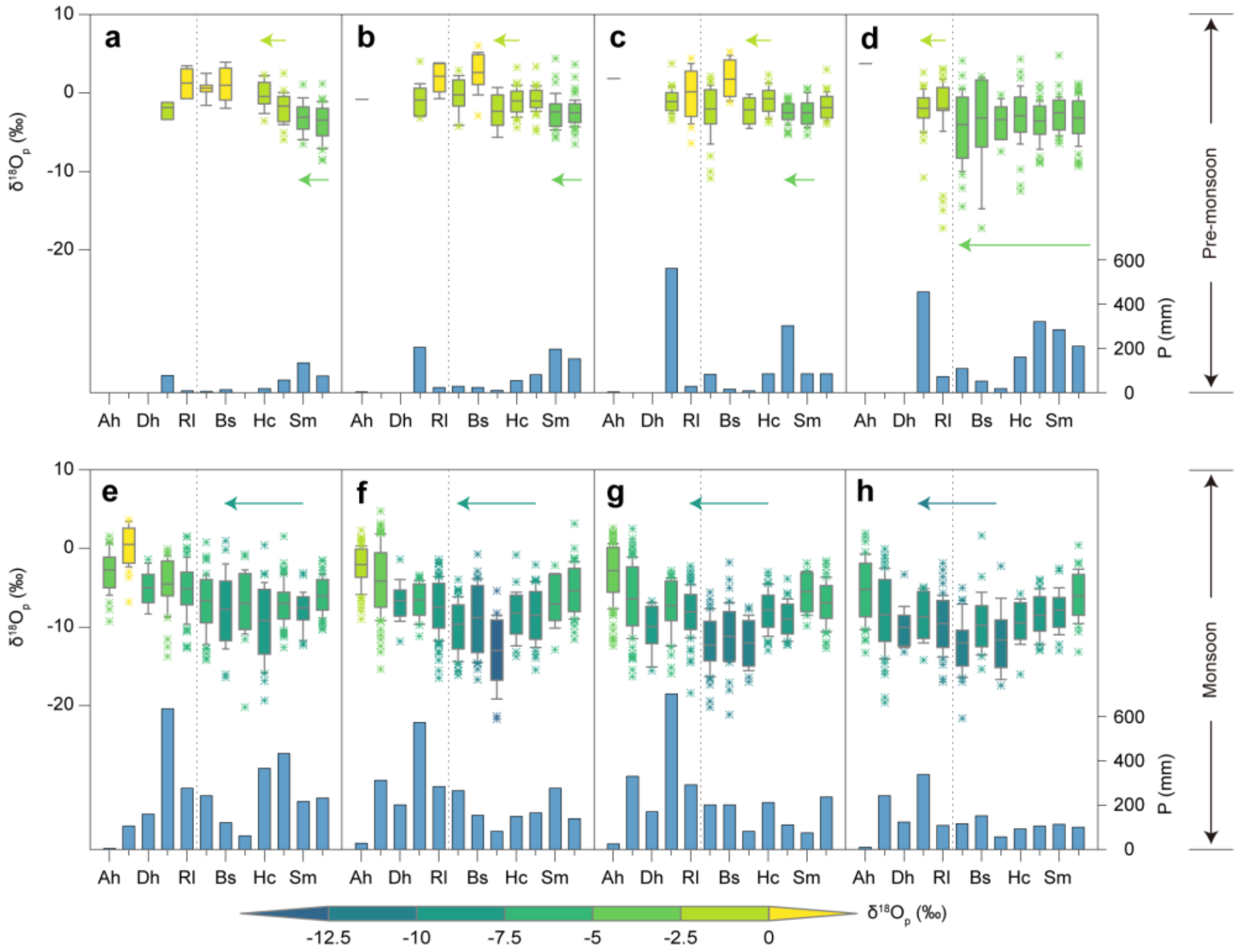

Figure R1. Spatial distributions of $\delta^{18}O_p$ at the 12 primary sites along the transect during the pre-monsoon and monsoon seasons. (a–d) $\delta^{18}O_p$ distributions during the pre-monsoon season: February (a), March (b), April (c), and May (d). (e–h) $\delta^{18}O_p$ distributions during the monsoon season: June (e), July (f), August (g), and September (h). Abbreviations: Ah (Ahmedabad), Dh (Dhanbad), Rl (Ruili), Bs (Baoshan), Hc (Hechi), and Sm (Sanming). In each box plot, the box represents the 20th–80th percentile range, whiskers extend to the 10th–90th percentiles, horizontal lines inside the boxes indicate the mean, and stars represent outliers. Horizontal arrows highlight the westward extension of the low $\delta^{18}O_p$ zone. Dotted lines mark the dividing line of the East Asian and South Asian monsoon regions. Note the spatial patterns of $\delta^{18}O_p$ along this transect mismatch those of precipitation amount, indicating that $\delta^{18}O_p$ is not primarily governed by the "amount effect." $\delta^{18}O_p$ values are expressed as per mil (‰) deviations relative to VSMOW or VSMOW2.

Line 325: For some reason, Sylhet appears to be an exception in terms of precipitation amount, which is also illustrated by the fact that comparatively high precipitation amounts are observed both in the pre-monsoon season and in the

monsoon season. Accordingly, the significance of Sylhet should be treated with caution in order to support the authors' argument here. Furthermore, the precipitation regime of the western transect cannot be assessed on the basis of a single station, as regional hydrology and microclimate could have a significant influence and thus distort the overall picture.

Re: We agree with your comments. Due to its unique geographical location at the head of the Bay of Bengal, the precipitation at the Sylhet station is indeed exceptional, and relying solely on data from a single station to assess the precipitation patterns of the entire western region would indeed introduce bias. Following your comments, we also found that data from the western station Sagar supports this conclusion.

However, as stated above, we removed the discussion on the impact of topography on precipitation. Consequently, we also removed this sentence. Please see L254 in the revised manuscript.

Line 328: Give examples of other monsoon regions.

Re: Following your suggestion, we provided an example (e.g., Africa). Please see L260 in the revised manuscript.

Lines 335-337: Is this the overriding aim of the study? Or another topic being tested as part of a broader study aim? We would also argue that these are not "climate drivers" by definition - they are systems. Drivers would be things such as insolation, ice volume, seasonality, etc...

Re: Yes, this is the primary objective of the study. Following your suggestion, we changed “climate drivers” to “climate systems.” Please see L269–L270 in the revised manuscript.

Line 357: During which season? Within the entire paragraph (lines 347 -359) there is no clear reference to a season.

Re: This paragraph discusses the pre-monsoon season. In the previous revision, there

were four separate paragraphs describing the pre-monsoon period, each of which was brief and lacked clear connections or distinctions between them. Therefore, we have now consolidated this content into a single paragraph and explicitly introduced it as a description of the pre-monsoon season. Please see L273–L296 in the revised manuscript.

Line 372: What do the authors mean with a westward shift of the ITCZ? According to Figure 5, a westward shift of the ITCZ prevails continuously throughout the seasons. The northward shift appears to have a much greater influence over the course of the season.

Re: We agree with your observation that the "northward migration" of the ITCZ is indeed its most prominent seasonal characteristic. However, our focus here is on describing its variation in the zonal (east-west) direction. As shown in Figure 5, we observed that as the ITCZ migrates northward seasonally, its region of intense convection also gradually expands westward, reaching the western Bay of Bengal by August. To avoid potential conceptual ambiguity associated with the term "shift" and to more accurately describe this spatial change process, we changed "shifts westward" to "expands westward." Please see L304 in the revised manuscript.

Lines 377-378: Does it? Or does it's expression simply change? The way this is worded, it sounds as though the ITCZ disappears (which if course it doesn't)

Re: We agree that the term "diminishes" might be misinterpreted as implying the ITCZ disappears, which is inaccurate since the ITCZ is continuously present. To avoid ambiguity, we revised the sentence to clarify that its influence weakens, i.e., we changed this sentence to "*In September, although the influence of the ITCZ weakens to some extent in the East Asian monsoon region, it remains relatively active in the South Asian monsoon region (Fig. 5h).*" Please see L309–L311 in the revised manuscript.

Lines 384-385: What happens to the moisture during the transport from the Pacific Ocean into the eastern zone of the South Asian monsoon region? One would expect

that a considerable amount of moisture contributes to rainout events in the East Asian monsoon region during the transport across the eastern domain.

Re: As you pointed out, moisture transported from the Pacific to the eastern part of the South Asian monsoon region does indeed contribute to precipitation events within the East Asian monsoon region. However, in September, the westward expansion of the western Pacific subtropical high (WPSH) as it shifts southward creates a high-pressure wedge over the East Asian monsoon region (as shown in Figure R2). This high-pressure system suppresses strong convection in the region. Consequently, despite the presence of moisture transport, the significantly weaker atmospheric updraft compared to the peak monsoon season results in no notable decrease in $\delta^{18}O_p$ values in September (in fact, they are higher than in August). This phenomenon further supports our core conclusion that variations in $\delta^{18}O_p$ are primarily controlled by atmospheric updraft rather than by advective moisture transport or the amount effect.

Indeed, we have even attempted to incorporate this detailed mechanistic explanation into the revised manuscript following your suggestion (draft text below): "*One would propose that the westward expansion of the WPSH in September drives significant Pacific moisture into eastern South Asia, thereby increasing precipitation in East Asia. However, as mentioned above, the atmospheric updraft in the East Asian region becomes relatively weak in September (Fig. 3a, b). Consequently, the correspondingly $\delta^{18}O_p$ values do not show a significant decrease (Fig. 2h).*"

However, during the review of the manuscript, we noted that including this detailed analysis caused the paragraph to become disproportionately long, even longer than the discussion of the peak monsoon period (June-August). This disrupted the overall balance of the manuscript and risked shifting the focus away from our core topic. To address this, we did not include the draft text as mentioned above and rewrote the sentences to "*During this period, the easterly winds driven by the westward expansion of the WPSH collide with the moisture from the Indian Ocean (Supplementary Fig. 10h), thereby strengthening atmospheric updraft in the South Asian monsoon region.*" Please see L312–L315 in the revised manuscript.

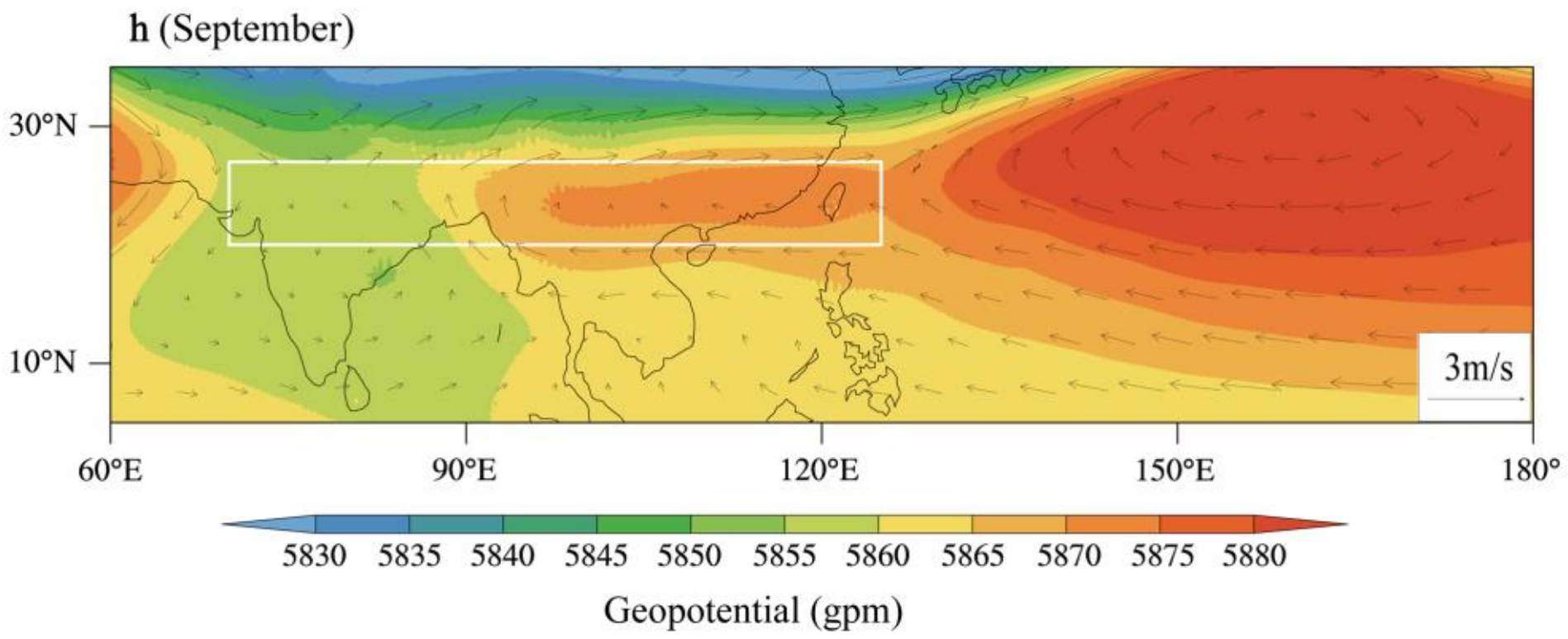

Figure R2. Spatial distributions of 500 hPa monthly geopotential height and wind field in September over 1979–2022. Shading represents geopotential height, arrows indicate the wind field, and the white rectangle marks the study region.

Lines 389-390: Figure 2h shows the lowest variability in $\delta^{18}O_p$ and a less pronounced v-pattern across the entire transect. Figure S15h also shows the lowest variability in precipitation across the entire transect. This suggests that the differences in convective intensity between the South and East Asian monsoon regions were not very pronounced, at least during September. If the ITCZ drives the westward movement of convection so significantly, and convection weakens in the East Asian monsoon region in September (line 380), then a significantly different pattern in $\delta^{18}O_p$ and precipitation amount should be observed in the South Asian monsoon region compared to the East Asian monsoon region during September, possibly causing a more pronounced V-pattern.

Re: We would like to clarify that our data (Fig. 3a, b in the main text and Fig. S4h in the Supplementary Information) indicate that convection in the South Asian monsoon region is stronger than in the East Asian monsoon region.

In fact, the continued influence of relatively active convection on $\delta^{18}O_p$ in the South Asian monsoon region led to lower $\delta^{18}O_p$ values in September than in August. ($\delta^{18}O_p$ values at five sites from the South Asian monsoon region in August and September are as follows: Ruili: August -8.04‰, September -9.53‰; Sylhet: August -6.95‰, September -8.86‰; Dhanbad: August -9.91‰, September -10.04‰; Sagar: August

-6.35‰, September -7.66‰; Ahmedabad: August -2.35‰, September -5.10‰; please see Fig. 1c in the main text).

Indeed, Fig. 2h shows the lowest variability in $\delta^{18}O_p$, and the V-shaped pattern across the entire transect is less pronounced. This likely reflects the South Asian monsoon region's proximity to the Indian Ocean, the dominant moisture source for the broader Asian monsoon system, which generally leads to higher background $\delta^{18}O_p$ values than those observed in the East Asian monsoon region (Yoshimura et al., 2008; Midhun and Ramesh, 2016). Consequently, the V-shaped spatial pattern of $\delta^{18}O_p$ in September across the transect is less distinct than for other months.

Based on the above analysis and following your suggestion, we have revised the relevant descriptions in the main text. That is, we changed the sentence to "*These lead to the lower $\delta^{18}O_p$ values in September than in August across the South Asian monsoon region (Fig. 1c and Supplementary Fig. 1b). It should be noted that September $\delta^{18}O_p$ values in the South Asian monsoon region are not lower than those in the East Asian monsoon region. This may be attributed to the generally higher background isotopic values in the former compared to the latter*[78,79]." Please see L315–L320 in the revised manuscript.

References:

78. Yoshimura, K., Kanamitsu, M., Noone, D. & Oki T. Historical isotope simulation using Reanalysis atmospheric data. *J. Geophys. Res. Atmos.* **113**, D19108 (2008).
79. Midhun, M., & Ramesh, R. Validation of $\delta^{18}O$ as a proxy for past monsoon rain by multi-GCM simulations. *Clim. Dyn.* **46**, 1371–1385 (2016).

Lines 395-397: Using questions as signposts is incredibly helpful for the reader.

Line 398: Not always - they can also record millennial scale signals. It is very much dependant on the archive.

Line 401: "distinct" is a strong word here. A distinct V-shaped pattern is not visible in

Figure S19, and so we advise the authors to be more careful with how definitively they attribute observations to updraft.

Lines 404-405: "this implies that...". Remember the updraft mechanism proposed is new to this study, and so cannot be taken (yet) as conclusive.

Line 415: same as line 401; “closely” is a strong word. While a pronounced V-shape pattern can be observed in Figure 7b, this pattern does not “closely mirror” the pattern observed in annual $\delta^{18}O_p$ values. Please tone down the argument.

Line 426: Not sure how Supplementary Figure 21 supports the point being made, as this wording implies the two conditions are compared.

Lines 699-701: What about resolution/U-Th dating information? Or even, what about the proposed interpretation of $\delta^{18}O$? Not all will have been interpreted in the same way, or capture identical processes. Also, what is the reason for setting the time limit to -50000 AD? This is very non-traditional.

Line 705: Please check sentence structure. Also, what sort of ‘deviations’ are being referred to?

Line 706: eliminated or excluded?

Lines 708-713: Okay, we have several concerns about this. First, creating composite records is not as simple as layering records on top of each other, and merging records runs a high risk of signal smoothing, and amplification of chronological uncertainties. Second, why was no composite record created for records with overlapping periods? This means that there is no transparency in the selection of the removed segments, whereas the inclusion of overlapping segments could provide a statistical basis for determining the extent to which the $\delta^{18}O_s$ signals are representative of the cave and the region.

Line 779: Why not a moving average?

Line 780: We are still not clear how these periods are defined, beyond simply looking at the data. Visual inspection is not sufficient to justify 'chopping up' the data for the purpose intended here, as one could make a case for why several different boundaries could be better. In other words, it is too subjective. One approach could be to run a change point analysis and seeing where significant breaks in the time series' are, or

using already published stratigraphic event timings (e.g., https://www.science.org/doi/10.1126/science.aay5538)

Line 789: How representative is the Fengyu $\delta^{18}O_s$ record to serve as a reference record? How can the authors justify that this record is representative?

Re: We thank you for pointing all these issues (from **Lines 395-397** to **Lines 789**) out. As you pointed out, paleoclimate records are better suited to be presented separately in another paper. We fully agree with your views. Therefore, we have removed $\delta^{18}O_s$ records, including all related analyses, figures, and discussions.

Lines 757-760: Why? These indices are not at all mentioned in the main text...

Re: The East Asian Summer Monsoon Index (EASMI), South Asian Summer Monsoon Index (SASMI), Indian Monsoon Index (IMI), and the Northwestern Pacific Monsoon Index (WNPMI) all refer to "monsoon indices." To ensure a smooth flow of the main text, we moved this section to the Supplementary Information. Please see L54–L57 in the revised Supplementary Information.

Line 875: It would be helpful to clarify in the legends of subfigures b and c that the graphs refer to the primary precipitation sites and not the cave sites.

Re: As stated above, we removed paleoclimate records ($\delta^{18}O_s$ records). Consequently, we removed the locations of the 13 caves from Fig. 1 in the main text. Please see the new Fig. 1 in the revised manuscript.

The current subfigures (b) and (c) specifically refer to the seasonal characteristics of $\delta^{18}O_p$ at the precipitation sites.

FIGURES

Fig. 1: Much improved.

Re: Thank you for your previous suggestions, which have led to significant improvements in this figure.

Fig. 5: Lines 945-949: Lots of text, but not entirely clear what this is showing. Is this showing directly measured data? Or a model output/estimation?

Re: It is a model output. That is, we applied a multivariate probabilistic framework (model) developed by Mamalakis & Foufoula-Georgiou (2018) and Mamalakis et al. (2021). The driving data for the model are derived from ERA5 reanalysis datasets (specifically, Cloud cover and precipitation). To improve clarity and readability, we simplified the caption and moved the detailed technical description of the calculation method to the "Materials and Methods" section. Please see L644–L655 in the revised manuscript.

Reference

Mamalakis, A. & Foufoula-Georgiou, E. A multivariate probabilistic framework for tracking the intertropical convergence zone: Analysis of recent climatology and past trends. *Geophys. Res. Lett.* **45**, 13,080–13,089 (2018).

Mamalakis, A. et al. Zonally contrasting shifts of the tropical rain belt in response to climate change. *Nat. Clim. Change* **11**, 143–151 (2021).

Fig. 6: This new addition is great, and extremely helpful. We suggest editing the wording to say: "westward migration". Also, why is the ocean red?

Re: Following your suggestion, we changed "Migrate westward" to "*Westward expansion*" during the pre-monsoon season (Figure R3a) and changed "Migrate westward" to "*Westward migration*" during the monsoon season (Figure R3b). Please see the new Fig. 6 in the revised manuscript.

The red shading over the ocean represents the Indo-Pacific Warm Pool (IPWP), symbolizing the region's characteristic warm sea surface temperatures.

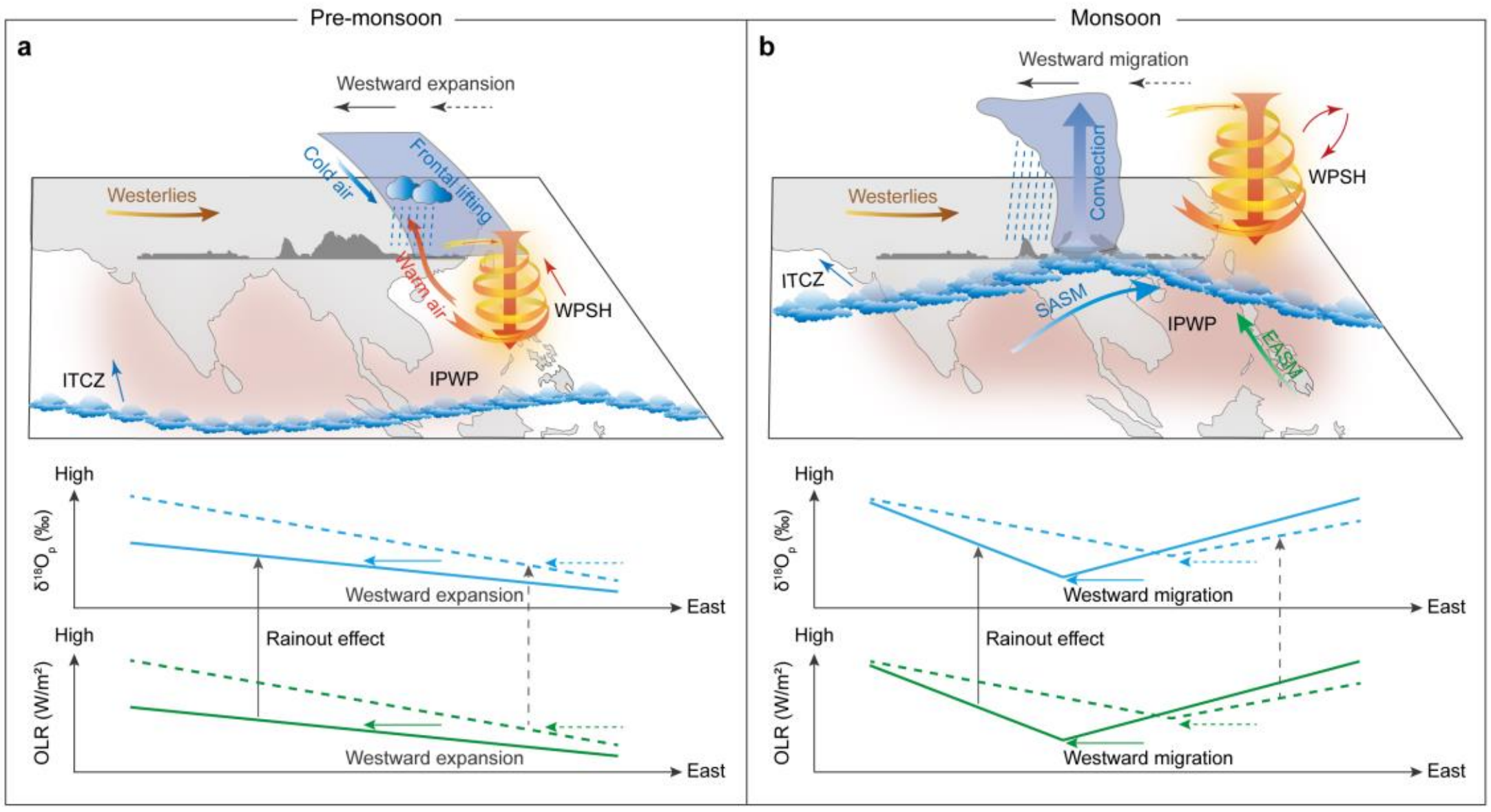

Figure R3. Schematic illustration of mechanisms controlling the spatial distributions of $\delta^{18}O_p$ along the transect during the pre-monsoon and monsoon seasons. (a) Pre-monsoon season. (b) Monsoon season. Horizontal solid arrows indicate the final state (May during the pre-monsoon season or September during the monsoon season) of stronger atmospheric updraft and lower $\delta^{18}O_p$ migrating westward, while horizontal dashed arrows represent the prior state (February during the pre-monsoon season or June during the monsoon season). The deep gray shading in the topographic map shows the terrain profile along the east–west transect. Abbreviations: ITCZ, Intertropical Convergence Zone; IPWP, Indo-Pacific Warm Pool; WPSH, Western Pacific subtropical high; SASM, South Asian summer monsoon; EASM, East Asian summer monsoon; OLR, outgoing longwave radiation. The blue and red arrows adjacent to ITCZ and WPSH indicate their movement directions during the pre-monsoon and monsoon seasons. Figure created with Adobe Illustrator 2024.

Fig. S2: Label for the x-axis is missing.

Re: We are sorry for this carelessness. We added “Label for the x-axis” (Figure R4). Please see new Fig. S1 in the revised Supplementary Information.

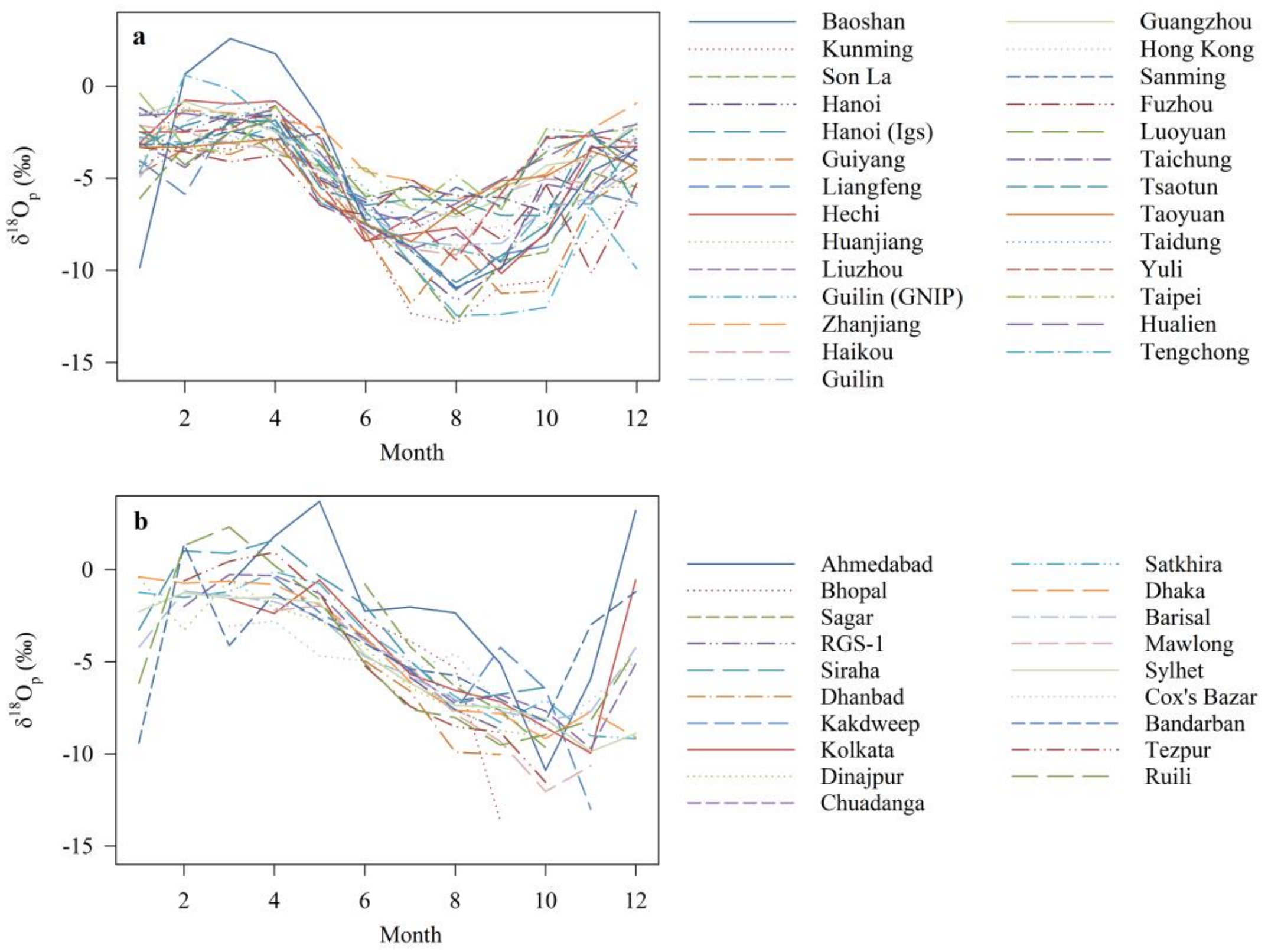

Figure R4. Seasonal variations in observed multi-year mean $\delta^{18}O_P$ at 46 precipitation sites across the Asian monsoon region. (a) East Asian monsoon region. (b) South Asian monsoon region. $\delta^{18}O_P$ values are expressed as per mil (‰) deviations relative to VSMOW or VSMOW2.

Fig. S3: Label for the x-axis is missing. In addition, we suggest aligning the figures in an east-west transect, as the authors refer to an east-west transect throughout the main text. This will facilitate the comparability of the proxy data and IsoGSM2-simulated data referred to by the authors in lines 135-136.

Re: We are sorry for this carelessness. As stated above, we removed the IsoGSM2 model data. Hence, we removed this figure as well. Please see L120 in the revised Supplementary Information.

Fig. S9: The figure refers to the pre-monsoon season on the right-hand side, but the caption refers to the post-monsoon season.

Re: We thank you for pointing this issue out. This figure corresponds to the

post-monsoon season. Hence, we changed "pre-monsoon" to "*post-monsoon*" (Figure R5). Please see new Fig. S5 in the revised Supplementary information.

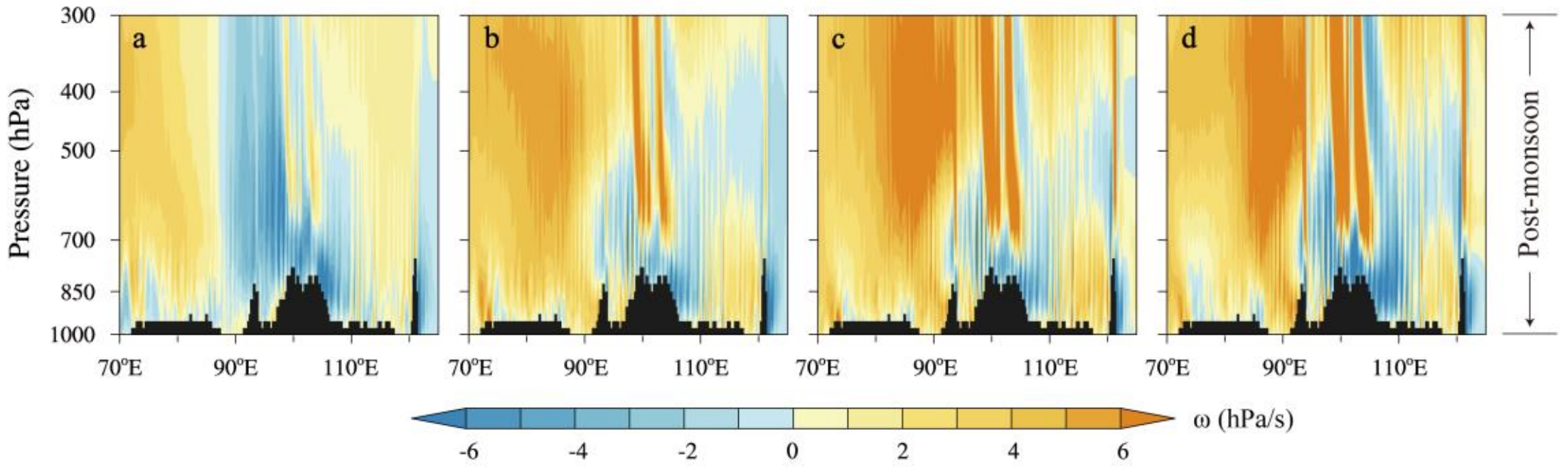

Figure R5. Vertical profiles of meridional average vertical velocity (ω) from 1979 to 2022 during the post-monsoon season. The meridional average is calculated over 20–27° N. Negative and positive ω values indicate upward and downward motions, respectively. The post-monsoon season includes October (a), November (b), December (c), and January (d).

Reviewer #2 (Remarks to the Author):

I co-reviewed this manuscript with one of the reviewers who provided the listed reports. This is part of the Nature Communications initiative to facilitate training in peer review and to provide appropriate recognition for Early Career Researchers who co-review manuscripts.

Re: We thank you very much for providing constructive comments and suggestions on our manuscript. Your comments and suggestions have significantly improved the quality of our manuscript. We have tried to address all the concerns raised by you and have carefully revised the manuscript. Please see our response as mentioned above.

Reviewer #3 (Remarks to the Author):

The authors provided comprehensive replies to the comments and have conducted a good amount of extra analysis. The authors have also provided a restructured manuscript and have improved the clarity of the writing and arguments. This revised manuscript provide is a much-refined version and is much easier to read. The additional references and analyses have provided further clarification.

Re: We sincerely thank you constructive comments and suggestions, which have significantly improved the quality of our manuscript. We have tried to address all the concerns raised by you and have carefully revised the manuscript as follows.

I am pleased the authors have taken time to address the methodology for the relative values of the speleothems. One minor point I could make, is that, at current, the text relies the reader to assume why this method has been chosen. A sentence or two explaining why this method is appropriate, and what it achieves, would help the reader. (One could argue that, assuming no shifts in the moisture source, then, based on the authors' logic, we should always expect every speleothem record to have a predictable longitudinal offset, irrespective of time, and thus we shouldn't need the relative values?).

Re: Thank you very much for your positive feedback on our efforts in the previous round of revisions. You are right. We agree with your view. If we could disregard the time factor, meaning that the $\delta^{18}O_s$ records from all 13 caves covered identical and continuous time periods, then the "relative values" method would indeed be unnecessary. However, following the comments from Reviewers #1 and #2, we removed all sections related to the $\delta^{18}O_s$ records from the manuscript (including all analyses, figures, and related discussions) in order to keep the manuscript more focused on the core argument regarding modern precipitation isotopes.

The additional work on the altitude effect I believe adds robustness to the authors' points. Indeed, the seasonal aspect shows that, whilst there is an altitude effect when

one looks at annual precipitation, this is not present seasonally. Thus, showing that another alternative (and seasonally driven) factor is a dominant control on precipitation $\delta^{18}O$, and that correlation with altitude (at an annual scale) may not be causation.

Re: Thank you very much for your positive comments. We did find an altitude effect in the analysis of annual $\delta^{18}O_p$, but this effect was not observed at the monthly scale.

However, I think it is very important that the authors do plot precipitation amount in the main text. I think rainfall data could be added to figure 2. The authors would need a secondary axis (on the right) and simply add the precipitation from fig R22 underneath the $\delta^{18}O_p$ boxplots. I really think this helps to demonstrate that $\delta^{18}O_p$ and rainfall amount are disconnected.

Re: Following your suggestion, we moved the precipitation data from Figure S15 (fig R22 in the previous response letter) in the Supplementary Information to Fig. 2 in the main text (Figure R6). Please see new Fig. 2 in the revised manuscript.

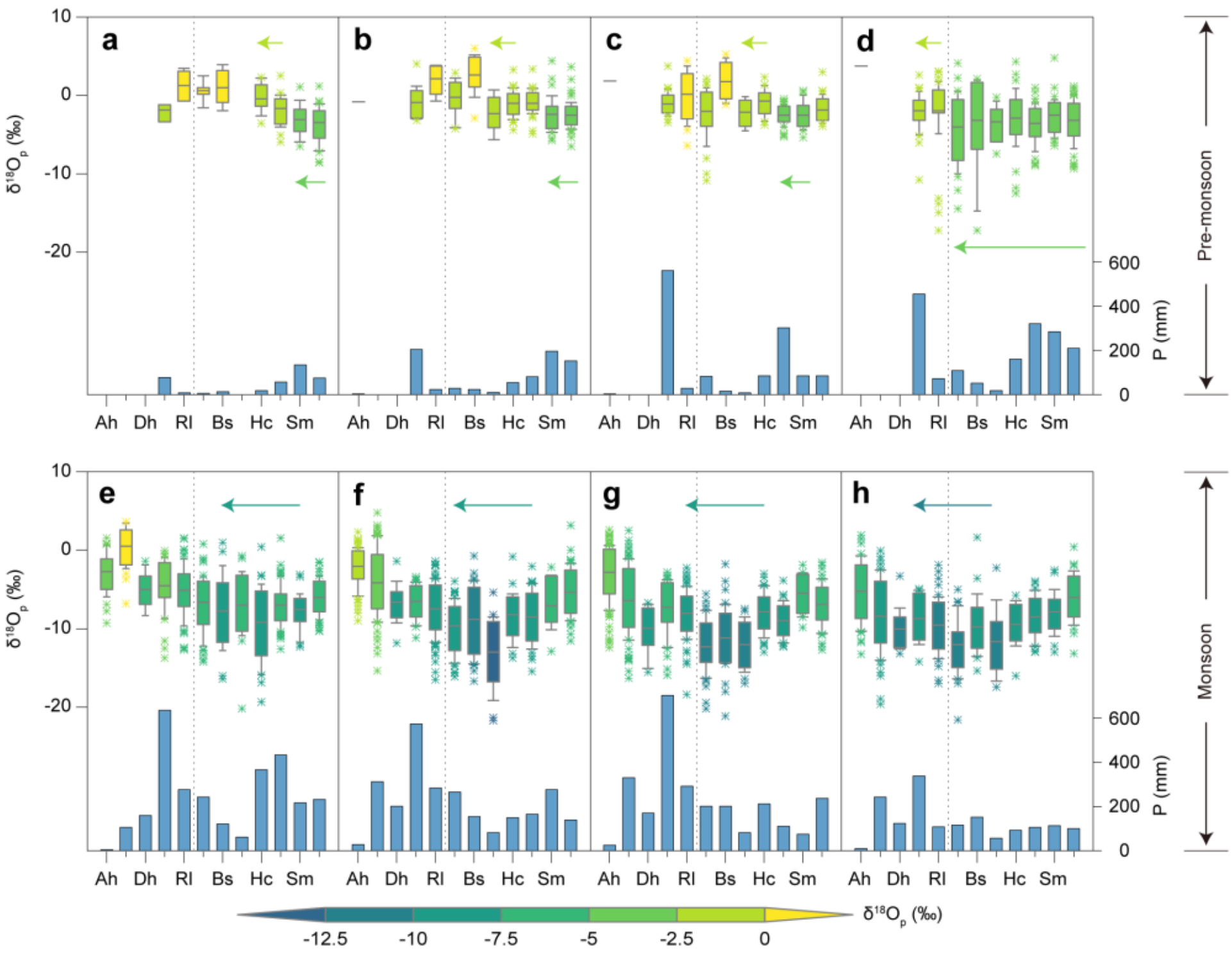

Figure R6. Spatial distributions of $\delta^{18}O_p$ at the 12 primary sites along the transect during the pre-monsoon and monsoon seasons. (a–d) $\delta^{18}O_p$ distributions during the

pre-monsoon season: February (a), March (b), April (c), and May (d). (e–h) $\delta^{18}O_p$ distributions during the monsoon season: June (e), July (f), August (g), and September (h). Abbreviations: Ah (Ahmedabad), Dh (Dhanbad), Rl (Ruili), Bs (Baoshan), Hc (Hechi), and Sm (Sanming). In each box plot, the box represents the 20th–80th percentile range, whiskers extend to the 10th–90th percentiles, horizontal lines inside the boxes indicate the mean, and stars represent outliers. Horizontal arrows highlight the westward extension of the low $\delta^{18}O_p$ zone. Dotted lines mark the dividing line of the East Asian and South Asian monsoon regions. Note the spatial patterns of $\delta^{18}O_p$ along this transect mismatch those of precipitation amount, indicating that $\delta^{18}O_p$ is not primarily governed by the "amount effect." $\delta^{18}O_p$ values are expressed as per mil (‰) deviations relative to VSMOW or VSMOW2.

Whilst I agree with the authors findings (and that vertical processes have been overlooked), I still suggest that they may wish to future-proof their work with a few words of caution in the text. One of the short-comings of much of the $\delta^{18}O_p$ work in the Asian monsoon region is that papers frequently advocate for a new understanding of $\delta^{18}O_p$ and overturning of previous work, only to be superseded a few years later. Likewise, as I alluded to in my previous review, traditional factors (e.g., rainfall amount) as well as others may still have another (perhaps less dominant) effect on the $\delta^{18}O_p$. This is why I suggested to conduct a multiple regression with numerous variables along this specific transect (not to try to trip up the authors, but to expand understand the contribution of multiple factors). So, perhaps the authors may wish to apply a little caution and avoid this trap by 1) do not state that there is a "false-altitude effect", but simply explain (as they have) how vertical processes independent of terrain elevation can provide additional information on $\delta^{18}O_p$, and 2) add to their conclusion a statement about how future research in the region should develop (integration of vertical processes, horizontal processes, altitude, moisture source, etc) to provide a holistic (rather than single effect) model for understanding $\delta^{18}O_p$. I admit, however, that much of this is stylistic and a matter of personal taste.

Re: We thank you for pointing these issues out. We have tried to address all the issues

as follows.

1) With regard to "false-altitude effect:" Following your suggestion, we removed the term "false-altitude effect" and the corresponding discussion. That is, we changed the discussion to "*One might argue that terrain could influence $\delta^{18}O_p$, given the abundance of high mountains in the middle of the transect. To assess the potential influence of terrain on $\delta^{18}O_p$, we examined, on a monthly basis, whether $\delta^{18}O_p$ values are consistently negatively correlated with altitude. Unfortunately, the results show that correlations change over time from weak positive, weak negative, and then to strong negative (Fig. 3c), reflecting the westward expansion or migration of low $\delta^{18}O_p$ from the pre-monsoon to monsoon seasons. This temporal variability stands in clear contrast to the inherent stability of the terrain. In addition, classification of sites by Köppen-Geiger climate zone[68] (Supplementary Fig. 6) or UNEP-WCMC altitude ranges[69] (Supplementary Fig. 7) shows inconsistent or absent negative $\delta^{18}O_p$–altitude correlations. These findings demonstrate that terrain exerts a limited influence on $\delta^{18}O_p$ variations across the Asian monsoon region.*" Please see L244–L255 in the revised manuscript.

2) With regard to "a holistic model for understanding $\delta^{18}O_p$:" Following your suggestion, we added some sentences on the prospects for future study in the "Implications and Outlook" section, i.e., "*We acknowledge that $\delta^{18}O_p$ variations arise from the interplay of multiple factors: vertical/horizontal processes, topography, and meteorological conditions (e.g., temperature and precipitation). We thus advocate incorporating these multiple factors to develop a holistic (rather than single-effect) model for understanding $\delta^{18}O_p$ variations not just across the Asian monsoon region but globally.*" Please see L332–L336 in the revised manuscript.

With exemption of these comments, I think this manuscript does indeed make an interesting contribution to understanding the isotopic composition of rainfall in the South Asian and East Asian monsoon region. The text has been substantially improved and the authors make a convincing case that vertical processes, rather than

only horizontal processes, impact $\delta^{18}O_p$. I think this would make an interesting published article and I am supportive of their work.

Re: We sincerely appreciate your constructive comments and recognition of our work.

Specific line and figure comments

Line 80: "Error! Reference source not found"

Re: We are sorry for this carelessness. Following your comment, we made the modification. Please see L81 in the revised manuscript.

Line 93: "dependent on the ratio of water vapor originating from distant oceans and local ocean" "Dependent on the ratio of water vapor originating from distant oceans and a local ocean to an observed site"

Re: We thank you for pointing this issue out. We merged the two sentences "the "circulation effect" where $\delta^{18}O_p$ is dependent on the ratio of water vapor originating from distant oceans and local ocean to an observed site" and "Many studies have also argued that $\delta^{18}O_p$ in the Asian monsoon region reflects moisture sources, particularly South Asian monsoon-related moisture transport", because the latter is just a reformulation of the former. That is, we changed these two sentences to "*Many studies also proposed the "circulation effect," suggesting that $\delta^{18}O_p$ in the Asian monsoon region reflects moisture sources*[45-49]*, particularly South Asian monsoon-related moisture*[50-53]."

Hence, we removed this sentence. Please see L96–98 in the revised manuscript.

Line 92-94: Is this not just a reformulation/localisation of the source water effect?

Re: Yes, this is indeed a rephrasing/localization of the water source effect. As stated above, we merged the two sentences. Please see L96–98 in the revised manuscript.

Line 97: "Error! Reference source not found"

Re: We are sorry for this carelessness. Following your comment, we made the

modification. Please see L98 in the revised manuscript.

Line 180-181: "Error! Reference source not found"

Re: We are sorry for this carelessness. We made the modification. Please see L131 in the revised manuscript.

189: What is meant be monsoon intensity? Amount of rainfall? Wind strength?

Re: Thank you for this important clarification. We recognize that "monsoon intensity" is defined quite differently across disciplinary contexts, and we should have been more explicit.

1) In meteorology and atmospheric dynamics, it typically refers to the strength of the large-scale circulation, often quantified using wind fields (e.g., 850-hPa zonal wind or shear vorticity) or moisture flux.

2) In speleothem paleoclimatology, earlier studies often loosely equated it with precipitation amount, invoking the "amount effect" to interpret $\delta^{18}O$ records.

In our study, we follow the meteorological convention. When we refer to "monsoon intensity," we specifically mean the strength of the atmospheric circulation (i.e., wind fields), rather than local precipitation amount. We have clarified this in the revised Supplementary Information to avoid any ambiguity ("*Here, "monsoon intensity" refers to the strength of atmospheric circulation (i.e., wind fields), rather than local precipitation amount*"). Please see L27-28 in the revised Supplementary Information.

Line 236: "reduce" reduces

Re: We are sorry for this carelessness. Following your comment, we changed "reduce" to "reduces". Please see L179 in the revised manuscript.

Line 318: "support" supports

Re: As stated above, we reorganized this paragraph. Hence, we removed this sentence. Please see L255 in the revised manuscript.

Line 442: https://doi.org/

Re: Following your comment, we removed "https://doi.org/". Please see L346 in the revised manuscript.

Line 471: "ofthe" -> the

Re: We are sorry for this carelessness. Following your comment, we changed "ofthe" to "of the". Please see L375 in the revised manuscript.

Line 569: "49. Baker, A. J. et al. Seasonalityof westerly moisture transport in theEast Asian summer monsoon and itsimplications for interpreting precipitation δ^{18}O. J. Geophys. Res. Atmos. 120, 5850—5862 (2015)."

"Seasonalityof" -> Seasonality of

"theEast" -> The East

Re: We are sorry for this carelessness. Following your comment, we changed "Baker, A. J. et al. Seasonalityof westerly moisture transport in theEast Asian summer monsoon and itsimplications for interpreting precipitation δ^{18}O. *J. Geophys. Res. Atmos.* **120**, 5850–5862 (2015)" to "Baker, A. J. et al. Seasonality of westerly moisture transport in the East Asian summer monsoon and its implications for interpreting precipitation δ^{18}O. *J. Geophys. Res. Atmos.* **120**, 5850–5862 (2015)." Please see L369–370 in the revised manuscript.

Line 570: "itsimplications" -> its implications

Re: Following your comment, we changed "itsimplications" to "its implications". Please see L479 in the revised manuscript.

Line 676: : -> , (or delete "including")

Re: Following your comment, we changed ":" to ",". Please see L592 in the revised manuscript.

Line 698: - tabstop

Re: As stated above, we removed $\delta^{18}O_s$ records, including all related analyses, figures, and discussions. Accordingly, we removed this sentence. Please see L617 in the revised manuscript.

Line 727: note that in this study

Re: As stated above, we removed the IsoGSM2 model data. Accordingly, we removed this sentence. Please see L617 in the revised manuscript.

Fig. S15. Over which time period where these data collected? What is the source of the data?

Re: Thank you for raising this point. The precipitation amount data for the 12 primary sites in Fig. S15 cover the same time periods as the $\delta^{18}O_p$ data. That is, the temporal coverage of the precipitation amount data matches that of the $\delta^{18}O_p$ records. The specific time coverage for each site is shown in Supplementary Table 1. The sources of the precipitation amount data have been provided in the "Meteorological Data" subsection of the Materials and Methods section, i.e., *"Observed precipitation amount data were obtained from the China Meteorological Administration National Meteorological Science Data Center (https://data.cma.cn/) and the National Oceanic and Atmospheric Administration (https://www.ncei.noaa.gov/maps/daily/)."* Please see L619–L622 in the revised manuscript.

Reviewer #4 (Remarks to the Author):

I co-reviewed this manuscript with one of the reviewers who provided the listed reports. This is part of the Nature Communications initiative to facilitate training in peer review and to provide appropriate recognition for Early Career Researchers who co-review manuscripts.

Re: Thank you very much for providing very fair and positive comments. Your comments and suggestions greatly improve the quality of our manuscript. We have tried to address all the concerns raised by you and have carefully revised the manuscript. Please see our response as mentioned above.

Reviewer #5 (Remarks to the Author):

Review report: Zhang et al: Re-interpreting Asian speleothem Isotope records: the hidden role of Atmospheric updraft.

Zhang et al. present an impressive synthesis of published $\delta^{18}O$ precipitation data across the Asian domain and develop a clear, testable hypothesis: that variability in $\delta^{18}O$ precip. is sensitive to atmospheric dynamics, particularly processes linked to vertical motion (e.g., updraft strength).

This is a commendable effort and a step in the right direction. However, the manuscript overlooks a fundamental piece of the argument that cannot be ignored. As currently narrated, it implicitly assumes that $\delta^{18}O$ in precipitation is transferred directly to cave drip waters and then faithfully preserved in speleothem calcite. This assumption is not justified (see for eg Gebregiorgis et al., 2020, Quaternary and references therein for a concise context and starting point on re-interpreting Asian speleothems) and represents a major flaw because it bypasses an extensive body of literature on the complexities of karst hydrology and how these processes shape speleothem $\delta^{18}O$ proxy formation. For example, see (Chen and Li, 2018, GCA; Zhang and Li, 2019, J. Hydrology) for the implications of in-cave processes for $\delta^{18}O$ and δD of drip water; (e.g., Duan et al., 2016, GCA) for storage and mixing in the epikarst; residence-time effects (e.g., Qiu et al., 2023, J. Hydrology and references therein); selective recharge/seasonal weighting (e.g., Duan et al., 2016, GCA; Tian et al., 2021, Frontiers in Earth Science); evaporation (e.g., Hendy, 1971, GCA for foundational insight; Markowska et al., 2016, QSR; Deininger et al., 2012, GCA; Duan et al., 2016, GCA); prior calcite precipitation (e.g., Patterson et al., 2024, Nature Communications); and cave ventilation effects (e.g., Lyu et al., 2024, Sci Total Environ), to name a select few, that we know to attenuate, lag, or even decouple the precipitation signal from the speleothem record. Addressing this properly would require an extensive synthesis of cave-drip $\delta^{18}O$ observations (which are the closest approximation to $\delta^{18}O$ in speleothem not $\delta^{18}O$ precip.) and karst hydrologic controls.

For this reason, I am unfortunately recommending rejection of the manuscript.

I very much appreciate the effort in addressing the reviewers' comments and agree that the proposed atmospheric updraft mechanism may plausibly explain variability in $\delta^{18}O$ precipitation. However, it cannot be extended to $\delta^{18}O$ in speleothems without a rigorous analysis of cave drip-water $\delta^{18}O$ data and consideration of karst geology and hydrology. As such, the manuscript makes an unsupported leap from precipitation isotopes to speleothem calcite isotopes, and the evidence presented cannot be used to critically interpret speleothem $\delta^{18}O$ or warrant publication in Nature Communications.

That said, the $\delta^{18}O$ precipitation-focused synthesis and atmospheric hypothesis would be a good fit in a more discipline-oriented journals such as GRL, but this would require substantial reframing of the manuscript both for scope and the broader implication of the work re: speleothem $\delta^{18}O$ proxy formation.

Re: We are sincerely grateful for your exceptionally detailed and constructive comments, which have been instrumental in helping us strengthen the manuscript. We agree wholeheartedly with your assessment: it was a logical leap to connect precipitation $\delta^{18}O$ directly to speleothem $\delta^{18}O$ without considering the well-documented complexities of karst hydrology and in-cave processes. Your point is well taken and, importantly, aligns with the concerns raised by Reviewers #1 and #2 ("We are not convinced that including paleoclimate records here adds value or is necessary. It almost feels as though these records were added as an afterthought and would be better presented separately in another paper"). We have carefully addressed these issues as follows:

1) Removing the speleothem discussion to maintain focus

To address this fundamental flaw substantively, we have undertaken a significant restructuring of the manuscript. We fully acknowledge that our initial, brief discussion of speleothem records implicitly made the unjustified assumption you identified. As

you correctly pointed out, this ignores a large body of work on cave processes (including drip water isotopes, storage and mixing, residence-time effects, prior calcite precipitation, and ventilation) that can attenuate or alter the original precipitation signal.

Consequently, to keep the narrative tightly focused and avoid this logical leap, we have removed all analysis and discussion related to speleothem $\delta^{18}O$ records. This includes the deletion of the corresponding figure (formerly Fig. 7) and related supplementary figures (Figs. S19–S21), as well as all text that purported to "directly explain" speleothem records. To ensure the scope is clear, we have also changed the title from "Reinterpreting Asian speleothem isotope records: the hidden role of atmospheric updraft" to "*Reinterpreting precipitation isotope records from the Asian monsoon region: the hidden role of atmospheric updraft.*"

As the speleothem section was brief (only 231 words), its removal does not affect the core narrative but instead strengthens it by making the manuscript a coherent story focused solely on modern precipitation isotopes. Importantly, we feel our key original findings remain valid and are worthy of publication in Nature Communications.

2) Clarifying the study's contribution and future perspective

While we have removed the speleothem analysis, we believe our "atmospheric updraft hypothesis," derived from modern precipitation processes, offers a valuable conceptual framework. As you know, similar studies (e.g., Chiang et al., 2020, PNAS; Aggarwal et al., 2016, Nature Geoscience) have used modern process-based insights to offer new ways of thinking about paleo-records. Notably, Chiang et al. (2020) opens with the statement "Speleothem oxygen isotope records over East Asia reveal apparently large and rapid paleoclimate changes over the last several hundred thousand years. However, what the isotopic variation actually represent in terms of the regional climate and circulation is debated. We present an answer that emerges from an analysis of the interannual variation in amount-weighted annual $\delta^{18}O$ of

precipitation over East Asia ...” Following your suggestion, we now offer a more cautious perspective on the implications of precipitation $\delta^{18}O$ more cautiously in the revised “Implications and Outlook” section. That is, we merely suggest that future paleoclimate work may consider reconstructing “atmospheric updraft” from speleothem $\delta^{18}O$ records as a potentially important climate variable, rather than connecting precipitation $\delta^{18}O$ directly to speleothem $\delta^{18}O$.

3) Incorporating your suggestions into the manuscript

We greatly appreciate you providing a clear entry point into these references. As you recommended, we have now added an outlook paragraph that explicitly acknowledges the role of in-cave processes. Specifically, we have added the following text, citing the key references you provided, i.e., *“To better interpret Asian $\delta^{18}O_s$ records, in-cave factors (e.g., dripwater $\delta^{18}O$, selective recharge) (Gebregiorgis et al., 2020; Hendy, 1971; Deininger et al., 2012; Duan et al., 2016; Markowska et al., 2016; Chen et al., 2018; Qiu et al., 2023; Patterson et al., 2024) should also be considered, as they may attenuate, lag, or even decouple the $\delta^{18}O_p$ signal before being archived. In addition, to further enhance interpretation reliability, it is recommended that future work adopt a “link” perspective, integrating $\delta^{18}O_p$ with dripwater $\delta^{18}O$ (Baker et al., 2019).*” Please see L336–L341 in the revised manuscript.

4) Acknowledging your guidance for future research

Your comments have not only helped us clarify the boundaries of this manuscript but have also provided a clear roadmap for our future work. We recognize that a complete understanding of speleothem isotopes requires not just the “outside-cave” (vertical) perspective presented here, but also a dedicated “in-cave” perspective and a "link" perspective that connects the two. We are very grateful for the valuable literature and ideas you have shared, and we plan to pursue these directions in our subsequent research.

In summary, we have transformed the manuscript from one that made an unsupported

leap into a tightly focused study on the mechanisms controlling precipitation $\delta^{18}O$. We have removed the speleothem $\delta^{18}O$ records section entirely, reframed the title and narrative, and added a cautious outlook that acknowledges the complexity of cave processes, citing the literature you suggested. We hope this revised version, now focused on the atmospheric updraft hypothesis as a clear conceptual advance, is better aligned with the standards of Nature Communications. We respectfully request that you reconsider its suitability for publication.

References:

Gebregiorgis, D., Clemens, S. C., Hathorne, E. C., Giosan, L., Thirumalai, K., & Frank, M. A Brief Commentary on the Interpretation of Chinese Speleothem $\delta^{18}O$ Records as Summer Monsoon Intensity Tracers. *Quaternary* **3**, 7 (2020).

Baker, A. et al. Global analysis reveals climatic controls on the oxygen isotope composition of cave drip water. *Nat. Commun.* **10**, 2984 (2019).

Chen, C., & Li, T. Geochemical characteristics of cave drip water respond to ENSO based on a 6-year monitoring work in Yangkou Cave, Southwest China. *J. Hydrol.* **561**, 896–907 (2018).

Deininger, M., Fohlmeister, J., Scholz, D. & Mangini, A. Isotope disequilibrium effects: The influence of evaporation and ventilation effects on the carbon and oxygen isotope composition of speleothems – A model approach. *Geochim. Cosmochim. Acta* **96**, 57–79 (2012).

Duan, W. et al. The transfer of seasonal isotopic variability between precipitation and drip water at eight caves in the monsoon regions of China. *Geochim. Cosmochim. Acta* **183**, 250–266 (2016).

Hendy, C. H. The isotopic geochemistry of speleothems—I. The calculation of the effects of different modes of formation on the isotopic composition of speleothems and their applicability as palaeoclimatic indicators. *Geochim. Cosmochim. Acta* **35**, 801–824 (1971).

Markowska, M. et al. Semi-arid zone caves: evaporation and hydrological controls on $\delta^{18}O$ drip water composition and implications for speleothem paleoclimate

reconstructions. *Quatern. Sci. Rev.* **131**, 285–301 (2016).
Patterson, E. W. et al. Local hydroclimate alters interpretation of speleothem $\delta^{18}O$ records. *Nat. Commun.* **15**, 9064 (2024).
Qiu, W. et al. Oxygen isotope variability in precipitation, dripwater, and modern calcite responding to ENSO based on 11 years' monitoring in Yuhua Cave, Southeast China. *J. Hydrol.* **624**, 129937 (2023).

Minor comments (general)

The debate on the interpretation of Asian speleothem $\delta^{18}O$ records is now decades old and was initially ignited by discrepancies between speleothem-based reconstructions and marine-based proxies of the Asian monsoon (e.g., Clemens et al., 2010, Paleoceanography; Gebregiorgis et al., 2018, Nature Communications; Clemens et al., 2021, Science Advances), rather than as implied in lines 83–90.

Re: We thank you for correcting the intellectual history of this important debate. We acknowledge that our original wording (lines 83–90) inaccurately implied that the debate over Asian speleothem $\delta^{18}O$ interpretation originated solely from climate signals. You are right that this debate was initially ignited by the observed discrepancies between speleothem-based reconstructions and marine-based monsoon proxies (e.g., Clemens et al., 2010; Gebregiorgis et al., 2018; Clemens et al., 2021). To correct this inaccuracy and properly frame the context for our study, we added the following statement to the manuscript: "*However, the interpretation of Asian* $\delta^{18}O_s$ *records remains a subject of rigorous debate, initially ignited by discrepancies between speleothem-based reconstructions and marine-based monsoon proxies (Clemens et al., 2010; Gebregiorgis et al., 2018; Clemens et al., 2021)*." Please see L83–L85 in the revised manuscript. We are grateful to you for helping us situate our study within the correct historical context.

References:

Gebregiorgis, D., Hathorne, E. C., Giosan, L., Clemens, S., Nürnberg, D. & Frank, M. Southern Hemisphere forcing of South Asian monsoon precipitation over the past

~1 million years. *Nat. Commun.* **9**, 4702 (2018).
Clemens, S. C. et al. Remote and local drivers of Pleistocene South Asian summer monsoon precipitation: A test for future predictions. *Sci. Adv.* **7**, eabg3848 (2021).
Clemens, S. C., Prell, W. L., & Sun, Y. Orbital-scale timing and mechanisms driving Late Pleistocene Indo-Asian summer monsoons: Reinterpreting cave speleothem $\delta^{18}O$. *Paleoceanogr. Paleocl.* **25**, PA4207 (2010).

I am not convinced by the statement that the traditional view is that "$\delta^{18}O$ of precipitation is primarily driven by South Asian monsoon moisture advection" (line 296). What is the evidence for this claim?

Re: We thank you for this important observation. You are correct that the statement "These findings are inconsistent with the traditional view that $\delta^{18}O_p$ is primarily driven by South Asian monsoon moisture advection" lacks clear evidentiary support and overstates the consensus in the field. We have therefore removed this claim to avoid making an unsupported generalization. That is, we change "the traditional view" to "*the previous views*." This revised wording is more cautious and accurate. Please see L240 in the revised manuscript.

Re: Mechanisms of atmospheric updraft changes

Line 397–400: Please cite appropriate references here, as there is no conclusive evidence (at least to me) to support the statement that speleothem $\delta^{18}O$ primarily reflects a climate signal at annual timescales. Are you referring to the temporal resolution of the speleothem records? If so, please state this explicitly. More importantly, why not investigate this claim using available cave drip-water isotope data instead of the 12 precipitation sites selected, which provide the closest modern analogue to $\delta^{18}O$ recorded in speleothem calcite?

Re: Thank you for this important methodological comment. You raise a fair point. Upon re-examination, we agree that the original statement (Lines 397–400) lacked direct evidentiary support. Specifically, there is no conclusive evidence that

speleothem $\delta^{18}O$ primarily reflects climate signals at strict annual timescales. Moreover, as you rightly note, if we were to investigate this claim, the most appropriate modern analogue would be dripwater $\delta^{18}O$ data (not precipitation $\delta^{18}O$) given the known complexities of karst hydrology that can modify the isotopic signal.

We acknowledge that using precipitation $\delta^{18}O$ as a proxy for speleothem $\delta^{18}O$ at annual resolution is indeed an oversimplification. For this reason, and to maintain consistency with our broader revisions (as detailed in our response to your major comment), this sentence should be removed. As stated above, we removed the speleothem $\delta^{18}O$ records section, including this sentence. Please see L322 in the revised manuscript.

Response to Reviewers

(Reviewers' statements in black, and our response in blue)

Line163, -the line 163 in the previous manuscript.

L114–L165, -the lines 114-165 in the revised manuscript.

Fig. 1, -the Figure 1 in the revised manuscript.

Figure R1, -the Figure 1 in the response.

REVIEWER COMMENTS

Reviewer #3 (Remarks to the Author):

The authors have made substantial revisions to the text and I appreciate that the authors have provided a section explaining the requirement for further research and provided caveats to their model. As pointed out by another reviewer, the application to the speleothem record will of course require specific in cave monitoring to understand karst processes influencing $\delta^{18}O$ variability, and I think removal of the speleothem section does help the flow of the paper. Overall, I find this an interesting paper worth discussion. However:

Re: We sincerely thank you for your positive and encouraging comments throughout the review process, as well as for your continued recognition of the significance of our study. We greatly appreciate your constructive suggestions, which have helped us substantially improve the clarity and quality of the manuscript. We also appreciate your acknowledgment of the revisions regarding the limitations of our framework and the need for further research, particularly concerning cave monitoring and karst processes influencing speleothem $\delta^{18}O$ variability. In response to the reviewers' comments, we have carefully revised the manuscript and addressed all concerns as detailed below.

As I have noted previously, my primary concern with this work is that the authors have not checked for the correlation of precipitation amount with $\delta^{18}O$ (as done for OLR/vertical velocity). Neither have they checked the correlation between precipitation amount and OLR/vertical velocity. My suspicion is that OLR/vertical velocity are strongly correlated with rainfall amount (as alluded to in Figure 6 [which is a lovely figure]) since they are meteorologically linked, meaning that the influence of either may not be statistically separated. Checking these is necessary since rainfall amount is a key influence of rainfall $\delta^{18}O$. The authors should also check the AIC scores for models running OLR/vertical velocity against $\delta^{18}O$ with and without precipitation amount included as an additional independent (x) variable, which would show whether the model's performance is better or worse with this included.

The authors have previously provided literature-based reasons for not doing this, but given that the authors 1) have the data; 2) there does seem to be some visual relationship between $\delta^{18}O$ and precipitation amount in Fig. 2; 3) there is a well-known process-based understanding of precipitation amount effect on $\delta^{18}O$; 4) convection and rainfall are linked, this is necessary to account for the effect of rainfall amount, distinguish effect of OLR/vertical velocity and confirm the authors results.

The authors previously argued that doing so would detract from the novelty of their work. I respectfully disagree. It is a process to ensure the robustness of the interpretation, and could even help their interpretation. Until this is done, whilst finding this work very interesting, I am finding it difficult to determine whether the results represent a significant advancement based on reasoned statistics, or whether this is an incomplete analysis of the system.

Re: Thank you very much for raising this important issue again. We now realize that

relying solely on literature-based arguments is insufficient, and that direct statistical tests are necessary to evaluate the robustness of our interpretation. Following your suggestions, we have now systematically examined:

1. the correlations between precipitation amount and $\delta^{18}O_p$;
2. the performance of regression models with and without precipitation amount as an additional predictor; and
3. the relationships between precipitation amount and OLR/vertical velocity.

Our analyses consistently show that precipitation amount plays a secondary role in modulating $\delta^{18}O_p$ variability along the transect, whereas atmospheric updraft (represented by OLR/vertical velocity) exerts the dominant control. Below, we summarize the main results.

1) Correlations between precipitation amount and $\delta^{18}O_p$:

Spatial pattern analysis: We first examined the spatial distribution of precipitation amount at the 12 primary sites along the transect (new Fig. R1). If the classical "amount effect" (higher precipitation amount associated with lower $\delta^{18}O_p$) were the dominant control, we would expect the following relationships:

(i) a progressive east-to-west decrease in precipitation amount during the pre-monsoon season; and

(ii) an inverted V-shaped (Λ-shaped) precipitation amount pattern during the monsoon season, corresponding to the minimum $\delta^{18}O_p$ values in the middle of the transect.

However, our observations do not support these expectations. During the pre-monsoon season, only the eastern domain shows an east-to-west decrease in precipitation amount, whereas the western domain exhibits anomalously high precipitation at Sylhet together with relatively high $\delta^{18}O_p$ values. During the monsoon season, precipitation amount follows a V-shaped rather than a Λ-shaped pattern: precipitation amount is relatively low in the middle of the transect, where $\delta^{18}O_p$ reaches its minimum. These spatial patterns are therefore inconsistent with a simple

“amount effect” interpretation.

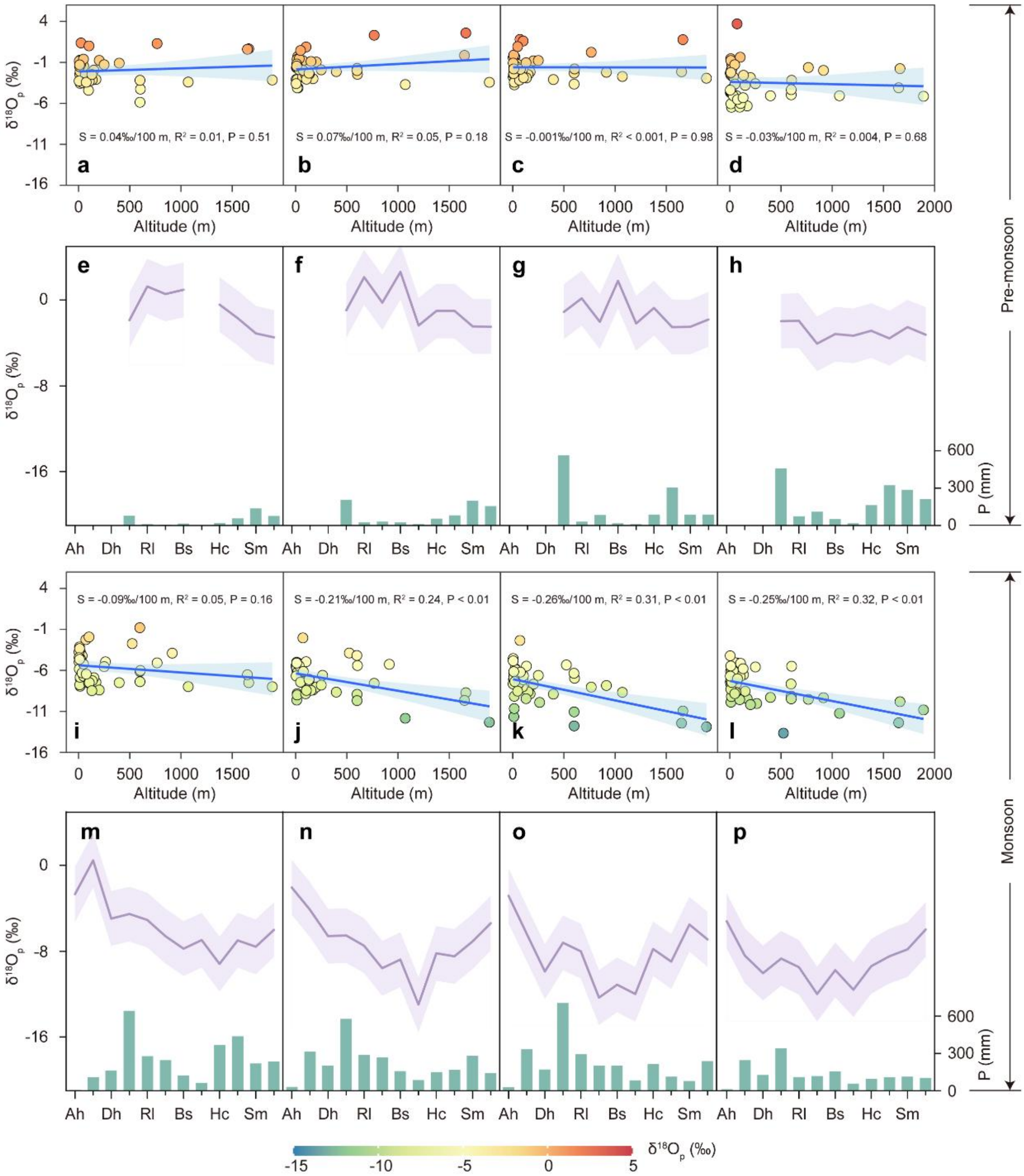

Figure R1. Seasonal variations in $\delta^{18}O_p$–altitude correlations at the 46 sites and spatial changes of $\delta^{18}O_p$ and precipitation amount at the 12 primary sites. a–d, i–l Linear regressions of $\delta^{18}O_p$ at the 46 sites across the study region against altitude, fitted for each month. **e–h**, **m–p** Multi-year mean monthly $\delta^{18}O_p$ (line) with ±1 standard deviation (shading) and precipitation amount distributions during the pre-monsoon and monsoon seasons: February (**e**), March (**f**), April (**g**), May (**h**), June (**m**), July (**n**), August (**o**), and September (**p**). Abbreviations: Ah (Ahmedabad), Dh (Dhanbad), Rl (Ruili), Bs (Baoshan), Hc (Hechi), and Sm (Sanming). $\delta^{18}O_p$ values are

expressed as per mil (‰) deviations relative to VSMOW or VSMOW2.

Site-wise correlation analysis: We further quantified the correlations between $\delta^{18}O_p$ and precipitation amount at all 46 sites (new Fig. R2a, b). Although negative correlations exist on both monthly and multi-year monthly mean timescales, these correlations are substantially weaker than those between $\delta^{18}O_p$ and OLR. In addition, the scatter of $\delta^{18}O_p$ versus precipitation amount is not evenly distributed around the regression lines (Fig. R2a, b), indicating considerable unexplained variability. Together, the spatial-pattern and correlation analyses suggest that precipitation amount is not the dominant factor controlling $\delta^{18}O_p$ along this transect.

2) Model comparison with and without precipitation amount

Following your suggestion, we constructed and compared two linear regression models:

Model 1: $\delta^{18}O_p \sim$ OLR

Model 2: $\delta^{18}O_p \sim$ OLR + precipitation amount

The results are summarized in new Table R1. Model 2 yields a slightly lower AIC (12,363.42) than Model 1 (12,390.25), consistent with your expectation. However, the increase in R^2 is very limited ($\Delta R^2 = 0.0098$). Moreover, five-fold cross-validation shows that Model 1 achieves a slightly lower RMSE (3.1227) than Model 2 (3.1310).

Importantly, in Model 2, the standardized regression coefficient for OLR (0.3514) is approximately three times larger than that for precipitation amount (−0.1158). Collectively, these results indicate that including precipitation amount provides only marginal improvement in model performance. Based on the principle of parsimony, the simpler OLR-only model provides the more robust representation of $\delta^{18}O_p$ variability.

These analyses therefore suggest that atmospheric updraft (represented by OLR) is the

primary driver of $\delta^{18}O_p$ variability, whereas precipitation amount exerts only a secondary influence.

Table R1. Summary of model comparison results

Metric	Model 1 (OLR only)	Model 2 (OLR and P)
AIC	12390.25	12363.42
R^2	0.1697	0.1795
RMSE (Five-fold)	3.1227	3.1310
Standardized regression coefficient (Beta)	—	Beta_OLR = 0.3514; Beta_P = –0.1158

Note: All models are based on the same set of observations (n = 2443).

3) Correlations between precipitation amount and OLR/vertical velocity

As you anticipated, precipitation amount is significantly correlated with both OLR and vertical velocity on monthly and multi-year monthly mean timescales (new Fig. R2c, d). This result is physically consistent with fundamental meteorology, since atmospheric vertical motion is one of the primary controls on precipitation formation.

Importantly, this close coupling implies that precipitation amount does not act as an independent driver of $\delta^{18}O_p$. Instead, its influence likely co-varies with, or is mediated by, atmospheric updraft, which simultaneously governs both precipitation formation and isotopic fractionation processes. Consequently, the dominant role of atmospheric updraft in explaining $\delta^{18}O_p$ variability is physically plausible, whereas the weaker contribution of precipitation amount most likely reflects its dependence on updraft intensity rather than an independent mechanistic control.

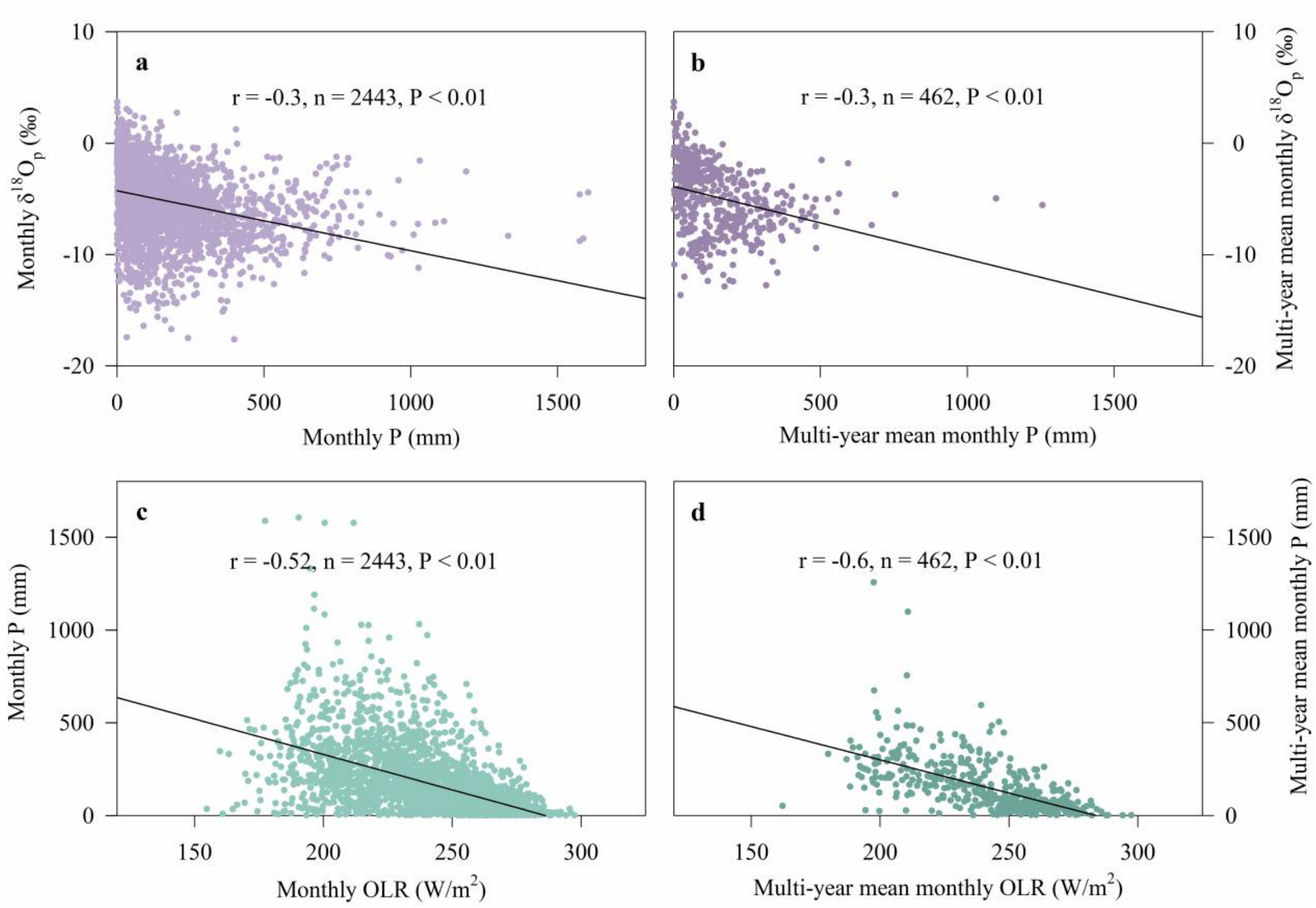

Figure R2. Correlations between precipitation amount (P) and both $\delta^{18}O_p$ and outgoing longwave radiation (OLR) along the transect. a–b Linear regressions of P against the corresponding $\delta^{18}O_p$ at the 46 sites across the study region on different timescales. **c–d** Linear regressions of P at the 46 sites across the study region against the corresponding OLR on different timescales.

In response to your comments, we have substantially revised both the main text and the Supplementary Information. Specifically:

- We added a new paragraph to the main text summarizing the secondary role of precipitation amount and its co-variation with OLR (L262–L271 in the revised manuscript).

- We added a new supplementary section, "Text S6. Assessing the influence of precipitation amount on $\delta^{18}O_p$" (L125–L178 in the revised Supplementary Information), which provides the full statistical analyses and interpretation.

- We also added new Fig. 4 to the main manuscript, and new Fig. S8 and Table S6

to the Supplementary Information.

These results demonstrate that (1) precipitation amount does not explain the spatial pattern of $\delta^{18}O_p$, (2) it provides only marginal improvement in statistical models, and (3) its influence is largely mediated through its co-variation with atmospheric updraft. Therefore, our central conclusion, that updraft is the primary driver, remains robust.

We sincerely appreciate your insistence on this point. Your comments substantially improved the statistical rigor, transparency, and robustness of our study. We now fully agree that explicitly testing the influence of precipitation amount strengthens, rather than detracts from, the novelty and credibility of our central conclusions.

Minor comments

Line 247: Why is this unfortunate? I don't think "unfortunately" is in context.

Re: Thank you for this comment. We agree that the term "unfortunately" was not appropriate in this context. Following your suggestion, as well as the related comment from Reviewer #5, we have removed this wording from the revised manuscript. Please see L252 in the revised manuscript.

Line 248: "correlations change over time from weak positive, weak negative, and then to strong negative". If the correlations are not significant, they don't need to be described as "weak positive" and "weak negative". There is just no significant correlation. Likewise, there is no need to described significant correlations as "strong negative". They are just significant negative correlations.

Re: Thank you for this helpful comment. We agree that non-significant correlations should not be described as "weak positive" or "weak negative," and that significant correlations can simply be described as significant positive or negative correlations. Following your suggestion, as well as the related comment from Reviewer #5, we revised the sentence from

"Unfortunately, the results show that correlations change over time from weak positive, weak negative, and then to strong negative (Fig. 3c), reflecting the westward expansion or migration of low $\delta^{18}O_p$ from the pre-monsoon to monsoon seasons"

to

"*The results show that correlations change over time from "positive" to negative (Fig. 4a-d, 4i-l), reflecting the westward expansion or migration of low* $\delta^{18}O_p$ *from the pre-monsoon to monsoon seasons.*"

Please see L252–L255 in the revised manuscript.

Line 253: We have discussed this previously and I think the authors are right that the seasonal variation shows terrain (altitude) isn't a primary driver of the spatial $\delta^{18}O$ variability. But, "absent negative $\delta^{18}O_p$-altitude correlations" what does this mean? There is either no significant correlation ($P > 0.05$) or a significant correlation ($P < 0.05$).

Re: Thank you for this helpful comment. We agree that the phrase "absent negative $\delta^{18}O_p$–altitude correlations" was ambiguous. Our intention was to indicate that no statistically significant negative correlation ($P > 0.05$) was detected between $\delta^{18}O_p$ and altitude. In other words, the expected "altitude effect"—where $\delta^{18}O_p$ decreases with increasing altitude—was not observed in our study region.

To clarify this point, we revised the text to state explicitly:

"*Classification by UNEP-WCMC altitude ranges*[69] *(Supplementary Fig. 7) shows that the negative correlations were not statistically significant ($P > 0.05$).*"

Please see L258–L260 in the revised manuscript.

In addition, to make the expected relationship clearer, we added the following explanatory sentence:

"*Theoretically, increasing altitude is expected to result in gradually lower* $\delta^{18}O_p$

values, implying a negative correlation between the two."

Please see L249–L251 in the revised manuscript.

We appreciate your careful reading and constructive suggestion, which helped us improve the clarity and precision of the manuscript.

Line 259-263: "Unlike other monsoon regions (e.g., Africa), precipitation in the Asian monsoon region is influenced not only by monsoonal circulation but also by the combined effects of the Intertropical Convergence Zone (ITCZ)" The African Summer Monsoon (as well as the Indian Summer Monsoon and the South American Monsoon) is indeed strongly influenced by the ITCZ. ITCZ can be removed from the list of influences. I think I know what the authors mean, but it's a bit awkwardly phrased.

Re: Thank you for this important clarification. We agree that the ITCZ strongly influences not only the African Summer Monsoon, but also other monsoon systems such as the Indian and South American monsoons. Therefore, the original comparison was inappropriate and potentially misleading.

Following your suggestion, we removed the problematic phrasing and revised the sentence to:

"*In the Asian monsoon region, precipitation is influenced not only by monsoonal circulation but also by the combined effects of the Intertropical Convergence Zone (ITCZ)*[70,71]*, the Indo-Pacific Warm Pool (IPWP)*[72,73]*, and the western Pacific subtropical high (WPSH)*[74-77]."

Please see L275–L278 in the revised manuscript.

We believe this revision more accurately conveys our intended meaning without implying an incorrect contrast with other monsoon systems. Thank you again for pointing this out.

Reviewer #5 (Remarks to the Author):

Review report: Zhang et al: Re-interpreting Asian speleothem Isotope records: the hidden role of Atmospheric updraft.

I am reviewing the manuscript by Zhang et al. for the second time. Previously, I recommended rejection of the manuscript despite the impressive synthesis of published d18O precipitation data across the Asian domain presented in the previous version of the manuscript. I base my recommendation then based on the implicit and incorrect assumption that d18O in precipitation is transferred directly to cave drip waters and then preserved in speleothem calcite. This has now been thoroughly corrected, and I would like to commend the authors for the effort. As the manuscript currently stands, I believe it has the potential to stimulate productive discussion about the mechanistic interpretation of d18O signals faithfully preserved in the Chinese speleothem archives (See Implications and Outlook). I therefore recommend publication of the manuscript pending minor revisions and I would like to commend the authors once again for the constructive engagement.

Re: We sincerely thank you for your highly constructive and encouraging comments, as well as for your careful re-evaluation of our manuscript. We greatly appreciate your recognition of the substantial revisions made in response to the previous round of review. In particular, your comments regarding cave processes were extremely valuable, as they made us recognize that we could not simply assume a direct transfer of $\delta^{18}O$ signals from precipitation to cave drip water and subsequently to speleothem calcite.

Following your suggestions, we substantially revised the manuscript and removed the oversimplified discussion of speleothem $\delta^{18}O$ processes. We agree that these revisions have significantly improved the focus and rigor of the study, allowing the manuscript to concentrate more clearly on the role of atmospheric updraft in shaping precipitation isotope variability. We are also encouraged by your view that the revised manuscript may stimulate productive discussion regarding the mechanistic interpretation of $\delta^{18}O$ signals preserved in Asian speleothem archives.

In this round of revision, we have carefully addressed all remaining comments and further improved the manuscript accordingly. Once again, we sincerely appreciate your thoughtful and professional feedback throughout the review process, which has greatly strengthened the quality and scientific rigor of our work.

Minor comments (general)

Line 82-83:

Previous studies have widely applied Asian $\delta^{18}O_s$ records as a proxy for monsoon precipitation and/or monsoon intensity7,8,20-25.

To improve clarity, I suggest you divide the references: monsoon precipitation (Refs) and monsoon intensity (Refs). Note that monsoon precipitation and monsoon intensity describe related but still different aspects of the monsoon system even if the two terms are used interchangeably in the literature.

Re: Thank you very much for this helpful suggestion. We agree that monsoon precipitation and monsoon intensity describe related but distinct aspects of the monsoon system, and that separating the references improves the clarity and precision of the statement.

Following your suggestion, we revised the sentence to: "*Previous studies have widely applied Asian $\delta^{18}O_s$ records as a proxy for monsoon precipitation*[20-22] *and/or monsoon intensity*[8,9,23-25]*.*"

Please see L82–83 in the revised manuscript.

Line 83-85: Similarly, I suggest dividing the references according to archive type for clarity and to make it easier to access the cited references, particularly for readers outside the field. Please also consider doing the same for the rest of the text.

Re: Thank you very much for this helpful suggestion. We agree that separating the references by archive type improves clarity and makes the cited literature easier to follow, especially for readers outside the field.

Following your suggestion, we revised the sentence to: "*However, the interpretation of Asian $\delta^{18}O_s$ records remains strongly debated, initially motivated by discrepancies between speleothem-based reconstructions[8] and marine-based monsoon proxies[26-28].*"

Please see L83–L85 in the revised manuscript.

We have also applied this approach elsewhere in the manuscript where appropriate, including L75–L76, L163–L165 and L354–L355 in the revised manuscript.

Line 168: Rephrase (repetitive):

Considering that atmospheric updraft cannot be ignored

Understanding how atmospheric updraft influences $\delta^{18}O_p$ changes at precipitation sites is important.

Re: Thank you for pointing this out. We agree that the original sentence was repetitive and could be stated more directly.

Following your suggestion, we revised the sentence from:

"Considering that atmospheric updraft cannot be ignored, it is important to understand how it affects $\delta^{18}O_p$ changes at precipitation sites"

to:

"*Understanding how atmospheric updraft influences $\delta^{18}O_p$ at precipitation sites is important.*"

Please see L167–L168 in the revised manuscript.

Line 247: Rephrase (retain some nuance but still reduce adjectives)

Unfortunately, the results show that correlations change over time from weak positive, weak negative, and then to strong negative (Fig. 3c), reflecting the westward expansion or migration of low $\delta^{18}O_p$ from the pre-monsoon to monsoon seasons.

The results show that correlations change over time from positive to negative (Fig. 3c),

reflecting the westward expansion or migration of low $\delta^{18}O_p$ from the pre-monsoon to monsoon seasons.

Re: Thank you very much for this helpful suggestion. We agree that the original wording used unnecessary adjectives and that the sentence could be made clearer and more concise while retaining the intended meaning.

Following your suggestion, we revised the sentence from:

"Unfortunately, the results show that correlations change over time from weak positive, weak negative, and then to strong negative (Fig. 3c), reflecting the westward expansion or migration of low $\delta^{18}O_p$ from the pre-monsoon to monsoon seasons"

to:

"*The results show that correlations change over time from "positive" to negative (Fig. 4a-d, 4i-l), reflecting the westward expansion or migration of low* $\delta^{18}O_p$ *from the pre-monsoon to monsoon seasons.*"

Please see L252–L255 in the revised manuscript.

Line 259-263: Please add references for each.

Unlike other monsoon regions (e.g., Africa) (e.g., refs), precipitation in the Asian monsoon region is influenced not only by monsoonal circulation but also by the combined effects of the Intertropical Convergence Zone (ITCZ) [ref], the Indo-Pacific Warm Pool (IPWP) (e.g., refs), and the Western Pacific Subtropical High (WPSH) (e.g., refs).

Re: Thank you very much for this helpful suggestion. We agree that adding references for each climate driver improves the clarity and support of this statement.

Following your suggestion, we revised the sentence from:

"Unlike other monsoon regions (e.g., Africa), precipitation in the Asian monsoon region is influenced not only by monsoonal circulation but also by the combined

effects of the Intertropical Convergence Zone (ITCZ), the Indo-Pacific Warm Pool (IPWP), and the western Pacific subtropical high (WPSH)"

to

"*In the Asian monsoon region, precipitation is influenced not only by monsoonal circulation but also by the combined effects of the Intertropical Convergence Zone (ITCZ)*[70,71]*, the Indo-Pacific Warm Pool (IPWP)*[72,73]*, and the western Pacific subtropical high (WPSH)*[74-77]."

Please see L275–L278 in the revised manuscript.

References:

70. Waliser, D. E. & Gautier C. A satellite-derived climatology of the ITCZ. *J. Climate* **6**, 2162–2174 (1993).

71. Berry, G. & Reeder, M. J. Objective identification of the intertropical convergence zone: Climatology and trends from the ERA-Interim. *J. Climate* **27**, 1894–1909 (2014).

72. Basconcillo, J., Moon, I. J., Wang, B. & Mistry, M. Possible influence of the warm pool ITCZ on compound climate extremes during the boreal summer. *Environ*. *Res*. *Lett*. **16**, 114039 (2021).

73. Graham, N. E. & Barnett, T, P. Sea surface temperature, surface wind divergence, and convection over tropical oceans. *Science* **238**, 657–659 (1987).

74. Lau, W. K. M. & Li, M. T. The monsoon over East Asia and its global association-a survey. *Bull. Am. Meteor. Soc*. **65**, 116–125 (1984).

75. Chung, P. H., Sui, C. H., & Li T. Interannual relationships between the tropical sea surface temperature and summertime subtropical anticyclone over the western

North Pacific. *J. Geophys. Res. Atmos.* **116**, D13111 (2011).

76. Huang, Y., Wang, B., Li, X. & Wang, H. Changes in the influence of the western Pacific subtropical high on Asian summer monsoon rainfall in the late 1990s. *Clim. Dyn.* **51**, 443–455 (2018).

77. Li, S. et al. The interdecadal changes of the relationship between May-June and July-August NWPSH and their physical mechanisms. *Geophys. Res. Lett.* **51**, e2024GL111289 (2024).

Reviewer #6 (Remarks to the Author):

In this manuscript, Zhang and co-authors provide novel perspectives into the importance of atmospheric updraft in explaining water isotope variability of precipitation ($\delta^{18}O$) in the Asian monsoon region. In particular, they utilized $\delta^{18}O$ rain gauge data (GNIP) along with atmospheric reanalysis/observations to illustrate how the seasonal evolution of $\delta^{18}O_p$ in the Asian monsoon sector can best be explained by the seasonal cycle of updraft circulation. The authors argue that updraft dynamics explains the local minima in $\delta^{18}O_p$ in the central Asian Monsoon sector during the summer monsoon season, which would not be expected from a purely large-scale advection rainout process, which should always show more depleted values downstream in East Asia. Notably, these findings have important implications for our interpretation of $\delta^{18}O$ of calcite records and our understanding of past hydroclimate variability.

This is my first time reviewing the manuscript although I recognize that the authors

have addressed a previous round of comments and made significant revisions in response to them. Overall, I think the manuscript presents important contributions to the field and would be well suited for eventual publication in a high-impact journal such as Nature Communications. I do have several comments (2 major and a few minor) that I believe should be addressed prior to publication that will improve the clarity and logic of the manuscript.

Re: We sincerely thank you for your positive and encouraging assessment of our manuscript. We greatly appreciate your recognition of the novelty and potential significance of our study, particularly regarding the role of atmospheric updraft in shaping precipitation $\delta^{18}O$ variability across the Asian monsoon region and its implications for interpreting paleoclimate archives.

We also thank you for your constructive major and minor comments, which have helped us further improve the clarity, logic, and robustness of the manuscript. We have carefully addressed all the concerns you raised and revised the manuscript accordingly, as detailed below.

Major comments:

1. Local minima of $\delta^{18}O_p$: The authors present a detailed discussion of how atmospheric updrafts can lead to local rainout effects that increasingly deplete the oxygen isotopes of the residual moisture, thus leading to the decreasing $\delta^{18}O_p$ in regions of high updrafts. However, I am a little confused as to how this $\delta^{18}O_p$ increases once again further downstream. Take Figure 2g as an example. I understand how there is a decrease in $\delta^{18}O_p$ around Baoshan due to high updrafts at this longitude (Figure 3b, August), but why does the $\delta^{18}O_p$ increase again further east (downstream) if the residual moisture of the air parcel that arrives here has already been depleted from the updrafts? Is because the East Asian $\delta^{18}O_p$ is somewhat Pacific sourced, replenishing its $\delta^{18}O_p$? To me, if updrafts are the only/primary factor controlling $\delta^{18}O_p$, I would expect not a "V" shape in $\delta^{18}O_p$, but an "L" shape where the angle corresponds to where updrafts occur. Perhaps I am missing something?

Re: Thank you very much for pointing out that non-intuitive feature in Figure 2g of the previous version, now Fig. 2a in the revised manuscript: $\delta^{18}O_p$ decreases near Baoshan, where strong updraft occurs, but increases again farther east, downstream of this region. We really appreciate your attention to detail, and you're right to highlight it. We agree that if progressive depletion of advected vapor by the horizontal "rainout effect" were the dominant control, one would expect a monotonically decreasing pattern along the transport pathway, that is, an L-shaped or "↘" trend. Instead, our data show a V-shaped pattern, which is a central result of this study.

The key to this pattern is the spatial migration of strong atmospheric updraft. During the pre-monsoon season, enhanced updraft is mainly located in the eastern part of the transect. As the ITCZ gradually moves northward and expands westward, together with seasonal changes in the IPWP and WPSH, the updraft center also expands and migrates westward. By August, the strongest updraft has shifted from the east toward the middle and western part of the transect, near Baoshan and farther west. This westward migration explains why $\delta^{18}O_p$ increases again at the eastern, downstream sites in August: the strong local updraft that previously lowered $\delta^{18}O_p$ has moved away from the eastern sites and is now centered farther west. As a result, the eastern sites experience relatively weaker local updraft, allowing $\delta^{18}O_p$ values to rise.

In other words, the $\delta^{18}O_p$ pattern along this transect is controlled primarily by the intensity and position of local atmospheric updraft, rather than by cumulative rainout along a horizontal advection pathway alone. To make this distinction clearer, we now explicitly separate two mechanisms in the revised manuscript:

- Rainout effect_{ad}, which occurs during horizontal advective vapor transport; and

- Rainout effect_{au}, which occurs within local atmospheric updraft.

We have added text to clarify this mechanism in the revised manuscript, including: "*Clearly, $\delta^{18}O_p$ in the East Asian monsoon region is spatially decoupled from the monotonically decreasing pattern (an expected "L-shaped" or "↘" trend) that would*

result from the "rainout effect$_{ad}$" occurring along South Asian monsoon moisture advection (see Supplementary Text 2 for details)."

Please see L147–L150 in the revised manuscript.

We also added:

"*Consequently, the enhanced atmospheric updraft migrates to the middle of the transect, causing $\delta^{18}O_p$ to decrease in the middle. In the east, where atmospheric updraft is relatively weaker, $\delta^{18}O_p$ is relatively high, explaining the V-shaped $\delta^{18}O_p$ pattern during the monsoon season rather than a monotonically decreasing L-shaped one (or " ↘ " trend) driven by the "rainout effect$_{ad}$.*"

Please see L206–L211 in the revised manuscript.

We acknowledge that East Asian $\delta^{18}O_p$ is, to some extent, influenced by moisture transport from the Pacific Ocean. However, numerous studies indicate that the dominant moisture source for East Asia is the Indian Ocean, and that even when Pacific moisture contributes, it does not play a leading role. In addition, if the $\delta^{18}O_p$ values at sites in the East Asian monsoon region were primarily controlled by moisture transport from the Pacific Ocean, they should be able to capture the signals of East Asian monsoon or Western North Pacific monsoon intensity. However, at our eastern sites — including Hong Kong, Guangzhou, and Kunming — the correlations between $\delta^{18}O_p$ and the East Asian Monsoon Index (EAMI) or the Western North Pacific Monsoon Index (WNPMI) are considerably weaker than those between $\delta^{18}O_p$ and local outgoing longwave radiation (OLR), both on monthly (Table S3) and annual (Table S4) scales. Therefore, we argue that the contribution of Pacific moisture transport to East Asian $\delta^{18}O_p$ is less significant than that of atmospheric updraft. We have provided a detailed clarification in Text S4 (Supporting Information) to address this issue.

We thank you again for this insightful observation. It helped us clarify one of the most important mechanistic distinctions in the manuscript and substantially improved the

explanation of the V-shaped $\delta^{18}O_p$ pattern.

2. Quantifying updraft contribution to $\delta^{18}O_p$ variability: I am not entirely convinced by the quantification analysis in Figure 4. Figure 4a,b show that a large fraction of $\delta^{18}O_p$ variability cannot be explained by local updrafts (correlation coefficient of only 0.4-0.48), suggesting other factors are important. The authors then compute the slopes of both OLR and $\delta^{18}O_p$ as an east-west transect across the GNIP stations and correlate them to illustrate high correlation (r=0.90) and thus strong influence of updrafts. However, I think that this correlation of monthly slopes does not properly illustrate the noise in their data, as clearly is present from Figure 4a,b. There is no representation of how well each of these monthly slope values explain the data (no RSME of regressions, no significance testing), thus its unclear how well these slopes actually represent the variability. In my mind, the high correlation in Figure 4g demonstrates that qualitatively, the timing of slope changes are largely synchronized between OLR and $\delta^{18}O_p$, which we can see visually in Figure 2,3. But it does not provide a precise quantification or explained variance of the role of updrafts.

Re: Thank you very much for raising these important points. We fully agree with your assessment and appreciate your rigorous and constructive critique. We have addressed these issues as follows.

First, regarding the analysis shown in Fig. 4, we agree that it does not, in a strict sense, quantify the contribution of atmospheric updraft to $\delta^{18}O_p$ variability. Rather, it assesses the relationship between $\delta^{18}O_p$ and atmospheric updraft indicators. We have therefore revised the wording throughout the manuscript to avoid overstating this result. Specifically, we changed:

"We further quantified the influence…"

to:

"*We further assessed the influence…*":

Please see L226 in the revised manuscript. We also softened related statements elsewhere in the manuscript.

Second, regarding the moderate correlations shown in Fig. 4a, b (r = 0.40–0.48), we agree that a substantial fraction of $\delta^{18}O_p$ variability remains unexplained, indicating that other factors also contribute to precipitation isotope variability. Accordingly, we have toned down claims implying dominance by atmospheric updraft. For example, we changed "key control" to "*important control*" (L65), " substantial control" to "*notable control*" (L243), and "plays a key role" to "*plays a very important role*" (L246) in the revised manuscript.

Third, regarding the correlation of monthly slopes in Fig. 4g, we agree that the high correlation (r = 0.90) primarily indicates synchronized timing of slope changes between $\delta^{18}O_p$ and OLR, rather than a precise quantification of explained variance. Following your suggestion, we have added a new table (see Table R2 as follows) reporting the RMSE and significance test results for the regressions used to derive each monthly slope value. We acknowledge that some monthly regressions are not statistically significant, which may partly reflect the small number of sites used for individual monthly slope estimates. Nevertheless, the overall consistency in the timing and direction of slope changes, also evident in Figs. 2 and 3, supports our qualitative conclusion that atmospheric updraft exerts an important influence on $\delta^{18}O_p$ along the transect. Please see Table S5 in the revised Supplementary Information.

Table R2. Monthly slopes of $\delta^{18}O_p$ and OLR with associated regression statistics (slope, R^2, RMSE, and p-value).

month	$\delta^{18}O_p$_slope	$\delta^{18}O_p$_R^2	$\delta^{18}O_p$_RMSE	$\delta^{18}O_p$_p value	OLR_slope	OLR_R^2	OLR_RMSE	OLR_p value
February	-0.1361	0.3105	0.4055	0.194	-1.5857	0.4604	3.4335	0.094
March	**-0.2679**	0.776	0.2878	**0.009**	-2.4982	0.5569	4.4567	0.054
April	-0.0668	0.2593	0.2258	0.243	**-3.315**	0.5826	5.6118	**0.046**
May	-0.0018	0.00007	0.4380	0.986	**-2.7989**	0.593	4.6372	**0.043**
June	0.0886	0.2617	0.2540	0.3	0.9220	0.5573	1.4035	0.088
July	0.2693	0.3039	0.8150	0.2	**8.8086**	0.9431	4.3262	**< 0.001**
August	0.2494	0.1731	1.2490	0.305	**7.0470**	0.8911	5.6448	**< 0.001**
September	0.2230	0.0908	1.8219	0.431	**6.1948**	0.8402	6.9752	**0.001**

Note: R^2, variance explained by the linear trend; RMSE, root-mean-square error; p-value, significance of the slope, bold indicates $p < 0.05$.

Fourth, we would like to clarify why the correlations of r = 0.40–0.48 remain meaningful in this context. In the Asian monsoon region, $\delta^{18}O_p$ is influenced by multiple interacting factors, including moisture advection and rainout, precipitation amount, topography, and local atmospheric processes. Therefore, a significant correlation between $\delta^{18}O_p$ and OLR at this spatial scale is non-trivial. However, we have also examined the correlations with these other potential contributors. As shown in Tables 3 and 4, correlations between $\delta^{18}O_p$ and monsoon indices at several long-term stations are generally weaker than those between $\delta^{18}O_p$ and OLR. Moreover, the traditional monsoon advection/rainout interpretation cannot explain three key observations in our study: temporal decoupling, spatial decoupling reflected by the V-shaped rather than L-shaped or "↘" pattern, and systematically weaker correlations with monsoon indices.

Fifth, we further assessed alternative controls, particularly topography and precipitation amount. For topography, Fig. 4a–d and 4i–l show that altitude effects are limited and cannot explain the observed seasonal migration of low $\delta^{18}O_p$ values. For precipitation amount, following comments from Reviewer #3, we conducted additional analyses, including spatial-pattern analyses (Fig. 4e–h, 4m–p), correlation analyses (Supplementary Fig. 8a, b), and linear model comparisons (Supplementary Text 6). These analyses indicate that precipitation amount plays a secondary role

relative to atmospheric updraft. Notably, the limited “amount effect” may itself partly reflect co-variation with atmospheric updraft, as shown in Supplementary Fig. 8c, d.

Finally, we have added a dedicated discussion in the “Outlook” subsection to explicitly acknowledge that multiple factors likely act together along this transect, as suggested by the reviewer in the previous round.

We thank you again for this insightful comment. It helped us avoid overstating the statistical interpretation of Fig. 4 and substantially improved the rigor, balance, and clarity of our manuscript

Minor comments:

1. I think Figure 1 would look nicer if panel b and c were vertically stacked, that way the readers could easily see the differences in seasonal timing along the x axis between the east Asian and south Asian sites.

Re: Thank you very much for this helpful suggestion. We agree that vertically stacking panels b and c makes the seasonal timing differences in precipitation isotope variations between the East Asian and South Asian sites easier to compare along the x-axis.

Following your suggestion, we have revised Fig. 1 by vertically stacking panels b and c. Additionally, following the suggestion of Prof. Lonnie, we illustrate the seasonal variations of $\delta^{18}O_p$ at the 34 ancillary sites in panels b and c. The updated figure is included in the revised manuscript, and the revised layout is also shown in Fig. R3 below.

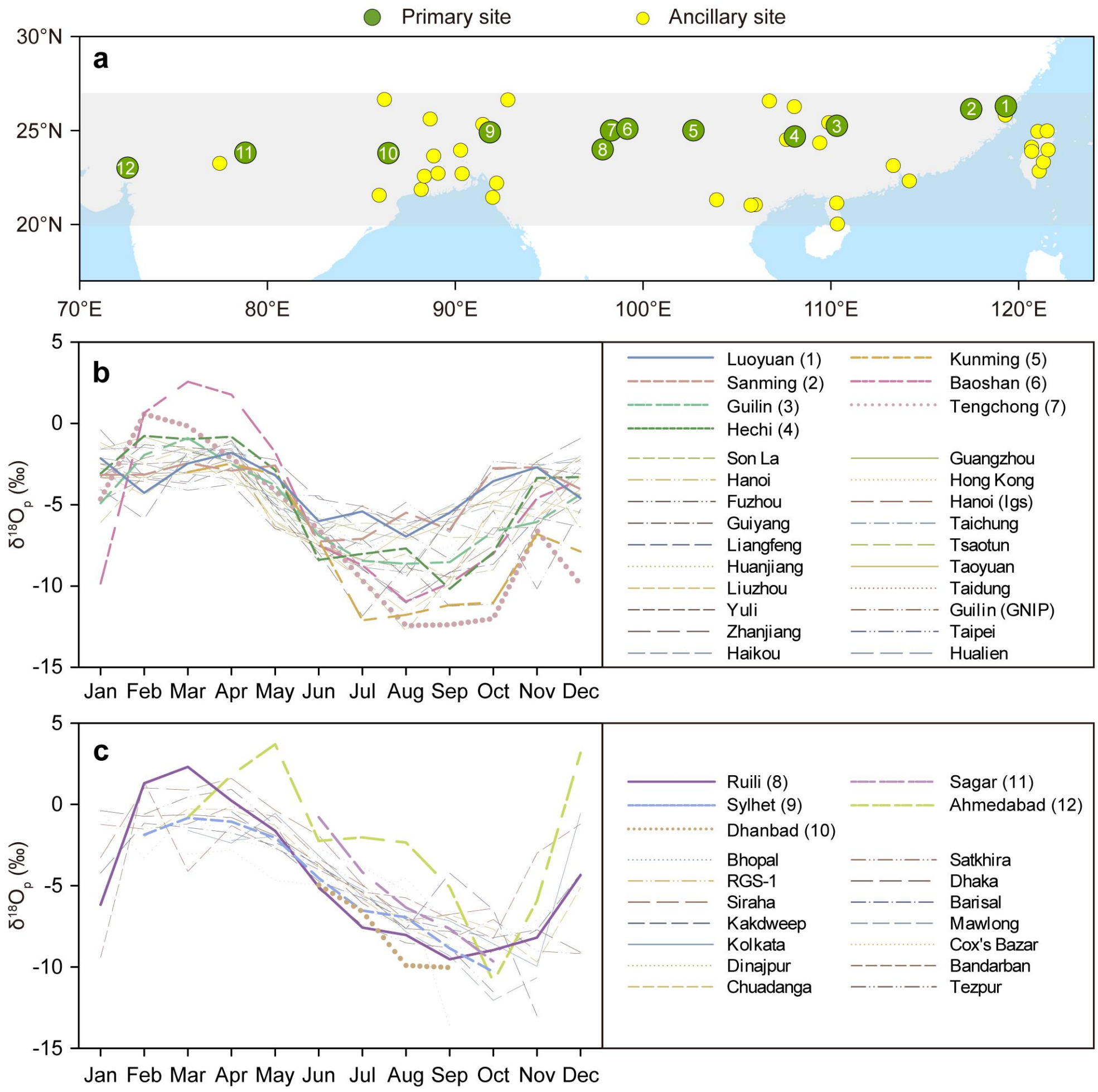

Figure R3. Sampling sites in the study region and seasonal variations of observed multi-year mean $\delta^{18}O_p$ at 46 precipitation sites across the Asian monsoon region. **a** Precipitation sampling sites, green dots indicate the 12 primary sites selected for this study (Supplementary Table 1), while yellow dots represent the 34 ancillary sites (Supplementary Table 2). **b, c** Seasonal variations of $\delta^{18}O_p$ at 46 precipitation sites in the East Asian (**b**) and South Asian (**c**) monsoon regions. The 12 primary sites are marked by thick lines and the 34 ancillary sites by thin lines. Numbers in the brackets in **b** and **c** correspond to the green dots in **a**. $\delta^{18}O_p$ values are expressed as per mil (‰) deviations relative to VSMOW or VSMOW2.

2. I found myself hopping between Figure 2 and Figure 3 to make the connections between $\delta^{18}O_p$ and the atmospheric properties from a seasonal cycle perspective. Is there a way to make these two plots more connected, either by adding the $\delta^{18}O_p$ to Figure 3 or otherwise? Also I found the column c to be a little distracting, given that it

is there to primarily make a negative point (perhaps this was in response to another reviewer's comment).

Re: Thank you very much for this helpful suggestion. We apologize that the previous figure layout made it difficult to follow the seasonal relationship between $\delta^{18}O_p$ variations and atmospheric updraft. After carefully re-examining Figs. 2 and 3, we agree that readers would need to move back and forth between the two figures to connect the isotope and atmospheric patterns.

Following your suggestion, we have merged the original Fig. 2 with the relevant atmospheric-updraft information from Fig. 3 to create a single integrated figure (shown as Fig. R4 below). This revised figure allows the seasonal evolution of $\delta^{18}O_p$, OLR, and vertical velocity to be compared more directly.

At the same time, we moved the original Fig. 3c out of this figure because, as you noted, it mainly served to address an alternative explanation related to altitude. We have now combined this analysis with the precipitation-amount analysis in a separate figure (shown as Fig. R5 below), in response to comments from other reviewers. With these revisions, the manuscript now uses one figure to illustrate the relationship between $\delta^{18}O_p$ and atmospheric updraft, and a separate figure to evaluate the limitations of other potential controls.

Please see the new Fig. 2 and Fig. 4 in the revised manuscript.

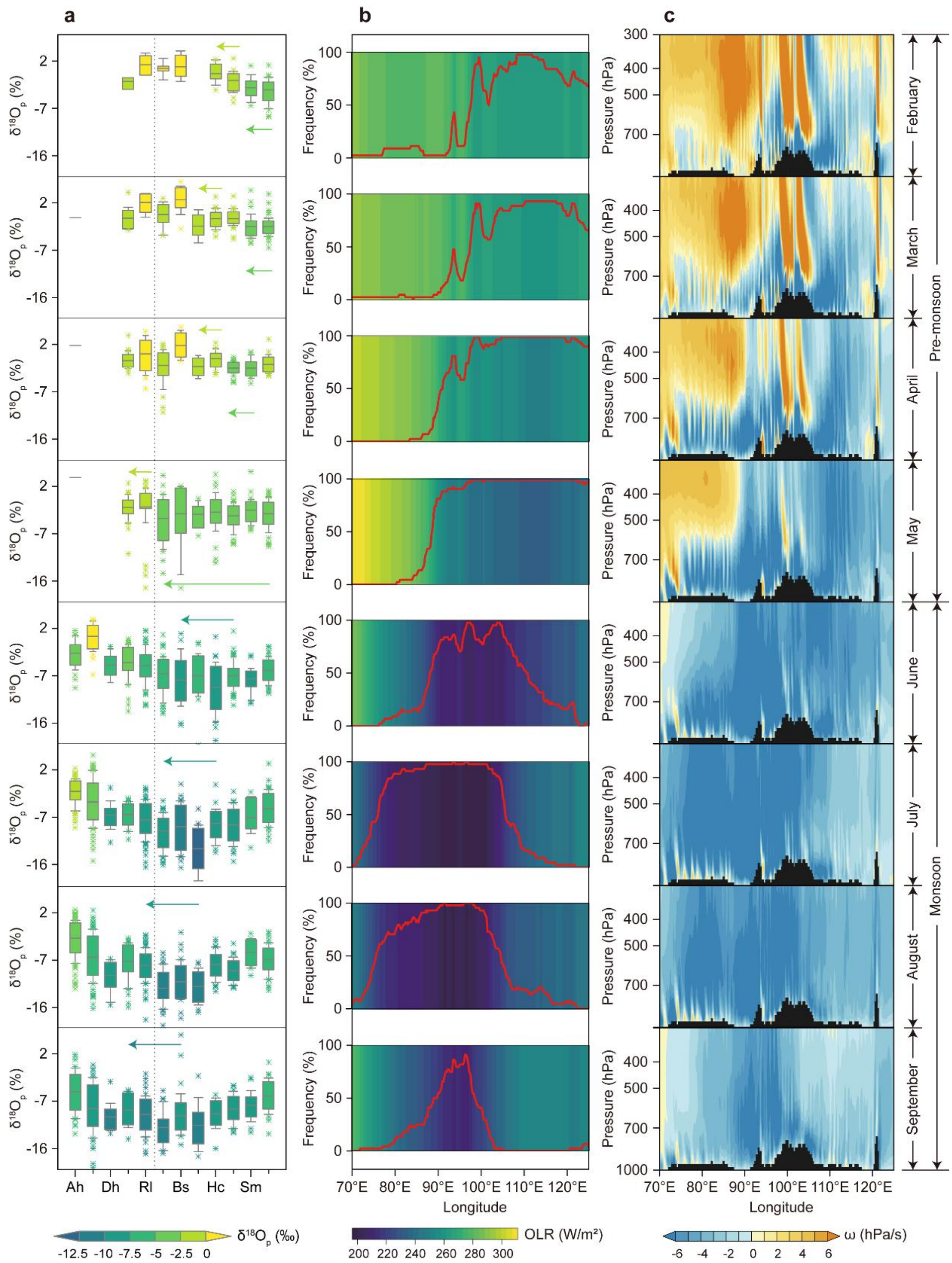

Figure R4. Spatial distributions of $\delta^{18}O_p$ at the 12 primary sites and seasonal changes of atmospheric updraft along the transect during the pre-monsoon and monsoon seasons over 1979–2022. a Spatial distributions of $\delta^{18}O_p$. Abbreviations: Ah (Ahmedabad), Dh (Dhanbad), Rl (Ruili), Bs (Baoshan), Hc (Hechi), and Sm (Sanming). In each box plot, the box represents the 20th–80th percentile range, whiskers extend to the 10th–90th percentiles, horizontal lines inside the boxes

indicate the mean, and stars represent outliers. Horizontal arrows highlight the westward extension of the low $\delta^{18}O_p$ zone. Dotted lines mark the dividing line of the East Asian and South Asian monsoon regions. $\delta^{18}O_p$ values are expressed as per mil (‰) deviations relative to VSMOW or VSMOW2. **b** Seasonal variations in outgoing longwave radiation (OLR, shading) and the frequencies of relatively low OLR values (red line) along the transect. Lower OLR values indicate stronger convection. **c** Vertical profiles of meridional mean vertical velocity (ω), averaged over 20–27° N. Negative ω values indicate upward motion (convection), while positive values indicate downward motion (subsidence).

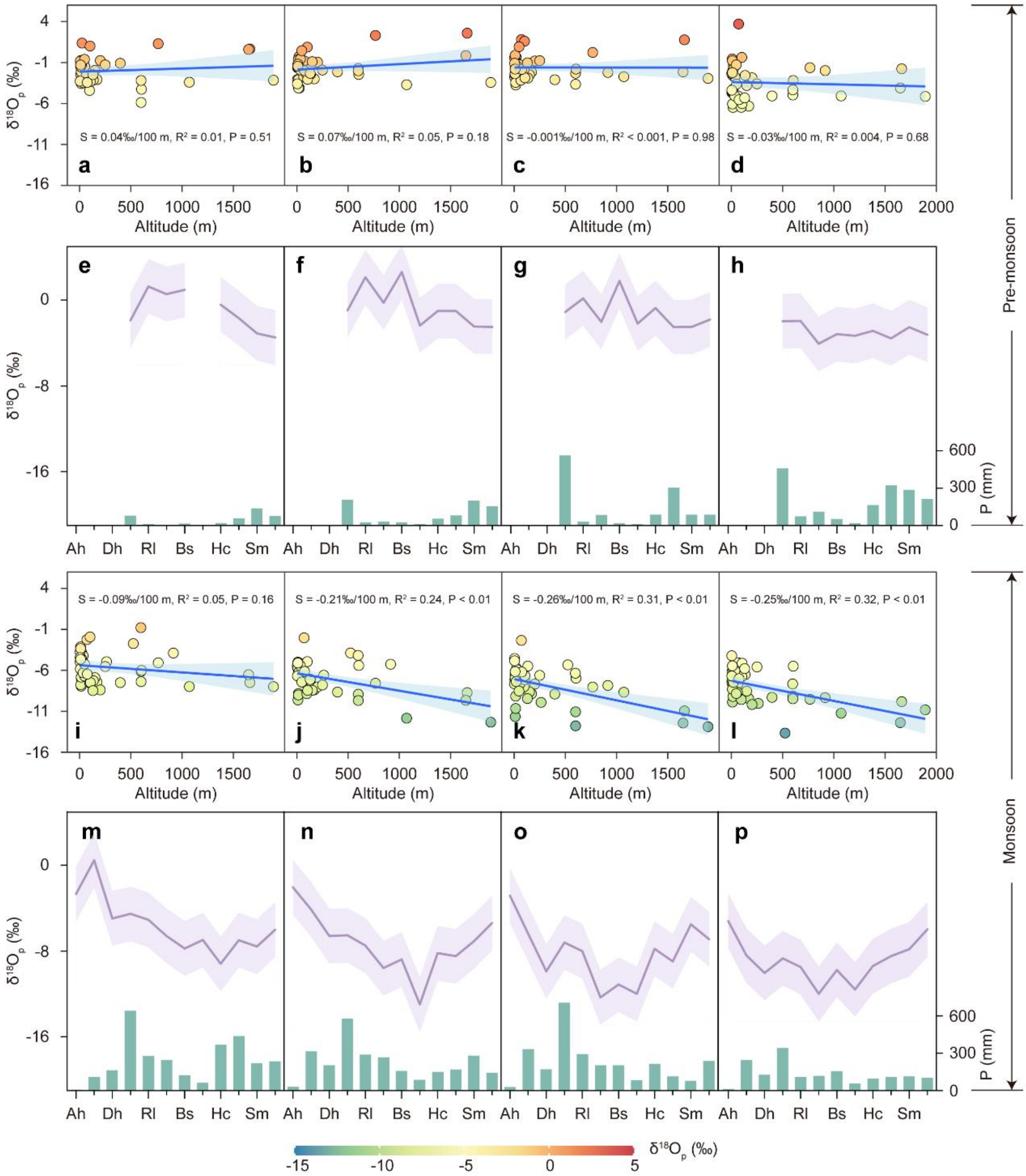

Figure R5. Seasonal variations in $\delta^{18}O_p$–altitude correlations at the 46 sites and spatial changes of $\delta^{18}O_p$ and precipitation amount at the 12 primary sites. a–d, i–l Linear regressions of $\delta^{18}O_p$ at the 46 sites across the study region against altitude, fitted for each month. **e–h**, **m–p** Multi-year mean monthly $\delta^{18}O_p$ (line) with ±1 standard deviation (shading) and precipitation amount distributions during the pre-monsoon and monsoon seasons: February (**e**), March (**f**), April (**g**), May (**h**), June (**m**), July (**n**), August (**o**), and September (**p**). Abbreviations: Ah (Ahmedabad), Dh (Dhanbad), Rl (Ruili), Bs (Baoshan), Hc (Hechi), and Sm (Sanming). $\delta^{18}O_p$ values are

expressed as per mil (‰) deviations relative to VSMOW or VSMOW2.

3. I think Figure 5 is not that useful for illustrating the influences of ITCZ, IPWP, and WPSH on updrafts (which I believe is the author's goal). I think something a bit more quantitative such as showing actual updraft velocities in specific regions versus ITCZ location or otherwise would be more helpful to establish the dynamical links.

Re: Thank you very much for pointing this out. We agree that the original Fig. 5 did not effectively illustrate the influence of the ITCZ, IPWP, and WPSH on atmospheric updraft, which was the intended purpose of the figure. Accordingly, we have moved this figure to the Supplementary Information, where it now appears as Fig. S9.

Following your thoughtful suggestion, we have also conducted additional quantitative analyses to better demonstrate the dynamical linkage between the ITCZ and atmospheric updraft. Specifically, we divided the transect into three subregions: western, central, and eastern. For each subregion, we calculated the monthly latitude of the northern boundary of the ITCZ within the corresponding longitude range and examined its relationship with OLR in that subregion. These analyses reveal significant correlations between the northern boundary of the ITCZ and OLR, thereby providing quantitative support for a dynamical link between ITCZ migration and atmospheric updraft along the transect. These results are shown in the new Fig.5 in the revised manuscript, with additional supporting information provided in the new Fig. S9 and Fig. S10 in the revised Supplementary Information.

To clarify the methodology, we have added the following paragraph to the Materials and Methods section:

"*To quantify the location of the ITCZ and its dynamical linkage with atmospheric updraft, we first divided the transect into three subregions: Western (70–95° E), Central (95–105° E), and Eastern (105–125° E). Using the empirical probability density functions (PDFs) derived from the multivariate probabilistic framework described above, we extracted the monthly northern boundary of the ITCZ within the*

longitudinal range of each subregion over 1979–2022. For a given month (e.g., January 1979), the procedure was as follows: for each longitudinal grid point within a subregion, we scanned southward from 27° N to 27° S and recorded the latitude where the PDF value first exceeded 0 as the ITCZ's northern boundary for that longitude and month. After extracting the northern boundaries for all longitudes that had a valid boundary in that month, we calculated the arithmetic mean of these latitudes across all longitudes within each subregion. This mean constitutes the ITCZ's northern boundary index for that subregion for that month. Repeating this procedure for every month from January 1979 to December 2022 yielded a continuous time series of the ITCZ's northern boundary for each of the three subregions over the entire period. Finally, we calculated the correlation between OLR within each subregion and the corresponding northern boundary of the ITCZ. The significant positive correlation between the ITCZ's northern boundary and OLR (Fig. 5i–k) supports a robust dynamical linkage between the ITCZ and atmospheric updraft along this transect."

Please see L672–L691 in the revised manuscript.

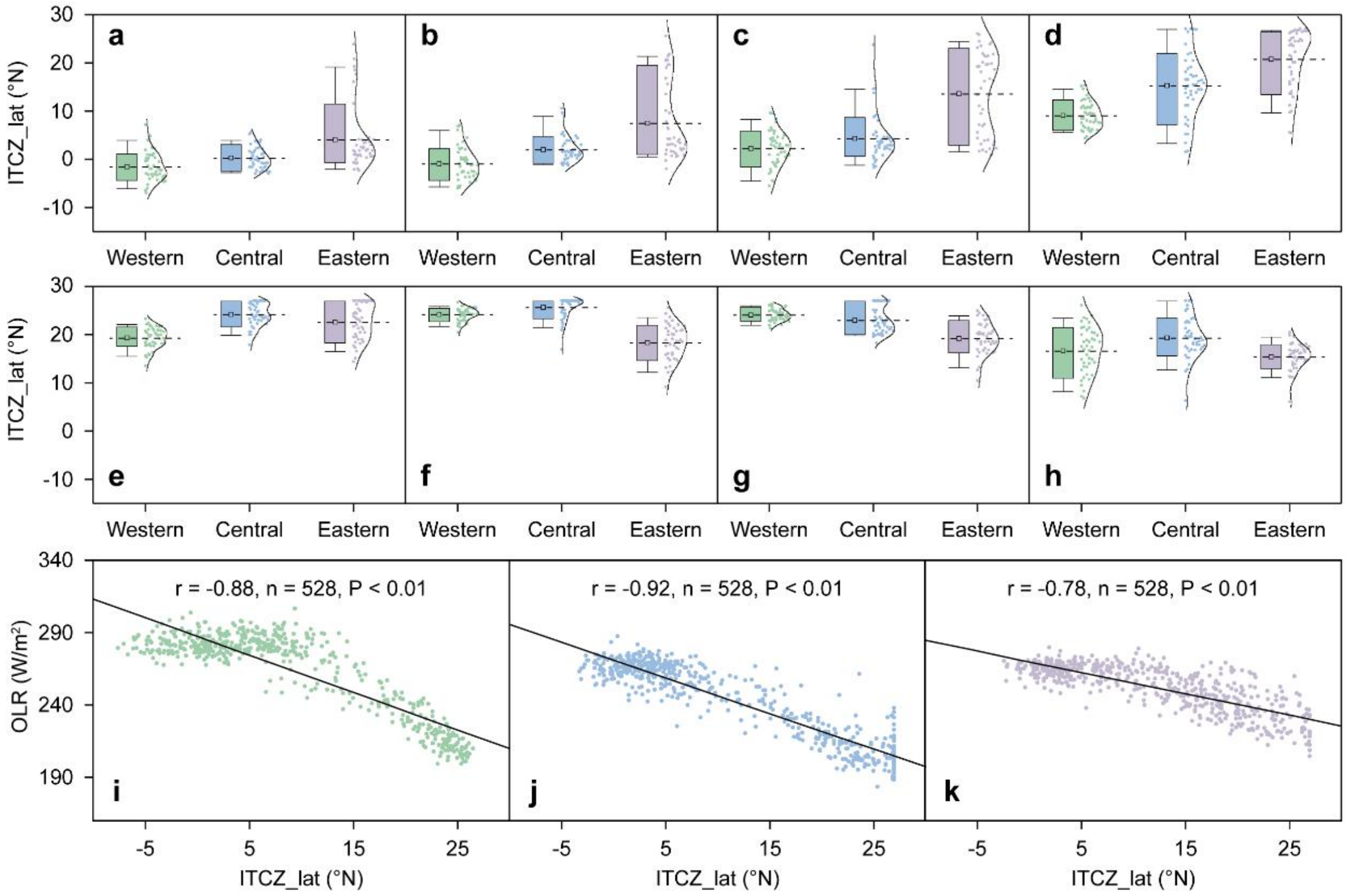

Figure R6. Intertropical Convergence Zone (ITCZ) northern boundary location within the respective longitude ranges of three subregions, and correlations between outgoing longwave radiation (OLR) in these subregions and the corresponding ITCZ northern boundary location during 1979–2022. a–d ITCZ northern boundary location (ITCZ_lat) during the pre-monsoon season: February (**a**), March (**b**), April (**c**), and May (**d**). **e–h** ITCZ northern boundary location during the monsoon season: June (**e**), July (**f**), August (**g**), and September (**h**). Western, Central, and Eastern represent the three subregions: Western (70–95° E), Central (95–105° E), and Eastern (105–125° E). **a–h** In each box plot, the box represents the 15th–85th percentile range, and whiskers extend to the 5th–95th percentiles. The mean is shown as a hollow square inside each box and as a horizontal dashed line that crosses the box, the right-hand scatterplot, and the kernel density curve. The kernel density curve is estimated using a smoothing kernel to visualize the distribution shape. **i–k** Correlations between OLR in the three subregions and ITCZ northern boundary location (1979–2022, all months combined): Western (**i**), Central (**j**), and Eastern (**k**).

4. As someone more familiar with tropical climate, I'm used to thinking of OLR as reflective of cloud cover and heavy rainfall, not necessarily updraft strength (although

I recognize that the two are related). Could the authors include some more analysis (like correlations) that demonstrate how OLR is a robust proxy for updrafts (vertical velocities)?

Re: Thank you very much for raising this important point. We agree that OLR is more commonly interpreted as an indicator of cloud-top temperature, deep convection, and heavy rainfall, and that its use as a proxy for atmospheric updraft should be explicitly supported in our study.

Following your suggestion, we conducted additional correlation analyses between OLR and vertical velocity at 300, 400, and 500 hPa. The results show significant positive correlations between OLR and vertical velocity at all three levels (Fig. R7). Because negative vertical velocity indicates upward motion, this positive relationship means that lower OLR corresponds to stronger upward motion, supporting the use of OLR as an effective indicator of atmospheric updraft intensity in this region.

To address this point in the revised manuscript, we added a new section and figure to the Supplementary Information:

"***Text S3. Correlations between OLR and vertical velocity***

To test whether OLR is a robust proxy for atmospheric updraft intensity, we examined correlations between OLR and vertical velocity. In this region, OLR exhibits significant positive correlations with vertical velocity at 300, 400, and 500 hPa (Supplementary Fig. 3). Because negative vertical velocity indicates upward motion, these positive correlations indicate that lower OLR corresponds to stronger atmospheric updraft. These results confirm that OLR can be used as an effective indicator of atmospheric updraft intensity in this study region."

Please see the new Text S3 and the new Fig. S3 in the revised Supplementary

Information.

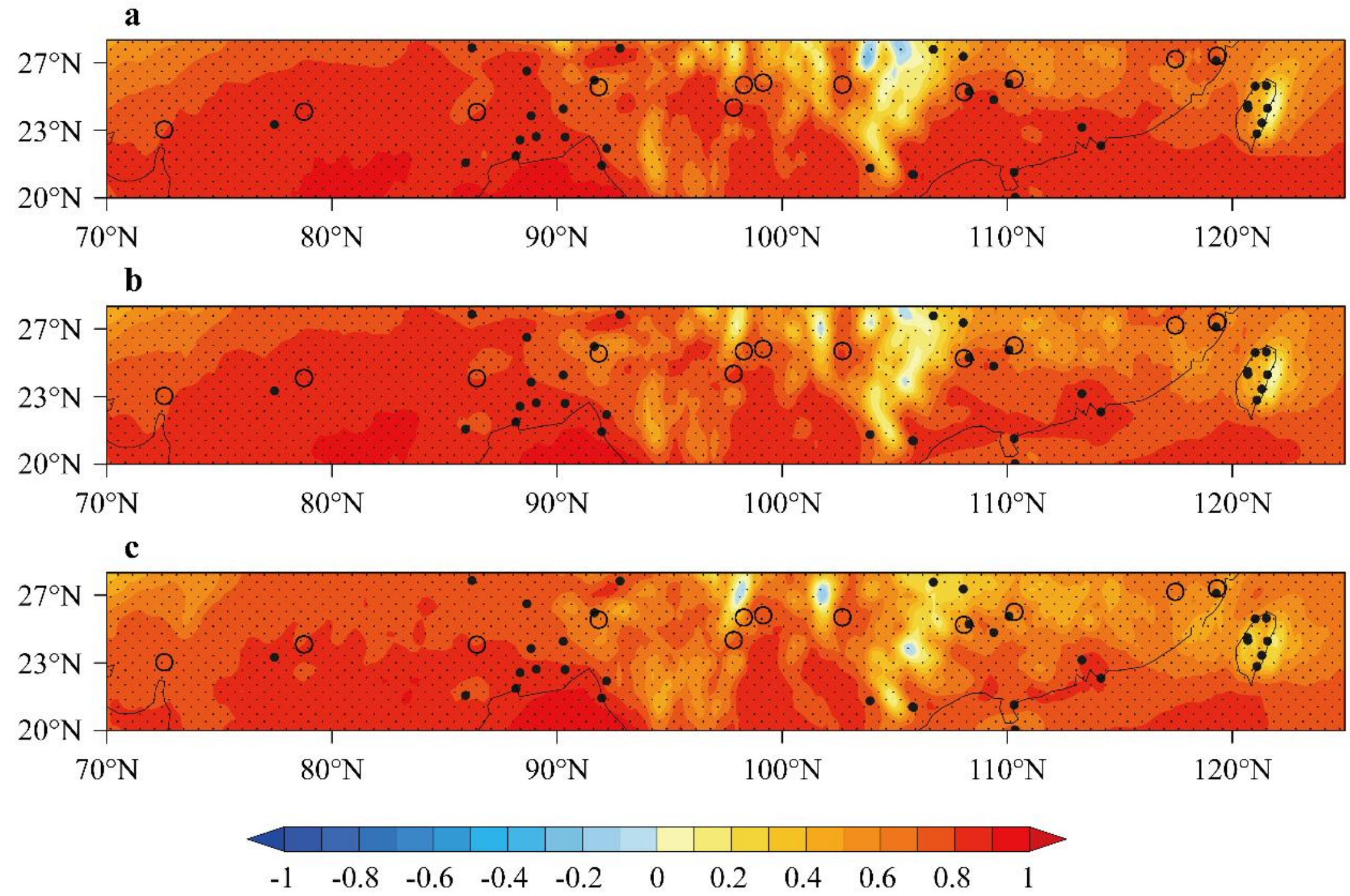

Figure R7. Spatial correlations between monthly vertical velocity (ω) and the corresponding outgoing longwave radiation (OLR) from 1979 to 2022. a Vertical velocity at 300 hPa. **b** Vertical velocity at 400 hPa. **c** Vertical velocity at 500 hPa. Open black circles indicate the 12 primary sites selected for this study (Supplementary Table 1), while black dots represent the 34 ancillary sites (Supplementary Table 2).

Response to Reviewers

(Reviewers' statements in black, and our response in blue)

Line232-233, -the line 232-233 in the previous manuscript.

L240–L245, -the lines 240-245 in the revised manuscript.

Fig. 1, -the Figure 1 in the revised manuscript.

Figure R1, -the Figure 1 in the response.

REVIEWER COMMENTS

Reviewer #3 (Remarks to the Author):

The revised manuscript is very well written and the authors have done a wonderful job in answering my concerns throughout the whole review processes.

I maintain to hold a little doubt, as the authors do show that the two are correlated and therefore not statistically distinguishable. The absence of a major difference between AIC values of the two models illustrates that inclusion of both does not improve the model, simply because they represent the same axis of variation. Nevertheless, I believe the authors have taken a good effort to try to show that precipitation amount does not explain the whole variability seen across the transect, and that atmospheric updraft motion is the primary control. I expect that vertical updraft is likely the primary mover, with rain amount acting as a secondary component of the same process. Whether this acts as a new understanding of the mechanism of the traditional amount effect, or is an independent effect, is a semantic debated, and the minor controls on rainfall $\delta^{18}O$ may be revealed over future research.

I believe this manuscript offers an interesting insight into spatial patterns of rainfall $\delta^{18}O$ in Asia, and I recommend the manuscript for publication following very minor corrections attached below.

Re: We sincerely thank you for your thoughtful and constructive comments throughout the review process. We greatly appreciate the time and care you have devoted to evaluating our manuscript, and we are particularly grateful for your positive assessment and recommendation for publication. Your comments have helped us sharpen both the presentation and interpretation of our results. We have carefully addressed the remaining minor points, as detailed below.

We agree with your assessment that precipitation amount and atmospheric updraft are closely coupled and therefore difficult to distinguish statistically. Because both variables capture related aspects of the same physical process, including precipitation amount in addition to OLR does not substantially improve the model, as indicated by the similar AIC values of Model 1 (OLR only) and Model 2 (OLR and precipitation amount).

We also agree with your interpretation that atmospheric updraft is likely the primary driver, whereas precipitation amount represents a secondary expression of the same process. This interpretation is consistent with our spatial analyses and model comparisons, which show that atmospheric updraft provides the more direct explanation for the observed $\delta^{18}O_p$ patterns across the transect. At the same time, we acknowledge that the distinction between a revised physical interpretation of the traditional "amount effect" and an independent updraft-related mechanism is not yet fully resolved. We have therefore moderated our wording in the revised manuscript and now present atmospheric updraft as a key physical process underlying much of the observed relationship between precipitation amount and $\delta^{18}O_p$. We agree that the relative contributions of these closely linked controls should be examined further in future studies.

Minor corrections

Line 232-233: Please state whether these negative gradients are significant

Re: We thank you for pointing this out. We re-examined the results reported in Supplementary Table 5 and found that the negative gradient is statistically significant only in March ($p < 0.05$); the gradients in February, April, and May are not individually significant. Nevertheless, the gradient remains consistently negative throughout the pre-monsoon season. We have revised the manuscript to report the statistical significance of these monthly gradients explicitly. Please see Lines L240–L245 of the revised manuscript.

Line 236: Again, are these significant?

Re: Thank you for raising this point. We re-examined the results in Supplementary Table 5 and found that the positive gradient is not statistically significant for most individual monsoon-season months. However, the gradient remains consistently positive throughout the monsoon season, in contrast to the consistently negative gradients observed during the pre-monsoon season. We therefore emphasize the seasonal reversal in gradient direction rather than the significance of any single monthly estimate. The manuscript has been revised accordingly to report this distinction clearly. Please see L240–L245 of the revised manuscript.

Line 242: report p value with the correlation coefficient.

Re: Thank you for pointing this issue out. Following your suggestion, we added p value ($p < 0.01$) with the correlation coefficient. Please see L247 in the revised manuscript.

Line 253: Not sure why positive is in quotation marks. If it's not significant, there is no significant correlation and is therefore not quantifiably positive. The authors could revise this sentence from:

"The results show that correlations change over time from "positive" to negative (Fig. 4a-d, 4i-l)"…

To something like:

“The results show that correlation is time transgressive, with only significant negative correlations occurring in the monsoon seasons”….

Also note that figure 4 does not provide the months of the correlation plots.

Re: Thank you for this helpful comment. Indeed, we used quotation marks around “positive” because the correlation does not attain statistical significance. We concur that, in the absence of such significance, it cannot be quantitatively considered positive. Following your suggestion, we changed “The results show that correlations change over time from ‘positive’ to negative” to

“*The results show that correlation is time transgressive, with only significant negative correlations occurring in the monsoon seasons.*”

Please see L259–L260 in the revised manuscript.

As you pointed out, Figure 4 does not provide the months for the correlation plots. Following your suggestion, we have added the months in Fig. 4:

“*a–d, i–l Linear regressions of $\delta^{18}O_p$ at the 46 sites across the study region against altitude, fitted for each month: February (a), March (b), April (c), May (d), June (i), July (j), August (k), and September (l).*”

Please see L820–L821 in the revised manuscript.

Line 303: “Hence, the combined influence of the ITCZ, IPWP, and WPSH results in a significantly stronger atmospheric updraft in the East Asian monsoon region than in the South Asian monsoon region during the pre-monsoon season, particularly in May (Fig. 6a).” Is this a test for statistical significance, i.e., t-test of updated in east vs west? If not, “significant” should be replaced with “substantially”. If it is, please report p-values.

Re: Thank you for your helpful comment. We acknowledge that this observation has not been statistically tested; nevertheless, it is clearly evident from Figs. 2b and 2c

that during the pre-monsoon season, particularly in May, atmospheric updraft in the East Asian monsoon region is substantially stronger than that in the South Asian monsoon region, as reflected by the substantially lower OLR and vertical velocity (ω) values in the East Asian monsoon region. Following your suggestion, we changed "significantly" to "*substantially*" and cited Fig. 2b and 2c. Please see L311 in the revised manuscript.

Reviewer #6 (Remarks to the Author):

I would like to thank the authors for their extensive work in revising the manuscript in consideration of my previous round of comments. The paper has greatly improved in terms of clarity, robustness, and balance of results. I have only two remaining comments on Figure 5 as well as the discussion/outlook before recommending for publication.

Re: We sincerely thank you for your positive assessment of the revised manuscript. We greatly appreciate the time and care you have devoted to reviewing our work, as well as your constructive suggestions throughout the review process. Your previous comments—particularly those concerning figure organization and the need for additional quantitative analysis of the relationship between the ITCZ and atmospheric updraft—have substantially improved the clarity, balance, and scientific rigor of the manuscript. We have now carefully addressed your two remaining comments regarding Figure 5 and the Discussion and Outlook section. Our detailed point-by-point responses and the corresponding revisions are provided below.

Figure 5 analysis:

I appreciate the authors significant efforts in improving the discussion of the various mechanisms that control updrafts in the Asian monsoon region, especially with the

new Figure 5 which aims to be more quantitative.

While I believe that Figure 5i,j,k provide good quantitative support that ITCZ latitude shapes OLR (and by association updrafts) variability, I personally don't find Figure 5a-h that useful in this context. These 8 subplots mainly serve to illustrate the seasonal migration of the ITCZ in the three domains but don't provide much else. The text argues that the timing of these ITCZ positions contribute to various processes that increase updrafts (such as frontal lifting) but there is no demonstration of the timing of these circulation changes in the Figure 5 itself. I would recommend perhaps collapsing Figures 5a-h into a single subplot that visualizes the seasonal cycle of ITCZ migration, which should make room for additional analysis.

Furthermore, the main text which discusses Figure 5 argues for the influences of the IPWP and WPSH, but much of this analysis is buried in the supplement. I recognize the space constraints of Nature Communications but I think showing some metric of these two features (IPWP and WPSH) and how they shape updrafts in the Asian monsoon in the Main Text would be valuable.

Re: We thank you for this helpful and constructive comment. We agree that the previous Figure 5a–h primarily illustrated the seasonal migration of the ITCZ but provided limited quantitative information on the timing of the associated circulation changes. We also agree that placing the analyses of the Indo-Pacific Warm Pool (IPWP) and the Western Pacific Subtropical High (WPSH) in the Supplementary Information weakened the mechanistic narrative in the main text. Following your suggestions, we have substantially reorganized the relevant figures and analyses as follows.

1) Consolidation of the original Figure 5a–h

We have combined the original Figure 5a–h into a single comprehensive panel, now presented as Figure 5a. This revised panel provides a clearer and more direct visualization of the seasonal migration of the ITCZ across the three subregions while allowing the quantitative relationships between ITCZ position and OLR to remain prominent in the figure. Please see the revised Figure 5a and Figure R1 below.

2) Incorporation of the IPWP and WPSH analyses into the main text

We agree that the IPWP and WPSH are central to the proposed mechanism controlling atmospheric updraft and should therefore be presented in the main text. Accordingly, we have moved the relevant analyses from the Supplementary Information and reorganized them into a new Figure 6. This figure shows the spatial distributions of the IPWP and WPSH during the pre-monsoon and monsoon seasons, thereby providing more direct support for their roles in modulating atmospheric updraft across the Asian monsoon region. The corresponding post-monsoon analyses have been retained and consolidated in Supplementary Figure 11. Please see the new Figure 6 in the revised manuscript and Supplementary Figure 11 in the revised Supplementary Information.

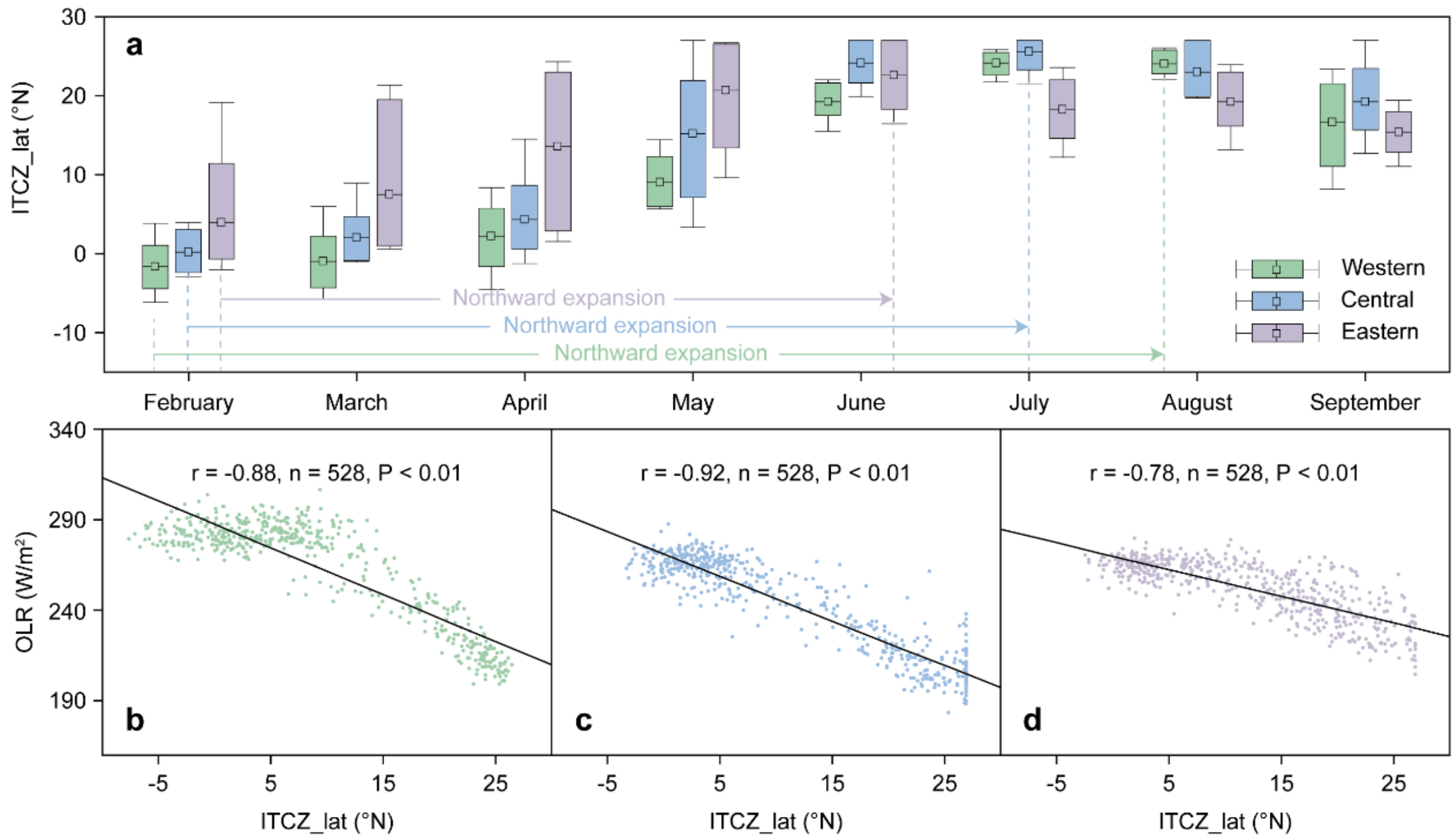

Figure R1. Intertropical Convergence Zone (ITCZ) northern boundary location within the respective longitude ranges of the three subregions, and correlations between outgoing longwave radiation (OLR) in these subregions and the corresponding ITCZ northern boundary location during 1979–2022. a ITCZ northern boundary location (ITCZ_lat) during the pre-monsoon and monsoon seasons. Western, Central, and Eastern represent the three subregions: Western (70–95° E), Central (95–105° E), and Eastern (105–125° E). In each box plot, the box represents the 15th–85th percentile range, and whiskers extend to the 5th–95th percentiles.

Inside each box, the mean is shown as both a hollow square and a horizontal line. **b–d** Correlations between OLR in the three subregions and ITCZ northern boundary location (1979–2022, all months combined): Western (**b**), Central (**c**), and Eastern (**d**).

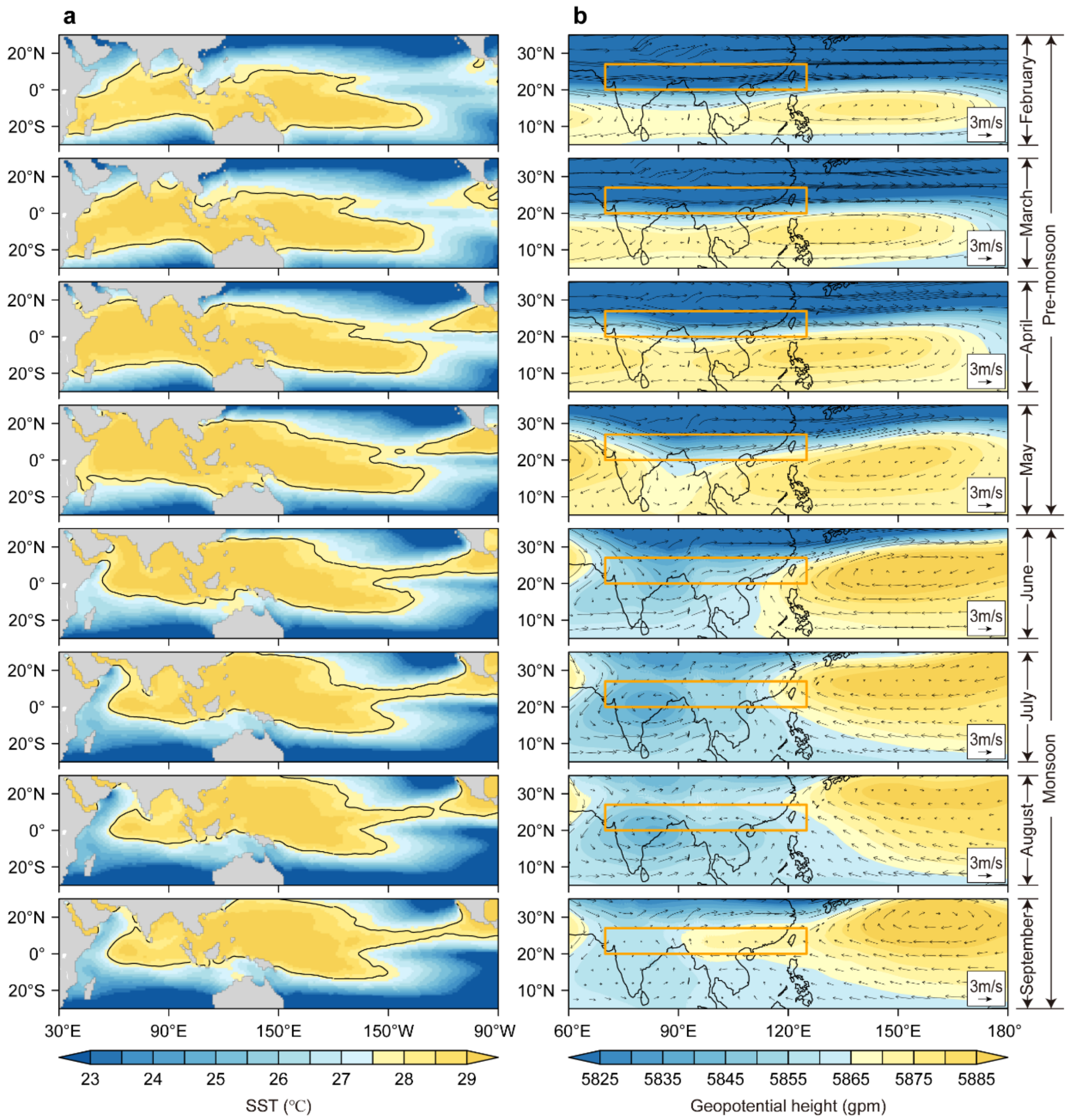

Figure R2. Spatial distributions of monthly sea surface temperatures (SSTs), 500 hPa geopotential height, and wind field over 1979–2022 during the pre-monsoon and monsoon seasons. a Spatial distributions of SSTs. The black line indicates the 28 °C SST, marking the extent of the Indo-Pacific Warm Pool. **b** Spatial distributions of 500 hPa geopotential height and wind field. Shading represents geopotential height, arrows indicate the wind field, and the orange rectangle marks the study region.

Discussion and outlook:

The authors main point, that atmospheric updrafts play an important role in Asian monsoon $\delta^{18}O_p$ variability, have important implications for a range of climate studies. While the authors acknowledge the implications for interpreting paleoclimate records, I think it is also worth discussing how updraft dynamics may explain biases in simulations of water isotope distributions in climate models. Could biases in model characterizations of convective updrafts explain offsets between isotope enabled models and observations? Furthermore, does this finding of updraft importance perhaps have implications for understanding drivers of other monsoon systems and their $\delta^{18}O_p$? I would appreciate some discussion of these points.

Re: We thank you for highlighting these important broader implications of our findings. In response, we have expanded the Discussion and Outlook section in two respects.

1) Atmospheric updraft as a potential source of bias in isotope-enabled climate models

We agree that biases in the representation of convective updrafts could contribute to discrepancies between simulated and observed precipitation isotope distributions. Although isotope-enabled General Circulation Models incorporate convective parameterizations, they may differ in how accurately they represent updraft strength, vertical moisture transport, condensation level, and associated isotopic fractionation. A direct evaluation of these model processes is beyond the scope of the present study; nevertheless, the observed relationship between $\delta^{18}O_p$ and atmospheric updraft provides a useful constraint for assessing whether isotope-enabled models adequately capture the isotopic effects of vertical atmospheric dynamics. We have added a brief discussion of this implication in L352–L356 of the revised manuscript.

2) Relevance to other monsoon systems

We also agree that the physical processes linking atmospheric updraft to isotopic

fractionation are unlikely to be unique to the Asian monsoon region. Similar dynamics may influence $\delta^{18}O_p$ variability in other major monsoon systems, including the African and South American monsoons. Although further observations and model analyses are needed to test the generality of this mechanism, our findings suggest that atmospheric updraft should be considered alongside moisture source and transport when interpreting precipitation isotope variability in other monsoon regions. We have added this broader perspective in Lines L356–L358 of the revised manuscript.

COMMENTS

(Reviewers' statements in black, and our response in blue)

Reviewer #6 (Remarks to the Author):

I would like to thank the authors for their detailed work in addressing my previous round of comments. I have no more additional comments and now recommend for publication.

Re: Thank you for your positive recommendation and for the thoughtful comments offered in the earlier review. We are pleased that our revisions have addressed the remaining concerns. We also appreciate the time and care in helping us improve the manuscript.

Review of manuscript NCOMMS-25-23387-T:

"*Reinterpreting Asian speleothem isotope records: the hidden role of atmospheric updraft*"

Note: Alongside the primary reviewer, this manuscript was co-reviewed by an ECR researcher as part of the Nature Communications initiative to facilitate training in peer review and to provide appropriate recognition for Early Career Researchers who co-review manuscripts. Hence, the feedback given in this report reflects the collective judgement of both reviewers.

GENERAL COMMENTS

In this manuscript, Zhang et al. compile $\delta^{18}O$ data from speleothems and rainwater samples across the Asian monsoon domain: to explore the extent to which atmospheric updraft could explain spatially conflicting trends in $\delta^{18}O$ signals, relative to established processes including 'rainout' and 'amount' effects. They suggest that their results pertain to a non-trivial influence of updraft as a driver of variations in $\delta^{18}O$ on both short (<1-year) and long- (>1000-year) timescales, and that their work underscores an important consideration for interpretation of speleothem $\delta^{18}O$ records in the future.

Overall, we believe this study will only be suitable for publication in Nature Communications once the following concerns have been addressed:

- The manuscript is written in a way that rests heavily on description of the figures, rather than explanation of how they can be interpreted. The reader is constantly referring to the figures in order to make sense of what the authors are describing in the text, and then these descriptions are often followed by 1-2 bold claims stating what they imply. However, we found none of these statements convincing in the absence of clear, mechanistic explanations for why the patterns were there in the first place. Perhaps the authors assumed that the reader would have a certain degree of prior knowledge in this field, but even if so, the narrative was incredibly hard to follow; to the point where we had to read certain passages several times to make sense of them. Moreover, the authors referred to a new "framework" for interpretation of $\delta^{18}O$ variability in speleothems, but we were hard-pressed to find a framework that could actually be applicable to future studies; beyond simply considering that updraft **may** be important to consider.
  We suggest that the authors make major revisions to the structure and narrative of the manuscript, where they…
  (a) Walk the reader through each analytical step carefully and thoroughly, and providing sufficient description of the processes, and phenomena, that could be underpinning the emerging patterns. These steps could then be more clearly linked to the research objectives stated at the beginning, and allow the reader to place the findings into a broader context.
  (b) Consider all relevant alternative explanations for their observations – to give the reader further confidence that their interpretations are well grounded, and not excessively deterministic.
  (c) Clearly link their observations and results to the main objective (and novelty) of the study, which we understood to be: to explore the extent to which atmospheric updraft controls the $\delta^{18}O$ signals encoded in speleothems across the Asian monsoon domain; relative to more established factors (e.g., rainout and amount effects).

- The lack of transparency regarding how datasets were compiled for use in this study. I found it surprising that, for a piece of research that rests so heavily on analysis of pre-existing datasets, very little information was given on the manner in which these records were identified, screened, and evaluated. The study makes no secret of the fact that there is huge diversity in the expressions of speleothem $\delta^{18}O$ signals across this domain, and so there is even greater need for a clear process of determining whether (or not) records were suitable for this work. Hence, we would like to see evidence for a clear, strategic procedure for data selection, and a means of quality-checking the datasets utilized in this study, which could be done by means of intra-study comparisons, data triangulation (e.g., cross-checking isotope measurements across different speleothems within the same system), and/or comparison of interpretations made from the same system.

- Frequent use of arbitrary boundaries and definitions. At several points in the manuscript, authors define spatial and temporal domains without clear justification, or rationale, for why these definitions were selected. This lack of justification for certain choices left me questioning several aspects of the study, and especially the interpretations made from the data. We suggest that where these boundaries are included, they are accompanied by 1-2 sentences justifying why they were chosen, and/or alongside the literature upon which these decisions were made.

- Presentation and aesthetic of figures. While we agree that personal taste plays a large role in figure creation, we can't help but feel as though these figures were assembled in a great hurry/as an afterthought, and some even seem unfinished. For example, there are multiple whisker plot figures, colour-coding is largely inconsistent across the figures, and many seem as though they have been merely copied and pasted from the software used to create them; again assuming that the reader already knows how to read them. For some journals this would suffice, however, papers in Nature Communications typically contain figures that not only look aesthetically pleasing, but are also accessible to both specialist and non-specialist readers. With this in mind, we suggest the authors consider (a) how their figures could be combined so not to repeat styles several times, and (b) inclusion of a conceptual, cartoon-style figure to help visualize their proposed mechanisms. Together, both would greatly improve the accessibility of the article to a broad audience.

- The extremely large number of supplementary figures. Having such a high number meant we were constantly switching back-and-forth between the manuscript and supplementary file, which made reading a lot more difficult. Moreover, we would argue that many of these figures did not add anything to the story and, in certain cases, obscured the key message of the paper.

DETAILED COMMENTS

Line 51: It would be good to have an indication of the timescales being considered in this study here; given that different timescales will require consideration of different mechanisms, signals, and processes.

Line 53: A new hypothesis for what? This sentence doesn't quite make sense in isolation, as it's not clear exactly what the "new" refers to. Is it (a) a new hypothesis for interpretation of speleothem $\delta^{18}O$ records, or (b) a new hypothesis for diverse expressions of monsoon intensity in speleothems? This should be made more clear.

Line 54: Are 'sites' and 'caves' not the same thing?

Line 57: "*isotope variations across **<u>the</u>** Asian monsoon region*."

Line 58: Authors say 'prolonged' - how long are we talking? Hours? Days? Weeks?

Line 59: If $\delta^{18}O$ signals are going to be differentiated using subscripts, there needs to be explicit definition of what these different subscripts refer to. Even in the abstract, and especially for a journal like NatComms where the readership is likely to be more broad, and not limited to subject specialists who would be familiar with this nomenclature.

Lines 65-66: This feels like an unsatisfactory end to the abstract, and excessively vague. As a reader, we want a clearer statement of what this 'new perspective' entails, how it is different to other perspectives that are currently available/proposed, and the implications of this revised appraisal of speleothem $\delta^{18}O$ records.

Line 73: 'Climate change' is a broad statement – more specificity is needed on which changes are of particular concern to this region. This will help to clearly set up why the work is novel, and valuable.

Line 74: On what timescales?

Lines 75-76: "*However, long-term instrumental records … scarce*." Please provide a reference.

Line 77: If $\delta^{18}O$ is going to be singled out, it needs to be made more clear on why this proxy is preferential over proxies such as trace elements, $\delta^{13}C$ etc... which can also provide valuable climatological information.

Lines 93-98: This paragraph is difficult to follow without a map or a more specific description of the locations within the Asian monsoon region. In addition, the authors consider a rather small area compared to the entire Asian monsoon region to argue for the uncertainties in the key controls on $\delta^{18}O_p$.

Line 98: Is Huanjiang a GNIP station as well?

Lines 99-100: Could it be possible for controls to be different under changing background conditions, or one considers decadal versus millennial timescales? Is there strong evidence for a single control in isolation?

Line 101: Spell out GNIP in full before abbreviating.

Line 102: Which "published sources" are the authors referring to? Please, provide the reference(s) for the published sources.

Lines 103-104: We would like to see a more detailed description of the workflow used to compile the datasets used in this study. For example:

- How was data sourced?

- How did the authors ensure that suitable records were not missed/overlooked?

- Were any datasets omitted, and if so, why?

We have little doubt that the authors conducted a thorough review of the literature in order to compile their records, but we think being transparent on the process used is important so it is clear the data used hasn't been 'cherry-picked'

Lines 104-105: This statement assumes prior knowledge of this specific model, which we are not sure all readers will have. Useful here would be a sentence or two explaining why this model was chosen, and what it offers. Also, the authors state that they used the IsoGSM2 model. Which model was used for the study, IsoGSM or IsoGSM2? Please add the provider of the model or a reference.

Lines 106-109: We like how the authors clearly lay out the discrete components of their study, although we think this could be written in a more sensical way. For example: "*Our study had four key objectives: (1) to identify...., (2) to evaluate..."* - and so on. This gives better opportunity for the reader to understand the 'why' behind this study. We could even go so far as to say that there is no need for more than two objectives….

Lines 112-113: What renders a site "primary" versus "ancillary"? How would the two be differentiated? The caption to Fig. 1 also refers to 34 ancillary precipitation sites. The abstract and the introduction refer to a total of 46 precipitation sites (12 primary + 34 ancillary). Are we right in assuming that 34 is the correct number?

Line 114: What time period do all locations cover? We suggest including this information in the text or at least making a reference to Table S1.

Lines 116-117: What is this distinction based on? An existing study? Instrumental data?

Line 118: Or “eastern <u>domain</u>”?

Line 120: Which $\delta^{18}O_p$ values are shown in Supplementary Fig. 2 (Mean, Median)? In addition, Supplementary Fig. 2 refers to East Asian monsoon and South Asian monsoon instead of “eastern part” (L. 118) and “western part” (L. 120). Please be more consistent in using technical terms.

Lines 124-126: This is a circular point: if the line between east and west regions has already been defined as 100° east, then the results don't "indicate" the difference, they simply support a pre-defined idea that the east and west are seasonally different. Thus, the source of this hypothesis should be clearly specified. Also, what would happen if you shifted this band? Would the same clear difference still exist? Also, we note that in line 116 the authors are referring to eastern and western parts of the region, when talking about the 100°E dividing line. Yet here (124), the authors are referring to the East Asian monsoon and South Asian monsoon region. Please be more consistent.

Line 128: Again, it needs to be made much clearer how well defined this distinction is already, and thus how much new knowledge this study is contributiing to this idea. If this phenomenon is already well established/characterized, what is the purpose of re-examining it here? We don't doubt it was done for a reason, but this reason is not clear from the text as-written.

Lines 130-131: Hasn’t this already been done in the previous section? Perhaps these two sections could be merged, to improve the narrative flow.

Lines 134-135: Are these definitions based on established literature?

Line 136: What correlation test is this referring to? (e.g., Pearson's, Spearman's...) Also, please give the statistical value.

Line 138: “*Additionally, previous studies…*” Which studies? Please add reference.

Lines 143-144: Not sure this point has been sufficiently "fleshed out". At the very least, we would like to see a couple of sentences where the authors consider <u>why</u> some records show a negative relationship, and some show a positive. Even if there is no conclusive answer, this should still be considered.

Lines 145-147: Why? Useful here would be a clear link back to the research objectives set out at the beginning, and description of the key characteristics of the pre- and intra-monsoon climate system over East Asia.

Line 154: Is Guilin one of the 12 primary precipitation sites. There is no reference in the previous text or Figure 1. Where is Guilin located in the map (Fig. 1). Why is Tengchong in brackets? According to Figure 1, Tengchong and Kunming are two different sites about 430km apart.

Line 155: We suggest improving Figure 2 by making it clearer for which precipitation sites the authors are referring to the eastern or western region.

Line 157: “*middle*”; Please be more specific.

Lines 159-161: We cannot fully agree with the observation made by the authors here. Unfortunately, the data for **Figure 2** are not included in the source file for a more detailed examination of the spatial evolution of $\delta^{18}O_p$. However, just by assessing and comparing Figure 2e-h, it appears that $\delta^{18}O_p$ values in both the east and west remain similar over time. The strongest change in $\delta^{18}O_p$ values during the monsoon period occurs in the middle of the transect around the dividing line of ~100°E ± 5°rather than at the margins. We cannot see a gradual increase in $\delta^{18}O_p$ values in the eastern part of the transect, the oscillation of the values does not allow a clear statement about the trend over time. The mean of $\delta^{18}O_p$ values for the eastern sites are almost identical in July and September. The IsoGSM-simulated δ18Op data (*Supplementary Fig. 7*) also shows the strongest change in $\delta^{18}O_p$ values in the middle of the transect, while the western and eastern regions stay fairly stable over time.

Lines 162-167: We are not entirely clear still how clearly this passage relates links back to the primary focus of this study, and so sentence or two summarising what these findings mean, and why they are important, would be really helpful.

Line 168: This section seems to lack a coherent narrative flow, and as a result is hard to follow.

Lines 170-177: Have the authors familiarized themselves with the deuterium excess in their precipitation data? To what extent does the deuterium excess in the precipitation data vary spatially and temporally? Is there a trend between the eastern and western parts of the Asian Monsson region similar to the $\delta^{18}O_p$ data? Also, what are “moisture flux fields”?

Line 171: The term “moisture channel” should be described for non-specialist readers.

Line 173: Seems strange to be opening this (important) section with reference to a supplementary figure...

Lines 178-187: This paragraph repeats the key points made in the paragraph above, and so I suggest merging the two for brevity.

Lines 188-191: This does not make sense, and is hard to follow.

Line 194: What aspects of the SASM? Moisture sources? Strength? Something else...?

Lines 196-197: Citation needed.

Lines 203-205: Repeating "pattern" three times in this sentence.

Line 206: Further evidence for what? Sufficient detail should be given in the opening line of any paragraph.

Line 207: Is decadal scale really long-term? And how long-term are we talking about here? Centuries? We also suggest providing an additional map to show the location of the five sites. This will provide information how these sites are located in relation to the different monsoon regions and the precipitation sites.

Lines 211-213: Please provide a reference.

Line 212: Where has this proxy been established as a convection indicator? Citation needed.

Line 218: Or “*alongside*”? Also, please provide a reference.

Lines 219-220: What constitutes a "continuous" event? Careful with this definition, as 'continuous' can be overly broad and obscure.

Line 221: Even if these processes are described on, the reader should be given an impression of what kinds of processes are being referring to.

Lines 232-233: Helpful would be to delineate between 'upward motion' and 'updraft' - a non-specialist reader may assume they are the same thing.

Line 248: “…*migrating westward*.” This cannot be clearly identified by the Figures. In Figure 3h vertical velocities and OLR tend to be higher in the western region. I suggest calling it a westward expansion of low vertical velocities and OLR rather than a migration. The spatial area of low vertical velocities and OLR is increasing westward over time.

Lines 249 -250: This observation does not match the changes in vertical velocities and OLR from July to August, showing a decrease in these values.

Lines 250-255: A V-shape spatial pattern of vertical velocity and ORL requires increasing velocities in the western transect as well. However, this contradicts the statement made in line 247-249, indicating low vertical velocities and OLR values in the western transect during the monsoon period. A clear V-shape spatial pattern of vertical velocity and ORL as it is described here (high values at the margins, low values in the middle of the transect) can only be observed during September. The authors clarify that the V-shaped pattern indicates a stronger updraft in the center and weaker updraft on the eastern and western margins (line 251-252). However, the months with the strongest V-pattern in the atmospheric updraft (September and to a certain degree June; Fig. 3e & h) are the months with the weakest V-pattern in the $\delta^{18}O_p$ values (Fig. 2e & h). By contrast, the V-shape of $\delta^{18}O_p$ values is strongest pronounced in July and August (Fig. 2f & g) associated with more consistent atmospheric updraft intensity (Fig. 3f & g) from the middle to the margins of the transect. This calls into question the subsequent statement that atmospheric updraft is driving the V-shaped spatial pattern in $\delta^{18}O_p$ (l. 253f).

Furthermore, there is no concrete statement on how atmospheric updraft affects $\delta^{18}O_p$. Does $\delta^{18}O_p$ decrease with increasing atmospheric updraft or vice versa? This requires a more detailed discussion.

Line 267: Isn't this just another version of the statement at the beginning of the section?

Line 268: Which correlation test was used, and why?

Lines 284-285: We are not entirely convinced based on the presentation of results here. The authors describe the figure, but don't offer any mechanistic or conceptual explanations for the observed trends. Please be more specific, how exactly atmospheric updraft affects $\delta^{18}O_p$ values. Stronger atmospheric updraft cause more negative $\delta^{18}O_p$ values or the other way around?

Lines 292-293: In what sense? Further clarification is needed here.

Lines 297-312: There are a series of statements made throughout this entire paragraph without any reference being made. Since these are general observations and relationships, we suggest providing appropriate references.

Lines 315-316: Why does this play a key role?

Line 323: Why not "*pre-monsoon period*", like in the previous sections to keep consistent pronunciation and improve readability.

Line 330: Are there no references to published literature that would be relevant here? Also, is this not an excessive number of supplementary figures to be referring to?

Line 349: Please provide a reference to the state of monsoon intensity. June to August = monsoon period

Lines 358-362: This paragraph requires further discussion. The extent to which the IPWP affects convection across the transect during the monsoon period is not sufficiently discussed. What "*combined influence*" are the authors referring to? How can a gradually westward strengthening convection result in weaker convection at the western margins?

Lines 360-361: Rather than a ton of supplementary screen-grabs from the analysis, we think the manuscript would benefit hugely from inclusion of a conceptual cartoon/diagram that clearly presents these mechanisms at work. This would help the reader to visualise what is going on, and how seasonal changes in updraft dynamics could explain the observed seasonal trends in precipitation $\delta^{18}O$.

Line 378: We think this is overly broad, and the question would be better cast as: "*What aspects of monsoonal variability do Asian $\delta^{18}O$ signals capture, and how could these signals be affected by geography?*"

Lines 382-383: In the instrumental data? Or the proxy data?

Line 392-394: Misleading argument. According to Fig. 1, there is no cave located at the western margin. The most western cave is located at ~93°E, which corresponds to the middle of the transect rather than the margins.

Lines 403-404: We think this is a bold statement that is not entirely backed up by the data. From what we understand, temperature differences between the surface and higher altitudes (e.g., lapse rate) can have an effect on updrafts such that greater differences lead to stronger updrafts - due to increased buoyancy. Also, what is to say that orographic lifting couldn't also affect precipitation? We think because the topography of Asia is so diverse, altitudinal and topographic effects need to be explored in much closer detail before all processes aside from updraft can be ruled out.

Line 407: Spatial pattern of what?

Line 409: We are not entirely sure what this "framework" is...By definition, a framework is a conceptual structure upon which ideas, hypotheses, and methods can be built. However, no such framework is presented in this study, and as a researcher, we would be unclear on how to use the results of this work to inform our interpretation of a speleothem record; beyond simply considering that updraft may be important. So, either edit the wording of this statement, or include a more systematic, step-wise description of how this study can be build into those in future.

Lines 410-411: But only on timescales relevant to the Late Pleistocene/Holocene right?

Line 628: Describe what these latitude and temperature effects are.

Line 744: There are 14 caves marked on the map, whereas the main text and caption refer to 13 caves. Please add numbers and names of the caves.

Line 809: ***Figure 6*** feels to have been assembled in a great hurry, and as a result appears quite messy. Why not colour-code the curves to match the whisker plots below? Also, we think U-Th ages for the featured speleothems should be included if one seeks to compared time-resolved changes in different datasets.

The revised manuscript is very well written and the authors have done a wonderful job in answering my concerns throughout the whole review processes.

I maintain to hold a little doubt, as the authors do show that the two are correlated and therefore not statistically distinguishable. The absence of a major difference between AIC values of the two models illustrates that inclusion of both does not improve the model, simply because they represent the same axis of variation. Nevertheless, I believe the authors have taken a good effort to try to show that precipitation amount does not explain the whole variability seen across the transect, and that atmospheric updraft motion is the primary control. I expect that vertical updraft is likely the primary mover, with rain amount acting as a secondary component of the same process. Whether this acts as a new understanding of the mechanism of the traditional *amount effect*, or is an independent effect, is a semantic debated, and the minor controls on rainfall $\delta^{18}O$ may be revealed over future research.

I believe this manuscript offers an interesting insight into spatial patterns of rainfall $\delta^{18}O$ in Asia, and I recommend the manuscript for publication following very minor corrections attached below.

Minor corrections

Line 232-233: Please state whether these negative gradients are significant

Line 236: Again, are these significant?

Line 242: report p value with the correlation coefficient.

Line 253: Not sure why positive is in quotation marks. If it's not significant, there is no significant correlation and is therefore not quantifiably positive. The authors could revise this sentence from:
"The results show that correlations change over time from "positive" to negative (Fig. 4a-d, 4i-l)"…

To something like:

"The results show that correlation is time transgressive, with only significant negative correlations occurring in in the monsoon seasons"….

Also note that figure 4 does not provide the months of the correlation plots.

Line 303: "Hence, the combined influence of the ITCZ, IPWP, and WPSH results in a significantly stronger atmospheric updraft in the East Asian monsoon region than in the South Asian monsoon region during the pre-monsoon season, particularly in May (Fig. 6a)."
Is this a test for statistical significance, i.e., t-test of updated in east vs west? If not, "significant" should be replaced with "substantially". If it is, please report p-values.
